# Brazilian Society of Rheumatology – 2025 recommendations on vaccination in immune-mediated rheumatic diseases

**DOI:** 10.1186/s42358-026-00520-8

**Published:** 2026-01-23

**Authors:** Gecilmara Cristina Salviato Pileggi, Vitor Alves Cruz, Ana Cristina de Medeiros-Ribeiro, Ana Karla Guedes de Melo, André Gustavo Cunha Trolese, Anna Carolina Faria Moreira Gomes Tavares, Cristiano Augusto de Freitas Zerbini, Erika Biegelmeyer, Flávia Maria Matos Melo Campos Peixoto, Gilda Aparecida Ferreira, Joana Starling de Carvalho, Ketty Lysie Libardi Lira Machado, Lilian David de Azevedo Valadares, Marcelo de Medeiros Pinheiro, Natália Sarzi Sartori, Priscila Dias Cardoso Ribeiro, Rejane Maria Rodrigues de Abreu Vieira, Ricardo Machado Xavier, Sandra Lúcia Euzébio Ribeiro, Vanessa de Oliveira Magalhães, Viviane Angelina de Souza

**Affiliations:** 1 Universidade Federal de São Paulo – Escola Paulista de Medicina (UNIFESP/EPM), São Paulo, Brazil; 2 Universidade Federal de Goiás (UFG), Goiânia, Brazil; 3 Universidade de São Paulo (USP), São Paulo, Brazil; 4 Universidade Federal da Paraíba (UFPB), João Pessoa, Brazil; 5 Universidade Federal de Minas Gerais (UFMG), Belo Horizonte, Brazil; 6 Centro Paulista de Investigação Clínica, São Paulo, Brazil; 7 Universidade Federal do Espírito Santo (UFES), Vitória, Brazil; 8 Universidade Federal de Pernambuco (UFPE), Recife, Brazil; 9 Universidade Federal do Rio Grande do Sul (UFRGS), Porto Alegre, Brazil; 10 Universidade de Fortaleza (UNIFOR), Fortaleza, Brazil; 11 Universidade Federal do Amazonas (UFAM), Manaus, Brazil; 12 Universidade Federal de Juiz de Fora (UFJF), Juiz de Fora, Brazil; 13 Faculdade de Medicina, Departamento de Clínica Médica, Universidade Federal de Goiás, Goiânia, Brazil

## Abstract

**Background:**

Patients with immune-mediated rheumatic diseases (IMRD) are at increased risk for infections due to both disease-related immune dysregulation and immunosuppressive therapy. Despite the benefits of vaccination, immunization rates in this population remain suboptimal, often due to concerns about safety, efficacy, and their potential for inducing disease flare. Regional-specific guidelines are necessary to address the particular epidemiological issues and aspects of the healthcare systems, especially in countries like Brazil.

**Objective:**

To provide updated, evidence-based, and nationally relevant recommendations on vaccination in adult patients with IMRD in Brazil, focusing on immunogenicity, safety and disease activity outcomes.

**Methods:**

A multidisciplinary task force from the Brazilian Society of Rheumatology conducted a systematic review and meta-analysis of studies addressing eleven clinical questions related to vaccine safety and efficacy in IMRD. Studies were selected using predefined PICO criteria. Risk of bias was assessed using JBI tools, and the certainty of evidence was evaluated with the GRADE approach. Statements were developed and submitted to a Delphi-based voting process; consensus was achieved if ≥80% of the panelists voted “agree” or “strongly agree” for all the statements.

**Results:**

Eleven recommendations were developed based on a systematic review of the literature, with meta-analyses conducted when appropriate. Inactivated vaccines demonstrated a favorable safety profile, with low flare rates and no significant increase in disease activity, even under immunosuppression. Live attenuated vaccines, including yellow fever, were considered safe when administered according to timing protocols. Immunogenicity may be reduced in patients receiving methotrexate, mycophenolate, corticosteroids, rituximab, and JAK inhibitors, although this does not appear to compromise clinical protection in most cases. Temporary treatment interruption was associated with improved immunogenicity in selected contexts, but without consistent evidence of clinical benefit and with potential risks related to disease control. Specific guidance was provided for influenza and hepatitis B vaccination, as well as for prioritizing vaccination before initiating immunosuppression whenever feasible. Statements also addressed the approach to revaccination and post-vaccination serologic testing. Despite the overall very low to moderate certainty of evidence, most recommendations reached strong consensus (≥80% agreement). Shared decision-making and individualized strategies were emphasized across all scenarios.

**Conclusion:**

These recommendations offer tailored guidance for improving vaccination strategies in IMRD patients in Brazil. Given the heterogeneity of evidence, clinical decisions should be individualized, considering disease activity, treatment regimen, vaccine availability, and patient preferences. Shared decision-making is essential in all scenarios to enhance vaccine uptake and align preventive care with patient-centered management.

**Supplementary Information:**

The online version contains supplementary material available at 10.1186/s42358-026-00520-8.

## Introduction

Patients with immune-mediated rheumatic disease (IMRD) are at increased risk of infections due not only to immunological dysregulation of the disease itself, but also to the frequent use of immunosuppressive therapies. Infectious complications remain a leading cause of morbidity and mortality in this population. Indeed, studies have shown that patients with rheumatic diseases are significantly more susceptible to developing severe infections than the general population, which often leads to increased hospitalization rates, emphasizing the need for effective preventive measures. Vaccination plays a critical role in reducing the incidence and severity of vaccine-preventable infections [1].

Despite the clear benefits of immunization, vaccine uptake among IMRD patients remains suboptimal. Several barriers contribute to low vaccination rates, concerns about the safety and efficacy of vaccines, and limited awareness of immunization guidelines for immunosuppressed individuals. Addressing these barriers requires a proactive approach by rheumatologists and other specialists, integrating vaccination assessments into routine clinical practice and engaging patients in shared decision-making [2].

The immune response to vaccines in patients with IMRD may be reduced or impaired by the immunosuppressive therapies. These agents, including glucocorticoids, conventional synthetic disease-modifying antirheumatic drugs (csDMARDs), biologic DMARDs (bDMARDs), and targeted synthetic DMARDs (tsDMARDs), which are essential for disease activity control, but may impair the development of an adequate vaccine-induced immune response [3].

The extent of vaccine response impairment varies according to the type and duration of immunosuppression. Methotrexate (MTX) and leflunomide (LEF), commonly used in IMRD treatment management, have been associated with a low to moderate reduction in antibody titers post-vaccination. In contrast, high-dose glucocorticoids and CD20-targeted therapies can lead to high impairment of immunogenicity, necessitating alternative strategies to optimize vaccine efficacy [4].

Given these challenges, appropriate timing of vaccination is critical. Ideally, patients should complete recommended vaccinations before initiating immunosuppressive therapy to maximize immune response. When this is not feasible, strategies such as booster doses, modified vaccine schedules, or temporary suspension of specific immunosuppressants in stable patients may improve immunogenicity. A patient-centered approach, involving shared decision-making between rheumatologists and patients, is essential to balancing vaccination efficacy with disease control [5].

Although general vaccination guidelines exist for the overall population, patients with IMRD require tailored recommendations that consider the impact of immunosuppression on vaccine safety and immunogenicity. While international guidelines from European Alliance of Associations for Rheumatology (EULAR) and American College of Rheumatology (ACR) provide a solid foundation, they are largely based on data from Europe and North America and may not fully address regional epidemiological patterns or healthcare infrastructure. [6] In Brazil, the development of national recommendations is especially relevant due to the high burden of vaccine-preventable infections, widespread use of immunosuppressive therapies, and endemic diseases such as tuberculosis and hepatitis B, which demand context-specific vaccination strategies [7].

Furthermore, disparities in vaccine implementation and accessibility, underscore the need for standardized national recommendations. Studies indicate that adherence to vaccination guidelines is often inconsistent among healthcare providers, leading to missed immunization opportunities. Establishing a structured, evidence-based national framework can improve vaccination coverage, ensuring optimal protection of IMID patients against preventable infections [8].

Over the past decade, international guidelines for vaccination in patients with IMRD - such as those issued by EULAR (2019) and ACR (2022) - have expanded their scope, incorporating evidence from observational studies, clinical trials, and expert consensus. These recommendations have progressively addressed the heterogeneity of immunosuppressive regimens, timing of vaccine administration, and specific vaccine platforms, including mRNA and recombinant vaccines. Nonetheless, important gaps remain regarding optimal vaccination strategies for patients under a high degree of immunosuppression, those with rare IMRD, and populations underrepresented in clinical studies, underscoring the need for continued research and regional adaptation [9–11].

Another critical barrier to vaccination adherence in IMRD patients is the inconsistent implementation of immunization guidelines by healthcare providers. Surveys indicate that rheumatologists and other specialists may hesitate to recommend certain vaccines due to uncertainties about immunogenicity in immunosuppressed patients or fears of adverse effects [12]. This lack of standardization, combined with logistical challenges—such as limited vaccine availability in specialized settings and the need for coordination with primary care—leads to missed opportunities for preventive care. Addressing these gaps requires a multifaceted approach, including educational initiatives for both patients and physicians, systematic integration of vaccination assessments into routine rheumatologic care, and improved communication between specialists and primary care providers. Shared decision-making is key to overcoming vaccine hesitancy, allowing individualized discussions about risks, benefits, and timing [13].

Given the complexity of vaccination in patients with IMRD, an updated, evidence-based consensus is essential to guide clinical practice. While international guidelines provide a strong foundation, the epidemiological profile, healthcare infrastructure, and vaccine availability, it is necessary to develop tailored recommendations to the Brazilian scenario. This document synthesizes the latest scientific evidence with expert consensus opinion to offer practical guidance on optimizing immunization strategies in this population. The main objective was to outline specific recommendations, focusing on vaccine efficacy, safety, and administration strategies to enhance protection while maintaining disease control.

## Methods

### Task force

The Committee of Endemic and Infectious Diseases of the Brazilian Society of Rheumatology conducted this project. The task force consisted of 19 rheumatologists.

These members drafted the clinical questions (CQ) that guided the literature review, selected the studies, extracted the data, analyzed the results, and drew up the recommendations. They also constituted the voting panel (VP), that comprised all the experts involved in the task force who evaluated the proposed recommendations and voted according to their experience. The systematic review protocol was not prospectively registered in a public database; however, all methodological steps were predefined and conducted in accordance with PRISMA and GRADE recommendations.

### Establishing key principles and clinical questions development

Initially, the task force established the following CQ that should guide the literature review, as follows:

**CQ1.** What is the risk of symptom flare-up or worsening of patients with IMRD after vaccination with inactivated vaccines?

**CQ2**. What is the risk of symptom flare-up or worsening of patients with IMRD after vaccination with live attenuated vaccines?

**CQ3.** What is the impact of the cs-DMARDs (MTX, LEF, sulfasalazine (SSZ), hydroxychloroquine (HCQ)) use on the response to inactivated vaccine in patients with IMRD?

**CQ4.** What is the impact of the immunosuppressants (azathioprine (AZA), mycophenolate mofetil (MMF), cyclosporine (CsA), cyclophosphamide (CYC)) use on the response to inactivated vaccines in patients with IMRD?

**CQ5.** What is the effect of DMARDsb use (except rituximab) on the response to inactivated vaccines in patients with IMRD?

**CQ6.** What is the impact of rituximab (RTX) use on the response to inactivated vaccines in patients with IMRD?

**CQ7.** What is the impact of corticosteroids use on the response to inactivated vaccines in patients with IMRD?

**CQ8.** What is the impact of janus kinase inhibitors (JAKi) use on the response to inactivated vaccines in patients with IMRD?

**CQ9.** Is there a benefit to vaccine response by temporarily stopping MTX, mycophenolate, JAKi before or after vaccination?

**CQ10.** Is there a difference in the immune response between conventional (tri- and tetravalent) and high-dose influenza vaccines in patients with IMRD)?

**CQ11**. Is there a difference in the immune response between conventional (tri- and tetravalent) and high-dose hepatitis B vaccines in patients with IMRD)?

### Literature search

*To gather relevant evidence for the 11 clinical questions (CQs), literature search strategies were designed based on acronyms PICO:* (P) population of interest for this study, (I) the specific focus of each question (intervention, treatment or complementary investigation), (C) appropriate comparators, and (O) the most relevant outcomes.

### Eligibility criteria and study selection

The eligibility criteria varied according to the CQ, involving patients > 18 years, with a diagnosis of an IMRD, mainly chronic inflammatory arthritis (rheumatoid arthritis and spondyloarthritis), systemic lupus erythematosus, systemic sclerosis, autoimmune myopathies, Sjogren’s disease, mixed connective tissue disease and systemic vasculitis. The medications included (Table 1) glucocorticoids, immunosuppressive drugs, synthetic, biological or targeted synthetic disease-modifying antirheumatic drugs (DMARDs), comparing exposed and unexposed populations. We included cross-sectional, cohort and case-control, randomized or non-randomized clinical trials.

**Table 1:** Immunosuppressive agents available in Brazil for the treatment of patients with immune-mediated rheumatic diseases

Class	Drugs
Immunosuppressants	Azathioprine, calcineurin inhibitors, mycophenolate mofetil, cyclophosphamide
csDMARDs	Methotrexate, leflunomide, hydroxychloroquine, sulfasalazine
bDMARDs TNF inhibitors	Infliximab, etanercept, adalimumab, golimumab, certolizumab pegol
bDMARDs non-TNF inhibitors	Rituximab (anti-CD20), belimumab (anti-BlyS), anifrolumab (anti-IFN1), abatacept (anti-CD28), tocilizumab (anti-IL6r), secukinumab (anti-IL17), ixekizumab (anti-IL17), guselkumab (anti-IL23), risankizumab (anti-IL23), ustekinumab (anti-IL12/23), vedolizumab (anti-integrins)
tsDMARDs	Tofacitinib, baricitinib, upadacitinib

Exclusion criteria encompassed studies involving pediatric populations ( < 18 years), non-IMRD autoimmune conditions, case reports, narrative reviews, conference abstracts, or studies with insufficient data for analysis.

Flare or worsening was defined as the onset or intensification of clinical symptoms, laboratory markers of inflammation, or the need for treatment escalation, as reported by the original studies included in the review. Definitions varied slightly across studies but typically encompassed increased joint pain or swelling, elevated acute-phase reactants, and/or the requirement for dose adjustment or initiation of immunosuppressive therapy.

The impact of the cs-DMARDs use on the response to inactivated vaccine in patients with IMRD was evaluated based on immunogenicity outcomes. Vaccine response was defined as seroconversion, seroprotection, or a significant increase in antibody titers post-vaccination, according to the criteria established in each individual study. Most studies considered a ≥ 4-fold increase in antibody levels or reaching protective antibody thresholds as indicative of an adequate response.

The present recommendations evaluated immunogenicity, safety, and potential impact on disease activity of inactivated and attenuated vaccines in patients with IMRD. Among the inactivated vaccines, were assessed influenza (seasonal and pandemic A/H1N1), pneumococcal (both polysaccharide and conjugate formulations), hepatitis B, Human papillomavirus vaccine (HPV vaccine) and the recombinant adjuvanted herpes zoster vaccine. Regarding live attenuated vaccines, only the yellow fever vaccine (17DD strain) was systematically analyzed. Each recommendation considered vaccine-specific evidence and stratified outcomes by immunosuppressive therapy and disease subtype, when applicable.

Table 1 provides a classification of immunosuppressive drugs available in Brazil for treating immune-mediated rheumatic diseases (IMRD), grouped by class (conventional synthetic, biological, and targeted synthetic DMARDs). Source: adapted from Gasparin AA, et al., 2023.

This systematic review and meta-analysis were based on recommendations from the Cochrane Guidelines for Systematic Reviews and was written according to Preferred Reporting Items for Systematic Reviews and Meta-Analyses (PRISMA) [14, 15].

#### Search strategy

In order to identify the studies, we searched four independent databases to perform the sensitive literature search: MEDLINE, EMBASE; Central (by Cochrane Library) and Latin American and Caribbean Health Science Information (LILACS). Additionally, we searched for ongoing reference lists of included studies and systematic reviews.

There was no language, date, document type, publication status or geographic restriction for inclusion of records. The last search was conducted in July, 2024. Descriptors were identified in Medical Subject Headings (MeSH), Descriptors em Ciências da Saúde (Decs) and Embase Subject Headings (Emtree). The search strategy was adapted based on descriptors in each database and are presented in the supplementary material.

#### Eligibility criteria

We included studies that evaluated patients with IMRD that have been vaccinated with inactivated or attenuated live vaccines, according to the CQ, and assessed the outcomes.

Study selection and data extraction

Electronic search results from defined databases were uploaded to the Rayyan Qatar Computing Research Institute [16].

A separate PRISMA flowchart was developed for each clinical question, reflecting independent search strategies and study selection processes, as detailed in the supplementary material.

#### Study selection and data extraction

Were independently performed by two investigators. A third reviewer resolved any disagreements. For duplicate records, only the most recent one was included. Authors initially screened titles and abstracts. Subsequently, they assessed each study to determine whether it met inclusion criteria.

We extracted data on study design (reference, location, title, journal, study design), participant characteristics (disease, drugs used, number, sex), intervention (inactivated vaccine, follow-up) and outcomes characteristics (type evaluation, symptom flare-up and worsening of IMRD disease activity in each group: vaccine use and not and/or control).

#### Quality assessment

Two investigators independently assessed the risk of bias in the selected studies according to the Joanna Briggs Institute (JBI) checklist 14. The percentage of risk of bias was calculated using the number of “yes” (Y) answers selected in the checklist. Questions with “not applicable” (NA) answers were not considered in the calculation. Methodological quality was classified using the following categories: low (scores up to 49.0%), moderate (scores between 50.0% and 70.0%), and high (scores above 70.0%) [17].

The overall certainty of the body of evidence was rated by using the Grading of Recommendations Assessment, Development and Evaluation (GRADE) approach, considering overall risk of bias, consistency of effect, imprecision, indirectness and publication bias to assess the certainty of the body of evidence [18]. If there were serious concerns in any of these domains, we rated down the quality of evidence.

#### Statistical analysis

Forest plots were used to visually assess the combined estimates and their corresponding 95% confidence intervals. Heterogeneity was evaluated using the Q statistic (with a significance level of *p* < 0.1) and the I^2^ statistic, with values above 50% indicating substantial heterogeneity and above 75% indicating high heterogeneity. A random-effects model was applied in the presence of moderate to high heterogeneity.

In a meta-analysis of prevalence, when the estimate for a study tends toward either 0% or 100%, the variance for that study moves toward zero. As a result, its weight is overestimated in the meta-analysis. Therefore, we conducted the meta-analysis with prevalence estimates that had been transformed using the raw proportions (PRAW) method with random effect. The final combined result and 95% confidence intervals were back transformed for ease of interpretation. Subgroup analyses were performed by type study.

For comparison groups, we opted to summarize the evidence using rheumatic disease and control. Treatment effects were expressed as risk ratios (RRs), as all outcomes were binary. Pooled RR were calculated using random effects models with the Dersimonian and Laird estimator and the Mantel-Haenszel method, as clinical heterogeneity was expected.

Furthermore, Hedge’s g standardized mean difference (SMD) was used as a simple measure to estimate the differences in the mean antibody concentrations before and after vaccination. The SMD and confidence interval were obtained using the random-effects model according to the heterogeneity among the studies (HIGGINS; THOMPSON, 2002).

Analyses were performed in the R studio software, version 4.1.0 (R: A Language and Environment for Statistical Computing, Vienna, Austria), by using the ‘Meta’ packages, versions 5.0–0. P-value < 0.05 was defined as statistically significant.

The supplementary material (Appendix 1) provides details on the search strategy for each CQ, the selection and inclusion stages of the studies following the recommendations of the Preferred Reporting Items for Systematic reviews and Meta-Analyses (PRISMA), the included and excluded studies and the risk of bias for each of them.

#### Consensus building

After analyzing the evidence presented in the 11 CQ, the task force drew up the 11 recommendations.

Following the Delphi Method, these statements were sent by email to each member of the VP via a Google® Form for individual and anonymous voting [19].

For each of the 11 recommendations, members should indicate their level of agreement (Table 2): 1. Strongly disagree; 2. Disagree, 3. Neither agree nor disagree (neutral); 4. Agree; and 5. Strongly agree [20].

**Table 2:** Grading of the certainty of evidence using the GRADE approach

Certainty of evidence	Interpretation
High	Very confident that the true effect lies close to that of the estimate of the effect.
Moderate	Moderately confident in the effect estimate; the true effect is likely to be close to the estimate, but there is a possibility that it is substantially different.
Low	Limited confidence in the effect estimate; the true effect may be substantially different.
Very low	Very little confidence in the effect estimate; the true effect is likely to be substantially different from the estimate.

Consensus was reached when the VP voted 4—agree—or 5—strongly agree at least 80% of all scenarios.

To support their decisions, all members of the VP also received an evidence report that summarized the entire process of collecting and analyzing the evidence obtained in the literature review.

After the voting process, the task force drafted the manuscript with the relevant recommendations. The drafted manuscript was sent to all task force members for approval before submission.

Additional details on search strategies, individual study characteristics, risk of bias assessments (using JBI tools), and specific PICO formulations for each clinical question are available in the supplementary material. Tables summarizing the included studies and risk of bias assessments can be found in the Supplementary Tables and Appendix documents.

Table 2 describes the GRADE approach (Grading of Recommendations, Assessment, Development, and Evaluation) used in this document to assess the certainty of the evidence. Ratings include: high, moderate, low, and very low. These reflect the authors’ confidence that the effect estimate is accurate. Factors that may lower the quality include risk of bias, inconsistency, indirectness, imprecision, and publication bias.

## Results

### Statements and recommendations

The present document provides recommendations on vaccination in patients with immune-mediated rheumatic diseases (IMRD), based on a comprehensive synthesis of evidence from systematic reviews and meta-analyses (Table 3). Given the variable quality of the studies—many being observational, heterogeneous in design, and conducted across different geographic and clinical contexts, most recommendations are conditional. Clinical decisions must therefore be individualized, considering the patient’s specific characteristics, underlying disease, epidemiological settings, immunosuppressive treatment and access to vaccines.

**Table 3:** Summary of the recommendations issued by the Brazilian Society of Rheumatology on vaccination in immune-mediated rheumatic diseases (IMRD)

Recommendation	Certainty of Evidence (GRADE)	Level of Agreement (LOA)
1) Inactivated vaccines are safe in patients with IMRD and should not be delayed due to concerns of flare. Shared decision-making remains essential.	Very Low	100%
2) Live attenuated vaccines appear to be safe in patients with IMRD. Flare rates and vaccine-related infection rates were low. Whenever possible, immunization should be scheduled prior to initiating immunosuppressive therapy or during periods when temporary withdrawal of these medications is feasible. A shared decision-making is strongly recommended.	Moderate	95%
3) Whenever possible, vaccination should be completed before initiating therapy with conventional synthetic DMARDs, including methotrexate, leflunomide, hychloroquine, and sulfasalazine. However, inactivated vaccines are considered safe and can be administered during treatment with these agents, with low risk of adverse events or disease flare. Patient education and shared decision-making are recommended.	Very Low	100%
4) Ideally, vaccination should be offered before initiating immunosuppressive therapy with agents such as azathioprine, mycophenolate mofetil, cyclosporine A, or cyclophosphamide. When this is not feasible, inactivated vaccines may still be administered, with careful assessment of timing, immunogenicity, and individual risk-benefit balance through shared decision-making.	Very Low	100%
5) The use of bDMARDs (excluding rituximab) has a minimal impact on the immune response to inactivated vaccines in patients with IMRD. Vaccination with inactivated vaccines is recommended even during treatment with these agents, with decisions guided by individualized assessment and shared decision-making.	Very Low	100%
6) In patients with IMRD, rituximab significantly reduces the humoral response to inactivated vaccines. Whenever possible, immunization should be completed prior to initiating RTX therapy. For patients who have already received RTX and are not at imminent risk of infection, inactivated vaccines should preferably be administered at least six months after the last RTX dose.	Very Low	100%
7) The use of inactivated vaccines is recommended for patients IMRD receiving corticosteroids, even during treatment. Although corticosteroids may reduce vaccine immunogenicity, their use should not preclude vaccination. Patients should be informed about the possibility of a reduced immune response, and shared decision-making is advised.	Very Low	100%
8) Inactivated vaccines are recommended for patients with IMRD receiving Janus kinase inhibitors (JAKi). Vaccination should preferably be administered before initiating treatment, especially for vaccines for severe infections such as herpes zoster. If this is not feasible, vaccination may be performed during JAKi monotherapy, provided a shared decision-making and individualized assessment.	Very Low	89%
9) There is insufficient evidence to support the systematic interruption of immunosuppressive therapy such as methotrexate (MTX), mycophenolate mofetil (MMF), or Janus kinase inhibitors (JAKi)—exclusively to improve the immune response to vaccination in patients with IMRD. Shared decision-making should guide individualized approaches, taking into account disease activity and the risks associated with treatment interruption.	Very Low	88.9%
10) The use of the available influenza vaccine is recommended as a general rule. When both conventional and high-dose formulations are accessible, high-dose influenza vaccines may be considered for older adults and patients with a high degree of immunosuppression, following shared decision-making.	Very Low	100%
11) In patients with IMRD, the decision regarding the use of double-dose or four-dose hepatitis B vaccination regimens should be individualized and made through shared decision-making with the patient.	Very Low	95%

In all cases, at least 80% of the panelists expressed agreement (4 – agree or 5 – strongly agree), indicating a high level of consistency in the recommendations provided, and consensus was reached.

A summary of the 11 recommendations is presented in Table 1. A complete listing of the references and the main characteristics of the included studies can be found in the supplementary material.

Each recommendation is accompanied by the certainty of the evidence, rated using the Grading of Recommendations, Assessment, Development and Evaluation (**GRADE**) approach, and the **Level of Agreement (LOA)** among the panelists. The **certainty of evidence** was classified into four levels (High, Moderate, Low, and Very Low) based on the GRADE methodology, considering factors such as risk of bias, inconsistency, indirectness, imprecision, and publication bias. The **LOA** reflects the consensus level reached by the voting panel members through a Delphi process. It is presented as the sum of responses marked as “Strongly agree” and “Agree” on a 5-point Likert scale (1 = Strongly disagree, 2 = Disagree, 3 = Neutral, 4 = Agree, 5 = Strongly agree). A LOA of ≥80% was required for a recommendation to be approved.

**RECOMMENDATION 1.** The risk of flare or worsening of disease activity after inactivated vaccine administration in patients with IMRD is low. Vaccination should not be postponed due to concerns about disease exacerbation.

A systematic review was performed to evaluate the safety profile of inactivated vaccines in individuals with IMRD, focusing specifically on the frequency of symptom flares or increased disease activity that occurred following immunization. A ‘flare’ was defined as either a relapse of previously controlled/remitted disease or an exacerbation (worsening/aggravation) of a preexisting disease with lower activity [1–88].

**Fig. 1:**
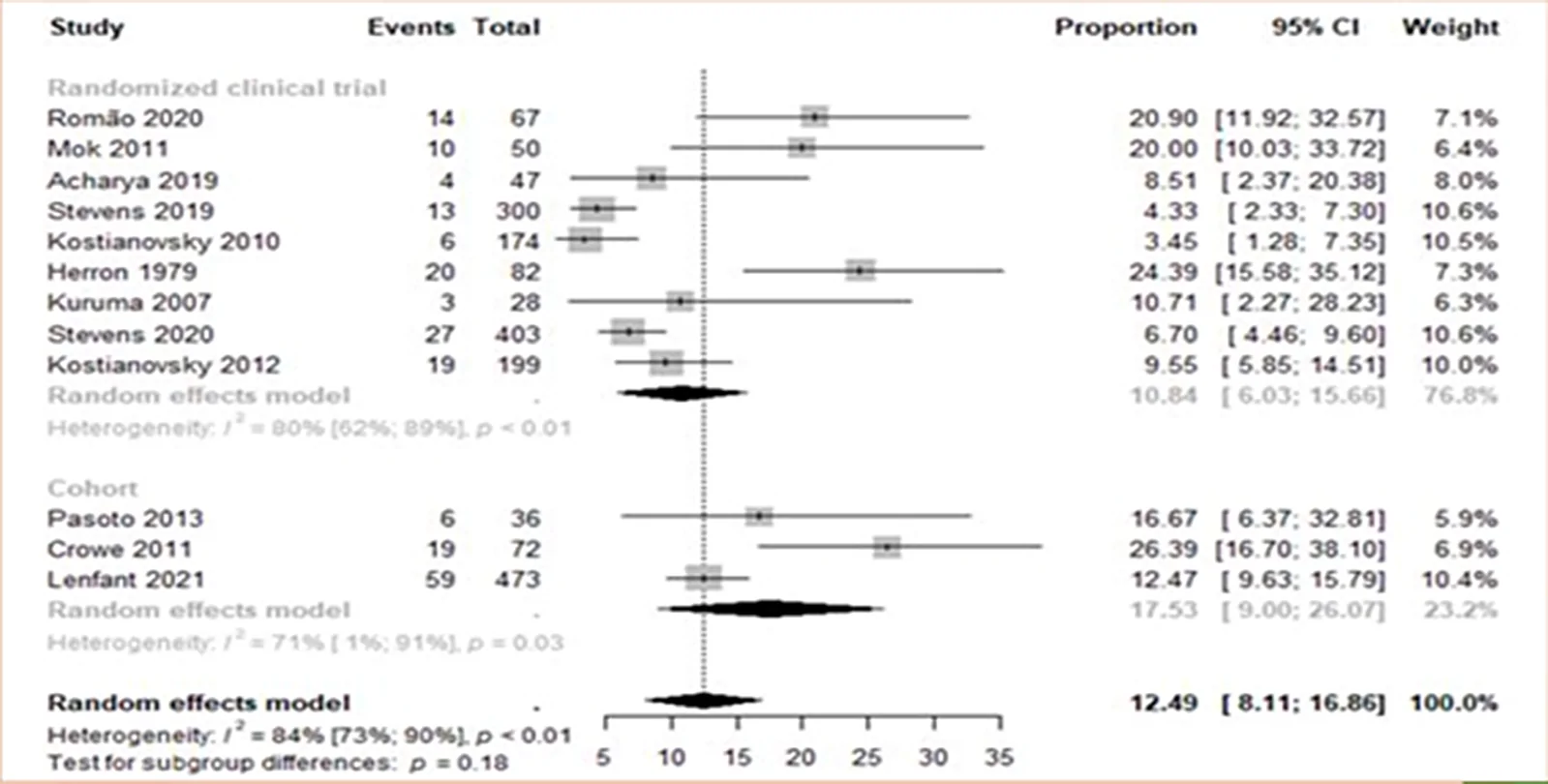
Pooled frequency of disease flare or worsening after inactivated vaccination in patients with immune-mediated rheumatic diseases, according to study design

A systematic review was conducted, identifying 91 eligible studies, including 1467 patients with IMRD. A total of 1467 individuals were analyzed. Twelve studies specifically evaluated flare symptoms following inactivated vaccination. The overall frequency of post-vaccine flares was 12.5%, with cohort studies reporting slightly higher flare rates (17.5%) than randomized controlled trials (10.8%) [22–33]. Importantly, most reported flares were mild and self-limited. These findings are supported by the meta-analysis shown in Fig. 1, which demonstrates a low pooled frequency of flares after inactivated vaccination across different study designs.

Disease activity was assessed using validated clinical indices. For patients with radiographic axial spondyloarthritis (r-axSpA) two studies evaluated changes in BASDAI, with a mild increase observed after vaccination: mean difference of 0.57 (95% CI 0.28–0.87) in randomized trials and 0.18 (95% CI −0.38 to 0.74) in observational cohorts [34, 35]. Among patients with rheumatoid arthritis, no significant change in DAS28 scores was found, and remission rates remained stable. Similarly, in systemic lupus erythematosus, vaccination was not associated with a significant increase in SLEDAI scores (SMD −0.10; 95% CI −0.81 to 0.60).

In addition to post-vaccine outcomes, disease activity at the time of vaccination was also described in eight studies. Among 552 patients with available data, 23.7% were in remission at the time of vaccination, 31.3% had low disease activity, 12.0% had moderate activity, and 8.0% were in a poorly controlled state [32]. These data reinforce the real-world applicability of the evidence, as they reflect a typical clinical spectrum of disease activity.

Although subgroup analyses did not reveal a higher flare risk associated with particular diagnoses or disease activity levels, the limited number of studies and heterogeneity in outcome reporting suggest that rare or severe flares cannot be definitively excluded. Nevertheless, the consistency observed across studies supports a favorable safety profile for inactivated vaccines in this population.

In summary, these findings show that inactivated vaccines do not significantly raise the risk of flares or disease worsening in this population. Vaccination should thus be encouraged routinely, including when disease activity is low or moderate, based on clinical judgment and patient-centered discussions.

**LOA:** 50.0% Strongly agree; 50.0% Agree.

**Sum of the percentage of “Strongly agree” and “Agree”**: 100**%**.

**Overall quality of evidence across all critical outcomes:** Very Low**.**

Forest plot showing the pooled frequency of disease flare or worsening after administration of inactivated vaccines in patients with immune-mediated rheumatic diseases (IMRD), stratified by study design (randomized clinical trials and cohort studies). A random-effects model was applied to estimate pooled proportions and corresponding 95% confidence intervals. Overall heterogeneity was assessed using the I^2^ statistic. The diamond represents the pooled estimate.

**RECOMMENDATION 2:** Live attenuated vaccines (LAVs) appear to be safe in patients with IMRD. Flare rates and vaccine-related infection rates were low. Whenever possible, immunization should be scheduled prior to initiating immunosuppressive therapy or during periods when temporary withdrawal of these medications is feasible. A shared decision-making process is strongly recommended.

A systematic review was conducted, identifying 18 eligible studies evaluating the safety of yellow fever, herpes zoster (live), measles, mumps, and rubella (MMR), and varicella vaccines, including a total of 47,133 patients with IMRD.

Flare rates after LAVs were low: **5.18%** with herpes zoster vaccine and **0%** with yellow fever vaccine. Infections caused by the vaccine virus were rare, with only **0.4%** for herpes zoster and **0%** for yellow fever [36, 37].These findings are consistent across multiple observational cohorts and interventional studies.

Disease activity remained stable following vaccination. No worsening in 28-joint Disease Activity Score (DAS28) was observed in rheumatoid arthritis patients. Similarly, no increases in Bath Ankylosing Spondylitis Disease Activity Index (BASDAI) scores were seen in r-axSpA. In systemic lupus erythematosus, no increase in Systemic Lupus Erythematosus Disease Activity Index (SLEDAI) was detected—even in patients vaccinated during active disease or using immunosuppressive therapies [38, 39].The safety of LAVs is further supported by post-vaccination surveillance data, where adverse events remained rare and predominantly mild. Most flares, when reported, were transient and did not lead to therapeutic changes [40].

All studies respected safety intervals for immunosuppressive drug withdrawal, including at least 3 months for conventional synthetic DMARDs, 5.5 half-lives for biologics, and 6 months after the last rituximab dose. These parameters align with Brazilian national recommendations for yellow fever vaccination in immunocompromised patients [6].

The risk of flare or worsening of disease activity following LAV in patients with IMRD is low. Immunization should be planned before starting immunosuppressants or during periods that allow temporary drug suspension, following a shared decision-making approach.

**LOA:** 60.0% Strongly agree; 35.0% Agree;

**Sum of “Strongly agree” and “Agree”:** 95%

**Overall quality of evidence across all critical outcomes:** Moderate

**RECOMMENDATION 3.** Whenever possible, vaccination should be completed before initiating therapy with conventional synthetic DMARDs, including methotrexate, leflunomide, hydroxychloroquine, and sulfasalazine. However, inactivated vaccines are considered safe and can be administered during treatment with these agents, with low risk of adverse events or disease flare. Patient education and shared decision-making are recommended.

A systematic review and meta-analysis was conducted, identifying 23 eligible studies evaluating the impact of csDMARDs on vaccine response, including a total of 2408 patients with IMRD. Among these, 15 studies assessed MTX, 5 evaluated HCQ, and 3 involved multiple csDMARDs (MTX, LEF, SSZ and HCQ). The analysis covered various inactivated vaccines: diphtheria-tetanus (dT, 2 studies), human papillomavirus (HPV, 1 study), H1N1 influenza (13 studies), influenza B (6 studies), H3N2 (5 studies), and pneumococcal vaccines (4 studies). The inverse variance method was employed to combine multiple study arms, ensuring that the number of healthy controls in the comparative analysis was not duplicated [41–61].

**Fig. 2:**
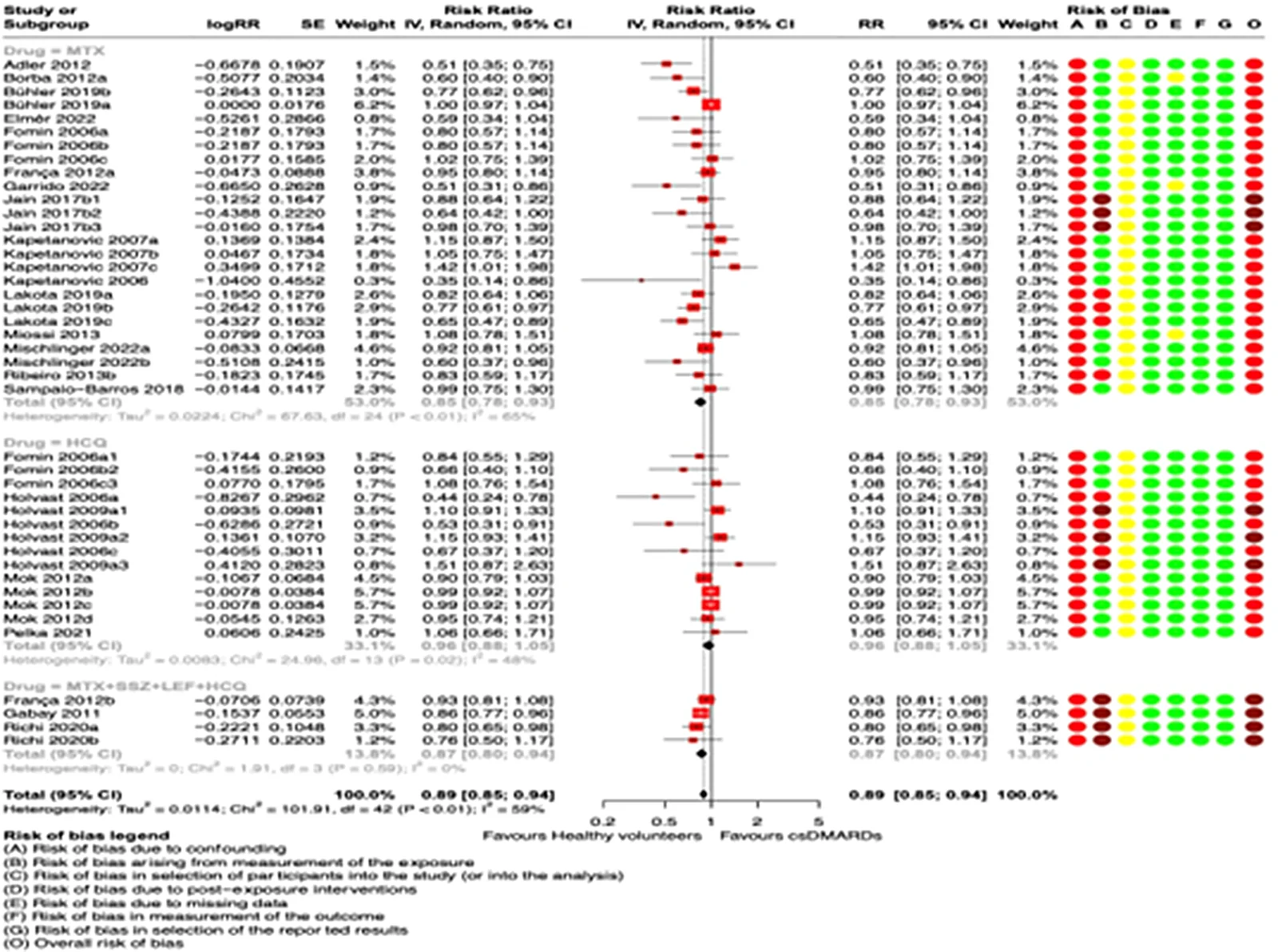
Effect of conventional synthetic DMARDs on vaccine immunogenicity in patients with immune-mediated rheumatic diseases

The pooled analysis showed an **11% reduction in vaccine response** (95% CI **5%-15%**) in patients receiving MTX compared to healthy individuals. These findings are supported by the meta-analysis shown in Fig. 2. Subgroup analyses considering disease type and specific vaccines **did not reveal significant differences**. These findings suggest that, while MTX may slightly attenuate vaccine immunogenicity, this reduction does not reach a clinically meaningful threshold, supporting continued vaccination in patients undergoing csDMARD therapy.

Vaccination is recommended for patients using csDMARDs, even if already under treatment, as the potential reduction in immunogenicity does not outweigh the benefits of immunization. Individualized strategies, such as temporary discontinuation of MTX before vaccination in selected cases, may be considered to optimize vaccine response.

**LOA:** 77.8% **Strongly agree**; 22.2% **Agree**.

**Sum of the percentage of “Strongly agree” and “Agree”**: 100**%**.

**Overall quality of evidence across all critical outcomes:** Very Low.

Forest plot showing the effect of conventional synthetic disease-modifying antirheumatic drugs (csDMARDs) on vaccine immunogenicity in patients with immune-mediated rheumatic diseases (IMRD). Risk ratios (RRs) compare vaccine responses in patients receiving csDMARDs versus healthy controls. Pooled estimates were calculated using a random-effects model with the inverse variance method. Subgroup analyses are presented according to the csDMARD used (methotrexate, hydroxychloroquine, and combined csDMARDs). Between-study heterogeneity was assessed using the I^2^ statistic. The diamond represents the pooled effect estimate.

**RECOMMENDATION 4.** Ideally, vaccination should be offered before initiating immunosuppressive therapy with agents such as azathioprine, mycophenolate mofetil, cyclosporine A, or cyclophosphamide. When this is not feasible, inactivated vaccines may still be administered, with careful assessment of timing, immunogenicity, and individual risk-benefit balance through shared decision-making.

A systematic review and meta-analysis was conducted, identifying 11 eligible studies evaluating the impact of AZA, MMF, or both on vaccine response, including a total of 1367 patients with IMRD. Of these, 5 studies assessed both drugs together, 6 focused specifically on AZA, and 3 on MMF. The vaccines assessed comprised diphtheria-tetanus (2 studies), HPV (1 study), influenza (6 studies: H1N1, B, H3N2), and pneumococcal vaccines (3 studies) [62–72].

The overall vaccine response was slightly reduced in immunosuppressed patients compared to healthy controls, with variability depending on the drug used:

- AZA: 7% reduction (range: 1%–16%)
- MMF: 37% reduction (range: 21%–50%)
- AZA + MMF + CYC: 10% reduction (range: 1%–22%)

Although subgroup analyses by underlying disease type revealed no statistically significant differences, stratification by vaccine type showed a 42% reduction (range: 20%–58%) in response to pneumococcal vaccines. However, this finding is limited by the small sample size across studies.

The safety profile of vaccination in this population was favorable. No serious adverse events (SAEs), such as Guillain-Barré syndrome or infections requiring hospitalization, were identified. The most reported adverse effects were mild and included fever, injection site pain, fatigue, and arthralgia, occurring at similar frequencies to those observed in the general population.

In conclusion, despite immunosuppressants (notably MMF) possibly dampening vaccine immunogenicity, inactivated vaccines are still advised during treatment.

**LOA:** 50% **Strongly agree**; 50% **Agree**.

**Sum of the percentage of “Strongly agree” and “Agree”**: 100**%**.

**Overall quality of evidence across all critical outcomes:** Very Low**.**

**RECOMMENDATION 5.** The use of biologic disease-modifying antirheumatic drugs (excluding rituximab) has a minimal impact on the immune response to inactivated vaccines in patients with IMRD. Vaccination is recommended even during treatment with these agents, with decisions guided by individualized assessment and shared decision-making.

The systematic review included 17 studies, with a total of 888 patients, evaluating inactivated vaccine responses in IMRD patients receiving bDMARDs. The analysis included TNF inhibitors (infliximab, etanercept, adalimumab, certolizumab pegol, golimumab) and non-TNF inhibitors (belimumab, anifrolumab, tocilizumab, secukinumab, ixekizumab, guselkumab, risankizumab, and vedolizumab).

A total of 17 studies were included in the meta-analysis: 12 involving TNF inhibitors, 3 with IL-17 inhibitors, and 2 with abatacept. Vaccine response was evaluated based on the type of vaccine: adult diphtheria-tetanus, hepatitis B, influenza (H1N1, influenza B, H3N2), recombinant herpes zoster, and pneumococcal vaccines [73–90].

**Fig. 3:**
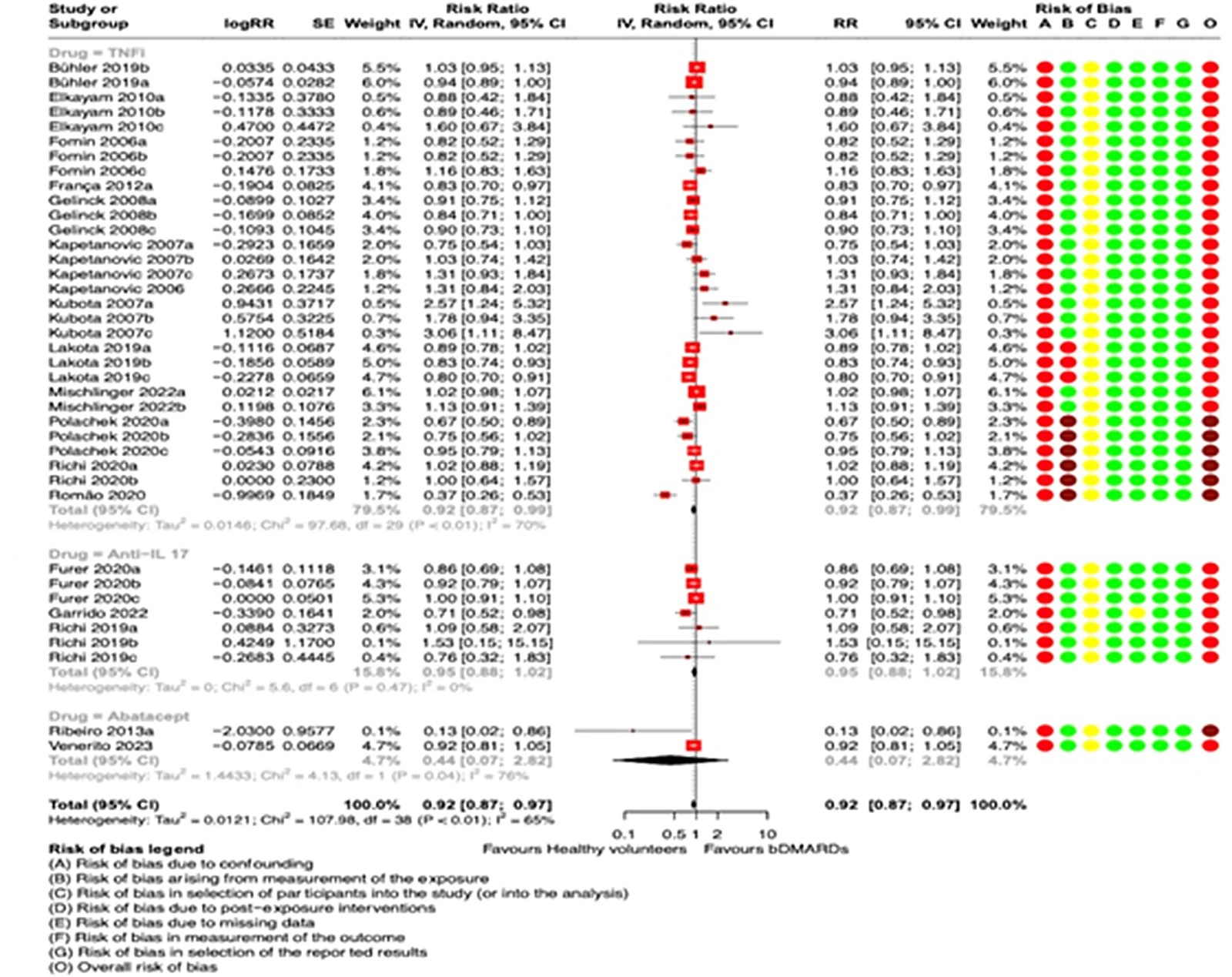
Effect of biologic disease-modifying antirheumatic drugs (excluding rituximab) on the immunogenic response to inactivated vaccines in patients with immune-mediated rheumatic diseases

Results showed a modest reduction in vaccine response compared to healthy individuals, with variability depending on the bDMARD class. These findings are supported by the meta-analysis shown in Fig. 3.

- TNF inhibitors: 8% reduction (range: 1%-13%)
- IL-17 inhibitors: 5% reduction (range: 0%-12%)
- Abatacept: inconclusive results due to high variability and a limited number of studies. One study reported a 60% reduction based on a sample of 11 patients, while another found no significant impact on vaccine response.

Although several subgroup analyses were performed based on disease type and vaccine type, no statistically significant differences were observed. Only one study evaluating the hepatitis B vaccine showed a statistically significant reduction in response, but this finding was limited by a small sample size.

The immunosuppressive effects of bDMARDs (excluding rituximab) do not significantly impair the response to inactivated vaccines, with reductions ranging from 5% to 8% (0%-12%). Therefore, inactivated vaccines are recommended even during treatment with these agents, with decisions guided by individualized assessment and shared decision-making.

**LOA:** 66.7% **Strongly agree**; 33.3% **Agree**.

**Sum of the percentage of “Strongly agree” and “Agree”**: 100**%**.

**Overall quality of evidence across all critical outcomes:** Very Low.

Forest plot showing the effect of biologic disease-modifying antirheumatic drugs (bDMARDs), excluding rituximab, on vaccine immunogenicity in patients with immune-mediated rheumatic diseases (IMRD). Risk ratios (RRs) compare vaccine responses in patients receiving bDMARDs versus healthy controls. Pooled estimates were calculated using a random-effects model with the inverse variance method. Subgroup analyses are presented according to bDMARD class (TNF inhibitors, IL-17 inhibitors, and abatacept). Between-study heterogeneity was assessed using the I^2^ statistic. The diamond represents the pooled effect estimate.

**RECOMMENDATION 6.** In patients with immune-mediated rheumatic diseases (IMRD), rituximab (RTX) significantly reduces the humoral response to inactivated vaccines. Whenever possible, immunization should be completed prior to initiating RTX therapy. For patients who have already received RTX and are not at imminent risk of infection, inactivated vaccines should preferably be administered at least six months after the last RTX dose.

A total of 41 studies were identified, with a total of 1750 patients, including 35 through database searches and 6 through manual search. These studies evaluated the immunogenicity of various inactivated vaccines in IMRD patients treated with RTX. Most of the evidence is related to influenza vaccine studies, which consistently showed reduced seroconversion and seroprotection rates, particularly when the vaccine was administered within the first 4 to 12 weeks after RTX infusion. In contrast, antibody titers were significantly higher when vaccination occurred beyond 24 weeks after RTX administration [91–107].

Data regarding other vaccines such as pneumococcal, hepatitis B, and dT vaccines confirm a similar trend of reduced humoral response. Seroconversion to hepatitis B vaccine was as low as 25%–29% among RTX users, and the proportion of patients achieving protective titers for pneumococcal and dT vaccines was also lower. T-cell mediated responses, however, appeared to be less affected and were preserved in several studies.

Although the evidence is heterogeneous in terms of population, vaccine type, and methodology, the overall findings consistently support a significant attenuation of antibody response to inactivated vaccines following RTX exposure. Timing of vaccination relative to RTX infusion plays a critical role in optimizing vaccine efficacy.

**LOA:** 88.9% Strongly agree; 11.1% Agree.

**Sum of the percentage of “Strongly agree” and “Agree”**: 100**%**.

**Overall quality of evidence across all critical outcomes:** Very Low.

**RECOMMENDATION 7.** The use of inactivated vaccines is recommended for patients with IMRD receiving corticosteroids, even during treatment. Although corticosteroids may reduce vaccine immunogenicity, their use should not preclude vaccination. Patients should be informed about the possibility of a reduced immune response, and shared decision-making is advised.

A systematic review was conducted, identifying 21 eligible studies, including 3896 patients with IMRD and 2373 healthy controls. A total of 9269 individuals were analyzed. Vaccines assessed included influenza (16 studies), pneumococcal (3 studies), HPV (1 study), and dT (1 study) [108–118].

**Fig. 4:**
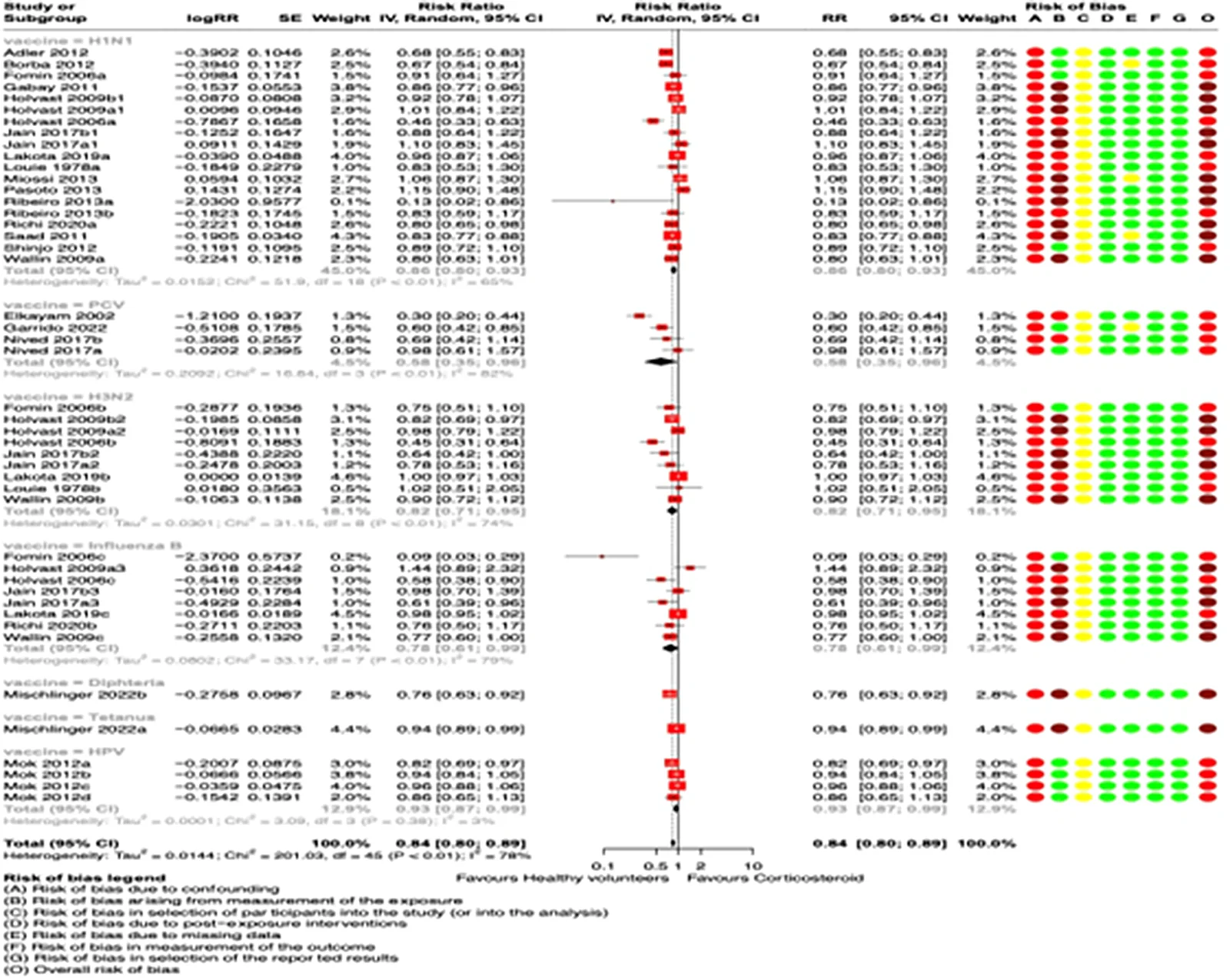
Effect of corticosteroid use on the immunogenic response to inactivated vaccines in patients with immune-mediated rheumatic diseases

Overall, corticosteroid use was associated with an 18% reduction in vaccine response compared to healthy controls (risk ratio 0.82; 95% CI 0.77–0.87). These findings are supported by the meta-analysis shown in Fig. 4. Subgroup analyses based on disease type or specific vaccine platform revealed no statistically significant differences, except for one small study on pneumococcal vaccines that showed a marked reduction in response. However, the limited sample size precluded definitive conclusions.

Due to the lack of consistent data on corticosteroid dosage across studies, a dose–response analysis could not be performed. Although the reduction in immunogenicity was statistically significant, its clinical relevance regarding vaccine efficacy remains uncertain.

**LOA:** 72.2% Strongly agree; 27.8% Agree.

**Sum of the percentage of “Strongly agree” and “Agree”**: 100%.

**Overall quality of evidence across all critical outcomes:** Very Low.

Forest plot showing the effect of corticosteroid use on vaccine immunogenicity after administration of inactivated vaccines in patients with immune-mediated rheumatic diseases (IMRD). Risk ratios (RRs) compare vaccine responses in patients receiving corticosteroids versus healthy controls. Pooled estimates were calculated using a random-effects model with the inverse variance method. Subgroup analyses are presented according to vaccine type. Between-study heterogeneity was assessed using the I^2^ statistic. The diamond represents the pooled effect estimate.

**RECOMMENDATION 8.** Inactivated vaccines are recommended for patients with IMRD receiving Janus kinase inhibitors (JAKi). Vaccination should preferably be administered before initiating treatment, especially for vaccines preventing severe infections such as herpes zoster. If that is not feasible, vaccination may be performed during JAKi monotherapy, provided that shared decision-making and individualized assessment are ensured.

This recommendation was based on a narrative review that summarized available evidence on vaccine immunogenicity in patients receiving JAK inhibitors. Although the data are limited and heterogeneous, the available study suggest that inactivated vaccines are generally safe and may induce a modest immune response in this population. Several studies have evaluated the immunogenicity of vaccines in patients using JAKi. One study assessed the response to the 13-valent pneumococcal conjugate vaccine (PCV13) and tetanus toxoid in patients with rheumatoid arthritis treated with baricitinib, a selective JAK1/2 inhibitor. The results showed that 68% of patients achieved an adequate response to the pneumococcal vaccine, and 43% to the tetanus toxoid. Notably, concomitant corticosteroid use did not reduce the response to the pneumococcal vaccine, although the tetanus response was suboptimal [119].

Another study investigated the impact of tofacitinib on the response to pneumococcal and influenza vaccines. The pneumococcal vaccine response was significantly reduced, particularly in patients on combination therapy with methotrexate. Temporary interruption of tofacitinib slightly improved immunogenicity, although the difference was not statistically significant. The influenza vaccine response remained largely preserved.

The recombinant herpes zoster vaccine (RZV) demonstrated good immunogenicity and tolerability in patients treated with JAK inhibitors. Post-vaccination IgG levels were similar to those observed in healthy individuals, and no major safety concerns were identified [120, 121].

An additional study comparing PCV13 response in patients on JAKi monotherapy, JAKi combined with methotrexate, and methotrexate monotherapy showed that monotherapy groups had better immunogenicity than the combination group [122].

A systematic review also supported that JAK inhibitors may impair vaccine immunogenicity, especially in combination with other immunosuppressive agents [123]. Furthermore, one observational study suggested a reduction in respiratory infections among JAKi users who had received pneumococcal vaccination [124].

Taken together, these findings indicate that JAK inhibitors may reduce immune response to certain vaccines, particularly those requiring a primary immune response. However, responses may still be satisfactory, especially when used as monotherapy and when corticosteroids are not co-administered.

**LOA:** 83.3% Strongly agree; 5.6% Agree.

**Sum of the percentage of “Strongly agree” and “Agree”**: 89**%**.

**Overall quality of evidence across all critical outcomes:** Very Low.

**RECOMMENDATION 9.** There is insufficient evidence to support the systematic interruption of immunosuppressive therapy—such as methotrexate (MTX), mycophenolate mofetil (MMF), or Janus kinase inhibitors (JAKi)—exclusively to improve the immune response to vaccination in patients with IMRD. Shared decision-making should guide individualized approaches, taking into account disease activity and the risks associated with treatment interruption.

This systematic review identified two randomized clinical trials that evaluated the temporary suspension of MTX or JAKi: both conducted in South Korea. No eligible studies were found addressing MMF discontinuation in this context. While both trials reported increased seropositivity among patients who temporarily suspended treatment, this advantage was not sustained in the pooled analysis. The meta-analysis revealed no statistically significant difference in vaccine response between those who interrupted therapy and those who continued: RR 1.16 (95% CI 0.81–1.66) [125, 126].

Furthermore, existing EULAR recommendations highlight the risk of disease flare or worsening activity associated with temporary withdrawal of immunomodulatory therapies for vaccination purposes. Given the very limited number of studies, the modest and nonsignificant effect size, and the potential harm from disease reactivation, the routine suspension of MTX, MMF, or JAKi for vaccination purposes is not recommended.

Vaccination should ideally occur during periods of disease remission or low disease activity and under the lowest possible level of immunosuppression. Any decision to interrupt therapy must be carefully balanced with the risk of disease flare and guided by individualized, shared decision-making with the patient.

**LOA:** 61.1% Strongly agree; 27.8% Agree.

**Sum of the percentage of “Strongly agree” and “Agree”**: 88.9**%**.

**Overall quality of evidence across all critical outcomes:** Very Low.

**RECOMMENDATION 10.** The use of the available influenza vaccine is recommended as a general rule. When both conventional and high-dose formulations are accessible, high-dose influenza vaccines may be considered for older adults and patients with a high degree of immunosuppression, following shared decision-making.

Although no study has directly compared the efficacy or effectiveness of conventional versus high-dose influenza vaccines specifically in patients with IMRD, indirect evidence suggests a potential benefit of high-dose formulations in individuals with higher degrees of immunosuppression [127–129].

In pooled data, patients vaccinated with conventional-dose formulations had a 13% lower seropositivity rate and a 22% lower seroprotection rate compared to those receiving high-dose vaccines. These findings indicate a modest immunogenic advantage for the high-dose formulations, although the evidence remains indirect and based on a limited number of studies, mostly in populations that include, but are not restricted to, IMRD patients [127–129].

Until further studies specifically focused on IMRD populations are available, the preferred strategy is to use the influenza vaccine that is most readily accessible. When high-dose vaccines are available, their use may be prioritized in older adults or individuals under intense immunosuppression, especially those with suboptimal vaccine responses, after appropriate clinical discussion and shared decision-making.

**LOA:** 72.2% Strongly agree; 27.8% Agree.

**Sum of the percentage of “Strongly agree” and “Agree”**: 100%.

**Overall quality of evidence across all critical outcomes:** Very Low.

**RECOMMENDATION 11.** In patients with IMRD, the decision regarding the use of double-dose or four-dose hepatitis B vaccination regimens should be individualized and made through shared decision-making with the patient.

The immune response to hepatitis B virus (HBV) vaccination may be suboptimal in patients with IMRD, particularly those receiving immunosuppressive therapies. This recommendation was informed by a narrative review of the literature, as no systematic reviews or meta-analyses specific to IMRD populations were identified. The available evidence includes studies conducted in IMRD and extrapolated data from patients with inflammatory bowel disease (IBD), due to similarities in immunosuppressive treatment and immune response patterns. These studies suggest that alternative strategies, such as increased antigen doses or additional vaccine doses, may enhance immunogenicity in this population [130–132].

Studies in patients with inflammatory bowel disease (IBD), which share immunological characteristics with IMRD, indicate that dose escalation or an additional booster dose may improve seroconversion rates. A meta-analysis demonstrated that immune response rates were comparable between standard-dose and double-dose regimens, as well as among different vaccination schedules. However, immunosuppressive therapy was consistently identified as a predictor of reduced serological response [130].

International guidelines, such as those from the **Canadian Association of Gastroenterology**, underscore the inconsistency in available data regarding the effectiveness of double-dose regimens in patients with autoimmune diseases. While some analyses suggest an improved serological response, others report no statistically significant difference compared to the standard regimen [131].

A study by Solay et al. assessed the immunogenicity of hepatitis B vaccination in individuals undergoing immunobiological therapy. Among the 109 patients evaluated, the seroconversion rate was 49.3% in the standard-dose group (three doses of 20 μg/ml) and 61.1% in the high-dose group (40 μg/ml), with no statistically significant difference between groups (*p* = 0.246) [132].

Currently, there is no robust evidence confirming the superiority of double-dose or four-dose regimens over the conventional hepatitis B vaccination schedule in patients with IMRD. Moreover, safety should be considered, particularly given potential reports of increased adverse events associated with double-dose schedules. Therefore, vaccination decisions must be highly individualized, carefully weighing patient-specific factors, predictors of reduced immune response, immunization benefits, and these safety aspects through shared decision-making with the patient.

**LOA:** 66.7% **Strongly agree**; 27.8% **Agree**; 5.6% **Neutral**.

**Sum of the percentage of “Strongly agree” and “Agree”**: 95**%**.

**Overall quality of evidence across all critical outcomes:** Very Low.

#### Conclusions

This consensus document, developed by an expert task force of the Brazilian Society of Rheumatology, provides evidence-based recommendations regarding the use of vaccines in adult patients with IMRD, considering both, safety and response. These recommendations were grounded in systematic reviews and meta-analyses of the available literature and reflect the Brazilian epidemiological setting and clinical context. The inclusion of quantitative syntheses and forest plots from the meta-analyses strengthens the robustness of these recommendations by providing clearer estimates of vaccine safety and immunogenicity across different immunosuppressive therapies.

It is important to recognize the methodological limitations and heterogeneity of the included studies, which resulted in low to moderate certainty of evidence in most outcomes. Therefore, the task force highlights the urgent need for further high-quality studies in the field of immunization in patients with IMRD to guide the development and improvement of future guidelines.

These recommendations aim to support healthcare professionals in the decision-making process for the vaccine-preventable infections in patients with IMRD. Nevertheless, the interpretation and application of these recommendations should consider the patient’s disease activity level, comorbidities, immunosuppressive regimen, vaccine access, and patient preferences. Shared decision-making is essential in all clinical scenarios [133].

## Electronic supplementary material

Below is the link to the electronic supplementary material.

Supplementary Material 1

Supplementary Material 2

Supplementary Material 3

## Data Availability

No datasets were generated or analysed during the current study.
