# Supplementary material for: Brazilian Society of Rheumatology – 2025 recommendations on vaccination in immune-mediated rheumatic diseases
Source: Adv Rheumatol. 2026 Jan 23;66:14. doi: 10.1186/s42358-026-00520-8 (PMC13488773; doi:10.1186/s42358-026-00520-8)
Supplement: Supplementary file 1 [file 42358_2026_520_MOESM1_ESM.docx]

**RECOMMENDATIONS OF THE BRAZILIAN SOCIETY OF RHEUMATOLOGY FOR VACCINATION IN PATIENTS WITH IMMUNE MEDIATED RHEUMATIC DISEASES**

1. **CENTRAL QUESTIONS AND ACRONYMS**

**Question 1.** What is the risk of symptom flare up or worsening of patients with immune-mediated rheumatic diseases after vaccination with the inactivated vaccine?

| **Population (P)** | Patients with immune-mediated rheumatic diseases |
| --- | --- |
| **Intervention (I)** | Vaccination with inactivated vaccine |
| **Comparator group (C)** | Not applicable |
| **Outcomes (O)** | Symptom flare up or worsening |

**Question 2.** What is the risk of symptom flare up or worsening of patients with immune-mediated rheumatic diseases after vaccination with live attenuated vaccine?

| **Population (P)** | Patients with immune-mediated rheumatic diseases |
| --- | --- |
| **Intervention (I)** | Vaccination with live attenuated vaccine |
| **Comparator group (C)** | Not applicable |
| **Outcomes (O)** | Symptom flare up or worsening |

**Question 3.** What is the impact of the cs-DMARDs (MTX, LEF, SZL, HXQ) use on the response to inactivated vaccine in patients with immune-mediated rheumatic diseases?

| **Population (P)** | Patients with immune-mediated rheumatic diseases |
| --- | --- |
| **Intervention (I)** | cs-DMARDs (MTX, LEF, SZL, HXQ) use |
| **Comparator group (C)** | Not applicable |
| **Outcomes (O)** | vaccine response to virus-inactivated vaccines: Influenza, pneumococcal conjugate, recombinant zoster, HBV, HPV |

**Question 4.** What is the impact of the immunosuppressants use (azathioprine, MMF, cyclosporine, cyclophosphamide) on the response to inactivated vaccine in patients with immune-mediated rheumatic diseases?

| **Population (P)** | Patients with immune-mediated rheumatic diseases |
| --- | --- |
| **Intervention (I)** | immunosuppressants use (azathioprine, MMF, cyclosporine, cyclophosphamide) |
| **Comparator group (C)** | Not applicable |
| **Outcomes (O)** | vaccine response to virus-inactivated vaccines: Influenza, pneumococcal conjugate, recombinant zoster, HBV, HPV |

**Question 5.** What effect of DMARDsb use (except RTX) have on the on the response to inactivated vaccine in patients with immune-mediated rheumatic diseases?

| **Population (P)** | Patients with immune-mediated rheumatic diseases |
| --- | --- |
| **Intervention (I)** | DMARDsb use (except RTX) |
| **Comparator group (C)** | Not applicable |
| **Outcomes (O)** | vaccine response to virus-inactivated vaccines: Influenza, pneumococcal conjugate, recombinant zoster, HBV, HPV |

**Question 6.** What is the impact of the RTX use on the response to inactivated vaccine in patients with immune-mediated rheumatic diseases?

| **Population (P)** | Patients with immune-mediated rheumatic diseases |
| --- | --- |
| **Intervention (I)** | RTX use |
| **Comparator group (C)** | Not applicable |
| **Outcomes (O)** | vaccine response to virus-inactivated vaccines: Influenza, pneumococcal conjugate, recombinant zoster, HBV, HPV |

**Question 7.** What is the impact of corticosteroids use on the response to inactivated vaccine in patients with immune-mediated rheumatic diseases?

| **Population (P)** | Patients with immune-mediated rheumatic diseases |
| --- | --- |
| **Intervention (I)** | corticosteroids use |
| **Comparator group (C)** | Not applicable |
| **Outcomes (O)** | vaccine response to virus-inactivated vaccines: Influenza, pneumococcal conjugate, recombinant zoster, HBV, HPV |

**Question 8.** What is the impact of JAKis use on the response to inactivated vaccine in patients with immune-mediated rheumatic diseases?

| **Population (P)** | Patients with immune-mediated rheumatic diseases |
| --- | --- |
| **Intervention (I)** | JAKi use |
| **Comparator group (C)** | Not applicable |
| **Outcomes (O)** | vaccine response to virus-inactivated vaccines: Influenza, pneumococcal conjugate, recombinant zoster, HBV, HPV |

**Question 9.** Is there a benefit to vaccine response by temporarily stopping methotrexate, mycophenolate, iJAKs before or after vaccination?

| **Population (P)** | Patients with immune-mediated rheumatic diseases |
| --- | --- |
| **Intervention (I)** | Drug Tapering (methotrexate, mycophenolate, iJAKs) |
| **Comparator group (C)** | Not applicable |
| **Outcomes (O)** | vaccine response to virus-inactivated vaccines: Influenza, pneumococcal conjugate, recombinant zoster, HBV, HPV |

**Question 10.**  Is there a difference in the immune response between conventional (tri- and tetravalent) and high-dose influenza vaccines in patients with immune-mediated rheumatic diseases “IMRD)?

| **Population (P)** | Patients with immune-mediated rheumatic diseases |
| --- | --- |
| **Intervention (I)** | conventional (tri- and tetravalent)  influenza vaccines |
| **Comparator group (C)** | high-dose influenza vaccines |
| **Outcomes (O)** | Immune response |

# Question 11.  Is there a difference in the immune response between conventional “tri- and tetravalent) and high-dose Hepatitis B Vaccines in patients with immune-mediated rheumatic diseases “IMRD)?

| **Population (P)** | Patients with immune-mediated rheumatic diseases |
| --- | --- |
| **Intervention (I)** | conventional (tri- and tetravalent)  hepatitis B vaccines |
| **Comparator group (C)** | high-dose influenza vaccines |
| **Outcomes (O)** | Immune response |

2. **SEARCHS KEY**

**Question 1.** What is the risk of symptom flare up or worsening of patients with immune-mediated rheumatic diseases after vaccination with the inactivated vaccine?

| Group | **MeSH Terms**  and definitions | **Entry Terms** | **Search Key** |
| --- | --- | --- | --- |
| Population  Patients with immune-mediated rheumatic diseases (IMRD) | [Rheumatic Diseases - MeSH - NCBI (nih.gov)](https://www.ncbi.nlm.nih.gov/mesh/68012216)  Disorders of connective tissue, especially the joints and related structures, characterized by inflammation, degeneration, or metabolic derangement. | - Rheumatic Disease - Rheumatism | "[Rheumatic Diseases](https://www.ncbi.nlm.nih.gov/mesh/68012216)"[Mesh:NoExp] OR "Rheumatic Disease" OR "Rheumatism" OR  "Immune-mediated rheumatic diseases"  (não utilizar [Mesh] pois a árvore do termo inclui gota, fibromialgia e crianças) |
|  | [Arthritis, Rheumatoid - MeSH - NCBI (nih.gov)](https://www.ncbi.nlm.nih.gov/mesh/68001172)  A chronic systemic disease, primarily of the joints, marked by inflammatory changes in the synovial membranes and articular structures, widespread fibrinoid degeneration of the collagen fibers in mesenchymal tissues, and by atrophy and rarefaction of bony structures. Etiology is unknown, but autoimmune mechanisms have been implicated. | - Rheumatoid Arthritis | "[Arthritis, Rheumatoid](https://www.ncbi.nlm.nih.gov/mesh/68001172)"[Mesh:NoExp] OR "Rheumatoid Arthritis" OR "[Rheumatoid Nodule](https://www.ncbi.nlm.nih.gov/mesh/68012218)" OR "[Rheumatoid Vasculitis](https://www.ncbi.nlm.nih.gov/mesh/68056653)" OR "[Sjogren's Syndrome](https://www.ncbi.nlm.nih.gov/mesh/68012859)"  (não utilizar [Mesh] pois a árvore do termo inclui caplan syndrome, felty syndrome) |
|  | [Spondylarthritis - MeSH - NCBI (nih.gov)](https://www.ncbi.nlm.nih.gov/mesh/68025241)  Inflammation of the joints of the SPINE, the intervertebral articulations. | - Spondylarthritides - Spinal Arthritis - Spinal Arthritides | "[Spondylarthritis](https://www.ncbi.nlm.nih.gov/mesh/68025241)" [Mesh] OR "[Spondylarthritis](https://www.ncbi.nlm.nih.gov/mesh/68025241)" OR "Spondylarthritides" OR "Spinal Arthritis" OR "Spinal Arthritides"  [Spondylitis](https://www.ncbi.nlm.nih.gov/mesh/68013166)  Spondylarthritis  [Osteoarthritis, Spine](https://www.ncbi.nlm.nih.gov/mesh/68055013)  [Spondylarthropathies](https://www.ncbi.nlm.nih.gov/mesh/68025242)  [Arthritis, Psoriatic](https://www.ncbi.nlm.nih.gov/mesh/68015535)  [Arthritis, Reactive](https://www.ncbi.nlm.nih.gov/mesh/68016918)  [Axial Spondyloarthritis](https://www.ncbi.nlm.nih.gov/mesh/2101123) + |
|  | [Arthritis, Psoriatic - MeSH - NCBI (nih.gov)](https://www.ncbi.nlm.nih.gov/mesh/68015535)  A type of inflammatory arthritis associated with PSORIASIS, often involving the axial joints and the peripheral terminal interphalangeal joints. It is characterized by the presence of HLA-B27-associated SPONDYLARTHROPATHY, and the absence of rheumatoid factor. | - Arthritic Psoriasis - Psoriatic Arthritis - Psoriasis Arthropathica - Psoriatic Arthropathy - Psoriatic Arthropathies | "[Arthritis, Psoriatic](https://www.ncbi.nlm.nih.gov/mesh/68015535)" [Mesh] OR "[Arthritis, Psoriatic](https://www.ncbi.nlm.nih.gov/mesh/68015535)" OR "Arthritic Psoriasis" OR "Psoriatic Arthritis" OR "Psoriasis Arthropathica" OR "Psoriatic Arthropathy" OR "Psoriatic Arthropathies"  [Spondylarthritis](https://www.ncbi.nlm.nih.gov/mesh/68025241)  [Spondylarthropathies](https://www.ncbi.nlm.nih.gov/mesh/68025242)  Arthritis, Psoriatic |
|  | [Spondylitis, Ankylosing - MeSH - NCBI (nih.gov)](https://www.ncbi.nlm.nih.gov/mesh/?term=ankylosing+spondylitis)  A chronic inflammatory condition affecting the axial joints, such as the SACROILIAC JOINT and other intervertebral or costovertebral joints. It occurs predominantly in young males and is characterized by pain and stiffness of joints (ANKYLOSIS) with inflammation at tendon insertions. | - Bechterew's Disease - Bechterews Disease - Marie Struempell Disease - Spondylarthritis Ankylopoietica - Spondyloarthritis Ankylopoietica - Ankylosing Spondylitis - Ankylosing Spondylarthritis - Ankylosing Spondylarthritides - Ankylosing Spondyloarthritis - Ankylosing Spondyloarthritides - Spondylitis Ankylopoietica - Bechterew Disease - Rheumatoid Spondylitis | "[Spondylitis, Ankylosing](https://www.ncbi.nlm.nih.gov/mesh/?term=ankylosing+spondylitis)" [Mesh] OR "[Spondylitis, Ankylosing](https://www.ncbi.nlm.nih.gov/mesh/?term=ankylosing+spondylitis)" OR "Bechterew's Disease" OR "Bechterews Disease" OR "Marie Struempell Disease" OR "Spondylarthritis Ankylopoietica" OR "Spondyloarthritis Ankylopoietica" OR "Ankylosing Spondylitis" OR "Ankylosing Spondylarthritis" OR "Ankylosing Spondylarthritides" OR "Ankylosing Spondyloarthritis" OR "Ankylosing Spondyloarthritides" OR "Spondylitis Ankylopoietica" OR "Bechterew Disease" OR "Rheumatoid Spondylitis"  [Spondylarthritis](https://www.ncbi.nlm.nih.gov/mesh/68025241)  [Spondylarthropathies](https://www.ncbi.nlm.nih.gov/mesh/68025242)  [Axial Spondyloarthritis](https://www.ncbi.nlm.nih.gov/mesh/2101123)  Spondylitis, Ankylosing |
|  | [Lupus Erythematosus, Systemic - MeSH - NCBI (nih.gov)](https://www.ncbi.nlm.nih.gov/mesh/68008180)  A chronic, relapsing, inflammatory, and often febrile multisystemic disorder of connective tissue, characterized principally by involvement of the skin, joints, kidneys, and serosal membranes. It is of unknown etiology, but is thought to represent a failure of the regulatory mechanisms of the autoimmune system. The disease is marked by a wide range of system dysfunctions, an elevated erythrocyte sedimentation rate, and the formation of LE cells in the blood or bone marrow. | - Systemic Lupus Erythematosus - Lupus Erythematosus Disseminatus - Libman Sacks Disease | "[Lupus Erythematosus, Systemic](https://www.ncbi.nlm.nih.gov/mesh/68008180)" [Mesh] OR "[Lupus Erythematosus, Systemic](https://www.ncbi.nlm.nih.gov/mesh/68008180)" OR "Systemic Lupus Erythematosus " OR "Lupus Erythematosus Disseminatus" OR "Libman Sacks Disease"  [Connective Tissue Diseases](https://www.ncbi.nlm.nih.gov/mesh/68003240)  Lupus Erythematosus, Systemic  [Lupus Nephritis](https://www.ncbi.nlm.nih.gov/mesh/68008181)  [Lupus Vasculitis, Central Nervous System](https://www.ncbi.nlm.nih.gov/mesh/68020945) |
|  | [Myositis - MeSH - NCBI (nih.gov)](https://www.ncbi.nlm.nih.gov/mesh/?term=inflammatory+myopathies)  Inflammation of a muscle or muscle tissue. | - Myositides - Inflammatory Muscle Diseases - Inflammatory Muscle Disease - Inflammatory Myopathy - Inflammatory Myopathies - Proliferative Myositides - Proliferative Myositis - Infectious Myositides - Infectious Myositis - Idiopathic Inflammatory Myopathies - Idiopathic Inflammatory Myopathy - Idiopathic Inflammatory Myositis - Focal Myositides - Focal Myositis | "[Myositis](https://www.ncbi.nlm.nih.gov/mesh/?term=inflammatory+myopathies)" [Mesh] OR "[Myositis](https://www.ncbi.nlm.nih.gov/mesh/?term=inflammatory+myopathies)" OR "Myositides" OR "Inflammatory Muscle Diseases" OR "Inflammatory Muscle Disease" OR "Inflammatory Myopathy" OR "Inflammatory Myopathies" OR "Proliferative Myositides" OR "Proliferative Myositis" OR "Infectious Myositides" OR "Infectious Myositis" OR "Idiopathic Inflammatory Myopathies" OR "Idiopathic Inflammatory Myopathy" OR "Idiopathic Inflammatory Myositis" OR "Focal Myositides" OR "Focal Myositis"  [Muscular Diseases](https://www.ncbi.nlm.nih.gov/mesh/68009135)  Myositis  [Myositis Ossificans](https://www.ncbi.nlm.nih.gov/mesh/68009221)  [Myositis, Inclusion Body](https://www.ncbi.nlm.nih.gov/mesh/68018979)  [Orbital Myositis](https://www.ncbi.nlm.nih.gov/mesh/68055622)  [Polymyositis](https://www.ncbi.nlm.nih.gov/mesh/68017285)  [Dermatomyositis](https://www.ncbi.nlm.nih.gov/mesh/68003882)  [Pyomyositis](https://www.ncbi.nlm.nih.gov/mesh/68052880) |
|  | [Systemic Vasculitis - MeSH - NCBI (nih.gov)](https://www.ncbi.nlm.nih.gov/mesh/?term=systemic+vasculitides)  A heterogeneous group of diseases characterized by inflammation and necrosis of the blood vessel walls. | - Systemic Vasculitides | "[Systemic Vasculitis](https://www.ncbi.nlm.nih.gov/mesh/?term=systemic+vasculitides)" [Mesh] OR "[Systemic Vasculitis](https://www.ncbi.nlm.nih.gov/mesh/?term=systemic+vasculitides)" OR "Systemic Vasculitides"  [Vasculitis](https://www.ncbi.nlm.nih.gov/mesh/68014657)  Systemic Vasculitis  [Anti-Neutrophil Cytoplasmic Antibody-Associated Vasculitis](https://www.ncbi.nlm.nih.gov/mesh/68056648)  [Churg-Strauss Syndrome](https://www.ncbi.nlm.nih.gov/mesh/68015267)  [Granulomatosis with Polyangiitis](https://www.ncbi.nlm.nih.gov/mesh/68014890)  [Microscopic Polyangiitis](https://www.ncbi.nlm.nih.gov/mesh/68055953)  [Polyarteritis Nodosa](https://www.ncbi.nlm.nih.gov/mesh/68010488)  [Rheumatoid Vasculitis](https://www.ncbi.nlm.nih.gov/mesh/68056653) |
|  | [Sjogren's Syndrome - MeSH - NCBI (nih.gov)](https://www.ncbi.nlm.nih.gov/mesh/68012859)  Chronic inflammatory and autoimmune disease in which the salivary and lacrimal glands undergo progressive destruction by lymphocytes and plasma cells resulting in decreased production of saliva and tears. The primary form, often called sicca syndrome, involves both KERATOCONJUNCTIVITIS SICCA and XEROSTOMIA. The secondary form includes, in addition, the presence of a connective tissue disease, usually rheumatoid arthritis. | - Sjogrens Syndrome - Sjogren Syndrome - Sicca Syndrome | "[Sjogren's Syndrome](https://www.ncbi.nlm.nih.gov/mesh/68012859)" [Mesh] OR "[Sjogren's Syndrome](https://www.ncbi.nlm.nih.gov/mesh/68012859)" OR "Sjogrens Syndrome" OR "Sjogren Syndrome" OR "Sicca Syndrome"  [Arthritis](https://www.ncbi.nlm.nih.gov/mesh/68001168)  [Arthritis, Rheumatoid](https://www.ncbi.nlm.nih.gov/mesh/68001172)  Sjogren's Syndrome |
|  | [Scleroderma, Systemic - MeSH - NCBI (nih.gov)](https://www.ncbi.nlm.nih.gov/mesh/68012595)  A chronic multi-system disorder of CONNECTIVE TISSUE. It is characterized by SCLEROSIS in the SKIN, the LUNGS, the HEART, the GASTROINTESTINAL TRACT, the KIDNEYS, and the MUSCULOSKELETAL SYSTEM. Other important features include diseased small BLOOD VESSELS and AUTOANTIBODIES. The disorder is named for its most prominent feature (hard skin), and classified into subsets by the extent of skin thickening: LIMITED SCLERODERMA and DIFFUSE SCLERODERMA. | - Systemic Sclerosis - Systemic Scleroderma | "[Scleroderma, Systemic](https://www.ncbi.nlm.nih.gov/mesh/68012595)" [Mesh] OR "[Scleroderma, Systemic](https://www.ncbi.nlm.nih.gov/mesh/68012595)" OR "Systemic Sclerosis" OR "Systemic Scleroderma"  [Connective Tissue Diseases](https://www.ncbi.nlm.nih.gov/mesh/68003240)  Scleroderma, Systemic  [Scleroderma, Diffuse](https://www.ncbi.nlm.nih.gov/mesh/68045743)  [Scleroderma, Limited](https://www.ncbi.nlm.nih.gov/mesh/68045745)  [CREST Syndrome](https://www.ncbi.nlm.nih.gov/mesh/68017675) |
|  | [Mixed Connective Tissue Disease - MeSH - NCBI “nih.gov)](https://www.ncbi.nlm.nih.gov/mesh/?term=Mixed+connective+tissue+disease)  A syndrome with overlapping clinical features of systemic lupus erythematosus, scleroderma, polymyositis, and Raynaud's phenomenon. The disease is differentially characterized by high serum titers of antibodies to ribonuclease-sensitive extractable “saline soluble) nuclear antigen and a "speckled" epidermal nuclear staining pattern on direct immunofluorescence. | - Sharp Syndrome - MCTD | "[Mixed Connective Tissue Disease](https://www.ncbi.nlm.nih.gov/mesh/?term=Mixed+connective+tissue+disease)" [Mesh] OR "[Mixed Connective Tissue Disease](https://www.ncbi.nlm.nih.gov/mesh/?term=Mixed+connective+tissue+disease)" OR "Sharp Syndrome" OR "MCTD"  [Connective Tissue Diseases](https://www.ncbi.nlm.nih.gov/mesh/68003240)  Mixed Connective Tissue Disease |
| Intervention  Vaccination with inactivated vaccine | [Vaccines, Inactivated - MeSH - NCBI “nih.gov)](https://www.ncbi.nlm.nih.gov/mesh/68015164)  Definition: Vaccines in which the infectious microbial nucleic acid components have been destroyed by chemical or physical treatment “e.g., formalin, beta-propiolactone, gamma radiation) without affecting the antigenicity or immunogenicity of the viral coat or bacterial outer membrane proteins. | Inactivated Vaccines  Killed Vaccine  Killed Vaccines  Inactivated Vaccine | "[Vaccines, Inactivated](https://www.ncbi.nlm.nih.gov/mesh/68015164)" [Mesh] OR "[Vaccines, Inactivated](https://www.ncbi.nlm.nih.gov/mesh/68015164)" OR "Inactivated Vaccines" OR "Killed Vaccine" OR "Killed Vaccines" OR "Inactivated Vaccine"  Vaccines, Inactivated  [Poliovirus Vaccine, Inactivated](https://www.ncbi.nlm.nih.gov/mesh/68011054) |
|  | [Influenza Vaccines - MeSH - NCBI “nih.gov)](https://www.ncbi.nlm.nih.gov/mesh/68007252)  Vaccines used to prevent infection by viruses in the family ORTHOMYXOVIRIDAE. It includes both killed and attenuated vaccines. The composition of the vaccines is changed each year in response to antigenic shifts and changes in prevalence of influenza virus strains. The flu vaccines may be mono- or multi-valent, which contains one or more INFLUENZAVIRUS A and INFLUENZAVIRUS B strains. | - Influenza Vaccine - Influenzavirus Vaccines - Influenza Virus Vaccines - Influenzavirus Vaccine - Influenza Virus Vaccine - High-Dose Trivalent Influenza Vaccine - High Dose Trivalent Influenza Vaccine - Trivalent Influenza Vaccine - Quadrivalent Influenza Vaccine - High dose | "[Influenza Vaccines](https://www.ncbi.nlm.nih.gov/mesh/68007252)"[Mesh:NoExp]  OR Influenza Vaccine" OR "Influenzavirus Vaccines" OR "Influenza Virus Vaccines" OR "Influenzavirus Vaccine" OR "Influenza Virus Vaccine" OR "High-Dose Trivalent Influenza Vaccine" OR "High Dose Trivalent Influenza Vaccine" OR "Trivalent Influenza Vaccine" OR "Quadrivalent Influenza Vaccine"  “não utilizar [Mesh] pois a árvore do termo inclui VVA e intra-nasal)  [Vaccines](https://www.ncbi.nlm.nih.gov/mesh/68014612)  [Viral Vaccines](https://www.ncbi.nlm.nih.gov/mesh/68014765)  Influenza Vaccines |
|  | [Pneumococcal Vaccines - MeSH - NCBI “nih.gov)](https://www.ncbi.nlm.nih.gov/mesh/68022242)  Vaccines or candidate vaccines used to prevent infections with STREPTOCOCCUS PNEUMONIAE. | - Pneumococcal Vaccine - Pneumovax - Pneumococcal Polysaccharide Vaccine - Pnu Imune Vaccine - PnuImune Vaccine - Conjugated penumococcal vaccine | "[Pneumococcal Vaccines](https://www.ncbi.nlm.nih.gov/mesh/68022242)" [Mesh] OR "[Pneumococcal Vaccines](https://www.ncbi.nlm.nih.gov/mesh/68022242)" OR "Pneumococcal Vaccine" OR "Pneumovax" OR "Pneumococcal Polysaccharide Vaccine" OR "Pnu Imune Vaccine" OR "PnuImune Vaccine" OR "Conjugated penumococcal vaccine"  Pneumococcal Vaccines  [Heptavalent Pneumococcal Conjugate Vaccine](https://www.ncbi.nlm.nih.gov/mesh/2009980)  13 pneumococcal conjugate vaccine |
|  | [Herpes Zoster Vaccine - MeSH - NCBI “nih.gov)](https://www.ncbi.nlm.nih.gov/mesh/68053061)  An attenuated vaccine used to prevent and/or treat HERPES ZOSTER, a disease caused by HUMAN HERPESVIRUS 3. | - Zoster Vaccine - Shingles Vaccine | "Herpes Zoster Vaccine"[Mesh:NoExp] OR " Zoster Vaccine" OR "Shingles Vaccine"  “não utilizar [Mesh] pois a árvore do termo inclui live attenuated zoster  e intra-nasal)  [Vaccines](https://www.ncbi.nlm.nih.gov/mesh/68014612)  [Viral Vaccines](https://www.ncbi.nlm.nih.gov/mesh/68014765)  [Herpesvirus Vaccines](https://www.ncbi.nlm.nih.gov/mesh/68022283)  Herpes Zoster Vaccine |
|  | [Hepatitis B Vaccines - MeSH - NCBI “nih.gov)](https://www.ncbi.nlm.nih.gov/mesh/68017325)  Vaccines or candidate vaccines containing inactivated hepatitis B or some of its component antigens and designed to prevent hepatitis B. Some vaccines may be recombinantly produced. | Hepatitis B Vaccine | "Hepatitis B Vaccines" [Mesh] OR "Hepatitis B Vaccines" OR "Hepatitis B Vaccine"  [Vaccines](https://www.ncbi.nlm.nih.gov/mesh/68014612)  [Viral Vaccines](https://www.ncbi.nlm.nih.gov/mesh/68014765)  [Viral Hepatitis Vaccines](https://www.ncbi.nlm.nih.gov/mesh/68014761)  Hepatitis B Vaccines |
|  | [Papillomavirus Vaccines - MeSH - NCBI “nih.gov)](https://www.ncbi.nlm.nih.gov/mesh/?term=papillomavirus+vaccines)   Vaccines or candidate vaccines used to prevent PAPILLOMAVIRUS INFECTIONS. Human vaccines are intended to reduce the incidence of UTERINE CERVICAL NEOPLASMS, so they are sometimes considered a type of CANCER VACCINES. They are often composed of CAPSID PROTEINS, especially L1 protein, from various types of ALPHAPAPILLOMAVIRUS | - Papillomavirus Vaccine   Human Papillomavirus Vaccines   - HPV Vaccine   Human Papilloma Virus Vaccine   - HPV Vaccines - Human Papilloma Virus Vaccines - Human Papillomavirus Vaccine | "Papillomavirus Vaccines" [Mesh] OR "Papillomavirus Vaccines" OR "Papillomavirus Vaccine" OR "Human Papillomavirus Vaccines" OR "HPV Vaccine" OR "Human Papilloma Virus Vaccine" OR "HPV Vaccines" OR "Human Papilloma Virus Vaccines" OR "Human Papillomavirus Vaccine"  [Vaccines](https://www.ncbi.nlm.nih.gov/mesh/68014612)  [Viral Vaccines](https://www.ncbi.nlm.nih.gov/mesh/68014765)  Papillomavirus Vaccines  [Human Papillomavirus](https://www.ncbi.nlm.nih.gov/mesh/2009889) |
|  | [Poliovirus Vaccines - MeSH - NCBI “nih.gov)](https://www.ncbi.nlm.nih.gov/mesh/68023321)   Vaccines used to prevent POLIOMYELITIS. They include inactivated “POLIOVIRUS VACCINE, INACTIVATED) | - Poliovirus Vaccine - salk | "Poliovirus Vaccines" [Mesh] OR "Poliovirus Vaccines" OR "Poliovirus Vaccine" OR "salk"  [Vaccines](https://www.ncbi.nlm.nih.gov/mesh/68014612)  [Viral Vaccines](https://www.ncbi.nlm.nih.gov/mesh/68014765)  [Poliovirus Vaccine, Inactivated](https://www.ncbi.nlm.nih.gov/mesh/68011054) |
|  | [Hepatitis A Vaccines - MeSH - NCBI “nih.gov)](https://www.ncbi.nlm.nih.gov/mesh/68022362)   Vaccines or candidate vaccines used to prevent infection with hepatitis A virus “HEPATOVIRUS). | - Hepatovirus Vaccines - Hepatitis A Vaccine - Vaqta - Havrix | "Hepatitis A Vaccines" [Mesh] OR "Hepatitis A Vaccines" OR "Hepatovirus Vaccines" OR "Hepatitis A Vaccine" OR "Vaqta" OR "Havrix"  [Vaccines](https://www.ncbi.nlm.nih.gov/mesh/68014612)  [Viral Hepatitis Vaccines](https://www.ncbi.nlm.nih.gov/mesh/68014761)  Hepatitis A Vaccines |
|  | [Meningococcal Vaccines - MeSH - NCBI “nih.gov)](https://www.ncbi.nlm.nih.gov/mesh/68022401)   Vaccines or candidate vaccines used to prevent infection with NEISSERIA MENINGITIDIS. | Meningococcal Vaccine  Menactra  Tetravalent Meningococcal Vaccine  Menomune  Menveo  Bivalent Meningococcal Vaccine  Meningococcal Polysaccharide Vaccine | "Meningococcal Vaccines" [Mesh] OR " Meningococcal Vaccine" OR "Menactra" OR "Tetravalent Meningococcal Vaccine" OR "Menomune OR "Menveo" OR "Bivalent Meningococcal Vaccine" OR "Meningococcal Polysaccharide Vaccine"    [Vaccines](https://www.ncbi.nlm.nih.gov/mesh/68014612)  Meningococcal Vaccines C, B or ACWY |
|  | [Diphtheria-Tetanus-Pertussis Vaccine - MeSH - NCBI “nih.gov)](https://www.ncbi.nlm.nih.gov/mesh/?term=vaccine+per)   A vaccine consisting of DIPHTHERIA TOXOID; TETANUS TOXOID; and whole-cell PERTUSSIS VACCINE. The vaccine protects against diphtheria, tetanus, and whooping cough. | Diphtheria Tetanus Pertussis Vaccine  DTwP Vaccine  DPT Vaccine  DTP Vaccine  Di Te Per Vaccine  Diphtheria Pertussis Tetanus Vaccine | "Diphtheria-Tetanus-Pertussis Vaccine" [Mesh] OR "Diphtheria-Tetanus-Pertussis Vaccine" OR " Diphtheria Tetanus Pertussis Vaccine" OR "DTwP Vaccine" OR "DPT Vaccine" OR "DTP Vaccine" OR "Di Te Per Vaccine" OR "Diphtheria Pertussis Tetanus Vaccine"  [Vaccines](https://www.ncbi.nlm.nih.gov/mesh/68014612)  [Pertussis Vaccine](https://www.ncbi.nlm.nih.gov/mesh/68010567)  Diphtheria-Tetanus-Pertussis Vaccine |
|  | [Haemophilus influenza type b polysaccharide vaccine-tetanus toxin conj - MeSH - NCBI “nih.gov)](https://www.ncbi.nlm.nih.gov/mesh/?term=haemophilus+influenza+type+b+polysaccharide+vaccine+tetanus+toxin+conjugate)   the type b capsular polysaccharide of HIB-vaccine is conjugated to tetanus toxoid | HIBest  PRP-T  Hib-TT  Hib capsular polysaccharide-tetanus vaccine  Hib CPS-tetanus vaccine  OmniHIB  ActHIB | "Haemophilus influenza type b polysaccharide vaccine-tetanus toxin conjugate" OR "HIBest" OR "PRP-T" OR "Hib-TT" OR "Hib capsular polysaccharide-tetanus vaccine" OR "Hib CPS-tetanus vaccine" OR "OmniHIB" OR "ActHIB" |

**Question 2.** What is the risk of infection and  symptom flare up or worsening of IMRD disease activity after vaccination with live attenuated vaccine?

| Group | **MeSH Terms**  and definitions | **Entry Terms** | **Search Key** |
| --- | --- | --- | --- |
| Population  Patients with immune-mediated rheumatic diseases “IMRD) | [Rheumatic Diseases - MeSH - NCBI “nih.gov)](https://www.ncbi.nlm.nih.gov/mesh/68012216)  Disorders of connective tissue, especially the joints and related structures, characterized by inflammation, degeneration, or metabolic derangement. | Rheumatic Disease  Rheumatism | ""[Rheumatic Diseases](https://www.ncbi.nlm.nih.gov/mesh/68012216)"[Mesh:NoExp] OR "Rheumatic Disease" OR "Rheumatism" OR  "Immune-mediated rheumatic diseases"    “não utilizar [Mesh] pois a árvore do termo inclui gota, fibromialgia e crianças) |
|  | [Arthritis, Rheumatoid - MeSH - NCBI “nih.gov)](https://www.ncbi.nlm.nih.gov/mesh/68001172)  A chronic systemic disease, primarily of the joints, marked by inflammatory changes in the synovial membranes and articular structures, widespread fibrinoid degeneration of the collagen fibers in mesenchymal tissues, and by atrophy and rarefaction of bony structures. Etiology is unknown, but autoimmune mechanisms have been implicated. | - Rheumatoid Arthritis | "[Arthritis, Rheumatoid](https://www.ncbi.nlm.nih.gov/mesh/68001172)"[Mesh:NoExp] OR "Rheumatoid Arthritis" OR "[Rheumatoid Nodule](https://www.ncbi.nlm.nih.gov/mesh/68012218)" OR "[Rheumatoid Vasculitis](https://www.ncbi.nlm.nih.gov/mesh/68056653)" OR "[Sjogren's Syndrome](https://www.ncbi.nlm.nih.gov/mesh/68012859)"  “não utilizar [Mesh] pois a árvore do termo inclui caplan syndrome, felty syndrome) |
|  | [Spondylarthritis - MeSH - NCBI “nih.gov)](https://www.ncbi.nlm.nih.gov/mesh/68025241)  Inflammation of the joints of the SPINE, the intervertebral articulations. | - Spondylarthritides - Spinal Arthritis - Spinal Arthritides | "[Spondylarthritis](https://www.ncbi.nlm.nih.gov/mesh/68025241)" [Mesh] OR "[Spondylarthritis](https://www.ncbi.nlm.nih.gov/mesh/68025241)" OR "Spondylarthritides" OR "Spinal Arthritis" OR "Spinal Arthritides"  [Spondylitis](https://www.ncbi.nlm.nih.gov/mesh/68013166)  Spondylarthritis  [Osteoarthritis, Spine](https://www.ncbi.nlm.nih.gov/mesh/68055013)  [Spondylarthropathies](https://www.ncbi.nlm.nih.gov/mesh/68025242)  [Arthritis, Psoriatic](https://www.ncbi.nlm.nih.gov/mesh/68015535)  [Arthritis, Reactive](https://www.ncbi.nlm.nih.gov/mesh/68016918)  [Axial Spondyloarthritis](https://www.ncbi.nlm.nih.gov/mesh/2101123) + |
|  | [Arthritis, Psoriatic - MeSH - NCBI “nih.gov)](https://www.ncbi.nlm.nih.gov/mesh/68015535)  A type of inflammatory arthritis associated with PSORIASIS, often involving the axial joints and the peripheral terminal interphalangeal joints. It is characterized by the presence of HLA-B27-associated SPONDYLARTHROPATHY, and the absence of rheumatoid factor. | - Arthritic Psoriasis - Psoriatic Arthritis - Psoriasis Arthropathica - Psoriatic Arthropathy - Psoriatic Arthropathies | "[Arthritis, Psoriatic](https://www.ncbi.nlm.nih.gov/mesh/68015535)" [Mesh] OR "[Arthritis, Psoriatic](https://www.ncbi.nlm.nih.gov/mesh/68015535)" OR "Arthritic Psoriasis" OR "Psoriatic Arthritis" OR "Psoriasis Arthropathica" OR "Psoriatic Arthropathy" OR "Psoriatic Arthropathies"  [Spondylarthritis](https://www.ncbi.nlm.nih.gov/mesh/68025241)  [Spondylarthropathies](https://www.ncbi.nlm.nih.gov/mesh/68025242)  Arthritis, Psoriatic |
|  | [Spondylitis, Ankylosing - MeSH - NCBI “nih.gov)](https://www.ncbi.nlm.nih.gov/mesh/?term=ankylosing+spondylitis)  A chronic inflammatory condition affecting the axial joints, such as the SACROILIAC JOINT and other intervertebral or costovertebral joints. It occurs predominantly in young males and is characterized by pain and stiffness of joints “ANKYLOSIS) with inflammation at tendon insertions. | - Bechterew's Disease - Bechterews Disease - Marie Struempell Disease - Spondylarthritis Ankylopoietica - Spondyloarthritis Ankylopoietica - Ankylosing Spondylitis - Ankylosing Spondylarthritis - Ankylosing Spondylarthritides - Ankylosing Spondyloarthritis - Ankylosing Spondyloarthritides - Spondylitis Ankylopoietica - Bechterew Disease - Rheumatoid Spondylitis | "[Spondylitis, Ankylosing](https://www.ncbi.nlm.nih.gov/mesh/?term=ankylosing+spondylitis)" [Mesh] OR "[Spondylitis, Ankylosing](https://www.ncbi.nlm.nih.gov/mesh/?term=ankylosing+spondylitis)" OR "Bechterew's Disease" OR "Bechterews Disease" OR "Marie Struempell Disease" OR "Spondylarthritis Ankylopoietica" OR "Spondyloarthritis Ankylopoietica" OR "Ankylosing Spondylitis" OR "Ankylosing Spondylarthritis" OR "Ankylosing Spondylarthritides" OR "Ankylosing Spondyloarthritis" OR "Ankylosing Spondyloarthritides" OR "Spondylitis Ankylopoietica" OR "Bechterew Disease" OR "Rheumatoid Spondylitis"  [Spondylarthritis](https://www.ncbi.nlm.nih.gov/mesh/68025241)  [Spondylarthropathies](https://www.ncbi.nlm.nih.gov/mesh/68025242)  [Axial Spondyloarthritis](https://www.ncbi.nlm.nih.gov/mesh/2101123)  Spondylitis, Ankylosing |
|  | [Lupus Erythematosus, Systemic - MeSH - NCBI “nih.gov)](https://www.ncbi.nlm.nih.gov/mesh/68008180)  A chronic, relapsing, inflammatory, and often febrile multisystemic disorder of connective tissue, characterized principally by involvement of the skin, joints, kidneys, and serosal membranes. It is of unknown etiology, but is thought to represent a failure of the regulatory mechanisms of the autoimmune system. The disease is marked by a wide range of system dysfunctions, an elevated erythrocyte sedimentation rate, and the formation of LE cells in the blood or bone marrow. | - Systemic Lupus Erythematosus - Lupus Erythematosus Disseminatus - Libman Sacks Disease | "[Lupus Erythematosus, Systemic](https://www.ncbi.nlm.nih.gov/mesh/68008180)" [Mesh] OR "[Lupus Erythematosus, Systemic](https://www.ncbi.nlm.nih.gov/mesh/68008180)" OR "Systemic Lupus Erythematosus " OR "Lupus Erythematosus Disseminatus" OR "Libman Sacks Disease"  [Connective Tissue Diseases](https://www.ncbi.nlm.nih.gov/mesh/68003240)  Lupus Erythematosus, Systemic  [Lupus Nephritis](https://www.ncbi.nlm.nih.gov/mesh/68008181)  [Lupus Vasculitis, Central Nervous System](https://www.ncbi.nlm.nih.gov/mesh/68020945) |
|  | [Myositis - MeSH - NCBI “nih.gov)](https://www.ncbi.nlm.nih.gov/mesh/?term=inflammatory+myopathies)  Inflammation of a muscle or muscle tissue. | - Myositides - Inflammatory Muscle Diseases - Inflammatory Muscle Disease - Inflammatory Myopathy - Inflammatory Myopathies - Proliferative Myositides - Proliferative Myositis - Infectious Myositides - Infectious Myositis - Idiopathic Inflammatory Myopathies - Idiopathic Inflammatory Myopathy - Idiopathic Inflammatory Myositis - Focal Myositides - Focal Myositis | "[Myositis](https://www.ncbi.nlm.nih.gov/mesh/?term=inflammatory+myopathies)" [Mesh] OR "[Myositis](https://www.ncbi.nlm.nih.gov/mesh/?term=inflammatory+myopathies)" OR "Myositides" OR "Inflammatory Muscle Diseases" OR "Inflammatory Muscle Disease" OR "Inflammatory Myopathy" OR "Inflammatory Myopathies" OR "Proliferative Myositides" OR "Proliferative Myositis" OR "Infectious Myositides" OR "Infectious Myositis" OR "Idiopathic Inflammatory Myopathies" OR "Idiopathic Inflammatory Myopathy" OR "Idiopathic Inflammatory Myositis" OR "Focal Myositides" OR "Focal Myositis"  [Muscular Diseases](https://www.ncbi.nlm.nih.gov/mesh/68009135)  Myositis  [Myositis Ossificans](https://www.ncbi.nlm.nih.gov/mesh/68009221)  [Myositis, Inclusion Body](https://www.ncbi.nlm.nih.gov/mesh/68018979)  [Orbital Myositis](https://www.ncbi.nlm.nih.gov/mesh/68055622)  [Polymyositis](https://www.ncbi.nlm.nih.gov/mesh/68017285)  [Dermatomyositis](https://www.ncbi.nlm.nih.gov/mesh/68003882)  [Pyomyositis](https://www.ncbi.nlm.nih.gov/mesh/68052880) |
|  | [Systemic Vasculitis - MeSH - NCBI “nih.gov)](https://www.ncbi.nlm.nih.gov/mesh/?term=systemic+vasculitides)  A heterogeneous group of diseases characterized by inflammation and necrosis of the blood vessel walls. | - Systemic Vasculitides | "[Systemic Vasculitis](https://www.ncbi.nlm.nih.gov/mesh/?term=systemic+vasculitides)" [Mesh] OR "[Systemic Vasculitis](https://www.ncbi.nlm.nih.gov/mesh/?term=systemic+vasculitides)" OR "Systemic Vasculitides"  [Vasculitis](https://www.ncbi.nlm.nih.gov/mesh/68014657)  Systemic Vasculitis  [Anti-Neutrophil Cytoplasmic Antibody-Associated Vasculitis](https://www.ncbi.nlm.nih.gov/mesh/68056648)  [Churg-Strauss Syndrome](https://www.ncbi.nlm.nih.gov/mesh/68015267)  [Granulomatosis with Polyangiitis](https://www.ncbi.nlm.nih.gov/mesh/68014890)  [Microscopic Polyangiitis](https://www.ncbi.nlm.nih.gov/mesh/68055953)  [Polyarteritis Nodosa](https://www.ncbi.nlm.nih.gov/mesh/68010488)  [Rheumatoid Vasculitis](https://www.ncbi.nlm.nih.gov/mesh/68056653) |
|  | [Sjogren's Syndrome - MeSH - NCBI “nih.gov)](https://www.ncbi.nlm.nih.gov/mesh/68012859)  Chronic inflammatory and autoimmune disease in which the salivary and lacrimal glands undergo progressive destruction by lymphocytes and plasma cells resulting in decreased production of saliva and tears. The primary form, often called sicca syndrome, involves both KERATOCONJUNCTIVITIS SICCA and XEROSTOMIA. The secondary form includes, in addition, the presence of a connective tissue disease, usually rheumatoid arthritis. | - Sjogrens Syndrome - Sjogren Syndrome - Sicca Syndrome | "[Sjogren's Syndrome](https://www.ncbi.nlm.nih.gov/mesh/68012859)" [Mesh] OR "[Sjogren's Syndrome](https://www.ncbi.nlm.nih.gov/mesh/68012859)"  OR "Sjogrens Syndrome" OR "Sjogren Syndrome" OR "Sicca Syndrome"  [Arthritis](https://www.ncbi.nlm.nih.gov/mesh/68001168)  [Arthritis, Rheumatoid](https://www.ncbi.nlm.nih.gov/mesh/68001172)  Sjogren's Syndrome |
|  | [Scleroderma, Systemic - MeSH - NCBI “nih.gov)](https://www.ncbi.nlm.nih.gov/mesh/68012595)  A chronic multi-system disorder of CONNECTIVE TISSUE. It is characterized by SCLEROSIS in the SKIN, the LUNGS, the HEART, the GASTROINTESTINAL TRACT, the KIDNEYS, and the MUSCULOSKELETAL SYSTEM. Other important features include diseased small BLOOD VESSELS and AUTOANTIBODIES. The disorder is named for its most prominent feature “hard skin), and classified into subsets by the extent of skin thickening: LIMITED SCLERODERMA and DIFFUSE SCLERODERMA. | - Systemic Sclerosis - Systemic Scleroderma | "[Scleroderma, Systemic](https://www.ncbi.nlm.nih.gov/mesh/68012595)" [Mesh] OR "[Scleroderma, Systemic](https://www.ncbi.nlm.nih.gov/mesh/68012595)" OR "Systemic Sclerosis" OR "Systemic Scleroderma"  [Connective Tissue Diseases](https://www.ncbi.nlm.nih.gov/mesh/68003240)  Scleroderma, Systemic  [Scleroderma, Diffuse](https://www.ncbi.nlm.nih.gov/mesh/68045743)  [Scleroderma, Limited](https://www.ncbi.nlm.nih.gov/mesh/68045745)  [CREST Syndrome](https://www.ncbi.nlm.nih.gov/mesh/68017675) |
|  | [Mixed Connective Tissue Disease - MeSH - NCBI “nih.gov)](https://www.ncbi.nlm.nih.gov/mesh/?term=Mixed+connective+tissue+disease)  A syndrome with overlapping clinical features of systemic lupus erythematosus, scleroderma, polymyositis, and Raynaud's phenomenon. The disease is differentially characterized by high serum titers of antibodies to ribonuclease-sensitive extractable “saline soluble) nuclear antigen and a "speckled" epidermal nuclear staining pattern on direct immunofluorescence. | - Sharp Syndrome - MCTD | "[Mixed Connective Tissue Disease](https://www.ncbi.nlm.nih.gov/mesh/?term=Mixed+connective+tissue+disease)" [Mesh] OR "[Mixed Connective Tissue Disease](https://www.ncbi.nlm.nih.gov/mesh/?term=Mixed+connective+tissue+disease)" OR "Sharp Syndrome" OR "MCTD"  [Connective Tissue Diseases](https://www.ncbi.nlm.nih.gov/mesh/68003240)  Mixed Connective Tissue Disease |
| Intervention  Vaccination with live unattenuated | [Vaccines, Live, Unattenuated](https://www.ncbi.nlm.nih.gov/mesh/68062690)  Live vaccines prepared from microorganisms without their virulence altered. | - Live Unattenuated Vaccines - Unattenuated Vaccines | "[Vaccines, Live, Unattenuated](https://www.ncbi.nlm.nih.gov/mesh/68062690)" [Mesh] OR "[Vaccines, Live, Unattenuated](https://www.ncbi.nlm.nih.gov/mesh/68062690)" OR "Live Unattenuated Vaccines" OR "Unattenuated Vaccines"  [Vaccines](https://www.ncbi.nlm.nih.gov/mesh/68014612)  Vaccines, Live, Unattenuated |
|  | [Yellow Fever Vaccine - MeSH - NCBI “nih.gov)](https://www.ncbi.nlm.nih.gov/mesh/68022341)  Vaccine used to prevent YELLOW FEVER. It consists of a live attenuated 17D strain of the YELLOW FEVER VIRUS. |  | "Yellow Fever Vaccine" [Mesh] OR " Yellow Fever Vaccine"  [Vaccines](https://www.ncbi.nlm.nih.gov/mesh/68014612)  [Viral Vaccines](https://www.ncbi.nlm.nih.gov/mesh/68014765)  Yellow Fever Vaccine |
|  | [Dengue Vaccines - MeSH - NCBI “nih.gov)](https://www.ncbi.nlm.nih.gov/mesh/?term=dengue+vaccine)  Vaccines or candidate vaccines used to prevent infection with DENGUE VIRUS. These include live-attenuated, subunit, DNA, and inactivated vaccines. | - Dengue Vaccine | "Dengue Vaccines" [Mesh] OR "Dengue Vaccines" OR "Dengue Vaccine"  [Vaccines](https://www.ncbi.nlm.nih.gov/mesh/68014612)  [Viral Vaccines](https://www.ncbi.nlm.nih.gov/mesh/68014765)  Dengue Vaccines |
|  | [Measles-Mumps-Rubella Vaccine - MeSH - NCBI “nih.gov)](https://www.ncbi.nlm.nih.gov/mesh/?term=MMR+vaccine)  A combined vaccine used to prevent MEASLES; MUMPS; and RUBELLA. | - Measles Mumps Rubella Vaccine - MMR Vaccine - Mumps Measles Rubella Vaccine - Triviraten Berna - Priorix - Trimovax - Pluserix - Virivac | "Measles-Mumps-Rubella Vaccine" [Mesh] OR "Measles-Mumps-Rubella Vaccine" OR "Measles Mumps Rubella Vaccine" OR "MMR Vaccine" OR "Mumps Measles Rubella Vaccine" OR "Triviraten Berna" OR "Priorix" OR "Trimovax" OR "Pluserix" OR "Virivac"  [Vaccines](https://www.ncbi.nlm.nih.gov/mesh/68014612)  [Vaccines, Combined](https://www.ncbi.nlm.nih.gov/mesh/68017778)  Measles-Mumps-Rubella Vaccine |
|  | [Chickenpox Vaccine - MeSH - NCBI “nih.gov)](https://www.ncbi.nlm.nih.gov/mesh/?term=chickenpox+vaccine)  A live, attenuated varicella virus vaccine used for immunization against chickenpox. It is recommended for children between the ages of 12 months and 13 years. | Varicella Vaccine  Oka Varicella Vaccine  Varivax | "Chickenpox Vaccine" [Mesh:NoExp]  OR "Varicella Vaccine" OR "Oka Varicella Vaccine " OR "Varivax"  [Vaccines](https://www.ncbi.nlm.nih.gov/mesh/68014612)  [Viral Vaccines](https://www.ncbi.nlm.nih.gov/mesh/68014765)  [Herpesvirus Vaccines](https://www.ncbi.nlm.nih.gov/mesh/68022283)  Chickenpox Vaccine  [Herpes Zoster Vaccine](https://www.ncbi.nlm.nih.gov/mesh/68053061) |

**Question 3.** What is the impact of the cs-DMARDs “com MTX, “LEF, SZL, HXQ) use on the response to inactivated vaccine in patients with immune-mediated rheumatic diseases?

| Group | **MeSH Terms**  **and definitions** | **Entry Terms** | **Search Key** |
| --- | --- | --- | --- |
| Population  Patients with immune-mediated rheumatic diseases “IMRD) | [Rheumatic Diseases - MeSH - NCBI “nih.gov)](https://www.ncbi.nlm.nih.gov/mesh/68012216)  Disorders of connective tissue, especially the joints and related structures, characterized by inflammation, degeneration, or metabolic derangement. | Rheumatic Disease  Rheumatism | "[Rheumatic Diseases](https://www.ncbi.nlm.nih.gov/mesh/68012216)" [Mesh:NoExp]  OR "Rheumatic Disease" OR "Rheumatism" OR  "Immune-mediated rheumatic diseases"  “não utilizar [Mesh] pois a árvore do termo inclui gota, fibromialgia e crianças) |
|  | [Arthritis, Rheumatoid - MeSH - NCBI “nih.gov)](https://www.ncbi.nlm.nih.gov/mesh/68001172)  A chronic systemic disease, primarily of the joints, marked by inflammatory changes in the synovial membranes and articular structures, widespread fibrinoid degeneration of the collagen fibers in mesenchymal tissues, and by atrophy and rarefaction of bony structures. Etiology is unknown, but autoimmune mechanisms have been implicated. | - Rheumatoid Arthritis | "[Arthritis, Rheumatoid](https://www.ncbi.nlm.nih.gov/mesh/68001172)" [Mesh:NoExp]  OR "Rheumatoid Arthritis" OR "[Rheumatoid Nodule](https://www.ncbi.nlm.nih.gov/mesh/68012218)" OR "[Rheumatoid Vasculitis](https://www.ncbi.nlm.nih.gov/mesh/68056653)" OR "[Sjogren's Syndrome](https://www.ncbi.nlm.nih.gov/mesh/68012859)"  “não utilizar [Mesh] pois a árvore do termo inclui caplan syndrome, felty syndrome) |
|  | [Spondylarthritis - MeSH - NCBI “nih.gov)](https://www.ncbi.nlm.nih.gov/mesh/68025241)  Inflammation of the joints of the SPINE, the intervertebral articulations. | - Spondylarthritides - Spinal Arthritis - Spinal Arthritides | "[Spondylarthritis](https://www.ncbi.nlm.nih.gov/mesh/68025241)" [Mesh] OR "[Spondylarthritis](https://www.ncbi.nlm.nih.gov/mesh/68025241)" OR "Spondylarthritides" OR "Spinal Arthritis" OR "Spinal Arthritides"  [Spondylitis](https://www.ncbi.nlm.nih.gov/mesh/68013166)  Spondylarthritis  [Osteoarthritis, Spine](https://www.ncbi.nlm.nih.gov/mesh/68055013)  [Spondylarthropathies](https://www.ncbi.nlm.nih.gov/mesh/68025242)  [Arthritis, Psoriatic](https://www.ncbi.nlm.nih.gov/mesh/68015535)  [Arthritis, Reactive](https://www.ncbi.nlm.nih.gov/mesh/68016918)  [Axial Spondyloarthritis](https://www.ncbi.nlm.nih.gov/mesh/2101123) + |
|  | [Arthritis, Psoriatic - MeSH - NCBI “nih.gov)](https://www.ncbi.nlm.nih.gov/mesh/68015535)  A type of inflammatory arthritis associated with PSORIASIS, often involving the axial joints and the peripheral terminal interphalangeal joints. It is characterized by the presence of HLA-B27-associated SPONDYLARTHROPATHY, and the absence of rheumatoid factor. | - Arthritic Psoriasis - Psoriatic Arthritis - Psoriasis Arthropathica - Psoriatic Arthropathy - Psoriatic Arthropathies | "[Arthritis, Psoriatic](https://www.ncbi.nlm.nih.gov/mesh/68015535)" [Mesh] OR "[Arthritis, Psoriatic](https://www.ncbi.nlm.nih.gov/mesh/68015535)" OR "Arthritic Psoriasis" OR "Psoriatic Arthritis" OR "Psoriasis Arthropathica" OR "Psoriatic Arthropathy" OR "Psoriatic Arthropathies"  [Spondylarthritis](https://www.ncbi.nlm.nih.gov/mesh/68025241)  [Spondylarthropathies](https://www.ncbi.nlm.nih.gov/mesh/68025242)  Arthritis, Psoriatic |
|  | [Spondylitis, Ankylosing - MeSH - NCBI “nih.gov)](https://www.ncbi.nlm.nih.gov/mesh/?term=ankylosing+spondylitis)  A chronic inflammatory condition affecting the axial joints, such as the SACROILIAC JOINT and other intervertebral or costovertebral joints. It occurs predominantly in young males and is characterized by pain and stiffness of joints “ANKYLOSIS) with inflammation at tendon insertions. | - Bechterew's Disease - Bechterews Disease - Marie Struempell Disease - Spondylarthritis Ankylopoietica - Spondyloarthritis Ankylopoietica - Ankylosing Spondylitis - Ankylosing Spondylarthritis - Ankylosing Spondylarthritides - Ankylosing Spondyloarthritis - Ankylosing Spondyloarthritides - Spondylitis Ankylopoietica - Bechterew Disease - Rheumatoid Spondylitis | "[Spondylitis, Ankylosing](https://www.ncbi.nlm.nih.gov/mesh/?term=ankylosing+spondylitis)" [Mesh] OR "[Spondylitis, Ankylosing](https://www.ncbi.nlm.nih.gov/mesh/?term=ankylosing+spondylitis)" OR "Bechterew's Disease" OR "Bechterews Disease" OR "Marie Struempell Disease" OR "Spondylarthritis Ankylopoietica" OR "Spondyloarthritis Ankylopoietica" OR "Ankylosing Spondylitis" OR "Ankylosing Spondylarthritis" OR "Ankylosing Spondylarthritides" OR "Ankylosing Spondyloarthritis" OR "Ankylosing Spondyloarthritides" OR "Spondylitis Ankylopoietica" OR "Bechterew Disease" OR "Rheumatoid Spondylitis"  [Spondylarthritis](https://www.ncbi.nlm.nih.gov/mesh/68025241)  [Spondylarthropathies](https://www.ncbi.nlm.nih.gov/mesh/68025242)  [Axial Spondyloarthritis](https://www.ncbi.nlm.nih.gov/mesh/2101123)  Spondylitis, Ankylosing |
|  | [Lupus Erythematosus, Systemic - MeSH - NCBI “nih.gov)](https://www.ncbi.nlm.nih.gov/mesh/68008180)  A chronic, relapsing, inflammatory, and often febrile multisystemic disorder of connective tissue, characterized principally by involvement of the skin, joints, kidneys, and serosal membranes. It is of unknown etiology, but is thought to represent a failure of the regulatory mechanisms of the autoimmune system. The disease is marked by a wide range of system dysfunctions, an elevated erythrocyte sedimentation rate, and the formation of LE cells in the blood or bone marrow. | - Systemic Lupus Erythematosus - Lupus Erythematosus Disseminatus - Libman Sacks Disease | "[Lupus Erythematosus, Systemic](https://www.ncbi.nlm.nih.gov/mesh/68008180)" [Mesh] OR "[Lupus Erythematosus, Systemic](https://www.ncbi.nlm.nih.gov/mesh/68008180)" OR "Systemic Lupus Erythematosus " OR "Lupus Erythematosus Disseminatus" OR "Libman Sacks Disease"  [Connective Tissue Diseases](https://www.ncbi.nlm.nih.gov/mesh/68003240)  Lupus Erythematosus, Systemic  [Lupus Nephritis](https://www.ncbi.nlm.nih.gov/mesh/68008181)  [Lupus Vasculitis, Central Nervous System](https://www.ncbi.nlm.nih.gov/mesh/68020945) |
|  | [Myositis - MeSH - NCBI “nih.gov)](https://www.ncbi.nlm.nih.gov/mesh/?term=inflammatory+myopathies)  Inflammation of a muscle or muscle tissue. | - Myositides - Inflammatory Muscle Diseases - Inflammatory Muscle Disease - Inflammatory Myopathy - Inflammatory Myopathies - Proliferative Myositides - Proliferative Myositis - Infectious Myositides - Infectious Myositis - Idiopathic Inflammatory Myopathies - Idiopathic Inflammatory Myopathy - Idiopathic Inflammatory Myositis - Focal Myositides - Focal Myositis | "[Myositis](https://www.ncbi.nlm.nih.gov/mesh/?term=inflammatory+myopathies)" [Mesh] OR "[Myositis](https://www.ncbi.nlm.nih.gov/mesh/?term=inflammatory+myopathies)" OR "Myositides" OR "Inflammatory Muscle Diseases" OR "Inflammatory Muscle Disease" OR "Inflammatory Myopathy" OR "Inflammatory Myopathies" OR "Proliferative Myositides" OR "Proliferative Myositis" OR "Infectious Myositides" OR "Infectious Myositis" OR "Idiopathic Inflammatory Myopathies" OR "Idiopathic Inflammatory Myopathy" OR "Idiopathic Inflammatory Myositis" OR "Focal Myositides" OR "Focal Myositis"  [Muscular Diseases](https://www.ncbi.nlm.nih.gov/mesh/68009135)  Myositis  [Myositis Ossificans](https://www.ncbi.nlm.nih.gov/mesh/68009221)  [Myositis, Inclusion Body](https://www.ncbi.nlm.nih.gov/mesh/68018979)  [Orbital Myositis](https://www.ncbi.nlm.nih.gov/mesh/68055622)  [Polymyositis](https://www.ncbi.nlm.nih.gov/mesh/68017285)  [Dermatomyositis](https://www.ncbi.nlm.nih.gov/mesh/68003882)  [Pyomyositis](https://www.ncbi.nlm.nih.gov/mesh/68052880) |
|  | [Systemic Vasculitis - MeSH - NCBI “nih.gov)](https://www.ncbi.nlm.nih.gov/mesh/?term=systemic+vasculitides)  A heterogeneous group of diseases characterized by inflammation and necrosis of the blood vessel walls. | - Systemic Vasculitides | "[Systemic Vasculitis](https://www.ncbi.nlm.nih.gov/mesh/?term=systemic+vasculitides)" [Mesh] OR "[Systemic Vasculitis](https://www.ncbi.nlm.nih.gov/mesh/?term=systemic+vasculitides)" OR "Systemic Vasculitides"  [Vasculitis](https://www.ncbi.nlm.nih.gov/mesh/68014657)  Systemic Vasculitis  [Anti-Neutrophil Cytoplasmic Antibody-Associated Vasculitis](https://www.ncbi.nlm.nih.gov/mesh/68056648)  [Churg-Strauss Syndrome](https://www.ncbi.nlm.nih.gov/mesh/68015267)  [Granulomatosis with Polyangiitis](https://www.ncbi.nlm.nih.gov/mesh/68014890)  [Microscopic Polyangiitis](https://www.ncbi.nlm.nih.gov/mesh/68055953)  [Polyarteritis Nodosa](https://www.ncbi.nlm.nih.gov/mesh/68010488)  [Rheumatoid Vasculitis](https://www.ncbi.nlm.nih.gov/mesh/68056653) |
|  | [Sjogren's Syndrome - MeSH - NCBI “nih.gov)](https://www.ncbi.nlm.nih.gov/mesh/68012859)  Chronic inflammatory and autoimmune disease in which the salivary and lacrimal glands undergo progressive destruction by lymphocytes and plasma cells resulting in decreased production of saliva and tears. The primary form, often called sicca syndrome, involves both KERATOCONJUNCTIVITIS SICCA and XEROSTOMIA. The secondary form includes, in addition, the presence of a connective tissue disease, usually rheumatoid arthritis. | - Sjogrens Syndrome - Sjogren Syndrome - Sicca Syndrome | "[Sjogren's Syndrome](https://www.ncbi.nlm.nih.gov/mesh/68012859)" [Mesh] OR "[Sjogren's Syndrome](https://www.ncbi.nlm.nih.gov/mesh/68012859)"  OR "Sjogrens Syndrome" OR "Sjogren Syndrome" OR "Sicca Syndrome"  [Arthritis](https://www.ncbi.nlm.nih.gov/mesh/68001168)  [Arthritis, Rheumatoid](https://www.ncbi.nlm.nih.gov/mesh/68001172)  Sjogren's Syndrome |
|  | [Scleroderma, Systemic - MeSH - NCBI “nih.gov)](https://www.ncbi.nlm.nih.gov/mesh/68012595)  A chronic multi-system disorder of CONNECTIVE TISSUE. It is characterized by SCLEROSIS in the SKIN, the LUNGS, the HEART, the GASTROINTESTINAL TRACT, the KIDNEYS, and the MUSCULOSKELETAL SYSTEM. Other important features include diseased small BLOOD VESSELS and AUTOANTIBODIES. The disorder is named for its most prominent feature “hard skin), and classified into subsets by the extent of skin thickening: LIMITED SCLERODERMA and DIFFUSE SCLERODERMA. | - Systemic Sclerosis - Systemic Scleroderma | "[Scleroderma, Systemic](https://www.ncbi.nlm.nih.gov/mesh/68012595)" [Mesh] OR "[Scleroderma, Systemic](https://www.ncbi.nlm.nih.gov/mesh/68012595)" OR "Systemic Sclerosis" OR "Systemic Scleroderma"  [Connective Tissue Diseases](https://www.ncbi.nlm.nih.gov/mesh/68003240)  Scleroderma, Systemic  [Scleroderma, Diffuse](https://www.ncbi.nlm.nih.gov/mesh/68045743)  [Scleroderma, Limited](https://www.ncbi.nlm.nih.gov/mesh/68045745)  [CREST Syndrome](https://www.ncbi.nlm.nih.gov/mesh/68017675) |
|  | [Mixed Connective Tissue Disease - MeSH - NCBI “nih.gov)](https://www.ncbi.nlm.nih.gov/mesh/?term=Mixed+connective+tissue+disease)  A syndrome with overlapping clinical features of systemic lupus erythematosus, scleroderma, polymyositis, and Raynaud's phenomenon. The disease is differentially characterized by high serum titers of antibodies to ribonuclease-sensitive extractable “saline soluble) nuclear antigen and a "speckled" epidermal nuclear staining pattern on direct immunofluorescence. | - Sharp Syndrome - MCTD | "[Mixed Connective Tissue Disease](https://www.ncbi.nlm.nih.gov/mesh/?term=Mixed+connective+tissue+disease)" [Mesh] OR "[Mixed Connective Tissue Disease](https://www.ncbi.nlm.nih.gov/mesh/?term=Mixed+connective+tissue+disease)" OR "Sharp Syndrome" OR "MCTD"  [Connective Tissue Diseases](https://www.ncbi.nlm.nih.gov/mesh/68003240)  Mixed Connective Tissue Disease |
| Intervention cs-DMARDs  conventional synthetic disease modifying anti-rheumatic drugs | “cs-DMARDs” OR “conventional synthetic disease modifying anti-rheumatic drugs” |  | "cs-DMARDs" OR "conventional synthetic disease modifying anti-rheumatic drugs" |
|  | [Leflunomide - MeSH - NCBI “nih.gov)](https://www.ncbi.nlm.nih.gov/mesh/2028012)  An isoxazole derivative that inhibits dihydroorotate dehydrogenase, the fourth enzyme in the pyrimidine biosynthetic pathway. It is used an immunosuppressive agent in the treatment of RHEUMATOID ARTHRITIS and PSORIATIC ARTHRITIS. | - N-”4-Trifluoromethyphenyl)-5-methylisoxazole-4-carboxamide - HWA 486 - HWA486 - SU101 - Arava | "Leflunomide" [Mesh] OR "Leflunomide" OR " N-”4-Trifluoromethyphenyl)-5-methylisoxazole-4-carboxamide" OR "HWA 486" OR "HWA486" OR "SU101" OR "Arava"  [Chemicals and Drugs Category](https://www.ncbi.nlm.nih.gov/mesh/1000068)  [Heterocyclic Compounds](https://www.ncbi.nlm.nih.gov/mesh/68006571)  [Heterocyclic Compounds, 1-Ring](https://www.ncbi.nlm.nih.gov/mesh/68006573)  [Azoles](https://www.ncbi.nlm.nih.gov/mesh/68001393)  [Isoxazoles](https://www.ncbi.nlm.nih.gov/mesh/68007555)  Leflunomide |
|  | [Sulfasalazine - MeSH - NCBI “nih.gov)](https://www.ncbi.nlm.nih.gov/mesh/?term=Sulfasalazine)  A drug that is used in the management of inflammatory bowel diseases. Its activity is generally considered to lie in its metabolic breakdown product, 5-aminosalicylic acid “see MESALAMINE) released in the colon. “From Martindale, The Extra Pharmacopoeia, 30th ed, p907) | - Salicylazosulfapyridine - Sulphasalazine - Salazosulfapyridine - Pyralin EN - Azulfadine - Azulfidine EN - Azulfidine - Asulfidine - Colo Pleon - Pleon - Ulcol - Sulfasalazin medac - Sulfasalazin Heyl - Sulfasalazine FNA - Ucine - Salazopyrin   ratio Sulfasalazine | "Sulfasalazine" [Mesh] OR "Sulfasalazine" OR "Salicylazosulfapyridine" OR "Sulphasalazine" OR "Salazosulfapyridine" OR "Pyralin EN" OR "Azulfadine" OR "Azulfidine EN" OR "Azulfidine" OR "Asulfidine" OR "Colo Pleon" OR "Pleon" OR "Ulcol" OR "Sulfasalazin medac" OR "Sulfasalazin Heyl" OR "Sulfasalazine FNA" OR "Ucine" OR "Salazopyrin ratio Sulfasalazine"  Sulfasalazine  [All MeSH Categories](https://www.ncbi.nlm.nih.gov/mesh/1000048)  [Chemicals and Drugs Category](https://www.ncbi.nlm.nih.gov/mesh/1000068)  [Organic Chemicals](https://www.ncbi.nlm.nih.gov/mesh/68009930)  [Sulfur Compounds](https://www.ncbi.nlm.nih.gov/mesh/68013457)  [Sulfones](https://www.ncbi.nlm.nih.gov/mesh/68013450)  [Sulfonamides](https://www.ncbi.nlm.nih.gov/mesh/68013449)  Sulfasalazine |
|  | [Chloroquine - MeSH - NCBI “nih.gov)](https://www.ncbi.nlm.nih.gov/mesh/68002738)  The prototypical antimalarial agent with a mechanism that is not well understood. It has also been used to treat rheumatoid arthritis, systemic lupus erythematosus, and in the systemic therapy of amebic liver abscesses. | - Chlorochin - Chingamin - Khingamin - Nivaquine - Chloroquine Sulfate   Chloroquine Sulphate  Aralen   - Arequin - Arechine | "Chloroquine" [Mesh] OR "Chloroquine"  OR "Chlorochin" OR "Chingamin" OR "Khingamin" OR "Nivaquine" OR "Chloroquine Sulfate" OR "Chloroquine Sulphate" OR "Aralen" OR "Arequin" OR "Arechine"  [All MeSH Categories](https://www.ncbi.nlm.nih.gov/mesh/1000048)  [Chemicals and Drugs Category](https://www.ncbi.nlm.nih.gov/mesh/1000068)  [Heterocyclic Compounds](https://www.ncbi.nlm.nih.gov/mesh/68006571)  [Heterocyclic Compounds, Fused-Ring](https://www.ncbi.nlm.nih.gov/mesh/2016711)  [Heterocyclic Compounds, 2-Ring](https://www.ncbi.nlm.nih.gov/mesh/68006574)  [Quinolines](https://www.ncbi.nlm.nih.gov/mesh/68011804)  [Aminoquinolines](https://www.ncbi.nlm.nih.gov/mesh/68000634)  Chloroquine  [Hydroxychloroquine](https://www.ncbi.nlm.nih.gov/mesh/68006886) |
|  | [Hydroxychloroquine - MeSH - NCBI “nih.gov)](https://www.ncbi.nlm.nih.gov/mesh/?term=Hydroxychloroquine)    A chemotherapeutic agent that acts against erythrocytic forms of malarial parasites. Hydroxychloroquine appears to concentrate in food vacuoles of affected protozoa. It inhibits plasmodial heme polymerase. “From Gilman et al., Goodman and Gilman's The Pharmacological Basis of Therapeutics, 9th ed, p970) | - Oxychlorochin - Oxychloroquine - Hydroxychlorochin - Plaquenil - Hydroxychloroquine Sulfate | "Hydroxychloroquine" [Mesh] OR Hydroxychloroquine"  OR "Oxychlorochin"  OR "Oxychloroquine"  OR "Hydroxychlorochin"  OR "Plaquenil"  OR "Hydroxychloroquine Sulfate"  [Aminoquinolines](https://www.ncbi.nlm.nih.gov/mesh/68000634)  [Chloroquine](https://www.ncbi.nlm.nih.gov/mesh/68002738)  Hydroxychloroquine |
|  | [Methotrexate - MeSH - NCBI “nih.gov)](https://www.ncbi.nlm.nih.gov/mesh/68008727)  An antineoplastic antimetabolite with immunosuppressant properties. It is an inhibitor of TETRAHYDROFOLATE DEHYDROGENASE and prevents the formation of tetrahydrofolate, necessary for synthesis of thymidylate, an essential component of DNA. | - Amethopterin - Mexate - Methotrexate Sodium - Methotrexate Hydrate - Dicesium Salt Methotrexate | "Methotrexate" [Mesh] OR "Methotrexate" OR "Amethopterin Mexate" OR "Methotrexate Sodium" OR "Methotrexate Hydrate" OR "Dicesium Salt Methotrexate"  [Pteridines](https://www.ncbi.nlm.nih.gov/mesh/68011621)  [Pterins](https://www.ncbi.nlm.nih.gov/mesh/68011622)  [Aminopterin](https://www.ncbi.nlm.nih.gov/mesh/68000630)  Methotrexate |
| Outcomes | [Vaccines, Inactivated - MeSH - NCBI “nih.gov)](https://www.ncbi.nlm.nih.gov/mesh/68015164)  Definition: Vaccines in which the infectious microbial nucleic acid components have been destroyed by chemical or physical treatment “e.g., formalin, beta-propiolactone, gamma radiation) without affecting the antigenicity or immunogenicity of the viral coat or bacterial outer membrane proteins. | Inactivated Vaccines  Killed Vaccine  Killed Vaccines  Inactivated Vaccine | "[Vaccines, Inactivated](https://www.ncbi.nlm.nih.gov/mesh/68015164)" [Mesh] OR "[Vaccines, Inactivated](https://www.ncbi.nlm.nih.gov/mesh/68015164)" OR "Inactivated Vaccines" OR "Killed Vaccine" OR "Killed Vaccines" OR "Inactivated Vaccine"  Vaccines, Inactivated  [Poliovirus Vaccine, Inactivated](https://www.ncbi.nlm.nih.gov/mesh/68011054) |
|  | [Influenza Vaccines - MeSH - NCBI “nih.gov)](https://www.ncbi.nlm.nih.gov/mesh/68007252)  Vaccines used to prevent infection by viruses in the family ORTHOMYXOVIRIDAE. It includes both killed and attenuated vaccines. The composition of the vaccines is changed each year in response to antigenic shifts and changes in prevalence of influenza virus strains. The flu vaccines may be mono- or multi-valent, which contains one or more INFLUENZAVIRUS A and INFLUENZAVIRUS B strains. | - Influenza Vaccine - Influenzavirus Vaccines - Influenza Virus Vaccines - Influenzavirus Vaccine - Influenza Virus Vaccine - High-Dose Trivalent Influenza Vaccine - High Dose Trivalent Influenza Vaccine - Trivalent Influenza Vaccine - Quadrivalent Influenza Vaccine - High dose | "[Influenza Vaccines](https://www.ncbi.nlm.nih.gov/mesh/68007252)"[Mesh:NoExp]   OR Influenza Vaccine" OR "Influenzavirus Vaccines" OR "Influenza Virus Vaccines" OR "Influenzavirus Vaccine" OR "Influenza Virus Vaccine" OR "High-Dose Trivalent Influenza Vaccine" OR "High Dose Trivalent Influenza Vaccine" OR "Trivalent Influenza Vaccine" OR "Quadrivalent Influenza Vaccine"  “não utilizar [Mesh] pois a árvore do termo inclui VVA e intra-nasal)  [Vaccines](https://www.ncbi.nlm.nih.gov/mesh/68014612)  [Viral Vaccines](https://www.ncbi.nlm.nih.gov/mesh/68014765)  Influenza Vaccines |
|  | [Pneumococcal Vaccines - MeSH - NCBI “nih.gov)](https://www.ncbi.nlm.nih.gov/mesh/68022242)  Vaccines or candidate vaccines used to prevent infections with STREPTOCOCCUS PNEUMONIAE. | - Pneumococcal Vaccine - Pneumovax - Pneumococcal Polysaccharide Vaccine - Pnu Imune Vaccine - PnuImune Vaccine - Conjugated penumococcal vaccine | "[Pneumococcal Vaccines](https://www.ncbi.nlm.nih.gov/mesh/68022242)" [Mesh] OR "[Pneumococcal Vaccines](https://www.ncbi.nlm.nih.gov/mesh/68022242)" OR "Pneumococcal Vaccine" OR "Pneumovax" OR "Pneumococcal Polysaccharide Vaccine" OR "Pnu Imune Vaccine" OR "PnuImune Vaccine" OR "Conjugated penumococcal vaccine"  Pneumococcal Vaccines  [Heptavalent Pneumococcal Conjugate Vaccine](https://www.ncbi.nlm.nih.gov/mesh/2009980)  13 pneumococcal conjugate vaccine |
|  | [Herpes Zoster Vaccine - MeSH - NCBI “nih.gov)](https://www.ncbi.nlm.nih.gov/mesh/68053061)  An attenuated vaccine used to prevent and/or treat HERPES ZOSTER, a disease caused by HUMAN HERPESVIRUS 3. | - Zoster Vaccine - Shingles Vaccine | "Herpes Zoster Vaccine" [Mesh:NoExp]  OR " Zoster Vaccine" OR "Shingles Vaccine"  “não utilizar [Mesh] pois a árvore do termo inclui live attenuated zoster  e intra-nasal)  [Vaccines](https://www.ncbi.nlm.nih.gov/mesh/68014612)  [Viral Vaccines](https://www.ncbi.nlm.nih.gov/mesh/68014765)  [Herpesvirus Vaccines](https://www.ncbi.nlm.nih.gov/mesh/68022283)  Herpes Zoster Vaccine |
|  | [Hepatitis B Vaccines - MeSH - NCBI “nih.gov)](https://www.ncbi.nlm.nih.gov/mesh/68017325)  Vaccines or candidate vaccines containing inactivated hepatitis B or some of its component antigens and designed to prevent hepatitis B. Some vaccines may be recombinantly produced. | - Hepatitis B Vaccine | "Hepatitis B Vaccines" [Mesh] OR "Hepatitis B Vaccines" OR "Hepatitis B Vaccine"  [Vaccines](https://www.ncbi.nlm.nih.gov/mesh/68014612)  [Viral Vaccines](https://www.ncbi.nlm.nih.gov/mesh/68014765)  [Viral Hepatitis Vaccines](https://www.ncbi.nlm.nih.gov/mesh/68014761)  Hepatitis B Vaccines |
|  | [Papillomavirus Vaccines - MeSH - NCBI “nih.gov)](https://www.ncbi.nlm.nih.gov/mesh/?term=papillomavirus+vaccines)   Vaccines or candidate vaccines used to prevent PAPILLOMAVIRUS INFECTIONS. Human vaccines are intended to reduce the incidence of UTERINE CERVICAL NEOPLASMS, so they are sometimes considered a type of CANCER VACCINES. They are often composed of CAPSID PROTEINS, especially L1 protein, from various types of ALPHAPAPILLOMAVIRUS | - Papillomavirus Vaccine   Human Papillomavirus Vaccines   - HPV Vaccine   Human Papilloma Virus Vaccine   - HPV Vaccines - Human Papilloma Virus Vaccines - Human Papillomavirus Vaccine | "Papillomavirus Vaccines" [Mesh] OR "Papillomavirus Vaccines" OR "Papillomavirus Vaccine" OR "Human Papillomavirus Vaccines" OR "HPV Vaccine" OR "Human Papilloma Virus Vaccine" OR "HPV Vaccines" OR "Human Papilloma Virus Vaccines" OR "Human Papillomavirus Vaccine"  [Vaccines](https://www.ncbi.nlm.nih.gov/mesh/68014612)  [Viral Vaccines](https://www.ncbi.nlm.nih.gov/mesh/68014765)  Papillomavirus Vaccines  [Human Papillomavirus](https://www.ncbi.nlm.nih.gov/mesh/2009889) |
|  | [Poliovirus Vaccines - MeSH - NCBI “nih.gov)](https://www.ncbi.nlm.nih.gov/mesh/68023321)    Vaccines used to prevent POLIOMYELITIS. They include inactivated “POLIOVIRUS VACCINE, INACTIVATED) | - Poliovirus Vaccine - salk | "Poliovirus Vaccines" [Mesh] OR "Poliovirus Vaccines" OR "Poliovirus Vaccine" OR "salk"  [Vaccines](https://www.ncbi.nlm.nih.gov/mesh/68014612)  [Viral Vaccines](https://www.ncbi.nlm.nih.gov/mesh/68014765)  [Poliovirus Vaccine, Inactivated](https://www.ncbi.nlm.nih.gov/mesh/68011054) |
|  | [Hepatitis A Vaccines - MeSH - NCBI “nih.gov)](https://www.ncbi.nlm.nih.gov/mesh/68022362)    Vaccines or candidate vaccines used to prevent infection with hepatitis A virus “HEPATOVIRUS). | - Hepatovirus Vaccines - Hepatitis A Vaccine - Vaqta - Havrix | "Hepatitis A Vaccines" [Mesh] OR "Hepatitis A Vaccines" OR "Hepatovirus Vaccines" OR "Hepatitis A Vaccine" OR "Vaqta" OR "Havrix"  [Vaccines](https://www.ncbi.nlm.nih.gov/mesh/68014612)  [Viral Hepatitis Vaccines](https://www.ncbi.nlm.nih.gov/mesh/68014761)  Hepatitis A Vaccines |
|  | [Meningococcal Vaccines - MeSH - NCBI “nih.gov)](https://www.ncbi.nlm.nih.gov/mesh/68022401)    Vaccines or candidate vaccines used to prevent infection with NEISSERIA MENINGITIDIS. | Meningococcal Vaccine  Menactra  Tetravalent Meningococcal Vaccine  Menomune  Menveo  Bivalent Meningococcal Vaccine  Meningococcal Polysaccharide Vaccine | "Meningococcal Vaccines" [Mesh] OR " Meningococcal Vaccine" OR "Menactra" OR "Tetravalent Meningococcal Vaccine" OR "Menomune OR "Menveo" OR "Bivalent Meningococcal Vaccine" OR "Meningococcal Polysaccharide Vaccine"    [Vaccines](https://www.ncbi.nlm.nih.gov/mesh/68014612)  Meningococcal Vaccines C, B or ACWY |
|  | [Diphtheria-Tetanus-Pertussis Vaccine - MeSH - NCBI “nih.gov)](https://www.ncbi.nlm.nih.gov/mesh/?term=vaccine+per)   A vaccine consisting of DIPHTHERIA TOXOID; TETANUS TOXOID; and whole-cell PERTUSSIS VACCINE. The vaccine protects against diphtheria, tetanus, and whooping cough. | Diphtheria Tetanus Pertussis Vaccine  DTwP Vaccine  DPT Vaccine  DTP Vaccine  Di Te Per Vaccine  Diphtheria Pertussis Tetanus Vaccine | "Diphtheria-Tetanus-Pertussis Vaccine" [Mesh] OR "Diphtheria-Tetanus-Pertussis Vaccine" OR " Diphtheria Tetanus Pertussis Vaccine" OR "DTwP Vaccine" OR "DPT Vaccine" OR "DTP Vaccine" OR "Di Te Per Vaccine" OR "Diphtheria Pertussis Tetanus Vaccine"  [Vaccines](https://www.ncbi.nlm.nih.gov/mesh/68014612)  [Pertussis Vaccine](https://www.ncbi.nlm.nih.gov/mesh/68010567)  Diphtheria-Tetanus-Pertussis Vaccine |
|  | [Haemophilus influenza type b polysaccharide vaccine-tetanus toxin conj - MeSH - NCBI “nih.gov)](https://www.ncbi.nlm.nih.gov/mesh/?term=haemophilus+influenza+type+b+polysaccharide+vaccine+tetanus+toxin+conjugate)    the type b capsular polysaccharide of HIB-vaccine is conjugated to tetanus toxoid | HIBest  PRP-T  Hib-TT  Hib capsular polysaccharide-tetanus vaccine  Hib CPS-tetanus vaccine  OmniHIB  ActHIB | "Haemophilus influenza type b polysaccharide vaccine-tetanus toxin conjugate" OR "HIBest" OR "PRP-T" OR "Hib-TT" OR "Hib capsular polysaccharide-tetanus vaccine" OR "Hib CPS-tetanus vaccine" OR "OmniHIB" OR "ActHIB" |

**Question 04.** What is the impact of the immunosuppressants “azathioprine, MMF, cyclosporine, cyclophosphamide) use on the response to inactivated vaccine in patients with immune-mediated rheumatic diseases?

| Group | MeSH Terms  and definitions | Entry Terms | Search Key |
| --- | --- | --- | --- |
| Population  Patients with immune-mediated rheumatic diseases “IMRD) | [Rheumatic Diseases - MeSH - NCBI “nih.gov)](https://www.ncbi.nlm.nih.gov/mesh/68012216)  Disorders of connective tissue, especially the joints and related structures, characterized by inflammation, degeneration, or metabolic derangement. | Rheumatic Disease  Rheumatism | "[Rheumatic Diseases](https://www.ncbi.nlm.nih.gov/mesh/68012216)"[Mesh:NoExp]   OR "Rheumatic Disease" OR "Rheumatism" OR  "Immune-mediated rheumatic diseases"  “não utilizar [Mesh] pois a árvore do termo inclui gota, fibromialgia e crianças) |
|  | [Arthritis, Rheumatoid - MeSH - NCBI “nih.gov)](https://www.ncbi.nlm.nih.gov/mesh/68001172)  A chronic systemic disease, primarily of the joints, marked by inflammatory changes in the synovial membranes and articular structures, widespread fibrinoid degeneration of the collagen fibers in mesenchymal tissues, and by atrophy and rarefaction of bony structures. Etiology is unknown, but autoimmune mechanisms have been implicated. | - Rheumatoid Arthritis | "[Arthritis, Rheumatoid](https://www.ncbi.nlm.nih.gov/mesh/68001172)" [Mesh:NoExp]  OR "Rheumatoid Arthritis" OR "[Rheumatoid Nodule](https://www.ncbi.nlm.nih.gov/mesh/68012218)" OR "[Rheumatoid Vasculitis](https://www.ncbi.nlm.nih.gov/mesh/68056653)" OR "[Sjogren's Syndrome](https://www.ncbi.nlm.nih.gov/mesh/68012859)"  “não utilizar [Mesh] pois a árvore do termo inclui caplan syndrome, felty syndrome) |
|  | [Spondylarthritis - MeSH - NCBI “nih.gov)](https://www.ncbi.nlm.nih.gov/mesh/68025241)  Inflammation of the joints of the SPINE, the intervertebral articulations. | - Spondylarthritides - Spinal Arthritis - Spinal Arthritides | "[Spondylarthritis](https://www.ncbi.nlm.nih.gov/mesh/68025241)" [Mesh] OR "[Spondylarthritis](https://www.ncbi.nlm.nih.gov/mesh/68025241)" OR "Spondylarthritides" OR "Spinal Arthritis" OR "Spinal Arthritides"  [Spondylitis](https://www.ncbi.nlm.nih.gov/mesh/68013166)  Spondylarthritis  [Osteoarthritis, Spine](https://www.ncbi.nlm.nih.gov/mesh/68055013)  [Spondylarthropathies](https://www.ncbi.nlm.nih.gov/mesh/68025242)  [Arthritis, Psoriatic](https://www.ncbi.nlm.nih.gov/mesh/68015535)  [Arthritis, Reactive](https://www.ncbi.nlm.nih.gov/mesh/68016918)  [Axial Spondyloarthritis](https://www.ncbi.nlm.nih.gov/mesh/2101123) + |
|  | [Arthritis, Psoriatic - MeSH - NCBI “nih.gov)](https://www.ncbi.nlm.nih.gov/mesh/68015535)  A type of inflammatory arthritis associated with PSORIASIS, often involving the axial joints and the peripheral terminal interphalangeal joints. It is characterized by the presence of HLA-B27-associated SPONDYLARTHROPATHY, and the absence of rheumatoid factor. | - Arthritic Psoriasis - Psoriatic Arthritis - Psoriasis Arthropathica - Psoriatic Arthropathy - Psoriatic Arthropathies | "[Arthritis, Psoriatic](https://www.ncbi.nlm.nih.gov/mesh/68015535)" [Mesh] OR "[Arthritis, Psoriatic](https://www.ncbi.nlm.nih.gov/mesh/68015535)" OR "Arthritic Psoriasis" OR "Psoriatic Arthritis" OR "Psoriasis Arthropathica" OR "Psoriatic Arthropathy" OR "Psoriatic Arthropathies"  [Spondylarthritis](https://www.ncbi.nlm.nih.gov/mesh/68025241)  [Spondylarthropathies](https://www.ncbi.nlm.nih.gov/mesh/68025242)  Arthritis, Psoriatic |
|  | [Spondylitis, Ankylosing - MeSH - NCBI “nih.gov)](https://www.ncbi.nlm.nih.gov/mesh/?term=ankylosing+spondylitis)  A chronic inflammatory condition affecting the axial joints, such as the SACROILIAC JOINT and other intervertebral or costovertebral joints. It occurs predominantly in young males and is characterized by pain and stiffness of joints “ANKYLOSIS) with inflammation at tendon insertions. | - Bechterew's Disease - Bechterews Disease - Marie Struempell Disease - Spondylarthritis Ankylopoietica - Spondyloarthritis Ankylopoietica - Ankylosing Spondylitis - Ankylosing Spondylarthritis - Ankylosing Spondylarthritides - Ankylosing Spondyloarthritis - Ankylosing Spondyloarthritides - Spondylitis Ankylopoietica - Bechterew Disease - Rheumatoid Spondylitis | "[Spondylitis, Ankylosing](https://www.ncbi.nlm.nih.gov/mesh/?term=ankylosing+spondylitis)" [Mesh] OR "[Spondylitis, Ankylosing](https://www.ncbi.nlm.nih.gov/mesh/?term=ankylosing+spondylitis)" OR "Bechterew's Disease" OR "Bechterews Disease" OR "Marie Struempell Disease" OR "Spondylarthritis Ankylopoietica" OR "Spondyloarthritis Ankylopoietica" OR "Ankylosing Spondylitis" OR "Ankylosing Spondylarthritis" OR "Ankylosing Spondylarthritides" OR "Ankylosing Spondyloarthritis" OR "Ankylosing Spondyloarthritides" OR "Spondylitis Ankylopoietica" OR "Bechterew Disease" OR "Rheumatoid Spondylitis"  [Spondylarthritis](https://www.ncbi.nlm.nih.gov/mesh/68025241)  [Spondylarthropathies](https://www.ncbi.nlm.nih.gov/mesh/68025242)  [Axial Spondyloarthritis](https://www.ncbi.nlm.nih.gov/mesh/2101123)  Spondylitis, Ankylosing |
|  | [Lupus Erythematosus, Systemic - MeSH - NCBI “nih.gov)](https://www.ncbi.nlm.nih.gov/mesh/68008180)  A chronic, relapsing, inflammatory, and often febrile multisystemic disorder of connective tissue, characterized principally by involvement of the skin, joints, kidneys, and serosal membranes. It is of unknown etiology, but is thought to represent a failure of the regulatory mechanisms of the autoimmune system. The disease is marked by a wide range of system dysfunctions, an elevated erythrocyte sedimentation rate, and the formation of LE cells in the blood or bone marrow. | - Systemic Lupus Erythematosus - Lupus Erythematosus Disseminatus - Libman Sacks Disease | "[Lupus Erythematosus, Systemic](https://www.ncbi.nlm.nih.gov/mesh/68008180)" [Mesh] OR "[Lupus Erythematosus, Systemic](https://www.ncbi.nlm.nih.gov/mesh/68008180)" OR "Systemic Lupus Erythematosus " OR "Lupus Erythematosus Disseminatus" OR "Libman Sacks Disease"  [Connective Tissue Diseases](https://www.ncbi.nlm.nih.gov/mesh/68003240)  Lupus Erythematosus, Systemic  [Lupus Nephritis](https://www.ncbi.nlm.nih.gov/mesh/68008181)  [Lupus Vasculitis, Central Nervous System](https://www.ncbi.nlm.nih.gov/mesh/68020945) |
|  | [Myositis - MeSH - NCBI “nih.gov)](https://www.ncbi.nlm.nih.gov/mesh/?term=inflammatory+myopathies)  Inflammation of a muscle or muscle tissue. | - Myositides - Inflammatory Muscle Diseases - Inflammatory Muscle Disease - Inflammatory Myopathy - Inflammatory Myopathies - Proliferative Myositides - Proliferative Myositis - Infectious Myositides - Infectious Myositis - Idiopathic Inflammatory Myopathies - Idiopathic Inflammatory Myopathy - Idiopathic Inflammatory Myositis - Focal Myositides - Focal Myositis | "[Myositis](https://www.ncbi.nlm.nih.gov/mesh/?term=inflammatory+myopathies)" [Mesh] OR "[Myositis](https://www.ncbi.nlm.nih.gov/mesh/?term=inflammatory+myopathies)" OR "Myositides" OR "Inflammatory Muscle Diseases" OR "Inflammatory Muscle Disease" OR "Inflammatory Myopathy" OR "Inflammatory Myopathies" OR "Proliferative Myositides" OR "Proliferative Myositis" OR "Infectious Myositides" OR "Infectious Myositis" OR "Idiopathic Inflammatory Myopathies" OR "Idiopathic Inflammatory Myopathy" OR "Idiopathic Inflammatory Myositis" OR "Focal Myositides" OR "Focal Myositis"  [Muscular Diseases](https://www.ncbi.nlm.nih.gov/mesh/68009135)  Myositis  [Myositis Ossificans](https://www.ncbi.nlm.nih.gov/mesh/68009221)  [Myositis, Inclusion Body](https://www.ncbi.nlm.nih.gov/mesh/68018979)  [Orbital Myositis](https://www.ncbi.nlm.nih.gov/mesh/68055622)  [Polymyositis](https://www.ncbi.nlm.nih.gov/mesh/68017285)  [Dermatomyositis](https://www.ncbi.nlm.nih.gov/mesh/68003882)  [Pyomyositis](https://www.ncbi.nlm.nih.gov/mesh/68052880) |
|  | [Systemic Vasculitis - MeSH - NCBI “nih.gov)](https://www.ncbi.nlm.nih.gov/mesh/?term=systemic+vasculitides)  A heterogeneous group of diseases characterized by inflammation and necrosis of the blood vessel walls. | - Systemic Vasculitides | "[Systemic Vasculitis](https://www.ncbi.nlm.nih.gov/mesh/?term=systemic+vasculitides)" [Mesh] OR "[Systemic Vasculitis](https://www.ncbi.nlm.nih.gov/mesh/?term=systemic+vasculitides)" OR "Systemic Vasculitides"  [Vasculitis](https://www.ncbi.nlm.nih.gov/mesh/68014657)  Systemic Vasculitis  [Anti-Neutrophil Cytoplasmic Antibody-Associated Vasculitis](https://www.ncbi.nlm.nih.gov/mesh/68056648)  [Churg-Strauss Syndrome](https://www.ncbi.nlm.nih.gov/mesh/68015267)  [Granulomatosis with Polyangiitis](https://www.ncbi.nlm.nih.gov/mesh/68014890)  [Microscopic Polyangiitis](https://www.ncbi.nlm.nih.gov/mesh/68055953)  [Polyarteritis Nodosa](https://www.ncbi.nlm.nih.gov/mesh/68010488)  [Rheumatoid Vasculitis](https://www.ncbi.nlm.nih.gov/mesh/68056653) |
|  | [Sjogren's Syndrome - MeSH - NCBI “nih.gov)](https://www.ncbi.nlm.nih.gov/mesh/68012859)  Chronic inflammatory and autoimmune disease in which the salivary and lacrimal glands undergo progressive destruction by lymphocytes and plasma cells resulting in decreased production of saliva and tears. The primary form, often called sicca syndrome, involves both KERATOCONJUNCTIVITIS SICCA and XEROSTOMIA. The secondary form includes, in addition, the presence of a connective tissue disease, usually rheumatoid arthritis. | - Sjogrens Syndrome - Sjogren Syndrome - Sicca Syndrome | "[Sjogren's Syndrome](https://www.ncbi.nlm.nih.gov/mesh/68012859)" [Mesh] OR "[Sjogren's Syndrome](https://www.ncbi.nlm.nih.gov/mesh/68012859)"  OR "Sjogrens Syndrome" OR "Sjogren Syndrome" OR "Sicca Syndrome"  [Arthritis](https://www.ncbi.nlm.nih.gov/mesh/68001168)  [Arthritis, Rheumatoid](https://www.ncbi.nlm.nih.gov/mesh/68001172)  Sjogren's Syndrome |
|  | [Scleroderma, Systemic - MeSH - NCBI “nih.gov)](https://www.ncbi.nlm.nih.gov/mesh/68012595)  A chronic multi-system disorder of CONNECTIVE TISSUE. It is characterized by SCLEROSIS in the SKIN, the LUNGS, the HEART, the GASTROINTESTINAL TRACT, the KIDNEYS, and the MUSCULOSKELETAL SYSTEM. Other important features include diseased small BLOOD VESSELS and AUTOANTIBODIES. The disorder is named for its most prominent feature “hard skin), and classified into subsets by the extent of skin thickening: LIMITED SCLERODERMA and DIFFUSE SCLERODERMA. | - Systemic Sclerosis - Systemic Scleroderma | "[Scleroderma, Systemic](https://www.ncbi.nlm.nih.gov/mesh/68012595)" [Mesh] OR "[Scleroderma, Systemic](https://www.ncbi.nlm.nih.gov/mesh/68012595)" OR "Systemic Sclerosis" OR "Systemic Scleroderma"  [Connective Tissue Diseases](https://www.ncbi.nlm.nih.gov/mesh/68003240)  Scleroderma, Systemic  [Scleroderma, Diffuse](https://www.ncbi.nlm.nih.gov/mesh/68045743)  [Scleroderma, Limited](https://www.ncbi.nlm.nih.gov/mesh/68045745)  [CREST Syndrome](https://www.ncbi.nlm.nih.gov/mesh/68017675) |
|  | [Mixed Connective Tissue Disease - MeSH - NCBI “nih.gov)](https://www.ncbi.nlm.nih.gov/mesh/?term=Mixed+connective+tissue+disease)  A syndrome with overlapping clinical features of systemic lupus erythematosus, scleroderma, polymyositis, and Raynaud's phenomenon. The disease is differentially characterized by high serum titers of antibodies to ribonuclease-sensitive extractable “saline soluble) nuclear antigen and a "speckled" epidermal nuclear staining pattern on direct immunofluorescence. | - Sharp Syndrome - MCTD | "[Mixed Connective Tissue Disease](https://www.ncbi.nlm.nih.gov/mesh/?term=Mixed+connective+tissue+disease)" [Mesh] OR "[Mixed Connective Tissue Disease](https://www.ncbi.nlm.nih.gov/mesh/?term=Mixed+connective+tissue+disease)" OR "Sharp Syndrome" OR "MCTD"  [Connective Tissue Diseases](https://www.ncbi.nlm.nih.gov/mesh/68003240)  Mixed Connective Tissue Disease |
| Intervention | [Immunosuppressive Agents - MeSH - NCBI “nih.gov)](https://www.ncbi.nlm.nih.gov/mesh/68007166)  Agents that suppress immune function by one of several mechanisms of action. Classical cytotoxic immunosuppressants act by inhibiting DNA synthesis. Others may act through activation of T-CELLS or by inhibiting the activation of HELPER CELLS. While immunosuppression has been brought about in the past primarily to prevent rejection of transplanted organs, new applications involving mediation of the effects of INTERLEUKINS and other CYTOKINES are emerging. | - Immunosuppressants - Immunosuppressive Agent - Immunosuppressant | "Immunosuppressive Agents" [Mesh] OR "Immunosuppressive Agents" OR "Immunosuppressants" OR "Immunosuppressive Agent" OR "Immunosuppressant"  [Immunologic Factors](https://www.ncbi.nlm.nih.gov/mesh/68007155)  Immunosuppressive Agents  [Complement Inactivating Agents](https://www.ncbi.nlm.nih.gov/mesh/68051056)  [Bradykinin B2 Receptor Antagonists](https://www.ncbi.nlm.nih.gov/mesh/68065094)  [Interleukin-6 Inhibitors](https://www.ncbi.nlm.nih.gov/mesh/2103223)  [Interleukin Inhibitors](https://www.ncbi.nlm.nih.gov/mesh/2101109)  [MTOR Inhibitors](https://www.ncbi.nlm.nih.gov/mesh/2101251)  [Myeloablative Agonists](https://www.ncbi.nlm.nih.gov/mesh/68019653)  [Sphingosine 1 Phosphate Receptor Modulators](https://www.ncbi.nlm.nih.gov/mesh/2031094) |
|  | [Azathioprine - MeSH - NCBI “nih.gov)](https://www.ncbi.nlm.nih.gov/mesh/?term=azathioprine)  An immunosuppressive agent used in combination with cyclophosphamide and hydroxychloroquine in the treatment of rheumatoid arthritis. According to the Fourth Annual Report on Carcinogens “NTP 85-002, 1985), this substance has been listed as a known carcinogen. “Merck Index, 11th ed) | - Azothioprine - Imurel - Imuran - Immuran - Azathioprine Sodium - Azathioprine Sodium Salt - Azathioprine Sulfate | "Azathioprine" [Mesh] OR "Azathioprine" OR "Azothioprine" OR "Imurel" OR "Imuran" OR "Immuran" OR "Azathioprine Sodium" OR "Azathioprine Sodium Salt" OR "Azathioprine Sulfate"  [Nucleic Acids, Nucleotides, and Nucleosides](https://www.ncbi.nlm.nih.gov/mesh/68009706)  [Nucleosides](https://www.ncbi.nlm.nih.gov/mesh/68009705)  [Thionucleosides](https://www.ncbi.nlm.nih.gov/mesh/68013872)  Azathioprine  [Heterocyclic Compounds, 2-Ring](https://www.ncbi.nlm.nih.gov/mesh/68006574)  [Purines](https://www.ncbi.nlm.nih.gov/mesh/68011687)  [Mercaptopurine](https://www.ncbi.nlm.nih.gov/mesh/68015122)  Azathioprine  [Organic Chemicals](https://www.ncbi.nlm.nih.gov/mesh/68009930)  [Sulfur Compounds](https://www.ncbi.nlm.nih.gov/mesh/68013457)  [Thionucleosides](https://www.ncbi.nlm.nih.gov/mesh/68013872)  Azathioprine |
|  | [Cyclophosphamide - MeSH - NCBI “nih.gov)](https://www.ncbi.nlm.nih.gov/mesh/68003520)    Precursor of an alkylating nitrogen mustard antineoplastic and immunosuppressive agent that must be activated in the LIVER to form the active aldophosphamide. It has been used in the treatment of LYMPHOMA and LEUKEMIA. Its side effect, ALOPECIA, has been used for defleecing sheep. Cyclophosphamide may also cause sterility, birth defects, mutations, and cancer. | - Sendoxan - B 518 - B518 - Cyclophosphamide Anhydrous - Cyclophosphamide, “R)-Isomer - Cyclophosphamide, “S)-Isomer - Cytophosphane - Cyclophosphamide Monohydrate - Cytophosphan - Cytoxan - Endoxan - Neosar - NSC 26271 - NSC26271 - Procytox - “+,-)-2-”bis”2-Chloroethyl)amino)tetrahydro-2H-1,3,2-oxazaphosphorine 2-Oxide Monohydrate - Cyclophosphane | "Cyclophosphamide" [Mesh] OR "Cyclophosphamide" OR  "Sendoxan" OR "B 518" OR "B518" OR "Cyclophosphamide Anhydrous" OR "Cyclophosphamide, “R)-Isomer" OR "Cyclophosphamide, “S)-Isomer" OR "Cytophosphane" OR "Cyclophosphamide Monohydrate" OR "Cytophosphan" OR "Cytoxan" OR "Endoxan" OR "Neosar" OR "NSC 26271" OR "NSC26271" OR "Procytox" OR "“+,-)-2-”bis”2-Chloroethyl)amino)tetrahydro-2H-1,3,2-oxazaphosphorine 2-Oxide Monohydrate" OR "Cyclophosphane"  [Hydrocarbons](https://www.ncbi.nlm.nih.gov/mesh/68006838)  [Hydrocarbons, Halogenated](https://www.ncbi.nlm.nih.gov/mesh/68006846)  [Mustard Compounds](https://www.ncbi.nlm.nih.gov/mesh/68009150)  [Nitrogen Mustard Compounds](https://www.ncbi.nlm.nih.gov/mesh/68009588)  [Phosphoramide Mustards](https://www.ncbi.nlm.nih.gov/mesh/68010752)  Cyclophosphamide  [Organic Chemicals](https://www.ncbi.nlm.nih.gov/mesh/68009930)  [Organophosphorus Compounds](https://www.ncbi.nlm.nih.gov/mesh/68009943)  [Phosphoramides](https://www.ncbi.nlm.nih.gov/mesh/68063088)  [Phosphoramide Mustards](https://www.ncbi.nlm.nih.gov/mesh/68010752)  Cyclophosphamide |
|  | [Cyclosporine - MeSH - NCBI “nih.gov)](https://www.ncbi.nlm.nih.gov/mesh/68016572)  A cyclic undecapeptide from an extract of soil fungi. It is a powerful immunosupressant with a specific action on T-lymphocytes. It is used for the prophylaxis of graft rejection in organ and tissue transplantation. “From Martindale, The Extra Pharmacopoeia, 30th ed). | - Cyclosporine A - Cyclosporin A - Ciclosporin - Cyclosporin - Neoral - Sandimmun Neoral - CyA NOF - Sandimmune - Sandimmun - CsA Neoral - CsANeoral - OL 27 400 - OL 27400 | "Cyclosporine" [Mesh] OR "Cyclosporine" OR "Cyclosporine A" OR "Cyclosporin A" OR "Ciclosporin" OR "Cyclosporin" OR "Neoral" OR "Sandimmun Neoral" OR "CyA NOF" OR "Sandimmune" OR "Sandimmun" OR "CsA Neoral" OR "CsANeoral" OR "OL 27 400" OR "OL 27400"  [Chemicals and Drugs Category](https://www.ncbi.nlm.nih.gov/mesh/1000068)  [Polycyclic Compounds](https://www.ncbi.nlm.nih.gov/mesh/68011083)  [Macrocyclic Compounds](https://www.ncbi.nlm.nih.gov/mesh/68047028)  [Peptides, Cyclic](https://www.ncbi.nlm.nih.gov/mesh/68010456)  [Cyclosporins](https://www.ncbi.nlm.nih.gov/mesh/68003524)  Cyclosporine |
|  | [Tacrolimus - MeSH - NCBI “nih.gov)](https://www.ncbi.nlm.nih.gov/mesh/68016559)  A macrolide isolated from the culture broth of a strain of Streptomyces tsukubaensis that has strong immunosuppressive activity in vivo and prevents the activation of T-lymphocytes in response to antigenic or mitogenic stimulation in vitro. | - Prograf - Prograft - FR 900506 - FR900506 - Anhydrous Tacrolimus - Tacrolimus Anhydrous - FK 506 - FK506 | "Tacrolimus" [Mesh] OR "Tacrolimus" OR " Prograf" OR "Prograft" OR "FR 900506" OR "FR900506" OR "Anhydrous Tacrolimus" OR "Tacrolimus Anhydrous" OR "FK 506" OR "FK506"  [Lactones](https://www.ncbi.nlm.nih.gov/mesh/68007783)  [Macrolides](https://www.ncbi.nlm.nih.gov/mesh/68018942)  Tacrolimus |
|  | [Mycophenolic Acid - MeSH - NCBI “nih.gov)](https://www.ncbi.nlm.nih.gov/mesh/68009173)  Compound derived from Penicillium stoloniferum and related species. It blocks de novo biosynthesis of purine nucleotides by inhibition of the enzyme inosine monophosphate dehydrogenase “IMP DEHYDROGENASE). Mycophenolic acid exerts selective effects on the immune system in which it prevents the proliferation of T-CELLS, LYMPHOCYTES, and the formation of antibodies from B-CELLS. It may also inhibit recruitment of LEUKOCYTES to sites of INFLAMMATION. | - Mycophenolate Mofetil - Mycophenolic Acid Morpholinoethyl Ester - Cellcept - Mycophenolate Sodium - Sodium Mycophenolate - Mycophenolate Mofetil Hydrochloride   RS 61443   - RS61443 | "Mycophenolic Acid" [Mesh] OR "Mycophenolic Acid" OR " Mycophenolate Mofetil" OR "Mycophenolic Acid Morpholinoethyl Ester" OR "Cellcept" OR "Mycophenolate Sodium" OR "Sodium Mycophenolate" OR "Mycophenolate Mofetil Hydrochloride" OR "RS 61443" OR "RS61443"  [Acids, Acyclic](https://www.ncbi.nlm.nih.gov/mesh/68000144)  [Caproates](https://www.ncbi.nlm.nih.gov/mesh/68002208)  Mycophenolic Acid |
| Outcomes | [Vaccines, Inactivated - MeSH - NCBI “nih.gov)](https://www.ncbi.nlm.nih.gov/mesh/68015164)  Definition: Vaccines in which the infectious microbial nucleic acid components have been destroyed by chemical or physical treatment “e.g., formalin, beta-propiolactone, gamma radiation) without affecting the antigenicity or immunogenicity of the viral coat or bacterial outer membrane proteins. | Inactivated Vaccines  Killed Vaccine  Killed Vaccines  Inactivated Vaccine | "[Vaccines, Inactivated](https://www.ncbi.nlm.nih.gov/mesh/68015164)" [Mesh] OR "[Vaccines, Inactivated](https://www.ncbi.nlm.nih.gov/mesh/68015164)" OR "Inactivated Vaccines" OR "Killed Vaccine" OR "Killed Vaccines" OR "Inactivated Vaccine"  Vaccines, Inactivated  [Poliovirus Vaccine, Inactivated](https://www.ncbi.nlm.nih.gov/mesh/68011054) |
|  | [Influenza Vaccines - MeSH - NCBI “nih.gov)](https://www.ncbi.nlm.nih.gov/mesh/68007252)  Vaccines used to prevent infection by viruses in the family ORTHOMYXOVIRIDAE. It includes both killed and attenuated vaccines. The composition of the vaccines is changed each year in response to antigenic shifts and changes in prevalence of influenza virus strains. The flu vaccines may be mono- or multi-valent, which contains one or more INFLUENZAVIRUS A and INFLUENZAVIRUS B strains. | - Influenza Vaccine - Influenzavirus Vaccines - Influenza Virus Vaccines - Influenzavirus Vaccine - Influenza Virus Vaccine - High-Dose Trivalent Influenza Vaccine - High Dose Trivalent Influenza Vaccine - Trivalent Influenza Vaccine - Quadrivalent Influenza Vaccine - High dose | "[Influenza Vaccines](https://www.ncbi.nlm.nih.gov/mesh/68007252)" [Mesh:NoExp]  OR Influenza Vaccine" OR "Influenzavirus Vaccines" OR "Influenza Virus Vaccines" OR "Influenzavirus Vaccine" OR "Influenza Virus Vaccine" OR "High-Dose Trivalent Influenza Vaccine" OR "High Dose Trivalent Influenza Vaccine" OR "Trivalent Influenza Vaccine" OR "Quadrivalent Influenza Vaccine"  “não utilizar [Mesh] pois a árvore do termo inclui VVA e intra-nasal)  [Vaccines](https://www.ncbi.nlm.nih.gov/mesh/68014612)  [Viral Vaccines](https://www.ncbi.nlm.nih.gov/mesh/68014765)  Influenza Vaccines |
|  | [Pneumococcal Vaccines - MeSH - NCBI “nih.gov)](https://www.ncbi.nlm.nih.gov/mesh/68022242)  Vaccines or candidate vaccines used to prevent infections with STREPTOCOCCUS PNEUMONIAE. | - Pneumococcal Vaccine - Pneumovax - Pneumococcal Polysaccharide Vaccine - Pnu Imune Vaccine - PnuImune Vaccine - Conjugated penumococcal vaccine | "[Pneumococcal Vaccines](https://www.ncbi.nlm.nih.gov/mesh/68022242)" [Mesh] OR "[Pneumococcal Vaccines](https://www.ncbi.nlm.nih.gov/mesh/68022242)" OR "Pneumococcal Vaccine" OR "Pneumovax" OR "Pneumococcal Polysaccharide Vaccine" OR "Pnu Imune Vaccine" OR "PnuImune Vaccine" OR "Conjugated penumococcal vaccine"  Pneumococcal Vaccines  [Heptavalent Pneumococcal Conjugate Vaccine](https://www.ncbi.nlm.nih.gov/mesh/2009980)  13 pneumococcal conjugate vaccine |
|  | [Herpes Zoster Vaccine - MeSH - NCBI “nih.gov)](https://www.ncbi.nlm.nih.gov/mesh/68053061)  An attenuated vaccine used to prevent and/or treat HERPES ZOSTER, a disease caused by HUMAN HERPESVIRUS 3. | - Zoster Vaccine - Shingles Vaccine | "Herpes Zoster Vaccine" [Mesh:NoExp]  OR " Zoster Vaccine" OR "Shingles Vaccine"  “não utilizar [Mesh] pois a árvore do termo inclui live attenuated zoster  e intra-nasal)  [Vaccines](https://www.ncbi.nlm.nih.gov/mesh/68014612)  [Viral Vaccines](https://www.ncbi.nlm.nih.gov/mesh/68014765)  [Herpesvirus Vaccines](https://www.ncbi.nlm.nih.gov/mesh/68022283)  Herpes Zoster Vaccine |
|  | [Hepatitis B Vaccines - MeSH - NCBI “nih.gov)](https://www.ncbi.nlm.nih.gov/mesh/68017325)  Vaccines or candidate vaccines containing inactivated hepatitis B or some of its component antigens and designed to prevent hepatitis B. Some vaccines may be recombinantly produced. | - Hepatitis B Vaccine | "Hepatitis B Vaccines" [Mesh] OR "Hepatitis B Vaccines" OR "Hepatitis B Vaccine"  [Vaccines](https://www.ncbi.nlm.nih.gov/mesh/68014612)  [Viral Vaccines](https://www.ncbi.nlm.nih.gov/mesh/68014765)  [Viral Hepatitis Vaccines](https://www.ncbi.nlm.nih.gov/mesh/68014761)  Hepatitis B Vaccines |
|  | [Papillomavirus Vaccines - MeSH - NCBI “nih.gov)](https://www.ncbi.nlm.nih.gov/mesh/?term=papillomavirus+vaccines)   Vaccines or candidate vaccines used to prevent PAPILLOMAVIRUS INFECTIONS. Human vaccines are intended to reduce the incidence of UTERINE CERVICAL NEOPLASMS, so they are sometimes considered a type of CANCER VACCINES. They are often composed of CAPSID PROTEINS, especially L1 protein, from various types of ALPHAPAPILLOMAVIRUS | - Papillomavirus Vaccine   Human Papillomavirus Vaccines   - HPV Vaccine   Human Papilloma Virus Vaccine   - HPV Vaccines - Human Papilloma Virus Vaccines - Human Papillomavirus Vaccine | "Papillomavirus Vaccines" [Mesh] OR "Papillomavirus Vaccines" OR "Papillomavirus Vaccine" OR "Human Papillomavirus Vaccines" OR "HPV Vaccine" OR "Human Papilloma Virus Vaccine" OR "HPV Vaccines" OR "Human Papilloma Virus Vaccines" OR "Human Papillomavirus Vaccine"  [Vaccines](https://www.ncbi.nlm.nih.gov/mesh/68014612)  [Viral Vaccines](https://www.ncbi.nlm.nih.gov/mesh/68014765)  Papillomavirus Vaccines  [Human Papillomavirus](https://www.ncbi.nlm.nih.gov/mesh/2009889) |
|  | [Poliovirus Vaccines - MeSH - NCBI “nih.gov)](https://www.ncbi.nlm.nih.gov/mesh/68023321)    Vaccines used to prevent POLIOMYELITIS. They include inactivated “POLIOVIRUS VACCINE, INACTIVATED) | - Poliovirus Vaccine - salk | "Poliovirus Vaccines" [Mesh] OR "Poliovirus Vaccines" OR "Poliovirus Vaccine" OR "salk"  [Vaccines](https://www.ncbi.nlm.nih.gov/mesh/68014612)  [Viral Vaccines](https://www.ncbi.nlm.nih.gov/mesh/68014765)  [Poliovirus Vaccine, Inactivated](https://www.ncbi.nlm.nih.gov/mesh/68011054) |
|  | [Hepatitis A Vaccines - MeSH - NCBI “nih.gov)](https://www.ncbi.nlm.nih.gov/mesh/68022362)    Vaccines or candidate vaccines used to prevent infection with hepatitis A virus “HEPATOVIRUS). | - Hepatovirus Vaccines - Hepatitis A Vaccine - Vaqta - Havrix | "Hepatitis A Vaccines" [Mesh] OR "Hepatitis A Vaccines" OR "Hepatovirus Vaccines" OR "Hepatitis A Vaccine" OR "Vaqta" OR "Havrix"  [Vaccines](https://www.ncbi.nlm.nih.gov/mesh/68014612)  [Viral Hepatitis Vaccines](https://www.ncbi.nlm.nih.gov/mesh/68014761)  Hepatitis A Vaccines |
|  | [Meningococcal Vaccines - MeSH - NCBI “nih.gov)](https://www.ncbi.nlm.nih.gov/mesh/68022401)    Vaccines or candidate vaccines used to prevent infection with NEISSERIA MENINGITIDIS. | Meningococcal Vaccine  Menactra  Tetravalent Meningococcal Vaccine  Menomune  Menveo  Bivalent Meningococcal Vaccine  Meningococcal Polysaccharide Vaccine | "Meningococcal Vaccines" [Mesh] OR " Meningococcal Vaccine" OR "Menactra" OR "Tetravalent Meningococcal Vaccine" OR "Menomune OR "Menveo" OR "Bivalent Meningococcal Vaccine" OR "Meningococcal Polysaccharide Vaccine"    [Vaccines](https://www.ncbi.nlm.nih.gov/mesh/68014612)  Meningococcal Vaccines C, B or ACWY |
|  | [Diphtheria-Tetanus-Pertussis Vaccine - MeSH - NCBI “nih.gov)](https://www.ncbi.nlm.nih.gov/mesh/?term=vaccine+per)   A vaccine consisting of DIPHTHERIA TOXOID; TETANUS TOXOID; and whole-cell PERTUSSIS VACCINE. The vaccine protects against diphtheria, tetanus, and whooping cough. | Diphtheria Tetanus Pertussis Vaccine  DTwP Vaccine  DPT Vaccine  DTP Vaccine  Di Te Per Vaccine  Diphtheria Pertussis Tetanus Vaccine | "Diphtheria-Tetanus-Pertussis Vaccine" [Mesh] OR "Diphtheria-Tetanus-Pertussis Vaccine" OR " Diphtheria Tetanus Pertussis Vaccine" OR "DTwP Vaccine" OR "DPT Vaccine" OR "DTP Vaccine" OR "Di Te Per Vaccine" OR "Diphtheria Pertussis Tetanus Vaccine"  [Vaccines](https://www.ncbi.nlm.nih.gov/mesh/68014612)  [Pertussis Vaccine](https://www.ncbi.nlm.nih.gov/mesh/68010567)  Diphtheria-Tetanus-Pertussis Vaccine |
|  | [Haemophilus influenza type b polysaccharide vaccine-tetanus toxin conj - MeSH - NCBI “nih.gov)](https://www.ncbi.nlm.nih.gov/mesh/?term=haemophilus+influenza+type+b+polysaccharide+vaccine+tetanus+toxin+conjugate)    the type b capsular polysaccharide of HIB-vaccine is conjugated to tetanus toxoid | HIBest  PRP-T  Hib-TT  Hib capsular polysaccharide-tetanus vaccine  Hib CPS-tetanus vaccine  OmniHIB  ActHIB | "Haemophilus influenza type b polysaccharide vaccine-tetanus toxin conjugate" OR "HIBest" OR "PRP-T" OR "Hib-TT" OR "Hib capsular polysaccharide-tetanus vaccine" OR "Hib CPS-tetanus vaccine" OR "OmniHIB" OR "ActHIB" |

**Question 05.** What effect of DMARDsb use (except RTX) have on the on the response to inactivated vaccine in patients with immune-mediated rheumatic diseases?

| Group | **MeSH Terms**  and definitions | **Entry Terms** | **Search Key** |
| --- | --- | --- | --- |
| Population  Patients with immune-mediated rheumatic diseases “IMRD) | [Rheumatic Diseases - MeSH - NCBI “nih.gov)](https://www.ncbi.nlm.nih.gov/mesh/68012216)  Disorders of connective tissue, especially the joints and related structures, characterized by inflammation, degeneration, or metabolic derangement. | Rheumatic Disease  Rheumatism | "[Rheumatic Diseases](https://www.ncbi.nlm.nih.gov/mesh/68012216)" [Mesh:NoExp]  OR "Rheumatic Disease" OR "Rheumatism" OR  "Immune-mediated rheumatic diseases" |
|  | [Arthritis, Rheumatoid - MeSH - NCBI “nih.gov)](https://www.ncbi.nlm.nih.gov/mesh/68001172)  A chronic systemic disease, primarily of the joints, marked by inflammatory changes in the synovial membranes and articular structures, widespread fibrinoid degeneration of the collagen fibers in mesenchymal tissues, and by atrophy and rarefaction of bony structures. Etiology is unknown, but autoimmune mechanisms have been implicated. | - Rheumatoid Arthritis | "[Arthritis, Rheumatoid](https://www.ncbi.nlm.nih.gov/mesh/68001172)" [Mesh:NoExp]  OR "Rheumatoid Arthritis" OR "[Rheumatoid Nodule](https://www.ncbi.nlm.nih.gov/mesh/68012218)" OR "[Rheumatoid Vasculitis](https://www.ncbi.nlm.nih.gov/mesh/68056653)" OR "[Sjogren's Syndrome](https://www.ncbi.nlm.nih.gov/mesh/68012859)"  “não utilizar [Mesh] pois a árvore do termo inclui caplan syndrome, felty syndrome) |
|  | [Spondylarthritis - MeSH - NCBI “nih.gov)](https://www.ncbi.nlm.nih.gov/mesh/68025241)  Inflammation of the joints of the SPINE, the intervertebral articulations. | - Spondylarthritides - Spinal Arthritis - Spinal Arthritides | "[Spondylarthritis](https://www.ncbi.nlm.nih.gov/mesh/68025241)" [Mesh] OR "[Spondylarthritis](https://www.ncbi.nlm.nih.gov/mesh/68025241)" OR "Spondylarthritides" OR "Spinal Arthritis" OR "Spinal Arthritides"  [Spondylitis](https://www.ncbi.nlm.nih.gov/mesh/68013166)  Spondylarthritis  [Osteoarthritis, Spine](https://www.ncbi.nlm.nih.gov/mesh/68055013)  [Spondylarthropathies](https://www.ncbi.nlm.nih.gov/mesh/68025242)  [Arthritis, Psoriatic](https://www.ncbi.nlm.nih.gov/mesh/68015535)  [Arthritis, Reactive](https://www.ncbi.nlm.nih.gov/mesh/68016918)  [Axial Spondyloarthritis](https://www.ncbi.nlm.nih.gov/mesh/2101123) + |
|  | [Arthritis, Psoriatic - MeSH - NCBI “nih.gov)](https://www.ncbi.nlm.nih.gov/mesh/68015535)  A type of inflammatory arthritis associated with PSORIASIS, often involving the axial joints and the peripheral terminal interphalangeal joints. It is characterized by the presence of HLA-B27-associated SPONDYLARTHROPATHY, and the absence of rheumatoid factor. | - Arthritic Psoriasis - Psoriatic Arthritis - Psoriasis Arthropathica - Psoriatic Arthropathy - Psoriatic Arthropathies | "[Arthritis, Psoriatic](https://www.ncbi.nlm.nih.gov/mesh/68015535)" [Mesh] OR "[Arthritis, Psoriatic](https://www.ncbi.nlm.nih.gov/mesh/68015535)" OR "Arthritic Psoriasis" OR "Psoriatic Arthritis" OR "Psoriasis Arthropathica" OR "Psoriatic Arthropathy" OR "Psoriatic Arthropathies"  [Spondylarthritis](https://www.ncbi.nlm.nih.gov/mesh/68025241)  [Spondylarthropathies](https://www.ncbi.nlm.nih.gov/mesh/68025242)  Arthritis, Psoriatic |
|  | [Spondylitis, Ankylosing - MeSH - NCBI “nih.gov)](https://www.ncbi.nlm.nih.gov/mesh/?term=ankylosing+spondylitis)  A chronic inflammatory condition affecting the axial joints, such as the SACROILIAC JOINT and other intervertebral or costovertebral joints. It occurs predominantly in young males and is characterized by pain and stiffness of joints “ANKYLOSIS) with inflammation at tendon insertions. | - Bechterew's Disease - Bechterews Disease - Marie Struempell Disease - Spondylarthritis Ankylopoietica - Spondyloarthritis Ankylopoietica - Ankylosing Spondylitis - Ankylosing Spondylarthritis - Ankylosing Spondylarthritides - Ankylosing Spondyloarthritis - Ankylosing Spondyloarthritides - Spondylitis Ankylopoietica - Bechterew Disease - Rheumatoid Spondylitis | "[Spondylitis, Ankylosing](https://www.ncbi.nlm.nih.gov/mesh/?term=ankylosing+spondylitis)" [Mesh] OR "[Spondylitis, Ankylosing](https://www.ncbi.nlm.nih.gov/mesh/?term=ankylosing+spondylitis)" OR "Bechterew's Disease" OR "Bechterews Disease" OR "Marie Struempell Disease" OR "Spondylarthritis Ankylopoietica" OR "Spondyloarthritis Ankylopoietica" OR "Ankylosing Spondylitis" OR "Ankylosing Spondylarthritis" OR "Ankylosing Spondylarthritides" OR "Ankylosing Spondyloarthritis" OR "Ankylosing Spondyloarthritides" OR "Spondylitis Ankylopoietica" OR "Bechterew Disease" OR "Rheumatoid Spondylitis"  [Spondylarthritis](https://www.ncbi.nlm.nih.gov/mesh/68025241)  [Spondylarthropathies](https://www.ncbi.nlm.nih.gov/mesh/68025242)  [Axial Spondyloarthritis](https://www.ncbi.nlm.nih.gov/mesh/2101123)  Spondylitis, Ankylosing |
|  | [Lupus Erythematosus, Systemic - MeSH - NCBI “nih.gov)](https://www.ncbi.nlm.nih.gov/mesh/68008180)  A chronic, relapsing, inflammatory, and often febrile multisystemic disorder of connective tissue, characterized principally by involvement of the skin, joints, kidneys, and serosal membranes. It is of unknown etiology, but is thought to represent a failure of the regulatory mechanisms of the autoimmune system. The disease is marked by a wide range of system dysfunctions, an elevated erythrocyte sedimentation rate, and the formation of LE cells in the blood or bone marrow. | - Systemic Lupus Erythematosus - Lupus Erythematosus Disseminatus - Libman Sacks Disease | "[Lupus Erythematosus, Systemic](https://www.ncbi.nlm.nih.gov/mesh/68008180)" [Mesh] OR "[Lupus Erythematosus, Systemic](https://www.ncbi.nlm.nih.gov/mesh/68008180)" OR "Systemic Lupus Erythematosus " OR "Lupus Erythematosus Disseminatus" OR "Libman Sacks Disease"  [Connective Tissue Diseases](https://www.ncbi.nlm.nih.gov/mesh/68003240)  Lupus Erythematosus, Systemic  [Lupus Nephritis](https://www.ncbi.nlm.nih.gov/mesh/68008181)  [Lupus Vasculitis, Central Nervous System](https://www.ncbi.nlm.nih.gov/mesh/68020945) |
|  | [Myositis - MeSH - NCBI “nih.gov)](https://www.ncbi.nlm.nih.gov/mesh/?term=inflammatory+myopathies)  Inflammation of a muscle or muscle tissue. | - Myositides - Inflammatory Muscle Diseases - Inflammatory Muscle Disease - Inflammatory Myopathy - Inflammatory Myopathies - Proliferative Myositides - Proliferative Myositis - Infectious Myositides - Infectious Myositis - Idiopathic Inflammatory Myopathies - Idiopathic Inflammatory Myopathy - Idiopathic Inflammatory Myositis - Focal Myositides - Focal Myositis | "[Myositis](https://www.ncbi.nlm.nih.gov/mesh/?term=inflammatory+myopathies)" [Mesh] OR "[Myositis](https://www.ncbi.nlm.nih.gov/mesh/?term=inflammatory+myopathies)" OR "Myositides" OR "Inflammatory Muscle Diseases" OR "Inflammatory Muscle Disease" OR "Inflammatory Myopathy" OR "Inflammatory Myopathies" OR "Proliferative Myositides" OR "Proliferative Myositis" OR "Infectious Myositides" OR "Infectious Myositis" OR "Idiopathic Inflammatory Myopathies" OR "Idiopathic Inflammatory Myopathy" OR "Idiopathic Inflammatory Myositis" OR "Focal Myositides" OR "Focal Myositis"  [Muscular Diseases](https://www.ncbi.nlm.nih.gov/mesh/68009135)  Myositis  [Myositis Ossificans](https://www.ncbi.nlm.nih.gov/mesh/68009221)  [Myositis, Inclusion Body](https://www.ncbi.nlm.nih.gov/mesh/68018979)  [Orbital Myositis](https://www.ncbi.nlm.nih.gov/mesh/68055622)  [Polymyositis](https://www.ncbi.nlm.nih.gov/mesh/68017285)  [Dermatomyositis](https://www.ncbi.nlm.nih.gov/mesh/68003882)  [Pyomyositis](https://www.ncbi.nlm.nih.gov/mesh/68052880) |
|  | [Systemic Vasculitis - MeSH - NCBI “nih.gov)](https://www.ncbi.nlm.nih.gov/mesh/?term=systemic+vasculitides)  A heterogeneous group of diseases characterized by inflammation and necrosis of the blood vessel walls. | - Systemic Vasculitides | "[Systemic Vasculitis](https://www.ncbi.nlm.nih.gov/mesh/?term=systemic+vasculitides)" [Mesh] OR "[Systemic Vasculitis](https://www.ncbi.nlm.nih.gov/mesh/?term=systemic+vasculitides)" OR "Systemic Vasculitides"  [Vasculitis](https://www.ncbi.nlm.nih.gov/mesh/68014657)  Systemic Vasculitis  [Anti-Neutrophil Cytoplasmic Antibody-Associated Vasculitis](https://www.ncbi.nlm.nih.gov/mesh/68056648)  [Churg-Strauss Syndrome](https://www.ncbi.nlm.nih.gov/mesh/68015267)  [Granulomatosis with Polyangiitis](https://www.ncbi.nlm.nih.gov/mesh/68014890)  [Microscopic Polyangiitis](https://www.ncbi.nlm.nih.gov/mesh/68055953)  [Polyarteritis Nodosa](https://www.ncbi.nlm.nih.gov/mesh/68010488)  [Rheumatoid Vasculitis](https://www.ncbi.nlm.nih.gov/mesh/68056653) |
|  | [Sjogren's Syndrome - MeSH - NCBI “nih.gov)](https://www.ncbi.nlm.nih.gov/mesh/68012859)  Chronic inflammatory and autoimmune disease in which the salivary and lacrimal glands undergo progressive destruction by lymphocytes and plasma cells resulting in decreased production of saliva and tears. The primary form, often called sicca syndrome, involves both KERATOCONJUNCTIVITIS SICCA and XEROSTOMIA. The secondary form includes, in addition, the presence of a connective tissue disease, usually rheumatoid arthritis. | - Sjogrens Syndrome - Sjogren Syndrome - Sicca Syndrome | "[Sjogren's Syndrome](https://www.ncbi.nlm.nih.gov/mesh/68012859)" [Mesh] OR "[Sjogren's Syndrome](https://www.ncbi.nlm.nih.gov/mesh/68012859)"  OR "Sjogrens Syndrome" OR "Sjogren Syndrome" OR "Sicca Syndrome"  [Arthritis](https://www.ncbi.nlm.nih.gov/mesh/68001168)  [Arthritis, Rheumatoid](https://www.ncbi.nlm.nih.gov/mesh/68001172)  Sjogren's Syndrome |
|  | [Scleroderma, Systemic - MeSH - NCBI “nih.gov)](https://www.ncbi.nlm.nih.gov/mesh/68012595)  A chronic multi-system disorder of CONNECTIVE TISSUE. It is characterized by SCLEROSIS in the SKIN, the LUNGS, the HEART, the GASTROINTESTINAL TRACT, the KIDNEYS, and the MUSCULOSKELETAL SYSTEM. Other important features include diseased small BLOOD VESSELS and AUTOANTIBODIES. The disorder is named for its most prominent feature “hard skin), and classified into subsets by the extent of skin thickening: LIMITED SCLERODERMA and DIFFUSE SCLERODERMA. | - Systemic Sclerosis - Systemic Scleroderma | "[Scleroderma, Systemic](https://www.ncbi.nlm.nih.gov/mesh/68012595)" [Mesh] OR "[Scleroderma, Systemic](https://www.ncbi.nlm.nih.gov/mesh/68012595)" OR "Systemic Sclerosis" OR "Systemic Scleroderma"  [Connective Tissue Diseases](https://www.ncbi.nlm.nih.gov/mesh/68003240)  Scleroderma, Systemic  [Scleroderma, Diffuse](https://www.ncbi.nlm.nih.gov/mesh/68045743)  [Scleroderma, Limited](https://www.ncbi.nlm.nih.gov/mesh/68045745)  [CREST Syndrome](https://www.ncbi.nlm.nih.gov/mesh/68017675) |
|  | [Mixed Connective Tissue Disease - MeSH - NCBI “nih.gov)](https://www.ncbi.nlm.nih.gov/mesh/?term=Mixed+connective+tissue+disease)  A syndrome with overlapping clinical features of systemic lupus erythematosus, scleroderma, polymyositis, and Raynaud's phenomenon. The disease is differentially characterized by high serum titers of antibodies to ribonuclease-sensitive extractable “saline soluble) nuclear antigen and a "speckled" epidermal nuclear staining pattern on direct immunofluorescence. | - Sharp Syndrome - MCTD | "[Mixed Connective Tissue Disease](https://www.ncbi.nlm.nih.gov/mesh/?term=Mixed+connective+tissue+disease)" [Mesh] OR "[Mixed Connective Tissue Disease](https://www.ncbi.nlm.nih.gov/mesh/?term=Mixed+connective+tissue+disease)" OR "Sharp Syndrome" OR "MCTD"  [Connective Tissue Diseases](https://www.ncbi.nlm.nih.gov/mesh/68003240)  Mixed Connective Tissue Disease |
| Intervention  DMARDsb use “except RTX) | [Antibodies, Monoclonal - MeSH - NCBI “nih.gov)](https://www.ncbi.nlm.nih.gov/mesh?Db=mesh&Term=%22ANTIBODIES,%20MONOCLONAL%20%22%5BMESH%5D)  Antibodies produced by a single clone of cells. | - Monoclonal Antibodies - Monoclonal Antibody | "Antibodies, Monoclonal" [Mesh] OR "Antibodies, Monoclonal" OR "Monoclonal Antibodies" OR "Monoclonal Antibody"  [Immunoglobulins](https://www.ncbi.nlm.nih.gov/mesh/68007136)  [Antibodies](https://www.ncbi.nlm.nih.gov/mesh/68000906)  Antibodies, Monoclonal  [Antibodies, Monoclonal, Humanized](https://www.ncbi.nlm.nih.gov/mesh/68061067)  [Abciximab](https://www.ncbi.nlm.nih.gov/mesh/2027974)  [Adalimumab](https://www.ncbi.nlm.nih.gov/mesh/2009894)  [Alemtuzumab](https://www.ncbi.nlm.nih.gov/mesh/2023293)  [Basiliximab](https://www.ncbi.nlm.nih.gov/mesh/2028066)  [Bevacizumab](https://www.ncbi.nlm.nih.gov/mesh/2009814)  [Brentuximab Vedotin](https://www.ncbi.nlm.nih.gov/mesh/2030888)  [Certolizumab Pegol](https://www.ncbi.nlm.nih.gov/mesh/2009848)  [Cetuximab](https://www.ncbi.nlm.nih.gov/mesh/2009883)  [Daclizumab](https://www.ncbi.nlm.nih.gov/mesh/2028075)  [Denosumab](https://www.ncbi.nlm.nih.gov/mesh/2009985)  [Gemtuzumab](https://www.ncbi.nlm.nih.gov/mesh/2030889)  [Inotuzumab Ozogamicin](https://www.ncbi.nlm.nih.gov/mesh/2030902)  [Ipilimumab](https://www.ncbi.nlm.nih.gov/mesh/2023294)  [Natalizumab](https://www.ncbi.nlm.nih.gov/mesh/2009979)  [Nivolumab](https://www.ncbi.nlm.nih.gov/mesh/2028090)  [Omalizumab](https://www.ncbi.nlm.nih.gov/mesh/2009981)  [Palivizumab](https://www.ncbi.nlm.nih.gov/mesh/2009992)  [Panitumumab](https://www.ncbi.nlm.nih.gov/mesh/2028058)  [Ranibizumab](https://www.ncbi.nlm.nih.gov/mesh/2010040)  [Trastuzumab](https://www.ncbi.nlm.nih.gov/mesh/2009893) +  [Ustekinumab](https://www.ncbi.nlm.nih.gov/mesh/2010028)  [Antibodies, Monoclonal, Murine-Derived](https://www.ncbi.nlm.nih.gov/mesh/68058846)  [Muromonab-CD3](https://www.ncbi.nlm.nih.gov/mesh/68016853)  [Rituximab](https://www.ncbi.nlm.nih.gov/mesh/2009941)  [Combined Antibody Therapeutics](https://www.ncbi.nlm.nih.gov/mesh/2103308)  [Infliximab](https://www.ncbi.nlm.nih.gov/mesh/2009943)  [Single-Chain Antibodies](https://www.ncbi.nlm.nih.gov/mesh/68057127) |
|  | Anti-CD20 | - | - |
|  | [veltuzumab - MeSH - NCBI “nih.gov)](https://www.ncbi.nlm.nih.gov/mesh/?term=Veltuzumab)  humanized anti-CD20 monoclonal antibody; used in combination with the humanized anti-CD22 antibody, epratuzumab, for the therapy of non-Hodgkin's lymphoma | - HA20 - monoclonal antibody IMMU-106 - IMMU 106 | "veltuzumab" [Mesh] OR "veltuzumab" OR "HA20" OR "monoclonal antibody" OR "IMMU-106" OR "IMMU 106" |
|  | [obinutuzumab - MeSH - NCBI “nih.gov)](https://www.ncbi.nlm.nih.gov/mesh/?term=Obinutuzumab)  anti-CD20 mAb for treating B-cell lymphoid cancers | - RO 5072759 - R 7159 - GA 101 - GA101 antibody   Gazyva   - gazyvaro - afutuzumab | "obinutuzumab" [Mesh] OR "obinutuzumab" OR "RO 5072759" OR "R 7159" OR "GA 101" OR "GA101 antibody" OR "Gazyva" OR "gazyvaro" OR "afutuzumab" |
|  | [Antibodies, Monoclonal, Humanized - MeSH - NCBI “nih.gov)](https://www.ncbi.nlm.nih.gov/mesh/68061067)    Antibodies from non-human species whose protein sequences have been modified to make them nearly identical with human antibodies. If the constant region and part of the variable region are replaced, they are called humanized. If only the constant region is modified they are called chimeric. INN names for humanized antibodies end in -zumab. | - Humanized Antibodies | "Antibodies, Monoclonal, Humanized" [Mesh] OR "Antibodies, Monoclonal, Humanized" OR "Humanized Antibodies"  [Immunoglobulins](https://www.ncbi.nlm.nih.gov/mesh/68007136)  [Antibodies](https://www.ncbi.nlm.nih.gov/mesh/68000906)  [Antibodies, Monoclonal](https://www.ncbi.nlm.nih.gov/mesh/68000911)  Antibodies, Monoclonal, Humanized  [Abciximab](https://www.ncbi.nlm.nih.gov/mesh/2027974)  [Adalimumab](https://www.ncbi.nlm.nih.gov/mesh/2009894)  [Alemtuzumab](https://www.ncbi.nlm.nih.gov/mesh/2023293)  [Basiliximab](https://www.ncbi.nlm.nih.gov/mesh/2028066)  [Bevacizumab](https://www.ncbi.nlm.nih.gov/mesh/2009814)  [Brentuximab Vedotin](https://www.ncbi.nlm.nih.gov/mesh/2030888)  [Certolizumab Pegol](https://www.ncbi.nlm.nih.gov/mesh/2009848)  [Cetuximab](https://www.ncbi.nlm.nih.gov/mesh/2009883)  [Daclizumab](https://www.ncbi.nlm.nih.gov/mesh/2028075)  [Denosumab](https://www.ncbi.nlm.nih.gov/mesh/2009985)  [Gemtuzumab](https://www.ncbi.nlm.nih.gov/mesh/2030889)  [Inotuzumab Ozogamicin](https://www.ncbi.nlm.nih.gov/mesh/2030902)  [Ipilimumab](https://www.ncbi.nlm.nih.gov/mesh/2023294)  [Natalizumab](https://www.ncbi.nlm.nih.gov/mesh/2009979)  [Nivolumab](https://www.ncbi.nlm.nih.gov/mesh/2028090)  [Omalizumab](https://www.ncbi.nlm.nih.gov/mesh/2009981)  [Palivizumab](https://www.ncbi.nlm.nih.gov/mesh/2009992)  [Panitumumab](https://www.ncbi.nlm.nih.gov/mesh/2028058)  [Ranibizumab](https://www.ncbi.nlm.nih.gov/mesh/2010040)  [Trastuzumab](https://www.ncbi.nlm.nih.gov/mesh/2009893)  [Ado-Trastuzumab Emtansine](https://www.ncbi.nlm.nih.gov/mesh/2030901)  [Ustekinumab](https://www.ncbi.nlm.nih.gov/mesh/2010028) |
|  | [ocrelizumab - MeSH - NCBI “nih.gov)](https://www.ncbi.nlm.nih.gov/mesh/?term=Ocrelizumab)  a humanized anti-CD20 antibody | - Ocrevus - R 1594 - PR 070769 | "ocrelizumab" [Mesh] OR "ocrelizumab" OR "Ocrevus" OR "R 1594" OR "PR 070769" |
|  | [ofatumumab - MeSH - NCBI “nih.gov)](https://www.ncbi.nlm.nih.gov/mesh/?term=Ofatumumab)  an anti-CD20 MAb used as an antineoplastic agent; in phase 1-2 clinical trials | - kesimpta - HUMAX-CD20-2F2 - GSKI841157 - arzerra | "ofatumumab" [Mesh] OR "ofatumumab" OR "kesimpta" OR "HUMAX-CD20-2F2" OR "GSKI841157" OR "arzerra" |
|  | [belimumab - MeSH - NCBI “nih.gov)](https://www.ncbi.nlm.nih.gov/mesh/?term=belimumab)  monoclonal antibody directed against human BLyS protein “B lymphocyte stimulator) for treatment of autoimmune diseases | - BEL114333 - HGS1006 - LymphoStat-B - GSK1550188 - Benlysta | "belimumab" [Mesh] OR "belimumab" OR "BEL114333" OR "HGS1006" OR "LymphoStat-B" OR "GSK1550188" OR "Benlysta" |
|  | [rilonacept - MeSH - NCBI “nih.gov)](https://www.ncbi.nlm.nih.gov/mesh/?term=Rilonacept)  an anti-arthritis agent derived from IL-1R and IL-1RAcP | - interleukin-1 Trap | "rilonacept" [Mesh] OR "rilonacept" OR "interleukin-1 Trap" |
|  | [Interleukin-1 - MeSH - NCBI “nih.gov)](https://www.ncbi.nlm.nih.gov/mesh/68007375)  A soluble factor produced by MONOCYTES; MACROPHAGES, and other cells which activates T-lymphocytes and potentiates their response to mitogens or antigens. Interleukin-1 is a general term refers to either of the two distinct proteins, INTERLEUKIN-1ALPHA and INTERLEUKIN-1BETA. The biological effects of IL-1 include the ability to replace macrophage requirements for T-cell activation. | - Interleukin 1 - IL-1 - T Helper Factor - Lymphocyte Activating Factor - Macrophage Cell Factor - Epidermal Cell Derived Thymocyte Activating Factor - Interleukin I | "Interleukin-1" [Mesh] OR "Interleukin-1" OR "Interleukin 1" OR "IL-1" OR "T Helper Factor" OR "Lymphocyte Activating Factor" OR "Macrophage Cell Factor" OR "Epidermal Cell Derived Thymocyte Activating Factor" OR "Interleukin I"  [Cytokines](https://www.ncbi.nlm.nih.gov/mesh/68016207)  [Interleukins](https://www.ncbi.nlm.nih.gov/mesh/68007378)  Interleukin-1  [Interleukin-1alpha](https://www.ncbi.nlm.nih.gov/mesh/68053582)  [Interleukin-1beta](https://www.ncbi.nlm.nih.gov/mesh/68053583) |
|  | c [- MeSH - NCBI “nih.gov)](https://www.ncbi.nlm.nih.gov/mesh/?term=Canakinumab)  a human anti-interleukin-1beta monoclonal antibody for treatment of cryopyrin-associated periodic syndrome | - Ilaris - ACZ885 - immunoglobulin G1, anti-”human interleukin 1beta) “human clone ACZ885 heavy chain V region) | "canakinumab" [Mesh] OR "canakinumab" OR "Ilaris" OR "ACZ885" OR "immunoglobulin G1, anti-”human interleukin 1beta) “human clone ACZ885 heavy chain V region) " |
|  | [Interleukin-6 - MeSH - NCBI “nih.gov)](https://www.ncbi.nlm.nih.gov/mesh/68015850)  A cytokine that stimulates the growth and differentiation of B-LYMPHOCYTES and is also a growth factor for HYBRIDOMAS and plasmacytomas. It is produced by many different cells including T-LYMPHOCYTES; MONOCYTES; and FIBROBLASTS. | - Interleukin 6 - IL6 - B-Cell Stimulatory Factor 2 - B-Cell Stimulatory Factor-2 - B-Cell Differentiation Factor-2 - B Cell Differentiation Factor 2 - BSF-2 - Hybridoma Growth Factor - IFN-beta 2 - Plasmacytoma Growth Factor - Hepatocyte Stimulating Factor - MGI-2 - Myeloid Differentiation-Inducing Protein - Myeloid Differentiation Inducing Protein - B Cell Differentiation Factor - IL-6 - Interferon beta-2 - Interferon beta 2 - B Cell Stimulatory Factor 2 | "Interleukin-6" [Mesh] OR "Interleukin-6" OR "Interleukin 6" OR "IL6" OR "B-Cell Stimulatory Factor 2" OR "B-Cell Stimulatory Factor-2" OR "B-Cell Differentiation Factor-2" OR "B Cell Differentiation Factor 2" OR "BSF-2" OR "Hybridoma Growth Factor" OR "IFN-beta 2" OR "Plasmacytoma Growth Factor" OR "Hepatocyte Stimulating Factor" OR "MGI-2" OR "Myeloid Differentiation-Inducing Protein" OR "Myeloid Differentiation Inducing Protein" OR "B Cell Differentiation Factor" OR "IL-6" OR "Interferon beta-2" OR "Interferon beta 2" OR "B Cell Stimulatory Factor 2"  [Cytokines](https://www.ncbi.nlm.nih.gov/mesh/68016207)  [Interleukins](https://www.ncbi.nlm.nih.gov/mesh/68007378)  Interleukin-6 |
|  | [tocilizumab - MeSH - NCBI “nih.gov)](https://www.ncbi.nlm.nih.gov/mesh/67502936)  a humanised anti-interleukin-6 receptor monoclonal antibody | - atlizumab - monoclonal antibody, MRA - BAT1806 - MSB11456 - RG-1569 - R-1569 - Actemra - Roactemra - RHPM-1 | "tocilizumab" [Mesh] OR "tocilizumab" OR "atlizumab" OR "monoclonal antibody, MRA" OR "BAT1806" OR "MSB11456" OR "RG-1569" OR "R-1569" OR "Actemra" OR "Roactemra" OR "RHPM-1" |
|  | [Tumor Necrosis Factor Inhibitors - MeSH - NCBI “nih.gov)](https://www.ncbi.nlm.nih.gov/mesh/2030852)  Compounds or agents that bind to and inhibit the synthesis or activity of TUMOR NECROSIS FACTOR-alpha. Such agents are used to treat inflammatory bowel diseases and other inflammatory diseases. | - Tumor Necrosis Factor Blockers - TNF Inhibitors - TNF Blockers - Tumor Necrosis Factor Blocker - Tumor Necrosis Factor Antagonist - Tumor Necrosis Factor Inhibitor - TNF Antagonist - TNF Blocker - TNF Inhibitor - Tumor Necrosis Factor Antagonists - TNF Antagonists - Tumor Necrosis Factor-a “TNF-a) Antagonists - Tumor Necrosis Factor-a “TNF-a) Antagonist - Tumor Necrosis Factor-a “TNF-a) Inhibitors - Tumor Necrosis Factor-a “TNF-a) Inhibitor - Tumor Necrosis Factor-a “TNF-a) Blockers - Tumor Necrosis Factor-a “TNF-a) Blocker | "Tumor Necrosis Factor Inhibitors " [Mesh] OR "Tumor Necrosis Factor Inhibitors" OR "Tumor Necrosis Factor Blockers" OR "TNF Inhibitors" OR "TNF Blockers" OR "Tumor Necrosis Factor Blocker" OR "Tumor Necrosis Factor Antagonist" OR "Tumor Necrosis Factor Inhibitor" OR "TNF Antagonist" OR "TNF Blocker" OR "TNF Inhibitor" OR "Tumor Necrosis Factor Antagonists" OR "TNF Antagonists" OR "Tumor Necrosis Factor-a “TNF-a) Antagonists" OR "Tumor Necrosis Factor-a “TNF-a) Antagonist" OR "Tumor Necrosis Factor-a “TNF-a) Inhibitors" OR "Tumor Necrosis Factor-a “TNF-a) Inhibitor" OR "Tumor Necrosis Factor-a “TNF-a) Blockers" OR "Tumor Necrosis Factor-a “TNF-a) Blocker"  [Pharmacologic Actions](https://www.ncbi.nlm.nih.gov/mesh/68020228)  [Therapeutic Uses](https://www.ncbi.nlm.nih.gov/mesh/68045506)  [Anti-Inflammatory Agents](https://www.ncbi.nlm.nih.gov/mesh/68000893)  Tumor Necrosis Factor Inhibitors |
|  | [Etanercept - MeSH - NCBI “nih.gov)](https://www.ncbi.nlm.nih.gov/mesh/2009879)  A recombinant version of soluble human TNF receptor fused to an IgG FC fragment that binds specifically to TUMOR NECROSIS FACTOR and inhibits its binding with endogenous TNF receptors. It prevents the inflammatory effect of TNF and is used to treat RHEUMATOID ARTHRITIS; PSORIATIC ARTHRITIS and ANKYLOSING SPONDYLITIS | - TNFR-Fc Fusion Protein - TNFR Fc Fusion Protein - TNR 001 - TNT Receptor Fusion Protein - TNTR-Fc - TNR-001 - TNR001 - Etanercept-szzs - TNF Receptor Type II-IgG Fusion Protein - TNF Receptor Type II IgG Fusion Protein - Erelzi - Recombinant Human Dimeric TNF Receptor Type II-IgG Fusion Protein - Recombinant Human Dimeric TNF Receptor Type II IgG Fusion Protein - Enbrel | "Etanercept" [Mesh] OR "Etanercept" OR "TNFR-Fc Fusion Protein" OR "TNFR Fc Fusion Protein" OR "TNR 001" OR "TNT Receptor Fusion Protein" OR "TNTR-Fc" OR "TNR-001" OR "TNR001" OR "Etanercept-szzs" OR "TNF Receptor Type II-IgG Fusion Protein" OR "TNF Receptor Type II IgG Fusion Protein" OR "Erelzi" OR "Recombinant Human Dimeric TNF Receptor Type II-IgG Fusion Protein" OR "Recombinant Human Dimeric TNF" OR               "Receptor Type II IgG Fusion Protein" OR "Enbrel"  [Peptides](https://www.ncbi.nlm.nih.gov/mesh/68010455)  [Peptide Fragments](https://www.ncbi.nlm.nih.gov/mesh/68010446)  [Immunoglobulin Fragments](https://www.ncbi.nlm.nih.gov/mesh/68007128)  [Immunoglobulin Fc Fragments](https://www.ncbi.nlm.nih.gov/mesh/68007141)  Etanercept |
|  | [Infliximab - MeSH - NCBI “nih.gov)](https://www.ncbi.nlm.nih.gov/mesh/2009943)  A chimeric monoclonal antibody to TNF-ALPHA that is used in the treatment of RHEUMATOID ARTHRITIS; ANKYLOSING SPONDYLITIS; PSORIATIC ARTHRITIS and CROHN'S DISEASE. | - MAb cA2 - Monoclonal Antibody cA2 - Infliximab dyyb - Inflectra - Remicade - Infliximab abda - Renflexis | "Infliximab" [Mesh] OR "Infliximab" OR "MAb cA2" OR "Monoclonal Antibody cA2" OR "Infliximab dyyb" OR "Inflectra" OR "Remicade" OR "Infliximab abda" OR "Renflexis"  [Proteins](https://www.ncbi.nlm.nih.gov/mesh/68011506)  [Blood Proteins](https://www.ncbi.nlm.nih.gov/mesh/68001798)  [Immunoproteins](https://www.ncbi.nlm.nih.gov/mesh/68007162)  [Immunoglobulins](https://www.ncbi.nlm.nih.gov/mesh/68007136)  [Antibodies](https://www.ncbi.nlm.nih.gov/mesh/68000906)  [Antibodies, Monoclonal](https://www.ncbi.nlm.nih.gov/mesh/68000911)  Infliximab |
|  | [Adalimumab - MeSH - NCBI “nih.gov)](https://www.ncbi.nlm.nih.gov/mesh/2009894)  A humanized monoclonal antibody that binds specifically to TNF-ALPHA and blocks its interaction with endogenous TNF RECEPTORS to modulate INFLAMMATION. It is used in the treatment of RHEUMATOID ARTHRITIS; PSORIATIC ARTHRITIS; CROHN'S DISEASE and ULCERATIVE COLITIS. | - Humira - Adalimumab-adbm - Amjevita - Adalimumab-atto - Cyltezo - D2E7 Antibody | "Adalimumab" [Mesh] OR "Adalimumab" OR "Humira" OR "Adalimumab-adbm" OR "Amjevita" OR "Adalimumab-atto" OR "Cyltezo" OR "D2E7 Antibody"  [Immunoproteins](https://www.ncbi.nlm.nih.gov/mesh/68007162)  [Immunoglobulins](https://www.ncbi.nlm.nih.gov/mesh/68007136)  [Antibodies](https://www.ncbi.nlm.nih.gov/mesh/68000906)  [Antibodies, Monoclonal](https://www.ncbi.nlm.nih.gov/mesh/68000911)  [Antibodies, Monoclonal, Humanized](https://www.ncbi.nlm.nih.gov/mesh/68061067)  Adalimumab |
|  | [golimumab - MeSH - NCBI “nih.gov)](https://www.ncbi.nlm.nih.gov/mesh/?term=Golimumab)  Golimumab plus MTX effectively reduces the signs and symptoms of RA and is generally well tolerated in patients with an inadequate response to MTX. | - CNTO 148 - Simponi | "golimumab" [Mesh] OR "golimumab" OR "CNTO 148" OR "Simponi" |
|  | [Certolizumab Pegol - MeSH - NCBI “nih.gov)](https://www.ncbi.nlm.nih.gov/mesh/?term=Certolizumab)  A polyethylene-glycolated Fab' fragment of TUMOR NECROSIS FACTOR antibody that binds specifically to TNF-ALPHA and neutralises it in a dose-dependent manner. It also inhibits the production of lipopolysaccharide-induced TNF-ALPHA and IL-1 BETA and is used to treat RHEUMATOID ARTHRITIS and PSORIATIC ARTHRITIS. | - Cimzia - CDP 870 | "Certolizumab Pegol" [Mesh] OR "Certolizumab Pegol" OR "Cimzia" OR "CDP 870"  [Polymers](https://www.ncbi.nlm.nih.gov/mesh/68011108)  [Polyethylene Glycols](https://www.ncbi.nlm.nih.gov/mesh/68011092)  Certolizumab Pegol |
|  | [Abatacept - MeSH - NCBI “nih.gov)](https://www.ncbi.nlm.nih.gov/mesh/?term=abatacept)  A fusion protein immunoconjugate of the extracellular domain of CTLA4 and the Fc domain of human IgG1. It functions as a T-cell co-stimulation blocker that inhibits TNF-ALPHA and prevents the activation of T-LYMPHOCYTES. It is used in the treatment of RHEUMATOID ARTHRITIS.  Year introduced: 2016 “2002) | - CTLA-4-Ig - Cytotoxic T Lymphocyte-Associated Antigen 4-Immunoglobulin - Cytotoxic T Lymphocyte Associated Antigen 4 Immunoglobulin - CTLA4-Ig Immunoconjugate - CTLA4 Ig - BMS 224818 - Belatacept - Orencia - BMS 188667 - LEA29Y - Nulojix | "Abatacept" [Mesh] OR "Abatacept" OR "CTLA-4-Ig" OR "Cytotoxic T Lymphocyte-Associated Antigen 4-Immunoglobulin" OR "Cytotoxic T Lymphocyte Associated Antigen 4 Immunoglobulin" OR "CTLA4-Ig Immunoconjugate" OR "CTLA4 Ig" OR "BMS 224818" OR "Belatacept" OR "Orencia" OR "BMS 188667" OR "LEA29Y" OR "Nulojix"  [Proteins](https://www.ncbi.nlm.nih.gov/mesh/68011506)  [Blood Proteins](https://www.ncbi.nlm.nih.gov/mesh/68001798)  [Serum Globulins](https://www.ncbi.nlm.nih.gov/mesh/68012712)  [Immunoglobulins](https://www.ncbi.nlm.nih.gov/mesh/68007136)  [Antibodies](https://www.ncbi.nlm.nih.gov/mesh/68000906)  [Immunoconjugates](https://www.ncbi.nlm.nih.gov/mesh/68018796)  Abatacept |
|  | [CTLA-4 Antigen - MeSH - NCBI “nih.gov)](https://www.ncbi.nlm.nih.gov/mesh/68060908)  An inhibitory T CELL receptor that is closely related to CD28 ANTIGEN. It has specificity for CD80 ANTIGEN and CD86 ANTIGEN and acts as a negative regulator of peripheral T cell function. CTLA-4 antigen is believed to play role in inducing PERIPHERAL TOLERANCE. | - CTLA 4 Antigen - Cytotoxic T-Lymphocyte-Associated Antigen 4 - Cytotoxic T Lymphocyte Associated Antigen 4 - CD152 Antigens - CD152 Antigen - CTLA-4 Protein - CTLA 4 Protein - Cytotoxic T-Lymphocyte Antigen 4 - Cytotoxic T Lymphocyte Antigen 4 | "CTLA-4 Antigen" [Mesh] OR "CTLA-4 Antigen" OR " CTLA 4 Antigen" OR "Cytotoxic T-Lymphocyte-Associated Antigen 4" OR "Cytotoxic T Lymphocyte Associated Antigen 4" OR "CD152 Antigens" OR "CD152 Antigen" OR "CTLA-4 Protein" OR "CTLA 4 Protein" OR "Cytotoxic T-Lymphocyte Antigen 4" OR "Cytotoxic T Lymphocyte Antigen 4"  [Proteins](https://www.ncbi.nlm.nih.gov/mesh/68011506)  [Immune Checkpoint Proteins](https://www.ncbi.nlm.nih.gov/mesh/2051965)  CTLA-4 Antigen |
|  | [secukinumab - MeSH - NCBI “nih.gov)](https://www.ncbi.nlm.nih.gov/mesh/?term=Secukinumab)  a fully human antibody to interleukin-17A for treatment of psoriasis, rheumatoid arthritis, and uveitis | - Cosentyx - AIN 457 - AIN457 | "secukinumab" [Mesh] OR "secukinumab" OR "Cosentyx" OR "AIN 457" OR "AIN457" |
|  | [Interleukin-17 - MeSH - NCBI “nih.gov)](https://www.ncbi.nlm.nih.gov/mesh/68020381)  A proinflammatory cytokine produced primarily by T-LYMPHOCYTES or their precursors. Several subtypes of interleukin-17 have been identified, each of which is a product of a unique gene. | - Interleukin 17 - CTLA-8 - IL-17 - Interleukin-17F - Interleukin 17F - IL-17F - Cytokine ML-1 - Cytokine ML 1 - Interleukin-17C - Interleukin 17C - IL-17C - Cytokine CX2 - Interleukin-17E - Interleukin 17E - Interleukin 25 - IL-17E - IL 17E - Interleukin-17A - Interleukin 17A - Cytotoxic T lymphocyte Associated Antigen 8 - IL-17A - CTLA8 - Interleukin 17B - IL-17B | "Interleukin-17" [Mesh] OR "Interleukin-17" OR " Interleukin 17" OR "CTLA-8" OR "IL-17" OR "Interleukin-17F" OR "Interleukin 17F" OR "IL-17F" OR "Cytokine ML-1" OR "Cytokine ML 1" OR "Interleukin-17C" OR "Interleukin 17C" OR "IL-17C" OR "Cytokine CX2" OR "Interleukin-17E" OR "Interleukin 17E" OR "Interleukin 25" OR "IL-17E" OR "IL 17E" OR "Interleukin-17A" OR "Interleukin 17A" OR "Cytotoxic T lymphocyte Associated Antigen 8" OR "IL-17A" OR "CTLA8" OR "Interleukin 17B" OR "IL-17B"  [Cytokines](https://www.ncbi.nlm.nih.gov/mesh/68016207)  [Interleukins](https://www.ncbi.nlm.nih.gov/mesh/68007378)  Interleukin-17 |
|  | [ixekizumab - MeSH - NCBI “nih.gov)](https://www.ncbi.nlm.nih.gov/mesh/?term=Ixekizumab)  A humanized anti-interleukin 17 monoclonal antibody used for the treatment of PSORIASIS. | - Taltz - LY2439821 | "ixekizumab" [Mesh] OR "ixekizumab" OR "Taltz" OR "LY2439821" |
|  | [brodalumab - MeSH - NCBI “nih.gov)](https://www.ncbi.nlm.nih.gov/mesh/?term=Brodalumab)  an anti-interleukin-17-receptor antibody for treatment of psoriasis | - Siliq - KHK4827 - AMG 827 | "brodalumab" [Mesh] OR "brodalumab" OR "Siliq" OR "KHK4827" OR "AMG 827" |
|  | [Ustekinumab - MeSH - NCBI “nih.gov)](https://www.ncbi.nlm.nih.gov/mesh/?term=Ustekinumab)  A humanized monoclonal antibody that binds to IL-12 and IL-23 and is used as a DERMATOLOGIC AGENT in the treatment of patients with plaque PSORIASIS who have not responded to other therapies. | - Stelara - CNTO 1275 | "Ustekinumab" [Mesh] OR "Ustekinumab" OR "Stelara" OR "CNTO 1275"  [Antibodies, Monoclonal](https://www.ncbi.nlm.nih.gov/mesh/68000911)  [Antibodies, Monoclonal, Humanized](https://www.ncbi.nlm.nih.gov/mesh/68061067)  Ustekinumab |
|  | [Interleukin-12 - MeSH - NCBI “nih.gov)](https://www.ncbi.nlm.nih.gov/mesh/68018664)  A heterodimeric cytokine that plays a role in innate and adaptive immune responses. Interleukin-12 is a 70 kDa protein that is composed of covalently linked 40 kDa and 35 kDa subunits. It is produced by DENDRITIC CELLS; MACROPHAGES and a variety of other immune cells and plays a role in the stimulation of INTERFERON-GAMMA production by T-LYMPHOCYTES and NATURAL KILLER CELLS. | - Natural Killer Cell Stimulatory Factor - IL-12 - Cytotoxic Lymphocyte Maturation Factor - IL 12 - Interleukin 12 p70 - Interleukin 12 - IL12 - Edodekin Alfa | "Interleukin-12" [Mesh] OR "Interleukin-12" OR "Natural Killer Cell Stimulatory Factor" OR "IL-12" OR "Cytotoxic Lymphocyte Maturation Factor" OR "IL 12" OR "Interleukin 12 p70" OR "Interleukin 12" OR "IL12" OR "Edodekin Alfa"  [Cytokines](https://www.ncbi.nlm.nih.gov/mesh/68016207)  [Interleukins](https://www.ncbi.nlm.nih.gov/mesh/68007378)  Interleukin-12  [Interleukin-12 Subunit p35](https://www.ncbi.nlm.nih.gov/mesh/68053765)  [Interleukin-12 Subunit p40](https://www.ncbi.nlm.nih.gov/mesh/68053762) |
|  | [Interleukin-23 - MeSH - NCBI “nih.gov)](https://www.ncbi.nlm.nih.gov/mesh/68053759)  A heterodimeric cytokine that plays a role in innate and adaptive immune responses. Interleukin-23 is comprised of a unique 19 kDa subunit and 40 kDa subunit that is shared with INTERLEUKIN-12. It is produced by DENDRITIC CELLS; MACROPHAGES and a variety of other immune cells | - Interleukin 23 - IL-23 | "Interleukin-23" [Mesh] OR "Interleukin-23" OR "Interleukin 23" OR "IL-23"  [Cytokines](https://www.ncbi.nlm.nih.gov/mesh/68016207)  [Interleukins](https://www.ncbi.nlm.nih.gov/mesh/68007378)  Interleukin-23  [Interleukin-12 Subunit p40](https://www.ncbi.nlm.nih.gov/mesh/68053762)  [Interleukin-23 Subunit p19](https://www.ncbi.nlm.nih.gov/mesh/68053760) |
|  | [risankizumab - MeSH - NCBI “nih.gov)](https://www.ncbi.nlm.nih.gov/mesh/?term=Risankizumab)  a fully human IgG1 mAb selective for IL-23A; for treatment of moderate-to-severe psoriasis | - skyrizi - risankizumab-rzaa - BI 655066 | "risankizumab" [Mesh] OR "risankizumab" OR " skyrizi" OR "risankizumab-rzaa" OR "BI 655066" |
|  | [guselkumab - MeSH - NCBI “nih.gov)](https://www.ncbi.nlm.nih.gov/mesh/?term=Guselkumab)  specific for IL-23 | - Tremfya - CNTO 1959 | "guselkumab" [Mesh] OR "guselkumab" OR "Tremfya" OR "CNTO 1959" |
|  | [vedolizumab - MeSH - NCBI “nih.gov)](https://www.ncbi.nlm.nih.gov/mesh/?term=vedolizumab)  a humanized monoclonal antibody that targets the 4beta7 integrin exclusively, and modulates inflammation in the gastrointestinal tract | - Entyvio - MLN0002 - MLN02 - MLN-0002 | "vedolizumab" [Mesh] OR "vedolizumab" OR "Entyvio" OR "MLN0002" OR "MLN02" OR "MLN-0002" |
|  | c[- MeSH - NCBI “nih.gov)](https://www.ncbi.nlm.nih.gov/mesh/68016023)  A family of transmembrane glycoproteins “MEMBRANE GLYCOPROTEINS) consisting of noncovalent heterodimers. They interact with a wide variety of ligands including EXTRACELLULAR MATRIX PROTEINS; COMPLEMENT, and other cells, while their intracellular domains interact with the CYTOSKELETON. The integrins consist of at least three identified families: the cytoadhesin receptors “RECEPTORS, CYTOADHESIN), the leukocyte adhesion receptors “RECEPTORS, LEUKOCYTE ADHESION), and the VERY LATE ANTIGEN RECEPTORS. Each family contains a common beta-subunit “INTEGRIN BETA CHAINS) combined with one or more distinct alpha-subunits “INTEGRIN ALPHA CHAINS). These receptors participate in cell-matrix and cell-cell adhesion in many physiologically important processes, including embryological development; HEMOSTASIS; THROMBOSIS; WOUND HEALING; immune and nonimmune defense mechanisms; and oncogenic transformation. | - Integrin | "Integrins" [Mesh] OR "Integrins" OR "Integrin"  [Receptors, Immunologic](https://www.ncbi.nlm.nih.gov/mesh/68011971)  Integrins  [Integrin alpha Chains](https://www.ncbi.nlm.nih.gov/mesh/68039001)  [CD11a Antigen](https://www.ncbi.nlm.nih.gov/mesh/68040881)  [CD11b Antigen](https://www.ncbi.nlm.nih.gov/mesh/68039481)  [CD11c Antigen](https://www.ncbi.nlm.nih.gov/mesh/68039521)  [Integrin alpha1](https://www.ncbi.nlm.nih.gov/mesh/68039423)  [Integrin alpha2](https://www.ncbi.nlm.nih.gov/mesh/68039421)  [Integrin alpha3](https://www.ncbi.nlm.nih.gov/mesh/68039422)  [Integrin alpha4](https://www.ncbi.nlm.nih.gov/mesh/68039441)  [Integrin alpha5](https://www.ncbi.nlm.nih.gov/mesh/68039482)  [Integrin alpha6](https://www.ncbi.nlm.nih.gov/mesh/68039503)  [Integrin alphaV](https://www.ncbi.nlm.nih.gov/mesh/68039564)  [Platelet Membrane Glycoprotein IIb](https://www.ncbi.nlm.nih.gov/mesh/68040201)  [Integrin beta Chains](https://www.ncbi.nlm.nih.gov/mesh/68039641)  [CD18 Antigens](https://www.ncbi.nlm.nih.gov/mesh/68018821)  [Integrin beta1](https://www.ncbi.nlm.nih.gov/mesh/68019012)  [Integrin beta3](https://www.ncbi.nlm.nih.gov/mesh/68039661)  [Integrin beta4](https://www.ncbi.nlm.nih.gov/mesh/68039663)  [Receptors, Cytoadhesin](https://www.ncbi.nlm.nih.gov/mesh/68016024)  [Integrin alpha6beta4](https://www.ncbi.nlm.nih.gov/mesh/68039161)  [Platelet Glycoprotein GPIIb-IIIa Complex](https://www.ncbi.nlm.nih.gov/mesh/68019039)  [Receptors, Vitronectin](https://www.ncbi.nlm.nih.gov/mesh/68019030) +  [Receptors, Fibrinogen](https://www.ncbi.nlm.nih.gov/mesh/68039341)  [Macrophage-1 Antigen](https://www.ncbi.nlm.nih.gov/mesh/68016177)  [Receptors, Fibronectin](https://www.ncbi.nlm.nih.gov/mesh/68017469)  [Integrin alpha2beta1](https://www.ncbi.nlm.nih.gov/mesh/68038982)  [Integrin alpha4beta1](https://www.ncbi.nlm.nih.gov/mesh/68039041)  [Integrin alpha5beta1](https://www.ncbi.nlm.nih.gov/mesh/68039081)  [Receptors, Leukocyte-Adhesion](https://www.ncbi.nlm.nih.gov/mesh/68016028)  [Integrin alphaXbeta2](https://www.ncbi.nlm.nih.gov/mesh/68016167) +  [Lymphocyte Function-Associated Antigen-1](https://www.ncbi.nlm.nih.gov/mesh/68016169)  [Macrophage-1 Antigen](https://www.ncbi.nlm.nih.gov/mesh/68016177)  [Receptors, Very Late Antigen](https://www.ncbi.nlm.nih.gov/mesh/68016029)  [Integrin alpha1beta1](https://www.ncbi.nlm.nih.gov/mesh/68039222)  [Integrin alpha2beta1](https://www.ncbi.nlm.nih.gov/mesh/68038982)  [Integrin alpha3beta1](https://www.ncbi.nlm.nih.gov/mesh/68039201)  [Integrin alpha4beta1](https://www.ncbi.nlm.nih.gov/mesh/68039041)  [Integrin alpha5beta1](https://www.ncbi.nlm.nih.gov/mesh/68039081)  [Integrin alpha6beta1](https://www.ncbi.nlm.nih.gov/mesh/68039121) |
|  |  |  | NOT "Rituximab" [Mesh] |
| Intervention  Vaccination with inactivated vaccine | [Vaccines, Inactivated - MeSH - NCBI “nih.gov)](https://www.ncbi.nlm.nih.gov/mesh/68015164)  Definition: Vaccines in which the infectious microbial nucleic acid components have been destroyed by chemical or physical treatment “e.g., formalin, beta-propiolactone, gamma radiation) without affecting the antigenicity or immunogenicity of the viral coat or bacterial outer membrane proteins. | Inactivated Vaccines  Killed Vaccine  Killed Vaccines  Inactivated Vaccine | "[Vaccines, Inactivated](https://www.ncbi.nlm.nih.gov/mesh/68015164)" [Mesh] OR "[Vaccines, Inactivated](https://www.ncbi.nlm.nih.gov/mesh/68015164)" OR "Inactivated Vaccines" OR "Killed Vaccine" OR "Killed Vaccines" OR "Inactivated Vaccine"  Vaccines, Inactivated  [Poliovirus Vaccine, Inactivated](https://www.ncbi.nlm.nih.gov/mesh/68011054) |
|  | [Influenza Vaccines - MeSH - NCBI “nih.gov)](https://www.ncbi.nlm.nih.gov/mesh/68007252)  Vaccines used to prevent infection by viruses in the family ORTHOMYXOVIRIDAE. It includes both killed and attenuated vaccines. The composition of the vaccines is changed each year in response to antigenic shifts and changes in prevalence of influenza virus strains. The flu vaccines may be mono- or multi-valent, which contains one or more INFLUENZAVIRUS A and INFLUENZAVIRUS B strains. | - Influenza Vaccine - Influenzavirus Vaccines - Influenza Virus Vaccines - Influenzavirus Vaccine - Influenza Virus Vaccine - High-Dose Trivalent Influenza Vaccine - High Dose Trivalent Influenza Vaccine - Trivalent Influenza Vaccine - Quadrivalent Influenza Vaccine - High dose | "[Influenza Vaccines](https://www.ncbi.nlm.nih.gov/mesh/68007252)" [Mesh:NoExp] OR Influenza Vaccine" OR "Influenzavirus Vaccines" OR "Influenza Virus Vaccines" OR "Influenzavirus Vaccine" OR "Influenza Virus Vaccine" OR "High-Dose Trivalent Influenza Vaccine" OR "High Dose Trivalent Influenza Vaccine" OR "Trivalent Influenza Vaccine" OR "Quadrivalent Influenza Vaccine"  “não utilizar [Mesh] pois a árvore do termo inclui VVA e intra-nasal)  [Vaccines](https://www.ncbi.nlm.nih.gov/mesh/68014612)  [Viral Vaccines](https://www.ncbi.nlm.nih.gov/mesh/68014765)  Influenza Vaccines |
|  | [Pneumococcal Vaccines - MeSH - NCBI “nih.gov)](https://www.ncbi.nlm.nih.gov/mesh/68022242)  Vaccines or candidate vaccines used to prevent infections with STREPTOCOCCUS PNEUMONIAE. | - Pneumococcal Vaccine - Pneumovax - Pneumococcal Polysaccharide Vaccine - Pnu Imune Vaccine - PnuImune Vaccine - Conjugated penumococcal vaccine | "[Pneumococcal Vaccines](https://www.ncbi.nlm.nih.gov/mesh/68022242)" [Mesh] OR "[Pneumococcal Vaccines](https://www.ncbi.nlm.nih.gov/mesh/68022242)" OR "Pneumococcal Vaccine" OR "Pneumovax" OR "Pneumococcal Polysaccharide Vaccine" OR "Pnu Imune Vaccine" OR "PnuImune Vaccine" OR "Conjugated penumococcal vaccine"  Pneumococcal Vaccines  [Heptavalent Pneumococcal Conjugate Vaccine](https://www.ncbi.nlm.nih.gov/mesh/2009980)  13 pneumococcal conjugate vaccine |
|  | [Herpes Zoster Vaccine - MeSH - NCBI “nih.gov)](https://www.ncbi.nlm.nih.gov/mesh/68053061)  An attenuated vaccine used to prevent and/or treat HERPES ZOSTER, a disease caused by HUMAN HERPESVIRUS 3. | - Zoster Vaccine - Shingles Vaccine | "Herpes Zoster Vaccine" [Mesh:NoExp] OR " Zoster Vaccine" OR "Shingles Vaccine"  “não utilizar [Mesh] pois a árvore do termo inclui live attenuated zoster  e intra-nasal)  [Vaccines](https://www.ncbi.nlm.nih.gov/mesh/68014612)  [Viral Vaccines](https://www.ncbi.nlm.nih.gov/mesh/68014765)  [Herpesvirus Vaccines](https://www.ncbi.nlm.nih.gov/mesh/68022283)  Herpes Zoster Vaccine |
|  | [Hepatitis B Vaccines - MeSH - NCBI “nih.gov)](https://www.ncbi.nlm.nih.gov/mesh/68017325)  Vaccines or candidate vaccines containing inactivated hepatitis B or some of its component antigens and designed to prevent hepatitis B. Some vaccines may be recombinantly produced. | Hepatitis B Vaccine | "Hepatitis B Vaccines" [Mesh] OR "Hepatitis B Vaccines" OR "Hepatitis B Vaccine"  [Vaccines](https://www.ncbi.nlm.nih.gov/mesh/68014612)  [Viral Vaccines](https://www.ncbi.nlm.nih.gov/mesh/68014765)  [Viral Hepatitis Vaccines](https://www.ncbi.nlm.nih.gov/mesh/68014761)  Hepatitis B Vaccines |
|  | [Papillomavirus Vaccines - MeSH - NCBI “nih.gov)](https://www.ncbi.nlm.nih.gov/mesh/?term=papillomavirus+vaccines)   Vaccines or candidate vaccines used to prevent PAPILLOMAVIRUS INFECTIONS. Human vaccines are intended to reduce the incidence of UTERINE CERVICAL NEOPLASMS, so they are sometimes considered a type of CANCER VACCINES. They are often composed of CAPSID PROTEINS, especially L1 protein, from various types of ALPHAPAPILLOMAVIRUS | - Papillomavirus Vaccine   Human Papillomavirus Vaccines   - HPV Vaccine   Human Papilloma Virus Vaccine   - HPV Vaccines - Human Papilloma Virus Vaccines - Human Papillomavirus Vaccine | "Papillomavirus Vaccines" [Mesh] OR "Papillomavirus Vaccines" OR "Papillomavirus Vaccine" OR "Human Papillomavirus Vaccines" OR "HPV Vaccine" OR "Human Papilloma Virus Vaccine" OR "HPV Vaccines" OR "Human Papilloma Virus Vaccines" OR "Human Papillomavirus Vaccine"  [Vaccines](https://www.ncbi.nlm.nih.gov/mesh/68014612)  [Viral Vaccines](https://www.ncbi.nlm.nih.gov/mesh/68014765)  Papillomavirus Vaccines  [Human Papillomavirus](https://www.ncbi.nlm.nih.gov/mesh/2009889) |
|  | [Poliovirus Vaccines - MeSH - NCBI “nih.gov)](https://www.ncbi.nlm.nih.gov/mesh/68023321)    Vaccines used to prevent POLIOMYELITIS. They include inactivated “POLIOVIRUS VACCINE, INACTIVATED) | - Poliovirus Vaccine - salk | "Poliovirus Vaccines" [Mesh] OR "Poliovirus Vaccines" OR "Poliovirus Vaccine" OR "salk"  [Vaccines](https://www.ncbi.nlm.nih.gov/mesh/68014612)  [Viral Vaccines](https://www.ncbi.nlm.nih.gov/mesh/68014765)  [Poliovirus Vaccine, Inactivated](https://www.ncbi.nlm.nih.gov/mesh/68011054) |
|  | [Hepatitis A Vaccines - MeSH - NCBI “nih.gov)](https://www.ncbi.nlm.nih.gov/mesh/68022362)    Vaccines or candidate vaccines used to prevent infection with hepatitis A virus “HEPATOVIRUS). | - Hepatovirus Vaccines - Hepatitis A Vaccine - Vaqta - Havrix | "Hepatitis A Vaccines" [Mesh] OR "Hepatitis A Vaccines" OR "Hepatovirus Vaccines" OR "Hepatitis A Vaccine" OR "Vaqta" OR "Havrix"  [Vaccines](https://www.ncbi.nlm.nih.gov/mesh/68014612)  [Viral Hepatitis Vaccines](https://www.ncbi.nlm.nih.gov/mesh/68014761)  Hepatitis A Vaccines |
|  | [Meningococcal Vaccines - MeSH - NCBI “nih.gov)](https://www.ncbi.nlm.nih.gov/mesh/68022401)    Vaccines or candidate vaccines used to prevent infection with NEISSERIA MENINGITIDIS. | Meningococcal Vaccine  Menactra  Tetravalent Meningococcal Vaccine  Menomune  Menveo  Bivalent Meningococcal Vaccine  Meningococcal Polysaccharide Vaccine | "Meningococcal Vaccines" [Mesh] OR " Meningococcal Vaccine" OR "Menactra" OR "Tetravalent Meningococcal Vaccine" OR "Menomune OR "Menveo" OR "Bivalent Meningococcal Vaccine" OR "Meningococcal Polysaccharide Vaccine"    [Vaccines](https://www.ncbi.nlm.nih.gov/mesh/68014612)  Meningococcal Vaccines C, B or ACWY |
|  | [Diphtheria-Tetanus-Pertussis Vaccine - MeSH - NCBI “nih.gov)](https://www.ncbi.nlm.nih.gov/mesh/?term=vaccine+per)   A vaccine consisting of DIPHTHERIA TOXOID; TETANUS TOXOID; and whole-cell PERTUSSIS VACCINE. The vaccine protects against diphtheria, tetanus, and whooping cough. | Diphtheria Tetanus Pertussis Vaccine  DTwP Vaccine  DPT Vaccine  DTP Vaccine  Di Te Per Vaccine  Diphtheria Pertussis Tetanus Vaccine | "Diphtheria-Tetanus-Pertussis Vaccine" [Mesh] OR "Diphtheria-Tetanus-Pertussis Vaccine" OR " Diphtheria Tetanus Pertussis Vaccine" OR "DTwP Vaccine" OR "DPT Vaccine" OR "DTP Vaccine" OR "Di Te Per Vaccine" OR "Diphtheria Pertussis Tetanus Vaccine"  [Vaccines](https://www.ncbi.nlm.nih.gov/mesh/68014612)  [Pertussis Vaccine](https://www.ncbi.nlm.nih.gov/mesh/68010567)  Diphtheria-Tetanus-Pertussis Vaccine |
|  | [Haemophilus influenza type b polysaccharide vaccine-tetanus toxin conj - MeSH - NCBI “nih.gov)](https://www.ncbi.nlm.nih.gov/mesh/?term=haemophilus+influenza+type+b+polysaccharide+vaccine+tetanus+toxin+conjugate)    the type b capsular polysaccharide of HIB-vaccine is conjugated to tetanus toxoid | HIBest  PRP-T  Hib-TT  Hib capsular polysaccharide-tetanus vaccine  Hib CPS-tetanus vaccine  OmniHIB  ActHIB | "Haemophilus influenza type b polysaccharide vaccine-tetanus toxin conjugate" OR "HIBest" OR "PRP-T" OR "Hib-TT" OR "Hib capsular polysaccharide-tetanus vaccine" OR "Hib CPS-tetanus vaccine" OR "OmniHIB" OR "ActHIB" |

**Question 06.** What is the impact of the RTX use on the response to inactivated vaccine in patients with immune-mediated rheumatic diseases?

| Group | MeSH Terms  and definitions | Entry Terms | Search Key |
| --- | --- | --- | --- |
| Population  Patients with immune-mediated rheumatic diseases “IMRD) | [Rheumatic Diseases - MeSH - NCBI “nih.gov)](https://www.ncbi.nlm.nih.gov/mesh/68012216)  Disorders of connective tissue, especially the joints and related structures, characterized by inflammation, degeneration, or metabolic derangement. | Rheumatic Disease  Rheumatism | "[Rheumatic Diseases](https://www.ncbi.nlm.nih.gov/mesh/68012216)" [Mesh:NoExp] OR "Rheumatic Disease" OR "Rheumatism" OR  "Immune-mediated rheumatic diseases"    “não utilizar [Mesh] pois a árvore do termo inclui gota, fibromialgia e crianças) |
|  | [Arthritis, Rheumatoid - MeSH - NCBI “nih.gov)](https://www.ncbi.nlm.nih.gov/mesh/68001172)  A chronic systemic disease, primarily of the joints, marked by inflammatory changes in the synovial membranes and articular structures, widespread fibrinoid degeneration of the collagen fibers in mesenchymal tissues, and by atrophy and rarefaction of bony structures. Etiology is unknown, but autoimmune mechanisms have been implicated. | - Rheumatoid Arthritis | "[Arthritis, Rheumatoid](https://www.ncbi.nlm.nih.gov/mesh/68001172)" [Mesh:NoExp] OR "Rheumatoid Arthritis" OR "[Rheumatoid Nodule](https://www.ncbi.nlm.nih.gov/mesh/68012218)" OR "[Rheumatoid Vasculitis](https://www.ncbi.nlm.nih.gov/mesh/68056653)" OR "[Sjogren's Syndrome](https://www.ncbi.nlm.nih.gov/mesh/68012859)"  “não utilizar [Mesh] pois a árvore do termo inclui caplan syndrome, felty syndrome) |
|  | [Spondylarthritis - MeSH - NCBI “nih.gov)](https://www.ncbi.nlm.nih.gov/mesh/68025241)  Inflammation of the joints of the SPINE, the intervertebral articulations. | - Spondylarthritides - Spinal Arthritis - Spinal Arthritides | "[Spondylarthritis](https://www.ncbi.nlm.nih.gov/mesh/68025241)" [Mesh] OR "[Spondylarthritis](https://www.ncbi.nlm.nih.gov/mesh/68025241)" OR "Spondylarthritides" OR "Spinal Arthritis" OR "Spinal Arthritides"  [Spondylitis](https://www.ncbi.nlm.nih.gov/mesh/68013166)  Spondylarthritis  [Osteoarthritis, Spine](https://www.ncbi.nlm.nih.gov/mesh/68055013)  [Spondylarthropathies](https://www.ncbi.nlm.nih.gov/mesh/68025242)  [Arthritis, Psoriatic](https://www.ncbi.nlm.nih.gov/mesh/68015535)  [Arthritis, Reactive](https://www.ncbi.nlm.nih.gov/mesh/68016918)  [Axial Spondyloarthritis](https://www.ncbi.nlm.nih.gov/mesh/2101123) + |
|  | [Arthritis, Psoriatic - MeSH - NCBI “nih.gov)](https://www.ncbi.nlm.nih.gov/mesh/68015535)  A type of inflammatory arthritis associated with PSORIASIS, often involving the axial joints and the peripheral terminal interphalangeal joints. It is characterized by the presence of HLA-B27-associated SPONDYLARTHROPATHY, and the absence of rheumatoid factor. | - Arthritic Psoriasis - Psoriatic Arthritis - Psoriasis Arthropathica - Psoriatic Arthropathy - Psoriatic Arthropathies | "[Arthritis, Psoriatic](https://www.ncbi.nlm.nih.gov/mesh/68015535)" [Mesh] OR "[Arthritis, Psoriatic](https://www.ncbi.nlm.nih.gov/mesh/68015535)" OR "Arthritic Psoriasis" OR "Psoriatic Arthritis" OR "Psoriasis Arthropathica" OR "Psoriatic Arthropathy" OR "Psoriatic Arthropathies"  [Spondylarthritis](https://www.ncbi.nlm.nih.gov/mesh/68025241)  [Spondylarthropathies](https://www.ncbi.nlm.nih.gov/mesh/68025242)  Arthritis, Psoriatic |
|  | [Spondylitis, Ankylosing - MeSH - NCBI “nih.gov)](https://www.ncbi.nlm.nih.gov/mesh/?term=ankylosing+spondylitis)  A chronic inflammatory condition affecting the axial joints, such as the SACROILIAC JOINT and other intervertebral or costovertebral joints. It occurs predominantly in young males and is characterized by pain and stiffness of joints “ANKYLOSIS) with inflammation at tendon insertions. | - Bechterew's Disease - Bechterews Disease - Marie Struempell Disease - Spondylarthritis Ankylopoietica - Spondyloarthritis Ankylopoietica - Ankylosing Spondylitis - Ankylosing Spondylarthritis - Ankylosing Spondylarthritides - Ankylosing Spondyloarthritis - Ankylosing Spondyloarthritides - Spondylitis Ankylopoietica - Bechterew Disease - Rheumatoid Spondylitis | "[Spondylitis, Ankylosing](https://www.ncbi.nlm.nih.gov/mesh/?term=ankylosing+spondylitis)" [Mesh] OR "[Spondylitis, Ankylosing](https://www.ncbi.nlm.nih.gov/mesh/?term=ankylosing+spondylitis)" OR "Bechterew's Disease" OR "Bechterews Disease" OR "Marie Struempell Disease" OR "Spondylarthritis Ankylopoietica" OR "Spondyloarthritis Ankylopoietica" OR "Ankylosing Spondylitis" OR "Ankylosing Spondylarthritis" OR "Ankylosing Spondylarthritides" OR "Ankylosing Spondyloarthritis" OR "Ankylosing Spondyloarthritides" OR "Spondylitis Ankylopoietica" OR "Bechterew Disease" OR "Rheumatoid Spondylitis"  [Spondylarthritis](https://www.ncbi.nlm.nih.gov/mesh/68025241)  [Spondylarthropathies](https://www.ncbi.nlm.nih.gov/mesh/68025242)  [Axial Spondyloarthritis](https://www.ncbi.nlm.nih.gov/mesh/2101123)  Spondylitis, Ankylosing |
|  | [Lupus Erythematosus, Systemic - MeSH - NCBI “nih.gov)](https://www.ncbi.nlm.nih.gov/mesh/68008180)  A chronic, relapsing, inflammatory, and often febrile multisystemic disorder of connective tissue, characterized principally by involvement of the skin, joints, kidneys, and serosal membranes. It is of unknown etiology, but is thought to represent a failure of the regulatory mechanisms of the autoimmune system. The disease is marked by a wide range of system dysfunctions, an elevated erythrocyte sedimentation rate, and the formation of LE cells in the blood or bone marrow. | - Systemic Lupus Erythematosus - Lupus Erythematosus Disseminatus - Libman Sacks Disease | "[Lupus Erythematosus, Systemic](https://www.ncbi.nlm.nih.gov/mesh/68008180)" [Mesh] OR "[Lupus Erythematosus, Systemic](https://www.ncbi.nlm.nih.gov/mesh/68008180)" OR "Systemic Lupus Erythematosus " OR "Lupus Erythematosus Disseminatus" OR "Libman Sacks Disease"  [Connective Tissue Diseases](https://www.ncbi.nlm.nih.gov/mesh/68003240)  Lupus Erythematosus, Systemic  [Lupus Nephritis](https://www.ncbi.nlm.nih.gov/mesh/68008181)  [Lupus Vasculitis, Central Nervous System](https://www.ncbi.nlm.nih.gov/mesh/68020945) |
|  | [Myositis - MeSH - NCBI “nih.gov)](https://www.ncbi.nlm.nih.gov/mesh/?term=inflammatory+myopathies)  Inflammation of a muscle or muscle tissue. | - Myositides - Inflammatory Muscle Diseases - Inflammatory Muscle Disease - Inflammatory Myopathy - Inflammatory Myopathies - Proliferative Myositides - Proliferative Myositis - Infectious Myositides - Infectious Myositis - Idiopathic Inflammatory Myopathies - Idiopathic Inflammatory Myopathy - Idiopathic Inflammatory Myositis - Focal Myositides - Focal Myositis | "[Myositis](https://www.ncbi.nlm.nih.gov/mesh/?term=inflammatory+myopathies)" [Mesh] OR "[Myositis](https://www.ncbi.nlm.nih.gov/mesh/?term=inflammatory+myopathies)" OR "Myositides" OR "Inflammatory Muscle Diseases" OR "Inflammatory Muscle Disease" OR "Inflammatory Myopathy" OR "Inflammatory Myopathies" OR "Proliferative Myositides" OR "Proliferative Myositis" OR "Infectious Myositides" OR "Infectious Myositis" OR "Idiopathic Inflammatory Myopathies" OR "Idiopathic Inflammatory Myopathy" OR "Idiopathic Inflammatory Myositis" OR "Focal Myositides" OR "Focal Myositis"  [Muscular Diseases](https://www.ncbi.nlm.nih.gov/mesh/68009135)  Myositis  [Myositis Ossificans](https://www.ncbi.nlm.nih.gov/mesh/68009221)  [Myositis, Inclusion Body](https://www.ncbi.nlm.nih.gov/mesh/68018979)  [Orbital Myositis](https://www.ncbi.nlm.nih.gov/mesh/68055622)  [Polymyositis](https://www.ncbi.nlm.nih.gov/mesh/68017285)  [Dermatomyositis](https://www.ncbi.nlm.nih.gov/mesh/68003882)  [Pyomyositis](https://www.ncbi.nlm.nih.gov/mesh/68052880) |
|  | [Systemic Vasculitis - MeSH - NCBI “nih.gov)](https://www.ncbi.nlm.nih.gov/mesh/?term=systemic+vasculitides)  A heterogeneous group of diseases characterized by inflammation and necrosis of the blood vessel walls. | - Systemic Vasculitides | "[Systemic Vasculitis](https://www.ncbi.nlm.nih.gov/mesh/?term=systemic+vasculitides)" [Mesh] OR "[Systemic Vasculitis](https://www.ncbi.nlm.nih.gov/mesh/?term=systemic+vasculitides)" OR "Systemic Vasculitides"  [Vasculitis](https://www.ncbi.nlm.nih.gov/mesh/68014657)  Systemic Vasculitis  [Anti-Neutrophil Cytoplasmic Antibody-Associated Vasculitis](https://www.ncbi.nlm.nih.gov/mesh/68056648)  [Churg-Strauss Syndrome](https://www.ncbi.nlm.nih.gov/mesh/68015267)  [Granulomatosis with Polyangiitis](https://www.ncbi.nlm.nih.gov/mesh/68014890)  [Microscopic Polyangiitis](https://www.ncbi.nlm.nih.gov/mesh/68055953)  [Polyarteritis Nodosa](https://www.ncbi.nlm.nih.gov/mesh/68010488)  [Rheumatoid Vasculitis](https://www.ncbi.nlm.nih.gov/mesh/68056653) |
|  | [Sjogren's Syndrome - MeSH - NCBI “nih.gov)](https://www.ncbi.nlm.nih.gov/mesh/68012859)  Chronic inflammatory and autoimmune disease in which the salivary and lacrimal glands undergo progressive destruction by lymphocytes and plasma cells resulting in decreased production of saliva and tears. The primary form, often called sicca syndrome, involves both KERATOCONJUNCTIVITIS SICCA and XEROSTOMIA. The secondary form includes, in addition, the presence of a connective tissue disease, usually rheumatoid arthritis. | - Sjogrens Syndrome - Sjogren Syndrome - Sicca Syndrome | "[Sjogren's Syndrome](https://www.ncbi.nlm.nih.gov/mesh/68012859)" [Mesh] OR "[Sjogren's Syndrome](https://www.ncbi.nlm.nih.gov/mesh/68012859)"  OR "Sjogrens Syndrome" OR "Sjogren Syndrome" OR "Sicca Syndrome"  [Arthritis](https://www.ncbi.nlm.nih.gov/mesh/68001168)  [Arthritis, Rheumatoid](https://www.ncbi.nlm.nih.gov/mesh/68001172)  Sjogren's Syndrome |
|  | [Scleroderma, Systemic - MeSH - NCBI “nih.gov)](https://www.ncbi.nlm.nih.gov/mesh/68012595)  A chronic multi-system disorder of CONNECTIVE TISSUE. It is characterized by SCLEROSIS in the SKIN, the LUNGS, the HEART, the GASTROINTESTINAL TRACT, the KIDNEYS, and the MUSCULOSKELETAL SYSTEM. Other important features include diseased small BLOOD VESSELS and AUTOANTIBODIES. The disorder is named for its most prominent feature “hard skin), and classified into subsets by the extent of skin thickening: LIMITED SCLERODERMA and DIFFUSE SCLERODERMA. | - Systemic Sclerosis - Systemic Scleroderma | "[Scleroderma, Systemic](https://www.ncbi.nlm.nih.gov/mesh/68012595)" [Mesh] OR "[Scleroderma, Systemic](https://www.ncbi.nlm.nih.gov/mesh/68012595)" OR "Systemic Sclerosis" OR "Systemic Scleroderma"  [Connective Tissue Diseases](https://www.ncbi.nlm.nih.gov/mesh/68003240)  Scleroderma, Systemic  [Scleroderma, Diffuse](https://www.ncbi.nlm.nih.gov/mesh/68045743)  [Scleroderma, Limited](https://www.ncbi.nlm.nih.gov/mesh/68045745)  [CREST Syndrome](https://www.ncbi.nlm.nih.gov/mesh/68017675) |
|  | [Mixed Connective Tissue Disease - MeSH - NCBI “nih.gov)](https://www.ncbi.nlm.nih.gov/mesh/?term=Mixed+connective+tissue+disease)  A syndrome with overlapping clinical features of systemic lupus erythematosus, scleroderma, polymyositis, and Raynaud's phenomenon. The disease is differentially characterized by high serum titers of antibodies to ribonuclease-sensitive extractable “saline soluble) nuclear antigen and a "speckled" epidermal nuclear staining pattern on direct immunofluorescence. | - Sharp Syndrome - MCTD | "[Mixed Connective Tissue Disease](https://www.ncbi.nlm.nih.gov/mesh/?term=Mixed+connective+tissue+disease)" [Mesh] OR "[Mixed Connective Tissue Disease](https://www.ncbi.nlm.nih.gov/mesh/?term=Mixed+connective+tissue+disease)" OR "Sharp Syndrome" OR "MCTD"  [Connective Tissue Diseases](https://www.ncbi.nlm.nih.gov/mesh/68003240)  Mixed Connective Tissue Disease |
| InterventionRTX | [Rituximab - MeSH - NCBI “nih.gov)](https://www.ncbi.nlm.nih.gov/mesh/2009941)  A murine-derived monoclonal antibody and ANTINEOPLASTIC AGENT that binds specifically to the CD20 ANTIGEN and is used in the treatment of LEUKEMIA; LYMPHOMA and RHEUMATOID ARTHRITIS. | - Rituximab CD20 Antibody - Mabthera - IDEC C2B8 Antibody - IDEC C2B8 - GP2013 - Rituxan | "Rituximab" [Mesh] OR "Rituximab" OR " Rituximab CD20 Antibody" OR "Mabthera" OR "IDEC C2B8 Antibody" OR "IDEC C2B8" OR "GP2013" OR "Rituxan"  [Antibodies](https://www.ncbi.nlm.nih.gov/mesh/68000906)  [Antibodies, Monoclonal](https://www.ncbi.nlm.nih.gov/mesh/68000911)  [Antibodies, Monoclonal, Murine-Derived](https://www.ncbi.nlm.nih.gov/mesh/68058846)  Rituximab |
| Outcomes  Vaccination with inactivated vaccine | [Vaccines, Inactivated - MeSH - NCBI “nih.gov)](https://www.ncbi.nlm.nih.gov/mesh/68015164)  Definition: Vaccines in which the infectious microbial nucleic acid components have been destroyed by chemical or physical treatment “e.g., formalin, beta-propiolactone, gamma radiation) without affecting the antigenicity or immunogenicity of the viral coat or bacterial outer membrane proteins. | Inactivated Vaccines  Killed Vaccine  Killed Vaccines  Inactivated Vaccine | "[Vaccines, Inactivated](https://www.ncbi.nlm.nih.gov/mesh/68015164)" [Mesh] OR "[Vaccines, Inactivated](https://www.ncbi.nlm.nih.gov/mesh/68015164)" OR "Inactivated Vaccines" OR "Killed Vaccine" OR "Killed Vaccines" OR "Inactivated Vaccine"  Vaccines, Inactivated  [Poliovirus Vaccine, Inactivated](https://www.ncbi.nlm.nih.gov/mesh/68011054) |
|  | [Influenza Vaccines - MeSH - NCBI “nih.gov)](https://www.ncbi.nlm.nih.gov/mesh/68007252)  Vaccines used to prevent infection by viruses in the family ORTHOMYXOVIRIDAE. It includes both killed and attenuated vaccines. The composition of the vaccines is changed each year in response to antigenic shifts and changes in prevalence of influenza virus strains. The flu vaccines may be mono- or multi-valent, which contains one or more INFLUENZAVIRUS A and INFLUENZAVIRUS B strains. | - Influenza Vaccine - Influenzavirus Vaccines - Influenza Virus Vaccines - Influenzavirus Vaccine - Influenza Virus Vaccine - High-Dose Trivalent Influenza Vaccine - High Dose Trivalent Influenza Vaccine - Trivalent Influenza Vaccine - Quadrivalent Influenza Vaccine - High dose | "[Influenza Vaccines](https://www.ncbi.nlm.nih.gov/mesh/68007252)" [Mesh:NoExp] OR Influenza Vaccine" OR "Influenzavirus Vaccines" OR "Influenza Virus Vaccines" OR "Influenzavirus Vaccine" OR "Influenza Virus Vaccine" OR "High-Dose Trivalent Influenza Vaccine" OR "High Dose Trivalent Influenza Vaccine" OR "Trivalent Influenza Vaccine" OR "Quadrivalent Influenza Vaccine"  “não utilizar [Mesh] pois a árvore do termo inclui VVA e intra-nasal)  [Vaccines](https://www.ncbi.nlm.nih.gov/mesh/68014612)  [Viral Vaccines](https://www.ncbi.nlm.nih.gov/mesh/68014765)  Influenza Vaccines |
|  | [Pneumococcal Vaccines - MeSH - NCBI “nih.gov)](https://www.ncbi.nlm.nih.gov/mesh/68022242)  Vaccines or candidate vaccines used to prevent infections with STREPTOCOCCUS PNEUMONIAE. | - Pneumococcal Vaccine - Pneumovax - Pneumococcal Polysaccharide Vaccine - Pnu Imune Vaccine - PnuImune Vaccine - Conjugated penumococcal vaccine | "[Pneumococcal Vaccines](https://www.ncbi.nlm.nih.gov/mesh/68022242)" [Mesh] OR "[Pneumococcal Vaccines](https://www.ncbi.nlm.nih.gov/mesh/68022242)" OR "Pneumococcal Vaccine" OR "Pneumovax" OR "Pneumococcal Polysaccharide Vaccine" OR "Pnu Imune Vaccine" OR "PnuImune Vaccine" OR "Conjugated penumococcal vaccine"  Pneumococcal Vaccines  [Heptavalent Pneumococcal Conjugate Vaccine](https://www.ncbi.nlm.nih.gov/mesh/2009980)  13 pneumococcal conjugate vaccine |
|  | [Herpes Zoster Vaccine - MeSH - NCBI “nih.gov)](https://www.ncbi.nlm.nih.gov/mesh/68053061)  An attenuated vaccine used to prevent and/or treat HERPES ZOSTER, a disease caused by HUMAN HERPESVIRUS 3. | - Zoster Vaccine - Shingles Vaccine | "Herpes Zoster Vaccine" [Mesh:NoExp] OR "Zoster Vaccine" OR "Shingles Vaccine"  “não utilizar [Mesh] pois a árvore do termo inclui live attenuated zoster  e intra-nasal)  [Vaccines](https://www.ncbi.nlm.nih.gov/mesh/68014612)  [Viral Vaccines](https://www.ncbi.nlm.nih.gov/mesh/68014765)  [Herpesvirus Vaccines](https://www.ncbi.nlm.nih.gov/mesh/68022283)  Herpes Zoster Vaccine |
|  | [Hepatitis B Vaccines - MeSH - NCBI “nih.gov)](https://www.ncbi.nlm.nih.gov/mesh/68017325)  Vaccines or candidate vaccines containing inactivated hepatitis B or some of its component antigens and designed to prevent hepatitis B. Some vaccines may be recombinantly produced. | Hepatitis B Vaccine | "Hepatitis B Vaccines" [Mesh] OR "Hepatitis B Vaccines" OR "Hepatitis B Vaccine"  [Vaccines](https://www.ncbi.nlm.nih.gov/mesh/68014612)  [Viral Vaccines](https://www.ncbi.nlm.nih.gov/mesh/68014765)  [Viral Hepatitis Vaccines](https://www.ncbi.nlm.nih.gov/mesh/68014761)  Hepatitis B Vaccines |
|  | [Papillomavirus Vaccines - MeSH - NCBI “nih.gov)](https://www.ncbi.nlm.nih.gov/mesh/?term=papillomavirus+vaccines)   Vaccines or candidate vaccines used to prevent PAPILLOMAVIRUS INFECTIONS. Human vaccines are intended to reduce the incidence of UTERINE CERVICAL NEOPLASMS, so they are sometimes considered a type of CANCER VACCINES. They are often composed of CAPSID PROTEINS, especially L1 protein, from various types of ALPHAPAPILLOMAVIRUS | - Papillomavirus Vaccine   Human Papillomavirus Vaccines   - HPV Vaccine   Human Papilloma Virus Vaccine   - HPV Vaccines - Human Papilloma Virus Vaccines - Human Papillomavirus Vaccine | "Papillomavirus Vaccines" [Mesh] OR "Papillomavirus Vaccines" OR "Papillomavirus Vaccine" OR "Human Papillomavirus Vaccines" OR "HPV Vaccine" OR "Human Papilloma Virus Vaccine" OR "HPV Vaccines" OR "Human Papilloma Virus Vaccines" OR "Human Papillomavirus Vaccine"  [Vaccines](https://www.ncbi.nlm.nih.gov/mesh/68014612)  [Viral Vaccines](https://www.ncbi.nlm.nih.gov/mesh/68014765)  Papillomavirus Vaccines  [Human Papillomavirus](https://www.ncbi.nlm.nih.gov/mesh/2009889) |
|  | [Poliovirus Vaccines - MeSH - NCBI “nih.gov)](https://www.ncbi.nlm.nih.gov/mesh/68023321)    Vaccines used to prevent POLIOMYELITIS. They include inactivated “POLIOVIRUS VACCINE, INACTIVATED) | - Poliovirus Vaccine - salk | "Poliovirus Vaccines" [Mesh] OR "Poliovirus Vaccines" OR "Poliovirus Vaccine" OR "salk"  [Vaccines](https://www.ncbi.nlm.nih.gov/mesh/68014612)  [Viral Vaccines](https://www.ncbi.nlm.nih.gov/mesh/68014765)  [Poliovirus Vaccine, Inactivated](https://www.ncbi.nlm.nih.gov/mesh/68011054) |
|  | [Hepatitis A Vaccines - MeSH - NCBI “nih.gov)](https://www.ncbi.nlm.nih.gov/mesh/68022362)    Vaccines or candidate vaccines used to prevent infection with hepatitis A virus “HEPATOVIRUS). | - Hepatovirus Vaccines - Hepatitis A Vaccine - Vaqta - Havrix | "Hepatitis A Vaccines" [Mesh] OR "Hepatitis A Vaccines" OR "Hepatovirus Vaccines" OR "Hepatitis A Vaccine" OR "Vaqta" OR "Havrix"  [Vaccines](https://www.ncbi.nlm.nih.gov/mesh/68014612)  [Viral Hepatitis Vaccines](https://www.ncbi.nlm.nih.gov/mesh/68014761)  Hepatitis A Vaccines |
|  | [Meningococcal Vaccines - MeSH - NCBI “nih.gov)](https://www.ncbi.nlm.nih.gov/mesh/68022401)    Vaccines or candidate vaccines used to prevent infection with NEISSERIA MENINGITIDIS. | Meningococcal Vaccine  Menactra  Tetravalent Meningococcal Vaccine  Menomune  Menveo  Bivalent Meningococcal Vaccine  Meningococcal Polysaccharide Vaccine | "Meningococcal Vaccines" [Mesh] OR " Meningococcal Vaccine" OR "Menactra" OR "Tetravalent Meningococcal Vaccine" OR "Menomune OR "Menveo" OR "Bivalent Meningococcal Vaccine" OR "Meningococcal Polysaccharide Vaccine"    [Vaccines](https://www.ncbi.nlm.nih.gov/mesh/68014612)  Meningococcal Vaccines C, B or ACWY |
|  | [Diphtheria-Tetanus-Pertussis Vaccine - MeSH - NCBI “nih.gov)](https://www.ncbi.nlm.nih.gov/mesh/?term=vaccine+per)   A vaccine consisting of DIPHTHERIA TOXOID; TETANUS TOXOID; and whole-cell PERTUSSIS VACCINE. The vaccine protects against diphtheria, tetanus, and whooping cough. | Diphtheria Tetanus Pertussis Vaccine  DTwP Vaccine  DPT Vaccine  DTP Vaccine  Di Te Per Vaccine  Diphtheria Pertussis Tetanus Vaccine | "Diphtheria-Tetanus-Pertussis Vaccine" [Mesh] OR "Diphtheria-Tetanus-Pertussis Vaccine" OR " Diphtheria Tetanus Pertussis Vaccine" OR "DTwP Vaccine" OR "DPT Vaccine" OR "DTP Vaccine" OR "Di Te Per Vaccine" OR "Diphtheria Pertussis Tetanus Vaccine"  [Vaccines](https://www.ncbi.nlm.nih.gov/mesh/68014612)  [Pertussis Vaccine](https://www.ncbi.nlm.nih.gov/mesh/68010567)  Diphtheria-Tetanus-Pertussis Vaccine |
|  | [Haemophilus influenza type b polysaccharide vaccine-tetanus toxin conj - MeSH - NCBI “nih.gov)](https://www.ncbi.nlm.nih.gov/mesh/?term=haemophilus+influenza+type+b+polysaccharide+vaccine+tetanus+toxin+conjugate)    the type b capsular polysaccharide of HIB-vaccine is conjugated to tetanus toxoid | HIBest  PRP-T  Hib-TT  Hib capsular polysaccharide-tetanus vaccine  Hib CPS-tetanus vaccine  OmniHIB  ActHIB | "Haemophilus influenza type b polysaccharide vaccine-tetanus toxin conjugate" OR "HIBest" OR "PRP-T" OR "Hib-TT" OR "Hib capsular polysaccharide-tetanus vaccine" OR "Hib CPS-tetanus vaccine" OR "OmniHIB" OR "ActHIB" |

**Question 07.** What is the impact of corticosteroids use on the response to inactivated vaccine in patients with immune-mediated rheumatic diseases?

| Group | **MeSH Terms**  and definitions | **Entry Terms** | **Search Key** |
| --- | --- | --- | --- |
| Population  Patients with immune-mediated rheumatic diseases “IMRD) | [Rheumatic Diseases - MeSH - NCBI “nih.gov)](https://www.ncbi.nlm.nih.gov/mesh/68012216)  Disorders of connective tissue, especially the joints and related structures, characterized by inflammation, degeneration, or metabolic derangement. | Rheumatic Disease  Rheumatism | "[Rheumatic Diseases](https://www.ncbi.nlm.nih.gov/mesh/68012216)" [Mesh:NoExp] OR "Rheumatic Disease" OR "Rheumatism" OR  "Immune-mediated rheumatic diseases"  “não utilizar [Mesh] pois a árvore do termo inclui gota, fibromialgia e crianças) |
|  | [Arthritis, Rheumatoid - MeSH - NCBI “nih.gov)](https://www.ncbi.nlm.nih.gov/mesh/68001172)  A chronic systemic disease, primarily of the joints, marked by inflammatory changes in the synovial membranes and articular structures, widespread fibrinoid degeneration of the collagen fibers in mesenchymal tissues, and by atrophy and rarefaction of bony structures. Etiology is unknown, but autoimmune mechanisms have been implicated. | - Rheumatoid Arthritis | "[Arthritis, Rheumatoid](https://www.ncbi.nlm.nih.gov/mesh/68001172)" [Mesh:NoExp] OR "Rheumatoid Arthritis" OR "[Rheumatoid Nodule](https://www.ncbi.nlm.nih.gov/mesh/68012218)" OR "[Rheumatoid Vasculitis](https://www.ncbi.nlm.nih.gov/mesh/68056653)" OR "[Sjogren's Syndrome](https://www.ncbi.nlm.nih.gov/mesh/68012859)"  “não utilizar [Mesh] pois a árvore do termo inclui caplan syndrome, felty syndrome) |
|  | [Spondylarthritis - MeSH - NCBI “nih.gov)](https://www.ncbi.nlm.nih.gov/mesh/68025241)  Inflammation of the joints of the SPINE, the intervertebral articulations. | - Spondylarthritides - Spinal Arthritis - Spinal Arthritides | "[Spondylarthritis](https://www.ncbi.nlm.nih.gov/mesh/68025241)" [Mesh] OR "[Spondylarthritis](https://www.ncbi.nlm.nih.gov/mesh/68025241)" OR "Spondylarthritides" OR "Spinal Arthritis" OR "Spinal Arthritides"  [Spondylitis](https://www.ncbi.nlm.nih.gov/mesh/68013166)  Spondylarthritis  [Osteoarthritis, Spine](https://www.ncbi.nlm.nih.gov/mesh/68055013)  [Spondylarthropathies](https://www.ncbi.nlm.nih.gov/mesh/68025242)  [Arthritis, Psoriatic](https://www.ncbi.nlm.nih.gov/mesh/68015535)  [Arthritis, Reactive](https://www.ncbi.nlm.nih.gov/mesh/68016918)  [Axial Spondyloarthritis](https://www.ncbi.nlm.nih.gov/mesh/2101123) + |
|  | [Arthritis, Psoriatic - MeSH - NCBI “nih.gov)](https://www.ncbi.nlm.nih.gov/mesh/68015535)  A type of inflammatory arthritis associated with PSORIASIS, often involving the axial joints and the peripheral terminal interphalangeal joints. It is characterized by the presence of HLA-B27-associated SPONDYLARTHROPATHY, and the absence of rheumatoid factor. | - Arthritic Psoriasis - Psoriatic Arthritis - Psoriasis Arthropathica - Psoriatic Arthropathy - Psoriatic Arthropathies | "[Arthritis, Psoriatic](https://www.ncbi.nlm.nih.gov/mesh/68015535)" [Mesh] OR "[Arthritis, Psoriatic](https://www.ncbi.nlm.nih.gov/mesh/68015535)" OR "Arthritic Psoriasis" OR "Psoriatic Arthritis" OR "Psoriasis Arthropathica" OR "Psoriatic Arthropathy" OR "Psoriatic Arthropathies"  [Spondylarthritis](https://www.ncbi.nlm.nih.gov/mesh/68025241)  [Spondylarthropathies](https://www.ncbi.nlm.nih.gov/mesh/68025242)  Arthritis, Psoriatic |
|  | [Spondylitis, Ankylosing - MeSH - NCBI “nih.gov)](https://www.ncbi.nlm.nih.gov/mesh/?term=ankylosing+spondylitis)  A chronic inflammatory condition affecting the axial joints, such as the SACROILIAC JOINT and other intervertebral or costovertebral joints. It occurs predominantly in young males and is characterized by pain and stiffness of joints “ANKYLOSIS) with inflammation at tendon insertions. | - Bechterew's Disease - Bechterews Disease - Marie Struempell Disease - Spondylarthritis Ankylopoietica - Spondyloarthritis Ankylopoietica - Ankylosing Spondylitis - Ankylosing Spondylarthritis - Ankylosing Spondylarthritides - Ankylosing Spondyloarthritis - Ankylosing Spondyloarthritides - Spondylitis Ankylopoietica - Bechterew Disease - Rheumatoid Spondylitis | "[Spondylitis, Ankylosing](https://www.ncbi.nlm.nih.gov/mesh/?term=ankylosing+spondylitis)" [Mesh] OR "[Spondylitis, Ankylosing](https://www.ncbi.nlm.nih.gov/mesh/?term=ankylosing+spondylitis)" OR "Bechterew's Disease" OR "Bechterews Disease" OR "Marie Struempell Disease" OR "Spondylarthritis Ankylopoietica" OR "Spondyloarthritis Ankylopoietica" OR "Ankylosing Spondylitis" OR "Ankylosing Spondylarthritis" OR "Ankylosing Spondylarthritides" OR "Ankylosing Spondyloarthritis" OR "Ankylosing Spondyloarthritides" OR "Spondylitis Ankylopoietica" OR "Bechterew Disease" OR "Rheumatoid Spondylitis"  [Spondylarthritis](https://www.ncbi.nlm.nih.gov/mesh/68025241)  [Spondylarthropathies](https://www.ncbi.nlm.nih.gov/mesh/68025242)  [Axial Spondyloarthritis](https://www.ncbi.nlm.nih.gov/mesh/2101123)  Spondylitis, Ankylosing |
|  | [Lupus Erythematosus, Systemic - MeSH - NCBI “nih.gov)](https://www.ncbi.nlm.nih.gov/mesh/68008180)  A chronic, relapsing, inflammatory, and often febrile multisystemic disorder of connective tissue, characterized principally by involvement of the skin, joints, kidneys, and serosal membranes. It is of unknown etiology, but is thought to represent a failure of the regulatory mechanisms of the autoimmune system. The disease is marked by a wide range of system dysfunctions, an elevated erythrocyte sedimentation rate, and the formation of LE cells in the blood or bone marrow. | - Systemic Lupus Erythematosus - Lupus Erythematosus Disseminatus - Libman Sacks Disease | "[Lupus Erythematosus, Systemic](https://www.ncbi.nlm.nih.gov/mesh/68008180)" [Mesh] OR "[Lupus Erythematosus, Systemic](https://www.ncbi.nlm.nih.gov/mesh/68008180)" OR "Systemic Lupus Erythematosus " OR "Lupus Erythematosus Disseminatus" OR "Libman Sacks Disease"  [Connective Tissue Diseases](https://www.ncbi.nlm.nih.gov/mesh/68003240)  Lupus Erythematosus, Systemic  [Lupus Nephritis](https://www.ncbi.nlm.nih.gov/mesh/68008181)  [Lupus Vasculitis, Central Nervous System](https://www.ncbi.nlm.nih.gov/mesh/68020945) |
|  | [Myositis - MeSH - NCBI “nih.gov)](https://www.ncbi.nlm.nih.gov/mesh/?term=inflammatory+myopathies)  Inflammation of a muscle or muscle tissue. | - Myositides - Inflammatory Muscle Diseases - Inflammatory Muscle Disease - Inflammatory Myopathy - Inflammatory Myopathies - Proliferative Myositides - Proliferative Myositis - Infectious Myositides - Infectious Myositis - Idiopathic Inflammatory Myopathies - Idiopathic Inflammatory Myopathy - Idiopathic Inflammatory Myositis - Focal Myositides - Focal Myositis | "[Myositis](https://www.ncbi.nlm.nih.gov/mesh/?term=inflammatory+myopathies)" [Mesh] OR "[Myositis](https://www.ncbi.nlm.nih.gov/mesh/?term=inflammatory+myopathies)" OR "Myositides" OR "Inflammatory Muscle Diseases" OR "Inflammatory Muscle Disease" OR "Inflammatory Myopathy" OR "Inflammatory Myopathies" OR "Proliferative Myositides" OR "Proliferative Myositis" OR "Infectious Myositides" OR "Infectious Myositis" OR "Idiopathic Inflammatory Myopathies" OR "Idiopathic Inflammatory Myopathy" OR "Idiopathic Inflammatory Myositis" OR "Focal Myositides" OR "Focal Myositis"  [Muscular Diseases](https://www.ncbi.nlm.nih.gov/mesh/68009135)  Myositis  [Myositis Ossificans](https://www.ncbi.nlm.nih.gov/mesh/68009221)  [Myositis, Inclusion Body](https://www.ncbi.nlm.nih.gov/mesh/68018979)  [Orbital Myositis](https://www.ncbi.nlm.nih.gov/mesh/68055622)  [Polymyositis](https://www.ncbi.nlm.nih.gov/mesh/68017285)  [Dermatomyositis](https://www.ncbi.nlm.nih.gov/mesh/68003882)  [Pyomyositis](https://www.ncbi.nlm.nih.gov/mesh/68052880) |
|  | [Systemic Vasculitis - MeSH - NCBI “nih.gov)](https://www.ncbi.nlm.nih.gov/mesh/?term=systemic+vasculitides)  A heterogeneous group of diseases characterized by inflammation and necrosis of the blood vessel walls. | - Systemic Vasculitides | "[Systemic Vasculitis](https://www.ncbi.nlm.nih.gov/mesh/?term=systemic+vasculitides)" [Mesh] OR "[Systemic Vasculitis](https://www.ncbi.nlm.nih.gov/mesh/?term=systemic+vasculitides)" OR "Systemic Vasculitides"  [Vasculitis](https://www.ncbi.nlm.nih.gov/mesh/68014657)  Systemic Vasculitis  [Anti-Neutrophil Cytoplasmic Antibody-Associated Vasculitis](https://www.ncbi.nlm.nih.gov/mesh/68056648)  [Churg-Strauss Syndrome](https://www.ncbi.nlm.nih.gov/mesh/68015267)  [Granulomatosis with Polyangiitis](https://www.ncbi.nlm.nih.gov/mesh/68014890)  [Microscopic Polyangiitis](https://www.ncbi.nlm.nih.gov/mesh/68055953)  [Polyarteritis Nodosa](https://www.ncbi.nlm.nih.gov/mesh/68010488)  [Rheumatoid Vasculitis](https://www.ncbi.nlm.nih.gov/mesh/68056653) |
|  | [Sjogren's Syndrome - MeSH - NCBI “nih.gov)](https://www.ncbi.nlm.nih.gov/mesh/68012859)  Chronic inflammatory and autoimmune disease in which the salivary and lacrimal glands undergo progressive destruction by lymphocytes and plasma cells resulting in decreased production of saliva and tears. The primary form, often called sicca syndrome, involves both KERATOCONJUNCTIVITIS SICCA and XEROSTOMIA. The secondary form includes, in addition, the presence of a connective tissue disease, usually rheumatoid arthritis. | - Sjogrens Syndrome - Sjogren Syndrome - Sicca Syndrome | "[Sjogren's Syndrome](https://www.ncbi.nlm.nih.gov/mesh/68012859)" [Mesh] OR "[Sjogren's Syndrome](https://www.ncbi.nlm.nih.gov/mesh/68012859)"  OR "Sjogrens Syndrome" OR "Sjogren Syndrome" OR "Sicca Syndrome"  [Arthritis](https://www.ncbi.nlm.nih.gov/mesh/68001168)  [Arthritis, Rheumatoid](https://www.ncbi.nlm.nih.gov/mesh/68001172)  Sjogren's Syndrome |
|  | [Scleroderma, Systemic - MeSH - NCBI “nih.gov)](https://www.ncbi.nlm.nih.gov/mesh/68012595)  A chronic multi-system disorder of CONNECTIVE TISSUE. It is characterized by SCLEROSIS in the SKIN, the LUNGS, the HEART, the GASTROINTESTINAL TRACT, the KIDNEYS, and the MUSCULOSKELETAL SYSTEM. Other important features include diseased small BLOOD VESSELS and AUTOANTIBODIES. The disorder is named for its most prominent feature “hard skin), and classified into subsets by the extent of skin thickening: LIMITED SCLERODERMA and DIFFUSE SCLERODERMA. | - Systemic Sclerosis - Systemic Scleroderma | "[Scleroderma, Systemic](https://www.ncbi.nlm.nih.gov/mesh/68012595)" [Mesh] OR "[Scleroderma, Systemic](https://www.ncbi.nlm.nih.gov/mesh/68012595)" OR "Systemic Sclerosis" OR "Systemic Scleroderma"  [Connective Tissue Diseases](https://www.ncbi.nlm.nih.gov/mesh/68003240)  Scleroderma, Systemic  [Scleroderma, Diffuse](https://www.ncbi.nlm.nih.gov/mesh/68045743)  [Scleroderma, Limited](https://www.ncbi.nlm.nih.gov/mesh/68045745)  [CREST Syndrome](https://www.ncbi.nlm.nih.gov/mesh/68017675) |
|  | [Mixed Connective Tissue Disease - MeSH - NCBI “nih.gov)](https://www.ncbi.nlm.nih.gov/mesh/?term=Mixed+connective+tissue+disease)  A syndrome with overlapping clinical features of systemic lupus erythematosus, scleroderma, polymyositis, and Raynaud's phenomenon. The disease is differentially characterized by high serum titers of antibodies to ribonuclease-sensitive extractable “saline soluble) nuclear antigen and a "speckled" epidermal nuclear staining pattern on direct immunofluorescence. | - Sharp Syndrome - MCTD | "[Mixed Connective Tissue Disease](https://www.ncbi.nlm.nih.gov/mesh/?term=Mixed+connective+tissue+disease)" [Mesh] OR "[Mixed Connective Tissue Disease](https://www.ncbi.nlm.nih.gov/mesh/?term=Mixed+connective+tissue+disease)" OR "Sharp Syndrome" OR "MCTD"  [Connective Tissue Diseases](https://www.ncbi.nlm.nih.gov/mesh/68003240)  Mixed Connective Tissue Disease |
| Intervention corticosteroids use | [Prednisolone - MeSH - NCBI “nih.gov)](https://www.ncbi.nlm.nih.gov/mesh/68011239)  A glucocorticoid with the general properties of the corticosteroids. It is the drug of choice for all conditions in which routine systemic corticosteroid therapy is indicated, except adrenal deficiency states. | - Predate - Predonine - Di Adreson F | "Prednisolone" [Mesh] OR "Prednisolone" OR " Predate" OR "Predonine" OR "Di Adreson F"  Steroids  Pregnanes  Pregnadienes  Pregnadienetriols  Prednisolone  Fluprednisolone  Methylprednisolone  Methylprednisolone Acetate  Methylprednisolone Hemisuccinate  Prednimustine |
|  | [Methylprednisolone - MeSH - NCBI “nih.gov)](https://www.ncbi.nlm.nih.gov/mesh/68008775)  A PREDNISOLONE derivative with similar anti-inflammatory action. | - Metipred - 6 Methylprednisolone - Urbason - Medrol | "Methylprednisolone" [Mesh] OR "Metipred" OR "6 Methylprednisolone" OR "Urbason" OR "Medrol"  [Pregnadienetriols](https://www.ncbi.nlm.nih.gov/mesh/68011246)  [Prednisolone](https://www.ncbi.nlm.nih.gov/mesh/68011239)  Methylprednisolone  [Methylprednisolone Acetate](https://www.ncbi.nlm.nih.gov/mesh/2028069) |
|  | [Glucocorticoids - MeSH - NCBI “nih.gov)](https://www.ncbi.nlm.nih.gov/mesh/68005938)  A group of CORTICOSTEROIDS that affect carbohydrate metabolism “GLUCONEOGENESIS, liver glycogen deposition, elevation of BLOOD SUGAR), inhibit ADRENOCORTICOTROPIC HORMONE secretion, and possess pronounced anti-inflammatory activity. They also play a role in fat and protein metabolism, maintenance of arterial blood pressure, alteration of the connective tissue response to injury, reduction in the number of circulating lymphocytes, and functioning of the central nervous system. | - Glucocorticoid - Glucocorticoid Effect - Glucorticoid Effects | "Glucocorticoids" [Mesh] OR "Glucocorticoids" OR "Glucocorticoid" OR "Glucocorticoid Effect" OR "Glucorticoid Effects"  [Hormones](https://www.ncbi.nlm.nih.gov/mesh/68006728)  [Adrenal Cortex Hormones](https://www.ncbi.nlm.nih.gov/mesh/68000305)  Glucocorticoids |
|  | [Dexamethasone - MeSH - NCBI “nih.gov)](https://www.ncbi.nlm.nih.gov/mesh/68003907)  An anti-inflammatory 9-fluoro-glucocorticoid. | - Methylfluorprednisolone - Hexadecadrol - Decameth - Decaspray - Dexasone - Dexpak - Maxidex - Millicorten - Oradexon - Decaject - Decaject L.A. - Hexadrol | "Dexamethasone" [Mesh] OR "Dexamethasone" OR "Methylfluorprednisolone" OR "Hexadecadrol" OR "Decameth" OR "Decaspray" OR "Dexasone" OR "Dexpak" OR"Maxidex" OR "Millicorten" OR "Oradexon" OR "Decaject" OR "Decaject L.A. " OR "Hexadrol"  Dexamethasone  [Dexamethasone Isonicotinate](https://www.ncbi.nlm.nih.gov/mesh/68003908)  [Tobramycin, Dexamethasone Drug Combination](https://www.ncbi.nlm.nih.gov/mesh/2028175) |
|  | cc [- MeSH - NCBI “nih.gov)](https://www.ncbi.nlm.nih.gov/mesh/?term=Pregnadienes)  Pregnane derivatives containing two double bonds anywhere within the ring structures. | - | "Pregnadienes" [Mesh] OR "Pregnadienes"  [Steroids](https://www.ncbi.nlm.nih.gov/mesh/68013256)  [Pregnanes](https://www.ncbi.nlm.nih.gov/mesh/68011278)  Pregnadienes  [Canrenoic Acid](https://www.ncbi.nlm.nih.gov/mesh/68002191)  [Canrenone](https://www.ncbi.nlm.nih.gov/mesh/68002192)  [Chlormadinone Acetate](https://www.ncbi.nlm.nih.gov/mesh/68002715)  [Cyproterone](https://www.ncbi.nlm.nih.gov/mesh/68003534)  [Cyproterone Acetate](https://www.ncbi.nlm.nih.gov/mesh/68017373)  [Danazol](https://www.ncbi.nlm.nih.gov/mesh/68003613)  [Dydrogesterone](https://www.ncbi.nlm.nih.gov/mesh/68004394)  [Fluocinolone Acetonide](https://www.ncbi.nlm.nih.gov/mesh/68005446)  [Fluocinonide](https://www.ncbi.nlm.nih.gov/mesh/68005447)  [Medrogestone](https://www.ncbi.nlm.nih.gov/mesh/68008524)  [Megestrol](https://www.ncbi.nlm.nih.gov/mesh/68008535)  [Megestrol Acetate](https://www.ncbi.nlm.nih.gov/mesh/68019290)  [Melengestrol Acetate](https://www.ncbi.nlm.nih.gov/mesh/68008552)  [Pregnadienediols](https://www.ncbi.nlm.nih.gov/mesh/68011244)  [Desoximetasone](https://www.ncbi.nlm.nih.gov/mesh/68003899)  [Fluocortolone](https://www.ncbi.nlm.nih.gov/mesh/68005448) +  [Fluorometholone](https://www.ncbi.nlm.nih.gov/mesh/68005469)  [Mometasone Furoate](https://www.ncbi.nlm.nih.gov/mesh/2009858) +  [Prednisone](https://www.ncbi.nlm.nih.gov/mesh/68011241)  [Pregnadienetriols](https://www.ncbi.nlm.nih.gov/mesh/68011246)  [Beclomethasone](https://www.ncbi.nlm.nih.gov/mesh/68001507)  [Betamethasone](https://www.ncbi.nlm.nih.gov/mesh/68001623) +  [Desonide](https://www.ncbi.nlm.nih.gov/mesh/68003898)  [Dexamethasone](https://www.ncbi.nlm.nih.gov/mesh/68003907) +  [Flumethasone](https://www.ncbi.nlm.nih.gov/mesh/68005443)  [Paramethasone](https://www.ncbi.nlm.nih.gov/mesh/68010248)  [Prednisolone](https://www.ncbi.nlm.nih.gov/mesh/68011239) +  [Triamcinolone](https://www.ncbi.nlm.nih.gov/mesh/68014221)  [Triamcinolone Acetonide](https://www.ncbi.nlm.nih.gov/mesh/68014222) |
|  | [Prednisone - MeSH - NCBI “nih.gov)](https://www.ncbi.nlm.nih.gov/mesh/68011241) | - Dehydrocortisone - delta-Cortisone - Rectodelt - Prednison Hexal - Sterapred - Ultracorten - Winpred - Apo-Prednisone - Cortan - Cortancyl - Panafcort - Cutason - Decortin - Dacortin - Decortisyl - Deltasone - Encortone - Encorton - Enkortolon - Kortancyl - Liquid Pred - Meticorten - Orasone - Panasol - Predni Tablinen - Prednidib - Predniment - Prednison Acsis - Pronisone - Sone - Prednison Galen | "Prednisone" [Mesh] OR "Prednisone" OR "Dehydrocortisone" OR "delta-Cortisone" OR "Rectodelt" OR "Prednison Hexal" OR "Sterapred" OR "Ultracorten" OR "Winpred" OR "Apo-Prednisone" OR "Cortan" OR "Cortancyl" OR"Panafcort" OR "Cutason" OR "Decortin" OR "Dacortin" OR "Decortisyl" OR "Deltasone" OR "Encortone" OR "Encorton" OR "Enkortolon" OR "Kortancyl" OR "Liquid Pred" OR "Meticorten" OR "Orasone" OR "Panasol" OR "Predni Tablinen" OR "Prednidib" OR "Predniment" OR "Prednison Acsis" OR "Pronisone" OR "Sone" OR "Prednison Galen"  [Steroids](https://www.ncbi.nlm.nih.gov/mesh/68013256)  [Pregnanes](https://www.ncbi.nlm.nih.gov/mesh/68011278)  [Pregnadienes](https://www.ncbi.nlm.nih.gov/mesh/68011245)  [Pregnadienediols](https://www.ncbi.nlm.nih.gov/mesh/68011244)  Prednisone |
| Outcomes  Vaccination with inactivated vaccine | [Vaccines, Inactivated - MeSH - NCBI “nih.gov)](https://www.ncbi.nlm.nih.gov/mesh/68015164)  Definition: Vaccines in which the infectious microbial nucleic acid components have been destroyed by chemical or physical treatment “e.g., formalin, beta-propiolactone, gamma radiation) without affecting the antigenicity or immunogenicity of the viral coat or bacterial outer membrane proteins. | Inactivated Vaccines  Killed Vaccine  Killed Vaccines  Inactivated Vaccine | "[Vaccines, Inactivated](https://www.ncbi.nlm.nih.gov/mesh/68015164)" [Mesh] OR "[Vaccines, Inactivated](https://www.ncbi.nlm.nih.gov/mesh/68015164)" OR "Inactivated Vaccines" OR "Killed Vaccine" OR "Killed Vaccines" OR "Inactivated Vaccine"  Vaccines, Inactivated  [Poliovirus Vaccine, Inactivated](https://www.ncbi.nlm.nih.gov/mesh/68011054) |
|  | [Influenza Vaccines - MeSH - NCBI “nih.gov)](https://www.ncbi.nlm.nih.gov/mesh/68007252)  Vaccines used to prevent infection by viruses in the family ORTHOMYXOVIRIDAE. It includes both killed and attenuated vaccines. The composition of the vaccines is changed each year in response to antigenic shifts and changes in prevalence of influenza virus strains. The flu vaccines may be mono- or multi-valent, which contains one or more INFLUENZAVIRUS A and INFLUENZAVIRUS B strains. | - Influenza Vaccine - Influenzavirus Vaccines - Influenza Virus Vaccines - Influenzavirus Vaccine - Influenza Virus Vaccine - High-Dose Trivalent Influenza Vaccine - High Dose Trivalent Influenza Vaccine - Trivalent Influenza Vaccine - Quadrivalent Influenza Vaccine - High dose | "[Influenza Vaccines](https://www.ncbi.nlm.nih.gov/mesh/68007252)"[Mesh:NoExp]  OR Influenza Vaccine" OR "Influenzavirus Vaccines" OR "Influenza Virus Vaccines" OR "Influenzavirus Vaccine" OR "Influenza Virus Vaccine" OR "High-Dose Trivalent Influenza Vaccine" OR "High Dose Trivalent Influenza Vaccine" OR "Trivalent Influenza Vaccine" OR "Quadrivalent Influenza Vaccine"  “não utilizar [Mesh] pois a árvore do termo inclui VVA e intra-nasal)  [Vaccines](https://www.ncbi.nlm.nih.gov/mesh/68014612)  [Viral Vaccines](https://www.ncbi.nlm.nih.gov/mesh/68014765)  Influenza Vaccines |
|  | [Pneumococcal Vaccines - MeSH - NCBI “nih.gov)](https://www.ncbi.nlm.nih.gov/mesh/68022242)  Vaccines or candidate vaccines used to prevent infections with STREPTOCOCCUS PNEUMONIAE. | - Pneumococcal Vaccine - Pneumovax - Pneumococcal Polysaccharide Vaccine - Pnu Imune Vaccine - PnuImune Vaccine - Conjugated penumococcal vaccine | "[Pneumococcal Vaccines](https://www.ncbi.nlm.nih.gov/mesh/68022242)" [Mesh] OR "[Pneumococcal Vaccines](https://www.ncbi.nlm.nih.gov/mesh/68022242)" OR "Pneumococcal Vaccine" OR "Pneumovax" OR "Pneumococcal Polysaccharide Vaccine" OR "Pnu Imune Vaccine" OR "PnuImune Vaccine" OR "Conjugated penumococcal vaccine"  Pneumococcal Vaccines  [Heptavalent Pneumococcal Conjugate Vaccine](https://www.ncbi.nlm.nih.gov/mesh/2009980)  13 pneumococcal conjugate vaccine |
|  | [Herpes Zoster Vaccine - MeSH - NCBI “nih.gov)](https://www.ncbi.nlm.nih.gov/mesh/68053061)  An attenuated vaccine used to prevent and/or treat HERPES ZOSTER, a disease caused by HUMAN HERPESVIRUS 3. | - Zoster Vaccine - Shingles Vaccine | "Herpes Zoster Vaccine" [Mesh:NoExp] OR " Zoster Vaccine" OR "Shingles Vaccine"  “não utilizar [Mesh] pois a árvore do termo inclui live attenuated zoster  e intra-nasal)  [Vaccines](https://www.ncbi.nlm.nih.gov/mesh/68014612)  [Viral Vaccines](https://www.ncbi.nlm.nih.gov/mesh/68014765)  [Herpesvirus Vaccines](https://www.ncbi.nlm.nih.gov/mesh/68022283)  Herpes Zoster Vaccine |
|  | [Hepatitis B Vaccines - MeSH - NCBI “nih.gov)](https://www.ncbi.nlm.nih.gov/mesh/68017325)  Vaccines or candidate vaccines containing inactivated hepatitis B or some of its component antigens and designed to prevent hepatitis B. Some vaccines may be recombinantly produced. | Hepatitis B Vaccine | "Hepatitis B Vaccines" [Mesh] OR "Hepatitis B Vaccines" OR "Hepatitis B Vaccine"  [Vaccines](https://www.ncbi.nlm.nih.gov/mesh/68014612)  [Viral Vaccines](https://www.ncbi.nlm.nih.gov/mesh/68014765)  [Viral Hepatitis Vaccines](https://www.ncbi.nlm.nih.gov/mesh/68014761)  Hepatitis B Vaccines |
|  | [Papillomavirus Vaccines - MeSH - NCBI “nih.gov)](https://www.ncbi.nlm.nih.gov/mesh/?term=papillomavirus+vaccines)   Vaccines or candidate vaccines used to prevent PAPILLOMAVIRUS INFECTIONS. Human vaccines are intended to reduce the incidence of UTERINE CERVICAL NEOPLASMS, so they are sometimes considered a type of CANCER VACCINES. They are often composed of CAPSID PROTEINS, especially L1 protein, from various types of ALPHAPAPILLOMAVIRUS | - Papillomavirus Vaccine   Human Papillomavirus Vaccines   - HPV Vaccine   Human Papilloma Virus Vaccine   - HPV Vaccines - Human Papilloma Virus Vaccines - Human Papillomavirus Vaccine | "Papillomavirus Vaccines" [Mesh] OR "Papillomavirus Vaccines" OR "Papillomavirus Vaccine" OR "Human Papillomavirus Vaccines" OR "HPV Vaccine" OR "Human Papilloma Virus Vaccine" OR "HPV Vaccines" OR "Human Papilloma Virus Vaccines" OR "Human Papillomavirus Vaccine"  [Vaccines](https://www.ncbi.nlm.nih.gov/mesh/68014612)  [Viral Vaccines](https://www.ncbi.nlm.nih.gov/mesh/68014765)  Papillomavirus Vaccines  [Human Papillomavirus](https://www.ncbi.nlm.nih.gov/mesh/2009889) |
|  | [Poliovirus Vaccines - MeSH - NCBI “nih.gov)](https://www.ncbi.nlm.nih.gov/mesh/68023321)    Vaccines used to prevent POLIOMYELITIS. They include inactivated “POLIOVIRUS VACCINE, INACTIVATED) | - Poliovirus Vaccine - salk | "Poliovirus Vaccines" [Mesh] OR "Poliovirus Vaccines" OR "Poliovirus Vaccine" OR "salk"  [Vaccines](https://www.ncbi.nlm.nih.gov/mesh/68014612)  [Viral Vaccines](https://www.ncbi.nlm.nih.gov/mesh/68014765)  [Poliovirus Vaccine, Inactivated](https://www.ncbi.nlm.nih.gov/mesh/68011054) |
|  | [Hepatitis A Vaccines - MeSH - NCBI “nih.gov)](https://www.ncbi.nlm.nih.gov/mesh/68022362)    Vaccines or candidate vaccines used to prevent infection with hepatitis A virus “HEPATOVIRUS). | - Hepatovirus Vaccines - Hepatitis A Vaccine - Vaqta - Havrix | "Hepatitis A Vaccines" [Mesh] OR "Hepatitis A Vaccines" OR "Hepatovirus Vaccines" OR "Hepatitis A Vaccine" OR "Vaqta" OR "Havrix"  [Vaccines](https://www.ncbi.nlm.nih.gov/mesh/68014612)  [Viral Hepatitis Vaccines](https://www.ncbi.nlm.nih.gov/mesh/68014761)  Hepatitis A Vaccines |
|  | [Meningococcal Vaccines - MeSH - NCBI “nih.gov)](https://www.ncbi.nlm.nih.gov/mesh/68022401)    Vaccines or candidate vaccines used to prevent infection with NEISSERIA MENINGITIDIS. | Meningococcal Vaccine  Menactra  Tetravalent Meningococcal Vaccine  Menomune  Menveo  Bivalent Meningococcal Vaccine  Meningococcal Polysaccharide Vaccine | "Meningococcal Vaccines" [Mesh] OR " Meningococcal Vaccine" OR "Menactra" OR "Tetravalent Meningococcal Vaccine" OR "Menomune OR "Menveo" OR "Bivalent Meningococcal Vaccine" OR "Meningococcal Polysaccharide Vaccine"    [Vaccines](https://www.ncbi.nlm.nih.gov/mesh/68014612)  Meningococcal Vaccines C, B or ACWY |
|  | [Diphtheria-Tetanus-Pertussis Vaccine - MeSH - NCBI “nih.gov)](https://www.ncbi.nlm.nih.gov/mesh/?term=vaccine+per)   A vaccine consisting of DIPHTHERIA TOXOID; TETANUS TOXOID; and whole-cell PERTUSSIS VACCINE. The vaccine protects against diphtheria, tetanus, and whooping cough. | Diphtheria Tetanus Pertussis Vaccine  DTwP Vaccine  DPT Vaccine  DTP Vaccine  Di Te Per Vaccine  Diphtheria Pertussis Tetanus Vaccine | "Diphtheria-Tetanus-Pertussis Vaccine" [Mesh] OR "Diphtheria-Tetanus-Pertussis Vaccine" OR " Diphtheria Tetanus Pertussis Vaccine" OR "DTwP Vaccine" OR "DPT Vaccine" OR "DTP Vaccine" OR "Di Te Per Vaccine" OR "Diphtheria Pertussis Tetanus Vaccine"  [Vaccines](https://www.ncbi.nlm.nih.gov/mesh/68014612)  [Pertussis Vaccine](https://www.ncbi.nlm.nih.gov/mesh/68010567)  Diphtheria-Tetanus-Pertussis Vaccine |
|  | [Haemophilus influenza type b polysaccharide vaccine-tetanus toxin conj - MeSH - NCBI “nih.gov)](https://www.ncbi.nlm.nih.gov/mesh/?term=haemophilus+influenza+type+b+polysaccharide+vaccine+tetanus+toxin+conjugate)    the type b capsular polysaccharide of HIB-vaccine is conjugated to tetanus toxoid | HIBest  PRP-T  Hib-TT  Hib capsular polysaccharide-tetanus vaccine  Hib CPS-tetanus vaccine  OmniHIB  ActHIB | "Haemophilus influenza type b polysaccharide vaccine-tetanus toxin conjugate" OR "HIBest" OR "PRP-T" OR "Hib-TT" OR "Hib capsular polysaccharide-tetanus vaccine" OR "Hib CPS-tetanus vaccine" OR "OmniHIB" OR "ActHIB" |

**Question 08.** What is the impact of JAKis use on the response to inactivated vaccine in patients with immune-mediated rheumatic diseases ?

| Group | **MeSH Terms**  and definitions | **Entry Terms** | **Search Key** |
| --- | --- | --- | --- |
| Population  Patients with immune-mediated rheumatic diseases “IMRD) | [Rheumatic Diseases - MeSH - NCBI “nih.gov)](https://www.ncbi.nlm.nih.gov/mesh/68012216)  Disorders of connective tissue, especially the joints and related structures, characterized by inflammation, degeneration, or metabolic derangement. | Rheumatic Disease  Rheumatism | "[Rheumatic Diseases](https://www.ncbi.nlm.nih.gov/mesh/68012216)" [Mesh:NoExp] OR "Rheumatic Disease" OR "Rheumatism" OR  "Immune-mediated rheumatic diseases"  “não utilizar [Mesh] pois a árvore do termo inclui gota, fibromialgia e crianças) |
|  | [Arthritis, Rheumatoid - MeSH - NCBI “nih.gov)](https://www.ncbi.nlm.nih.gov/mesh/68001172)  A chronic systemic disease, primarily of the joints, marked by inflammatory changes in the synovial membranes and articular structures, widespread fibrinoid degeneration of the collagen fibers in mesenchymal tissues, and by atrophy and rarefaction of bony structures. Etiology is unknown, but autoimmune mechanisms have been implicated. | - Rheumatoid Arthritis | "[Arthritis, Rheumatoid](https://www.ncbi.nlm.nih.gov/mesh/68001172)" [Mesh:NoExp] OR "Rheumatoid Arthritis" OR "[Rheumatoid Nodule](https://www.ncbi.nlm.nih.gov/mesh/68012218)" OR "[Rheumatoid Vasculitis](https://www.ncbi.nlm.nih.gov/mesh/68056653)" OR "[Sjogren's Syndrome](https://www.ncbi.nlm.nih.gov/mesh/68012859)"  “não utilizar [Mesh] pois a árvore do termo inclui caplan syndrome, felty syndrome) |
|  | [Spondylarthritis - MeSH - NCBI “nih.gov)](https://www.ncbi.nlm.nih.gov/mesh/68025241)  Inflammation of the joints of the SPINE, the intervertebral articulations. | - Spondylarthritides - Spinal Arthritis - Spinal Arthritides | "[Spondylarthritis](https://www.ncbi.nlm.nih.gov/mesh/68025241)" [Mesh] OR "[Spondylarthritis](https://www.ncbi.nlm.nih.gov/mesh/68025241)" OR "Spondylarthritides" OR "Spinal Arthritis" OR "Spinal Arthritides"  [Spondylitis](https://www.ncbi.nlm.nih.gov/mesh/68013166)  Spondylarthritis  [Osteoarthritis, Spine](https://www.ncbi.nlm.nih.gov/mesh/68055013)  [Spondylarthropathies](https://www.ncbi.nlm.nih.gov/mesh/68025242)  [Arthritis, Psoriatic](https://www.ncbi.nlm.nih.gov/mesh/68015535)  [Arthritis, Reactive](https://www.ncbi.nlm.nih.gov/mesh/68016918)  [Axial Spondyloarthritis](https://www.ncbi.nlm.nih.gov/mesh/2101123) + |
|  | [Arthritis, Psoriatic - MeSH - NCBI “nih.gov)](https://www.ncbi.nlm.nih.gov/mesh/68015535)  A type of inflammatory arthritis associated with PSORIASIS, often involving the axial joints and the peripheral terminal interphalangeal joints. It is characterized by the presence of HLA-B27-associated SPONDYLARTHROPATHY, and the absence of rheumatoid factor. | - Arthritic Psoriasis - Psoriatic Arthritis - Psoriasis Arthropathica - Psoriatic Arthropathy - Psoriatic Arthropathies | "[Arthritis, Psoriatic](https://www.ncbi.nlm.nih.gov/mesh/68015535)" [Mesh] OR "[Arthritis, Psoriatic](https://www.ncbi.nlm.nih.gov/mesh/68015535)" OR "Arthritic Psoriasis" OR "Psoriatic Arthritis" OR "Psoriasis Arthropathica" OR "Psoriatic Arthropathy" OR "Psoriatic Arthropathies"  [Spondylarthritis](https://www.ncbi.nlm.nih.gov/mesh/68025241)  [Spondylarthropathies](https://www.ncbi.nlm.nih.gov/mesh/68025242)  Arthritis, Psoriatic |
|  | [Spondylitis, Ankylosing - MeSH - NCBI “nih.gov)](https://www.ncbi.nlm.nih.gov/mesh/?term=ankylosing+spondylitis)  A chronic inflammatory condition affecting the axial joints, such as the SACROILIAC JOINT and other intervertebral or costovertebral joints. It occurs predominantly in young males and is characterized by pain and stiffness of joints “ANKYLOSIS) with inflammation at tendon insertions. | - Bechterew's Disease - Bechterews Disease - Marie Struempell Disease - Spondylarthritis Ankylopoietica - Spondyloarthritis Ankylopoietica - Ankylosing Spondylitis - Ankylosing Spondylarthritis - Ankylosing Spondylarthritides - Ankylosing Spondyloarthritis - Ankylosing Spondyloarthritides - Spondylitis Ankylopoietica - Bechterew Disease - Rheumatoid Spondylitis | "[Spondylitis, Ankylosing](https://www.ncbi.nlm.nih.gov/mesh/?term=ankylosing+spondylitis)" [Mesh] OR "[Spondylitis, Ankylosing](https://www.ncbi.nlm.nih.gov/mesh/?term=ankylosing+spondylitis)" OR "Bechterew's Disease" OR "Bechterews Disease" OR "Marie Struempell Disease" OR "Spondylarthritis Ankylopoietica" OR "Spondyloarthritis Ankylopoietica" OR "Ankylosing Spondylitis" OR "Ankylosing Spondylarthritis" OR "Ankylosing Spondylarthritides" OR "Ankylosing Spondyloarthritis" OR "Ankylosing Spondyloarthritides" OR "Spondylitis Ankylopoietica" OR "Bechterew Disease" OR "Rheumatoid Spondylitis"  [Spondylarthritis](https://www.ncbi.nlm.nih.gov/mesh/68025241)  [Spondylarthropathies](https://www.ncbi.nlm.nih.gov/mesh/68025242)  [Axial Spondyloarthritis](https://www.ncbi.nlm.nih.gov/mesh/2101123)  Spondylitis, Ankylosing |
|  | [Lupus Erythematosus, Systemic - MeSH - NCBI “nih.gov)](https://www.ncbi.nlm.nih.gov/mesh/68008180)  A chronic, relapsing, inflammatory, and often febrile multisystemic disorder of connective tissue, characterized principally by involvement of the skin, joints, kidneys, and serosal membranes. It is of unknown etiology, but is thought to represent a failure of the regulatory mechanisms of the autoimmune system. The disease is marked by a wide range of system dysfunctions, an elevated erythrocyte sedimentation rate, and the formation of LE cells in the blood or bone marrow. | - Systemic Lupus Erythematosus - Lupus Erythematosus Disseminatus - Libman Sacks Disease | "[Lupus Erythematosus, Systemic](https://www.ncbi.nlm.nih.gov/mesh/68008180)" [Mesh] OR "[Lupus Erythematosus, Systemic](https://www.ncbi.nlm.nih.gov/mesh/68008180)" OR "Systemic Lupus Erythematosus " OR "Lupus Erythematosus Disseminatus" OR "Libman Sacks Disease"  [Connective Tissue Diseases](https://www.ncbi.nlm.nih.gov/mesh/68003240)  Lupus Erythematosus, Systemic  [Lupus Nephritis](https://www.ncbi.nlm.nih.gov/mesh/68008181)  [Lupus Vasculitis, Central Nervous System](https://www.ncbi.nlm.nih.gov/mesh/68020945) |
|  | [Myositis - MeSH - NCBI “nih.gov)](https://www.ncbi.nlm.nih.gov/mesh/?term=inflammatory+myopathies)  Inflammation of a muscle or muscle tissue. | - Myositides - Inflammatory Muscle Diseases - Inflammatory Muscle Disease - Inflammatory Myopathy - Inflammatory Myopathies - Proliferative Myositides - Proliferative Myositis - Infectious Myositides - Infectious Myositis - Idiopathic Inflammatory Myopathies - Idiopathic Inflammatory Myopathy - Idiopathic Inflammatory Myositis - Focal Myositides - Focal Myositis | "[Myositis](https://www.ncbi.nlm.nih.gov/mesh/?term=inflammatory+myopathies)" [Mesh] OR "[Myositis](https://www.ncbi.nlm.nih.gov/mesh/?term=inflammatory+myopathies)" OR "Myositides" OR "Inflammatory Muscle Diseases" OR "Inflammatory Muscle Disease" OR "Inflammatory Myopathy" OR "Inflammatory Myopathies" OR "Proliferative Myositides" OR "Proliferative Myositis" OR "Infectious Myositides" OR "Infectious Myositis" OR "Idiopathic Inflammatory Myopathies" OR "Idiopathic Inflammatory Myopathy" OR "Idiopathic Inflammatory Myositis" OR "Focal Myositides" OR "Focal Myositis"  [Muscular Diseases](https://www.ncbi.nlm.nih.gov/mesh/68009135)  Myositis  [Myositis Ossificans](https://www.ncbi.nlm.nih.gov/mesh/68009221)  [Myositis, Inclusion Body](https://www.ncbi.nlm.nih.gov/mesh/68018979)  [Orbital Myositis](https://www.ncbi.nlm.nih.gov/mesh/68055622)  [Polymyositis](https://www.ncbi.nlm.nih.gov/mesh/68017285)  [Dermatomyositis](https://www.ncbi.nlm.nih.gov/mesh/68003882)  [Pyomyositis](https://www.ncbi.nlm.nih.gov/mesh/68052880) |
|  | [Systemic Vasculitis - MeSH - NCBI “nih.gov)](https://www.ncbi.nlm.nih.gov/mesh/?term=systemic+vasculitides)  A heterogeneous group of diseases characterized by inflammation and necrosis of the blood vessel walls. | - Systemic Vasculitides | "[Systemic Vasculitis](https://www.ncbi.nlm.nih.gov/mesh/?term=systemic+vasculitides)" [Mesh] OR "[Systemic Vasculitis](https://www.ncbi.nlm.nih.gov/mesh/?term=systemic+vasculitides)" OR "Systemic Vasculitides"  [Vasculitis](https://www.ncbi.nlm.nih.gov/mesh/68014657)  Systemic Vasculitis  [Anti-Neutrophil Cytoplasmic Antibody-Associated Vasculitis](https://www.ncbi.nlm.nih.gov/mesh/68056648)  [Churg-Strauss Syndrome](https://www.ncbi.nlm.nih.gov/mesh/68015267)  [Granulomatosis with Polyangiitis](https://www.ncbi.nlm.nih.gov/mesh/68014890)  [Microscopic Polyangiitis](https://www.ncbi.nlm.nih.gov/mesh/68055953)  [Polyarteritis Nodosa](https://www.ncbi.nlm.nih.gov/mesh/68010488)  [Rheumatoid Vasculitis](https://www.ncbi.nlm.nih.gov/mesh/68056653) |
|  | [Sjogren's Syndrome - MeSH - NCBI “nih.gov)](https://www.ncbi.nlm.nih.gov/mesh/68012859)  Chronic inflammatory and autoimmune disease in which the salivary and lacrimal glands undergo progressive destruction by lymphocytes and plasma cells resulting in decreased production of saliva and tears. The primary form, often called sicca syndrome, involves both KERATOCONJUNCTIVITIS SICCA and XEROSTOMIA. The secondary form includes, in addition, the presence of a connective tissue disease, usually rheumatoid arthritis. | - Sjogrens Syndrome - Sjogren Syndrome - Sicca Syndrome | "[Sjogren's Syndrome](https://www.ncbi.nlm.nih.gov/mesh/68012859)" [Mesh] OR "[Sjogren's Syndrome](https://www.ncbi.nlm.nih.gov/mesh/68012859)"  OR "Sjogrens Syndrome" OR "Sjogren Syndrome" OR "Sicca Syndrome"  [Arthritis](https://www.ncbi.nlm.nih.gov/mesh/68001168)  [Arthritis, Rheumatoid](https://www.ncbi.nlm.nih.gov/mesh/68001172)  Sjogren's Syndrome |
|  | [Scleroderma, Systemic - MeSH - NCBI “nih.gov)](https://www.ncbi.nlm.nih.gov/mesh/68012595)  A chronic multi-system disorder of CONNECTIVE TISSUE. It is characterized by SCLEROSIS in the SKIN, the LUNGS, the HEART, the GASTROINTESTINAL TRACT, the KIDNEYS, and the MUSCULOSKELETAL SYSTEM. Other important features include diseased small BLOOD VESSELS and AUTOANTIBODIES. The disorder is named for its most prominent feature “hard skin), and classified into subsets by the extent of skin thickening: LIMITED SCLERODERMA and DIFFUSE SCLERODERMA. | - Systemic Sclerosis - Systemic Scleroderma | "[Scleroderma, Systemic](https://www.ncbi.nlm.nih.gov/mesh/68012595)" [Mesh] OR "[Scleroderma, Systemic](https://www.ncbi.nlm.nih.gov/mesh/68012595)" OR "Systemic Sclerosis" OR "Systemic Scleroderma"  [Connective Tissue Diseases](https://www.ncbi.nlm.nih.gov/mesh/68003240)  Scleroderma, Systemic  [Scleroderma, Diffuse](https://www.ncbi.nlm.nih.gov/mesh/68045743)  [Scleroderma, Limited](https://www.ncbi.nlm.nih.gov/mesh/68045745)  [CREST Syndrome](https://www.ncbi.nlm.nih.gov/mesh/68017675) |
|  | [Mixed Connective Tissue Disease - MeSH - NCBI “nih.gov)](https://www.ncbi.nlm.nih.gov/mesh/?term=Mixed+connective+tissue+disease)  A syndrome with overlapping clinical features of systemic lupus erythematosus, scleroderma, polymyositis, and Raynaud's phenomenon. The disease is differentially characterized by high serum titers of antibodies to ribonuclease-sensitive extractable “saline soluble) nuclear antigen and a "speckled" epidermal nuclear staining pattern on direct immunofluorescence. | - Sharp Syndrome - MCTD | "[Mixed Connective Tissue Disease](https://www.ncbi.nlm.nih.gov/mesh/?term=Mixed+connective+tissue+disease)" [Mesh] OR "[Mixed Connective Tissue Disease](https://www.ncbi.nlm.nih.gov/mesh/?term=Mixed+connective+tissue+disease)" OR "Sharp Syndrome" OR "MCTD"  [Connective Tissue Diseases](https://www.ncbi.nlm.nih.gov/mesh/68003240)  Mixed Connective Tissue Disease |
| Intervention |  |  |  |
|  | [Mycophenolic Acid - MeSH - NCBI “nih.gov)](https://www.ncbi.nlm.nih.gov/mesh/68009173)  Compound derived from Penicillium stoloniferum and related species. It blocks de novo biosynthesis of purine nucleotides by inhibition of the enzyme inosine monophosphate dehydrogenase “IMP DEHYDROGENASE). Mycophenolic acid exerts selective effects on the immune system in which it prevents the proliferation of T-CELLS, LYMPHOCYTES, and the formation of antibodies from B-CELLS. It may also inhibit recruitment of LEUKOCYTES to sites of INFLAMMATION. | - Mycophenolate Mofetil - Mycophenolic Acid Morpholinoethyl Ester - Cellcept - Mycophenolate Sodium - Sodium Mycophenolat - Myfortic - Mycophenolate Mofetil Hydrochloride - RS 61443 | "Mycophenolic Acid" [Mesh] OR "Mycophenolic Acid" OR " Mycophenolate Mofetil" OR "Mycophenolic Acid" OR "Morpholinoethyl Ester" OR "Cellcept" OR "Mycophenolate Sodium" OR "Sodium Mycophenolat" OR "Myfortic" OR "Mycophenolate Mofetil Hydrochloride" OR "RS 61443"  [Acids, Acyclic](https://www.ncbi.nlm.nih.gov/mesh/68000144)  [Caproates](https://www.ncbi.nlm.nih.gov/mesh/68002208)  Mycophenolic Acid |
|  | [Abatacept - MeSH - NCBI “nih.gov)](https://www.ncbi.nlm.nih.gov/mesh/?term=abatacept)  A fusion protein immunoconjugate of the extracellular domain of CTLA4 and the Fc domain of human IgG1. It functions as a T-cell co-stimulation blocker that inhibits TNF-ALPHA and prevents the activation of T-LYMPHOCYTES. It is used in the treatment of RHEUMATOID ARTHRITIS. | - CTLA-4-Ig - Cytotoxic T Lymphocyte-Associated Antigen 4-Immunoglobulin - Cytotoxic T Lymphocyte Associated Antigen 4 Immunoglobulin - CTLA4-Ig - CTLA4-Ig Immunoconjugate - CTLA4 Ig Immunoconjugate - BMS224818 - BMS 224818 - Belatacept - Orencia - BMS 188667 - BMS188667 - LEA29Y - Nulojix | "Abatacept" [Mesh] OR "Abatacept" OR "CTLA-4-Ig" OR "Cytotoxic T Lymphocyte-Associated Antigen 4-Immunoglobulin" OR "Cytotoxic T Lymphocyte Associated Antigen 4 Immunoglobulin" OR "CTLA4-Ig" OR "CTLA4-Ig Immunoconjugate" OR "CTLA4 Ig Immunoconjugate" OR "BMS224818" OR "BMS 224818" OR "Belatacept" OR "Orencia" OR "BMS 188667" OR "LEA29Y" OR "Nulojix"  [Antibodies](https://www.ncbi.nlm.nih.gov/mesh/68000906)  [Immunoconjugates](https://www.ncbi.nlm.nih.gov/mesh/68018796)  Abatacept |
|  | [Janus Kinase Inhibitors - MeSH - NCBI “nih.gov)](https://www.ncbi.nlm.nih.gov/mesh/2023419)  Agents that inhibit JANUS KINASES. | - JAK Inhibitors - Janus Kinase Inhibitor - JAK Inhibitor | "Janus Kinase Inhibitors" [Mesh] OR "Janus Kinase Inhibitors" OR "JAK Inhibitors" OR "Janus Kinase Inhibitor" OR "JAK Inhibitor"  [Molecular Mechanisms of Pharmacological Action](https://www.ncbi.nlm.nih.gov/mesh/68045504)  [Enzyme Inhibitors](https://www.ncbi.nlm.nih.gov/mesh/68004791)  [Protein Kinase Inhibitors](https://www.ncbi.nlm.nih.gov/mesh/68047428)  Janus Kinase Inhibitors |
|  | [upadacitinib - MeSH - NCBI “nih.gov)](https://www.ncbi.nlm.nih.gov/mesh/?term=Upadacitinib)  a JAK-1 inhibitor; for treatment of rheumatoid arthritis | - “3S,4R)-3-ethyl-4-”3H-imidazo”1,2-a)pyrrolo”2,3-e)pyrazin-8-yl)-N-”2,2,2-trifluoroethyl)-1-pyrrolidinecarboxamide - ABT-494 - Rinvoq | "upadacitinib" [Mesh] OR "upadacitinib" OR "“3S,4R)-3-ethyl-4-”3H-imidazo”1,2-a)pyrrolo”2,3-e)pyrazin-8-yl)-N-”2,2,2-trifluoroethyl)-1-pyrrolidinecarboxamide" OR "ABT-494" OR "Rinvoq" |
|  | [Protein Kinase Inhibitors - MeSH - NCBI “nih.gov)](https://www.ncbi.nlm.nih.gov/mesh/68047428)  Agents that inhibit PROTEIN KINASES. | Protein Kinase Inhibitor | "Protein Kinase Inhibitors" [Mesh] OR "Protein Kinase Inhibitors" OR " Protein Kinase Inhibitor"  [Enzyme Inhibitors](https://www.ncbi.nlm.nih.gov/mesh/68004791)  Protein Kinase Inhibitors  [Janus Kinase Inhibitors](https://www.ncbi.nlm.nih.gov/mesh/2023419)  [MTOR Inhibitors](https://www.ncbi.nlm.nih.gov/mesh/2101251)  [Tyrosine Protein Kinase Inhibitors](https://www.ncbi.nlm.nih.gov/mesh/2103139) |
|  | [tofacitinib - MeSH - NCBI “nih.gov)](https://www.ncbi.nlm.nih.gov/mesh/?term=Tofacitinib) | - tasocitinib - tofacitinib citrate - Xeljanz - CP 690550 | "tofacitinib" [Mesh] OR "tofacitinib" OR "tasocitinib" OR "tofacitinib citrate" OR "Xeljanz" OR "CP 690550" |
|  | [baricitinib - MeSH - NCBI “nih.gov)](https://www.ncbi.nlm.nih.gov/mesh/?term=Baricitinib) | - 3-azetidineacetonitrile, 1-”ethylsulfonyl)-3-”4-”7H-pyrrolo”2,3-d)pyrimidin-4-yl)-1H-pyrazol-1-yl) - INCB-28050 - Olumiant - baricitinib phosphate - baricitinib phosphate salt - INCB028050   - LY3009104 | "baricitinib" [Mesh] OR "baricitinib" OR "3-azetidineacetonitrile, 1-”ethylsulfonyl)-3-”4-”7H-pyrrolo”2,3-d)pyrimidin-4-yl)-1H-pyrazol-1-yl) " OR "INCB-28050" OR "Olumiant" OR "baricitinib phosphate" OR "baricitinib phosphate salt" OR "INCB028050" OR "LY3009104"  [All MeSH Categories](https://www.ncbi.nlm.nih.gov/mesh/1000048)  baricitinib |
| Outcomes  Vaccination with inactivated vaccine | [Vaccines, Inactivated - MeSH - NCBI “nih.gov)](https://www.ncbi.nlm.nih.gov/mesh/68015164)  Definition: Vaccines in which the infectious microbial nucleic acid components have been destroyed by chemical or physical treatment “e.g., formalin, beta-propiolactone, gamma radiation) without affecting the antigenicity or immunogenicity of the viral coat or bacterial outer membrane proteins. | Inactivated Vaccines  Killed Vaccine  Killed Vaccines  Inactivated Vaccine | "[Vaccines, Inactivated](https://www.ncbi.nlm.nih.gov/mesh/68015164)" [Mesh] OR "[Vaccines, Inactivated](https://www.ncbi.nlm.nih.gov/mesh/68015164)" OR "Inactivated Vaccines" OR "Killed Vaccine" OR "Killed Vaccines" OR "Inactivated Vaccine"  Vaccines, Inactivated  [Poliovirus Vaccine, Inactivated](https://www.ncbi.nlm.nih.gov/mesh/68011054) |
|  | [Influenza Vaccines - MeSH - NCBI “nih.gov)](https://www.ncbi.nlm.nih.gov/mesh/68007252)  Vaccines used to prevent infection by viruses in the family ORTHOMYXOVIRIDAE. It includes both killed and attenuated vaccines. The composition of the vaccines is changed each year in response to antigenic shifts and changes in prevalence of influenza virus strains. The flu vaccines may be mono- or multi-valent, which contains one or more INFLUENZAVIRUS A and INFLUENZAVIRUS B strains. | - Influenza Vaccine - Influenzavirus Vaccines - Influenza Virus Vaccines - Influenzavirus Vaccine - Influenza Virus Vaccine - High-Dose Trivalent Influenza Vaccine - High Dose Trivalent Influenza Vaccine - Trivalent Influenza Vaccine - Quadrivalent Influenza Vaccine - High dose | "[Influenza Vaccines](https://www.ncbi.nlm.nih.gov/mesh/68007252)" [Mesh:NoExp] OR Influenza Vaccine" OR "Influenzavirus Vaccines" OR "Influenza Virus Vaccines" OR "Influenzavirus Vaccine" OR "Influenza Virus Vaccine" OR "High-Dose Trivalent Influenza Vaccine" OR "High Dose Trivalent Influenza Vaccine" OR "Trivalent Influenza Vaccine" OR "Quadrivalent Influenza Vaccine"  “não utilizar [Mesh] pois a árvore do termo inclui VVA e intra-nasal)  [Vaccines](https://www.ncbi.nlm.nih.gov/mesh/68014612)  [Viral Vaccines](https://www.ncbi.nlm.nih.gov/mesh/68014765)  Influenza Vaccines |
|  | [Pneumococcal Vaccines - MeSH - NCBI “nih.gov)](https://www.ncbi.nlm.nih.gov/mesh/68022242)  Vaccines or candidate vaccines used to prevent infections with STREPTOCOCCUS PNEUMONIAE. | - Pneumococcal Vaccine - Pneumovax - Pneumococcal Polysaccharide Vaccine - Pnu Imune Vaccine - PnuImune Vaccine - Conjugated penumococcal vaccine | "[Pneumococcal Vaccines](https://www.ncbi.nlm.nih.gov/mesh/68022242)" [Mesh] OR "[Pneumococcal Vaccines](https://www.ncbi.nlm.nih.gov/mesh/68022242)" OR "Pneumococcal Vaccine" OR "Pneumovax" OR "Pneumococcal Polysaccharide Vaccine" OR "Pnu Imune Vaccine" OR "PnuImune Vaccine" OR "Conjugated penumococcal vaccine"  Pneumococcal Vaccines  [Heptavalent Pneumococcal Conjugate Vaccine](https://www.ncbi.nlm.nih.gov/mesh/2009980)  13 pneumococcal conjugate vaccine |
|  | [Herpes Zoster Vaccine - MeSH - NCBI “nih.gov)](https://www.ncbi.nlm.nih.gov/mesh/68053061)  An attenuated vaccine used to prevent and/or treat HERPES ZOSTER, a disease caused by HUMAN HERPESVIRUS 3. | - Zoster Vaccine - Shingles Vaccine | "Herpes Zoster Vaccine" [Mesh:NoExp] OR " Zoster Vaccine" OR "Shingles Vaccine"  “não utilizar [Mesh] pois a árvore do termo inclui live attenuated zoster  e intra-nasal)  [Vaccines](https://www.ncbi.nlm.nih.gov/mesh/68014612)  [Viral Vaccines](https://www.ncbi.nlm.nih.gov/mesh/68014765)  [Herpesvirus Vaccines](https://www.ncbi.nlm.nih.gov/mesh/68022283)  Herpes Zoster Vaccine |
|  | [Hepatitis B Vaccines - MeSH - NCBI “nih.gov)](https://www.ncbi.nlm.nih.gov/mesh/68017325)  Vaccines or candidate vaccines containing inactivated hepatitis B or some of its component antigens and designed to prevent hepatitis B. Some vaccines may be recombinantly produced. | Hepatitis B Vaccine | "Hepatitis B Vaccines" [Mesh] OR "Hepatitis B Vaccines" OR "Hepatitis B Vaccine"  [Vaccines](https://www.ncbi.nlm.nih.gov/mesh/68014612)  [Viral Vaccines](https://www.ncbi.nlm.nih.gov/mesh/68014765)  [Viral Hepatitis Vaccines](https://www.ncbi.nlm.nih.gov/mesh/68014761)  Hepatitis B Vaccines |
|  | [Papillomavirus Vaccines - MeSH - NCBI “nih.gov)](https://www.ncbi.nlm.nih.gov/mesh/?term=papillomavirus+vaccines)   Vaccines or candidate vaccines used to prevent PAPILLOMAVIRUS INFECTIONS. Human vaccines are intended to reduce the incidence of UTERINE CERVICAL NEOPLASMS, so they are sometimes considered a type of CANCER VACCINES. They are often composed of CAPSID PROTEINS, especially L1 protein, from various types of ALPHAPAPILLOMAVIRUS | - Papillomavirus Vaccine   Human Papillomavirus Vaccines   - HPV Vaccine   Human Papilloma Virus Vaccine   - HPV Vaccines - Human Papilloma Virus Vaccines - Human Papillomavirus Vaccine | "Papillomavirus Vaccines" [Mesh] OR "Papillomavirus Vaccines" OR "Papillomavirus Vaccine" OR "Human Papillomavirus Vaccines" OR "HPV Vaccine" OR "Human Papilloma Virus Vaccine" OR "HPV Vaccines" OR "Human Papilloma Virus Vaccines" OR "Human Papillomavirus Vaccine"  [Vaccines](https://www.ncbi.nlm.nih.gov/mesh/68014612)  [Viral Vaccines](https://www.ncbi.nlm.nih.gov/mesh/68014765)  Papillomavirus Vaccines  [Human Papillomavirus](https://www.ncbi.nlm.nih.gov/mesh/2009889) |
|  | [Poliovirus Vaccines - MeSH - NCBI “nih.gov)](https://www.ncbi.nlm.nih.gov/mesh/68023321)    Vaccines used to prevent POLIOMYELITIS. They include inactivated “POLIOVIRUS VACCINE, INACTIVATED) | - Poliovirus Vaccine - salk | "Poliovirus Vaccines" [Mesh] OR "Poliovirus Vaccines" OR "Poliovirus Vaccine" OR "salk"  [Vaccines](https://www.ncbi.nlm.nih.gov/mesh/68014612)  [Viral Vaccines](https://www.ncbi.nlm.nih.gov/mesh/68014765)  [Poliovirus Vaccine, Inactivated](https://www.ncbi.nlm.nih.gov/mesh/68011054) |
|  | [Hepatitis A Vaccines - MeSH - NCBI “nih.gov)](https://www.ncbi.nlm.nih.gov/mesh/68022362)    Vaccines or candidate vaccines used to prevent infection with hepatitis A virus “HEPATOVIRUS). | - Hepatovirus Vaccines - Hepatitis A Vaccine - Vaqta - Havrix | "Hepatitis A Vaccines" [Mesh] OR "Hepatitis A Vaccines" OR "Hepatovirus Vaccines" OR "Hepatitis A Vaccine" OR "Vaqta" OR "Havrix"  [Vaccines](https://www.ncbi.nlm.nih.gov/mesh/68014612)  [Viral Hepatitis Vaccines](https://www.ncbi.nlm.nih.gov/mesh/68014761)  Hepatitis A Vaccines |
|  | [Meningococcal Vaccines - MeSH - NCBI “nih.gov)](https://www.ncbi.nlm.nih.gov/mesh/68022401)    Vaccines or candidate vaccines used to prevent infection with NEISSERIA MENINGITIDIS. | Meningococcal Vaccine  Menactra  Tetravalent Meningococcal Vaccine  Menomune  Menveo  Bivalent Meningococcal Vaccine  Meningococcal Polysaccharide Vaccine | "Meningococcal Vaccines" [Mesh] OR " Meningococcal Vaccine" OR "Menactra" OR "Tetravalent Meningococcal Vaccine" OR "Menomune OR "Menveo" OR "Bivalent Meningococcal Vaccine" OR "Meningococcal Polysaccharide Vaccine"    [Vaccines](https://www.ncbi.nlm.nih.gov/mesh/68014612)  Meningococcal Vaccines C, B or ACWY |
|  | [Diphtheria-Tetanus-Pertussis Vaccine - MeSH - NCBI “nih.gov)](https://www.ncbi.nlm.nih.gov/mesh/?term=vaccine+per)   A vaccine consisting of DIPHTHERIA TOXOID; TETANUS TOXOID; and whole-cell PERTUSSIS VACCINE. The vaccine protects against diphtheria, tetanus, and whooping cough. | Diphtheria Tetanus Pertussis Vaccine  DTwP Vaccine  DPT Vaccine  DTP Vaccine  Di Te Per Vaccine  Diphtheria Pertussis Tetanus Vaccine | "Diphtheria-Tetanus-Pertussis Vaccine" [Mesh] OR "Diphtheria-Tetanus-Pertussis Vaccine" OR " Diphtheria Tetanus Pertussis Vaccine" OR "DTwP Vaccine" OR "DPT Vaccine" OR "DTP Vaccine" OR "Di Te Per Vaccine" OR "Diphtheria Pertussis Tetanus Vaccine"  [Vaccines](https://www.ncbi.nlm.nih.gov/mesh/68014612)  [Pertussis Vaccine](https://www.ncbi.nlm.nih.gov/mesh/68010567)  Diphtheria-Tetanus-Pertussis Vaccine |
|  | [Haemophilus influenza type b polysaccharide vaccine-tetanus toxin conj - MeSH - NCBI “nih.gov)](https://www.ncbi.nlm.nih.gov/mesh/?term=haemophilus+influenza+type+b+polysaccharide+vaccine+tetanus+toxin+conjugate)    the type b capsular polysaccharide of HIB-vaccine is conjugated to tetanus toxoid | HIBest  PRP-T  Hib-TT  Hib capsular polysaccharide-tetanus vaccine  Hib CPS-tetanus vaccine  OmniHIB  ActHIB | "Haemophilus influenza type b polysaccharide vaccine-tetanus toxin conjugate" OR "HIBest" OR "PRP-T" OR "Hib-TT" OR "Hib capsular polysaccharide-tetanus vaccine" OR "Hib CPS-tetanus vaccine" OR "OmniHIB" OR "ActHIB" |

**Question 09.** Is there a benefit to vaccine response by temporarily stopping methotrexate, mycophenolate, iJAKs before or after vaccination?

| Group | **MeSH Terms**  and definitions | **Entry Terms** | **Search Key** |
| --- | --- | --- | --- |
| Population  Patients with immune-mediated rheumatic diseases “IMRD) | [Rheumatic Diseases - MeSH - NCBI “nih.gov)](https://www.ncbi.nlm.nih.gov/mesh/68012216)  Disorders of connective tissue, especially the joints and related structures, characterized by inflammation, degeneration, or metabolic derangement. | Rheumatic Disease  Rheumatism | "[Rheumatic Diseases](https://www.ncbi.nlm.nih.gov/mesh/68012216)" [Mesh:NoExp] OR "Rheumatic Disease" OR "Rheumatism" OR  "Immune-mediated rheumatic diseases"  “não utilizar [Mesh] pois a árvore do termo inclui gota, fibromialgia e crianças) |
|  | [Arthritis, Rheumatoid - MeSH - NCBI “nih.gov)](https://www.ncbi.nlm.nih.gov/mesh/68001172)  A chronic systemic disease, primarily of the joints, marked by inflammatory changes in the synovial membranes and articular structures, widespread fibrinoid degeneration of the collagen fibers in mesenchymal tissues, and by atrophy and rarefaction of bony structures. Etiology is unknown, but autoimmune mechanisms have been implicated. | - Rheumatoid Arthritis | "[Arthritis, Rheumatoid](https://www.ncbi.nlm.nih.gov/mesh/68001172)" [Mesh:NoExp]  OR "Rheumatoid Arthritis" OR "[Rheumatoid Nodule](https://www.ncbi.nlm.nih.gov/mesh/68012218)" OR "[Rheumatoid Vasculitis](https://www.ncbi.nlm.nih.gov/mesh/68056653)" OR "[Sjogren's Syndrome](https://www.ncbi.nlm.nih.gov/mesh/68012859)"  “não utilizar [Mesh] pois a árvore do termo inclui caplan syndrome, felty syndrome) |
|  | [Spondylarthritis - MeSH - NCBI “nih.gov)](https://www.ncbi.nlm.nih.gov/mesh/68025241)  Inflammation of the joints of the SPINE, the intervertebral articulations. | - Spondylarthritides - Spinal Arthritis - Spinal Arthritides | "[Spondylarthritis](https://www.ncbi.nlm.nih.gov/mesh/68025241)" [Mesh] OR "[Spondylarthritis](https://www.ncbi.nlm.nih.gov/mesh/68025241)" OR "Spondylarthritides" OR "Spinal Arthritis" OR "Spinal Arthritides"  [Spondylitis](https://www.ncbi.nlm.nih.gov/mesh/68013166)  Spondylarthritis  [Osteoarthritis, Spine](https://www.ncbi.nlm.nih.gov/mesh/68055013)  [Spondylarthropathies](https://www.ncbi.nlm.nih.gov/mesh/68025242)  [Arthritis, Psoriatic](https://www.ncbi.nlm.nih.gov/mesh/68015535)  [Arthritis, Reactive](https://www.ncbi.nlm.nih.gov/mesh/68016918)  [Axial Spondyloarthritis](https://www.ncbi.nlm.nih.gov/mesh/2101123) + |
|  | [Arthritis, Psoriatic - MeSH - NCBI “nih.gov)](https://www.ncbi.nlm.nih.gov/mesh/68015535)  A type of inflammatory arthritis associated with PSORIASIS, often involving the axial joints and the peripheral terminal interphalangeal joints. It is characterized by the presence of HLA-B27-associated SPONDYLARTHROPATHY, and the absence of rheumatoid factor. | - Arthritic Psoriasis - Psoriatic Arthritis - Psoriasis Arthropathica - Psoriatic Arthropathy - Psoriatic Arthropathies | "[Arthritis, Psoriatic](https://www.ncbi.nlm.nih.gov/mesh/68015535)" [Mesh] OR "[Arthritis, Psoriatic](https://www.ncbi.nlm.nih.gov/mesh/68015535)" OR "Arthritic Psoriasis" OR "Psoriatic Arthritis" OR "Psoriasis Arthropathica" OR "Psoriatic Arthropathy" OR "Psoriatic Arthropathies"  [Spondylarthritis](https://www.ncbi.nlm.nih.gov/mesh/68025241)  [Spondylarthropathies](https://www.ncbi.nlm.nih.gov/mesh/68025242)  Arthritis, Psoriatic |
|  | [Spondylitis, Ankylosing - MeSH - NCBI “nih.gov)](https://www.ncbi.nlm.nih.gov/mesh/?term=ankylosing+spondylitis)  A chronic inflammatory condition affecting the axial joints, such as the SACROILIAC JOINT and other intervertebral or costovertebral joints. It occurs predominantly in young males and is characterized by pain and stiffness of joints “ANKYLOSIS) with inflammation at tendon insertions. | - Bechterew's Disease - Bechterews Disease - Marie Struempell Disease - Spondylarthritis Ankylopoietica - Spondyloarthritis Ankylopoietica - Ankylosing Spondylitis - Ankylosing Spondylarthritis - Ankylosing Spondylarthritides - Ankylosing Spondyloarthritis - Ankylosing Spondyloarthritides - Spondylitis Ankylopoietica - Bechterew Disease - Rheumatoid Spondylitis | "[Spondylitis, Ankylosing](https://www.ncbi.nlm.nih.gov/mesh/?term=ankylosing+spondylitis)" [Mesh] OR "[Spondylitis, Ankylosing](https://www.ncbi.nlm.nih.gov/mesh/?term=ankylosing+spondylitis)" OR "Bechterew's Disease" OR "Bechterews Disease" OR "Marie Struempell Disease" OR "Spondylarthritis Ankylopoietica" OR "Spondyloarthritis Ankylopoietica" OR "Ankylosing Spondylitis" OR "Ankylosing Spondylarthritis" OR "Ankylosing Spondylarthritides" OR "Ankylosing Spondyloarthritis" OR "Ankylosing Spondyloarthritides" OR "Spondylitis Ankylopoietica" OR "Bechterew Disease" OR "Rheumatoid Spondylitis"  [Spondylarthritis](https://www.ncbi.nlm.nih.gov/mesh/68025241)  [Spondylarthropathies](https://www.ncbi.nlm.nih.gov/mesh/68025242)  [Axial Spondyloarthritis](https://www.ncbi.nlm.nih.gov/mesh/2101123)  Spondylitis, Ankylosing |
|  | [Lupus Erythematosus, Systemic - MeSH - NCBI “nih.gov)](https://www.ncbi.nlm.nih.gov/mesh/68008180)  A chronic, relapsing, inflammatory, and often febrile multisystemic disorder of connective tissue, characterized principally by involvement of the skin, joints, kidneys, and serosal membranes. It is of unknown etiology, but is thought to represent a failure of the regulatory mechanisms of the autoimmune system. The disease is marked by a wide range of system dysfunctions, an elevated erythrocyte sedimentation rate, and the formation of LE cells in the blood or bone marrow. | - Systemic Lupus Erythematosus - Lupus Erythematosus Disseminatus - Libman Sacks Disease | "[Lupus Erythematosus, Systemic](https://www.ncbi.nlm.nih.gov/mesh/68008180)" [Mesh] OR "[Lupus Erythematosus, Systemic](https://www.ncbi.nlm.nih.gov/mesh/68008180)" OR "Systemic Lupus Erythematosus " OR "Lupus Erythematosus Disseminatus" OR "Libman Sacks Disease"  [Connective Tissue Diseases](https://www.ncbi.nlm.nih.gov/mesh/68003240)  Lupus Erythematosus, Systemic  [Lupus Nephritis](https://www.ncbi.nlm.nih.gov/mesh/68008181)  [Lupus Vasculitis, Central Nervous System](https://www.ncbi.nlm.nih.gov/mesh/68020945) |
|  | [Myositis - MeSH - NCBI “nih.gov)](https://www.ncbi.nlm.nih.gov/mesh/?term=inflammatory+myopathies)  Inflammation of a muscle or muscle tissue. | - Myositides - Inflammatory Muscle Diseases - Inflammatory Muscle Disease - Inflammatory Myopathy - Inflammatory Myopathies - Proliferative Myositides - Proliferative Myositis - Infectious Myositides - Infectious Myositis - Idiopathic Inflammatory Myopathies - Idiopathic Inflammatory Myopathy - Idiopathic Inflammatory Myositis - Focal Myositides - Focal Myositis | "[Myositis](https://www.ncbi.nlm.nih.gov/mesh/?term=inflammatory+myopathies)" [Mesh] OR "[Myositis](https://www.ncbi.nlm.nih.gov/mesh/?term=inflammatory+myopathies)" OR "Myositides" OR "Inflammatory Muscle Diseases" OR "Inflammatory Muscle Disease" OR "Inflammatory Myopathy" OR "Inflammatory Myopathies" OR "Proliferative Myositides" OR "Proliferative Myositis" OR "Infectious Myositides" OR "Infectious Myositis" OR "Idiopathic Inflammatory Myopathies" OR "Idiopathic Inflammatory Myopathy" OR "Idiopathic Inflammatory Myositis" OR "Focal Myositides" OR "Focal Myositis"  [Muscular Diseases](https://www.ncbi.nlm.nih.gov/mesh/68009135)  Myositis  [Myositis Ossificans](https://www.ncbi.nlm.nih.gov/mesh/68009221)  [Myositis, Inclusion Body](https://www.ncbi.nlm.nih.gov/mesh/68018979)  [Orbital Myositis](https://www.ncbi.nlm.nih.gov/mesh/68055622)  [Polymyositis](https://www.ncbi.nlm.nih.gov/mesh/68017285)  [Dermatomyositis](https://www.ncbi.nlm.nih.gov/mesh/68003882)  [Pyomyositis](https://www.ncbi.nlm.nih.gov/mesh/68052880) |
|  | [Systemic Vasculitis - MeSH - NCBI “nih.gov)](https://www.ncbi.nlm.nih.gov/mesh/?term=systemic+vasculitides)  A heterogeneous group of diseases characterized by inflammation and necrosis of the blood vessel walls. | - Systemic Vasculitides | "[Systemic Vasculitis](https://www.ncbi.nlm.nih.gov/mesh/?term=systemic+vasculitides)" [Mesh] OR "[Systemic Vasculitis](https://www.ncbi.nlm.nih.gov/mesh/?term=systemic+vasculitides)" OR "Systemic Vasculitides"  [Vasculitis](https://www.ncbi.nlm.nih.gov/mesh/68014657)  Systemic Vasculitis  [Anti-Neutrophil Cytoplasmic Antibody-Associated Vasculitis](https://www.ncbi.nlm.nih.gov/mesh/68056648)  [Churg-Strauss Syndrome](https://www.ncbi.nlm.nih.gov/mesh/68015267)  [Granulomatosis with Polyangiitis](https://www.ncbi.nlm.nih.gov/mesh/68014890)  [Microscopic Polyangiitis](https://www.ncbi.nlm.nih.gov/mesh/68055953)  [Polyarteritis Nodosa](https://www.ncbi.nlm.nih.gov/mesh/68010488)  [Rheumatoid Vasculitis](https://www.ncbi.nlm.nih.gov/mesh/68056653) |
|  | [Sjogren's Syndrome - MeSH - NCBI “nih.gov)](https://www.ncbi.nlm.nih.gov/mesh/68012859)  Chronic inflammatory and autoimmune disease in which the salivary and lacrimal glands undergo progressive destruction by lymphocytes and plasma cells resulting in decreased production of saliva and tears. The primary form, often called sicca syndrome, involves both KERATOCONJUNCTIVITIS SICCA and XEROSTOMIA. The secondary form includes, in addition, the presence of a connective tissue disease, usually rheumatoid arthritis. | - Sjogrens Syndrome - Sjogren Syndrome - Sicca Syndrome | "[Sjogren's Syndrome](https://www.ncbi.nlm.nih.gov/mesh/68012859)" [Mesh] OR "[Sjogren's Syndrome](https://www.ncbi.nlm.nih.gov/mesh/68012859)"  OR "Sjogrens Syndrome" OR "Sjogren Syndrome" OR "Sicca Syndrome"  [Arthritis](https://www.ncbi.nlm.nih.gov/mesh/68001168)  [Arthritis, Rheumatoid](https://www.ncbi.nlm.nih.gov/mesh/68001172)  Sjogren's Syndrome |
|  | [Scleroderma, Systemic - MeSH - NCBI “nih.gov)](https://www.ncbi.nlm.nih.gov/mesh/68012595)  A chronic multi-system disorder of CONNECTIVE TISSUE. It is characterized by SCLEROSIS in the SKIN, the LUNGS, the HEART, the GASTROINTESTINAL TRACT, the KIDNEYS, and the MUSCULOSKELETAL SYSTEM. Other important features include diseased small BLOOD VESSELS and AUTOANTIBODIES. The disorder is named for its most prominent feature “hard skin), and classified into subsets by the extent of skin thickening: LIMITED SCLERODERMA and DIFFUSE SCLERODERMA. | - Systemic Sclerosis - Systemic Scleroderma | "[Scleroderma, Systemic](https://www.ncbi.nlm.nih.gov/mesh/68012595)" [Mesh] OR "[Scleroderma, Systemic](https://www.ncbi.nlm.nih.gov/mesh/68012595)" OR "Systemic Sclerosis" OR "Systemic Scleroderma"  [Connective Tissue Diseases](https://www.ncbi.nlm.nih.gov/mesh/68003240)  Scleroderma, Systemic  [Scleroderma, Diffuse](https://www.ncbi.nlm.nih.gov/mesh/68045743)  [Scleroderma, Limited](https://www.ncbi.nlm.nih.gov/mesh/68045745)  [CREST Syndrome](https://www.ncbi.nlm.nih.gov/mesh/68017675) |
|  | [Mixed Connective Tissue Disease - MeSH - NCBI “nih.gov)](https://www.ncbi.nlm.nih.gov/mesh/?term=Mixed+connective+tissue+disease)  A syndrome with overlapping clinical features of systemic lupus erythematosus, scleroderma, polymyositis, and Raynaud's phenomenon. The disease is differentially characterized by high serum titers of antibodies to ribonuclease-sensitive extractable “saline soluble) nuclear antigen and a "speckled" epidermal nuclear staining pattern on direct immunofluorescence. | - Sharp Syndrome - MCTD | "[Mixed Connective Tissue Disease](https://www.ncbi.nlm.nih.gov/mesh/?term=Mixed+connective+tissue+disease)" [Mesh] OR "[Mixed Connective Tissue Disease](https://www.ncbi.nlm.nih.gov/mesh/?term=Mixed+connective+tissue+disease)" OR "Sharp Syndrome" OR "MCTD"  [Connective Tissue Diseases](https://www.ncbi.nlm.nih.gov/mesh/68003240)  Mixed Connective Tissue Disease |
| Intervention | [Drug Tapering - MeSH - NCBI “nih.gov)](https://www.ncbi.nlm.nih.gov/mesh/?term=drug+discontinuation)  Gradual discontinuation or reduction of a therapeutic dose of a particular drug over a period of time. | - Dose Reduction - Dose Reductions - Dose Tapering - Dose Taperings | "Drug Tapering" [Mesh] OR "Drug Tapering"  OR "Dose Reduction"  OR "Dose Reductions"  OR "Dose Tapering"  OR "Dose Taperings" |
|  | [Methotrexate - MeSH - NCBI “nih.gov)](https://www.ncbi.nlm.nih.gov/mesh/68008727)  An antineoplastic antimetabolite with immunosuppressant properties. It is an inhibitor of TETRAHYDROFOLATE DEHYDROGENASE and prevents the formation of tetrahydrofolate, necessary for synthesis of thymidylate, an essential component of DNA. | - Amethopterin - Methotrexate, “D)-Isomer - Methotrexate, “DL)-Isomer - Mexate - Methotrexate Sodium   Methotrexate Hydrate   - Dicesium Salt Methotrexate | "Methotrexate" [Mesh] OR "Methotrexate" OR "Amethopterin" OR "Methotrexate, “D)-Isomer" OR "Methotrexate, “DL)-Isomer" OR "Mexate" OR "Methotrexate Sodium" OR "Methotrexate Hydrate" OR "Dicesium Salt Methotrexate"  [Pteridines](https://www.ncbi.nlm.nih.gov/mesh/68011621)  [Pterins](https://www.ncbi.nlm.nih.gov/mesh/68011622)  [Aminopterin](https://www.ncbi.nlm.nih.gov/mesh/68000630)  Methotrexate |
|  | [Mycophenolic Acid - MeSH - NCBI “nih.gov)](https://www.ncbi.nlm.nih.gov/mesh/68009173)  Compound derived from Penicillium stoloniferum and related species. It blocks de novo biosynthesis of purine nucleotides by inhibition of the enzyme inosine monophosphate dehydrogenase “IMP DEHYDROGENASE). Mycophenolic acid exerts selective effects on the immune system in which it prevents the proliferation of T-CELLS, LYMPHOCYTES, and the formation of antibodies from B-CELLS. It may also inhibit recruitment of LEUKOCYTES to sites of INFLAMMATION. | - Mycophenolate Mofetil - Mycophenolic Acid Morpholinoethyl Ester - Cellcept - Mycophenolate Sodium - Sodium Mycophenolat - Myfortic - Mycophenolate Mofetil Hydrochloride - RS 61443 | "Mycophenolic Acid" [Mesh] OR "Mycophenolic Acid" OR " Mycophenolate Mofetil" OR "Mycophenolic Acid" OR "Morpholinoethyl Ester" OR "Cellcept" OR "Mycophenolate Sodium" OR "Sodium Mycophenolat" OR "Myfortic" OR "Mycophenolate Mofetil Hydrochloride" OR "RS 61443"  [Acids, Acyclic](https://www.ncbi.nlm.nih.gov/mesh/68000144)  [Caproates](https://www.ncbi.nlm.nih.gov/mesh/68002208)  Mycophenolic Acid |
|  | [Abatacept - MeSH - NCBI “nih.gov)](https://www.ncbi.nlm.nih.gov/mesh/?term=abatacept)  A fusion protein immunoconjugate of the extracellular domain of CTLA4 and the Fc domain of human IgG1. It functions as a T-cell co-stimulation blocker that inhibits TNF-ALPHA and prevents the activation of T-LYMPHOCYTES. It is used in the treatment of RHEUMATOID ARTHRITIS. | - CTLA-4-Ig - Cytotoxic T Lymphocyte-Associated Antigen 4-Immunoglobulin - Cytotoxic T Lymphocyte Associated Antigen 4 Immunoglobulin - CTLA4-Ig - CTLA4-Ig Immunoconjugate - CTLA4 Ig Immunoconjugate - BMS224818 - BMS 224818 - Belatacept - Orencia - BMS 188667 - BMS188667 - LEA29Y - Nulojix | "Abatacept" [Mesh] OR "Abatacept" OR "CTLA-4-Ig" OR "Cytotoxic T Lymphocyte-Associated Antigen 4-Immunoglobulin" OR "Cytotoxic T Lymphocyte Associated Antigen 4 Immunoglobulin" OR "CTLA4-Ig" OR "CTLA4-Ig Immunoconjugate" OR "CTLA4 Ig Immunoconjugate" OR "BMS224818" OR "BMS 224818" OR "Belatacept" OR "Orencia" OR "BMS 188667" OR "LEA29Y" OR "Nulojix"  [Antibodies](https://www.ncbi.nlm.nih.gov/mesh/68000906)  [Immunoconjugates](https://www.ncbi.nlm.nih.gov/mesh/68018796)  Abatacept |
|  | [Janus Kinase Inhibitors - MeSH - NCBI “nih.gov)](https://www.ncbi.nlm.nih.gov/mesh/2023419)  Agents that inhibit JANUS KINASES. | - JAK Inhibitors - Janus Kinase Inhibitor - JAK Inhibitor | "Janus Kinase Inhibitors" [Mesh] OR "Janus Kinase Inhibitors" OR "JAK Inhibitors" OR "Janus Kinase Inhibitor" OR "JAK Inhibitor"  [Molecular Mechanisms of Pharmacological Action](https://www.ncbi.nlm.nih.gov/mesh/68045504)  [Enzyme Inhibitors](https://www.ncbi.nlm.nih.gov/mesh/68004791)  [Protein Kinase Inhibitors](https://www.ncbi.nlm.nih.gov/mesh/68047428)  Janus Kinase Inhibitors |
|  | [upadacitinib - MeSH - NCBI “nih.gov)](https://www.ncbi.nlm.nih.gov/mesh/?term=Upadacitinib)  a JAK-1 inhibitor; for treatment of rheumatoid arthritis | - “3S,4R)-3-ethyl-4-”3H-imidazo”1,2-a)pyrrolo”2,3-e)pyrazin-8-yl)-N-”2,2,2-trifluoroethyl)-1-pyrrolidinecarboxamide - ABT-494 - Rinvoq | "upadacitinib" [Mesh] OR "upadacitinib" OR "“3S,4R)-3-ethyl-4-”3H-imidazo”1,2-a)pyrrolo”2,3-e)pyrazin-8-yl)-N-”2,2,2-trifluoroethyl)-1-pyrrolidinecarboxamide" OR "ABT-494" OR "Rinvoq" |
|  | [Protein Kinase Inhibitors - MeSH - NCBI “nih.gov)](https://www.ncbi.nlm.nih.gov/mesh/68047428)  Agents that inhibit PROTEIN KINASES. | Protein Kinase Inhibitor | "Protein Kinase Inhibitors" [Mesh] OR "Protein Kinase Inhibitors" OR " Protein Kinase Inhibitor"  [Enzyme Inhibitors](https://www.ncbi.nlm.nih.gov/mesh/68004791)  Protein Kinase Inhibitors  [Janus Kinase Inhibitors](https://www.ncbi.nlm.nih.gov/mesh/2023419)  [MTOR Inhibitors](https://www.ncbi.nlm.nih.gov/mesh/2101251)  [Tyrosine Protein Kinase Inhibitors](https://www.ncbi.nlm.nih.gov/mesh/2103139) |
|  | [tofacitinib - MeSH - NCBI “nih.gov)](https://www.ncbi.nlm.nih.gov/mesh/?term=Tofacitinib) | - tasocitinib - tofacitinib citrate - Xeljanz - CP 690550 | "tofacitinib" [Mesh] OR "tofacitinib" OR "tasocitinib" OR "tofacitinib citrate" OR "Xeljanz" OR "CP 690550" |
|  | [baricitinib - MeSH - NCBI “nih.gov)](https://www.ncbi.nlm.nih.gov/mesh/?term=Baricitinib)  inhibits Janus kinases JAK1 and JAK2 | - 3-azetidineacetonitrile, 1-”ethylsulfonyl)-3-”4-”7H-pyrrolo”2,3-d)pyrimidin-4-yl)-1H-pyrazol-1-yl) - INCB-28050 - Olumiant - baricitinib phosphate - baricitinib phosphate salt - INCB028050   - LY3009104 | "baricitinib" [Mesh] OR "baricitinib" OR "3-azetidineacetonitrile, 1-”ethylsulfonyl)-3-”4-”7H-pyrrolo”2,3-d)pyrimidin-4-yl)-1H-pyrazol-1-yl) " OR "INCB-28050" OR "Olumiant" OR "baricitinib phosphate" OR "baricitinib phosphate salt" OR "INCB028050" OR "LY3009104"  [All MeSH Categories](https://www.ncbi.nlm.nih.gov/mesh/1000048)  Baricitinib |
|  | [Haemophilus influenza type b polysaccharide vaccine-tetanus toxin conj - MeSH - NCBI “nih.gov)](https://www.ncbi.nlm.nih.gov/mesh/?term=haemophilus+influenza+type+b+polysaccharide+vaccine+tetanus+toxin+conjugate)    the type b capsular polysaccharide of HIB-vaccine is conjugated to tetanus toxoid | HIBest  PRP-T  Hib-TT  Hib capsular polysaccharide-tetanus vaccine  Hib CPS-tetanus vaccine  OmniHIB  ActHIB | "Haemophilus influenza type b polysaccharide vaccine-tetanus toxin conjugate" OR "HIBest" OR "PRP-T" OR "Hib-TT" OR "Hib capsular polysaccharide-tetanus vaccine" OR "Hib CPS-tetanus vaccine" OR "OmniHIB" OR "ActHIB" |

**Question 10.**  Is there a difference in the immune response between conventional “tri- and tetravalent) and high-dose influenza vaccines in patients with immune-mediated rheumatic diseases “IMRD)?

| Group | **MeSH Terms**  and definitions | **Entry Terms** | **Search Key** |
| --- | --- | --- | --- |
| Population  Patients with immune-mediated rheumatic diseases “IMRD) | [Rheumatic Diseases - MeSH - NCBI “nih.gov)](https://www.ncbi.nlm.nih.gov/mesh/68012216)  Disorders of connective tissue, especially the joints and related structures, characterized by inflammation, degeneration, or metabolic derangement. | Rheumatic Disease  Rheumatism | "[Rheumatic Diseases](https://www.ncbi.nlm.nih.gov/mesh/68012216)" [Mesh:NoExp] OR "Rheumatic Disease" OR "Rheumatism" OR  "Immune-mediated rheumatic diseases"  “não utilizar [Mesh] pois a árvore do termo inclui gota, fibromialgia e crianças) |
|  | [Arthritis, Rheumatoid - MeSH - NCBI “nih.gov)](https://www.ncbi.nlm.nih.gov/mesh/68001172)  A chronic systemic disease, primarily of the joints, marked by inflammatory changes in the synovial membranes and articular structures, widespread fibrinoid degeneration of the collagen fibers in mesenchymal tissues, and by atrophy and rarefaction of bony structures. Etiology is unknown, but autoimmune mechanisms have been implicated. | - Rheumatoid Arthritis | "[Arthritis, Rheumatoid](https://www.ncbi.nlm.nih.gov/mesh/68001172)" [Mesh:NoExp]  OR "Rheumatoid Arthritis" OR "[Rheumatoid Nodule](https://www.ncbi.nlm.nih.gov/mesh/68012218)" OR "[Rheumatoid Vasculitis](https://www.ncbi.nlm.nih.gov/mesh/68056653)" OR "[Sjogren's Syndrome](https://www.ncbi.nlm.nih.gov/mesh/68012859)"  “não utilizar [Mesh] pois a árvore do termo inclui caplan syndrome, felty syndrome) |
|  | [Spondylarthritis - MeSH - NCBI “nih.gov)](https://www.ncbi.nlm.nih.gov/mesh/68025241)  Inflammation of the joints of the SPINE, the intervertebral articulations. | - Spondylarthritides - Spinal Arthritis - Spinal Arthritides | "[Spondylarthritis](https://www.ncbi.nlm.nih.gov/mesh/68025241)" [Mesh] OR "[Spondylarthritis](https://www.ncbi.nlm.nih.gov/mesh/68025241)" OR "Spondylarthritides" OR "Spinal Arthritis" OR "Spinal Arthritides"  [Spondylitis](https://www.ncbi.nlm.nih.gov/mesh/68013166)  Spondylarthritis  [Osteoarthritis, Spine](https://www.ncbi.nlm.nih.gov/mesh/68055013)  [Spondylarthropathies](https://www.ncbi.nlm.nih.gov/mesh/68025242)  [Arthritis, Psoriatic](https://www.ncbi.nlm.nih.gov/mesh/68015535)  [Arthritis, Reactive](https://www.ncbi.nlm.nih.gov/mesh/68016918)  [Axial Spondyloarthritis](https://www.ncbi.nlm.nih.gov/mesh/2101123) + |
|  | [Arthritis, Psoriatic - MeSH - NCBI “nih.gov)](https://www.ncbi.nlm.nih.gov/mesh/68015535)  A type of inflammatory arthritis associated with PSORIASIS, often involving the axial joints and the peripheral terminal interphalangeal joints. It is characterized by the presence of HLA-B27-associated SPONDYLARTHROPATHY, and the absence of rheumatoid factor. | - Arthritic Psoriasis - Psoriatic Arthritis - Psoriasis Arthropathica - Psoriatic Arthropathy - Psoriatic Arthropathies | "[Arthritis, Psoriatic](https://www.ncbi.nlm.nih.gov/mesh/68015535)" [Mesh] OR "[Arthritis, Psoriatic](https://www.ncbi.nlm.nih.gov/mesh/68015535)" OR "Arthritic Psoriasis" OR "Psoriatic Arthritis" OR "Psoriasis Arthropathica" OR "Psoriatic Arthropathy" OR "Psoriatic Arthropathies"  [Spondylarthritis](https://www.ncbi.nlm.nih.gov/mesh/68025241)  [Spondylarthropathies](https://www.ncbi.nlm.nih.gov/mesh/68025242)  Arthritis, Psoriatic |
|  | [Spondylitis, Ankylosing - MeSH - NCBI “nih.gov)](https://www.ncbi.nlm.nih.gov/mesh/?term=ankylosing+spondylitis)  A chronic inflammatory condition affecting the axial joints, such as the SACROILIAC JOINT and other intervertebral or costovertebral joints. It occurs predominantly in young males and is characterized by pain and stiffness of joints “ANKYLOSIS) with inflammation at tendon insertions. | - Bechterew's Disease - Bechterews Disease - Marie Struempell Disease - Spondylarthritis Ankylopoietica - Spondyloarthritis Ankylopoietica - Ankylosing Spondylitis - Ankylosing Spondylarthritis - Ankylosing Spondylarthritides - Ankylosing Spondyloarthritis - Ankylosing Spondyloarthritides - Spondylitis Ankylopoietica - Bechterew Disease - Rheumatoid Spondylitis | "[Spondylitis, Ankylosing](https://www.ncbi.nlm.nih.gov/mesh/?term=ankylosing+spondylitis)" [Mesh] OR "[Spondylitis, Ankylosing](https://www.ncbi.nlm.nih.gov/mesh/?term=ankylosing+spondylitis)" OR "Bechterew's Disease" OR "Bechterews Disease" OR "Marie Struempell Disease" OR "Spondylarthritis Ankylopoietica" OR "Spondyloarthritis Ankylopoietica" OR "Ankylosing Spondylitis" OR "Ankylosing Spondylarthritis" OR "Ankylosing Spondylarthritides" OR "Ankylosing Spondyloarthritis" OR "Ankylosing Spondyloarthritides" OR "Spondylitis Ankylopoietica" OR "Bechterew Disease" OR "Rheumatoid Spondylitis"  [Spondylarthritis](https://www.ncbi.nlm.nih.gov/mesh/68025241)  [Spondylarthropathies](https://www.ncbi.nlm.nih.gov/mesh/68025242)  [Axial Spondyloarthritis](https://www.ncbi.nlm.nih.gov/mesh/2101123)  Spondylitis, Ankylosing |
|  | [Lupus Erythematosus, Systemic - MeSH - NCBI “nih.gov)](https://www.ncbi.nlm.nih.gov/mesh/68008180)  A chronic, relapsing, inflammatory, and often febrile multisystemic disorder of connective tissue, characterized principally by involvement of the skin, joints, kidneys, and serosal membranes. It is of unknown etiology, but is thought to represent a failure of the regulatory mechanisms of the autoimmune system. The disease is marked by a wide range of system dysfunctions, an elevated erythrocyte sedimentation rate, and the formation of LE cells in the blood or bone marrow. | - Systemic Lupus Erythematosus - Lupus Erythematosus Disseminatus - Libman Sacks Disease | "[Lupus Erythematosus, Systemic](https://www.ncbi.nlm.nih.gov/mesh/68008180)" [Mesh] OR "[Lupus Erythematosus, Systemic](https://www.ncbi.nlm.nih.gov/mesh/68008180)" OR "Systemic Lupus Erythematosus " OR "Lupus Erythematosus Disseminatus" OR "Libman Sacks Disease"  [Connective Tissue Diseases](https://www.ncbi.nlm.nih.gov/mesh/68003240)  Lupus Erythematosus, Systemic  [Lupus Nephritis](https://www.ncbi.nlm.nih.gov/mesh/68008181)  [Lupus Vasculitis, Central Nervous System](https://www.ncbi.nlm.nih.gov/mesh/68020945) |
|  | [Myositis - MeSH - NCBI “nih.gov)](https://www.ncbi.nlm.nih.gov/mesh/?term=inflammatory+myopathies)  Inflammation of a muscle or muscle tissue. | - Myositides - Inflammatory Muscle Diseases - Inflammatory Muscle Disease - Inflammatory Myopathy - Inflammatory Myopathies - Proliferative Myositides - Proliferative Myositis - Infectious Myositides - Infectious Myositis - Idiopathic Inflammatory Myopathies - Idiopathic Inflammatory Myopathy - Idiopathic Inflammatory Myositis - Focal Myositides - Focal Myositis | "[Myositis](https://www.ncbi.nlm.nih.gov/mesh/?term=inflammatory+myopathies)" [Mesh] OR "[Myositis](https://www.ncbi.nlm.nih.gov/mesh/?term=inflammatory+myopathies)" OR "Myositides" OR "Inflammatory Muscle Diseases" OR "Inflammatory Muscle Disease" OR "Inflammatory Myopathy" OR "Inflammatory Myopathies" OR "Proliferative Myositides" OR "Proliferative Myositis" OR "Infectious Myositides" OR "Infectious Myositis" OR "Idiopathic Inflammatory Myopathies" OR "Idiopathic Inflammatory Myopathy" OR "Idiopathic Inflammatory Myositis" OR "Focal Myositides" OR "Focal Myositis"  [Muscular Diseases](https://www.ncbi.nlm.nih.gov/mesh/68009135)  Myositis  [Myositis Ossificans](https://www.ncbi.nlm.nih.gov/mesh/68009221)  [Myositis, Inclusion Body](https://www.ncbi.nlm.nih.gov/mesh/68018979)  [Orbital Myositis](https://www.ncbi.nlm.nih.gov/mesh/68055622)  [Polymyositis](https://www.ncbi.nlm.nih.gov/mesh/68017285)  [Dermatomyositis](https://www.ncbi.nlm.nih.gov/mesh/68003882)  [Pyomyositis](https://www.ncbi.nlm.nih.gov/mesh/68052880) |
|  | [Systemic Vasculitis - MeSH - NCBI “nih.gov)](https://www.ncbi.nlm.nih.gov/mesh/?term=systemic+vasculitides)  A heterogeneous group of diseases characterized by inflammation and necrosis of the blood vessel walls. | - Systemic Vasculitides | "[Systemic Vasculitis](https://www.ncbi.nlm.nih.gov/mesh/?term=systemic+vasculitides)" [Mesh] OR "[Systemic Vasculitis](https://www.ncbi.nlm.nih.gov/mesh/?term=systemic+vasculitides)" OR "Systemic Vasculitides"  [Vasculitis](https://www.ncbi.nlm.nih.gov/mesh/68014657)  Systemic Vasculitis  [Anti-Neutrophil Cytoplasmic Antibody-Associated Vasculitis](https://www.ncbi.nlm.nih.gov/mesh/68056648)  [Churg-Strauss Syndrome](https://www.ncbi.nlm.nih.gov/mesh/68015267)  [Granulomatosis with Polyangiitis](https://www.ncbi.nlm.nih.gov/mesh/68014890)  [Microscopic Polyangiitis](https://www.ncbi.nlm.nih.gov/mesh/68055953)  [Polyarteritis Nodosa](https://www.ncbi.nlm.nih.gov/mesh/68010488)  [Rheumatoid Vasculitis](https://www.ncbi.nlm.nih.gov/mesh/68056653) |
|  | [Sjogren's Syndrome - MeSH - NCBI “nih.gov)](https://www.ncbi.nlm.nih.gov/mesh/68012859)  Chronic inflammatory and autoimmune disease in which the salivary and lacrimal glands undergo progressive destruction by lymphocytes and plasma cells resulting in decreased production of saliva and tears. The primary form, often called sicca syndrome, involves both KERATOCONJUNCTIVITIS SICCA and XEROSTOMIA. The secondary form includes, in addition, the presence of a connective tissue disease, usually rheumatoid arthritis. | - Sjogrens Syndrome - Sjogren Syndrome - Sicca Syndrome | "[Sjogren's Syndrome](https://www.ncbi.nlm.nih.gov/mesh/68012859)" [Mesh] OR "[Sjogren's Syndrome](https://www.ncbi.nlm.nih.gov/mesh/68012859)"  OR "Sjogrens Syndrome" OR "Sjogren Syndrome" OR "Sicca Syndrome"  [Arthritis](https://www.ncbi.nlm.nih.gov/mesh/68001168)  [Arthritis, Rheumatoid](https://www.ncbi.nlm.nih.gov/mesh/68001172)  Sjogren's Syndrome |
|  | [Scleroderma, Systemic - MeSH - NCBI “nih.gov)](https://www.ncbi.nlm.nih.gov/mesh/68012595)  A chronic multi-system disorder of CONNECTIVE TISSUE. It is characterized by SCLEROSIS in the SKIN, the LUNGS, the HEART, the GASTROINTESTINAL TRACT, the KIDNEYS, and the MUSCULOSKELETAL SYSTEM. Other important features include diseased small BLOOD VESSELS and AUTOANTIBODIES. The disorder is named for its most prominent feature “hard skin), and classified into subsets by the extent of skin thickening: LIMITED SCLERODERMA and DIFFUSE SCLERODERMA. | - Systemic Sclerosis - Systemic Scleroderma | "[Scleroderma, Systemic](https://www.ncbi.nlm.nih.gov/mesh/68012595)" [Mesh] OR "[Scleroderma, Systemic](https://www.ncbi.nlm.nih.gov/mesh/68012595)" OR "Systemic Sclerosis" OR "Systemic Scleroderma"  [Connective Tissue Diseases](https://www.ncbi.nlm.nih.gov/mesh/68003240)  Scleroderma, Systemic  [Scleroderma, Diffuse](https://www.ncbi.nlm.nih.gov/mesh/68045743)  [Scleroderma, Limited](https://www.ncbi.nlm.nih.gov/mesh/68045745)  [CREST Syndrome](https://www.ncbi.nlm.nih.gov/mesh/68017675) |
|  | [Mixed Connective Tissue Disease - MeSH - NCBI “nih.gov)](https://www.ncbi.nlm.nih.gov/mesh/?term=Mixed+connective+tissue+disease)  A syndrome with overlapping clinical features of systemic lupus erythematosus, scleroderma, polymyositis, and Raynaud's phenomenon. The disease is differentially characterized by high serum titers of antibodies to ribonuclease-sensitive extractable “saline soluble) nuclear antigen and a "speckled" epidermal nuclear staining pattern on direct immunofluorescence. | - Sharp Syndrome - MCTD | "[Mixed Connective Tissue Disease](https://www.ncbi.nlm.nih.gov/mesh/?term=Mixed+connective+tissue+disease)" [Mesh] OR "[Mixed Connective Tissue Disease](https://www.ncbi.nlm.nih.gov/mesh/?term=Mixed+connective+tissue+disease)" OR "Sharp Syndrome" OR "MCTD"  [Connective Tissue Diseases](https://www.ncbi.nlm.nih.gov/mesh/68003240)  Mixed Connective Tissue Disease |
| Intervention | [Influenza Vaccines - MeSH - NCBI “nih.gov)](https://www.ncbi.nlm.nih.gov/mesh/68007252)  Vaccines used to prevent infection by viruses in the family ORTHOMYXOVIRIDAE. It includes both killed and attenuated vaccines. The composition of the vaccines is changed each year in response to antigenic shifts and changes in prevalence of influenza virus strains. The flu vaccines may be mono- or multi-valent, which contains one or more INFLUENZAVIRUS A and INFLUENZAVIRUS B strains. | - Influenza Vaccine - Influenzavirus Vaccines - Influenza Virus Vaccines - Influenzavirus Vaccine - Influenza Virus Vaccine - High-Dose Trivalent Influenza Vaccine - High Dose Trivalent Influenza Vaccine - Trivalent Influenza Vaccine - Quadrivalent Influenza Vaccine - High dose | "[Influenza Vaccines](https://www.ncbi.nlm.nih.gov/mesh/68007252)" [Mesh:NoExp] OR Influenza Vaccine" OR "Influenzavirus Vaccines" OR "Influenza Virus Vaccines" OR "Influenzavirus Vaccine" OR "Influenza Virus Vaccine" OR "High-Dose Trivalent Influenza Vaccine" OR "High Dose Trivalent Influenza Vaccine" OR "Trivalent Influenza Vaccine" OR "Quadrivalent Influenza Vaccine"  “não utilizar [Mesh] pois a árvore do termo inclui VVA e intra-nasal)  [Vaccines](https://www.ncbi.nlm.nih.gov/mesh/68014612)  [Viral Vaccines](https://www.ncbi.nlm.nih.gov/mesh/68014765)  Influenza Vaccines |

# Question 11.  Is there a difference in the immune response between conventional “tri- and tetravalent) and high-dose Hepatitis B Vaccines in patients with immune-mediated rheumatic diseases “IMRD)?

| **Acronym** | **MeSH Terms**  **and definitions** | **Entry Terms** | [**MeSH Categories**](https://www.ncbi.nlm.nih.gov/mesh/1000048) |
| --- | --- | --- | --- |
| Population  Patients with immune-mediated rheumatic diseases “IMRD) | [Rheumatic Diseases - MeSH - NCBI “nih.gov)](https://www.ncbi.nlm.nih.gov/mesh/68012216)  Disorders of connective tissue, especially the joints and related structures, characterized by inflammation, degeneration, or metabolic derangement. | - Rheumatic Disease - Rheumatism | "[Rheumatic Diseases](https://www.ncbi.nlm.nih.gov/mesh/68012216)" [Mesh:NoExp] OR "Rheumatic Disease" OR "Rheumatism" OR  "Immune-mediated rheumatic diseases"  “não utilizar [Mesh] pois a árvore do termo inclui gota, fibromialgia e crianças) |
|  | [Arthritis, Rheumatoid - MeSH - NCBI “nih.gov)](https://www.ncbi.nlm.nih.gov/mesh/68001172)  A chronic systemic disease, primarily of the joints, marked by inflammatory changes in the synovial membranes and articular structures, widespread fibrinoid degeneration of the collagen fibers in mesenchymal tissues, and by atrophy and rarefaction of bony structures. Etiology is unknown, but autoimmune mechanisms have been implicated. | - Rheumatoid Arthritis | "[Arthritis, Rheumatoid](https://www.ncbi.nlm.nih.gov/mesh/68001172)" [Mesh:NoExp]  OR "Rheumatoid Arthritis" OR "[Rheumatoid Nodule](https://www.ncbi.nlm.nih.gov/mesh/68012218)" OR "[Rheumatoid Vasculitis](https://www.ncbi.nlm.nih.gov/mesh/68056653)" OR "[Sjogren's Syndrome](https://www.ncbi.nlm.nih.gov/mesh/68012859)"  “não utilizar [Mesh] pois a árvore do termo inclui caplan syndrome, felty syndrome) |
|  | [Spondylarthritis - MeSH - NCBI “nih.gov)](https://www.ncbi.nlm.nih.gov/mesh/68025241)  Inflammation of the joints of the SPINE, the intervertebral articulations. | - Spondylarthritides - Spinal Arthritis - Spinal Arthritides | "[Spondylarthritis](https://www.ncbi.nlm.nih.gov/mesh/68025241)" [Mesh] OR "[Spondylarthritis](https://www.ncbi.nlm.nih.gov/mesh/68025241)" OR "Spondylarthritides" OR "Spinal Arthritis" OR "Spinal Arthritides"  [Spondylitis](https://www.ncbi.nlm.nih.gov/mesh/68013166)  Spondylarthritis  [Osteoarthritis, Spine](https://www.ncbi.nlm.nih.gov/mesh/68055013)  [Spondylarthropathies](https://www.ncbi.nlm.nih.gov/mesh/68025242)  [Arthritis, Psoriatic](https://www.ncbi.nlm.nih.gov/mesh/68015535)  [Arthritis, Reactive](https://www.ncbi.nlm.nih.gov/mesh/68016918)  [Axial Spondyloarthritis](https://www.ncbi.nlm.nih.gov/mesh/2101123) + |
|  | [Arthritis, Psoriatic - MeSH - NCBI “nih.gov)](https://www.ncbi.nlm.nih.gov/mesh/68015535)  A type of inflammatory arthritis associated with PSORIASIS, often involving the axial joints and the peripheral terminal interphalangeal joints. It is characterized by the presence of HLA-B27-associated SPONDYLARTHROPATHY, and the absence of rheumatoid factor. | - Arthritic Psoriasis - Psoriatic Arthritis - Psoriasis Arthropathica - Psoriatic Arthropathy - Psoriatic Arthropathies | "[Arthritis, Psoriatic](https://www.ncbi.nlm.nih.gov/mesh/68015535)" [Mesh] OR "[Arthritis, Psoriatic](https://www.ncbi.nlm.nih.gov/mesh/68015535)" OR "Arthritic Psoriasis" OR "Psoriatic Arthritis" OR "Psoriasis Arthropathica" OR "Psoriatic Arthropathy" OR "Psoriatic Arthropathies"  [Spondylarthritis](https://www.ncbi.nlm.nih.gov/mesh/68025241)  [Spondylarthropathies](https://www.ncbi.nlm.nih.gov/mesh/68025242)  Arthritis, Psoriatic |
|  | [Spondylitis, Ankylosing - MeSH - NCBI “nih.gov)](https://www.ncbi.nlm.nih.gov/mesh/?term=ankylosing+spondylitis)  A chronic inflammatory condition affecting the axial joints, such as the SACROILIAC JOINT and other intervertebral or costovertebral joints. It occurs predominantly in young males and is characterized by pain and stiffness of joints “ANKYLOSIS) with inflammation at tendon insertions. | - Bechterew's Disease - Bechterews Disease - Marie Struempell Disease - Spondylarthritis Ankylopoietica - Spondyloarthritis Ankylopoietica - Ankylosing Spondylitis - Ankylosing Spondylarthritis - Ankylosing Spondylarthritides - Ankylosing Spondyloarthritis - Ankylosing Spondyloarthritides - Spondylitis Ankylopoietica - Bechterew Disease - Rheumatoid Spondylitis | "[Spondylitis, Ankylosing](https://www.ncbi.nlm.nih.gov/mesh/?term=ankylosing+spondylitis)" [Mesh] OR "[Spondylitis, Ankylosing](https://www.ncbi.nlm.nih.gov/mesh/?term=ankylosing+spondylitis)" OR "Bechterew's Disease" OR "Bechterews Disease" OR "Marie Struempell Disease" OR "Spondylarthritis Ankylopoietica" OR "Spondyloarthritis Ankylopoietica" OR "Ankylosing Spondylitis" OR "Ankylosing Spondylarthritis" OR "Ankylosing Spondylarthritides" OR "Ankylosing Spondyloarthritis" OR "Ankylosing Spondyloarthritides" OR "Spondylitis Ankylopoietica" OR "Bechterew Disease" OR "Rheumatoid Spondylitis"  [Spondylarthritis](https://www.ncbi.nlm.nih.gov/mesh/68025241)  [Spondylarthropathies](https://www.ncbi.nlm.nih.gov/mesh/68025242)  [Axial Spondyloarthritis](https://www.ncbi.nlm.nih.gov/mesh/2101123)  Spondylitis, Ankylosing |
|  | [Lupus Erythematosus, Systemic - MeSH - NCBI “nih.gov)](https://www.ncbi.nlm.nih.gov/mesh/68008180)  A chronic, relapsing, inflammatory, and often febrile multisystemic disorder of connective tissue, characterized principally by involvement of the skin, joints, kidneys, and serosal membranes. It is of unknown etiology, but is thought to represent a failure of the regulatory mechanisms of the autoimmune system. The disease is marked by a wide range of system dysfunctions, an elevated erythrocyte sedimentation rate, and the formation of LE cells in the blood or bone marrow. | - Systemic Lupus Erythematosus - Lupus Erythematosus Disseminatus - Libman Sacks Disease | "[Lupus Erythematosus, Systemic](https://www.ncbi.nlm.nih.gov/mesh/68008180)" [Mesh] OR "[Lupus Erythematosus, Systemic](https://www.ncbi.nlm.nih.gov/mesh/68008180)" OR "Systemic Lupus Erythematosus " OR "Lupus Erythematosus Disseminatus" OR "Libman Sacks Disease"  [Connective Tissue Diseases](https://www.ncbi.nlm.nih.gov/mesh/68003240)  Lupus Erythematosus, Systemic  [Lupus Nephritis](https://www.ncbi.nlm.nih.gov/mesh/68008181)  [Lupus Vasculitis, Central Nervous System](https://www.ncbi.nlm.nih.gov/mesh/68020945) |
|  | [Myositis - MeSH - NCBI “nih.gov)](https://www.ncbi.nlm.nih.gov/mesh/?term=inflammatory+myopathies)  Inflammation of a muscle or muscle tissue. | - Myositides - Inflammatory Muscle Diseases - Inflammatory Muscle Disease - Inflammatory Myopathy - Inflammatory Myopathies - Proliferative Myositides - Proliferative Myositis - Infectious Myositides - Infectious Myositis - Idiopathic Inflammatory Myopathies - Idiopathic Inflammatory Myopathy - Idiopathic Inflammatory Myositis - Focal Myositides - Focal Myositis | "[Myositis](https://www.ncbi.nlm.nih.gov/mesh/?term=inflammatory+myopathies)" [Mesh] OR "[Myositis](https://www.ncbi.nlm.nih.gov/mesh/?term=inflammatory+myopathies)" OR "Myositides" OR "Inflammatory Muscle Diseases" OR "Inflammatory Muscle Disease" OR "Inflammatory Myopathy" OR "Inflammatory Myopathies" OR "Proliferative Myositides" OR "Proliferative Myositis" OR "Infectious Myositides" OR "Infectious Myositis" OR "Idiopathic Inflammatory Myopathies" OR "Idiopathic Inflammatory Myopathy" OR "Idiopathic Inflammatory Myositis" OR "Focal Myositides" OR "Focal Myositis"  [Muscular Diseases](https://www.ncbi.nlm.nih.gov/mesh/68009135)  Myositis  [Myositis Ossificans](https://www.ncbi.nlm.nih.gov/mesh/68009221)  [Myositis, Inclusion Body](https://www.ncbi.nlm.nih.gov/mesh/68018979)  [Orbital Myositis](https://www.ncbi.nlm.nih.gov/mesh/68055622)  [Polymyositis](https://www.ncbi.nlm.nih.gov/mesh/68017285)  [Dermatomyositis](https://www.ncbi.nlm.nih.gov/mesh/68003882)  [Pyomyositis](https://www.ncbi.nlm.nih.gov/mesh/68052880) |
|  | [Systemic Vasculitis - MeSH - NCBI “nih.gov)](https://www.ncbi.nlm.nih.gov/mesh/?term=systemic+vasculitides)  A heterogeneous group of diseases characterized by inflammation and necrosis of the blood vessel walls. | - Systemic Vasculitides | "[Systemic Vasculitis](https://www.ncbi.nlm.nih.gov/mesh/?term=systemic+vasculitides)" [Mesh] OR "[Systemic Vasculitis](https://www.ncbi.nlm.nih.gov/mesh/?term=systemic+vasculitides)" OR "Systemic Vasculitides"  [Vasculitis](https://www.ncbi.nlm.nih.gov/mesh/68014657)  Systemic Vasculitis  [Anti-Neutrophil Cytoplasmic Antibody-Associated Vasculitis](https://www.ncbi.nlm.nih.gov/mesh/68056648)  [Churg-Strauss Syndrome](https://www.ncbi.nlm.nih.gov/mesh/68015267)  [Granulomatosis with Polyangiitis](https://www.ncbi.nlm.nih.gov/mesh/68014890)  [Microscopic Polyangiitis](https://www.ncbi.nlm.nih.gov/mesh/68055953)  [Polyarteritis Nodosa](https://www.ncbi.nlm.nih.gov/mesh/68010488)  [Rheumatoid Vasculitis](https://www.ncbi.nlm.nih.gov/mesh/68056653) |
|  | [Sjogren's Syndrome - MeSH - NCBI “nih.gov)](https://www.ncbi.nlm.nih.gov/mesh/68012859)  Chronic inflammatory and autoimmune disease in which the salivary and lacrimal glands undergo progressive destruction by lymphocytes and plasma cells resulting in decreased production of saliva and tears. The primary form, often called sicca syndrome, involves both KERATOCONJUNCTIVITIS SICCA and XEROSTOMIA. The secondary form includes, in addition, the presence of a connective tissue disease, usually rheumatoid arthritis. | - Sjogrens Syndrome - Sjogren Syndrome - Sicca Syndrome | "[Sjogren's Syndrome](https://www.ncbi.nlm.nih.gov/mesh/68012859)" [Mesh] OR "[Sjogren's Syndrome](https://www.ncbi.nlm.nih.gov/mesh/68012859)"  OR "Sjogrens Syndrome" OR "Sjogren Syndrome" OR "Sicca Syndrome"  [Arthritis](https://www.ncbi.nlm.nih.gov/mesh/68001168)  [Arthritis, Rheumatoid](https://www.ncbi.nlm.nih.gov/mesh/68001172)  Sjogren's Syndrome |
|  | [Scleroderma, Systemic - MeSH - NCBI “nih.gov)](https://www.ncbi.nlm.nih.gov/mesh/68012595)  A chronic multi-system disorder of CONNECTIVE TISSUE. It is characterized by SCLEROSIS in the SKIN, the LUNGS, the HEART, the GASTROINTESTINAL TRACT, the KIDNEYS, and the MUSCULOSKELETAL SYSTEM. Other important features include diseased small BLOOD VESSELS and AUTOANTIBODIES. The disorder is named for its most prominent feature “hard skin), and classified into subsets by the extent of skin thickening: LIMITED SCLERODERMA and DIFFUSE SCLERODERMA. | - Systemic Sclerosis - Systemic Scleroderma | "[Scleroderma, Systemic](https://www.ncbi.nlm.nih.gov/mesh/68012595)" [Mesh] OR "[Scleroderma, Systemic](https://www.ncbi.nlm.nih.gov/mesh/68012595)" OR "Systemic Sclerosis" OR "Systemic Scleroderma"  [Connective Tissue Diseases](https://www.ncbi.nlm.nih.gov/mesh/68003240)  Scleroderma, Systemic  [Scleroderma, Diffuse](https://www.ncbi.nlm.nih.gov/mesh/68045743)  [Scleroderma, Limited](https://www.ncbi.nlm.nih.gov/mesh/68045745)  [CREST Syndrome](https://www.ncbi.nlm.nih.gov/mesh/68017675) |
|  | [Mixed Connective Tissue Disease - MeSH - NCBI “nih.gov)](https://www.ncbi.nlm.nih.gov/mesh/?term=Mixed+connective+tissue+disease)  A syndrome with overlapping clinical features of systemic lupus erythematosus, scleroderma, polymyositis, and Raynaud's phenomenon. The disease is differentially characterized by high serum titers of antibodies to ribonuclease-sensitive extractable “saline soluble) nuclear antigen and a "speckled" epidermal nuclear staining pattern on direct immunofluorescence. | - Sharp Syndrome - MCTD | "[Mixed Connective Tissue Disease](https://www.ncbi.nlm.nih.gov/mesh/?term=Mixed+connective+tissue+disease)" [Mesh] OR "[Mixed Connective Tissue Disease](https://www.ncbi.nlm.nih.gov/mesh/?term=Mixed+connective+tissue+disease)" OR "Sharp Syndrome" OR "MCTD"  [Connective Tissue Diseases](https://www.ncbi.nlm.nih.gov/mesh/68003240)  Mixed Connective Tissue Disease |
| Intervention | [Hepatitis B Vaccines - MeSH - NCBI (nih.gov)](https://www.ncbi.nlm.nih.gov/mesh/68017325)  Vaccines or candidate vaccines containing inactivated hepatitis B or some of its component antigens and designed to prevent hepatitis B. Some vaccines may be recombinantly produced. | - Vaccines, Hepatitis B - Hepatitis B Vaccine - Vaccine, Hepatitis B | "Hepatitis B Vaccines" [Mesh] OR "Vaccines, Hepatitis B "OR "Hepatitis B Vaccine" OR "Vaccine, Hepatitis B" |

3. **SEARCHS STRATEGIES**

**QUESTION 1. What is the risk of symptom flare up or worsening of IMRD disease activity after vaccination with the inactivated vaccine?**

**1.1 Following there are search strategy in PubMed/MEDLINE**

**P = Patients with immune-mediated rheumatic diseases**

(Rheumatic Diseases) OR (Rheumatic Disease) OR (Rheumatism) OR (Immune-mediated rheumatic diseases) OR (Arthritis, Rheumatoid) OR (Rheumatoid Arthritis) OR (Rheumatoid Nodule) OR (Rheumatoid Vasculitis) OR (Sjogren's Syndrome) OR "Spondylarthritis" [Mesh] OR (Spondylarthritis) OR (Spondylarthritides) OR (Spinal Arthritis) OR "Arthritis, Psoriatic" [Mesh] OR (Arthritis, Psoriatic) OR (Arthritic Psoriasis) OR (Psoriatic Arthritis) OR (Psoriasis Arthropathica) OR (Psoriatic Arthropathy) OR (Psoriatic Arthropathies) OR "Spondylitis, Ankylosing" [Mesh] OR (Spondylitis, Ankylosing) OR (Bechterew's Disease) OR (Bechterews Disease) OR (Marie Struempell Disease) OR (Spondylarthritis Ankylopoietica) OR (Spondyloarthritis Ankylopoietica) OR (Ankylosing Spondylitis) OR (Ankylosing Spondylarthritis) OR (Ankylosing Spondylarthritides) OR (Ankylosing Spondyloarthritis) OR (Spondylitis Ankylopoietica) OR (Bechterew Disease) OR (Rheumatoid Spondylitis) OR "Lupus Erythematosus, Systemic" [Mesh] OR (Lupus Erythematosus, Systemic) OR (Systemic Lupus Erythematosus) OR (Lupus Erythematosus Disseminatus) OR (Libman Sacks Disease) OR "Myositis" [Mesh] OR (Myositis) OR (Myositides) OR (Inflammatory Muscle Diseases) OR (Inflammatory Muscle Disease) OR (Inflammatory Myopathy) OR (Inflammatory Myopathies) OR (Proliferative Myositis) OR (Infectious Myositides) OR (Infectious Myositis) OR (Idiopathic Inflammatory Myopathies) OR (Idiopathic Inflammatory Myopathy) OR (Idiopathic Inflammatory Myositis) OR (Focal Myositis) OR "Systemic Vasculitis" [Mesh] OR (Systemic Vasculitis) OR (Systemic Vasculitides) OR "Sjogren's Syndrome" [Mesh] OR (Sjogren's Syndrome) OR (Sjogrens Syndrome) OR (Sjogren Syndrome) OR (Sicca Syndrome) OR "Scleroderma, Systemic" [Mesh] OR (Scleroderma, Systemic) OR (Systemic Sclerosis) OR (Systemic Scleroderma) OR "Mixed Connective Tissue Disease" [Mesh] OR (Mixed Connective Tissue Disease) OR (Sharp Syndrome) OR (MCTD)

I = Vaccination with inactivated vaccine

"Vaccines, Inactivated" [Mesh] OR (Vaccines, Inactivated) OR (Inactivated Vaccines) OR (Killed Vaccine) OR (Killed Vaccines) OR (Inactivated Vaccine) OR (Influenza Vaccines) OR (Influenza Vaccine) OR (Influenzavirus Vaccines) OR (Influenza Virus Vaccines) OR (Influenzavirus Vaccine) OR (Influenza Virus Vaccine) OR (High-Dose Trivalent Influenza Vaccine) OR (High Dose Trivalent Influenza Vaccine) OR (Trivalent Influenza Vaccine) OR (Quadrivalent Influenza Vaccine) OR "Pneumococcal Vaccines" [Mesh] OR (Pneumococcal Vaccines) OR (Pneumococcal Vaccine) OR (Pneumovax) OR (Pneumococcal Polysaccharide Vaccine) OR (Pnu Imune Vaccine) OR (PnuImune Vaccine) OR (Conjugated penumococcal vaccine) OR (Herpes Zoster Vaccine) OR (Zoster Vaccine) OR (Shingles Vaccine) OR "Hepatitis B Vaccines" [Mesh] OR (Hepatitis B Vaccines) OR (Hepatitis B Vaccine) OR "Papillomavirus Vaccines" [Mesh] OR (Papillomavirus Vaccines) OR (Papillomavirus Vaccine) OR (Human Papillomavirus Vaccines) OR (HPV Vaccine) OR (Human Papilloma Virus Vaccine) OR (HPV Vaccines) OR (Human Papilloma Virus Vaccines) OR (Human Papillomavirus Vaccine) OR "Poliovirus Vaccines" [Mesh] OR (Poliovirus Vaccines) OR (Poliovirus Vaccine) OR (salk) OR "Hepatitis A Vaccines" [Mesh] OR (Hepatitis A Vaccines) OR (Hepatovirus Vaccines) OR (Hepatitis A Vaccine) OR (Vaqta) OR (Havrix) OR "Meningococcal Vaccines" [Mesh] OR (Meningococcal Vaccine) OR (Menactra) OR (Tetravalent Meningococcal Vaccine) OR (Menomune) OR (Menveo) OR (Bivalent Meningococcal Vaccine) OR (Meningococcal Polysaccharide Vaccine) OR "Diphtheria-Tetanus-Pertussis Vaccine" [Mesh] OR (Diphtheria-Tetanus-Pertussis Vaccine) OR (Diphtheria Tetanus Pertussis Vaccine) OR (DTwP Vaccine) OR (DPT Vaccine) OR (DTP Vaccine) OR (Di Te Per Vaccine) OR (Diphtheria Pertussis Tetanus Vaccine) OR (Haemophilus influenza type b polysaccharide vaccine-tetanus toxin conjugate) OR (PRP-T) OR (Hib-TT) OR (OmniHIB) OR (ActHIB)

#1 AND #2

("rheumatic diseases"[MeSH Terms] OR ("rheumatic"[All Fields] AND "diseases"[All Fields]) OR "rheumatic diseases"[All Fields] OR ("rheumatic diseases"[MeSH Terms] OR ("rheumatic"[All Fields] AND "diseases"[All Fields]) OR "rheumatic diseases"[All Fields] OR ("rheumatic"[All Fields] AND "disease"[All Fields]) OR "rheumatic disease"[All Fields]) OR ("rheumatic diseases"[MeSH Terms] OR ("rheumatic"[All Fields] AND "diseases"[All Fields]) OR "rheumatic diseases"[All Fields] OR "rheumatics"[All Fields] OR "rheumatism"[All Fields] OR "rheumatic"[All Fields] OR "rheumatisms"[All Fields]) OR ("Immune-mediated"[All Fields] AND ("rheumatic diseases"[MeSH Terms] OR ("rheumatic"[All Fields] AND "diseases"[All Fields]) OR "rheumatic diseases"[All Fields])) OR ("arthritis, rheumatoid"[MeSH Terms] OR ("arthritis"[All Fields] AND "rheumatoid"[All Fields]) OR "rheumatoid arthritis"[All Fields] OR ("arthritis"[All Fields] AND "rheumatoid"[All Fields]) OR "arthritis rheumatoid"[All Fields]) OR ("arthritis, rheumatoid"[MeSH Terms] OR ("arthritis"[All Fields] AND "rheumatoid"[All Fields]) OR "rheumatoid arthritis"[All Fields] OR ("rheumatoid"[All Fields] AND "arthritis"[All Fields])) OR ("rheumatoid nodule"[MeSH Terms] OR ("rheumatoid"[All Fields] AND "nodule"[All Fields]) OR "rheumatoid nodule"[All Fields]) OR ("rheumatoid vasculitis"[MeSH Terms] OR ("rheumatoid"[All Fields] AND "vasculitis"[All Fields]) OR "rheumatoid vasculitis"[All Fields]) OR ("Sjogren's Syndrome"[MeSH Terms] OR ("sjogren s"[All Fields] AND "syndrome"[All Fields]) OR "Sjogren's Syndrome"[All Fields]) OR "Spondylarthritis"[MeSH Terms] OR ("Spondylarthritis"[MeSH Terms] OR "Spondylarthritis"[All Fields] OR "spondylarthritides"[All Fields]) OR ("Spondylarthritis"[MeSH Terms] OR "Spondylarthritis"[All Fields] OR "spondylarthritides"[All Fields]) OR ("Spondylarthritis"[MeSH Terms] OR "Spondylarthritis"[All Fields] OR ("spinal"[All Fields] AND "arthritis"[All Fields]) OR "spinal arthritis"[All Fields]) OR "arthritis, psoriatic"[MeSH Terms] OR ("arthritis, psoriatic"[MeSH Terms] OR ("arthritis"[All Fields] AND "psoriatic"[All Fields]) OR "psoriatic arthritis"[All Fields] OR ("arthritis"[All Fields] AND "psoriatic"[All Fields]) OR "arthritis psoriatic"[All Fields]) OR ("arthritis, psoriatic"[MeSH Terms] OR ("arthritis"[All Fields] AND "psoriatic"[All Fields]) OR "psoriatic arthritis"[All Fields] OR ("arthritic"[All Fields] AND "psoriasis"[All Fields]) OR "arthritic psoriasis"[All Fields]) OR ("arthritis, psoriatic"[MeSH Terms] OR ("arthritis"[All Fields] AND "psoriatic"[All Fields]) OR "psoriatic arthritis"[All Fields] OR ("psoriatic"[All Fields] AND "arthritis"[All Fields])) OR ("arthritis, psoriatic"[MeSH Terms] OR ("arthritis"[All Fields] AND "psoriatic"[All Fields]) OR "psoriatic arthritis"[All Fields] OR ("psoriasis"[All Fields] AND "arthropathica"[All Fields]) OR "psoriasis arthropathica"[All Fields]) OR ("arthritis, psoriatic"[MeSH Terms] OR ("arthritis"[All Fields] AND "psoriatic"[All Fields]) OR "psoriatic arthritis"[All Fields] OR ("psoriatic"[All Fields] AND "arthropathy"[All Fields]) OR "psoriatic arthropathy"[All Fields]) OR ("arthritis, psoriatic"[MeSH Terms] OR ("arthritis"[All Fields] AND "psoriatic"[All Fields]) OR "psoriatic arthritis"[All Fields] OR ("psoriatic"[All Fields] AND "arthropathies"[All Fields]) OR "psoriatic arthropathies"[All Fields]) OR "spondylitis, ankylosing"[MeSH Terms] OR ("spondylitis, ankylosing"[MeSH Terms] OR ("spondylitis"[All Fields] AND "ankylosing"[All Fields]) OR "ankylosing spondylitis"[All Fields] OR ("spondylitis"[All Fields] AND "ankylosing"[All Fields]) OR "spondylitis ankylosing"[All Fields]) OR ("spondylitis, ankylosing"[MeSH Terms] OR ("spondylitis"[All Fields] AND "ankylosing"[All Fields]) OR "ankylosing spondylitis"[All Fields] OR ("bechterew s"[All Fields] AND "disease"[All Fields]) OR "bechterew s disease"[All Fields]) OR ("spondylitis, ankylosing"[MeSH Terms] OR ("spondylitis"[All Fields] AND "ankylosing"[All Fields]) OR "ankylosing spondylitis"[All Fields] OR ("bechterews"[All Fields] AND "disease"[All Fields]) OR "bechterews disease"[All Fields]) OR ("spondylitis, ankylosing"[MeSH Terms] OR ("spondylitis"[All Fields] AND "ankylosing"[All Fields]) OR "ankylosing spondylitis"[All Fields] OR ("marie"[All Fields] AND "struempell"[All Fields] AND "disease"[All Fields]) OR "marie struempell disease"[All Fields]) OR ("spondylitis, ankylosing"[MeSH Terms] OR ("spondylitis"[All Fields] AND "ankylosing"[All Fields]) OR "ankylosing spondylitis"[All Fields] OR ("Spondylarthritis"[All Fields] AND "ankylopoietica"[All Fields]) OR "spondylarthritis ankylopoietica"[All Fields]) OR ("spondylitis, ankylosing"[MeSH Terms] OR ("spondylitis"[All Fields] AND "ankylosing"[All Fields]) OR "ankylosing spondylitis"[All Fields] OR ("spondyloarthritis"[All Fields] AND "ankylopoietica"[All Fields]) OR "spondyloarthritis ankylopoietica"[All Fields]) OR ("spondylitis, ankylosing"[MeSH Terms] OR ("spondylitis"[All Fields] AND "ankylosing"[All Fields]) OR "ankylosing spondylitis"[All Fields] OR ("ankylosing"[All Fields] AND "spondylitis"[All Fields])) OR ("spondylitis, ankylosing"[MeSH Terms] OR ("spondylitis"[All Fields] AND "ankylosing"[All Fields]) OR "ankylosing spondylitis"[All Fields] OR ("ankylosing"[All Fields] AND "Spondylarthritis"[All Fields]) OR "ankylosing spondylarthritis"[All Fields]) OR ("spondylitis, ankylosing"[MeSH Terms] OR ("spondylitis"[All Fields] AND "ankylosing"[All Fields]) OR "ankylosing spondylitis"[All Fields] OR ("ankylosing"[All Fields] AND "spondylarthritides"[All Fields]) OR "ankylosing spondylarthritides"[All Fields]) OR ("spondylitis, ankylosing"[MeSH Terms] OR ("spondylitis"[All Fields] AND "ankylosing"[All Fields]) OR "ankylosing spondylitis"[All Fields] OR ("ankylosing"[All Fields] AND "spondyloarthritis"[All Fields]) OR "ankylosing spondyloarthritis"[All Fields]) OR ("spondylitis, ankylosing"[MeSH Terms] OR ("spondylitis"[All Fields] AND "ankylosing"[All Fields]) OR "ankylosing spondylitis"[All Fields] OR ("spondylitis"[All Fields] AND "ankylopoietica"[All Fields]) OR "spondylitis ankylopoietica"[All Fields]) OR ("spondylitis, ankylosing"[MeSH Terms] OR ("spondylitis"[All Fields] AND "ankylosing"[All Fields]) OR "ankylosing spondylitis"[All Fields] OR ("bechterew"[All Fields] AND "disease"[All Fields]) OR "bechterew disease"[All Fields]) OR ("spondylitis, ankylosing"[MeSH Terms] OR ("spondylitis"[All Fields] AND "ankylosing"[All Fields]) OR "ankylosing spondylitis"[All Fields] OR ("rheumatoid"[All Fields] AND "spondylitis"[All Fields]) OR "rheumatoid spondylitis"[All Fields]) OR "lupus erythematosus, systemic"[MeSH Terms] OR ("lupus erythematosus, systemic"[MeSH Terms] OR ("lupus"[All Fields] AND "erythematosus"[All Fields] AND "systemic"[All Fields]) OR "systemic lupus erythematosus"[All Fields] OR ("lupus"[All Fields] AND "erythematosus"[All Fields] AND "systemic"[All Fields]) OR "lupus erythematosus systemic"[All Fields]) OR ("lupus erythematosus, systemic"[MeSH Terms] OR ("lupus"[All Fields] AND "erythematosus"[All Fields] AND "systemic"[All Fields]) OR "systemic lupus erythematosus"[All Fields] OR ("systemic"[All Fields] AND "lupus"[All Fields] AND "erythematosus"[All Fields])) OR ("lupus erythematosus, systemic"[MeSH Terms] OR ("lupus"[All Fields] AND "erythematosus"[All Fields] AND "systemic"[All Fields]) OR "systemic lupus erythematosus"[All Fields] OR ("lupus"[All Fields] AND "erythematosus"[All Fields] AND "disseminatus"[All Fields]) OR "lupus erythematosus disseminatus"[All Fields]) OR ("lupus erythematosus, systemic"[MeSH Terms] OR ("lupus"[All Fields] AND "erythematosus"[All Fields] AND "systemic"[All Fields]) OR "systemic lupus erythematosus"[All Fields] OR ("libman"[All Fields] AND "sacks"[All Fields] AND "disease"[All Fields]) OR "libman sacks disease"[All Fields]) OR "Myositis"[MeSH Terms] OR ("Myositis"[MeSH Terms] OR "Myositis"[All Fields] OR "myositides"[All Fields]) OR ("Myositis"[MeSH Terms] OR "Myositis"[All Fields] OR "myositides"[All Fields]) OR ("Myositis"[MeSH Terms] OR "Myositis"[All Fields] OR ("inflammatory"[All Fields] AND "muscle"[All Fields] AND "diseases"[All Fields]) OR "inflammatory muscle diseases"[All Fields]) OR ("Myositis"[MeSH Terms] OR "Myositis"[All Fields] OR ("inflammatory"[All Fields] AND "muscle"[All Fields] AND "disease"[All Fields]) OR "inflammatory muscle disease"[All Fields]) OR ("Myositis"[MeSH Terms] OR "Myositis"[All Fields] OR ("inflammatory"[All Fields] AND "myopathy"[All Fields]) OR "inflammatory myopathy"[All Fields]) OR ("Myositis"[MeSH Terms] OR "Myositis"[All Fields] OR ("inflammatory"[All Fields] AND "myopathies"[All Fields]) OR "inflammatory myopathies"[All Fields]) OR ("Myositis"[MeSH Terms] OR "Myositis"[All Fields] OR ("proliferative"[All Fields] AND "Myositis"[All Fields]) OR "proliferative myositis"[All Fields]) OR ("Myositis"[MeSH Terms] OR "Myositis"[All Fields] OR ("infectious"[All Fields] AND "myositides"[All Fields]) OR "infectious myositides"[All Fields]) OR ("Myositis"[MeSH Terms] OR "Myositis"[All Fields] OR ("infectious"[All Fields] AND "Myositis"[All Fields]) OR "infectious myositis"[All Fields]) OR ("Myositis"[MeSH Terms] OR "Myositis"[All Fields] OR ("idiopathic"[All Fields] AND "inflammatory"[All Fields] AND "myopathies"[All Fields]) OR "idiopathic inflammatory myopathies"[All Fields]) OR ("Myositis"[MeSH Terms] OR "Myositis"[All Fields] OR ("idiopathic"[All Fields] AND "inflammatory"[All Fields] AND "myopathy"[All Fields]) OR "idiopathic inflammatory myopathy"[All Fields]) OR ("Myositis"[MeSH Terms] OR "Myositis"[All Fields] OR ("idiopathic"[All Fields] AND "inflammatory"[All Fields] AND "Myositis"[All Fields]) OR "idiopathic inflammatory myositis"[All Fields]) OR ("Myositis"[MeSH Terms] OR "Myositis"[All Fields] OR ("focal"[All Fields] AND "Myositis"[All Fields]) OR "focal myositis"[All Fields]) OR "Systemic Vasculitis"[MeSH Terms] OR ("Systemic Vasculitis"[MeSH Terms] OR ("systemic"[All Fields] AND "vasculitis"[All Fields]) OR "Systemic Vasculitis"[All Fields]) OR ("Systemic Vasculitis"[MeSH Terms] OR ("systemic"[All Fields] AND "vasculitis"[All Fields]) OR "Systemic Vasculitis"[All Fields] OR ("systemic"[All Fields] AND "vasculitides"[All Fields]) OR "systemic vasculitides"[All Fields]) OR "Sjogren's Syndrome"[MeSH Terms] OR ("Sjogren's Syndrome"[MeSH Terms] OR ("sjogren s"[All Fields] AND "syndrome"[All Fields]) OR "Sjogren's Syndrome"[All Fields]) OR ("Sjogren's Syndrome"[MeSH Terms] OR ("sjogren s"[All Fields] AND "syndrome"[All Fields]) OR "Sjogren's Syndrome"[All Fields] OR ("sjogrens"[All Fields] AND "syndrome"[All Fields]) OR "sjogrens syndrome"[All Fields]) OR ("Sjogren's Syndrome"[MeSH Terms] OR ("sjogren s"[All Fields] AND "syndrome"[All Fields]) OR "Sjogren's Syndrome"[All Fields] OR ("sjogren"[All Fields] AND "syndrome"[All Fields]) OR "sjogren syndrome"[All Fields]) OR ("Sjogren's Syndrome"[MeSH Terms] OR ("sjogren s"[All Fields] AND "syndrome"[All Fields]) OR "Sjogren's Syndrome"[All Fields] OR ("sicca"[All Fields] AND "syndrome"[All Fields]) OR "sicca syndrome"[All Fields]) OR "scleroderma, systemic"[MeSH Terms] OR ("scleroderma, systemic"[MeSH Terms] OR ("scleroderma"[All Fields] AND "systemic"[All Fields]) OR "systemic scleroderma"[All Fields] OR ("scleroderma"[All Fields] AND "systemic"[All Fields]) OR "scleroderma systemic"[All Fields]) OR ("scleroderma, systemic"[MeSH Terms] OR ("scleroderma"[All Fields] AND "systemic"[All Fields]) OR "systemic scleroderma"[All Fields] OR ("systemic"[All Fields] AND "sclerosis"[All Fields]) OR "systemic sclerosis"[All Fields]) OR ("scleroderma, systemic"[MeSH Terms] OR ("scleroderma"[All Fields] AND "systemic"[All Fields]) OR "systemic scleroderma"[All Fields] OR ("systemic"[All Fields] AND "scleroderma"[All Fields])) OR "Mixed Connective Tissue Disease"[MeSH Terms] OR ("Mixed Connective Tissue Disease"[MeSH Terms] OR ("mixed"[All Fields] AND "connective"[All Fields] AND "tissue"[All Fields] AND "disease"[All Fields]) OR "Mixed Connective Tissue Disease"[All Fields]) OR ("Mixed Connective Tissue Disease"[MeSH Terms] OR ("mixed"[All Fields] AND "connective"[All Fields] AND "tissue"[All Fields] AND "disease"[All Fields]) OR "Mixed Connective Tissue Disease"[All Fields] OR ("sharp"[All Fields] AND "syndrome"[All Fields]) OR "sharp syndrome"[All Fields]) OR ("Mixed Connective Tissue Disease"[MeSH Terms] OR ("mixed"[All Fields] AND "connective"[All Fields] AND "tissue"[All Fields] AND "disease"[All Fields]) OR "Mixed Connective Tissue Disease"[All Fields] OR "mctd"[All Fields])) AND ("vaccines, inactivated"[MeSH Terms] OR ("vaccines, inactivated"[MeSH Terms] OR ("vaccines"[All Fields] AND "inactivated"[All Fields]) OR "inactivated vaccines"[All Fields] OR ("vaccines"[All Fields] AND "inactivated"[All Fields]) OR "vaccines inactivated"[All Fields]) OR ("vaccines, inactivated"[MeSH Terms] OR ("vaccines"[All Fields] AND "inactivated"[All Fields]) OR "inactivated vaccines"[All Fields] OR ("inactivated"[All Fields] AND "vaccines"[All Fields])) OR ("vaccines, inactivated"[MeSH Terms] OR ("vaccines"[All Fields] AND "inactivated"[All Fields]) OR "inactivated vaccines"[All Fields] OR ("killed"[All Fields] AND "vaccine"[All Fields]) OR "killed vaccine"[All Fields]) OR ("vaccines, inactivated"[MeSH Terms] OR ("vaccines"[All Fields] AND "inactivated"[All Fields]) OR "inactivated vaccines"[All Fields] OR ("killed"[All Fields] AND "vaccines"[All Fields]) OR "killed vaccines"[All Fields]) OR ("vaccines, inactivated"[MeSH Terms] OR ("vaccines"[All Fields] AND "inactivated"[All Fields]) OR "inactivated vaccines"[All Fields] OR ("inactivated"[All Fields] AND "vaccine"[All Fields]) OR "inactivated vaccine"[All Fields]) OR ("influenza vaccines"[MeSH Terms] OR ("influenza"[All Fields] AND "vaccines"[All Fields]) OR "influenza vaccines"[All Fields]) OR ("influenza vaccines"[MeSH Terms] OR ("influenza"[All Fields] AND "vaccines"[All Fields]) OR "influenza vaccines"[All Fields] OR ("influenza"[All Fields] AND "vaccine"[All Fields]) OR "influenza vaccine"[All Fields]) OR ("influenza vaccines"[MeSH Terms] OR ("influenza"[All Fields] AND "vaccines"[All Fields]) OR "influenza vaccines"[All Fields] OR ("influenzavirus"[All Fields] AND "vaccines"[All Fields])) OR ("influenza vaccines"[MeSH Terms] OR ("influenza"[All Fields] AND "vaccines"[All Fields]) OR "influenza vaccines"[All Fields] OR ("influenza"[All Fields] AND "virus"[All Fields] AND "vaccines"[All Fields]) OR "influenza virus vaccines"[All Fields]) OR ("influenza vaccines"[MeSH Terms] OR ("influenza"[All Fields] AND "vaccines"[All Fields]) OR "influenza vaccines"[All Fields] OR ("influenzavirus"[All Fields] AND "vaccine"[All Fields]) OR "influenzavirus vaccine"[All Fields]) OR ("influenza vaccines"[MeSH Terms] OR ("influenza"[All Fields] AND "vaccines"[All Fields]) OR "influenza vaccines"[All Fields] OR ("influenza"[All Fields] AND "virus"[All Fields] AND "vaccine"[All Fields]) OR "influenza virus vaccine"[All Fields]) OR ("influenza vaccines"[MeSH Terms] OR ("influenza"[All Fields] AND "vaccines"[All Fields]) OR "influenza vaccines"[All Fields] OR ("high"[All Fields] AND "dose"[All Fields] AND "trivalent"[All Fields] AND "influenza"[All Fields] AND "vaccine"[All Fields]) OR "high dose trivalent influenza vaccine"[All Fields]) OR ("influenza vaccines"[MeSH Terms] OR ("influenza"[All Fields] AND "vaccines"[All Fields]) OR "influenza vaccines"[All Fields] OR ("high"[All Fields] AND "dose"[All Fields] AND "trivalent"[All Fields] AND "influenza"[All Fields] AND "vaccine"[All Fields]) OR "high dose trivalent influenza vaccine"[All Fields]) OR ("influenza vaccines"[MeSH Terms] OR ("influenza"[All Fields] AND "vaccines"[All Fields]) OR "influenza vaccines"[All Fields] OR ("trivalent"[All Fields] AND "influenza"[All Fields] AND "vaccine"[All Fields]) OR "trivalent influenza vaccine"[All Fields]) OR ("influenza vaccines"[MeSH Terms] OR ("influenza"[All Fields] AND "vaccines"[All Fields]) OR "influenza vaccines"[All Fields] OR ("quadrivalent"[All Fields] AND "influenza"[All Fields] AND "vaccine"[All Fields]) OR "quadrivalent influenza vaccine"[All Fields]) OR "Pneumococcal Vaccines"[MeSH Terms] OR ("Pneumococcal Vaccines"[MeSH Terms] OR ("pneumococcal"[All Fields] AND "vaccines"[All Fields]) OR "Pneumococcal Vaccines"[All Fields]) OR ("Pneumococcal Vaccines"[MeSH Terms] OR ("pneumococcal"[All Fields] AND "vaccines"[All Fields]) OR "Pneumococcal Vaccines"[All Fields] OR ("pneumococcal"[All Fields] AND "vaccine"[All Fields]) OR "pneumococcal vaccine"[All Fields]) OR ("Pneumococcal Vaccines"[MeSH Terms] OR ("pneumococcal"[All Fields] AND "vaccines"[All Fields]) OR "Pneumococcal Vaccines"[All Fields] OR "pneumovax"[All Fields]) OR ("Pneumococcal Vaccines"[MeSH Terms] OR ("pneumococcal"[All Fields] AND "vaccines"[All Fields]) OR "Pneumococcal Vaccines"[All Fields] OR ("pneumococcal"[All Fields] AND "polysaccharide"[All Fields] AND "vaccine"[All Fields]) OR "pneumococcal polysaccharide vaccine"[All Fields]) OR ("Pneumococcal Vaccines"[MeSH Terms] OR ("pneumococcal"[All Fields] AND "vaccines"[All Fields]) OR "Pneumococcal Vaccines"[All Fields] OR ("pnu"[All Fields] AND "imune"[All Fields] AND "vaccine"[All Fields]) OR "pnu imune vaccine"[All Fields]) OR ("Pneumococcal Vaccines"[MeSH Terms] OR ("pneumococcal"[All Fields] AND "vaccines"[All Fields]) OR "Pneumococcal Vaccines"[All Fields] OR ("pnuimune"[All Fields] AND "vaccine"[All Fields])) OR (("conjugable"[All Fields] OR "conjugal"[All Fields] OR "conjugally"[All Fields] OR "conjugant"[All Fields] OR "conjugants"[All Fields] OR "conjugate"[All Fields] OR "conjugate s"[All Fields] OR "conjugated"[All Fields] OR "conjugates"[All Fields] OR "conjugating"[All Fields] OR "conjugation"[All Fields] OR "conjugational"[All Fields] OR "conjugations"[All Fields] OR "conjugative"[All Fields] OR "conjugatively"[All Fields]) AND "penumococcal"[All Fields] AND ("vaccin"[Supplementary Concept] OR "vaccin"[All Fields] OR "vaccination"[MeSH Terms] OR "vaccination"[All Fields] OR "vaccinable"[All Fields] OR "vaccinal"[All Fields] OR "vaccinate"[All Fields] OR "vaccinated"[All Fields] OR "vaccinates"[All Fields] OR "vaccinating"[All Fields] OR "vaccinations"[All Fields] OR "vaccination s"[All Fields] OR "vaccinator"[All Fields] OR "vaccinators"[All Fields] OR "vaccine s"[All Fields] OR "vaccined"[All Fields] OR "vaccines"[MeSH Terms] OR "vaccines"[All Fields] OR "vaccine"[All Fields] OR "vaccins"[All Fields])) OR ("herpes zoster vaccine"[MeSH Terms] OR ("herpes"[All Fields] AND "zoster"[All Fields] AND "vaccine"[All Fields]) OR "herpes zoster vaccine"[All Fields]) OR ("herpes zoster vaccine"[MeSH Terms] OR ("herpes"[All Fields] AND "zoster"[All Fields] AND "vaccine"[All Fields]) OR "herpes zoster vaccine"[All Fields] OR ("zoster"[All Fields] AND "vaccine"[All Fields]) OR "zoster vaccine"[All Fields]) OR ("herpes zoster vaccine"[MeSH Terms] OR ("herpes"[All Fields] AND "zoster"[All Fields] AND "vaccine"[All Fields]) OR "herpes zoster vaccine"[All Fields] OR ("shingles"[All Fields] AND "vaccine"[All Fields]) OR "shingles vaccine"[All Fields]) OR "Hepatitis B Vaccines"[MeSH Terms] OR ("Hepatitis B Vaccines"[MeSH Terms] OR "Hepatitis B Vaccines"[All Fields]) OR ("Hepatitis B Vaccines"[MeSH Terms] OR "Hepatitis B Vaccines"[All Fields] OR "hepatitis b vaccine"[All Fields]) OR "Papillomavirus Vaccines"[MeSH Terms] OR ("Papillomavirus Vaccines"[MeSH Terms] OR ("papillomavirus"[All Fields] AND "vaccines"[All Fields]) OR "Papillomavirus Vaccines"[All Fields]) OR ("Papillomavirus Vaccines"[MeSH Terms] OR ("papillomavirus"[All Fields] AND "vaccines"[All Fields]) OR "Papillomavirus Vaccines"[All Fields] OR ("papillomavirus"[All Fields] AND "vaccine"[All Fields]) OR "papillomavirus vaccine"[All Fields]) OR ("Papillomavirus Vaccines"[MeSH Terms] OR ("papillomavirus"[All Fields] AND "vaccines"[All Fields]) OR "Papillomavirus Vaccines"[All Fields] OR ("human"[All Fields] AND "papillomavirus"[All Fields] AND "vaccines"[All Fields]) OR "human papillomavirus vaccines"[All Fields]) OR ("Papillomavirus Vaccines"[MeSH Terms] OR ("papillomavirus"[All Fields] AND "vaccines"[All Fields]) OR "Papillomavirus Vaccines"[All Fields] OR ("hpv"[All Fields] AND "vaccine"[All Fields]) OR "hpv vaccine"[All Fields]) OR ("Papillomavirus Vaccines"[MeSH Terms] OR ("papillomavirus"[All Fields] AND "vaccines"[All Fields]) OR "Papillomavirus Vaccines"[All Fields] OR ("human"[All Fields] AND "papilloma"[All Fields] AND "virus"[All Fields] AND "vaccine"[All Fields]) OR "human papilloma virus vaccine"[All Fields]) OR ("Papillomavirus Vaccines"[MeSH Terms] OR ("papillomavirus"[All Fields] AND "vaccines"[All Fields]) OR "Papillomavirus Vaccines"[All Fields] OR ("hpv"[All Fields] AND "vaccines"[All Fields]) OR "hpv vaccines"[All Fields]) OR ("Papillomavirus Vaccines"[MeSH Terms] OR ("papillomavirus"[All Fields] AND "vaccines"[All Fields]) OR "Papillomavirus Vaccines"[All Fields] OR ("human"[All Fields] AND "papilloma"[All Fields] AND "virus"[All Fields] AND "vaccines"[All Fields]) OR "human papilloma virus vaccines"[All Fields]) OR ("Papillomavirus Vaccines"[MeSH Terms] OR ("papillomavirus"[All Fields] AND "vaccines"[All Fields]) OR "Papillomavirus Vaccines"[All Fields] OR ("human"[All Fields] AND "papillomavirus"[All Fields] AND "vaccine"[All Fields]) OR "human papillomavirus vaccine"[All Fields]) OR "Poliovirus Vaccines"[MeSH Terms] OR ("Poliovirus Vaccines"[MeSH Terms] OR ("poliovirus"[All Fields] AND "vaccines"[All Fields]) OR "Poliovirus Vaccines"[All Fields]) OR ("Poliovirus Vaccines"[MeSH Terms] OR ("poliovirus"[All Fields] AND "vaccines"[All Fields]) OR "Poliovirus Vaccines"[All Fields] OR ("poliovirus"[All Fields] AND "vaccine"[All Fields]) OR "poliovirus vaccine"[All Fields]) OR "salk"[All Fields] OR "Hepatitis A Vaccines"[MeSH Terms] OR ("Hepatitis A Vaccines"[MeSH Terms] OR "Hepatitis A Vaccines"[All Fields]) OR ("Hepatitis A Vaccines"[MeSH Terms] OR "Hepatitis A Vaccines"[All Fields] OR ("hepatovirus"[All Fields] AND "vaccines"[All Fields])) OR ("Hepatitis A Vaccines"[MeSH Terms] OR "Hepatitis A Vaccines"[All Fields] OR "hepatitis a vaccine"[All Fields]) OR ("hepatitis a vaccine inactivated"[All Fields] OR "Hepatitis A Vaccines"[MeSH Terms] OR "Hepatitis A Vaccines"[All Fields] OR "vaqta"[All Fields]) OR ("Hepatitis A Vaccines"[MeSH Terms] OR "Hepatitis A Vaccines"[All Fields] OR "havrix"[All Fields]) OR "Meningococcal Vaccines"[MeSH Terms] OR ("Meningococcal Vaccines"[MeSH Terms] OR ("meningococcal"[All Fields] AND "vaccines"[All Fields]) OR "Meningococcal Vaccines"[All Fields] OR ("meningococcal"[All Fields] AND "vaccine"[All Fields]) OR "meningococcal vaccine"[All Fields]) OR ("Meningococcal Vaccines"[MeSH Terms] OR ("meningococcal"[All Fields] AND "vaccines"[All Fields]) OR "Meningococcal Vaccines"[All Fields] OR "menactra"[All Fields]) OR ("Meningococcal Vaccines"[MeSH Terms] OR ("meningococcal"[All Fields] AND "vaccines"[All Fields]) OR "Meningococcal Vaccines"[All Fields] OR ("tetravalent"[All Fields] AND "meningococcal"[All Fields] AND "vaccine"[All Fields]) OR "tetravalent meningococcal vaccine"[All Fields]) OR ("Meningococcal Vaccines"[MeSH Terms] OR ("meningococcal"[All Fields] AND "vaccines"[All Fields]) OR "Meningococcal Vaccines"[All Fields] OR "menomune"[All Fields]) OR ("Meningococcal Vaccines"[MeSH Terms] OR ("meningococcal"[All Fields] AND "vaccines"[All Fields]) OR "Meningococcal Vaccines"[All Fields] OR "menveo"[All Fields]) OR ("Meningococcal Vaccines"[MeSH Terms] OR ("meningococcal"[All Fields] AND "vaccines"[All Fields]) OR "Meningococcal Vaccines"[All Fields] OR ("bivalent"[All Fields] AND "meningococcal"[All Fields] AND "vaccine"[All Fields]) OR "bivalent meningococcal vaccine"[All Fields]) OR ("Meningococcal Vaccines"[MeSH Terms] OR ("meningococcal"[All Fields] AND "vaccines"[All Fields]) OR "Meningococcal Vaccines"[All Fields] OR ("meningococcal"[All Fields] AND "polysaccharide"[All Fields] AND "vaccine"[All Fields]) OR "meningococcal polysaccharide vaccine"[All Fields]) OR "Diphtheria-Tetanus-Pertussis Vaccine"[MeSH Terms] OR ("Diphtheria-Tetanus-Pertussis Vaccine"[MeSH Terms] OR ("diphtheria tetanus pertussis"[All Fields] AND "vaccine"[All Fields]) OR "Diphtheria-Tetanus-Pertussis Vaccine"[All Fields] OR ("diphtheria"[All Fields] AND "tetanus"[All Fields] AND "pertussis"[All Fields] AND "vaccine"[All Fields]) OR "Diphtheria-Tetanus-Pertussis Vaccine"[All Fields]) OR ("Diphtheria-Tetanus-Pertussis Vaccine"[MeSH Terms] OR ("diphtheria tetanus pertussis"[All Fields] AND "vaccine"[All Fields]) OR "Diphtheria-Tetanus-Pertussis Vaccine"[All Fields] OR ("diphtheria"[All Fields] AND "tetanus"[All Fields] AND "pertussis"[All Fields] AND "vaccine"[All Fields]) OR "Diphtheria-Tetanus-Pertussis Vaccine"[All Fields]) OR ("Diphtheria-Tetanus-Pertussis Vaccine"[MeSH Terms] OR ("diphtheria tetanus pertussis"[All Fields] AND "vaccine"[All Fields]) OR "Diphtheria-Tetanus-Pertussis Vaccine"[All Fields] OR ("dtwp"[All Fields] AND "vaccine"[All Fields]) OR "dtwp vaccine"[All Fields]) OR ("Diphtheria-Tetanus-Pertussis Vaccine"[MeSH Terms] OR ("diphtheria tetanus pertussis"[All Fields] AND "vaccine"[All Fields]) OR "Diphtheria-Tetanus-Pertussis Vaccine"[All Fields] OR ("dpt"[All Fields] AND "vaccine"[All Fields]) OR "dpt vaccine"[All Fields]) OR ("Diphtheria-Tetanus-Pertussis Vaccine"[MeSH Terms] OR ("diphtheria tetanus pertussis"[All Fields] AND "vaccine"[All Fields]) OR "Diphtheria-Tetanus-Pertussis Vaccine"[All Fields] OR ("dtp"[All Fields] AND "vaccine"[All Fields]) OR "dtp vaccine"[All Fields]) OR ("Diphtheria-Tetanus-Pertussis Vaccine"[MeSH Terms] OR ("diphtheria tetanus pertussis"[All Fields] AND "vaccine"[All Fields]) OR "Diphtheria-Tetanus-Pertussis Vaccine"[All Fields] OR ("di"[All Fields] AND "te"[All Fields] AND "per"[All Fields] AND "vaccine"[All Fields]) OR "di te per vaccine"[All Fields]) OR ("Diphtheria-Tetanus-Pertussis Vaccine"[MeSH Terms] OR ("diphtheria tetanus pertussis"[All Fields] AND "vaccine"[All Fields]) OR "Diphtheria-Tetanus-Pertussis Vaccine"[All Fields] OR ("diphtheria"[All Fields] AND "pertussis"[All Fields] AND "tetanus"[All Fields] AND "vaccine"[All Fields]) OR "diphtheria pertussis tetanus vaccine"[All Fields]) OR ("haemophilus influenza type b polysaccharide vaccine tetanus toxin conjugate"[Supplementary Concept] OR "haemophilus influenza type b polysaccharide vaccine tetanus toxin conjugate"[All Fields] OR "haemophilus influenza type b polysaccharide vaccine tetanus toxin conjugate"[All Fields]) OR ("haemophilus influenza type b polysaccharide vaccine tetanus toxin conjugate"[Supplementary Concept] OR "haemophilus influenza type b polysaccharide vaccine tetanus toxin conjugate"[All Fields] OR "prp t"[All Fields]) OR ("haemophilus influenza type b polysaccharide vaccine tetanus toxin conjugate"[Supplementary Concept] OR "haemophilus influenza type b polysaccharide vaccine tetanus toxin conjugate"[All Fields] OR "hib tt"[All Fields]) OR ("haemophilus influenza type b polysaccharide vaccine tetanus toxin conjugate"[Supplementary Concept] OR "haemophilus influenza type b polysaccharide vaccine tetanus toxin conjugate"[All Fields] OR "omnihib"[All Fields]) OR ("haemophilus influenza type b polysaccharide vaccine tetanus toxin conjugate"[Supplementary Concept] OR "haemophilus influenza type b polysaccharide vaccine tetanus toxin conjugate"[All Fields] OR "acthib"[All Fields]))

1,128 results

1.2 Following there are search strategy in Embase

No.

Query

Results

3,124

#25

#12 AND #24

149,994

#24

#13 OR #14 OR #15 OR #16 OR #17 OR #18 OR #19 OR #20 OR #21 OR #22 OR #23

36

#23

'haemophilus influenza type b polysaccharide vaccine tetanus toxin conjugate'/syn

22,584

#22

'diphtheria pertussis tetanus vaccine'/syn

8,774

#21

'meningococcal vaccines'/syn

5,982

#20

'hepatitis a vaccines'/syn

15,713

#19

'poliovirus vaccines'/syn

18,091

#18

'human papilloma virus vaccine'/syn

25,125

#17

'hepatitis b vaccine'/syn

4,441

#16

'herpes zoster vaccine'/syn

25,896

#15

'pneumococcus vaccine'/syn

48,734

#14

'influenza vaccines'/syn

10,379

#13

'inactivated vaccine'/syn

582,415

#12

#1 OR #2 OR #3 OR #4 OR #5 OR #6 OR #7 OR #8 OR #9 OR #10 OR #11

5,506

#11

'mixed connective tissue disease'/syn

43,158

#10

'systemic sclerosis'/syn

35,919

#9

'sjoegren syndrome'/syn

9,231

#8

'systemic vasculitis'/syn

52,684

#7

'myositis'/syn

141,094

#6

'systemic lupus erythematosus'/syn

40,970

#5

'ankylosing spondylitis'/syn

33,640

#4

'psoriatic arthritis'/syn

73,128

#3

'spondylarthritis'/syn

282,694

#2

'rheumatoid arthritis'/syn

56,077

#1

'rheumatic disease'/de

1.3 Following there are search strategy in Cochrane Library

ID	Search	Hits

#1	MeSH descriptor: [Rheumatic Diseases] explode all trees	20731

#2	MeSH descriptor: [Arthritis, Rheumatoid] explode all trees	7302

#3	MeSH descriptor: [Spondylarthritis] explode all trees	1823

#4	MeSH descriptor: [Arthritis, Psoriatic] explode all trees	634

#5	MeSH descriptor: [Spondylitis, Ankylosing] explode all trees	826

#6	MeSH descriptor: [Lupus Erythematosus, Systemic] explode all trees	1425

#7	MeSH descriptor: [Myositis] explode all trees	318

#8	MeSH descriptor: [Systemic Vasculitis] explode all trees	266

#9	MeSH descriptor: [Sjogren's Syndrome] explode all trees	365

#10	MeSH descriptor: [Scleroderma, Systemic] explode all trees	717

#11	MeSH descriptor: [Mixed Connective Tissue Disease] explode all trees	13

#12	#1 OR #2 OR #3 OR #4 OR #5 OR #6 OR #7 OR #8 OR #9 OR #10 OR #11	24888

#13	MeSH descriptor: [Vaccines, Inactivated] explode all trees	1148

#14	MeSH descriptor: [Influenza Vaccines] explode all trees	1853

#15	MeSH descriptor: [Pneumococcal Vaccines] explode all trees	1178

#16	MeSH descriptor: [Herpes Zoster Vaccine] explode all trees	110

#17	MeSH descriptor: [Hepatitis B Vaccines] explode all trees	1003

#18	MeSH descriptor: [Papillomavirus Vaccines] explode all trees	549

#19	MeSH descriptor: [Poliovirus Vaccines] explode all trees	544

#20	MeSH descriptor: [Hepatitis A Vaccines] explode all trees	258

#21	MeSH descriptor: [Meningococcal Vaccines] explode all trees	438

#22	MeSH descriptor: [Diphtheria-Tetanus-Pertussis Vaccine] explode all trees	585

#23	#13 OR #14 OR #15 OR #16 OR #17 OR #18 OR #19 OR #20 OR #21 OR #22	5937

#24	#12 AND #23	62

**QUESTION 2. What is the risk of infection and  symptom flare up or worsening of IMRD disease activity after vaccination with live attenuated vaccine (LAV)?**

**1.1 Following there are search strategy in PubMed/MEDLINE**

**P = Patients with immune-mediated rheumatic diseases**

(Rheumatic Diseases) OR (Rheumatic Disease) OR (Rheumatism) OR (Immune-mediated rheumatic diseases) OR (Arthritis, Rheumatoid) OR (Rheumatoid Arthritis) OR (Rheumatoid Nodule) OR (Rheumatoid Vasculitis) OR (Sjogren's Syndrome) OR "Spondylarthritis" [Mesh] OR (Spondylarthritis) OR (Spondylarthritides) OR (Spinal Arthritis) OR "Arthritis, Psoriatic" [Mesh] OR (Arthritis, Psoriatic) OR (Arthritic Psoriasis) OR (Psoriatic Arthritis) OR (Psoriasis Arthropathica) OR (Psoriatic Arthropathy) OR (Psoriatic Arthropathies) OR "Spondylitis, Ankylosing" [Mesh] OR (Spondylitis, Ankylosing) OR (Bechterew's Disease) OR (Bechterews Disease) OR (Marie Struempell Disease) OR (Spondylarthritis Ankylopoietica) OR (Spondyloarthritis Ankylopoietica) OR (Ankylosing Spondylitis) OR (Ankylosing Spondylarthritis) OR (Ankylosing Spondylarthritides) OR (Ankylosing Spondyloarthritis) OR (Spondylitis Ankylopoietica) OR (Bechterew Disease) OR (Rheumatoid Spondylitis) OR "Lupus Erythematosus, Systemic" [Mesh] OR (Lupus Erythematosus, Systemic) OR (Systemic Lupus Erythematosus) OR (Lupus Erythematosus Disseminatus) OR (Libman Sacks Disease) OR "Myositis" [Mesh] OR (Myositis) OR (Myositides) OR (Inflammatory Muscle Diseases) OR (Inflammatory Muscle Disease) OR (Inflammatory Myopathy) OR (Inflammatory Myopathies) OR (Proliferative Myositis) OR (Infectious Myositides) OR (Infectious Myositis) OR (Idiopathic Inflammatory Myopathies) OR (Idiopathic Inflammatory Myopathy) OR (Idiopathic Inflammatory Myositis) OR (Focal Myositis) OR "Systemic Vasculitis" [Mesh] OR (Systemic Vasculitis) OR (Systemic Vasculitides) OR "Sjogren's Syndrome" [Mesh] OR (Sjogren's Syndrome) OR (Sjogrens Syndrome) OR (Sjogren Syndrome) OR (Sicca Syndrome) OR "Scleroderma, Systemic" [Mesh] OR (Scleroderma, Systemic) OR (Systemic Sclerosis) OR (Systemic Scleroderma) OR "Mixed Connective Tissue Disease" [Mesh] OR (Mixed Connective Tissue Disease) OR (Sharp Syndrome) OR (MCTD)

**I = Vaccination with live attenuated vaccine (LAV)**

"[Vaccines, Live, Unattenuated](https://www.ncbi.nlm.nih.gov/mesh/68062690)" [Mesh] OR ([Vaccines, Live, Unattenuated](https://www.ncbi.nlm.nih.gov/mesh/68062690)) OR (Live Unattenuated Vaccines) OR (Unattenuated Vaccines) OR "Yellow Fever Vaccine" [Mesh] OR (Yellow Fever Vaccine) OR "Dengue Vaccines" [Mesh] OR (Dengue Vaccines) OR (Dengue Vaccine) OR "Measles-Mumps-Rubella Vaccine" [Mesh] OR (Measles-Mumps-Rubella Vaccine) OR (Measles Mumps Rubella Vaccine) OR (MMR Vaccine) OR (Mumps Measles Rubella Vaccine) OR (Triviraten Berna) OR (Priorix) OR (Trimovax) OR (Pluserix) OR (Virivac) OR **(**Chickenpox Vaccine) OR (Varicella Vaccine) OR (Oka Varicella Vaccine) OR (Varivax)

#1 AND #2

("rheumatic diseases"[MeSH Terms] OR ("rheumatic"[All Fields] AND "diseases"[All Fields]) OR "rheumatic diseases"[All Fields] OR ("rheumatic diseases"[MeSH Terms] OR ("rheumatic"[All Fields] AND "diseases"[All Fields]) OR "rheumatic diseases"[All Fields] OR ("rheumatic"[All Fields] AND "disease"[All Fields]) OR "rheumatic disease"[All Fields]) OR ("rheumatic diseases"[MeSH Terms] OR ("rheumatic"[All Fields] AND "diseases"[All Fields]) OR "rheumatic diseases"[All Fields] OR "rheumatics"[All Fields] OR "rheumatism"[All Fields] OR "rheumatic"[All Fields] OR "rheumatisms"[All Fields]) OR ("Immune-mediated"[All Fields] AND ("rheumatic diseases"[MeSH Terms] OR ("rheumatic"[All Fields] AND "diseases"[All Fields]) OR "rheumatic diseases"[All Fields])) OR ("arthritis, rheumatoid"[MeSH Terms] OR ("arthritis"[All Fields] AND "rheumatoid"[All Fields]) OR "rheumatoid arthritis"[All Fields] OR ("arthritis"[All Fields] AND "rheumatoid"[All Fields]) OR "arthritis rheumatoid"[All Fields]) OR ("arthritis, rheumatoid"[MeSH Terms] OR ("arthritis"[All Fields] AND "rheumatoid"[All Fields]) OR "rheumatoid arthritis"[All Fields] OR ("rheumatoid"[All Fields] AND "arthritis"[All Fields])) OR ("rheumatoid nodule"[MeSH Terms] OR ("rheumatoid"[All Fields] AND "nodule"[All Fields]) OR "rheumatoid nodule"[All Fields]) OR ("rheumatoid vasculitis"[MeSH Terms] OR ("rheumatoid"[All Fields] AND "vasculitis"[All Fields]) OR "rheumatoid vasculitis"[All Fields]) OR ("Sjogren's Syndrome"[MeSH Terms] OR ("sjogren s"[All Fields] AND "syndrome"[All Fields]) OR "Sjogren's Syndrome"[All Fields]) OR "Spondylarthritis"[MeSH Terms] OR ("Spondylarthritis"[MeSH Terms] OR "Spondylarthritis"[All Fields] OR "spondylarthritides"[All Fields]) OR ("Spondylarthritis"[MeSH Terms] OR "Spondylarthritis"[All Fields] OR "spondylarthritides"[All Fields]) OR ("Spondylarthritis"[MeSH Terms] OR "Spondylarthritis"[All Fields] OR ("spinal"[All Fields] AND "arthritis"[All Fields]) OR "spinal arthritis"[All Fields]) OR "arthritis, psoriatic"[MeSH Terms] OR ("arthritis, psoriatic"[MeSH Terms] OR ("arthritis"[All Fields] AND "psoriatic"[All Fields]) OR "psoriatic arthritis"[All Fields] OR ("arthritis"[All Fields] AND "psoriatic"[All Fields]) OR "arthritis psoriatic"[All Fields]) OR ("arthritis, psoriatic"[MeSH Terms] OR ("arthritis"[All Fields] AND "psoriatic"[All Fields]) OR "psoriatic arthritis"[All Fields] OR ("arthritic"[All Fields] AND "psoriasis"[All Fields]) OR "arthritic psoriasis"[All Fields]) OR ("arthritis, psoriatic"[MeSH Terms] OR ("arthritis"[All Fields] AND "psoriatic"[All Fields]) OR "psoriatic arthritis"[All Fields] OR ("psoriatic"[All Fields] AND "arthritis"[All Fields])) OR ("arthritis, psoriatic"[MeSH Terms] OR ("arthritis"[All Fields] AND "psoriatic"[All Fields]) OR "psoriatic arthritis"[All Fields] OR ("psoriasis"[All Fields] AND "arthropathica"[All Fields]) OR "psoriasis arthropathica"[All Fields]) OR ("arthritis, psoriatic"[MeSH Terms] OR ("arthritis"[All Fields] AND "psoriatic"[All Fields]) OR "psoriatic arthritis"[All Fields] OR ("psoriatic"[All Fields] AND "arthropathy"[All Fields]) OR "psoriatic arthropathy"[All Fields]) OR ("arthritis, psoriatic"[MeSH Terms] OR ("arthritis"[All Fields] AND "psoriatic"[All Fields]) OR "psoriatic arthritis"[All Fields] OR ("psoriatic"[All Fields] AND "arthropathies"[All Fields]) OR "psoriatic arthropathies"[All Fields]) OR "spondylitis, ankylosing"[MeSH Terms] OR ("spondylitis, ankylosing"[MeSH Terms] OR ("spondylitis"[All Fields] AND "ankylosing"[All Fields]) OR "ankylosing spondylitis"[All Fields] OR ("spondylitis"[All Fields] AND "ankylosing"[All Fields]) OR "spondylitis ankylosing"[All Fields]) OR ("spondylitis, ankylosing"[MeSH Terms] OR ("spondylitis"[All Fields] AND "ankylosing"[All Fields]) OR "ankylosing spondylitis"[All Fields] OR ("bechterew s"[All Fields] AND "disease"[All Fields]) OR "bechterew s disease"[All Fields]) OR ("spondylitis, ankylosing"[MeSH Terms] OR ("spondylitis"[All Fields] AND "ankylosing"[All Fields]) OR "ankylosing spondylitis"[All Fields] OR ("bechterews"[All Fields] AND "disease"[All Fields]) OR "bechterews disease"[All Fields]) OR ("spondylitis, ankylosing"[MeSH Terms] OR ("spondylitis"[All Fields] AND "ankylosing"[All Fields]) OR "ankylosing spondylitis"[All Fields] OR ("marie"[All Fields] AND "struempell"[All Fields] AND "disease"[All Fields]) OR "marie struempell disease"[All Fields]) OR ("spondylitis, ankylosing"[MeSH Terms] OR ("spondylitis"[All Fields] AND "ankylosing"[All Fields]) OR "ankylosing spondylitis"[All Fields] OR ("Spondylarthritis"[All Fields] AND "ankylopoietica"[All Fields]) OR "spondylarthritis ankylopoietica"[All Fields]) OR ("spondylitis, ankylosing"[MeSH Terms] OR ("spondylitis"[All Fields] AND "ankylosing"[All Fields]) OR "ankylosing spondylitis"[All Fields] OR ("spondyloarthritis"[All Fields] AND "ankylopoietica"[All Fields]) OR "spondyloarthritis ankylopoietica"[All Fields]) OR ("spondylitis, ankylosing"[MeSH Terms] OR ("spondylitis"[All Fields] AND "ankylosing"[All Fields]) OR "ankylosing spondylitis"[All Fields] OR ("ankylosing"[All Fields] AND "spondylitis"[All Fields])) OR ("spondylitis, ankylosing"[MeSH Terms] OR ("spondylitis"[All Fields] AND "ankylosing"[All Fields]) OR "ankylosing spondylitis"[All Fields] OR ("ankylosing"[All Fields] AND "Spondylarthritis"[All Fields]) OR "ankylosing spondylarthritis"[All Fields]) OR ("spondylitis, ankylosing"[MeSH Terms] OR ("spondylitis"[All Fields] AND "ankylosing"[All Fields]) OR "ankylosing spondylitis"[All Fields] OR ("ankylosing"[All Fields] AND "spondylarthritides"[All Fields]) OR "ankylosing spondylarthritides"[All Fields]) OR ("spondylitis, ankylosing"[MeSH Terms] OR ("spondylitis"[All Fields] AND "ankylosing"[All Fields]) OR "ankylosing spondylitis"[All Fields] OR ("ankylosing"[All Fields] AND "spondyloarthritis"[All Fields]) OR "ankylosing spondyloarthritis"[All Fields]) OR ("spondylitis, ankylosing"[MeSH Terms] OR ("spondylitis"[All Fields] AND "ankylosing"[All Fields]) OR "ankylosing spondylitis"[All Fields] OR ("spondylitis"[All Fields] AND "ankylopoietica"[All Fields]) OR "spondylitis ankylopoietica"[All Fields]) OR ("spondylitis, ankylosing"[MeSH Terms] OR ("spondylitis"[All Fields] AND "ankylosing"[All Fields]) OR "ankylosing spondylitis"[All Fields] OR ("bechterew"[All Fields] AND "disease"[All Fields]) OR "bechterew disease"[All Fields]) OR ("spondylitis, ankylosing"[MeSH Terms] OR ("spondylitis"[All Fields] AND "ankylosing"[All Fields]) OR "ankylosing spondylitis"[All Fields] OR ("rheumatoid"[All Fields] AND "spondylitis"[All Fields]) OR "rheumatoid spondylitis"[All Fields]) OR "lupus erythematosus, systemic"[MeSH Terms] OR ("lupus erythematosus, systemic"[MeSH Terms] OR ("lupus"[All Fields] AND "erythematosus"[All Fields] AND "systemic"[All Fields]) OR "systemic lupus erythematosus"[All Fields] OR ("lupus"[All Fields] AND "erythematosus"[All Fields] AND "systemic"[All Fields]) OR "lupus erythematosus systemic"[All Fields]) OR ("lupus erythematosus, systemic"[MeSH Terms] OR ("lupus"[All Fields] AND "erythematosus"[All Fields] AND "systemic"[All Fields]) OR "systemic lupus erythematosus"[All Fields] OR ("systemic"[All Fields] AND "lupus"[All Fields] AND "erythematosus"[All Fields])) OR ("lupus erythematosus, systemic"[MeSH Terms] OR ("lupus"[All Fields] AND "erythematosus"[All Fields] AND "systemic"[All Fields]) OR "systemic lupus erythematosus"[All Fields] OR ("lupus"[All Fields] AND "erythematosus"[All Fields] AND "disseminatus"[All Fields]) OR "lupus erythematosus disseminatus"[All Fields]) OR ("lupus erythematosus, systemic"[MeSH Terms] OR ("lupus"[All Fields] AND "erythematosus"[All Fields] AND "systemic"[All Fields]) OR "systemic lupus erythematosus"[All Fields] OR ("libman"[All Fields] AND "sacks"[All Fields] AND "disease"[All Fields]) OR "libman sacks disease"[All Fields]) OR "Myositis"[MeSH Terms] OR ("Myositis"[MeSH Terms] OR "Myositis"[All Fields] OR "myositides"[All Fields]) OR ("Myositis"[MeSH Terms] OR "Myositis"[All Fields] OR "myositides"[All Fields]) OR ("Myositis"[MeSH Terms] OR "Myositis"[All Fields] OR ("inflammatory"[All Fields] AND "muscle"[All Fields] AND "diseases"[All Fields]) OR "inflammatory muscle diseases"[All Fields]) OR ("Myositis"[MeSH Terms] OR "Myositis"[All Fields] OR ("inflammatory"[All Fields] AND "muscle"[All Fields] AND "disease"[All Fields]) OR "inflammatory muscle disease"[All Fields]) OR ("Myositis"[MeSH Terms] OR "Myositis"[All Fields] OR ("inflammatory"[All Fields] AND "myopathy"[All Fields]) OR "inflammatory myopathy"[All Fields]) OR ("Myositis"[MeSH Terms] OR "Myositis"[All Fields] OR ("inflammatory"[All Fields] AND "myopathies"[All Fields]) OR "inflammatory myopathies"[All Fields]) OR ("Myositis"[MeSH Terms] OR "Myositis"[All Fields] OR ("proliferative"[All Fields] AND "Myositis"[All Fields]) OR "proliferative myositis"[All Fields]) OR ("Myositis"[MeSH Terms] OR "Myositis"[All Fields] OR ("infectious"[All Fields] AND "myositides"[All Fields]) OR "infectious myositides"[All Fields]) OR ("Myositis"[MeSH Terms] OR "Myositis"[All Fields] OR ("infectious"[All Fields] AND "Myositis"[All Fields]) OR "infectious myositis"[All Fields]) OR ("Myositis"[MeSH Terms] OR "Myositis"[All Fields] OR ("idiopathic"[All Fields] AND "inflammatory"[All Fields] AND "myopathies"[All Fields]) OR "idiopathic inflammatory myopathies"[All Fields]) OR ("Myositis"[MeSH Terms] OR "Myositis"[All Fields] OR ("idiopathic"[All Fields] AND "inflammatory"[All Fields] AND "myopathy"[All Fields]) OR "idiopathic inflammatory myopathy"[All Fields]) OR ("Myositis"[MeSH Terms] OR "Myositis"[All Fields] OR ("idiopathic"[All Fields] AND "inflammatory"[All Fields] AND "Myositis"[All Fields]) OR "idiopathic inflammatory myositis"[All Fields]) OR ("Myositis"[MeSH Terms] OR "Myositis"[All Fields] OR ("focal"[All Fields] AND "Myositis"[All Fields]) OR "focal myositis"[All Fields]) OR "Systemic Vasculitis"[MeSH Terms] OR ("Systemic Vasculitis"[MeSH Terms] OR ("systemic"[All Fields] AND "vasculitis"[All Fields]) OR "Systemic Vasculitis"[All Fields]) OR ("Systemic Vasculitis"[MeSH Terms] OR ("systemic"[All Fields] AND "vasculitis"[All Fields]) OR "Systemic Vasculitis"[All Fields] OR ("systemic"[All Fields] AND "vasculitides"[All Fields]) OR "systemic vasculitides"[All Fields]) OR "Sjogren's Syndrome"[MeSH Terms] OR ("Sjogren's Syndrome"[MeSH Terms] OR ("sjogren s"[All Fields] AND "syndrome"[All Fields]) OR "Sjogren's Syndrome"[All Fields]) OR ("Sjogren's Syndrome"[MeSH Terms] OR ("sjogren s"[All Fields] AND "syndrome"[All Fields]) OR "Sjogren's Syndrome"[All Fields] OR ("sjogrens"[All Fields] AND "syndrome"[All Fields]) OR "sjogrens syndrome"[All Fields]) OR ("Sjogren's Syndrome"[MeSH Terms] OR ("sjogren s"[All Fields] AND "syndrome"[All Fields]) OR "Sjogren's Syndrome"[All Fields] OR ("sjogren"[All Fields] AND "syndrome"[All Fields]) OR "sjogren syndrome"[All Fields]) OR ("Sjogren's Syndrome"[MeSH Terms] OR ("sjogren s"[All Fields] AND "syndrome"[All Fields]) OR "Sjogren's Syndrome"[All Fields] OR ("sicca"[All Fields] AND "syndrome"[All Fields]) OR "sicca syndrome"[All Fields]) OR "scleroderma, systemic"[MeSH Terms] OR ("scleroderma, systemic"[MeSH Terms] OR ("scleroderma"[All Fields] AND "systemic"[All Fields]) OR "systemic scleroderma"[All Fields] OR ("scleroderma"[All Fields] AND "systemic"[All Fields]) OR "scleroderma systemic"[All Fields]) OR ("scleroderma, systemic"[MeSH Terms] OR ("scleroderma"[All Fields] AND "systemic"[All Fields]) OR "systemic scleroderma"[All Fields] OR ("systemic"[All Fields] AND "sclerosis"[All Fields]) OR "systemic sclerosis"[All Fields]) OR ("scleroderma, systemic"[MeSH Terms] OR ("scleroderma"[All Fields] AND "systemic"[All Fields]) OR "systemic scleroderma"[All Fields] OR ("systemic"[All Fields] AND "scleroderma"[All Fields])) OR "Mixed Connective Tissue Disease"[MeSH Terms] OR ("Mixed Connective Tissue Disease"[MeSH Terms] OR ("mixed"[All Fields] AND "connective"[All Fields] AND "tissue"[All Fields] AND "disease"[All Fields]) OR "Mixed Connective Tissue Disease"[All Fields]) OR ("Mixed Connective Tissue Disease"[MeSH Terms] OR ("mixed"[All Fields] AND "connective"[All Fields] AND "tissue"[All Fields] AND "disease"[All Fields]) OR "Mixed Connective Tissue Disease"[All Fields] OR ("sharp"[All Fields] AND "syndrome"[All Fields]) OR "sharp syndrome"[All Fields]) OR ("Mixed Connective Tissue Disease"[MeSH Terms] OR ("mixed"[All Fields] AND "connective"[All Fields] AND "tissue"[All Fields] AND "disease"[All Fields]) OR "Mixed Connective Tissue Disease"[All Fields] OR "mctd"[All Fields])) AND ("vaccines, live, unattenuated"[MeSH Terms] OR ("vaccines, live, unattenuated"[MeSH Terms] OR ("vaccines"[All Fields] AND "live"[All Fields] AND "unattenuated"[All Fields]) OR "unattenuated live vaccines"[All Fields] OR ("vaccines"[All Fields] AND "live"[All Fields] AND "unattenuated"[All Fields]) OR "vaccines live unattenuated"[All Fields]) OR ("vaccines, live, unattenuated"[MeSH Terms] OR ("vaccines"[All Fields] AND "live"[All Fields] AND "unattenuated"[All Fields]) OR "unattenuated live vaccines"[All Fields] OR ("live"[All Fields] AND "unattenuated"[All Fields] AND "vaccines"[All Fields]) OR "live unattenuated vaccines"[All Fields]) OR ("vaccines, live, unattenuated"[MeSH Terms] OR ("vaccines"[All Fields] AND "live"[All Fields] AND "unattenuated"[All Fields]) OR "unattenuated live vaccines"[All Fields] OR ("unattenuated"[All Fields] AND "vaccines"[All Fields]) OR "unattenuated vaccines"[All Fields]) OR "Yellow Fever Vaccine"[MeSH Terms] OR ("Yellow Fever Vaccine"[MeSH Terms] OR ("yellow"[All Fields] AND "fever"[All Fields] AND "vaccine"[All Fields]) OR "Yellow Fever Vaccine"[All Fields]) OR "Dengue Vaccines"[MeSH Terms] OR ("Dengue Vaccines"[MeSH Terms] OR ("dengue"[All Fields] AND "vaccines"[All Fields]) OR "Dengue Vaccines"[All Fields]) OR ("Dengue Vaccines"[MeSH Terms] OR ("dengue"[All Fields] AND "vaccines"[All Fields]) OR "Dengue Vaccines"[All Fields] OR ("dengue"[All Fields] AND "vaccine"[All Fields]) OR "dengue vaccine"[All Fields]) OR "Measles-Mumps-Rubella Vaccine"[MeSH Terms] OR ("Measles-Mumps-Rubella Vaccine"[MeSH Terms] OR ("measles mumps rubella"[All Fields] AND "vaccine"[All Fields]) OR "Measles-Mumps-Rubella Vaccine"[All Fields] OR ("measles"[All Fields] AND "mumps"[All Fields] AND "rubella"[All Fields] AND "vaccine"[All Fields]) OR "Measles-Mumps-Rubella Vaccine"[All Fields]) OR ("Measles-Mumps-Rubella Vaccine"[MeSH Terms] OR ("measles mumps rubella"[All Fields] AND "vaccine"[All Fields]) OR "Measles-Mumps-Rubella Vaccine"[All Fields] OR ("measles"[All Fields] AND "mumps"[All Fields] AND "rubella"[All Fields] AND "vaccine"[All Fields]) OR "Measles-Mumps-Rubella Vaccine"[All Fields]) OR ("Measles-Mumps-Rubella Vaccine"[MeSH Terms] OR ("measles mumps rubella"[All Fields] AND "vaccine"[All Fields]) OR "Measles-Mumps-Rubella Vaccine"[All Fields] OR ("mmr"[All Fields] AND "vaccine"[All Fields]) OR "mmr vaccine"[All Fields]) OR ("Measles-Mumps-Rubella Vaccine"[MeSH Terms] OR ("measles mumps rubella"[All Fields] AND "vaccine"[All Fields]) OR "Measles-Mumps-Rubella Vaccine"[All Fields] OR ("mumps"[All Fields] AND "measles"[All Fields] AND "rubella"[All Fields] AND "vaccine"[All Fields]) OR "mumps measles rubella vaccine"[All Fields]) OR ("Measles-Mumps-Rubella Vaccine"[MeSH Terms] OR ("measles mumps rubella"[All Fields] AND "vaccine"[All Fields]) OR "Measles-Mumps-Rubella Vaccine"[All Fields] OR ("triviraten"[All Fields] AND "berna"[All Fields]) OR "triviraten berna"[All Fields]) OR ("Measles-Mumps-Rubella Vaccine"[MeSH Terms] OR ("measles mumps rubella"[All Fields] AND "vaccine"[All Fields]) OR "Measles-Mumps-Rubella Vaccine"[All Fields] OR "priorix"[All Fields]) OR ("Measles-Mumps-Rubella Vaccine"[MeSH Terms] OR ("measles mumps rubella"[All Fields] AND "vaccine"[All Fields]) OR "Measles-Mumps-Rubella Vaccine"[All Fields] OR "trimovax"[All Fields]) OR ("Measles-Mumps-Rubella Vaccine"[MeSH Terms] OR ("measles mumps rubella"[All Fields] AND "vaccine"[All Fields]) OR "Measles-Mumps-Rubella Vaccine"[All Fields] OR "pluserix"[All Fields]) OR ("Measles-Mumps-Rubella Vaccine"[MeSH Terms] OR ("measles mumps rubella"[All Fields] AND "vaccine"[All Fields]) OR "Measles-Mumps-Rubella Vaccine"[All Fields] OR "virivac"[All Fields]) OR ("chickenpox vaccine"[MeSH Terms] OR ("chickenpox"[All Fields] AND "vaccine"[All Fields]) OR "chickenpox vaccine"[All Fields]) OR ("chickenpox vaccine"[MeSH Terms] OR ("chickenpox"[All Fields] AND "vaccine"[All Fields]) OR "chickenpox vaccine"[All Fields] OR ("varicella"[All Fields] AND "vaccine"[All Fields]) OR "varicella vaccine"[All Fields]) OR ("chickenpox vaccine"[MeSH Terms] OR ("chickenpox"[All Fields] AND "vaccine"[All Fields]) OR "chickenpox vaccine"[All Fields] OR ("oka"[All Fields] AND "varicella"[All Fields] AND "vaccine"[All Fields]) OR "oka varicella vaccine"[All Fields]) OR ("chickenpox vaccine"[MeSH Terms] OR ("chickenpox"[All Fields] AND "vaccine"[All Fields]) OR "chickenpox vaccine"[All Fields] OR "varivax"[All Fields]))

181 results

**1.2 Following there are search strategy in Embase**

**No.**

**Query**

**Results**

663

**#19**

**#12** AND **#18**

37,396

**#18**

**#13** OR **#14** OR **#15** OR **#16** OR **#17**

5,857

**#17**

**'chickenpox vaccine'**/syn

9,159

**#16**

**'measles mumps rubella vaccine'**/syn

2,954

**#15**

**'dengue vaccine'**/syn

3,234

**#14**

**'yellow fever vaccine'**/syn

20,877

**#13**

**'live vaccine'**/syn

582,415

**#12**

**#1** OR **#2** OR **#3** OR **#4** OR **#5** OR **#6** OR **#7** OR **#8** OR **#9** OR **#10** OR **#11**

5,506

**#11**

**'mixed connective tissue disease'**/syn

43,158

**#10**

**'systemic sclerosis'**/syn

35,919

**#9**

**'sjoegren syndrome'**/syn

9,231

**#8**

**'systemic vasculitis'**/syn

52,684

**#7**

**'myositis'**/syn

141,094

**#6**

**'systemic lupus erythematosus'**/syn

40,970

**#5**

**'ankylosing spondylitis'**/syn

33,640

**#4**

**'psoriatic arthritis'**/syn

73,128

**#3**

**'spondylarthritis'**/syn

282,694

**#2**

**'rheumatoid arthritis'**/syn

56,077

**#1**

**'rheumatic disease'**/de

**1.3 Following there are search strategy in Cochrane Library**

ID	Search	Hits

#1	MeSH descriptor: [Rheumatic Diseases] explode all trees	20731

#2	MeSH descriptor: [Arthritis, Rheumatoid] explode all trees	7302

#3	MeSH descriptor: [Spondylarthritis] explode all trees	1823

#4	MeSH descriptor: [Arthritis, Psoriatic] explode all trees	634

#5	MeSH descriptor: [Spondylitis, Ankylosing] explode all trees	826

#6	MeSH descriptor: [Lupus Erythematosus, Systemic] explode all trees	1425

#7	MeSH descriptor: [Myositis] explode all trees	318

#8	MeSH descriptor: [Systemic Vasculitis] explode all trees	266

#9	MeSH descriptor: [Sjogren's Syndrome] explode all trees	365

#10	MeSH descriptor: [Scleroderma, Systemic] explode all trees	717

#11	MeSH descriptor: [Mixed Connective Tissue Disease] explode all trees	13

#12	#1 OR #2 OR #3 OR #4 OR #5 OR #6 OR #7 OR #8 OR #9 OR #10 OR #11	24888

#13	MeSH descriptor: [Vaccines, Attenuated] explode all trees	870

#14	MeSH descriptor: [Yellow Fever Vaccine] explode all trees	46

#15	MeSH descriptor: [Dengue Vaccines] explode all trees	110

#16	MeSH descriptor: [Measles-Mumps-Rubella Vaccine] explode all trees	200

#17	MeSH descriptor: [Chickenpox Vaccine] explode all trees	253

#18	#13 OR #14 OR #15 OR #16 OR #17	1273

#19	#12 AND #18	    9

**QUESTION 03. What is the impact of the cs-DMARDs (MTX, LEF, SZL, HXQ) use on the response to inactivated vaccines in patients with immune-mediated rheumatic diseases?**

**Research Algorithms**

1.1 Following there are search strategy in PubMed/MEDLINE

**P = Patients with immune-mediated rheumatic diseases**

(Rheumatic Diseases) OR (Rheumatic Disease) OR (Rheumatism) OR (Immune-mediated rheumatic diseases) OR (Arthritis, Rheumatoid) OR (Rheumatoid Arthritis) OR (Rheumatoid Nodule) OR (Rheumatoid Vasculitis) OR (Sjogren's Syndrome) OR "Spondylarthritis" [Mesh] OR (Spondylarthritis) OR (Spondylarthritides) OR (Spinal Arthritis) OR "Arthritis, Psoriatic" [Mesh] OR (Arthritis, Psoriatic) OR (Arthritic Psoriasis) OR (Psoriatic Arthritis) OR (Psoriasis Arthropathica) OR (Psoriatic Arthropathy) OR (Psoriatic Arthropathies) OR "Spondylitis, Ankylosing" [Mesh] OR (Spondylitis, Ankylosing) OR (Bechterew's Disease) OR (Bechterews Disease) OR (Marie Struempell Disease) OR (Spondylarthritis Ankylopoietica) OR (Spondyloarthritis Ankylopoietica) OR (Ankylosing Spondylitis) OR (Ankylosing Spondylarthritis) OR (Ankylosing Spondylarthritides) OR (Ankylosing Spondyloarthritis) OR (Spondylitis Ankylopoietica) OR (Bechterew Disease) OR (Rheumatoid Spondylitis) OR "Lupus Erythematosus, Systemic" [Mesh] OR (Lupus Erythematosus, Systemic) OR (Systemic Lupus Erythematosus) OR (Lupus Erythematosus Disseminatus) OR (Libman Sacks Disease) OR "Myositis" [Mesh] OR (Myositis) OR (Myositides) OR (Inflammatory Muscle Diseases) OR (Inflammatory Muscle Disease) OR (Inflammatory Myopathy) OR (Inflammatory Myopathies) OR (Proliferative Myositis) OR (Infectious Myositides) OR (Infectious Myositis) OR (Idiopathic Inflammatory Myopathies) OR (Idiopathic Inflammatory Myopathy) OR (Idiopathic Inflammatory Myositis) OR (Focal Myositis) OR "Systemic Vasculitis" [Mesh] OR (Systemic Vasculitis) OR (Systemic Vasculitides) OR "Sjogren's Syndrome" [Mesh] OR (Sjogren's Syndrome) OR (Sjogrens Syndrome) OR (Sjogren Syndrome) OR (Sicca Syndrome) OR "Scleroderma, Systemic" [Mesh] OR (Scleroderma, Systemic) OR (Systemic Sclerosis) OR (Systemic Scleroderma) OR "Mixed Connective Tissue Disease" [Mesh] OR (Mixed Connective Tissue Disease) OR (Sharp Syndrome) OR (MCTD)

**I = cs-DMARDs**

(cs-DMARDs) OR (conventional synthetic disease modifying anti-rheumatic drugs) OR "Leflunomide" [Mesh] OR (Leflunomide) OR (N-(4-Trifluoromethyphenyl)-5-methylisoxazole-4-carboxamide) OR (HWA 486) OR (HWA486) OR (SU101) OR (Arava) OR "Sulfasalazine" [Mesh] OR (Sulfasalazine) OR (Salicylazosulfapyridine) OR (Sulphasalazine) OR (Salazosulfapyridine) OR (Pyralin EN) OR (Azulfadine) OR (Azulfidine EN) OR (Azulfidine) OR (Asulfidine) OR (Colo Pleon) OR (Pleon) OR (Ulcol) OR (Sulfasalazin medac) OR (Sulfasalazin Heyl) OR (Sulfasalazine FNA) OR (Ucine) OR (Salazopyrin ratio Sulfasalazine) OR "Chloroquine" [Mesh] OR (Chloroquine) OR (Chlorochin) OR (Chingamin) OR (Khingamin) OR (Nivaquine) OR (Chloroquine Sulfate) OR (Chloroquine Sulphate) OR (Aralen) OR (Arequin) OR (Arechine) OR "Hydroxychloroquine" [Mesh] OR (Hydroxychloroquine) OR (Oxychlorochin) OR (Oxychloroquine) OR (Hydroxychlorochin) OR (Plaquenil) OR (Hydroxychloroquine Sulfate) OR "Methotrexate" [Mesh] OR (Methotrexate) OR (Amethopterin Mexate) OR (Methotrexate Sodium) OR (Methotrexate Hydrate) OR (Dicesium Salt Methotrexate)

**O = Vaccination with inactivated vaccine**

"Vaccines, Inactivated" [Mesh] OR (Vaccines, Inactivated) OR (Inactivated Vaccines) OR (Killed Vaccine) OR (Killed Vaccines) OR (Inactivated Vaccine) OR (Influenza Vaccines) OR (Influenza Vaccine) OR (Influenzavirus Vaccines) OR (Influenza Virus Vaccines) OR (Influenzavirus Vaccine) OR (Influenza Virus Vaccine) OR (High-Dose Trivalent Influenza Vaccine) OR (High Dose Trivalent Influenza Vaccine) OR (Trivalent Influenza Vaccine) OR (Quadrivalent Influenza Vaccine) OR "Pneumococcal Vaccines" [Mesh] OR (Pneumococcal Vaccines) OR (Pneumococcal Vaccine) OR (Pneumovax) OR (Pneumococcal Polysaccharide Vaccine) OR (Pnu Imune Vaccine) OR (PnuImune Vaccine) OR (Conjugated penumococcal vaccine) OR (Herpes Zoster Vaccine) OR (Zoster Vaccine) OR (Shingles Vaccine) OR "Hepatitis B Vaccines" [Mesh] OR (Hepatitis B Vaccines) OR (Hepatitis B Vaccine) OR "Papillomavirus Vaccines" [Mesh] OR (Papillomavirus Vaccines) OR (Papillomavirus Vaccine) OR (Human Papillomavirus Vaccines) OR (HPV Vaccine) OR (Human Papilloma Virus Vaccine) OR (HPV Vaccines) OR (Human Papilloma Virus Vaccines) OR (Human Papillomavirus Vaccine) OR "Poliovirus Vaccines" [Mesh] OR (Poliovirus Vaccines) OR (Poliovirus Vaccine) OR (salk) OR "Hepatitis A Vaccines" [Mesh] OR (Hepatitis A Vaccines) OR (Hepatovirus Vaccines) OR (Hepatitis A Vaccine) OR (Vaqta) OR (Havrix) OR "Meningococcal Vaccines" [Mesh] OR (Meningococcal Vaccine) OR (Menactra) OR (Tetravalent Meningococcal Vaccine) OR (Menomune) OR (Menveo) OR (Bivalent Meningococcal Vaccine) OR (Meningococcal Polysaccharide Vaccine) OR "Diphtheria-Tetanus-Pertussis Vaccine" [Mesh] OR (Diphtheria-Tetanus-Pertussis Vaccine) OR (Diphtheria Tetanus Pertussis Vaccine) OR (DTwP Vaccine) OR (DPT Vaccine) OR (DTP Vaccine) OR (Di Te Per Vaccine) OR (Diphtheria Pertussis Tetanus Vaccine) OR (Haemophilus influenza type b polysaccharide vaccine-tetanus toxin conjugate) OR (PRP-T) OR (Hib-TT) OR (OmniHIB) OR (ActHIB)

#1 AND #2 AND #3

("rheumatic diseases"[MeSH Terms] OR ("rheumatic"[All Fields] AND "diseases"[All Fields]) OR "rheumatic diseases"[All Fields] OR ("rheumatic diseases"[MeSH Terms] OR ("rheumatic"[All Fields] AND "diseases"[All Fields]) OR "rheumatic diseases"[All Fields] OR ("rheumatic"[All Fields] AND "disease"[All Fields]) OR "rheumatic disease"[All Fields]) OR ("rheumatic diseases"[MeSH Terms] OR ("rheumatic"[All Fields] AND "diseases"[All Fields]) OR "rheumatic diseases"[All Fields] OR "rheumatics"[All Fields] OR "rheumatism"[All Fields] OR "rheumatic"[All Fields] OR "rheumatisms"[All Fields]) OR ("Immune-mediated"[All Fields] AND ("rheumatic diseases"[MeSH Terms] OR ("rheumatic"[All Fields] AND "diseases"[All Fields]) OR "rheumatic diseases"[All Fields])) OR ("arthritis, rheumatoid"[MeSH Terms] OR ("arthritis"[All Fields] AND "rheumatoid"[All Fields]) OR "rheumatoid arthritis"[All Fields] OR ("arthritis"[All Fields] AND "rheumatoid"[All Fields]) OR "arthritis rheumatoid"[All Fields]) OR ("arthritis, rheumatoid"[MeSH Terms] OR ("arthritis"[All Fields] AND "rheumatoid"[All Fields]) OR "rheumatoid arthritis"[All Fields] OR ("rheumatoid"[All Fields] AND "arthritis"[All Fields])) OR ("rheumatoid nodule"[MeSH Terms] OR ("rheumatoid"[All Fields] AND "nodule"[All Fields]) OR "rheumatoid nodule"[All Fields]) OR ("rheumatoid vasculitis"[MeSH Terms] OR ("rheumatoid"[All Fields] AND "vasculitis"[All Fields]) OR "rheumatoid vasculitis"[All Fields]) OR ("Sjogren's Syndrome"[MeSH Terms] OR ("sjogren s"[All Fields] AND "syndrome"[All Fields]) OR "Sjogren's Syndrome"[All Fields]) OR "Spondylarthritis"[MeSH Terms] OR ("Spondylarthritis"[MeSH Terms] OR "Spondylarthritis"[All Fields] OR "spondylarthritides"[All Fields]) OR ("Spondylarthritis"[MeSH Terms] OR "Spondylarthritis"[All Fields] OR "spondylarthritides"[All Fields]) OR ("Spondylarthritis"[MeSH Terms] OR "Spondylarthritis"[All Fields] OR ("spinal"[All Fields] AND "arthritis"[All Fields]) OR "spinal arthritis"[All Fields]) OR "arthritis, psoriatic"[MeSH Terms] OR ("arthritis, psoriatic"[MeSH Terms] OR ("arthritis"[All Fields] AND "psoriatic"[All Fields]) OR "psoriatic arthritis"[All Fields] OR ("arthritis"[All Fields] AND "psoriatic"[All Fields]) OR "arthritis psoriatic"[All Fields]) OR ("arthritis, psoriatic"[MeSH Terms] OR ("arthritis"[All Fields] AND "psoriatic"[All Fields]) OR "psoriatic arthritis"[All Fields] OR ("arthritic"[All Fields] AND "psoriasis"[All Fields]) OR "arthritic psoriasis"[All Fields]) OR ("arthritis, psoriatic"[MeSH Terms] OR ("arthritis"[All Fields] AND "psoriatic"[All Fields]) OR "psoriatic arthritis"[All Fields] OR ("psoriatic"[All Fields] AND "arthritis"[All Fields])) OR ("arthritis, psoriatic"[MeSH Terms] OR ("arthritis"[All Fields] AND "psoriatic"[All Fields]) OR "psoriatic arthritis"[All Fields] OR ("psoriasis"[All Fields] AND "arthropathica"[All Fields]) OR "psoriasis arthropathica"[All Fields]) OR ("arthritis, psoriatic"[MeSH Terms] OR ("arthritis"[All Fields] AND "psoriatic"[All Fields]) OR "psoriatic arthritis"[All Fields] OR ("psoriatic"[All Fields] AND "arthropathy"[All Fields]) OR "psoriatic arthropathy"[All Fields]) OR ("arthritis, psoriatic"[MeSH Terms] OR ("arthritis"[All Fields] AND "psoriatic"[All Fields]) OR "psoriatic arthritis"[All Fields] OR ("psoriatic"[All Fields] AND "arthropathies"[All Fields]) OR "psoriatic arthropathies"[All Fields]) OR "spondylitis, ankylosing"[MeSH Terms] OR ("spondylitis, ankylosing"[MeSH Terms] OR ("spondylitis"[All Fields] AND "ankylosing"[All Fields]) OR "ankylosing spondylitis"[All Fields] OR ("spondylitis"[All Fields] AND "ankylosing"[All Fields]) OR "spondylitis ankylosing"[All Fields]) OR ("spondylitis, ankylosing"[MeSH Terms] OR ("spondylitis"[All Fields] AND "ankylosing"[All Fields]) OR "ankylosing spondylitis"[All Fields] OR ("bechterew s"[All Fields] AND "disease"[All Fields]) OR "bechterew s disease"[All Fields]) OR ("spondylitis, ankylosing"[MeSH Terms] OR ("spondylitis"[All Fields] AND "ankylosing"[All Fields]) OR "ankylosing spondylitis"[All Fields] OR ("bechterews"[All Fields] AND "disease"[All Fields]) OR "bechterews disease"[All Fields]) OR ("spondylitis, ankylosing"[MeSH Terms] OR ("spondylitis"[All Fields] AND "ankylosing"[All Fields]) OR "ankylosing spondylitis"[All Fields] OR ("marie"[All Fields] AND "struempell"[All Fields] AND "disease"[All Fields]) OR "marie struempell disease"[All Fields]) OR ("spondylitis, ankylosing"[MeSH Terms] OR ("spondylitis"[All Fields] AND "ankylosing"[All Fields]) OR "ankylosing spondylitis"[All Fields] OR ("Spondylarthritis"[All Fields] AND "ankylopoietica"[All Fields]) OR "spondylarthritis ankylopoietica"[All Fields]) OR ("spondylitis, ankylosing"[MeSH Terms] OR ("spondylitis"[All Fields] AND "ankylosing"[All Fields]) OR "ankylosing spondylitis"[All Fields] OR ("spondyloarthritis"[All Fields] AND "ankylopoietica"[All Fields]) OR "spondyloarthritis ankylopoietica"[All Fields]) OR ("spondylitis, ankylosing"[MeSH Terms] OR ("spondylitis"[All Fields] AND "ankylosing"[All Fields]) OR "ankylosing spondylitis"[All Fields] OR ("ankylosing"[All Fields] AND "spondylitis"[All Fields])) OR ("spondylitis, ankylosing"[MeSH Terms] OR ("spondylitis"[All Fields] AND "ankylosing"[All Fields]) OR "ankylosing spondylitis"[All Fields] OR ("ankylosing"[All Fields] AND "Spondylarthritis"[All Fields]) OR "ankylosing spondylarthritis"[All Fields]) OR ("spondylitis, ankylosing"[MeSH Terms] OR ("spondylitis"[All Fields] AND "ankylosing"[All Fields]) OR "ankylosing spondylitis"[All Fields] OR ("ankylosing"[All Fields] AND "spondylarthritides"[All Fields]) OR "ankylosing spondylarthritides"[All Fields]) OR ("spondylitis, ankylosing"[MeSH Terms] OR ("spondylitis"[All Fields] AND "ankylosing"[All Fields]) OR "ankylosing spondylitis"[All Fields] OR ("ankylosing"[All Fields] AND "spondyloarthritis"[All Fields]) OR "ankylosing spondyloarthritis"[All Fields]) OR ("spondylitis, ankylosing"[MeSH Terms] OR ("spondylitis"[All Fields] AND "ankylosing"[All Fields]) OR "ankylosing spondylitis"[All Fields] OR ("spondylitis"[All Fields] AND "ankylopoietica"[All Fields]) OR "spondylitis ankylopoietica"[All Fields]) OR ("spondylitis, ankylosing"[MeSH Terms] OR ("spondylitis"[All Fields] AND "ankylosing"[All Fields]) OR "ankylosing spondylitis"[All Fields] OR ("bechterew"[All Fields] AND "disease"[All Fields]) OR "bechterew disease"[All Fields]) OR ("spondylitis, ankylosing"[MeSH Terms] OR ("spondylitis"[All Fields] AND "ankylosing"[All Fields]) OR "ankylosing spondylitis"[All Fields] OR ("rheumatoid"[All Fields] AND "spondylitis"[All Fields]) OR "rheumatoid spondylitis"[All Fields]) OR "lupus erythematosus, systemic"[MeSH Terms] OR ("lupus erythematosus, systemic"[MeSH Terms] OR ("lupus"[All Fields] AND "erythematosus"[All Fields] AND "systemic"[All Fields]) OR "systemic lupus erythematosus"[All Fields] OR ("lupus"[All Fields] AND "erythematosus"[All Fields] AND "systemic"[All Fields]) OR "lupus erythematosus systemic"[All Fields]) OR ("lupus erythematosus, systemic"[MeSH Terms] OR ("lupus"[All Fields] AND "erythematosus"[All Fields] AND "systemic"[All Fields]) OR "systemic lupus erythematosus"[All Fields] OR ("systemic"[All Fields] AND "lupus"[All Fields] AND "erythematosus"[All Fields])) OR ("lupus erythematosus, systemic"[MeSH Terms] OR ("lupus"[All Fields] AND "erythematosus"[All Fields] AND "systemic"[All Fields]) OR "systemic lupus erythematosus"[All Fields] OR ("lupus"[All Fields] AND "erythematosus"[All Fields] AND "disseminatus"[All Fields]) OR "lupus erythematosus disseminatus"[All Fields]) OR ("lupus erythematosus, systemic"[MeSH Terms] OR ("lupus"[All Fields] AND "erythematosus"[All Fields] AND "systemic"[All Fields]) OR "systemic lupus erythematosus"[All Fields] OR ("libman"[All Fields] AND "sacks"[All Fields] AND "disease"[All Fields]) OR "libman sacks disease"[All Fields]) OR "Myositis"[MeSH Terms] OR ("Myositis"[MeSH Terms] OR "Myositis"[All Fields] OR "myositides"[All Fields]) OR ("Myositis"[MeSH Terms] OR "Myositis"[All Fields] OR "myositides"[All Fields]) OR ("Myositis"[MeSH Terms] OR "Myositis"[All Fields] OR ("inflammatory"[All Fields] AND "muscle"[All Fields] AND "diseases"[All Fields]) OR "inflammatory muscle diseases"[All Fields]) OR ("Myositis"[MeSH Terms] OR "Myositis"[All Fields] OR ("inflammatory"[All Fields] AND "muscle"[All Fields] AND "disease"[All Fields]) OR "inflammatory muscle disease"[All Fields]) OR ("Myositis"[MeSH Terms] OR "Myositis"[All Fields] OR ("inflammatory"[All Fields] AND "myopathy"[All Fields]) OR "inflammatory myopathy"[All Fields]) OR ("Myositis"[MeSH Terms] OR "Myositis"[All Fields] OR ("inflammatory"[All Fields] AND "myopathies"[All Fields]) OR "inflammatory myopathies"[All Fields]) OR ("Myositis"[MeSH Terms] OR "Myositis"[All Fields] OR ("proliferative"[All Fields] AND "Myositis"[All Fields]) OR "proliferative myositis"[All Fields]) OR ("Myositis"[MeSH Terms] OR "Myositis"[All Fields] OR ("infectious"[All Fields] AND "myositides"[All Fields]) OR "infectious myositides"[All Fields]) OR ("Myositis"[MeSH Terms] OR "Myositis"[All Fields] OR ("infectious"[All Fields] AND "Myositis"[All Fields]) OR "infectious myositis"[All Fields]) OR ("Myositis"[MeSH Terms] OR "Myositis"[All Fields] OR ("idiopathic"[All Fields] AND "inflammatory"[All Fields] AND "myopathies"[All Fields]) OR "idiopathic inflammatory myopathies"[All Fields]) OR ("Myositis"[MeSH Terms] OR "Myositis"[All Fields] OR ("idiopathic"[All Fields] AND "inflammatory"[All Fields] AND "myopathy"[All Fields]) OR "idiopathic inflammatory myopathy"[All Fields]) OR ("Myositis"[MeSH Terms] OR "Myositis"[All Fields] OR ("idiopathic"[All Fields] AND "inflammatory"[All Fields] AND "Myositis"[All Fields]) OR "idiopathic inflammatory myositis"[All Fields]) OR ("Myositis"[MeSH Terms] OR "Myositis"[All Fields] OR ("focal"[All Fields] AND "Myositis"[All Fields]) OR "focal myositis"[All Fields]) OR "Systemic Vasculitis"[MeSH Terms] OR ("Systemic Vasculitis"[MeSH Terms] OR ("systemic"[All Fields] AND "vasculitis"[All Fields]) OR "Systemic Vasculitis"[All Fields]) OR ("Systemic Vasculitis"[MeSH Terms] OR ("systemic"[All Fields] AND "vasculitis"[All Fields]) OR "Systemic Vasculitis"[All Fields] OR ("systemic"[All Fields] AND "vasculitides"[All Fields]) OR "systemic vasculitides"[All Fields]) OR "Sjogren's Syndrome"[MeSH Terms] OR ("Sjogren's Syndrome"[MeSH Terms] OR ("sjogren s"[All Fields] AND "syndrome"[All Fields]) OR "Sjogren's Syndrome"[All Fields]) OR ("Sjogren's Syndrome"[MeSH Terms] OR ("sjogren s"[All Fields] AND "syndrome"[All Fields]) OR "Sjogren's Syndrome"[All Fields] OR ("sjogrens"[All Fields] AND "syndrome"[All Fields]) OR "sjogrens syndrome"[All Fields]) OR ("Sjogren's Syndrome"[MeSH Terms] OR ("sjogren s"[All Fields] AND "syndrome"[All Fields]) OR "Sjogren's Syndrome"[All Fields] OR ("sjogren"[All Fields] AND "syndrome"[All Fields]) OR "sjogren syndrome"[All Fields]) OR ("Sjogren's Syndrome"[MeSH Terms] OR ("sjogren s"[All Fields] AND "syndrome"[All Fields]) OR "Sjogren's Syndrome"[All Fields] OR ("sicca"[All Fields] AND "syndrome"[All Fields]) OR "sicca syndrome"[All Fields]) OR "scleroderma, systemic"[MeSH Terms] OR ("scleroderma, systemic"[MeSH Terms] OR ("scleroderma"[All Fields] AND "systemic"[All Fields]) OR "systemic scleroderma"[All Fields] OR ("scleroderma"[All Fields] AND "systemic"[All Fields]) OR "scleroderma systemic"[All Fields]) OR ("scleroderma, systemic"[MeSH Terms] OR ("scleroderma"[All Fields] AND "systemic"[All Fields]) OR "systemic scleroderma"[All Fields] OR ("systemic"[All Fields] AND "sclerosis"[All Fields]) OR "systemic sclerosis"[All Fields]) OR ("scleroderma, systemic"[MeSH Terms] OR ("scleroderma"[All Fields] AND "systemic"[All Fields]) OR "systemic scleroderma"[All Fields] OR ("systemic"[All Fields] AND "scleroderma"[All Fields])) OR "Mixed Connective Tissue Disease"[MeSH Terms] OR ("Mixed Connective Tissue Disease"[MeSH Terms] OR ("mixed"[All Fields] AND "connective"[All Fields] AND "tissue"[All Fields] AND "disease"[All Fields]) OR "Mixed Connective Tissue Disease"[All Fields]) OR ("Mixed Connective Tissue Disease"[MeSH Terms] OR ("mixed"[All Fields] AND "connective"[All Fields] AND "tissue"[All Fields] AND "disease"[All Fields]) OR "Mixed Connective Tissue Disease"[All Fields] OR ("sharp"[All Fields] AND "syndrome"[All Fields]) OR "sharp syndrome"[All Fields]) OR ("Mixed Connective Tissue Disease"[MeSH Terms] OR ("mixed"[All Fields] AND "connective"[All Fields] AND "tissue"[All Fields] AND "disease"[All Fields]) OR "Mixed Connective Tissue Disease"[All Fields] OR "mctd"[All Fields])) AND ("cs-DMARDs"[All Fields] OR (("conventional"[All Fields] OR "conventionals"[All Fields]) AND ("synthetic"[All Fields] OR "synthetically"[All Fields] OR "synthetics"[All Fields] OR "synthetize"[All Fields] OR "synthetized"[All Fields] OR "synthetizing"[All Fields]) AND ("disease"[MeSH Terms] OR "disease"[All Fields] OR "diseases"[All Fields] OR "disease s"[All Fields] OR "diseased"[All Fields]) AND ("modifiable"[All Fields] OR "modified"[All Fields] OR "modifier"[All Fields] OR "modifiers"[All Fields] OR "modifies"[All Fields] OR "modify"[All Fields] OR "modifying"[All Fields]) AND ("antirheumatic agents"[Pharmacological Action] OR "antirheumatic agents"[MeSH Terms] OR ("antirheumatic"[All Fields] AND "agents"[All Fields]) OR "antirheumatic agents"[All Fields] OR ("anti"[All Fields] AND "rheumatic"[All Fields] AND "drugs"[All Fields]) OR "anti rheumatic drugs"[All Fields])) OR "Leflunomide"[MeSH Terms] OR ("leflunomid"[All Fields] OR "Leflunomide"[MeSH Terms] OR "Leflunomide"[All Fields] OR "leflunomide s"[All Fields]) OR (("n"[All Fields] AND "4-Trifluoromethyphenyl"[All Fields]) AND "5 methylisoxazole 4 carboxamide"[All Fields]) OR ("Leflunomide"[MeSH Terms] OR "Leflunomide"[All Fields] OR "hwa 486"[All Fields]) OR ("Leflunomide"[MeSH Terms] OR "Leflunomide"[All Fields] OR "hwa486"[All Fields]) OR ("Leflunomide"[MeSH Terms] OR "Leflunomide"[All Fields] OR "su101"[All Fields]) OR ("leflunomid"[All Fields] OR "Leflunomide"[MeSH Terms] OR "Leflunomide"[All Fields] OR "arava"[All Fields] OR "leflunomide s"[All Fields]) OR "Sulfasalazine"[MeSH Terms] OR ("Sulfasalazine"[MeSH Terms] OR "Sulfasalazine"[All Fields] OR "sulfasalazin"[All Fields] OR "sulphasalazine"[All Fields]) OR ("Sulfasalazine"[MeSH Terms] OR "Sulfasalazine"[All Fields] OR "salicylazosulfapyridine"[All Fields]) OR ("Sulfasalazine"[MeSH Terms] OR "Sulfasalazine"[All Fields] OR "sulfasalazin"[All Fields] OR "sulphasalazine"[All Fields]) OR ("Sulfasalazine"[MeSH Terms] OR "Sulfasalazine"[All Fields] OR "salazosulfapyridine"[All Fields]) OR ("Sulfasalazine"[MeSH Terms] OR "Sulfasalazine"[All Fields] OR ("pyralin"[All Fields] AND "en"[All Fields])) OR ("Sulfasalazine"[MeSH Terms] OR "Sulfasalazine"[All Fields]) OR ("Sulfasalazine"[MeSH Terms] OR "Sulfasalazine"[All Fields] OR ("azulfidine"[All Fields] AND "en"[All Fields]) OR "azulfidine en"[All Fields]) OR ("Sulfasalazine"[MeSH Terms] OR "Sulfasalazine"[All Fields] OR "azulfidine"[All Fields] OR "sulfasalazin"[All Fields] OR "sulphasalazine"[All Fields]) OR ("Sulfasalazine"[MeSH Terms] OR "Sulfasalazine"[All Fields] OR "asulfidine"[All Fields]) OR ("Sulfasalazine"[MeSH Terms] OR "Sulfasalazine"[All Fields] OR ("colo"[All Fields] AND "pleon"[All Fields]) OR "colo pleon"[All Fields]) OR ("Sulfasalazine"[MeSH Terms] OR "Sulfasalazine"[All Fields] OR "pleon"[All Fields]) OR ("Sulfasalazine"[MeSH Terms] OR "Sulfasalazine"[All Fields]) OR ("Sulfasalazine"[MeSH Terms] OR "Sulfasalazine"[All Fields] OR ("sulfasalazin"[All Fields] AND "medac"[All Fields])) OR ("Sulfasalazine"[MeSH Terms] OR "Sulfasalazine"[All Fields] OR ("sulfasalazin"[All Fields] AND "heyl"[All Fields])) OR ("Sulfasalazine"[MeSH Terms] OR "Sulfasalazine"[All Fields] OR ("Sulfasalazine"[All Fields] AND "fna"[All Fields])) OR ("Sulfasalazine"[MeSH Terms] OR "Sulfasalazine"[All Fields] OR "ucine"[All Fields]) OR (("salazopyrine"[Supplementary Concept] OR "salazopyrine"[All Fields] OR "salazopyrine"[All Fields] OR "Sulfasalazine"[MeSH Terms] OR "Sulfasalazine"[All Fields] OR "salazopyrin"[All Fields]) AND ("Sulfasalazine"[MeSH Terms] OR "Sulfasalazine"[All Fields] OR ("ratio"[All Fields] AND "Sulfasalazine"[All Fields]))) OR "Chloroquine"[MeSH Terms] OR ("chloroquin"[All Fields] OR "Chloroquine"[MeSH Terms] OR "Chloroquine"[All Fields] OR "chloroquine s"[All Fields] OR "chloroquines"[All Fields]) OR ("Chloroquine"[MeSH Terms] OR "Chloroquine"[All Fields] OR "chlorochin"[All Fields]) OR ("Chloroquine"[MeSH Terms] OR "Chloroquine"[All Fields] OR "chingamin"[All Fields]) OR ("Chloroquine"[MeSH Terms] OR "Chloroquine"[All Fields] OR "khingamin"[All Fields]) OR ("Chloroquine"[MeSH Terms] OR "Chloroquine"[All Fields] OR "nivaquine"[All Fields]) OR ("chloroquine sulphate"[All Fields] OR "Chloroquine"[MeSH Terms] OR "Chloroquine"[All Fields] OR ("Chloroquine"[All Fields] AND "sulfate"[All Fields]) OR "chloroquine sulfate"[All Fields]) OR ("chloroquine sulphate"[All Fields] OR "Chloroquine"[MeSH Terms] OR "Chloroquine"[All Fields] OR ("Chloroquine"[All Fields] AND "sulfate"[All Fields]) OR "chloroquine sulfate"[All Fields]) OR ("Chloroquine"[MeSH Terms] OR "Chloroquine"[All Fields] OR "aralen"[All Fields]) OR ("Chloroquine"[MeSH Terms] OR "Chloroquine"[All Fields]) OR ("Chloroquine"[MeSH Terms] OR "Chloroquine"[All Fields] OR "arechine"[All Fields]) OR "Hydroxychloroquine"[MeSH Terms] OR ("Hydroxychloroquine"[MeSH Terms] OR "Hydroxychloroquine"[All Fields]) OR ("Hydroxychloroquine"[MeSH Terms] OR "Hydroxychloroquine"[All Fields]) OR ("Hydroxychloroquine"[MeSH Terms] OR "Hydroxychloroquine"[All Fields] OR "oxychloroquine"[All Fields]) OR ("Hydroxychloroquine"[MeSH Terms] OR "Hydroxychloroquine"[All Fields]) OR ("Hydroxychloroquine"[MeSH Terms] OR "Hydroxychloroquine"[All Fields] OR "plaquenil"[All Fields]) OR ("hydroxychloroquine sulphate"[All Fields] OR "Hydroxychloroquine"[MeSH Terms] OR "Hydroxychloroquine"[All Fields] OR ("Hydroxychloroquine"[All Fields] AND "sulfate"[All Fields]) OR "hydroxychloroquine sulfate"[All Fields]) OR "Methotrexate"[MeSH Terms] OR ("Methotrexate"[MeSH Terms] OR "Methotrexate"[All Fields] OR "methotrexate s"[All Fields] OR "methotrexates"[All Fields]) OR (("Methotrexate"[MeSH Terms] OR "Methotrexate"[All Fields] OR "amethopterin"[All Fields]) AND ("Methotrexate"[MeSH Terms] OR "Methotrexate"[All Fields] OR "mexate"[All Fields])) OR ("Methotrexate"[MeSH Terms] OR "Methotrexate"[All Fields] OR ("Methotrexate"[All Fields] AND "sodium"[All Fields]) OR "methotrexate sodium"[All Fields]) OR ("Methotrexate"[MeSH Terms] OR "Methotrexate"[All Fields] OR ("Methotrexate"[All Fields] AND "hydrate"[All Fields]) OR "methotrexate hydrate"[All Fields]) OR ("Methotrexate"[MeSH Terms] OR "Methotrexate"[All Fields] OR ("dicesium"[All Fields] AND "salt"[All Fields] AND "Methotrexate"[All Fields]))) AND ("vaccines, inactivated"[MeSH Terms] OR ("vaccines, inactivated"[MeSH Terms] OR ("vaccines"[All Fields] AND "inactivated"[All Fields]) OR "inactivated vaccines"[All Fields] OR ("vaccines"[All Fields] AND "inactivated"[All Fields]) OR "vaccines inactivated"[All Fields]) OR ("vaccines, inactivated"[MeSH Terms] OR ("vaccines"[All Fields] AND "inactivated"[All Fields]) OR "inactivated vaccines"[All Fields] OR ("inactivated"[All Fields] AND "vaccines"[All Fields])) OR ("vaccines, inactivated"[MeSH Terms] OR ("vaccines"[All Fields] AND "inactivated"[All Fields]) OR "inactivated vaccines"[All Fields] OR ("killed"[All Fields] AND "vaccine"[All Fields]) OR "killed vaccine"[All Fields]) OR ("vaccines, inactivated"[MeSH Terms] OR ("vaccines"[All Fields] AND "inactivated"[All Fields]) OR "inactivated vaccines"[All Fields] OR ("killed"[All Fields] AND "vaccines"[All Fields]) OR "killed vaccines"[All Fields]) OR ("vaccines, inactivated"[MeSH Terms] OR ("vaccines"[All Fields] AND "inactivated"[All Fields]) OR "inactivated vaccines"[All Fields] OR ("inactivated"[All Fields] AND "vaccine"[All Fields]) OR "inactivated vaccine"[All Fields]) OR ("influenza vaccines"[MeSH Terms] OR ("influenza"[All Fields] AND "vaccines"[All Fields]) OR "influenza vaccines"[All Fields]) OR ("influenza vaccines"[MeSH Terms] OR ("influenza"[All Fields] AND "vaccines"[All Fields]) OR "influenza vaccines"[All Fields] OR ("influenza"[All Fields] AND "vaccine"[All Fields]) OR "influenza vaccine"[All Fields]) OR ("influenza vaccines"[MeSH Terms] OR ("influenza"[All Fields] AND "vaccines"[All Fields]) OR "influenza vaccines"[All Fields] OR ("influenzavirus"[All Fields] AND "vaccines"[All Fields])) OR ("influenza vaccines"[MeSH Terms] OR ("influenza"[All Fields] AND "vaccines"[All Fields]) OR "influenza vaccines"[All Fields] OR ("influenza"[All Fields] AND "virus"[All Fields] AND "vaccines"[All Fields]) OR "influenza virus vaccines"[All Fields]) OR ("influenza vaccines"[MeSH Terms] OR ("influenza"[All Fields] AND "vaccines"[All Fields]) OR "influenza vaccines"[All Fields] OR ("influenzavirus"[All Fields] AND "vaccine"[All Fields]) OR "influenzavirus vaccine"[All Fields]) OR ("influenza vaccines"[MeSH Terms] OR ("influenza"[All Fields] AND "vaccines"[All Fields]) OR "influenza vaccines"[All Fields] OR ("influenza"[All Fields] AND "virus"[All Fields] AND "vaccine"[All Fields]) OR "influenza virus vaccine"[All Fields]) OR ("influenza vaccines"[MeSH Terms] OR ("influenza"[All Fields] AND "vaccines"[All Fields]) OR "influenza vaccines"[All Fields] OR ("high"[All Fields] AND "dose"[All Fields] AND "trivalent"[All Fields] AND "influenza"[All Fields] AND "vaccine"[All Fields]) OR "high dose trivalent influenza vaccine"[All Fields]) OR ("influenza vaccines"[MeSH Terms] OR ("influenza"[All Fields] AND "vaccines"[All Fields]) OR "influenza vaccines"[All Fields] OR ("high"[All Fields] AND "dose"[All Fields] AND "trivalent"[All Fields] AND "influenza"[All Fields] AND "vaccine"[All Fields]) OR "high dose trivalent influenza vaccine"[All Fields]) OR ("influenza vaccines"[MeSH Terms] OR ("influenza"[All Fields] AND "vaccines"[All Fields]) OR "influenza vaccines"[All Fields] OR ("trivalent"[All Fields] AND "influenza"[All Fields] AND "vaccine"[All Fields]) OR "trivalent influenza vaccine"[All Fields]) OR ("influenza vaccines"[MeSH Terms] OR ("influenza"[All Fields] AND "vaccines"[All Fields]) OR "influenza vaccines"[All Fields] OR ("quadrivalent"[All Fields] AND "influenza"[All Fields] AND "vaccine"[All Fields]) OR "quadrivalent influenza vaccine"[All Fields]) OR "Pneumococcal Vaccines"[MeSH Terms] OR ("Pneumococcal Vaccines"[MeSH Terms] OR ("pneumococcal"[All Fields] AND "vaccines"[All Fields]) OR "Pneumococcal Vaccines"[All Fields]) OR ("Pneumococcal Vaccines"[MeSH Terms] OR ("pneumococcal"[All Fields] AND "vaccines"[All Fields]) OR "Pneumococcal Vaccines"[All Fields] OR ("pneumococcal"[All Fields] AND "vaccine"[All Fields]) OR "pneumococcal vaccine"[All Fields]) OR ("Pneumococcal Vaccines"[MeSH Terms] OR ("pneumococcal"[All Fields] AND "vaccines"[All Fields]) OR "Pneumococcal Vaccines"[All Fields] OR "pneumovax"[All Fields]) OR ("Pneumococcal Vaccines"[MeSH Terms] OR ("pneumococcal"[All Fields] AND "vaccines"[All Fields]) OR "Pneumococcal Vaccines"[All Fields] OR ("pneumococcal"[All Fields] AND "polysaccharide"[All Fields] AND "vaccine"[All Fields]) OR "pneumococcal polysaccharide vaccine"[All Fields]) OR ("Pneumococcal Vaccines"[MeSH Terms] OR ("pneumococcal"[All Fields] AND "vaccines"[All Fields]) OR "Pneumococcal Vaccines"[All Fields] OR ("pnu"[All Fields] AND "imune"[All Fields] AND "vaccine"[All Fields]) OR "pnu imune vaccine"[All Fields]) OR ("Pneumococcal Vaccines"[MeSH Terms] OR ("pneumococcal"[All Fields] AND "vaccines"[All Fields]) OR "Pneumococcal Vaccines"[All Fields] OR ("pnuimune"[All Fields] AND "vaccine"[All Fields])) OR (("conjugable"[All Fields] OR "conjugal"[All Fields] OR "conjugally"[All Fields] OR "conjugant"[All Fields] OR "conjugants"[All Fields] OR "conjugate"[All Fields] OR "conjugate s"[All Fields] OR "conjugated"[All Fields] OR "conjugates"[All Fields] OR "conjugating"[All Fields] OR "conjugation"[All Fields] OR "conjugational"[All Fields] OR "conjugations"[All Fields] OR "conjugative"[All Fields] OR "conjugatively"[All Fields]) AND "penumococcal"[All Fields] AND ("vaccin"[Supplementary Concept] OR "vaccin"[All Fields] OR "vaccination"[MeSH Terms] OR "vaccination"[All Fields] OR "vaccinable"[All Fields] OR "vaccinal"[All Fields] OR "vaccinate"[All Fields] OR "vaccinated"[All Fields] OR "vaccinates"[All Fields] OR "vaccinating"[All Fields] OR "vaccinations"[All Fields] OR "vaccination s"[All Fields] OR "vaccinator"[All Fields] OR "vaccinators"[All Fields] OR "vaccine s"[All Fields] OR "vaccined"[All Fields] OR "vaccines"[MeSH Terms] OR "vaccines"[All Fields] OR "vaccine"[All Fields] OR "vaccins"[All Fields])) OR ("herpes zoster vaccine"[MeSH Terms] OR ("herpes"[All Fields] AND "zoster"[All Fields] AND "vaccine"[All Fields]) OR "herpes zoster vaccine"[All Fields]) OR ("herpes zoster vaccine"[MeSH Terms] OR ("herpes"[All Fields] AND "zoster"[All Fields] AND "vaccine"[All Fields]) OR "herpes zoster vaccine"[All Fields] OR ("zoster"[All Fields] AND "vaccine"[All Fields]) OR "zoster vaccine"[All Fields]) OR ("herpes zoster vaccine"[MeSH Terms] OR ("herpes"[All Fields] AND "zoster"[All Fields] AND "vaccine"[All Fields]) OR "herpes zoster vaccine"[All Fields] OR ("shingles"[All Fields] AND "vaccine"[All Fields]) OR "shingles vaccine"[All Fields]) OR "Hepatitis B Vaccines"[MeSH Terms] OR ("Hepatitis B Vaccines"[MeSH Terms] OR "Hepatitis B Vaccines"[All Fields]) OR ("Hepatitis B Vaccines"[MeSH Terms] OR "Hepatitis B Vaccines"[All Fields] OR "hepatitis b vaccine"[All Fields]) OR "Papillomavirus Vaccines"[MeSH Terms] OR ("Papillomavirus Vaccines"[MeSH Terms] OR ("papillomavirus"[All Fields] AND "vaccines"[All Fields]) OR "Papillomavirus Vaccines"[All Fields]) OR ("Papillomavirus Vaccines"[MeSH Terms] OR ("papillomavirus"[All Fields] AND "vaccines"[All Fields]) OR "Papillomavirus Vaccines"[All Fields] OR ("papillomavirus"[All Fields] AND "vaccine"[All Fields]) OR "papillomavirus vaccine"[All Fields]) OR ("Papillomavirus Vaccines"[MeSH Terms] OR ("papillomavirus"[All Fields] AND "vaccines"[All Fields]) OR "Papillomavirus Vaccines"[All Fields] OR ("human"[All Fields] AND "papillomavirus"[All Fields] AND "vaccines"[All Fields]) OR "human papillomavirus vaccines"[All Fields]) OR ("Papillomavirus Vaccines"[MeSH Terms] OR ("papillomavirus"[All Fields] AND "vaccines"[All Fields]) OR "Papillomavirus Vaccines"[All Fields] OR ("hpv"[All Fields] AND "vaccine"[All Fields]) OR "hpv vaccine"[All Fields]) OR ("Papillomavirus Vaccines"[MeSH Terms] OR ("papillomavirus"[All Fields] AND "vaccines"[All Fields]) OR "Papillomavirus Vaccines"[All Fields] OR ("human"[All Fields] AND "papilloma"[All Fields] AND "virus"[All Fields] AND "vaccine"[All Fields]) OR "human papilloma virus vaccine"[All Fields]) OR ("Papillomavirus Vaccines"[MeSH Terms] OR ("papillomavirus"[All Fields] AND "vaccines"[All Fields]) OR "Papillomavirus Vaccines"[All Fields] OR ("hpv"[All Fields] AND "vaccines"[All Fields]) OR "hpv vaccines"[All Fields]) OR ("Papillomavirus Vaccines"[MeSH Terms] OR ("papillomavirus"[All Fields] AND "vaccines"[All Fields]) OR "Papillomavirus Vaccines"[All Fields] OR ("human"[All Fields] AND "papilloma"[All Fields] AND "virus"[All Fields] AND "vaccines"[All Fields]) OR "human papilloma virus vaccines"[All Fields]) OR ("Papillomavirus Vaccines"[MeSH Terms] OR ("papillomavirus"[All Fields] AND "vaccines"[All Fields]) OR "Papillomavirus Vaccines"[All Fields] OR ("human"[All Fields] AND "papillomavirus"[All Fields] AND "vaccine"[All Fields]) OR "human papillomavirus vaccine"[All Fields]) OR "Poliovirus Vaccines"[MeSH Terms] OR ("Poliovirus Vaccines"[MeSH Terms] OR ("poliovirus"[All Fields] AND "vaccines"[All Fields]) OR "Poliovirus Vaccines"[All Fields]) OR ("Poliovirus Vaccines"[MeSH Terms] OR ("poliovirus"[All Fields] AND "vaccines"[All Fields]) OR "Poliovirus Vaccines"[All Fields] OR ("poliovirus"[All Fields] AND "vaccine"[All Fields]) OR "poliovirus vaccine"[All Fields]) OR "salk"[All Fields] OR "Hepatitis A Vaccines"[MeSH Terms] OR ("Hepatitis A Vaccines"[MeSH Terms] OR "Hepatitis A Vaccines"[All Fields]) OR ("Hepatitis A Vaccines"[MeSH Terms] OR "Hepatitis A Vaccines"[All Fields] OR ("hepatovirus"[All Fields] AND "vaccines"[All Fields])) OR ("Hepatitis A Vaccines"[MeSH Terms] OR "Hepatitis A Vaccines"[All Fields] OR "hepatitis a vaccine"[All Fields]) OR ("hepatitis a vaccine inactivated"[All Fields] OR "Hepatitis A Vaccines"[MeSH Terms] OR "Hepatitis A Vaccines"[All Fields] OR "vaqta"[All Fields]) OR ("Hepatitis A Vaccines"[MeSH Terms] OR "Hepatitis A Vaccines"[All Fields] OR "havrix"[All Fields]) OR "Meningococcal Vaccines"[MeSH Terms] OR ("Meningococcal Vaccines"[MeSH Terms] OR ("meningococcal"[All Fields] AND "vaccines"[All Fields]) OR "Meningococcal Vaccines"[All Fields] OR ("meningococcal"[All Fields] AND "vaccine"[All Fields]) OR "meningococcal vaccine"[All Fields]) OR ("Meningococcal Vaccines"[MeSH Terms] OR ("meningococcal"[All Fields] AND "vaccines"[All Fields]) OR "Meningococcal Vaccines"[All Fields] OR "menactra"[All Fields]) OR ("Meningococcal Vaccines"[MeSH Terms] OR ("meningococcal"[All Fields] AND "vaccines"[All Fields]) OR "Meningococcal Vaccines"[All Fields] OR ("tetravalent"[All Fields] AND "meningococcal"[All Fields] AND "vaccine"[All Fields]) OR "tetravalent meningococcal vaccine"[All Fields]) OR ("Meningococcal Vaccines"[MeSH Terms] OR ("meningococcal"[All Fields] AND "vaccines"[All Fields]) OR "Meningococcal Vaccines"[All Fields] OR "menomune"[All Fields]) OR ("Meningococcal Vaccines"[MeSH Terms] OR ("meningococcal"[All Fields] AND "vaccines"[All Fields]) OR "Meningococcal Vaccines"[All Fields] OR "menveo"[All Fields]) OR ("Meningococcal Vaccines"[MeSH Terms] OR ("meningococcal"[All Fields] AND "vaccines"[All Fields]) OR "Meningococcal Vaccines"[All Fields] OR ("bivalent"[All Fields] AND "meningococcal"[All Fields] AND "vaccine"[All Fields]) OR "bivalent meningococcal vaccine"[All Fields]) OR ("Meningococcal Vaccines"[MeSH Terms] OR ("meningococcal"[All Fields] AND "vaccines"[All Fields]) OR "Meningococcal Vaccines"[All Fields] OR ("meningococcal"[All Fields] AND "polysaccharide"[All Fields] AND "vaccine"[All Fields]) OR "meningococcal polysaccharide vaccine"[All Fields]) OR "Diphtheria-Tetanus-Pertussis Vaccine"[MeSH Terms] OR ("Diphtheria-Tetanus-Pertussis Vaccine"[MeSH Terms] OR ("diphtheria tetanus pertussis"[All Fields] AND "vaccine"[All Fields]) OR "Diphtheria-Tetanus-Pertussis Vaccine"[All Fields] OR ("diphtheria"[All Fields] AND "tetanus"[All Fields] AND "pertussis"[All Fields] AND "vaccine"[All Fields]) OR "Diphtheria-Tetanus-Pertussis Vaccine"[All Fields]) OR ("Diphtheria-Tetanus-Pertussis Vaccine"[MeSH Terms] OR ("diphtheria tetanus pertussis"[All Fields] AND "vaccine"[All Fields]) OR "Diphtheria-Tetanus-Pertussis Vaccine"[All Fields] OR ("diphtheria"[All Fields] AND "tetanus"[All Fields] AND "pertussis"[All Fields] AND "vaccine"[All Fields]) OR "Diphtheria-Tetanus-Pertussis Vaccine"[All Fields]) OR ("Diphtheria-Tetanus-Pertussis Vaccine"[MeSH Terms] OR ("diphtheria tetanus pertussis"[All Fields] AND "vaccine"[All Fields]) OR "Diphtheria-Tetanus-Pertussis Vaccine"[All Fields] OR ("dtwp"[All Fields] AND "vaccine"[All Fields]) OR "dtwp vaccine"[All Fields]) OR ("Diphtheria-Tetanus-Pertussis Vaccine"[MeSH Terms] OR ("diphtheria tetanus pertussis"[All Fields] AND "vaccine"[All Fields]) OR "Diphtheria-Tetanus-Pertussis Vaccine"[All Fields] OR ("dpt"[All Fields] AND "vaccine"[All Fields]) OR "dpt vaccine"[All Fields]) OR ("Diphtheria-Tetanus-Pertussis Vaccine"[MeSH Terms] OR ("diphtheria tetanus pertussis"[All Fields] AND "vaccine"[All Fields]) OR "Diphtheria-Tetanus-Pertussis Vaccine"[All Fields] OR ("dtp"[All Fields] AND "vaccine"[All Fields]) OR "dtp vaccine"[All Fields]) OR ("Diphtheria-Tetanus-Pertussis Vaccine"[MeSH Terms] OR ("diphtheria tetanus pertussis"[All Fields] AND "vaccine"[All Fields]) OR "Diphtheria-Tetanus-Pertussis Vaccine"[All Fields] OR ("di"[All Fields] AND "te"[All Fields] AND "per"[All Fields] AND "vaccine"[All Fields]) OR "di te per vaccine"[All Fields]) OR ("Diphtheria-Tetanus-Pertussis Vaccine"[MeSH Terms] OR ("diphtheria tetanus pertussis"[All Fields] AND "vaccine"[All Fields]) OR "Diphtheria-Tetanus-Pertussis Vaccine"[All Fields] OR ("diphtheria"[All Fields] AND "pertussis"[All Fields] AND "tetanus"[All Fields] AND "vaccine"[All Fields]) OR "diphtheria pertussis tetanus vaccine"[All Fields]) OR ("haemophilus influenza type b polysaccharide vaccine tetanus toxin conjugate"[Supplementary Concept] OR "haemophilus influenza type b polysaccharide vaccine tetanus toxin conjugate"[All Fields] OR "haemophilus influenza type b polysaccharide vaccine tetanus toxin conjugate"[All Fields]) OR ("haemophilus influenza type b polysaccharide vaccine tetanus toxin conjugate"[Supplementary Concept] OR "haemophilus influenza type b polysaccharide vaccine tetanus toxin conjugate"[All Fields] OR "prp t"[All Fields]) OR ("haemophilus influenza type b polysaccharide vaccine tetanus toxin conjugate"[Supplementary Concept] OR "haemophilus influenza type b polysaccharide vaccine tetanus toxin conjugate"[All Fields] OR "hib tt"[All Fields]) OR ("haemophilus influenza type b polysaccharide vaccine tetanus toxin conjugate"[Supplementary Concept] OR "haemophilus influenza type b polysaccharide vaccine tetanus toxin conjugate"[All Fields] OR "omnihib"[All Fields]) OR ("haemophilus influenza type b polysaccharide vaccine tetanus toxin conjugate"[Supplementary Concept] OR "haemophilus influenza type b polysaccharide vaccine tetanus toxin conjugate"[All Fields] OR "acthib"[All Fields]))

139 results

1.2 Following there are search strategy in Embase

**No.**

**Query**

**Results**

**1,027**

**#31**

**#12 AND #18 AND #30**

**149,994**

**#30**

**#19 OR #20 OR #21 OR #22 OR #23 OR #24 OR #25 OR #26 OR #27 OR #28 OR #29**

**36**

**#29**

**'haemophilus influenza type b polysaccharide vaccine tetanus toxin conjugate'/syn**

**22,584**

**#28**

**'diphtheria pertussis tetanus vaccine'/syn**

**8,774**

**#27**

**'meningococcal vaccines'/syn**

**5,982**

**#26**

**'hepatitis a vaccines'/syn**

**15,713**

**#25**

**'poliovirus vaccines'/syn**

**18,091**

**#24**

**'human papilloma virus vaccine'/syn**

**25,125**

**#23**

**'hepatitis b vaccine'/syn**

**4,441**

**#22**

**'herpes zoster vaccine'/syn**

**25,896**

**#21**

**'pneumococcus vaccine'/syn**

**48,734**

**#20**

**'influenza vaccines'/syn**

**10,379**

**#19**

**'inactivated vaccine'/syn**

**315,759**

**#18**

**#13 OR #14 OR #15 OR #16 OR #17**

**220,948**

**#17**

**'methotrexate'/syn**

**46,732**

**#16**

**'hydroxychloroquine'/syn**

**51,849**

**#15**

**'chloroquine'/syn**

**30,752**

**#14**

**'sulfasalazine'/syn**

**16,151**

**#13**

**'leflunomide'/syn**

**582,415**

**#12**

**#1 OR #2 OR #3 OR #4 OR #5 OR #6 OR #7 OR #8 OR #9 OR #10 OR #11**

**5,506**

**#11**

**'mixed connective tissue disease'/syn**

**43,158**

**#10**

**'systemic sclerosis'/syn**

**35,919**

**#9**

**'sjoegren syndrome'/syn**

**9,231**

**#8**

**'systemic vasculitis'/syn**

**52,684**

**#7**

**'myositis'/syn**

**141,094**

**#6**

**'systemic lupus erythematosus'/syn**

**40,970**

**#5**

**'ankylosing spondylitis'/syn**

**33,640**

**#4**

**'psoriatic arthritis'/syn**

**73,128**

**#3**

**'spondylarthritis'/syn**

**282,694**

**#2**

**'rheumatoid arthritis'/syn**

**56,077**

**#1**

**'rheumatic disease'/de**

1.3 Following there are search strategy in Cochrane Library

ID	Search	Hits

#1	MeSH descriptor: [Rheumatic Diseases] explode all trees	20731

#2	MeSH descriptor: [Arthritis, Rheumatoid] explode all trees	7302

#3	MeSH descriptor: [Spondylarthritis] explode all trees	1823

#4	MeSH descriptor: [Arthritis, Psoriatic] explode all trees	634

#5	MeSH descriptor: [Spondylitis, Ankylosing] explode all trees	826

#6	MeSH descriptor: [Lupus Erythematosus, Systemic] explode all trees	1425

#7	MeSH descriptor: [Myositis] explode all trees	318

#8	MeSH descriptor: [Systemic Vasculitis] explode all trees	266

#9	MeSH descriptor: [Sjogren's Syndrome] explode all trees	365

#10	MeSH descriptor: [Scleroderma, Systemic] explode all trees	717

#11	MeSH descriptor: [Mixed Connective Tissue Disease] explode all trees	13

#12	#1 OR #2 OR #3 OR #4 OR #5 OR #6 OR #7 OR #8 OR #9 OR #10 OR #11	24888

#13	MeSH descriptor: [Vaccines, Inactivated] explode all trees	1148

#14	MeSH descriptor: [Influenza Vaccines] explode all trees	1853

#15	MeSH descriptor: [Pneumococcal Vaccines] explode all trees	1178

#16	MeSH descriptor: [Herpes Zoster Vaccine] explode all trees	110

#17	MeSH descriptor: [Hepatitis B Vaccines] explode all trees	1003

#18	MeSH descriptor: [Papillomavirus Vaccines] explode all trees	549

#19	MeSH descriptor: [Poliovirus Vaccines] explode all trees	544

#20	MeSH descriptor: [Hepatitis A Vaccines] explode all trees	258

#21	MeSH descriptor: [Meningococcal Vaccines] explode all trees	438

#22	MeSH descriptor: [Diphtheria-Tetanus-Pertussis Vaccine] explode all trees	585

#23	#13 OR #14 OR #15 OR #16 OR #17 OR #18 OR #19 OR #20 OR #21 OR #22	5937

#24	#12 AND #23	62

#25	MeSH descriptor: [Leflunomide] explode all trees	194

#26	MeSH descriptor: [Sulfasalazine] explode all trees	527

#27	MeSH descriptor: [Chloroquine] explode all trees	1467

#28	MeSH descriptor: [Hydroxychloroquine] explode all trees	724

#29	MeSH descriptor: [Methotrexate] explode all trees	4768

#30	#25 OR #26 OR #27 OR #28 OR #29	6514

#31	#24 AND #30	23

**QUESTION 4. What is the impact of the immunosuppressants “azathioprine, mycophenolate mofetil, cyclosporine, cyclophosphamide’’ use on the response to inactivated vaccine in patients with immune-mediated rheumatic diseases?**

**1.1 Following there are search strategy in PubMed/MEDLINE**

**P = Patients with immune-mediated rheumatic diseases**

(Rheumatic Diseases) OR (Rheumatic Disease) OR (Rheumatism) OR (Immune-mediated rheumatic diseases) OR (Arthritis, Rheumatoid) OR (Rheumatoid Arthritis) OR (Rheumatoid Nodule) OR (Rheumatoid Vasculitis) OR (Sjogren's Syndrome) OR "Spondylarthritis" [Mesh] OR (Spondylarthritis) OR (Spondylarthritides) OR (Spinal Arthritis) OR "Arthritis, Psoriatic" [Mesh] OR (Arthritis, Psoriatic) OR (Arthritic Psoriasis) OR (Psoriatic Arthritis) OR (Psoriasis Arthropathica) OR (Psoriatic Arthropathy) OR (Psoriatic Arthropathies) OR "Spondylitis, Ankylosing" [Mesh] OR (Spondylitis, Ankylosing) OR (Bechterew's Disease) OR (Bechterews Disease) OR (Marie Struempell Disease) OR (Spondylarthritis Ankylopoietica) OR (Spondyloarthritis Ankylopoietica) OR (Ankylosing Spondylitis) OR (Ankylosing Spondylarthritis) OR (Ankylosing Spondylarthritides) OR (Ankylosing Spondyloarthritis) OR (Spondylitis Ankylopoietica) OR (Bechterew Disease) OR (Rheumatoid Spondylitis) OR "Lupus Erythematosus, Systemic" [Mesh] OR (Lupus Erythematosus, Systemic) OR (Systemic Lupus Erythematosus) OR (Lupus Erythematosus Disseminatus) OR (Libman Sacks Disease) OR "Myositis" [Mesh] OR (Myositis) OR (Myositides) OR (Inflammatory Muscle Diseases) OR (Inflammatory Muscle Disease) OR (Inflammatory Myopathy) OR (Inflammatory Myopathies) OR (Proliferative Myositis) OR (Infectious Myositides) OR (Infectious Myositis) OR (Idiopathic Inflammatory Myopathies) OR (Idiopathic Inflammatory Myopathy) OR (Idiopathic Inflammatory Myositis) OR (Focal Myositis) OR "Systemic Vasculitis" [Mesh] OR (Systemic Vasculitis) OR (Systemic Vasculitides) OR "Sjogren's Syndrome" [Mesh] OR (Sjogren's Syndrome) OR (Sjogrens Syndrome) OR (Sjogren Syndrome) OR (Sicca Syndrome) OR "Scleroderma, Systemic" [Mesh] OR (Scleroderma, Systemic) OR (Systemic Sclerosis) OR (Systemic Scleroderma) OR "Mixed Connective Tissue Disease" [Mesh] OR (Mixed Connective Tissue Disease) OR (Sharp Syndrome) OR (MCTD)

**I = Vaccination with inactivated vaccine**

"Vaccines, Inactivated" [Mesh] OR (Vaccines, Inactivated) OR (Inactivated Vaccines) OR (Killed Vaccine) OR (Killed Vaccines) OR (Inactivated Vaccine) OR (Influenza Vaccines) OR (Influenza Vaccine) OR (Influenzavirus Vaccines) OR (Influenza Virus Vaccines) OR (Influenzavirus Vaccine) OR (Influenza Virus Vaccine) OR (High-Dose Trivalent Influenza Vaccine) OR (High Dose Trivalent Influenza Vaccine) OR (Trivalent Influenza Vaccine) OR (Quadrivalent Influenza Vaccine) OR "Pneumococcal Vaccines" [Mesh] OR (Pneumococcal Vaccines) OR (Pneumococcal Vaccine) OR (Pneumovax) OR (Pneumococcal Polysaccharide Vaccine) OR (Pnu Imune Vaccine) OR (PnuImune Vaccine) OR (Conjugated penumococcal vaccine) OR (Herpes Zoster Vaccine) OR (Zoster Vaccine) OR (Shingles Vaccine) OR "Hepatitis B Vaccines" [Mesh] OR (Hepatitis B Vaccines) OR (Hepatitis B Vaccine) OR "Papillomavirus Vaccines" [Mesh] OR (Papillomavirus Vaccines) OR (Papillomavirus Vaccine) OR (Human Papillomavirus Vaccines) OR (HPV Vaccine) OR (Human Papilloma Virus Vaccine) OR (HPV Vaccines) OR (Human Papilloma Virus Vaccines) OR (Human Papillomavirus Vaccine) OR "Poliovirus Vaccines" [Mesh] OR (Poliovirus Vaccines) OR (Poliovirus Vaccine) OR (salk) OR "Hepatitis A Vaccines" [Mesh] OR (Hepatitis A Vaccines) OR (Hepatovirus Vaccines) OR (Hepatitis A Vaccine) OR (Vaqta) OR (Havrix) OR "Meningococcal Vaccines" [Mesh] OR (Meningococcal Vaccine) OR (Menactra) OR (Tetravalent Meningococcal Vaccine) OR (Menomune) OR (Menveo) OR (Bivalent Meningococcal Vaccine) OR (Meningococcal Polysaccharide Vaccine) OR "Diphtheria-Tetanus-Pertussis Vaccine" [Mesh] OR (Diphtheria-Tetanus-Pertussis Vaccine) OR (Diphtheria Tetanus Pertussis Vaccine) OR (DTwP Vaccine) OR (DPT Vaccine) OR (DTP Vaccine) OR (Di Te Per Vaccine) OR (Diphtheria Pertussis Tetanus Vaccine) OR (Haemophilus influenza type b polysaccharide vaccine-tetanus toxin conjugate) OR (PRP-T) OR (Hib-TT) OR (OmniHIB) OR (ActHIB)

#1 AND #2

("rheumatic diseases"[MeSH Terms] OR ("rheumatic"[All Fields] AND "diseases"[All Fields]) OR "rheumatic diseases"[All Fields] OR ("rheumatic diseases"[MeSH Terms] OR ("rheumatic"[All Fields] AND "diseases"[All Fields]) OR "rheumatic diseases"[All Fields] OR ("rheumatic"[All Fields] AND "disease"[All Fields]) OR "rheumatic disease"[All Fields]) OR ("rheumatic diseases"[MeSH Terms] OR ("rheumatic"[All Fields] AND "diseases"[All Fields]) OR "rheumatic diseases"[All Fields] OR "rheumatics"[All Fields] OR "rheumatism"[All Fields] OR "rheumatic"[All Fields] OR "rheumatisms"[All Fields]) OR ("Immune-mediated"[All Fields] AND ("rheumatic diseases"[MeSH Terms] OR ("rheumatic"[All Fields] AND "diseases"[All Fields]) OR "rheumatic diseases"[All Fields])) OR ("arthritis, rheumatoid"[MeSH Terms] OR ("arthritis"[All Fields] AND "rheumatoid"[All Fields]) OR "rheumatoid arthritis"[All Fields] OR ("arthritis"[All Fields] AND "rheumatoid"[All Fields]) OR "arthritis rheumatoid"[All Fields]) OR ("arthritis, rheumatoid"[MeSH Terms] OR ("arthritis"[All Fields] AND "rheumatoid"[All Fields]) OR "rheumatoid arthritis"[All Fields] OR ("rheumatoid"[All Fields] AND "arthritis"[All Fields])) OR ("rheumatoid nodule"[MeSH Terms] OR ("rheumatoid"[All Fields] AND "nodule"[All Fields]) OR "rheumatoid nodule"[All Fields]) OR ("rheumatoid vasculitis"[MeSH Terms] OR ("rheumatoid"[All Fields] AND "vasculitis"[All Fields]) OR "rheumatoid vasculitis"[All Fields]) OR ("Sjogren's Syndrome"[MeSH Terms] OR ("sjogren s"[All Fields] AND "syndrome"[All Fields]) OR "Sjogren's Syndrome"[All Fields]) OR "Spondylarthritis"[MeSH Terms] OR ("Spondylarthritis"[MeSH Terms] OR "Spondylarthritis"[All Fields] OR "spondylarthritides"[All Fields]) OR ("Spondylarthritis"[MeSH Terms] OR "Spondylarthritis"[All Fields] OR "spondylarthritides"[All Fields]) OR ("Spondylarthritis"[MeSH Terms] OR "Spondylarthritis"[All Fields] OR ("spinal"[All Fields] AND "arthritis"[All Fields]) OR "spinal arthritis"[All Fields]) OR "arthritis, psoriatic"[MeSH Terms] OR ("arthritis, psoriatic"[MeSH Terms] OR ("arthritis"[All Fields] AND "psoriatic"[All Fields]) OR "psoriatic arthritis"[All Fields] OR ("arthritis"[All Fields] AND "psoriatic"[All Fields]) OR "arthritis psoriatic"[All Fields]) OR ("arthritis, psoriatic"[MeSH Terms] OR ("arthritis"[All Fields] AND "psoriatic"[All Fields]) OR "psoriatic arthritis"[All Fields] OR ("arthritic"[All Fields] AND "psoriasis"[All Fields]) OR "arthritic psoriasis"[All Fields]) OR ("arthritis, psoriatic"[MeSH Terms] OR ("arthritis"[All Fields] AND "psoriatic"[All Fields]) OR "psoriatic arthritis"[All Fields] OR ("psoriatic"[All Fields] AND "arthritis"[All Fields])) OR ("arthritis, psoriatic"[MeSH Terms] OR ("arthritis"[All Fields] AND "psoriatic"[All Fields]) OR "psoriatic arthritis"[All Fields] OR ("psoriasis"[All Fields] AND "arthropathica"[All Fields]) OR "psoriasis arthropathica"[All Fields]) OR ("arthritis, psoriatic"[MeSH Terms] OR ("arthritis"[All Fields] AND "psoriatic"[All Fields]) OR "psoriatic arthritis"[All Fields] OR ("psoriatic"[All Fields] AND "arthropathy"[All Fields]) OR "psoriatic arthropathy"[All Fields]) OR ("arthritis, psoriatic"[MeSH Terms] OR ("arthritis"[All Fields] AND "psoriatic"[All Fields]) OR "psoriatic arthritis"[All Fields] OR ("psoriatic"[All Fields] AND "arthropathies"[All Fields]) OR "psoriatic arthropathies"[All Fields]) OR "spondylitis, ankylosing"[MeSH Terms] OR ("spondylitis, ankylosing"[MeSH Terms] OR ("spondylitis"[All Fields] AND "ankylosing"[All Fields]) OR "ankylosing spondylitis"[All Fields] OR ("spondylitis"[All Fields] AND "ankylosing"[All Fields]) OR "spondylitis ankylosing"[All Fields]) OR ("spondylitis, ankylosing"[MeSH Terms] OR ("spondylitis"[All Fields] AND "ankylosing"[All Fields]) OR "ankylosing spondylitis"[All Fields] OR ("bechterew s"[All Fields] AND "disease"[All Fields]) OR "bechterew s disease"[All Fields]) OR ("spondylitis, ankylosing"[MeSH Terms] OR ("spondylitis"[All Fields] AND "ankylosing"[All Fields]) OR "ankylosing spondylitis"[All Fields] OR ("bechterews"[All Fields] AND "disease"[All Fields]) OR "bechterews disease"[All Fields]) OR ("spondylitis, ankylosing"[MeSH Terms] OR ("spondylitis"[All Fields] AND "ankylosing"[All Fields]) OR "ankylosing spondylitis"[All Fields] OR ("marie"[All Fields] AND "struempell"[All Fields] AND "disease"[All Fields]) OR "marie struempell disease"[All Fields]) OR ("spondylitis, ankylosing"[MeSH Terms] OR ("spondylitis"[All Fields] AND "ankylosing"[All Fields]) OR "ankylosing spondylitis"[All Fields] OR ("Spondylarthritis"[All Fields] AND "ankylopoietica"[All Fields]) OR "spondylarthritis ankylopoietica"[All Fields]) OR ("spondylitis, ankylosing"[MeSH Terms] OR ("spondylitis"[All Fields] AND "ankylosing"[All Fields]) OR "ankylosing spondylitis"[All Fields] OR ("spondyloarthritis"[All Fields] AND "ankylopoietica"[All Fields]) OR "spondyloarthritis ankylopoietica"[All Fields]) OR ("spondylitis, ankylosing"[MeSH Terms] OR ("spondylitis"[All Fields] AND "ankylosing"[All Fields]) OR "ankylosing spondylitis"[All Fields] OR ("ankylosing"[All Fields] AND "spondylitis"[All Fields])) OR ("spondylitis, ankylosing"[MeSH Terms] OR ("spondylitis"[All Fields] AND "ankylosing"[All Fields]) OR "ankylosing spondylitis"[All Fields] OR ("ankylosing"[All Fields] AND "Spondylarthritis"[All Fields]) OR "ankylosing spondylarthritis"[All Fields]) OR ("spondylitis, ankylosing"[MeSH Terms] OR ("spondylitis"[All Fields] AND "ankylosing"[All Fields]) OR "ankylosing spondylitis"[All Fields] OR ("ankylosing"[All Fields] AND "spondylarthritides"[All Fields]) OR "ankylosing spondylarthritides"[All Fields]) OR ("spondylitis, ankylosing"[MeSH Terms] OR ("spondylitis"[All Fields] AND "ankylosing"[All Fields]) OR "ankylosing spondylitis"[All Fields] OR ("ankylosing"[All Fields] AND "spondyloarthritis"[All Fields]) OR "ankylosing spondyloarthritis"[All Fields]) OR ("spondylitis, ankylosing"[MeSH Terms] OR ("spondylitis"[All Fields] AND "ankylosing"[All Fields]) OR "ankylosing spondylitis"[All Fields] OR ("spondylitis"[All Fields] AND "ankylopoietica"[All Fields]) OR "spondylitis ankylopoietica"[All Fields]) OR ("spondylitis, ankylosing"[MeSH Terms] OR ("spondylitis"[All Fields] AND "ankylosing"[All Fields]) OR "ankylosing spondylitis"[All Fields] OR ("bechterew"[All Fields] AND "disease"[All Fields]) OR "bechterew disease"[All Fields]) OR ("spondylitis, ankylosing"[MeSH Terms] OR ("spondylitis"[All Fields] AND "ankylosing"[All Fields]) OR "ankylosing spondylitis"[All Fields] OR ("rheumatoid"[All Fields] AND "spondylitis"[All Fields]) OR "rheumatoid spondylitis"[All Fields]) OR "lupus erythematosus, systemic"[MeSH Terms] OR ("lupus erythematosus, systemic"[MeSH Terms] OR ("lupus"[All Fields] AND "erythematosus"[All Fields] AND "systemic"[All Fields]) OR "systemic lupus erythematosus"[All Fields] OR ("lupus"[All Fields] AND "erythematosus"[All Fields] AND "systemic"[All Fields]) OR "lupus erythematosus systemic"[All Fields]) OR ("lupus erythematosus, systemic"[MeSH Terms] OR ("lupus"[All Fields] AND "erythematosus"[All Fields] AND "systemic"[All Fields]) OR "systemic lupus erythematosus"[All Fields] OR ("systemic"[All Fields] AND "lupus"[All Fields] AND "erythematosus"[All Fields])) OR ("lupus erythematosus, systemic"[MeSH Terms] OR ("lupus"[All Fields] AND "erythematosus"[All Fields] AND "systemic"[All Fields]) OR "systemic lupus erythematosus"[All Fields] OR ("lupus"[All Fields] AND "erythematosus"[All Fields] AND "disseminatus"[All Fields]) OR "lupus erythematosus disseminatus"[All Fields]) OR ("lupus erythematosus, systemic"[MeSH Terms] OR ("lupus"[All Fields] AND "erythematosus"[All Fields] AND "systemic"[All Fields]) OR "systemic lupus erythematosus"[All Fields] OR ("libman"[All Fields] AND "sacks"[All Fields] AND "disease"[All Fields]) OR "libman sacks disease"[All Fields]) OR "Myositis"[MeSH Terms] OR ("Myositis"[MeSH Terms] OR "Myositis"[All Fields] OR "myositides"[All Fields]) OR ("Myositis"[MeSH Terms] OR "Myositis"[All Fields] OR "myositides"[All Fields]) OR ("Myositis"[MeSH Terms] OR "Myositis"[All Fields] OR ("inflammatory"[All Fields] AND "muscle"[All Fields] AND "diseases"[All Fields]) OR "inflammatory muscle diseases"[All Fields]) OR ("Myositis"[MeSH Terms] OR "Myositis"[All Fields] OR ("inflammatory"[All Fields] AND "muscle"[All Fields] AND "disease"[All Fields]) OR "inflammatory muscle disease"[All Fields]) OR ("Myositis"[MeSH Terms] OR "Myositis"[All Fields] OR ("inflammatory"[All Fields] AND "myopathy"[All Fields]) OR "inflammatory myopathy"[All Fields]) OR ("Myositis"[MeSH Terms] OR "Myositis"[All Fields] OR ("inflammatory"[All Fields] AND "myopathies"[All Fields]) OR "inflammatory myopathies"[All Fields]) OR ("Myositis"[MeSH Terms] OR "Myositis"[All Fields] OR ("proliferative"[All Fields] AND "Myositis"[All Fields]) OR "proliferative myositis"[All Fields]) OR ("Myositis"[MeSH Terms] OR "Myositis"[All Fields] OR ("infectious"[All Fields] AND "myositides"[All Fields]) OR "infectious myositides"[All Fields]) OR ("Myositis"[MeSH Terms] OR "Myositis"[All Fields] OR ("infectious"[All Fields] AND "Myositis"[All Fields]) OR "infectious myositis"[All Fields]) OR ("Myositis"[MeSH Terms] OR "Myositis"[All Fields] OR ("idiopathic"[All Fields] AND "inflammatory"[All Fields] AND "myopathies"[All Fields]) OR "idiopathic inflammatory myopathies"[All Fields]) OR ("Myositis"[MeSH Terms] OR "Myositis"[All Fields] OR ("idiopathic"[All Fields] AND "inflammatory"[All Fields] AND "myopathy"[All Fields]) OR "idiopathic inflammatory myopathy"[All Fields]) OR ("Myositis"[MeSH Terms] OR "Myositis"[All Fields] OR ("idiopathic"[All Fields] AND "inflammatory"[All Fields] AND "Myositis"[All Fields]) OR "idiopathic inflammatory myositis"[All Fields]) OR ("Myositis"[MeSH Terms] OR "Myositis"[All Fields] OR ("focal"[All Fields] AND "Myositis"[All Fields]) OR "focal myositis"[All Fields]) OR "Systemic Vasculitis"[MeSH Terms] OR ("Systemic Vasculitis"[MeSH Terms] OR ("systemic"[All Fields] AND "vasculitis"[All Fields]) OR "Systemic Vasculitis"[All Fields]) OR ("Systemic Vasculitis"[MeSH Terms] OR ("systemic"[All Fields] AND "vasculitis"[All Fields]) OR "Systemic Vasculitis"[All Fields] OR ("systemic"[All Fields] AND "vasculitides"[All Fields]) OR "systemic vasculitides"[All Fields]) OR "Sjogren's Syndrome"[MeSH Terms] OR ("Sjogren's Syndrome"[MeSH Terms] OR ("sjogren s"[All Fields] AND "syndrome"[All Fields]) OR "Sjogren's Syndrome"[All Fields]) OR ("Sjogren's Syndrome"[MeSH Terms] OR ("sjogren s"[All Fields] AND "syndrome"[All Fields]) OR "Sjogren's Syndrome"[All Fields] OR ("sjogrens"[All Fields] AND "syndrome"[All Fields]) OR "sjogrens syndrome"[All Fields]) OR ("Sjogren's Syndrome"[MeSH Terms] OR ("sjogren s"[All Fields] AND "syndrome"[All Fields]) OR "Sjogren's Syndrome"[All Fields] OR ("sjogren"[All Fields] AND "syndrome"[All Fields]) OR "sjogren syndrome"[All Fields]) OR ("Sjogren's Syndrome"[MeSH Terms] OR ("sjogren s"[All Fields] AND "syndrome"[All Fields]) OR "Sjogren's Syndrome"[All Fields] OR ("sicca"[All Fields] AND "syndrome"[All Fields]) OR "sicca syndrome"[All Fields]) OR "scleroderma, systemic"[MeSH Terms] OR ("scleroderma, systemic"[MeSH Terms] OR ("scleroderma"[All Fields] AND "systemic"[All Fields]) OR "systemic scleroderma"[All Fields] OR ("scleroderma"[All Fields] AND "systemic"[All Fields]) OR "scleroderma systemic"[All Fields]) OR ("scleroderma, systemic"[MeSH Terms] OR ("scleroderma"[All Fields] AND "systemic"[All Fields]) OR "systemic scleroderma"[All Fields] OR ("systemic"[All Fields] AND "sclerosis"[All Fields]) OR "systemic sclerosis"[All Fields]) OR ("scleroderma, systemic"[MeSH Terms] OR ("scleroderma"[All Fields] AND "systemic"[All Fields]) OR "systemic scleroderma"[All Fields] OR ("systemic"[All Fields] AND "scleroderma"[All Fields])) OR "Mixed Connective Tissue Disease"[MeSH Terms] OR ("Mixed Connective Tissue Disease"[MeSH Terms] OR ("mixed"[All Fields] AND "connective"[All Fields] AND "tissue"[All Fields] AND "disease"[All Fields]) OR "Mixed Connective Tissue Disease"[All Fields]) OR ("Mixed Connective Tissue Disease"[MeSH Terms] OR ("mixed"[All Fields] AND "connective"[All Fields] AND "tissue"[All Fields] AND "disease"[All Fields]) OR "Mixed Connective Tissue Disease"[All Fields] OR ("sharp"[All Fields] AND "syndrome"[All Fields]) OR "sharp syndrome"[All Fields]) OR ("Mixed Connective Tissue Disease"[MeSH Terms] OR ("mixed"[All Fields] AND "connective"[All Fields] AND "tissue"[All Fields] AND "disease"[All Fields]) OR "Mixed Connective Tissue Disease"[All Fields] OR "mctd"[All Fields])) AND ("vaccines, inactivated"[MeSH Terms] OR ("vaccines, inactivated"[MeSH Terms] OR ("vaccines"[All Fields] AND "inactivated"[All Fields]) OR "inactivated vaccines"[All Fields] OR ("vaccines"[All Fields] AND "inactivated"[All Fields]) OR "vaccines inactivated"[All Fields]) OR ("vaccines, inactivated"[MeSH Terms] OR ("vaccines"[All Fields] AND "inactivated"[All Fields]) OR "inactivated vaccines"[All Fields] OR ("inactivated"[All Fields] AND "vaccines"[All Fields])) OR ("vaccines, inactivated"[MeSH Terms] OR ("vaccines"[All Fields] AND "inactivated"[All Fields]) OR "inactivated vaccines"[All Fields] OR ("killed"[All Fields] AND "vaccine"[All Fields]) OR "killed vaccine"[All Fields]) OR ("vaccines, inactivated"[MeSH Terms] OR ("vaccines"[All Fields] AND "inactivated"[All Fields]) OR "inactivated vaccines"[All Fields] OR ("killed"[All Fields] AND "vaccines"[All Fields]) OR "killed vaccines"[All Fields]) OR ("vaccines, inactivated"[MeSH Terms] OR ("vaccines"[All Fields] AND "inactivated"[All Fields]) OR "inactivated vaccines"[All Fields] OR ("inactivated"[All Fields] AND "vaccine"[All Fields]) OR "inactivated vaccine"[All Fields]) OR ("influenza vaccines"[MeSH Terms] OR ("influenza"[All Fields] AND "vaccines"[All Fields]) OR "influenza vaccines"[All Fields]) OR ("influenza vaccines"[MeSH Terms] OR ("influenza"[All Fields] AND "vaccines"[All Fields]) OR "influenza vaccines"[All Fields] OR ("influenza"[All Fields] AND "vaccine"[All Fields]) OR "influenza vaccine"[All Fields]) OR ("influenza vaccines"[MeSH Terms] OR ("influenza"[All Fields] AND "vaccines"[All Fields]) OR "influenza vaccines"[All Fields] OR ("influenzavirus"[All Fields] AND "vaccines"[All Fields])) OR ("influenza vaccines"[MeSH Terms] OR ("influenza"[All Fields] AND "vaccines"[All Fields]) OR "influenza vaccines"[All Fields] OR ("influenza"[All Fields] AND "virus"[All Fields] AND "vaccines"[All Fields]) OR "influenza virus vaccines"[All Fields]) OR ("influenza vaccines"[MeSH Terms] OR ("influenza"[All Fields] AND "vaccines"[All Fields]) OR "influenza vaccines"[All Fields] OR ("influenzavirus"[All Fields] AND "vaccine"[All Fields]) OR "influenzavirus vaccine"[All Fields]) OR ("influenza vaccines"[MeSH Terms] OR ("influenza"[All Fields] AND "vaccines"[All Fields]) OR "influenza vaccines"[All Fields] OR ("influenza"[All Fields] AND "virus"[All Fields] AND "vaccine"[All Fields]) OR "influenza virus vaccine"[All Fields]) OR ("influenza vaccines"[MeSH Terms] OR ("influenza"[All Fields] AND "vaccines"[All Fields]) OR "influenza vaccines"[All Fields] OR ("high"[All Fields] AND "dose"[All Fields] AND "trivalent"[All Fields] AND "influenza"[All Fields] AND "vaccine"[All Fields]) OR "high dose trivalent influenza vaccine"[All Fields]) OR ("influenza vaccines"[MeSH Terms] OR ("influenza"[All Fields] AND "vaccines"[All Fields]) OR "influenza vaccines"[All Fields] OR ("high"[All Fields] AND "dose"[All Fields] AND "trivalent"[All Fields] AND "influenza"[All Fields] AND "vaccine"[All Fields]) OR "high dose trivalent influenza vaccine"[All Fields]) OR ("influenza vaccines"[MeSH Terms] OR ("influenza"[All Fields] AND "vaccines"[All Fields]) OR "influenza vaccines"[All Fields] OR ("trivalent"[All Fields] AND "influenza"[All Fields] AND "vaccine"[All Fields]) OR "trivalent influenza vaccine"[All Fields]) OR ("influenza vaccines"[MeSH Terms] OR ("influenza"[All Fields] AND "vaccines"[All Fields]) OR "influenza vaccines"[All Fields] OR ("quadrivalent"[All Fields] AND "influenza"[All Fields] AND "vaccine"[All Fields]) OR "quadrivalent influenza vaccine"[All Fields]) OR "Pneumococcal Vaccines"[MeSH Terms] OR ("Pneumococcal Vaccines"[MeSH Terms] OR ("pneumococcal"[All Fields] AND "vaccines"[All Fields]) OR "Pneumococcal Vaccines"[All Fields]) OR ("Pneumococcal Vaccines"[MeSH Terms] OR ("pneumococcal"[All Fields] AND "vaccines"[All Fields]) OR "Pneumococcal Vaccines"[All Fields] OR ("pneumococcal"[All Fields] AND "vaccine"[All Fields]) OR "pneumococcal vaccine"[All Fields]) OR ("Pneumococcal Vaccines"[MeSH Terms] OR ("pneumococcal"[All Fields] AND "vaccines"[All Fields]) OR "Pneumococcal Vaccines"[All Fields] OR "pneumovax"[All Fields]) OR ("Pneumococcal Vaccines"[MeSH Terms] OR ("pneumococcal"[All Fields] AND "vaccines"[All Fields]) OR "Pneumococcal Vaccines"[All Fields] OR ("pneumococcal"[All Fields] AND "polysaccharide"[All Fields] AND "vaccine"[All Fields]) OR "pneumococcal polysaccharide vaccine"[All Fields]) OR ("Pneumococcal Vaccines"[MeSH Terms] OR ("pneumococcal"[All Fields] AND "vaccines"[All Fields]) OR "Pneumococcal Vaccines"[All Fields] OR ("pnu"[All Fields] AND "imune"[All Fields] AND "vaccine"[All Fields]) OR "pnu imune vaccine"[All Fields]) OR ("Pneumococcal Vaccines"[MeSH Terms] OR ("pneumococcal"[All Fields] AND "vaccines"[All Fields]) OR "Pneumococcal Vaccines"[All Fields] OR ("pnuimune"[All Fields] AND "vaccine"[All Fields])) OR (("conjugable"[All Fields] OR "conjugal"[All Fields] OR "conjugally"[All Fields] OR "conjugant"[All Fields] OR "conjugants"[All Fields] OR "conjugate"[All Fields] OR "conjugate s"[All Fields] OR "conjugated"[All Fields] OR "conjugates"[All Fields] OR "conjugating"[All Fields] OR "conjugation"[All Fields] OR "conjugational"[All Fields] OR "conjugations"[All Fields] OR "conjugative"[All Fields] OR "conjugatively"[All Fields]) AND "penumococcal"[All Fields] AND ("vaccin"[Supplementary Concept] OR "vaccin"[All Fields] OR "vaccination"[MeSH Terms] OR "vaccination"[All Fields] OR "vaccinable"[All Fields] OR "vaccinal"[All Fields] OR "vaccinate"[All Fields] OR "vaccinated"[All Fields] OR "vaccinates"[All Fields] OR "vaccinating"[All Fields] OR "vaccinations"[All Fields] OR "vaccination s"[All Fields] OR "vaccinator"[All Fields] OR "vaccinators"[All Fields] OR "vaccine s"[All Fields] OR "vaccined"[All Fields] OR "vaccines"[MeSH Terms] OR "vaccines"[All Fields] OR "vaccine"[All Fields] OR "vaccins"[All Fields])) OR ("herpes zoster vaccine"[MeSH Terms] OR ("herpes"[All Fields] AND "zoster"[All Fields] AND "vaccine"[All Fields]) OR "herpes zoster vaccine"[All Fields]) OR ("herpes zoster vaccine"[MeSH Terms] OR ("herpes"[All Fields] AND "zoster"[All Fields] AND "vaccine"[All Fields]) OR "herpes zoster vaccine"[All Fields] OR ("zoster"[All Fields] AND "vaccine"[All Fields]) OR "zoster vaccine"[All Fields]) OR ("herpes zoster vaccine"[MeSH Terms] OR ("herpes"[All Fields] AND "zoster"[All Fields] AND "vaccine"[All Fields]) OR "herpes zoster vaccine"[All Fields] OR ("shingles"[All Fields] AND "vaccine"[All Fields]) OR "shingles vaccine"[All Fields]) OR "Hepatitis B Vaccines"[MeSH Terms] OR ("Hepatitis B Vaccines"[MeSH Terms] OR "Hepatitis B Vaccines"[All Fields]) OR ("Hepatitis B Vaccines"[MeSH Terms] OR "Hepatitis B Vaccines"[All Fields] OR "hepatitis b vaccine"[All Fields]) OR "Papillomavirus Vaccines"[MeSH Terms] OR ("Papillomavirus Vaccines"[MeSH Terms] OR ("papillomavirus"[All Fields] AND "vaccines"[All Fields]) OR "Papillomavirus Vaccines"[All Fields]) OR ("Papillomavirus Vaccines"[MeSH Terms] OR ("papillomavirus"[All Fields] AND "vaccines"[All Fields]) OR "Papillomavirus Vaccines"[All Fields] OR ("papillomavirus"[All Fields] AND "vaccine"[All Fields]) OR "papillomavirus vaccine"[All Fields]) OR ("Papillomavirus Vaccines"[MeSH Terms] OR ("papillomavirus"[All Fields] AND "vaccines"[All Fields]) OR "Papillomavirus Vaccines"[All Fields] OR ("human"[All Fields] AND "papillomavirus"[All Fields] AND "vaccines"[All Fields]) OR "human papillomavirus vaccines"[All Fields]) OR ("Papillomavirus Vaccines"[MeSH Terms] OR ("papillomavirus"[All Fields] AND "vaccines"[All Fields]) OR "Papillomavirus Vaccines"[All Fields] OR ("hpv"[All Fields] AND "vaccine"[All Fields]) OR "hpv vaccine"[All Fields]) OR ("Papillomavirus Vaccines"[MeSH Terms] OR ("papillomavirus"[All Fields] AND "vaccines"[All Fields]) OR "Papillomavirus Vaccines"[All Fields] OR ("human"[All Fields] AND "papilloma"[All Fields] AND "virus"[All Fields] AND "vaccine"[All Fields]) OR "human papilloma virus vaccine"[All Fields]) OR ("Papillomavirus Vaccines"[MeSH Terms] OR ("papillomavirus"[All Fields] AND "vaccines"[All Fields]) OR "Papillomavirus Vaccines"[All Fields] OR ("hpv"[All Fields] AND "vaccines"[All Fields]) OR "hpv vaccines"[All Fields]) OR ("Papillomavirus Vaccines"[MeSH Terms] OR ("papillomavirus"[All Fields] AND "vaccines"[All Fields]) OR "Papillomavirus Vaccines"[All Fields] OR ("human"[All Fields] AND "papilloma"[All Fields] AND "virus"[All Fields] AND "vaccines"[All Fields]) OR "human papilloma virus vaccines"[All Fields]) OR ("Papillomavirus Vaccines"[MeSH Terms] OR ("papillomavirus"[All Fields] AND "vaccines"[All Fields]) OR "Papillomavirus Vaccines"[All Fields] OR ("human"[All Fields] AND "papillomavirus"[All Fields] AND "vaccine"[All Fields]) OR "human papillomavirus vaccine"[All Fields]) OR "Poliovirus Vaccines"[MeSH Terms] OR ("Poliovirus Vaccines"[MeSH Terms] OR ("poliovirus"[All Fields] AND "vaccines"[All Fields]) OR "Poliovirus Vaccines"[All Fields]) OR ("Poliovirus Vaccines"[MeSH Terms] OR ("poliovirus"[All Fields] AND "vaccines"[All Fields]) OR "Poliovirus Vaccines"[All Fields] OR ("poliovirus"[All Fields] AND "vaccine"[All Fields]) OR "poliovirus vaccine"[All Fields]) OR "salk"[All Fields] OR "Hepatitis A Vaccines"[MeSH Terms] OR ("Hepatitis A Vaccines"[MeSH Terms] OR "Hepatitis A Vaccines"[All Fields]) OR ("Hepatitis A Vaccines"[MeSH Terms] OR "Hepatitis A Vaccines"[All Fields] OR ("hepatovirus"[All Fields] AND "vaccines"[All Fields])) OR ("Hepatitis A Vaccines"[MeSH Terms] OR "Hepatitis A Vaccines"[All Fields] OR "hepatitis a vaccine"[All Fields]) OR ("hepatitis a vaccine inactivated"[All Fields] OR "Hepatitis A Vaccines"[MeSH Terms] OR "Hepatitis A Vaccines"[All Fields] OR "vaqta"[All Fields]) OR ("Hepatitis A Vaccines"[MeSH Terms] OR "Hepatitis A Vaccines"[All Fields] OR "havrix"[All Fields]) OR "Meningococcal Vaccines"[MeSH Terms] OR ("Meningococcal Vaccines"[MeSH Terms] OR ("meningococcal"[All Fields] AND "vaccines"[All Fields]) OR "Meningococcal Vaccines"[All Fields] OR ("meningococcal"[All Fields] AND "vaccine"[All Fields]) OR "meningococcal vaccine"[All Fields]) OR ("Meningococcal Vaccines"[MeSH Terms] OR ("meningococcal"[All Fields] AND "vaccines"[All Fields]) OR "Meningococcal Vaccines"[All Fields] OR "menactra"[All Fields]) OR ("Meningococcal Vaccines"[MeSH Terms] OR ("meningococcal"[All Fields] AND "vaccines"[All Fields]) OR "Meningococcal Vaccines"[All Fields] OR ("tetravalent"[All Fields] AND "meningococcal"[All Fields] AND "vaccine"[All Fields]) OR "tetravalent meningococcal vaccine"[All Fields]) OR ("Meningococcal Vaccines"[MeSH Terms] OR ("meningococcal"[All Fields] AND "vaccines"[All Fields]) OR "Meningococcal Vaccines"[All Fields] OR "menomune"[All Fields]) OR ("Meningococcal Vaccines"[MeSH Terms] OR ("meningococcal"[All Fields] AND "vaccines"[All Fields]) OR "Meningococcal Vaccines"[All Fields] OR "menveo"[All Fields]) OR ("Meningococcal Vaccines"[MeSH Terms] OR ("meningococcal"[All Fields] AND "vaccines"[All Fields]) OR "Meningococcal Vaccines"[All Fields] OR ("bivalent"[All Fields] AND "meningococcal"[All Fields] AND "vaccine"[All Fields]) OR "bivalent meningococcal vaccine"[All Fields]) OR ("Meningococcal Vaccines"[MeSH Terms] OR ("meningococcal"[All Fields] AND "vaccines"[All Fields]) OR "Meningococcal Vaccines"[All Fields] OR ("meningococcal"[All Fields] AND "polysaccharide"[All Fields] AND "vaccine"[All Fields]) OR "meningococcal polysaccharide vaccine"[All Fields]) OR "Diphtheria-Tetanus-Pertussis Vaccine"[MeSH Terms] OR ("Diphtheria-Tetanus-Pertussis Vaccine"[MeSH Terms] OR ("diphtheria tetanus pertussis"[All Fields] AND "vaccine"[All Fields]) OR "Diphtheria-Tetanus-Pertussis Vaccine"[All Fields] OR ("diphtheria"[All Fields] AND "tetanus"[All Fields] AND "pertussis"[All Fields] AND "vaccine"[All Fields]) OR "Diphtheria-Tetanus-Pertussis Vaccine"[All Fields]) OR ("Diphtheria-Tetanus-Pertussis Vaccine"[MeSH Terms] OR ("diphtheria tetanus pertussis"[All Fields] AND "vaccine"[All Fields]) OR "Diphtheria-Tetanus-Pertussis Vaccine"[All Fields] OR ("diphtheria"[All Fields] AND "tetanus"[All Fields] AND "pertussis"[All Fields] AND "vaccine"[All Fields]) OR "Diphtheria-Tetanus-Pertussis Vaccine"[All Fields]) OR ("Diphtheria-Tetanus-Pertussis Vaccine"[MeSH Terms] OR ("diphtheria tetanus pertussis"[All Fields] AND "vaccine"[All Fields]) OR "Diphtheria-Tetanus-Pertussis Vaccine"[All Fields] OR ("dtwp"[All Fields] AND "vaccine"[All Fields]) OR "dtwp vaccine"[All Fields]) OR ("Diphtheria-Tetanus-Pertussis Vaccine"[MeSH Terms] OR ("diphtheria tetanus pertussis"[All Fields] AND "vaccine"[All Fields]) OR "Diphtheria-Tetanus-Pertussis Vaccine"[All Fields] OR ("dpt"[All Fields] AND "vaccine"[All Fields]) OR "dpt vaccine"[All Fields]) OR ("Diphtheria-Tetanus-Pertussis Vaccine"[MeSH Terms] OR ("diphtheria tetanus pertussis"[All Fields] AND "vaccine"[All Fields]) OR "Diphtheria-Tetanus-Pertussis Vaccine"[All Fields] OR ("dtp"[All Fields] AND "vaccine"[All Fields]) OR "dtp vaccine"[All Fields]) OR ("Diphtheria-Tetanus-Pertussis Vaccine"[MeSH Terms] OR ("diphtheria tetanus pertussis"[All Fields] AND "vaccine"[All Fields]) OR "Diphtheria-Tetanus-Pertussis Vaccine"[All Fields] OR ("di"[All Fields] AND "te"[All Fields] AND "per"[All Fields] AND "vaccine"[All Fields]) OR "di te per vaccine"[All Fields]) OR ("Diphtheria-Tetanus-Pertussis Vaccine"[MeSH Terms] OR ("diphtheria tetanus pertussis"[All Fields] AND "vaccine"[All Fields]) OR "Diphtheria-Tetanus-Pertussis Vaccine"[All Fields] OR ("diphtheria"[All Fields] AND "pertussis"[All Fields] AND "tetanus"[All Fields] AND "vaccine"[All Fields]) OR "diphtheria pertussis tetanus vaccine"[All Fields]) OR ("haemophilus influenza type b polysaccharide vaccine tetanus toxin conjugate"[Supplementary Concept] OR "haemophilus influenza type b polysaccharide vaccine tetanus toxin conjugate"[All Fields] OR "haemophilus influenza type b polysaccharide vaccine tetanus toxin conjugate"[All Fields]) OR ("haemophilus influenza type b polysaccharide vaccine tetanus toxin conjugate"[Supplementary Concept] OR "haemophilus influenza type b polysaccharide vaccine tetanus toxin conjugate"[All Fields] OR "prp t"[All Fields]) OR ("haemophilus influenza type b polysaccharide vaccine tetanus toxin conjugate"[Supplementary Concept] OR "haemophilus influenza type b polysaccharide vaccine tetanus toxin conjugate"[All Fields] OR "hib tt"[All Fields]) OR ("haemophilus influenza type b polysaccharide vaccine tetanus toxin conjugate"[Supplementary Concept] OR "haemophilus influenza type b polysaccharide vaccine tetanus toxin conjugate"[All Fields] OR "omnihib"[All Fields]) OR ("haemophilus influenza type b polysaccharide vaccine tetanus toxin conjugate"[Supplementary Concept] OR "haemophilus influenza type b polysaccharide vaccine tetanus toxin conjugate"[All Fields] OR "acthib"[All Fields]))

1,128 results

**1.2 Following there are search strategy in Embase**

**No.**

**Query**

**Results**

**3,124**

**#25**

**#12 AND #24**

**149,994**

**#24**

**#13 OR #14 OR #15 OR #16 OR #17 OR #18 OR #19 OR #20 OR #21 OR #22 OR #23**

**36**

**#23**

**'haemophilus influenza type b polysaccharide vaccine tetanus toxin conjugate'/syn**

**22,584**

**#22**

**'diphtheria pertussis tetanus vaccine'/syn**

**8,774**

**#21**

**'meningococcal vaccines'/syn**

**5,982**

**#20**

**'hepatitis a vaccines'/syn**

**15,713**

**#19**

**'poliovirus vaccines'/syn**

**18,091**

**#18**

**'human papilloma virus vaccine'/syn**

**25,125**

**#17**

**'hepatitis b vaccine'/syn**

**4,441**

**#16**

**'herpes zoster vaccine'/syn**

**25,896**

**#15**

**'pneumococcus vaccine'/syn**

**48,734**

**#14**

**'influenza vaccines'/syn**

**10,379**

**#13**

**'inactivated vaccine'/syn**

**582,415**

**#12**

**#1 OR #2 OR #3 OR #4 OR #5 OR #6 OR #7 OR #8 OR #9 OR #10 OR #11**

**5,506**

**#11**

**'mixed connective tissue disease'/syn**

**43,158**

**#10**

**'systemic sclerosis'/syn**

**35,919**

**#9**

**'sjoegren syndrome'/syn**

**9,231**

**#8**

**'systemic vasculitis'/syn**

**52,684**

**#7**

**'myositis'/syn**

**141,094**

**#6**

**'systemic lupus erythematosus'/syn**

**40,970**

**#5**

**'ankylosing spondylitis'/syn**

**33,640**

**#4**

**'psoriatic arthritis'/syn**

**73,128**

**#3**

**'spondylarthritis'/syn**

**282,694**

**#2**

**'rheumatoid arthritis'/syn**

**56,077**

**#1**

**'rheumatic disease'/de**

**1.3 Following there are search strategy in Cochrane Library**

ID	Search	Hits

#1	MeSH descriptor: [Rheumatic Diseases] explode all trees	20731

#2	MeSH descriptor: [Arthritis, Rheumatoid] explode all trees	7302

#3	MeSH descriptor: [Spondylarthritis] explode all trees	1823

#4	MeSH descriptor: [Arthritis, Psoriatic] explode all trees	634

#5	MeSH descriptor: [Spondylitis, Ankylosing] explode all trees	826

#6	MeSH descriptor: [Lupus Erythematosus, Systemic] explode all trees	1425

#7	MeSH descriptor: [Myositis] explode all trees	318

#8	MeSH descriptor: [Systemic Vasculitis] explode all trees	266

#9	MeSH descriptor: [Sjogren's Syndrome] explode all trees	365

#10	MeSH descriptor: [Scleroderma, Systemic] explode all trees	717

#11	MeSH descriptor: [Mixed Connective Tissue Disease] explode all trees	13

#12	#1 OR #2 OR #3 OR #4 OR #5 OR #6 OR #7 OR #8 OR #9 OR #10 OR #11	24888

#13	MeSH descriptor: [Vaccines, Inactivated] explode all trees	1148

#14	MeSH descriptor: [Influenza Vaccines] explode all trees	1853

#15	MeSH descriptor: [Pneumococcal Vaccines] explode all trees	1178

#16	MeSH descriptor: [Herpes Zoster Vaccine] explode all trees	110

#17	MeSH descriptor: [Hepatitis B Vaccines] explode all trees	1003

#18	MeSH descriptor: [Papillomavirus Vaccines] explode all trees	549

#19	MeSH descriptor: [Poliovirus Vaccines] explode all trees	544

#20	MeSH descriptor: [Hepatitis A Vaccines] explode all trees	258

#21	MeSH descriptor: [Meningococcal Vaccines] explode all trees	438

#22	MeSH descriptor: [Diphtheria-Tetanus-Pertussis Vaccine] explode all trees	585

#23	#13 OR #14 OR #15 OR #16 OR #17 OR #18 OR #19 OR #20 OR #21 OR #22	5937

#24	#12 AND #23	62

**QUESTION 5. What is the effect of bDMARDs use (except RTX) on the response to inactivated vaccines in patients with immune-mediated rheumatic diseases (IMRD)?**

**Table S1.** Search strategy by database.

| **Database** | **Group** | **Terms** |
| --- | --- | --- |
| PubMed/MEDLINE | Population | "Rheumatic Diseases" [Mesh:NoExp] OR "Rheumatic Disease" OR "Rheumatism" OR "Immune-mediated rheumatic diseases" OR "Arthritis, Rheumatoid" [Mesh:NoExp] OR "Rheumatoid Arthritis" OR "Rheumatoid Nodule" OR "Rheumatoid Vasculitis" OR "Sjogren's Syndrome" OR "Spondylarthritis" [Mesh] OR "Spondylarthritis" OR "Spondylarthritides" OR "Spinal Arthritis" OR "Spinal Arthritides" OR "Arthritis, Psoriatic" [Mesh] OR "Arthritis, Psoriatic" OR "Arthritic Psoriasis" OR "Psoriatic Arthritis" OR "Psoriasis Arthropathica" OR "Psoriatic Arthropathy" OR "Psoriatic Arthropathies" OR "Spondylitis, Ankylosing" [Mesh] OR "Spondylitis, Ankylosing" OR "Bechterew's Disease" OR "Bechterews Disease" OR "Marie Struempell Disease" OR "Spondylarthritis Ankylopoietica" OR "Spondyloarthritis Ankylopoietica" OR "Ankylosing Spondylitis" OR "Ankylosing Spondylarthritis" OR "Ankylosing Spondylarthritides" OR "Ankylosing Spondyloarthritis" OR "Ankylosing Spondyloarthritides" OR "Spondylitis Ankylopoietica" OR "Bechterew Disease" OR "Rheumatoid Spondylitis" OR "Lupus Erythematosus, Systemic" [Mesh] OR "Lupus Erythematosus, Systemic" OR "Systemic Lupus Erythematosus " OR "Lupus Erythematosus Disseminatus" OR "Libman Sacks Disease" OR "Myositis" [Mesh] OR "Myositis" OR "Myositides" OR "Inflammatory Muscle Diseases" OR "Inflammatory Muscle Disease" OR "Inflammatory Myopathy" OR "Inflammatory Myopathies" OR "Proliferative Myositides" OR "Proliferative Myositis" OR "Infectious Myositides" OR "Infectious Myositis" OR "Idiopathic Inflammatory Myopathies" OR "Idiopathic Inflammatory Myopathy" OR "Idiopathic Inflammatory Myositis" OR "Focal Myositides" OR "Focal Myositis" OR "Systemic Vasculitis" [Mesh] OR "Systemic Vasculitis" OR "Systemic Vasculitides" OR "Sjogren's Syndrome" [Mesh] OR "Sjogren's Syndrome" OR "Sjogrens Syndrome" OR "Sjogren Syndrome" OR "Sicca Syndrome" OR "Scleroderma, Systemic" [Mesh] OR "Scleroderma, Systemic" OR "Systemic Sclerosis" OR "Systemic Scleroderma" OR "Mixed Connective Tissue Disease" [Mesh] OR "Mixed Connective Tissue Disease" OR "Sharp Syndrome" OR "MCTD" |
|  | Drugs | "Antibodies, Monoclonal" [Mesh] OR "Antibodies, Monoclonal" OR "Monoclonal Antibodies" OR "Monoclonal Antibody" OR "veltuzumab" [Mesh] OR "veltuzumab" OR "HA20" OR "monoclonal antibody" OR "IMMU-106" OR "IMMU 106" OR "obinutuzumab" [Mesh] OR "obinutuzumab" OR "RO 5072759" OR "R 7159" OR "GA 101" OR "GA101 antibody" OR "Gazyva" OR "gazyvaro" OR "afutuzumab" OR "Antibodies, Monoclonal, Humanized" [Mesh] OR "Antibodies, Monoclonal, Humanized" OR "Humanized Antibodies" OR "ocrelizumab" [Mesh] OR "ocrelizumab" OR "Ocrevus" OR "R 1594" OR "PR 070769" OR "ofatumumab" [Mesh] OR "ofatumumab" OR "kesimpta" OR "HUMAX-CD20-2F2" OR "GSKI841157" OR "arzerra" OR "belimumab" [Mesh] OR "belimumab" OR "BEL114333" OR "HGS1006" OR "LymphoStat-B" OR "GSK1550188" OR "Benlysta" OR "rilonacept" [Mesh] OR "rilonacept" OR "interleukin-1 Trap" OR "Interleukin-1" [Mesh] OR "Interleukin-1" OR "Interleukin 1" OR "IL-1" OR "T Helper Factor" OR "Lymphocyte Activating Factor" OR "Macrophage Cell Factor" OR "Epidermal Cell Derived Thymocyte Activating Factor" OR "Interleukin I" OR "canakinumab" [Mesh] OR "canakinumab" OR "Ilaris" OR "ACZ885" OR "immunoglobulin G1, anti-”human interleukin 1beta) “human clone ACZ885 heavy chain V region)" OR "Interleukin-6" [Mesh] OR "Interleukin-6" OR "Interleukin 6" OR "IL6" OR "B-Cell Stimulatory Factor 2" OR "B-Cell Stimulatory Factor-2" OR "B-Cell Differentiation Factor-2" OR "B Cell Differentiation Factor 2" OR "BSF-2" OR "Hybridoma Growth Factor" OR "IFN-beta 2" OR "Plasmacytoma Growth Factor" OR "Hepatocyte Stimulating Factor" OR "MGI-2" OR "Myeloid Differentiation-Inducing Protein" OR "Myeloid Differentiation Inducing Protein" OR "B Cell Differentiation Factor" OR "IL-6" OR "Interferon beta-2" OR "Interferon beta 2" OR "B Cell Stimulatory Factor 2" OR "tocilizumab" [Mesh] OR "tocilizumab" OR "atlizumab" OR "monoclonal antibody, MRA" OR "BAT1806" OR "MSB11456" OR "RG-1569" OR "R-1569" OR "Actemra" OR "Roactemra" OR "RHPM-1" OR "Tumor Necrosis Factor Inhibitors " [Mesh] OR "Tumor Necrosis Factor Inhibitors" OR "Tumor Necrosis Factor Blockers" OR "TNF Inhibitors" OR "TNF Blockers" OR "Tumor Necrosis Factor Blocker" OR "Tumor Necrosis Factor Antagonist" OR "Tumor Necrosis Factor Inhibitor" OR "TNF Antagonist" OR "TNF Blocker" OR "TNF Inhibitor" OR "Tumor Necrosis Factor Antagonists" OR "TNF Antagonists" OR "Tumor Necrosis Factor-a “TNF-a) Antagonists" OR "Tumor Necrosis Factor-a “TNF-a) Antagonist" OR "Tumor Necrosis Factor-a “TNF-a) Inhibitors" OR "Tumor Necrosis Factor-a “TNF-a) Inhibitor" OR "Tumor Necrosis Factor-a “TNF-a) Blockers" OR "Tumor Necrosis Factor-a “TNF-a) Blocker" OR "Etanercept" [Mesh] OR "Etanercept" OR "TNFR-Fc Fusion Protein" OR "TNFR Fc Fusion Protein" OR "TNR 001" OR "TNT Receptor Fusion Protein" OR "TNTR-Fc" OR "TNR-001" OR "TNR001" OR "Etanercept-szzs" OR "TNF Receptor Type II-IgG Fusion Protein" OR "TNF Receptor Type II IgG Fusion Protein" OR "Erelzi" OR "Recombinant Human Dimeric TNF Receptor Type II-IgG Fusion Protein" OR "Recombinant Human Dimeric TNF" OR "Receptor Type II IgG Fusion Protein" OR "Enbrel" OR "Infliximab" [Mesh] OR "Infliximab" OR "MAb cA2" OR "Monoclonal Antibody cA2" OR "Infliximab dyyb" OR "Inflectra" OR "Remicade" OR "Infliximab abda" OR "Renflexis" OR "Adalimumab" [Mesh] OR "Adalimumab" OR "Humira" OR "Adalimumab-adbm" OR "Amjevita" OR "Adalimumab-atto" OR "Cyltezo" OR "D2E7 Antibody" OR "golimumab" [Mesh] OR "golimumab" OR "CNTO 148" OR "Simponi" OR "Certolizumab Pegol" [Mesh] OR "Certolizumab Pegol" OR "Cimzia" OR "CDP 870" OR "Abatacept" [Mesh] OR "Abatacept" OR "CTLA-4-Ig" OR "Cytotoxic T Lymphocyte-Associated Antigen 4-Immunoglobulin" OR "Cytotoxic T Lymphocyte Associated Antigen 4 Immunoglobulin" OR "CTLA4-Ig Immunoconjugate" OR "CTLA4 Ig" OR "BMS 224818" OR "Belatacept" OR "Orencia" OR "BMS 188667" OR "LEA29Y" OR "Nulojix" OR "CTLA-4 Antigen" [Mesh] OR "CTLA-4 Antigen" OR " CTLA 4 Antigen" OR "Cytotoxic T-Lymphocyte-Associated Antigen 4" OR "Cytotoxic T Lymphocyte Associated Antigen 4" OR "CD152 Antigens" OR "CD152 Antigen" OR "CTLA-4 Protein" OR "CTLA 4 Protein" OR "Cytotoxic T-Lymphocyte Antigen 4" OR "Cytotoxic T Lymphocyte Antigen 4" OR "secukinumab" [Mesh] OR "secukinumab" OR "Cosentyx" OR "AIN 457" OR "AIN457" OR "Interleukin-17" [Mesh] OR "Interleukin-17" OR " Interleukin 17" OR "CTLA-8" OR "IL-17" OR "Interleukin-17F" OR "Interleukin 17F" OR "IL-17F" OR "Cytokine ML-1" OR "Cytokine ML 1" OR "Interleukin-17C" OR "Interleukin 17C" OR "IL-17C" OR "Cytokine CX2" OR "Interleukin-17E" OR "Interleukin 17E" OR "Interleukin 25" OR "IL-17E" OR "IL 17E" OR "Interleukin-17A" OR "Interleukin 17A" OR "Cytotoxic T lymphocyte Associated Antigen 8" OR "IL-17A" OR "CTLA8" OR "Interleukin 17B" OR "IL-17B" OR "ixekizumab" [Mesh] OR "ixekizumab" OR "Taltz" OR "LY2439821" OR "brodalumab" [Mesh] OR "brodalumab" OR "Siliq" OR "KHK4827" OR "AMG 827" OR "Ustekinumab" [Mesh] OR "Ustekinumab" OR "Stelara" OR "CNTO 1275" OR "Interleukin-12" [Mesh] OR "Interleukin-12" OR "Natural Killer Cell Stimulatory Factor" OR "IL-12" OR "Cytotoxic Lymphocyte Maturation Factor" OR "IL 12" OR "Interleukin 12 p70" OR "Interleukin 12" OR "IL12" OR "Edodekin Alfa" OR "Interleukin-23" [Mesh] OR "Interleukin-23" OR "Interleukin 23" OR "IL-23" OR "risankizumab" [Mesh] OR "risankizumab" OR " skyrizi" OR "risankizumab-rzaa" OR "BI 655066" OR "guselkumab" [Mesh] OR "guselkumab" OR "Tremfya" OR "CNTO 1959" OR "vedolizumab" [Mesh] OR "vedolizumab" OR "Entyvio" OR "MLN0002" OR "MLN02" OR "MLN-0002" OR "Integrins" [Mesh] OR "Integrins" OR "Integrin" NOT "Rituximab" [Mesh] |
|  | Vaccines | "Vaccines, Inactivated" [Mesh] OR "Vaccines, Inactivated" OR "Inactivated Vaccines" OR "Killed Vaccine" OR "Killed Vaccines" OR "Inactivated Vaccine" OR "Influenza Vaccines" [Mesh:NoExp] OR Influenza Vaccine" OR "Influenzavirus Vaccines" OR "Influenza Virus Vaccines" OR "Influenzavirus Vaccine" OR "Influenza Virus Vaccine" OR "High-Dose Trivalent Influenza Vaccine" OR "High Dose Trivalent Influenza Vaccine" OR "Trivalent Influenza Vaccine" OR "Quadrivalent Influenza Vaccine" OR "Pneumococcal Vaccines" [Mesh] OR "Pneumococcal Vaccines" OR "Pneumococcal Vaccine" OR "Pneumovax" OR "Pneumococcal Polysaccharide Vaccine" OR "Pnu Imune Vaccine" OR "PnuImune Vaccine" OR "Conjugated penumococcal vaccine" OR "Herpes Zoster Vaccine" [Mesh:NoExp] OR " Zoster Vaccine" OR "Shingles Vaccine" OR "Hepatitis B Vaccines" [Mesh] OR "Hepatitis B Vaccines" OR "Hepatitis B Vaccine" OR "Papillomavirus Vaccines" [Mesh] OR "Papillomavirus Vaccines" OR "Papillomavirus Vaccine" OR "Human Papillomavirus Vaccines" OR "HPV Vaccine" OR "Human Papilloma Virus Vaccine" OR "HPV Vaccines" OR "Human Papilloma Virus Vaccines" OR "Human Papillomavirus Vaccine" OR "Poliovirus Vaccines" [Mesh] OR "Poliovirus Vaccines" OR "Poliovirus Vaccine" OR "salk" OR "Hepatitis A Vaccines" [Mesh] OR "Hepatitis A Vaccines" OR "Hepatovirus Vaccines" OR "Hepatitis A Vaccine" OR "Vaqta" OR "Havrix" OR "Meningococcal Vaccines" [Mesh] OR " Meningococcal Vaccine" OR "Menactra" OR "Tetravalent Meningococcal Vaccine" OR "Menomune OR "Menveo" OR "Bivalent Meningococcal Vaccine" OR "Meningococcal Polysaccharide Vaccine" OR "Diphtheria-Tetanus-Pertussis Vaccine" [Mesh] OR "Diphtheria-Tetanus-Pertussis Vaccine" OR " Diphtheria Tetanus Pertussis Vaccine" OR "DTwP Vaccine" OR "DPT Vaccine" OR "DTP Vaccine" OR "Di Te Per Vaccine" OR "Diphtheria Pertussis Tetanus Vaccine" OR "Haemophilus influenza type b polysaccharide vaccine-tetanus toxin conjugate" OR "HIBest" OR "PRP-T" OR "Hib-TT" OR "Hib capsular polysaccharide-tetanus vaccine" OR "Hib CPS-tetanus vaccine" OR "OmniHIB" OR "ActHIB" |
| Embase | Population | Rheumatic Disease OR Rheumatism OR Rheumatoid Arthritis OR Spondylarthritides OR Spinal Arthritis OR Spinal Arthritides OR Arthritic Psoriasis OR Psoriatic Arthritis OR Psoriasis Arthropathica OR Psoriatic Arthropathy OR Psoriatic Arthropathies OR Bechterew's Disease OR Bechterews Disease OR Marie Struempell Disease OR Spondylarthritis Ankylopoietica OR Spondyloarthritis Ankylopoietica OR Ankylosing Spondylitis OR Ankylosing Spondylarthritis OR Ankylosing Spondylarthritides OR Ankylosing Spondyloarthritis OR Ankylosing Spondyloarthritides OR Spondylitis Ankylopoietica OR Bechterew Disease OR Rheumatoid Spondylitis OR Systemic Lupus Erythematosus OR Lupus Erythematosus Disseminatus OR Libman Sacks Disease OR Myositides OR Inflammatory Muscle Diseases OR Inflammatory Muscle Disease OR Inflammatory Myopathy OR Inflammatory Myopathies OR Proliferative Myositides OR Proliferative Myositis OR Infectious Myositides OR Infectious Myositis OR Idiopathic Inflammatory Myopathies OR Idiopathic Inflammatory Myopathy OR Idiopathic Inflammatory Myositis OR Focal Myositides OR Focal Myositis OR Systemic Vasculitides OR Sjogrens Syndrome OR Sjogren Syndrome OR Sicca Syndrome OR Systemic Sclerosis OR Systemic Scleroderma OR Sharp Syndrome OR MCTD |
|  | Drugs | Monoclonal Antibodies OR Monoclonal Antibody OR HA20 OR monoclonal antibody IMMU-106 OR IMMU 106 OR RO 5072759 OR R 7159 OR GA 101 OR GA101 antibody Gazyva OR gazyvaro OR afutuzumab OR Humanized Antibodies OR Ocrevus OR R 1594 OR PR 070769 OR kesimpta OR HUMAX-CD20-2F2 OR GSKI841157 OR arzerra OR BEL114333 OR HGS1006 OR LymphoStat-B OR GSK1550188 OR Benlysta OR interleukin-1 Trap OR Interleukin 1 OR IL-1 OR T Helper Factor OR Lymphocyte Activating Factor OR Macrophage Cell Factor OR Epidermal Cell Derived Thymocyte Activating Factor OR Interleukin I OR Ilaris OR ACZ885 OR immunoglobulin G1, anti-”human interleukin 1beta) “human clone ACZ885 heavy chain V region) OR Interleukin 6 OR IL6 OR B-Cell Stimulatory Factor 2 OR B-Cell Stimulatory Factor-2 OR B-Cell Differentiation Factor-2 OR B Cell Differentiation Factor 2 OR BSF-2 OR Hybridoma Growth Factor OR IFN-beta 2 OR Plasmacytoma Growth Factor OR Hepatocyte Stimulating Factor OR MGI-2 OR Myeloid Differentiation-Inducing Protein OR Myeloid Differentiation Inducing Protein OR B Cell Differentiation Factor OR IL-6 OR Interferon beta-2 OR Interferon beta 2 OR B Cell Stimulatory Factor 2 OR atlizumab OR monoclonal antibody, MRA OR BAT1806 OR MSB11456 OR RG-1569 OR R-1569 OR Actemra OR Roactemra OR RHPM-1 OR Tumor Necrosis Factor Blockers OR TNF Inhibitors OR TNF Blockers OR Tumor Necrosis Factor Blocker OR Tumor Necrosis Factor Antagonist OR Tumor Necrosis Factor Inhibitor OR TNF Antagonist OR TNF Blocker OR TNF Inhibitor OR Tumor Necrosis Factor Antagonists OR TNF Antagonists OR Tumor Necrosis Factor-a “TNF-a) Antagonists OR Tumor Necrosis Factor-a “TNF-a) Antagonist OR Tumor Necrosis Factor-a “TNF-a) Inhibitors OR Tumor Necrosis Factor-a “TNF-a) Inhibitor OR Tumor Necrosis Factor-a “TNF-a) Blockers OR Tumor Necrosis Factor-a “TNF-a) Blocker OR TNFR-Fc Fusion Protein OR TNFR Fc Fusion Protein OR TNR 001 OR TNT Receptor Fusion Protein OR TNTR-Fc OR TNR-001 OR TNR001 OR Etanercept-szzs OR TNF Receptor Type II-IgG Fusion Protein 9OR TNF Receptor Type II IgG Fusion Protein OR Erelzi OR Recombinant Human Dimeric TNF Receptor Type II-IgG Fusion Protein OR Recombinant Human Dimeric TNF Receptor Type II IgG Fusion Protein OR Enbrel OR MAb cA2 OR Monoclonal Antibody cA2 OR Infliximab dyyb OR Inflectra OR Remicade OR Infliximab abda OR Renflexis OR Humira OR Adalimumab-adbm OR Amjevita OR Adalimumab-atto OR Cyltezo OR D2E7 Antibody OR CNTO 148 OR Simponi OR Cimzia OR CDP 870 OR CTLA-4-Ig OR Cytotoxic T Lymphocyte-Associated Antigen 4-Immunoglobulin OR Cytotoxic T Lymphocyte Associated Antigen 4 Immunoglobulin OR CTLA4-Ig Immunoconjugate OR CTLA4 Ig OR BMS 224818 OR Belatacept OR Orencia OR BMS 188667 OR LEA29Y OR Nulojix OR CTLA 4 Antigen OR Cytotoxic T-Lymphocyte-Associated Antigen 4 OR Cytotoxic T Lymphocyte Associated Antigen 4 OR CD152 Antigens OR CD152 Antigen OR CTLA-4 Protein OR CTLA 4 Protein OR Cytotoxic T-Lymphocyte Antigen 4 OR Cytotoxic T Lymphocyte Antigen 4 OR Cosentyx OR AIN 457 OR AIN457 OR Interleukin 17 OR CTLA-8 OR IL-17 OR Interleukin-17F OR Interleukin 17F OR IL-17F OR Cytokine ML-1 OR Cytokine ML 1 OR Interleukin-17C OR Interleukin 17C OR IL-17C OR Cytokine CX2 OR Interleukin-17E OR Interleukin 17E OR Interleukin 25 OR IL-17E OR IL 17E OR Interleukin-17A OR Interleukin 17A OR Cytotoxic T lymphocyte Associated Antigen 8 OR IL-17A OR CTLA8 OR Interleukin 17B OR IL-17B OR Taltz OR LY2439821 OR Siliq OR KHK4827 OR AMG 827 OR Stelara OR CNTO 1275 OR Natural Killer Cell Stimulatory Factor OR IL-12 OR Cytotoxic Lymphocyte Maturation Factor OR IL 12 OR Interleukin 12 p70 OR Interleukin 12 OR IL12 OR Edodekin Alfa OR Interleukin 23 OR IL-23 OR skyrizi OR risankizumab-rzaa OR BI 655066 OR Tremfya OR CNTO 1959 OR Entyvio OR MLN0002 OR MLN02 OR MLN-0002 OR Integrin |
|  | Vaccines | Inactivated Vaccines OR Killed Vaccine OR Killed Vaccines OR Inactivated Vaccine OR Influenza Vaccine OR Influenzavirus Vaccines OR Influenza Virus Vaccines OR Influenzavirus Vaccine OR Influenza Virus Vaccine OR High-Dose Trivalent Influenza Vaccine OR High Dose Trivalent Influenza Vaccine OR Trivalent Influenza Vaccine OR Quadrivalent Influenza Vaccine OR High dose OR Pneumococcal Vaccine OR Pneumovax OR Pneumococcal Polysaccharide Vaccine OR Pnu Imune Vaccine OR PnuImune Vaccine OR Conjugated penumococcal vaccine OR Zoster Vaccine OR Shingles Vaccine OR Hepatitis B Vaccine OR Papillomavirus Vaccine Human Papillomavirus Vaccines OR HPV Vaccine OR Human Papilloma Virus Vaccine OR HPV Vaccines OR Human Papilloma Virus Vaccines OR Human Papillomavirus Vaccine OR Poliovirus Vaccine OR salk OR Hepatovirus Vaccines OR Hepatitis A Vaccine OR Vaqta OR Havrix OR Meningococcal Vaccine OR Menactra OR Tetravalent Meningococcal Vaccine OR Menomune OR Menveo OR Bivalent Meningococcal Vaccine OR Meningococcal Polysaccharide Vaccine OR Diphtheria Tetanus Pertussis Vaccine OR DTwP Vaccine OR DPT Vaccine DTP Vaccine OR Di Te Per Vaccine OR Diphtheria Pertussis Tetanus Vaccine OR HIBest OR PRP-T OR Hib-TT OR Hib capsular polysaccharide-tetanus vaccine OR Hib CPS-tetanus vaccine OR OmniHIB OR ActHIB |

**QUESTION 6 - What is the impact of the RTX use on the response to inactivated vaccine in patients with immune-mediated rheumatic diseases?**

1.1 Following there are search strategy in PubMed/MEDLINE

**P = Patients with immune-mediated rheumatic diseases**

(Rheumatic Diseases) OR (Rheumatic Disease) OR (Rheumatism) OR (Immune-mediated rheumatic diseases) OR (Arthritis, Rheumatoid) OR (Rheumatoid Arthritis) OR (Rheumatoid Nodule) OR (Rheumatoid Vasculitis) OR (Sjogren's Syndrome) OR "Spondylarthritis" [Mesh] OR (Spondylarthritis) OR (Spondylarthritides) OR (Spinal Arthritis) OR "Arthritis, Psoriatic" [Mesh] OR (Arthritis, Psoriatic) OR (Arthritic Psoriasis) OR (Psoriatic Arthritis) OR (Psoriasis Arthropathica) OR (Psoriatic Arthropathy) OR (Psoriatic Arthropathies) OR "Spondylitis, Ankylosing" [Mesh] OR (Spondylitis, Ankylosing) OR (Bechterew's Disease) OR (Bechterews Disease) OR (Marie Struempell Disease) OR (Spondylarthritis Ankylopoietica) OR (Spondyloarthritis Ankylopoietica) OR (Ankylosing Spondylitis) OR (Ankylosing Spondylarthritis) OR (Ankylosing Spondylarthritides) OR (Ankylosing Spondyloarthritis) OR (Spondylitis Ankylopoietica) OR (Bechterew Disease) OR (Rheumatoid Spondylitis) OR "Lupus Erythematosus, Systemic" [Mesh] OR (Lupus Erythematosus, Systemic) OR (Systemic Lupus Erythematosus) OR (Lupus Erythematosus Disseminatus) OR (Libman Sacks Disease) OR "Myositis" [Mesh] OR (Myositis) OR (Myositides) OR (Inflammatory Muscle Diseases) OR (Inflammatory Muscle Disease) OR (Inflammatory Myopathy) OR (Inflammatory Myopathies) OR (Proliferative Myositis) OR (Infectious Myositides) OR (Infectious Myositis) OR (Idiopathic Inflammatory Myopathies) OR (Idiopathic Inflammatory Myopathy) OR (Idiopathic Inflammatory Myositis) OR (Focal Myositis) OR "Systemic Vasculitis" [Mesh] OR (Systemic Vasculitis) OR (Systemic Vasculitides) OR "Sjogren's Syndrome" [Mesh] OR (Sjogren's Syndrome) OR (Sjogrens Syndrome) OR (Sjogren Syndrome) OR (Sicca Syndrome) OR "Scleroderma, Systemic" [Mesh] OR (Scleroderma, Systemic) OR (Systemic Sclerosis) OR (Systemic Scleroderma) OR "Mixed Connective Tissue Disease" [Mesh] OR (Mixed Connective Tissue Disease) OR (Sharp Syndrome) OR (MCTD)

**I =**

"Rituximab" [Mesh] OR (Rituximab) OR (Rituximab CD20 Antibody) OR (Mabthera) OR (IDEC C2B8 Antibody) OR (IDEC C2B8) OR (GP2013) OR (Rituxan)

**O = Vaccination with inactivated vaccine**

"Vaccines, Inactivated" [Mesh] OR (Vaccines, Inactivated) OR (Inactivated Vaccines) OR (Killed Vaccine) OR (Killed Vaccines) OR (Inactivated Vaccine) OR (Influenza Vaccines) OR (Influenza Vaccine) OR (Influenzavirus Vaccines) OR (Influenza Virus Vaccines) OR (Influenzavirus Vaccine) OR (Influenza Virus Vaccine) OR (High-Dose Trivalent Influenza Vaccine) OR (High Dose Trivalent Influenza Vaccine) OR (Trivalent Influenza Vaccine) OR (Quadrivalent Influenza Vaccine) OR "Pneumococcal Vaccines" [Mesh] OR (Pneumococcal Vaccines) OR (Pneumococcal Vaccine) OR (Pneumovax) OR (Pneumococcal Polysaccharide Vaccine) OR (Pnu Imune Vaccine) OR (PnuImune Vaccine) OR (Conjugated penumococcal vaccine) OR (Herpes Zoster Vaccine) OR (Zoster Vaccine) OR (Shingles Vaccine) OR "Hepatitis B Vaccines" [Mesh] OR (Hepatitis B Vaccines) OR (Hepatitis B Vaccine) OR "Papillomavirus Vaccines" [Mesh] OR (Papillomavirus Vaccines) OR (Papillomavirus Vaccine) OR (Human Papillomavirus Vaccines) OR (HPV Vaccine) OR (Human Papilloma Virus Vaccine) OR (HPV Vaccines) OR (Human Papilloma Virus Vaccines) OR (Human Papillomavirus Vaccine) OR "Poliovirus Vaccines" [Mesh] OR (Poliovirus Vaccines) OR (Poliovirus Vaccine) OR (salk) OR "Hepatitis A Vaccines" [Mesh] OR (Hepatitis A Vaccines) OR (Hepatovirus Vaccines) OR (Hepatitis A Vaccine) OR (Vaqta) OR (Havrix) OR "Meningococcal Vaccines" [Mesh] OR (Meningococcal Vaccine) OR (Menactra) OR (Tetravalent Meningococcal Vaccine) OR (Menomune) OR (Menveo) OR (Bivalent Meningococcal Vaccine) OR (Meningococcal Polysaccharide Vaccine) OR "Diphtheria-Tetanus-Pertussis Vaccine" [Mesh] OR (Diphtheria-Tetanus-Pertussis Vaccine) OR (Diphtheria Tetanus Pertussis Vaccine) OR (DTwP Vaccine) OR (DPT Vaccine) OR (DTP Vaccine) OR (Di Te Per Vaccine) OR (Diphtheria Pertussis Tetanus Vaccine) OR (Haemophilus influenza type b polysaccharide vaccine-tetanus toxin conjugate) OR (PRP-T) OR (Hib-TT) OR (OmniHIB) OR (ActHIB)

#1 AND #2 AND #3

("rheumatic diseases"[MeSH Terms] OR ("rheumatic"[All Fields] AND "diseases"[All Fields]) OR "rheumatic diseases"[All Fields] OR ("rheumatic diseases"[MeSH Terms] OR ("rheumatic"[All Fields] AND "diseases"[All Fields]) OR "rheumatic diseases"[All Fields] OR ("rheumatic"[All Fields] AND "disease"[All Fields]) OR "rheumatic disease"[All Fields]) OR ("rheumatic diseases"[MeSH Terms] OR ("rheumatic"[All Fields] AND "diseases"[All Fields]) OR "rheumatic diseases"[All Fields] OR "rheumatics"[All Fields] OR "rheumatism"[All Fields] OR "rheumatic"[All Fields] OR "rheumatisms"[All Fields]) OR ("Immune-mediated"[All Fields] AND ("rheumatic diseases"[MeSH Terms] OR ("rheumatic"[All Fields] AND "diseases"[All Fields]) OR "rheumatic diseases"[All Fields])) OR ("arthritis, rheumatoid"[MeSH Terms] OR ("arthritis"[All Fields] AND "rheumatoid"[All Fields]) OR "rheumatoid arthritis"[All Fields] OR ("arthritis"[All Fields] AND "rheumatoid"[All Fields]) OR "arthritis rheumatoid"[All Fields]) OR ("arthritis, rheumatoid"[MeSH Terms] OR ("arthritis"[All Fields] AND "rheumatoid"[All Fields]) OR "rheumatoid arthritis"[All Fields] OR ("rheumatoid"[All Fields] AND "arthritis"[All Fields])) OR ("rheumatoid nodule"[MeSH Terms] OR ("rheumatoid"[All Fields] AND "nodule"[All Fields]) OR "rheumatoid nodule"[All Fields]) OR ("rheumatoid vasculitis"[MeSH Terms] OR ("rheumatoid"[All Fields] AND "vasculitis"[All Fields]) OR "rheumatoid vasculitis"[All Fields]) OR ("Sjogren's Syndrome"[MeSH Terms] OR ("sjogren s"[All Fields] AND "syndrome"[All Fields]) OR "Sjogren's Syndrome"[All Fields]) OR "Spondylarthritis"[MeSH Terms] OR ("Spondylarthritis"[MeSH Terms] OR "Spondylarthritis"[All Fields] OR "spondylarthritides"[All Fields]) OR ("Spondylarthritis"[MeSH Terms] OR "Spondylarthritis"[All Fields] OR "spondylarthritides"[All Fields]) OR ("Spondylarthritis"[MeSH Terms] OR "Spondylarthritis"[All Fields] OR ("spinal"[All Fields] AND "arthritis"[All Fields]) OR "spinal arthritis"[All Fields]) OR "arthritis, psoriatic"[MeSH Terms] OR ("arthritis, psoriatic"[MeSH Terms] OR ("arthritis"[All Fields] AND "psoriatic"[All Fields]) OR "psoriatic arthritis"[All Fields] OR ("arthritis"[All Fields] AND "psoriatic"[All Fields]) OR "arthritis psoriatic"[All Fields]) OR ("arthritis, psoriatic"[MeSH Terms] OR ("arthritis"[All Fields] AND "psoriatic"[All Fields]) OR "psoriatic arthritis"[All Fields] OR ("arthritic"[All Fields] AND "psoriasis"[All Fields]) OR "arthritic psoriasis"[All Fields]) OR ("arthritis, psoriatic"[MeSH Terms] OR ("arthritis"[All Fields] AND "psoriatic"[All Fields]) OR "psoriatic arthritis"[All Fields] OR ("psoriatic"[All Fields] AND "arthritis"[All Fields])) OR ("arthritis, psoriatic"[MeSH Terms] OR ("arthritis"[All Fields] AND "psoriatic"[All Fields]) OR "psoriatic arthritis"[All Fields] OR ("psoriasis"[All Fields] AND "arthropathica"[All Fields]) OR "psoriasis arthropathica"[All Fields]) OR ("arthritis, psoriatic"[MeSH Terms] OR ("arthritis"[All Fields] AND "psoriatic"[All Fields]) OR "psoriatic arthritis"[All Fields] OR ("psoriatic"[All Fields] AND "arthropathy"[All Fields]) OR "psoriatic arthropathy"[All Fields]) OR ("arthritis, psoriatic"[MeSH Terms] OR ("arthritis"[All Fields] AND "psoriatic"[All Fields]) OR "psoriatic arthritis"[All Fields] OR ("psoriatic"[All Fields] AND "arthropathies"[All Fields]) OR "psoriatic arthropathies"[All Fields]) OR "spondylitis, ankylosing"[MeSH Terms] OR ("spondylitis, ankylosing"[MeSH Terms] OR ("spondylitis"[All Fields] AND "ankylosing"[All Fields]) OR "ankylosing spondylitis"[All Fields] OR ("spondylitis"[All Fields] AND "ankylosing"[All Fields]) OR "spondylitis ankylosing"[All Fields]) OR ("spondylitis, ankylosing"[MeSH Terms] OR ("spondylitis"[All Fields] AND "ankylosing"[All Fields]) OR "ankylosing spondylitis"[All Fields] OR ("bechterew s"[All Fields] AND "disease"[All Fields]) OR "bechterew s disease"[All Fields]) OR ("spondylitis, ankylosing"[MeSH Terms] OR ("spondylitis"[All Fields] AND "ankylosing"[All Fields]) OR "ankylosing spondylitis"[All Fields] OR ("bechterews"[All Fields] AND "disease"[All Fields]) OR "bechterews disease"[All Fields]) OR ("spondylitis, ankylosing"[MeSH Terms] OR ("spondylitis"[All Fields] AND "ankylosing"[All Fields]) OR "ankylosing spondylitis"[All Fields] OR ("marie"[All Fields] AND "struempell"[All Fields] AND "disease"[All Fields]) OR "marie struempell disease"[All Fields]) OR ("spondylitis, ankylosing"[MeSH Terms] OR ("spondylitis"[All Fields] AND "ankylosing"[All Fields]) OR "ankylosing spondylitis"[All Fields] OR ("Spondylarthritis"[All Fields] AND "ankylopoietica"[All Fields]) OR "spondylarthritis ankylopoietica"[All Fields]) OR ("spondylitis, ankylosing"[MeSH Terms] OR ("spondylitis"[All Fields] AND "ankylosing"[All Fields]) OR "ankylosing spondylitis"[All Fields] OR ("spondyloarthritis"[All Fields] AND "ankylopoietica"[All Fields]) OR "spondyloarthritis ankylopoietica"[All Fields]) OR ("spondylitis, ankylosing"[MeSH Terms] OR ("spondylitis"[All Fields] AND "ankylosing"[All Fields]) OR "ankylosing spondylitis"[All Fields] OR ("ankylosing"[All Fields] AND "spondylitis"[All Fields])) OR ("spondylitis, ankylosing"[MeSH Terms] OR ("spondylitis"[All Fields] AND "ankylosing"[All Fields]) OR "ankylosing spondylitis"[All Fields] OR ("ankylosing"[All Fields] AND "Spondylarthritis"[All Fields]) OR "ankylosing spondylarthritis"[All Fields]) OR ("spondylitis, ankylosing"[MeSH Terms] OR ("spondylitis"[All Fields] AND "ankylosing"[All Fields]) OR "ankylosing spondylitis"[All Fields] OR ("ankylosing"[All Fields] AND "spondylarthritides"[All Fields]) OR "ankylosing spondylarthritides"[All Fields]) OR ("spondylitis, ankylosing"[MeSH Terms] OR ("spondylitis"[All Fields] AND "ankylosing"[All Fields]) OR "ankylosing spondylitis"[All Fields] OR ("ankylosing"[All Fields] AND "spondyloarthritis"[All Fields]) OR "ankylosing spondyloarthritis"[All Fields]) OR ("spondylitis, ankylosing"[MeSH Terms] OR ("spondylitis"[All Fields] AND "ankylosing"[All Fields]) OR "ankylosing spondylitis"[All Fields] OR ("spondylitis"[All Fields] AND "ankylopoietica"[All Fields]) OR "spondylitis ankylopoietica"[All Fields]) OR ("spondylitis, ankylosing"[MeSH Terms] OR ("spondylitis"[All Fields] AND "ankylosing"[All Fields]) OR "ankylosing spondylitis"[All Fields] OR ("bechterew"[All Fields] AND "disease"[All Fields]) OR "bechterew disease"[All Fields]) OR ("spondylitis, ankylosing"[MeSH Terms] OR ("spondylitis"[All Fields] AND "ankylosing"[All Fields]) OR "ankylosing spondylitis"[All Fields] OR ("rheumatoid"[All Fields] AND "spondylitis"[All Fields]) OR "rheumatoid spondylitis"[All Fields]) OR "lupus erythematosus, systemic"[MeSH Terms] OR ("lupus erythematosus, systemic"[MeSH Terms] OR ("lupus"[All Fields] AND "erythematosus"[All Fields] AND "systemic"[All Fields]) OR "systemic lupus erythematosus"[All Fields] OR ("lupus"[All Fields] AND "erythematosus"[All Fields] AND "systemic"[All Fields]) OR "lupus erythematosus systemic"[All Fields]) OR ("lupus erythematosus, systemic"[MeSH Terms] OR ("lupus"[All Fields] AND "erythematosus"[All Fields] AND "systemic"[All Fields]) OR "systemic lupus erythematosus"[All Fields] OR ("systemic"[All Fields] AND "lupus"[All Fields] AND "erythematosus"[All Fields])) OR ("lupus erythematosus, systemic"[MeSH Terms] OR ("lupus"[All Fields] AND "erythematosus"[All Fields] AND "systemic"[All Fields]) OR "systemic lupus erythematosus"[All Fields] OR ("lupus"[All Fields] AND "erythematosus"[All Fields] AND "disseminatus"[All Fields]) OR "lupus erythematosus disseminatus"[All Fields]) OR ("lupus erythematosus, systemic"[MeSH Terms] OR ("lupus"[All Fields] AND "erythematosus"[All Fields] AND "systemic"[All Fields]) OR "systemic lupus erythematosus"[All Fields] OR ("libman"[All Fields] AND "sacks"[All Fields] AND "disease"[All Fields]) OR "libman sacks disease"[All Fields]) OR "Myositis"[MeSH Terms] OR ("Myositis"[MeSH Terms] OR "Myositis"[All Fields] OR "myositides"[All Fields]) OR ("Myositis"[MeSH Terms] OR "Myositis"[All Fields] OR "myositides"[All Fields]) OR ("Myositis"[MeSH Terms] OR "Myositis"[All Fields] OR ("inflammatory"[All Fields] AND "muscle"[All Fields] AND "diseases"[All Fields]) OR "inflammatory muscle diseases"[All Fields]) OR ("Myositis"[MeSH Terms] OR "Myositis"[All Fields] OR ("inflammatory"[All Fields] AND "muscle"[All Fields] AND "disease"[All Fields]) OR "inflammatory muscle disease"[All Fields]) OR ("Myositis"[MeSH Terms] OR "Myositis"[All Fields] OR ("inflammatory"[All Fields] AND "myopathy"[All Fields]) OR "inflammatory myopathy"[All Fields]) OR ("Myositis"[MeSH Terms] OR "Myositis"[All Fields] OR ("inflammatory"[All Fields] AND "myopathies"[All Fields]) OR "inflammatory myopathies"[All Fields]) OR ("Myositis"[MeSH Terms] OR "Myositis"[All Fields] OR ("proliferative"[All Fields] AND "Myositis"[All Fields]) OR "proliferative myositis"[All Fields]) OR ("Myositis"[MeSH Terms] OR "Myositis"[All Fields] OR ("infectious"[All Fields] AND "myositides"[All Fields]) OR "infectious myositides"[All Fields]) OR ("Myositis"[MeSH Terms] OR "Myositis"[All Fields] OR ("infectious"[All Fields] AND "Myositis"[All Fields]) OR "infectious myositis"[All Fields]) OR ("Myositis"[MeSH Terms] OR "Myositis"[All Fields] OR ("idiopathic"[All Fields] AND "inflammatory"[All Fields] AND "myopathies"[All Fields]) OR "idiopathic inflammatory myopathies"[All Fields]) OR ("Myositis"[MeSH Terms] OR "Myositis"[All Fields] OR ("idiopathic"[All Fields] AND "inflammatory"[All Fields] AND "myopathy"[All Fields]) OR "idiopathic inflammatory myopathy"[All Fields]) OR ("Myositis"[MeSH Terms] OR "Myositis"[All Fields] OR ("idiopathic"[All Fields] AND "inflammatory"[All Fields] AND "Myositis"[All Fields]) OR "idiopathic inflammatory myositis"[All Fields]) OR ("Myositis"[MeSH Terms] OR "Myositis"[All Fields] OR ("focal"[All Fields] AND "Myositis"[All Fields]) OR "focal myositis"[All Fields]) OR "Systemic Vasculitis"[MeSH Terms] OR ("Systemic Vasculitis"[MeSH Terms] OR ("systemic"[All Fields] AND "vasculitis"[All Fields]) OR "Systemic Vasculitis"[All Fields]) OR ("Systemic Vasculitis"[MeSH Terms] OR ("systemic"[All Fields] AND "vasculitis"[All Fields]) OR "Systemic Vasculitis"[All Fields] OR ("systemic"[All Fields] AND "vasculitides"[All Fields]) OR "systemic vasculitides"[All Fields]) OR "Sjogren's Syndrome"[MeSH Terms] OR ("Sjogren's Syndrome"[MeSH Terms] OR ("sjogren s"[All Fields] AND "syndrome"[All Fields]) OR "Sjogren's Syndrome"[All Fields]) OR ("Sjogren's Syndrome"[MeSH Terms] OR ("sjogren s"[All Fields] AND "syndrome"[All Fields]) OR "Sjogren's Syndrome"[All Fields] OR ("sjogrens"[All Fields] AND "syndrome"[All Fields]) OR "sjogrens syndrome"[All Fields]) OR ("Sjogren's Syndrome"[MeSH Terms] OR ("sjogren s"[All Fields] AND "syndrome"[All Fields]) OR "Sjogren's Syndrome"[All Fields] OR ("sjogren"[All Fields] AND "syndrome"[All Fields]) OR "sjogren syndrome"[All Fields]) OR ("Sjogren's Syndrome"[MeSH Terms] OR ("sjogren s"[All Fields] AND "syndrome"[All Fields]) OR "Sjogren's Syndrome"[All Fields] OR ("sicca"[All Fields] AND "syndrome"[All Fields]) OR "sicca syndrome"[All Fields]) OR "scleroderma, systemic"[MeSH Terms] OR ("scleroderma, systemic"[MeSH Terms] OR ("scleroderma"[All Fields] AND "systemic"[All Fields]) OR "systemic scleroderma"[All Fields] OR ("scleroderma"[All Fields] AND "systemic"[All Fields]) OR "scleroderma systemic"[All Fields]) OR ("scleroderma, systemic"[MeSH Terms] OR ("scleroderma"[All Fields] AND "systemic"[All Fields]) OR "systemic scleroderma"[All Fields] OR ("systemic"[All Fields] AND "sclerosis"[All Fields]) OR "systemic sclerosis"[All Fields]) OR ("scleroderma, systemic"[MeSH Terms] OR ("scleroderma"[All Fields] AND "systemic"[All Fields]) OR "systemic scleroderma"[All Fields] OR ("systemic"[All Fields] AND "scleroderma"[All Fields])) OR "Mixed Connective Tissue Disease"[MeSH Terms] OR ("Mixed Connective Tissue Disease"[MeSH Terms] OR ("mixed"[All Fields] AND "connective"[All Fields] AND "tissue"[All Fields] AND "disease"[All Fields]) OR "Mixed Connective Tissue Disease"[All Fields]) OR ("Mixed Connective Tissue Disease"[MeSH Terms] OR ("mixed"[All Fields] AND "connective"[All Fields] AND "tissue"[All Fields] AND "disease"[All Fields]) OR "Mixed Connective Tissue Disease"[All Fields] OR ("sharp"[All Fields] AND "syndrome"[All Fields]) OR "sharp syndrome"[All Fields]) OR ("Mixed Connective Tissue Disease"[MeSH Terms] OR ("mixed"[All Fields] AND "connective"[All Fields] AND "tissue"[All Fields] AND "disease"[All Fields]) OR "Mixed Connective Tissue Disease"[All Fields] OR "mctd"[All Fields])) AND ("Rituximab"[MeSH Terms] OR ("Rituximab"[MeSH Terms] OR "Rituximab"[All Fields] OR "rituximab s"[All Fields]) OR ("Rituximab"[MeSH Terms] OR "Rituximab"[All Fields] OR ("Rituximab"[All Fields] AND "cd20"[All Fields] AND "antibody"[All Fields]) OR "rituximab cd20 antibody"[All Fields]) OR ("Rituximab"[MeSH Terms] OR "Rituximab"[All Fields] OR "mabthera"[All Fields]) OR ("Rituximab"[MeSH Terms] OR "Rituximab"[All Fields] OR ("idec"[All Fields] AND "c2b8"[All Fields] AND "antibody"[All Fields]) OR "idec c2b8 antibody"[All Fields]) OR ("Rituximab"[MeSH Terms] OR "Rituximab"[All Fields] OR ("idec"[All Fields] AND "c2b8"[All Fields]) OR "idec c2b8"[All Fields]) OR ("Rituximab"[MeSH Terms] OR "Rituximab"[All Fields] OR "gp2013"[All Fields]) OR ("Rituximab"[MeSH Terms] OR "Rituximab"[All Fields] OR "rituxan"[All Fields] OR "rituximab s"[All Fields])) AND ("vaccines, inactivated"[MeSH Terms] OR ("vaccines, inactivated"[MeSH Terms] OR ("vaccines"[All Fields] AND "inactivated"[All Fields]) OR "inactivated vaccines"[All Fields] OR ("vaccines"[All Fields] AND "inactivated"[All Fields]) OR "vaccines inactivated"[All Fields]) OR ("vaccines, inactivated"[MeSH Terms] OR ("vaccines"[All Fields] AND "inactivated"[All Fields]) OR "inactivated vaccines"[All Fields] OR ("inactivated"[All Fields] AND "vaccines"[All Fields])) OR ("vaccines, inactivated"[MeSH Terms] OR ("vaccines"[All Fields] AND "inactivated"[All Fields]) OR "inactivated vaccines"[All Fields] OR ("killed"[All Fields] AND "vaccine"[All Fields]) OR "killed vaccine"[All Fields]) OR ("vaccines, inactivated"[MeSH Terms] OR ("vaccines"[All Fields] AND "inactivated"[All Fields]) OR "inactivated vaccines"[All Fields] OR ("killed"[All Fields] AND "vaccines"[All Fields]) OR "killed vaccines"[All Fields]) OR ("vaccines, inactivated"[MeSH Terms] OR ("vaccines"[All Fields] AND "inactivated"[All Fields]) OR "inactivated vaccines"[All Fields] OR ("inactivated"[All Fields] AND "vaccine"[All Fields]) OR "inactivated vaccine"[All Fields]) OR ("influenza vaccines"[MeSH Terms] OR ("influenza"[All Fields] AND "vaccines"[All Fields]) OR "influenza vaccines"[All Fields]) OR ("influenza vaccines"[MeSH Terms] OR ("influenza"[All Fields] AND "vaccines"[All Fields]) OR "influenza vaccines"[All Fields] OR ("influenza"[All Fields] AND "vaccine"[All Fields]) OR "influenza vaccine"[All Fields]) OR ("influenza vaccines"[MeSH Terms] OR ("influenza"[All Fields] AND "vaccines"[All Fields]) OR "influenza vaccines"[All Fields] OR ("influenzavirus"[All Fields] AND "vaccines"[All Fields])) OR ("influenza vaccines"[MeSH Terms] OR ("influenza"[All Fields] AND "vaccines"[All Fields]) OR "influenza vaccines"[All Fields] OR ("influenza"[All Fields] AND "virus"[All Fields] AND "vaccines"[All Fields]) OR "influenza virus vaccines"[All Fields]) OR ("influenza vaccines"[MeSH Terms] OR ("influenza"[All Fields] AND "vaccines"[All Fields]) OR "influenza vaccines"[All Fields] OR ("influenzavirus"[All Fields] AND "vaccine"[All Fields]) OR "influenzavirus vaccine"[All Fields]) OR ("influenza vaccines"[MeSH Terms] OR ("influenza"[All Fields] AND "vaccines"[All Fields]) OR "influenza vaccines"[All Fields] OR ("influenza"[All Fields] AND "virus"[All Fields] AND "vaccine"[All Fields]) OR "influenza virus vaccine"[All Fields]) OR ("influenza vaccines"[MeSH Terms] OR ("influenza"[All Fields] AND "vaccines"[All Fields]) OR "influenza vaccines"[All Fields] OR ("high"[All Fields] AND "dose"[All Fields] AND "trivalent"[All Fields] AND "influenza"[All Fields] AND "vaccine"[All Fields]) OR "high dose trivalent influenza vaccine"[All Fields]) OR ("influenza vaccines"[MeSH Terms] OR ("influenza"[All Fields] AND "vaccines"[All Fields]) OR "influenza vaccines"[All Fields] OR ("high"[All Fields] AND "dose"[All Fields] AND "trivalent"[All Fields] AND "influenza"[All Fields] AND "vaccine"[All Fields]) OR "high dose trivalent influenza vaccine"[All Fields]) OR ("influenza vaccines"[MeSH Terms] OR ("influenza"[All Fields] AND "vaccines"[All Fields]) OR "influenza vaccines"[All Fields] OR ("trivalent"[All Fields] AND "influenza"[All Fields] AND "vaccine"[All Fields]) OR "trivalent influenza vaccine"[All Fields]) OR ("influenza vaccines"[MeSH Terms] OR ("influenza"[All Fields] AND "vaccines"[All Fields]) OR "influenza vaccines"[All Fields] OR ("quadrivalent"[All Fields] AND "influenza"[All Fields] AND "vaccine"[All Fields]) OR "quadrivalent influenza vaccine"[All Fields]) OR "Pneumococcal Vaccines"[MeSH Terms] OR ("Pneumococcal Vaccines"[MeSH Terms] OR ("pneumococcal"[All Fields] AND "vaccines"[All Fields]) OR "Pneumococcal Vaccines"[All Fields]) OR ("Pneumococcal Vaccines"[MeSH Terms] OR ("pneumococcal"[All Fields] AND "vaccines"[All Fields]) OR "Pneumococcal Vaccines"[All Fields] OR ("pneumococcal"[All Fields] AND "vaccine"[All Fields]) OR "pneumococcal vaccine"[All Fields]) OR ("Pneumococcal Vaccines"[MeSH Terms] OR ("pneumococcal"[All Fields] AND "vaccines"[All Fields]) OR "Pneumococcal Vaccines"[All Fields] OR "pneumovax"[All Fields]) OR ("Pneumococcal Vaccines"[MeSH Terms] OR ("pneumococcal"[All Fields] AND "vaccines"[All Fields]) OR "Pneumococcal Vaccines"[All Fields] OR ("pneumococcal"[All Fields] AND "polysaccharide"[All Fields] AND "vaccine"[All Fields]) OR "pneumococcal polysaccharide vaccine"[All Fields]) OR ("Pneumococcal Vaccines"[MeSH Terms] OR ("pneumococcal"[All Fields] AND "vaccines"[All Fields]) OR "Pneumococcal Vaccines"[All Fields] OR ("pnu"[All Fields] AND "imune"[All Fields] AND "vaccine"[All Fields]) OR "pnu imune vaccine"[All Fields]) OR ("Pneumococcal Vaccines"[MeSH Terms] OR ("pneumococcal"[All Fields] AND "vaccines"[All Fields]) OR "Pneumococcal Vaccines"[All Fields] OR ("pnuimune"[All Fields] AND "vaccine"[All Fields])) OR (("conjugable"[All Fields] OR "conjugal"[All Fields] OR "conjugally"[All Fields] OR "conjugant"[All Fields] OR "conjugants"[All Fields] OR "conjugate"[All Fields] OR "conjugate s"[All Fields] OR "conjugated"[All Fields] OR "conjugates"[All Fields] OR "conjugating"[All Fields] OR "conjugation"[All Fields] OR "conjugational"[All Fields] OR "conjugations"[All Fields] OR "conjugative"[All Fields] OR "conjugatively"[All Fields]) AND "penumococcal"[All Fields] AND ("vaccin"[Supplementary Concept] OR "vaccin"[All Fields] OR "vaccination"[MeSH Terms] OR "vaccination"[All Fields] OR "vaccinable"[All Fields] OR "vaccinal"[All Fields] OR "vaccinate"[All Fields] OR "vaccinated"[All Fields] OR "vaccinates"[All Fields] OR "vaccinating"[All Fields] OR "vaccinations"[All Fields] OR "vaccination s"[All Fields] OR "vaccinator"[All Fields] OR "vaccinators"[All Fields] OR "vaccine s"[All Fields] OR "vaccined"[All Fields] OR "vaccines"[MeSH Terms] OR "vaccines"[All Fields] OR "vaccine"[All Fields] OR "vaccins"[All Fields])) OR ("herpes zoster vaccine"[MeSH Terms] OR ("herpes"[All Fields] AND "zoster"[All Fields] AND "vaccine"[All Fields]) OR "herpes zoster vaccine"[All Fields]) OR ("herpes zoster vaccine"[MeSH Terms] OR ("herpes"[All Fields] AND "zoster"[All Fields] AND "vaccine"[All Fields]) OR "herpes zoster vaccine"[All Fields] OR ("zoster"[All Fields] AND "vaccine"[All Fields]) OR "zoster vaccine"[All Fields]) OR ("herpes zoster vaccine"[MeSH Terms] OR ("herpes"[All Fields] AND "zoster"[All Fields] AND "vaccine"[All Fields]) OR "herpes zoster vaccine"[All Fields] OR ("shingles"[All Fields] AND "vaccine"[All Fields]) OR "shingles vaccine"[All Fields]) OR "Hepatitis B Vaccines"[MeSH Terms] OR ("Hepatitis B Vaccines"[MeSH Terms] OR "Hepatitis B Vaccines"[All Fields]) OR ("Hepatitis B Vaccines"[MeSH Terms] OR "Hepatitis B Vaccines"[All Fields] OR "hepatitis b vaccine"[All Fields]) OR "Papillomavirus Vaccines"[MeSH Terms] OR ("Papillomavirus Vaccines"[MeSH Terms] OR ("papillomavirus"[All Fields] AND "vaccines"[All Fields]) OR "Papillomavirus Vaccines"[All Fields]) OR ("Papillomavirus Vaccines"[MeSH Terms] OR ("papillomavirus"[All Fields] AND "vaccines"[All Fields]) OR "Papillomavirus Vaccines"[All Fields] OR ("papillomavirus"[All Fields] AND "vaccine"[All Fields]) OR "papillomavirus vaccine"[All Fields]) OR ("Papillomavirus Vaccines"[MeSH Terms] OR ("papillomavirus"[All Fields] AND "vaccines"[All Fields]) OR "Papillomavirus Vaccines"[All Fields] OR ("human"[All Fields] AND "papillomavirus"[All Fields] AND "vaccines"[All Fields]) OR "human papillomavirus vaccines"[All Fields]) OR ("Papillomavirus Vaccines"[MeSH Terms] OR ("papillomavirus"[All Fields] AND "vaccines"[All Fields]) OR "Papillomavirus Vaccines"[All Fields] OR ("hpv"[All Fields] AND "vaccine"[All Fields]) OR "hpv vaccine"[All Fields]) OR ("Papillomavirus Vaccines"[MeSH Terms] OR ("papillomavirus"[All Fields] AND "vaccines"[All Fields]) OR "Papillomavirus Vaccines"[All Fields] OR ("human"[All Fields] AND "papilloma"[All Fields] AND "virus"[All Fields] AND "vaccine"[All Fields]) OR "human papilloma virus vaccine"[All Fields]) OR ("Papillomavirus Vaccines"[MeSH Terms] OR ("papillomavirus"[All Fields] AND "vaccines"[All Fields]) OR "Papillomavirus Vaccines"[All Fields] OR ("hpv"[All Fields] AND "vaccines"[All Fields]) OR "hpv vaccines"[All Fields]) OR ("Papillomavirus Vaccines"[MeSH Terms] OR ("papillomavirus"[All Fields] AND "vaccines"[All Fields]) OR "Papillomavirus Vaccines"[All Fields] OR ("human"[All Fields] AND "papilloma"[All Fields] AND "virus"[All Fields] AND "vaccines"[All Fields]) OR "human papilloma virus vaccines"[All Fields]) OR ("Papillomavirus Vaccines"[MeSH Terms] OR ("papillomavirus"[All Fields] AND "vaccines"[All Fields]) OR "Papillomavirus Vaccines"[All Fields] OR ("human"[All Fields] AND "papillomavirus"[All Fields] AND "vaccine"[All Fields]) OR "human papillomavirus vaccine"[All Fields]) OR "Poliovirus Vaccines"[MeSH Terms] OR ("Poliovirus Vaccines"[MeSH Terms] OR ("poliovirus"[All Fields] AND "vaccines"[All Fields]) OR "Poliovirus Vaccines"[All Fields]) OR ("Poliovirus Vaccines"[MeSH Terms] OR ("poliovirus"[All Fields] AND "vaccines"[All Fields]) OR "Poliovirus Vaccines"[All Fields] OR ("poliovirus"[All Fields] AND "vaccine"[All Fields]) OR "poliovirus vaccine"[All Fields]) OR "salk"[All Fields] OR "Hepatitis A Vaccines"[MeSH Terms] OR ("Hepatitis A Vaccines"[MeSH Terms] OR "Hepatitis A Vaccines"[All Fields]) OR ("Hepatitis A Vaccines"[MeSH Terms] OR "Hepatitis A Vaccines"[All Fields] OR ("hepatovirus"[All Fields] AND "vaccines"[All Fields])) OR ("Hepatitis A Vaccines"[MeSH Terms] OR "Hepatitis A Vaccines"[All Fields] OR "hepatitis a vaccine"[All Fields]) OR ("hepatitis a vaccine inactivated"[All Fields] OR "Hepatitis A Vaccines"[MeSH Terms] OR "Hepatitis A Vaccines"[All Fields] OR "vaqta"[All Fields]) OR ("Hepatitis A Vaccines"[MeSH Terms] OR "Hepatitis A Vaccines"[All Fields] OR "havrix"[All Fields]) OR "Meningococcal Vaccines"[MeSH Terms] OR ("Meningococcal Vaccines"[MeSH Terms] OR ("meningococcal"[All Fields] AND "vaccines"[All Fields]) OR "Meningococcal Vaccines"[All Fields] OR ("meningococcal"[All Fields] AND "vaccine"[All Fields]) OR "meningococcal vaccine"[All Fields]) OR ("Meningococcal Vaccines"[MeSH Terms] OR ("meningococcal"[All Fields] AND "vaccines"[All Fields]) OR "Meningococcal Vaccines"[All Fields] OR "menactra"[All Fields]) OR ("Meningococcal Vaccines"[MeSH Terms] OR ("meningococcal"[All Fields] AND "vaccines"[All Fields]) OR "Meningococcal Vaccines"[All Fields] OR ("tetravalent"[All Fields] AND "meningococcal"[All Fields] AND "vaccine"[All Fields]) OR "tetravalent meningococcal vaccine"[All Fields]) OR ("Meningococcal Vaccines"[MeSH Terms] OR ("meningococcal"[All Fields] AND "vaccines"[All Fields]) OR "Meningococcal Vaccines"[All Fields] OR "menomune"[All Fields]) OR ("Meningococcal Vaccines"[MeSH Terms] OR ("meningococcal"[All Fields] AND "vaccines"[All Fields]) OR "Meningococcal Vaccines"[All Fields] OR "menveo"[All Fields]) OR ("Meningococcal Vaccines"[MeSH Terms] OR ("meningococcal"[All Fields] AND "vaccines"[All Fields]) OR "Meningococcal Vaccines"[All Fields] OR ("bivalent"[All Fields] AND "meningococcal"[All Fields] AND "vaccine"[All Fields]) OR "bivalent meningococcal vaccine"[All Fields]) OR ("Meningococcal Vaccines"[MeSH Terms] OR ("meningococcal"[All Fields] AND "vaccines"[All Fields]) OR "Meningococcal Vaccines"[All Fields] OR ("meningococcal"[All Fields] AND "polysaccharide"[All Fields] AND "vaccine"[All Fields]) OR "meningococcal polysaccharide vaccine"[All Fields]) OR "Diphtheria-Tetanus-Pertussis Vaccine"[MeSH Terms] OR ("Diphtheria-Tetanus-Pertussis Vaccine"[MeSH Terms] OR ("diphtheria tetanus pertussis"[All Fields] AND "vaccine"[All Fields]) OR "Diphtheria-Tetanus-Pertussis Vaccine"[All Fields] OR ("diphtheria"[All Fields] AND "tetanus"[All Fields] AND "pertussis"[All Fields] AND "vaccine"[All Fields]) OR "Diphtheria-Tetanus-Pertussis Vaccine"[All Fields]) OR ("Diphtheria-Tetanus-Pertussis Vaccine"[MeSH Terms] OR ("diphtheria tetanus pertussis"[All Fields] AND "vaccine"[All Fields]) OR "Diphtheria-Tetanus-Pertussis Vaccine"[All Fields] OR ("diphtheria"[All Fields] AND "tetanus"[All Fields] AND "pertussis"[All Fields] AND "vaccine"[All Fields]) OR "Diphtheria-Tetanus-Pertussis Vaccine"[All Fields]) OR ("Diphtheria-Tetanus-Pertussis Vaccine"[MeSH Terms] OR ("diphtheria tetanus pertussis"[All Fields] AND "vaccine"[All Fields]) OR "Diphtheria-Tetanus-Pertussis Vaccine"[All Fields] OR ("dtwp"[All Fields] AND "vaccine"[All Fields]) OR "dtwp vaccine"[All Fields]) OR ("Diphtheria-Tetanus-Pertussis Vaccine"[MeSH Terms] OR ("diphtheria tetanus pertussis"[All Fields] AND "vaccine"[All Fields]) OR "Diphtheria-Tetanus-Pertussis Vaccine"[All Fields] OR ("dpt"[All Fields] AND "vaccine"[All Fields]) OR "dpt vaccine"[All Fields]) OR ("Diphtheria-Tetanus-Pertussis Vaccine"[MeSH Terms] OR ("diphtheria tetanus pertussis"[All Fields] AND "vaccine"[All Fields]) OR "Diphtheria-Tetanus-Pertussis Vaccine"[All Fields] OR ("dtp"[All Fields] AND "vaccine"[All Fields]) OR "dtp vaccine"[All Fields]) OR ("Diphtheria-Tetanus-Pertussis Vaccine"[MeSH Terms] OR ("diphtheria tetanus pertussis"[All Fields] AND "vaccine"[All Fields]) OR "Diphtheria-Tetanus-Pertussis Vaccine"[All Fields] OR ("di"[All Fields] AND "te"[All Fields] AND "per"[All Fields] AND "vaccine"[All Fields]) OR "di te per vaccine"[All Fields]) OR ("Diphtheria-Tetanus-Pertussis Vaccine"[MeSH Terms] OR ("diphtheria tetanus pertussis"[All Fields] AND "vaccine"[All Fields]) OR "Diphtheria-Tetanus-Pertussis Vaccine"[All Fields] OR ("diphtheria"[All Fields] AND "pertussis"[All Fields] AND "tetanus"[All Fields] AND "vaccine"[All Fields]) OR "diphtheria pertussis tetanus vaccine"[All Fields]) OR ("haemophilus influenza type b polysaccharide vaccine tetanus toxin conjugate"[Supplementary Concept] OR "haemophilus influenza type b polysaccharide vaccine tetanus toxin conjugate"[All Fields] OR "haemophilus influenza type b polysaccharide vaccine tetanus toxin conjugate"[All Fields]) OR ("haemophilus influenza type b polysaccharide vaccine tetanus toxin conjugate"[Supplementary Concept] OR "haemophilus influenza type b polysaccharide vaccine tetanus toxin conjugate"[All Fields] OR "prp t"[All Fields]) OR ("haemophilus influenza type b polysaccharide vaccine tetanus toxin conjugate"[Supplementary Concept] OR "haemophilus influenza type b polysaccharide vaccine tetanus toxin conjugate"[All Fields] OR "hib tt"[All Fields]) OR ("haemophilus influenza type b polysaccharide vaccine tetanus toxin conjugate"[Supplementary Concept] OR "haemophilus influenza type b polysaccharide vaccine tetanus toxin conjugate"[All Fields] OR "omnihib"[All Fields]) OR ("haemophilus influenza type b polysaccharide vaccine tetanus toxin conjugate"[Supplementary Concept] OR "haemophilus influenza type b polysaccharide vaccine tetanus toxin conjugate"[All Fields] OR "acthib"[All Fields]))

61 results

1.2 Following there are search strategy in Embase

**No.**

**Query**

**Results**

**474**

**#27**

**#25 AND #26**

**113,450**

**#26**

**'rituximab'/syn**

**3,124**

**#25**

**#12 AND #24**

**149,994**

**#24**

**#13 OR #14 OR #15 OR #16 OR #17 OR #18 OR #19 OR #20 OR #21 OR #22 OR #23**

**36**

**#23**

**'haemophilus influenza type b polysaccharide vaccine tetanus toxin conjugate'/syn**

**22,584**

**#22**

**'diphtheria pertussis tetanus vaccine'/syn**

**8,774**

**#21**

**'meningococcal vaccines'/syn**

**5,982**

**#20**

**'hepatitis a vaccines'/syn**

**15,713**

**#19**

**'poliovirus vaccines'/syn**

**18,091**

**#18**

**'human papilloma virus vaccine'/syn**

**25,125**

**#17**

**'hepatitis b vaccine'/syn**

**4,441**

**#16**

**'herpes zoster vaccine'/syn**

**25,896**

**#15**

**'pneumococcus vaccine'/syn**

**48,734**

**#14**

**'influenza vaccines'/syn**

**10,379**

**#13**

**'inactivated vaccine'/syn**

**582,415**

**#12**

**#1 OR #2 OR #3 OR #4 OR #5 OR #6 OR #7 OR #8 OR #9 OR #10 OR #11**

**5,506**

**#11**

**'mixed connective tissue disease'/syn**

**43,158**

**#10**

**'systemic sclerosis'/syn**

**35,919**

**#9**

**'sjoegren syndrome'/syn**

**9,231**

**#8**

**'systemic vasculitis'/syn**

**52,684**

**#7**

**'myositis'/syn**

**141,094**

**#6**

**'systemic lupus erythematosus'/syn**

**40,970**

**#5**

**'ankylosing spondylitis'/syn**

**33,640**

**#4**

**'psoriatic arthritis'/syn**

**73,128**

**#3**

**'spondylarthritis'/syn**

**282,694**

**#2**

**'rheumatoid arthritis'/syn**

**56,077**

**#1**

**'rheumatic disease'/de**

1.3 Following there are search strategy in Cochrane Library

**ID	Search	Hits**

**#1	MeSH descriptor: [Rheumatic Diseases] explode all trees	20731**

**#2	MeSH descriptor: [Arthritis, Rheumatoid] explode all trees	7302**

**#3	MeSH descriptor: [Spondylarthritis] explode all trees	1823**

**#4	MeSH descriptor: [Arthritis, Psoriatic] explode all trees	634**

**#5	MeSH descriptor: [Spondylitis, Ankylosing] explode all trees	826**

**#6	MeSH descriptor: [Lupus Erythematosus, Systemic] explode all trees	1425**

**#7	MeSH descriptor: [Myositis] explode all trees	318**

**#8	MeSH descriptor: [Systemic Vasculitis] explode all trees	266**

**#9	MeSH descriptor: [Sjogren's Syndrome] explode all trees	365**

**#10	MeSH descriptor: [Scleroderma, Systemic] explode all trees	717**

**#11	MeSH descriptor: [Mixed Connective Tissue Disease] explode all trees	13**

**#12	#1 OR #2 OR #3 OR #4 OR #5 OR #6 OR #7 OR #8 OR #9 OR #10 OR #11	24888**

**#13	MeSH descriptor: [Vaccines, Inactivated] explode all trees	1148**

**#14	MeSH descriptor: [Influenza Vaccines] explode all trees	1853**

**#15	MeSH descriptor: [Pneumococcal Vaccines] explode all trees	1178**

**#16	MeSH descriptor: [Herpes Zoster Vaccine] explode all trees	110**

**#17	MeSH descriptor: [Hepatitis B Vaccines] explode all trees	1003**

**#18	MeSH descriptor: [Papillomavirus Vaccines] explode all trees	549**

**#19	MeSH descriptor: [Poliovirus Vaccines] explode all trees	544**

**#20	MeSH descriptor: [Hepatitis A Vaccines] explode all trees	258**

**#21	MeSH descriptor: [Meningococcal Vaccines] explode all trees	438**

**#22	MeSH descriptor: [Diphtheria-Tetanus-Pertussis Vaccine] explode all trees	585**

**#23	#13 OR #14 OR #15 OR #16 OR #17 OR #18 OR #19 OR #20 OR #21 OR #22	5937**

**#24	MeSH descriptor: [Rituximab] explode all trees	1708**

**#25	#12 AND #23 AND #24	3**

**QUESTION 7. What is the effect of corticosteroid use on the response to inactivated vaccines in patients with immune-mediated rheumatic diseases?**

**Table S1.** Search strategy by database.

| **Database** | **Group** | **Terms** |
| --- | --- | --- |
| PubMed/MEDLINE | Population | "Rheumatic Diseases" [Mesh:NoExp] OR "Rheumatic Disease" OR "Rheumatism" OR "Immune-mediated rheumatic diseases" OR "Arthritis, Rheumatoid" [Mesh:NoExp] OR "Rheumatoid Arthritis" OR "Rheumatoid Nodule" OR "Rheumatoid Vasculitis" OR "Sjogren's Syndrome" OR "Spondylarthritis" [Mesh] OR "Spondylarthritis" OR "Spondylarthritides" OR "Spinal Arthritis" OR "Spinal Arthritides" OR "Arthritis, Psoriatic" [Mesh] OR "Arthritis, Psoriatic" OR "Arthritic Psoriasis" OR "Psoriatic Arthritis" OR "Psoriasis Arthropathica" OR "Psoriatic Arthropathy" OR "Psoriatic Arthropathies" OR "Spondylitis, Ankylosing" [Mesh] OR "Spondylitis, Ankylosing" OR "Bechterew's Disease" OR "Bechterews Disease" OR "Marie Struempell Disease" OR "Spondylarthritis Ankylopoietica" OR "Spondyloarthritis Ankylopoietica" OR "Ankylosing Spondylitis" OR "Ankylosing Spondylarthritis" OR "Ankylosing Spondylarthritides" OR "Ankylosing Spondyloarthritis" OR "Ankylosing Spondyloarthritides" OR "Spondylitis Ankylopoietica" OR "Bechterew Disease" OR "Rheumatoid Spondylitis" OR "Lupus Erythematosus, Systemic" [Mesh] OR "Lupus Erythematosus, Systemic" OR "Systemic Lupus Erythematosus " OR "Lupus Erythematosus Disseminatus" OR "Libman Sacks Disease" OR "Myositis" [Mesh] OR "Myositis" OR "Myositides" OR "Inflammatory Muscle Diseases" OR "Inflammatory Muscle Disease" OR "Inflammatory Myopathy" OR "Inflammatory Myopathies" OR "Proliferative Myositides" OR "Proliferative Myositis" OR "Infectious Myositides" OR "Infectious Myositis" OR "Idiopathic Inflammatory Myopathies" OR "Idiopathic Inflammatory Myopathy" OR "Idiopathic Inflammatory Myositis" OR "Focal Myositides" OR "Focal Myositis" OR "Systemic Vasculitis" [Mesh] OR "Systemic Vasculitis" OR "Systemic Vasculitides" OR "Sjogren's Syndrome" [Mesh] OR "Sjogren's Syndrome" OR "Sjogrens Syndrome" OR "Sjogren Syndrome" OR "Sicca Syndrome" OR "Scleroderma, Systemic" [Mesh] OR "Scleroderma, Systemic" OR "Systemic Sclerosis" OR "Systemic Scleroderma" OR "Mixed Connective Tissue Disease" [Mesh] OR "Mixed Connective Tissue Disease" OR "Sharp Syndrome" OR "MCTD" |
|  | Drugs | "Prednisolone" [Mesh] OR "Prednisolone" OR " Predate" OR "Predonine" OR "Di Adreson F" OR "Methylprednisolone" [Mesh] OR "Metipred" OR "6 Methylprednisolone" OR "Urbason" OR "Medrol" OR "Glucocorticoids" [Mesh] OR "Glucocorticoids" OR "Glucocorticoid" OR "Glucocorticoid Effect" OR "Glucorticoid Effects" OR "Dexamethasone" [Mesh] OR "Dexamethasone" OR "Methylfluorprednisolone" OR "Hexadecadrol" OR "Decameth" OR "Decaspray" OR "Dexasone" OR "Dexpak" OR"Maxidex" OR "Millicorten" OR "Oradexon" OR "Decaject" OR "Decaject L.A. " OR "Hexadrol" OR "Pregnadienes" [Mesh] OR "Pregnadienes" OR "Prednisone" [Mesh] OR "Prednisone" OR "Dehydrocortisone" OR "delta-Cortisone" OR "Rectodelt" OR "Prednison Hexal" OR "Sterapred" OR "Ultracorten" OR "Winpred" OR "Apo-Prednisone" OR "Cortan" OR "Cortancyl" OR"Panafcort" OR "Cutason" OR "Decortin" OR "Dacortin" OR "Decortisyl" OR "Deltasone" OR "Encortone" OR "Encorton" OR "Enkortolon" OR "Kortancyl" OR "Liquid Pred" OR "Meticorten" OR "Orasone" OR "Panasol" OR "Predni Tablinen" OR "Prednidib" OR "Predniment" OR "Prednison Acsis" OR "Pronisone" OR "Sone" OR "Prednison Galen" |
|  | Vaccines | "Vaccines, Inactivated" [Mesh] OR "Vaccines, Inactivated" OR "Inactivated Vaccines" OR "Killed Vaccine" OR "Killed Vaccines" OR "Inactivated Vaccine" OR "Influenza Vaccines" [Mesh:NoExp] OR Influenza Vaccine" OR "Influenzavirus Vaccines" OR "Influenza Virus Vaccines" OR "Influenzavirus Vaccine" OR "Influenza Virus Vaccine" OR "High-Dose Trivalent Influenza Vaccine" OR "High Dose Trivalent Influenza Vaccine" OR "Trivalent Influenza Vaccine" OR "Quadrivalent Influenza Vaccine" OR "Pneumococcal Vaccines" [Mesh] OR "Pneumococcal Vaccines" OR "Pneumococcal Vaccine" OR "Pneumovax" OR "Pneumococcal Polysaccharide Vaccine" OR "Pnu Imune Vaccine" OR "PnuImune Vaccine" OR "Conjugated penumococcal vaccine" OR "Herpes Zoster Vaccine" [Mesh:NoExp] OR " Zoster Vaccine" OR "Shingles Vaccine" OR "Hepatitis B Vaccines" [Mesh] OR "Hepatitis B Vaccines" OR "Hepatitis B Vaccine" OR "Papillomavirus Vaccines" [Mesh] OR "Papillomavirus Vaccines" OR "Papillomavirus Vaccine" OR "Human Papillomavirus Vaccines" OR "HPV Vaccine" OR "Human Papilloma Virus Vaccine" OR "HPV Vaccines" OR "Human Papilloma Virus Vaccines" OR "Human Papillomavirus Vaccine" OR "Poliovirus Vaccines" [Mesh] OR "Poliovirus Vaccines" OR "Poliovirus Vaccine" OR "salk" OR "Hepatitis A Vaccines" [Mesh] OR "Hepatitis A Vaccines" OR "Hepatovirus Vaccines" OR "Hepatitis A Vaccine" OR "Vaqta" OR "Havrix" OR "Meningococcal Vaccines" [Mesh] OR " Meningococcal Vaccine" OR "Menactra" OR "Tetravalent Meningococcal Vaccine" OR "Menomune OR "Menveo" OR "Bivalent Meningococcal Vaccine" OR "Meningococcal Polysaccharide Vaccine" OR "Diphtheria-Tetanus-Pertussis Vaccine" [Mesh] OR "Diphtheria-Tetanus-Pertussis Vaccine" OR " Diphtheria Tetanus Pertussis Vaccine" OR "DTwP Vaccine" OR "DPT Vaccine" OR "DTP Vaccine" OR "Di Te Per Vaccine" OR "Diphtheria Pertussis Tetanus Vaccine" OR "Haemophilus influenza type b polysaccharide vaccine-tetanus toxin conjugate" OR "HIBest" OR "PRP-T" OR "Hib-TT" OR "Hib capsular polysaccharide-tetanus vaccine" OR "Hib CPS-tetanus vaccine" OR "OmniHIB" OR "ActHIB" |
| Embase | Population | Rheumatic Disease OR Rheumatism OR Rheumatoid Arthritis OR Spondylarthritides OR Spinal Arthritis OR Spinal Arthritides OR Arthritic Psoriasis OR Psoriatic Arthritis OR Psoriasis Arthropathica OR Psoriatic Arthropathy OR Psoriatic Arthropathies OR Bechterew's Disease OR Bechterews Disease OR Marie Struempell Disease OR Spondylarthritis Ankylopoietica OR Spondyloarthritis Ankylopoietica OR Ankylosing Spondylitis OR Ankylosing Spondylarthritis OR Ankylosing Spondylarthritides OR Ankylosing Spondyloarthritis OR Ankylosing Spondyloarthritides OR Spondylitis Ankylopoietica OR Bechterew Disease OR Rheumatoid Spondylitis OR Systemic Lupus Erythematosus OR Lupus Erythematosus Disseminatus OR Libman Sacks Disease OR Myositides OR Inflammatory Muscle Diseases OR Inflammatory Muscle Disease OR Inflammatory Myopathy OR Inflammatory Myopathies OR Proliferative Myositides OR Proliferative Myositis OR Infectious Myositides OR Infectious Myositis OR Idiopathic Inflammatory Myopathies OR Idiopathic Inflammatory Myopathy OR Idiopathic Inflammatory Myositis OR Focal Myositides OR Focal Myositis OR Systemic Vasculitides OR Sjogrens Syndrome OR Sjogren Syndrome OR Sicca Syndrome OR Systemic Sclerosis OR Systemic Scleroderma OR Sharp Syndrome OR MCTD |
|  | Drugs | Predate OR Predonine OR Di Adreson F OR Metipred OR 6 Methylprednisolone OR Urbason OR Medrol OR Glucocorticoid OR Glucocorticoid Effect OR Glucorticoid Effects OR Methylfluorprednisolone OR Hexadecadrol OR Decameth OR Decaspray OR Dexasone OR Dexpak OR Maxidex OR Millicorten OR Oradexon OR Decaject OR Decaject L.A. OR Hexadrol OR Dexamethasone OR Dexamethasone Isonicotinate OR Dehydrocortisone OR delta-Cortisone OR Rectodelt OR Prednison Hexal OR Sterapred OR Ultracorten OR Winpred OR Apo-Prednisone OR Cortan OR Cortancyl OR Panafcort OR Cutason OR Decortin OR Dacortin OR Decortisyl OR Deltasone OR Encortone OR Encorton OR Enkortolon OR Kortancyl OR Liquid Pred OR Meticorten OR Orasone OR Panasol OR Predni Tablinen OR Prednidib OR Predniment OR Prednison Acsis OR Pronisone OR Sone OR Prednison Galen |
|  | Vaccines | Inactivated Vaccines OR Killed Vaccine OR Killed Vaccines OR Inactivated Vaccine OR Influenza Vaccine OR Influenzavirus Vaccines OR Influenza Virus Vaccines OR Influenzavirus Vaccine OR Influenza Virus Vaccine OR High-Dose Trivalent Influenza Vaccine OR High Dose Trivalent Influenza Vaccine OR Trivalent Influenza Vaccine OR Quadrivalent Influenza Vaccine OR High dose OR Pneumococcal Vaccine OR Pneumovax OR Pneumococcal Polysaccharide Vaccine OR Pnu Imune Vaccine OR PnuImune Vaccine OR Conjugated penumococcal vaccine OR Zoster Vaccine OR Shingles Vaccine OR Hepatitis B Vaccine OR Papillomavirus Vaccine Human Papillomavirus Vaccines OR HPV Vaccine OR Human Papilloma Virus Vaccine OR HPV Vaccines OR Human Papilloma Virus Vaccines OR Human Papillomavirus Vaccine OR Poliovirus Vaccine OR salk OR Hepatovirus Vaccines OR Hepatitis A Vaccine OR Vaqta OR Havrix OR Meningococcal Vaccine OR Menactra OR Tetravalent Meningococcal Vaccine OR Menomune OR Menveo OR Bivalent Meningococcal Vaccine OR Meningococcal Polysaccharide Vaccine OR Diphtheria Tetanus Pertussis Vaccine OR DTwP Vaccine OR DPT Vaccine DTP Vaccine OR Di Te Per Vaccine OR Diphtheria Pertussis Tetanus Vaccine OR HIBest OR PRP-T OR Hib-TT OR Hib capsular polysaccharide-tetanus vaccine OR Hib CPS-tetanus vaccine OR OmniHIB OR ActHIB |

**QUESTION 8. What is the impact of JAKis use on the response to inactivated vaccines in patients with immune-mediated rheumatic diseases?**

**1.1 Following there are search strategy in PubMed/MEDLINE**

**P = Patients with immune-mediated rheumatic diseases**

(Rheumatic Diseases) OR (Rheumatic Disease) OR (Rheumatism) OR (Immune-mediated rheumatic diseases) OR (Arthritis, Rheumatoid) OR (Rheumatoid Arthritis) OR (Rheumatoid Nodule) OR (Rheumatoid Vasculitis) OR (Sjogren's Syndrome) OR "Spondylarthritis" [Mesh] OR (Spondylarthritis) OR (Spondylarthritides) OR (Spinal Arthritis) OR "Arthritis, Psoriatic" [Mesh] OR (Arthritis, Psoriatic) OR (Arthritic Psoriasis) OR (Psoriatic Arthritis) OR (Psoriasis Arthropathica) OR (Psoriatic Arthropathy) OR (Psoriatic Arthropathies) OR "Spondylitis, Ankylosing" [Mesh] OR (Spondylitis, Ankylosing) OR (Bechterew's Disease) OR (Bechterews Disease) OR (Marie Struempell Disease) OR (Spondylarthritis Ankylopoietica) OR (Spondyloarthritis Ankylopoietica) OR (Ankylosing Spondylitis) OR (Ankylosing Spondylarthritis) OR (Ankylosing Spondylarthritides) OR (Ankylosing Spondyloarthritis) OR (Spondylitis Ankylopoietica) OR (Bechterew Disease) OR (Rheumatoid Spondylitis) OR "Lupus Erythematosus, Systemic" [Mesh] OR (Lupus Erythematosus, Systemic) OR (Systemic Lupus Erythematosus) OR (Lupus Erythematosus Disseminatus) OR (Libman Sacks Disease) OR "Myositis" [Mesh] OR (Myositis) OR (Myositides) OR (Inflammatory Muscle Diseases) OR (Inflammatory Muscle Disease) OR (Inflammatory Myopathy) OR (Inflammatory Myopathies) OR (Proliferative Myositis) OR (Infectious Myositides) OR (Infectious Myositis) OR (Idiopathic Inflammatory Myopathies) OR (Idiopathic Inflammatory Myopathy) OR (Idiopathic Inflammatory Myositis) OR (Focal Myositis) OR "Systemic Vasculitis" [Mesh] OR (Systemic Vasculitis) OR (Systemic Vasculitides) OR "Sjogren's Syndrome" [Mesh] OR (Sjogren's Syndrome) OR (Sjogrens Syndrome) OR (Sjogren Syndrome) OR (Sicca Syndrome) OR "Scleroderma, Systemic" [Mesh] OR (Scleroderma, Systemic) OR (Systemic Sclerosis) OR (Systemic Scleroderma) OR "Mixed Connective Tissue Disease" [Mesh] OR (Mixed Connective Tissue Disease) OR (Sharp Syndrome) OR (MCTD)

**I = JAKis use**

"Janus Kinase Inhibitors" [Mesh] OR (Janus Kinase Inhibitors) OR (JAK Inhibitors) OR (Janus Kinase Inhibitor) OR (JAK Inhibitor) OR "upadacitinib" [Mesh] OR (upadacitinib) OR (ABT-494) OR (Rinvoq) OR "Protein Kinase Inhibitors" [Mesh] OR (Protein Kinase Inhibitors) OR (Protein Kinase Inhibitor) OR "tofacitinib" [Mesh] OR (tofacitinib) OR (tasocitinib) OR (tofacitinib citrate) OR (Xeljanz) OR (CP 690550) OR "baricitinib" [Mesh] OR (baricitinib) OR (INCB-28050) OR (Olumiant) OR (baricitinib phosphate) OR (baricitinib phosphate salt) OR (INCB028050) OR (LY3009104)

**O = Vaccination with inactivated vaccine**

"Vaccines, Inactivated" [Mesh] OR (Vaccines, Inactivated) OR (Inactivated Vaccines) OR (Killed Vaccine) OR (Killed Vaccines) OR (Inactivated Vaccine) OR (Influenza Vaccines) OR (Influenza Vaccine) OR (Influenzavirus Vaccines) OR (Influenza Virus Vaccines) OR (Influenzavirus Vaccine) OR (Influenza Virus Vaccine) OR (High-Dose Trivalent Influenza Vaccine) OR (High Dose Trivalent Influenza Vaccine) OR (Trivalent Influenza Vaccine) OR (Quadrivalent Influenza Vaccine) OR "Pneumococcal Vaccines" [Mesh] OR (Pneumococcal Vaccines) OR (Pneumococcal Vaccine) OR (Pneumovax) OR (Pneumococcal Polysaccharide Vaccine) OR (Pnu Imune Vaccine) OR (PnuImune Vaccine) OR (Conjugated penumococcal vaccine) OR (Herpes Zoster Vaccine) OR (Zoster Vaccine) OR (Shingles Vaccine) OR "Hepatitis B Vaccines" [Mesh] OR (Hepatitis B Vaccines) OR (Hepatitis B Vaccine) OR "Papillomavirus Vaccines" [Mesh] OR (Papillomavirus Vaccines) OR (Papillomavirus Vaccine) OR (Human Papillomavirus Vaccines) OR (HPV Vaccine) OR (Human Papilloma Virus Vaccine) OR (HPV Vaccines) OR (Human Papilloma Virus Vaccines) OR (Human Papillomavirus Vaccine) OR "Poliovirus Vaccines" [Mesh] OR (Poliovirus Vaccines) OR (Poliovirus Vaccine) OR (salk) OR "Hepatitis A Vaccines" [Mesh] OR (Hepatitis A Vaccines) OR (Hepatovirus Vaccines) OR (Hepatitis A Vaccine) OR (Vaqta) OR (Havrix) OR "Meningococcal Vaccines" [Mesh] OR (Meningococcal Vaccine) OR (Menactra) OR (Tetravalent Meningococcal Vaccine) OR (Menomune) OR (Menveo) OR (Bivalent Meningococcal Vaccine) OR (Meningococcal Polysaccharide Vaccine) OR "Diphtheria-Tetanus-Pertussis Vaccine" [Mesh] OR (Diphtheria-Tetanus-Pertussis Vaccine) OR (Diphtheria Tetanus Pertussis Vaccine) OR (DTwP Vaccine) OR (DPT Vaccine) OR (DTP Vaccine) OR (Di Te Per Vaccine) OR (Diphtheria Pertussis Tetanus Vaccine) OR (Haemophilus influenza type b polysaccharide vaccine-tetanus toxin conjugate) OR (PRP-T) OR (Hib-TT) OR (OmniHIB) OR (ActHIB)

#1 AND #2 AND #3

("rheumatic diseases"[MeSH Terms] OR ("rheumatic"[All Fields] AND "diseases"[All Fields]) OR "rheumatic diseases"[All Fields] OR ("rheumatic diseases"[MeSH Terms] OR ("rheumatic"[All Fields] AND "diseases"[All Fields]) OR "rheumatic diseases"[All Fields] OR ("rheumatic"[All Fields] AND "disease"[All Fields]) OR "rheumatic disease"[All Fields]) OR ("rheumatic diseases"[MeSH Terms] OR ("rheumatic"[All Fields] AND "diseases"[All Fields]) OR "rheumatic diseases"[All Fields] OR "rheumatics"[All Fields] OR "rheumatism"[All Fields] OR "rheumatic"[All Fields] OR "rheumatisms"[All Fields]) OR ("Immune-mediated"[All Fields] AND ("rheumatic diseases"[MeSH Terms] OR ("rheumatic"[All Fields] AND "diseases"[All Fields]) OR "rheumatic diseases"[All Fields])) OR ("arthritis, rheumatoid"[MeSH Terms] OR ("arthritis"[All Fields] AND "rheumatoid"[All Fields]) OR "rheumatoid arthritis"[All Fields] OR ("arthritis"[All Fields] AND "rheumatoid"[All Fields]) OR "arthritis rheumatoid"[All Fields]) OR ("arthritis, rheumatoid"[MeSH Terms] OR ("arthritis"[All Fields] AND "rheumatoid"[All Fields]) OR "rheumatoid arthritis"[All Fields] OR ("rheumatoid"[All Fields] AND "arthritis"[All Fields])) OR ("rheumatoid nodule"[MeSH Terms] OR ("rheumatoid"[All Fields] AND "nodule"[All Fields]) OR "rheumatoid nodule"[All Fields]) OR ("rheumatoid vasculitis"[MeSH Terms] OR ("rheumatoid"[All Fields] AND "vasculitis"[All Fields]) OR "rheumatoid vasculitis"[All Fields]) OR ("Sjogren's Syndrome"[MeSH Terms] OR ("sjogren s"[All Fields] AND "syndrome"[All Fields]) OR "Sjogren's Syndrome"[All Fields]) OR "Spondylarthritis"[MeSH Terms] OR ("Spondylarthritis"[MeSH Terms] OR "Spondylarthritis"[All Fields] OR "spondylarthritides"[All Fields]) OR ("Spondylarthritis"[MeSH Terms] OR "Spondylarthritis"[All Fields] OR "spondylarthritides"[All Fields]) OR ("Spondylarthritis"[MeSH Terms] OR "Spondylarthritis"[All Fields] OR ("spinal"[All Fields] AND "arthritis"[All Fields]) OR "spinal arthritis"[All Fields]) OR "arthritis, psoriatic"[MeSH Terms] OR ("arthritis, psoriatic"[MeSH Terms] OR ("arthritis"[All Fields] AND "psoriatic"[All Fields]) OR "psoriatic arthritis"[All Fields] OR ("arthritis"[All Fields] AND "psoriatic"[All Fields]) OR "arthritis psoriatic"[All Fields]) OR ("arthritis, psoriatic"[MeSH Terms] OR ("arthritis"[All Fields] AND "psoriatic"[All Fields]) OR "psoriatic arthritis"[All Fields] OR ("arthritic"[All Fields] AND "psoriasis"[All Fields]) OR "arthritic psoriasis"[All Fields]) OR ("arthritis, psoriatic"[MeSH Terms] OR ("arthritis"[All Fields] AND "psoriatic"[All Fields]) OR "psoriatic arthritis"[All Fields] OR ("psoriatic"[All Fields] AND "arthritis"[All Fields])) OR ("arthritis, psoriatic"[MeSH Terms] OR ("arthritis"[All Fields] AND "psoriatic"[All Fields]) OR "psoriatic arthritis"[All Fields] OR ("psoriasis"[All Fields] AND "arthropathica"[All Fields]) OR "psoriasis arthropathica"[All Fields]) OR ("arthritis, psoriatic"[MeSH Terms] OR ("arthritis"[All Fields] AND "psoriatic"[All Fields]) OR "psoriatic arthritis"[All Fields] OR ("psoriatic"[All Fields] AND "arthropathy"[All Fields]) OR "psoriatic arthropathy"[All Fields]) OR ("arthritis, psoriatic"[MeSH Terms] OR ("arthritis"[All Fields] AND "psoriatic"[All Fields]) OR "psoriatic arthritis"[All Fields] OR ("psoriatic"[All Fields] AND "arthropathies"[All Fields]) OR "psoriatic arthropathies"[All Fields]) OR "spondylitis, ankylosing"[MeSH Terms] OR ("spondylitis, ankylosing"[MeSH Terms] OR ("spondylitis"[All Fields] AND "ankylosing"[All Fields]) OR "ankylosing spondylitis"[All Fields] OR ("spondylitis"[All Fields] AND "ankylosing"[All Fields]) OR "spondylitis ankylosing"[All Fields]) OR ("spondylitis, ankylosing"[MeSH Terms] OR ("spondylitis"[All Fields] AND "ankylosing"[All Fields]) OR "ankylosing spondylitis"[All Fields] OR ("bechterew s"[All Fields] AND "disease"[All Fields]) OR "bechterew s disease"[All Fields]) OR ("spondylitis, ankylosing"[MeSH Terms] OR ("spondylitis"[All Fields] AND "ankylosing"[All Fields]) OR "ankylosing spondylitis"[All Fields] OR ("bechterews"[All Fields] AND "disease"[All Fields]) OR "bechterews disease"[All Fields]) OR ("spondylitis, ankylosing"[MeSH Terms] OR ("spondylitis"[All Fields] AND "ankylosing"[All Fields]) OR "ankylosing spondylitis"[All Fields] OR ("marie"[All Fields] AND "struempell"[All Fields] AND "disease"[All Fields]) OR "marie struempell disease"[All Fields]) OR ("spondylitis, ankylosing"[MeSH Terms] OR ("spondylitis"[All Fields] AND "ankylosing"[All Fields]) OR "ankylosing spondylitis"[All Fields] OR ("Spondylarthritis"[All Fields] AND "ankylopoietica"[All Fields]) OR "spondylarthritis ankylopoietica"[All Fields]) OR ("spondylitis, ankylosing"[MeSH Terms] OR ("spondylitis"[All Fields] AND "ankylosing"[All Fields]) OR "ankylosing spondylitis"[All Fields] OR ("spondyloarthritis"[All Fields] AND "ankylopoietica"[All Fields]) OR "spondyloarthritis ankylopoietica"[All Fields]) OR ("spondylitis, ankylosing"[MeSH Terms] OR ("spondylitis"[All Fields] AND "ankylosing"[All Fields]) OR "ankylosing spondylitis"[All Fields] OR ("ankylosing"[All Fields] AND "spondylitis"[All Fields])) OR ("spondylitis, ankylosing"[MeSH Terms] OR ("spondylitis"[All Fields] AND "ankylosing"[All Fields]) OR "ankylosing spondylitis"[All Fields] OR ("ankylosing"[All Fields] AND "Spondylarthritis"[All Fields]) OR "ankylosing spondylarthritis"[All Fields]) OR ("spondylitis, ankylosing"[MeSH Terms] OR ("spondylitis"[All Fields] AND "ankylosing"[All Fields]) OR "ankylosing spondylitis"[All Fields] OR ("ankylosing"[All Fields] AND "spondylarthritides"[All Fields]) OR "ankylosing spondylarthritides"[All Fields]) OR ("spondylitis, ankylosing"[MeSH Terms] OR ("spondylitis"[All Fields] AND "ankylosing"[All Fields]) OR "ankylosing spondylitis"[All Fields] OR ("ankylosing"[All Fields] AND "spondyloarthritis"[All Fields]) OR "ankylosing spondyloarthritis"[All Fields]) OR ("spondylitis, ankylosing"[MeSH Terms] OR ("spondylitis"[All Fields] AND "ankylosing"[All Fields]) OR "ankylosing spondylitis"[All Fields] OR ("spondylitis"[All Fields] AND "ankylopoietica"[All Fields]) OR "spondylitis ankylopoietica"[All Fields]) OR ("spondylitis, ankylosing"[MeSH Terms] OR ("spondylitis"[All Fields] AND "ankylosing"[All Fields]) OR "ankylosing spondylitis"[All Fields] OR ("bechterew"[All Fields] AND "disease"[All Fields]) OR "bechterew disease"[All Fields]) OR ("spondylitis, ankylosing"[MeSH Terms] OR ("spondylitis"[All Fields] AND "ankylosing"[All Fields]) OR "ankylosing spondylitis"[All Fields] OR ("rheumatoid"[All Fields] AND "spondylitis"[All Fields]) OR "rheumatoid spondylitis"[All Fields]) OR "lupus erythematosus, systemic"[MeSH Terms] OR ("lupus erythematosus, systemic"[MeSH Terms] OR ("lupus"[All Fields] AND "erythematosus"[All Fields] AND "systemic"[All Fields]) OR "systemic lupus erythematosus"[All Fields] OR ("lupus"[All Fields] AND "erythematosus"[All Fields] AND "systemic"[All Fields]) OR "lupus erythematosus systemic"[All Fields]) OR ("lupus erythematosus, systemic"[MeSH Terms] OR ("lupus"[All Fields] AND "erythematosus"[All Fields] AND "systemic"[All Fields]) OR "systemic lupus erythematosus"[All Fields] OR ("systemic"[All Fields] AND "lupus"[All Fields] AND "erythematosus"[All Fields])) OR ("lupus erythematosus, systemic"[MeSH Terms] OR ("lupus"[All Fields] AND "erythematosus"[All Fields] AND "systemic"[All Fields]) OR "systemic lupus erythematosus"[All Fields] OR ("lupus"[All Fields] AND "erythematosus"[All Fields] AND "disseminatus"[All Fields]) OR "lupus erythematosus disseminatus"[All Fields]) OR ("lupus erythematosus, systemic"[MeSH Terms] OR ("lupus"[All Fields] AND "erythematosus"[All Fields] AND "systemic"[All Fields]) OR "systemic lupus erythematosus"[All Fields] OR ("libman"[All Fields] AND "sacks"[All Fields] AND "disease"[All Fields]) OR "libman sacks disease"[All Fields]) OR "Myositis"[MeSH Terms] OR ("Myositis"[MeSH Terms] OR "Myositis"[All Fields] OR "myositides"[All Fields]) OR ("Myositis"[MeSH Terms] OR "Myositis"[All Fields] OR "myositides"[All Fields]) OR ("Myositis"[MeSH Terms] OR "Myositis"[All Fields] OR ("inflammatory"[All Fields] AND "muscle"[All Fields] AND "diseases"[All Fields]) OR "inflammatory muscle diseases"[All Fields]) OR ("Myositis"[MeSH Terms] OR "Myositis"[All Fields] OR ("inflammatory"[All Fields] AND "muscle"[All Fields] AND "disease"[All Fields]) OR "inflammatory muscle disease"[All Fields]) OR ("Myositis"[MeSH Terms] OR "Myositis"[All Fields] OR ("inflammatory"[All Fields] AND "myopathy"[All Fields]) OR "inflammatory myopathy"[All Fields]) OR ("Myositis"[MeSH Terms] OR "Myositis"[All Fields] OR ("inflammatory"[All Fields] AND "myopathies"[All Fields]) OR "inflammatory myopathies"[All Fields]) OR ("Myositis"[MeSH Terms] OR "Myositis"[All Fields] OR ("proliferative"[All Fields] AND "Myositis"[All Fields]) OR "proliferative myositis"[All Fields]) OR ("Myositis"[MeSH Terms] OR "Myositis"[All Fields] OR ("infectious"[All Fields] AND "myositides"[All Fields]) OR "infectious myositides"[All Fields]) OR ("Myositis"[MeSH Terms] OR "Myositis"[All Fields] OR ("infectious"[All Fields] AND "Myositis"[All Fields]) OR "infectious myositis"[All Fields]) OR ("Myositis"[MeSH Terms] OR "Myositis"[All Fields] OR ("idiopathic"[All Fields] AND "inflammatory"[All Fields] AND "myopathies"[All Fields]) OR "idiopathic inflammatory myopathies"[All Fields]) OR ("Myositis"[MeSH Terms] OR "Myositis"[All Fields] OR ("idiopathic"[All Fields] AND "inflammatory"[All Fields] AND "myopathy"[All Fields]) OR "idiopathic inflammatory myopathy"[All Fields]) OR ("Myositis"[MeSH Terms] OR "Myositis"[All Fields] OR ("idiopathic"[All Fields] AND "inflammatory"[All Fields] AND "Myositis"[All Fields]) OR "idiopathic inflammatory myositis"[All Fields]) OR ("Myositis"[MeSH Terms] OR "Myositis"[All Fields] OR ("focal"[All Fields] AND "Myositis"[All Fields]) OR "focal myositis"[All Fields]) OR "Systemic Vasculitis"[MeSH Terms] OR ("Systemic Vasculitis"[MeSH Terms] OR ("systemic"[All Fields] AND "vasculitis"[All Fields]) OR "Systemic Vasculitis"[All Fields]) OR ("Systemic Vasculitis"[MeSH Terms] OR ("systemic"[All Fields] AND "vasculitis"[All Fields]) OR "Systemic Vasculitis"[All Fields] OR ("systemic"[All Fields] AND "vasculitides"[All Fields]) OR "systemic vasculitides"[All Fields]) OR "Sjogren's Syndrome"[MeSH Terms] OR ("Sjogren's Syndrome"[MeSH Terms] OR ("sjogren s"[All Fields] AND "syndrome"[All Fields]) OR "Sjogren's Syndrome"[All Fields]) OR ("Sjogren's Syndrome"[MeSH Terms] OR ("sjogren s"[All Fields] AND "syndrome"[All Fields]) OR "Sjogren's Syndrome"[All Fields] OR ("sjogrens"[All Fields] AND "syndrome"[All Fields]) OR "sjogrens syndrome"[All Fields]) OR ("Sjogren's Syndrome"[MeSH Terms] OR ("sjogren s"[All Fields] AND "syndrome"[All Fields]) OR "Sjogren's Syndrome"[All Fields] OR ("sjogren"[All Fields] AND "syndrome"[All Fields]) OR "sjogren syndrome"[All Fields]) OR ("Sjogren's Syndrome"[MeSH Terms] OR ("sjogren s"[All Fields] AND "syndrome"[All Fields]) OR "Sjogren's Syndrome"[All Fields] OR ("sicca"[All Fields] AND "syndrome"[All Fields]) OR "sicca syndrome"[All Fields]) OR "scleroderma, systemic"[MeSH Terms] OR ("scleroderma, systemic"[MeSH Terms] OR ("scleroderma"[All Fields] AND "systemic"[All Fields]) OR "systemic scleroderma"[All Fields] OR ("scleroderma"[All Fields] AND "systemic"[All Fields]) OR "scleroderma systemic"[All Fields]) OR ("scleroderma, systemic"[MeSH Terms] OR ("scleroderma"[All Fields] AND "systemic"[All Fields]) OR "systemic scleroderma"[All Fields] OR ("systemic"[All Fields] AND "sclerosis"[All Fields]) OR "systemic sclerosis"[All Fields]) OR ("scleroderma, systemic"[MeSH Terms] OR ("scleroderma"[All Fields] AND "systemic"[All Fields]) OR "systemic scleroderma"[All Fields] OR ("systemic"[All Fields] AND "scleroderma"[All Fields])) OR "Mixed Connective Tissue Disease"[MeSH Terms] OR ("Mixed Connective Tissue Disease"[MeSH Terms] OR ("mixed"[All Fields] AND "connective"[All Fields] AND "tissue"[All Fields] AND "disease"[All Fields]) OR "Mixed Connective Tissue Disease"[All Fields]) OR ("Mixed Connective Tissue Disease"[MeSH Terms] OR ("mixed"[All Fields] AND "connective"[All Fields] AND "tissue"[All Fields] AND "disease"[All Fields]) OR "Mixed Connective Tissue Disease"[All Fields] OR ("sharp"[All Fields] AND "syndrome"[All Fields]) OR "sharp syndrome"[All Fields]) OR ("Mixed Connective Tissue Disease"[MeSH Terms] OR ("mixed"[All Fields] AND "connective"[All Fields] AND "tissue"[All Fields] AND "disease"[All Fields]) OR "Mixed Connective Tissue Disease"[All Fields] OR "mctd"[All Fields])) AND ("Janus Kinase Inhibitors"[MeSH Terms] OR ("Janus Kinase Inhibitors"[Pharmacological Action] OR "Janus Kinase Inhibitors"[MeSH Terms] OR ("janus"[All Fields] AND "kinase"[All Fields] AND "inhibitors"[All Fields]) OR "Janus Kinase Inhibitors"[All Fields]) OR ("Janus Kinase Inhibitors"[Pharmacological Action] OR "Janus Kinase Inhibitors"[MeSH Terms] OR ("janus"[All Fields] AND "kinase"[All Fields] AND "inhibitors"[All Fields]) OR "Janus Kinase Inhibitors"[All Fields] OR ("jak"[All Fields] AND "inhibitors"[All Fields]) OR "jak inhibitors"[All Fields]) OR ("Janus Kinase Inhibitors"[Pharmacological Action] OR "Janus Kinase Inhibitors"[MeSH Terms] OR ("janus"[All Fields] AND "kinase"[All Fields] AND "inhibitors"[All Fields]) OR "Janus Kinase Inhibitors"[All Fields] OR ("janus"[All Fields] AND "kinase"[All Fields] AND "inhibitor"[All Fields]) OR "janus kinase inhibitor"[All Fields]) OR ("Janus Kinase Inhibitors"[Pharmacological Action] OR "Janus Kinase Inhibitors"[MeSH Terms] OR ("janus"[All Fields] AND "kinase"[All Fields] AND "inhibitors"[All Fields]) OR "Janus Kinase Inhibitors"[All Fields] OR ("jak"[All Fields] AND "inhibitor"[All Fields]) OR "jak inhibitor"[All Fields]) OR ("upadacitinib"[Supplementary Concept] OR "upadacitinib"[All Fields]) OR ("upadacitinib"[Supplementary Concept] OR "upadacitinib"[All Fields] OR "abt 494"[All Fields]) OR ("upadacitinib"[Supplementary Concept] OR "upadacitinib"[All Fields] OR "rinvoq"[All Fields]) OR "Protein Kinase Inhibitors"[MeSH Terms] OR ("Protein Kinase Inhibitors"[Pharmacological Action] OR "Protein Kinase Inhibitors"[MeSH Terms] OR ("protein"[All Fields] AND "kinase"[All Fields] AND "inhibitors"[All Fields]) OR "Protein Kinase Inhibitors"[All Fields]) OR ("Protein Kinase Inhibitors"[Pharmacological Action] OR "Protein Kinase Inhibitors"[MeSH Terms] OR ("protein"[All Fields] AND "kinase"[All Fields] AND "inhibitors"[All Fields]) OR "Protein Kinase Inhibitors"[All Fields] OR ("protein"[All Fields] AND "kinase"[All Fields] AND "inhibitor"[All Fields]) OR "protein kinase inhibitor"[All Fields]) OR ("tofacitinib"[Supplementary Concept] OR "tofacitinib"[All Fields] OR "tofacitinib s"[All Fields]) OR ("tofacitinib"[Supplementary Concept] OR "tofacitinib"[All Fields] OR "tasocitinib"[All Fields]) OR ("tofacitinib"[Supplementary Concept] OR "tofacitinib"[All Fields] OR "tofacitinib citrate"[All Fields]) OR ("tofacitinib"[Supplementary Concept] OR "tofacitinib"[All Fields] OR "xeljanz"[All Fields] OR "tofacitinib s"[All Fields]) OR ("tofacitinib"[Supplementary Concept] OR "tofacitinib"[All Fields] OR "cp 690550"[All Fields]) OR ("baricitinib"[Supplementary Concept] OR "baricitinib"[All Fields]) OR ("baricitinib"[Supplementary Concept] OR "baricitinib"[All Fields]) OR ("baricitinib"[Supplementary Concept] OR "baricitinib"[All Fields] OR "olumiant"[All Fields]) OR ("baricitinib"[Supplementary Concept] OR "baricitinib"[All Fields]) OR ("baricitinib"[Supplementary Concept] OR "baricitinib"[All Fields]) OR ("baricitinib"[Supplementary Concept] OR "baricitinib"[All Fields] OR "incb028050"[All Fields]) OR ("baricitinib"[Supplementary Concept] OR "baricitinib"[All Fields] OR "ly3009104"[All Fields])) AND ("vaccines, inactivated"[MeSH Terms] OR ("vaccines, inactivated"[MeSH Terms] OR ("vaccines"[All Fields] AND "inactivated"[All Fields]) OR "inactivated vaccines"[All Fields] OR ("vaccines"[All Fields] AND "inactivated"[All Fields]) OR "vaccines inactivated"[All Fields]) OR ("vaccines, inactivated"[MeSH Terms] OR ("vaccines"[All Fields] AND "inactivated"[All Fields]) OR "inactivated vaccines"[All Fields] OR ("inactivated"[All Fields] AND "vaccines"[All Fields])) OR ("vaccines, inactivated"[MeSH Terms] OR ("vaccines"[All Fields] AND "inactivated"[All Fields]) OR "inactivated vaccines"[All Fields] OR ("killed"[All Fields] AND "vaccine"[All Fields]) OR "killed vaccine"[All Fields]) OR ("vaccines, inactivated"[MeSH Terms] OR ("vaccines"[All Fields] AND "inactivated"[All Fields]) OR "inactivated vaccines"[All Fields] OR ("killed"[All Fields] AND "vaccines"[All Fields]) OR "killed vaccines"[All Fields]) OR ("vaccines, inactivated"[MeSH Terms] OR ("vaccines"[All Fields] AND "inactivated"[All Fields]) OR "inactivated vaccines"[All Fields] OR ("inactivated"[All Fields] AND "vaccine"[All Fields]) OR "inactivated vaccine"[All Fields]) OR ("influenza vaccines"[MeSH Terms] OR ("influenza"[All Fields] AND "vaccines"[All Fields]) OR "influenza vaccines"[All Fields]) OR ("influenza vaccines"[MeSH Terms] OR ("influenza"[All Fields] AND "vaccines"[All Fields]) OR "influenza vaccines"[All Fields] OR ("influenza"[All Fields] AND "vaccine"[All Fields]) OR "influenza vaccine"[All Fields]) OR ("influenza vaccines"[MeSH Terms] OR ("influenza"[All Fields] AND "vaccines"[All Fields]) OR "influenza vaccines"[All Fields] OR ("influenzavirus"[All Fields] AND "vaccines"[All Fields])) OR ("influenza vaccines"[MeSH Terms] OR ("influenza"[All Fields] AND "vaccines"[All Fields]) OR "influenza vaccines"[All Fields] OR ("influenza"[All Fields] AND "virus"[All Fields] AND "vaccines"[All Fields]) OR "influenza virus vaccines"[All Fields]) OR ("influenza vaccines"[MeSH Terms] OR ("influenza"[All Fields] AND "vaccines"[All Fields]) OR "influenza vaccines"[All Fields] OR ("influenzavirus"[All Fields] AND "vaccine"[All Fields]) OR "influenzavirus vaccine"[All Fields]) OR ("influenza vaccines"[MeSH Terms] OR ("influenza"[All Fields] AND "vaccines"[All Fields]) OR "influenza vaccines"[All Fields] OR ("influenza"[All Fields] AND "virus"[All Fields] AND "vaccine"[All Fields]) OR "influenza virus vaccine"[All Fields]) OR ("influenza vaccines"[MeSH Terms] OR ("influenza"[All Fields] AND "vaccines"[All Fields]) OR "influenza vaccines"[All Fields] OR ("high"[All Fields] AND "dose"[All Fields] AND "trivalent"[All Fields] AND "influenza"[All Fields] AND "vaccine"[All Fields]) OR "high dose trivalent influenza vaccine"[All Fields]) OR ("influenza vaccines"[MeSH Terms] OR ("influenza"[All Fields] AND "vaccines"[All Fields]) OR "influenza vaccines"[All Fields] OR ("high"[All Fields] AND "dose"[All Fields] AND "trivalent"[All Fields] AND "influenza"[All Fields] AND "vaccine"[All Fields]) OR "high dose trivalent influenza vaccine"[All Fields]) OR ("influenza vaccines"[MeSH Terms] OR ("influenza"[All Fields] AND "vaccines"[All Fields]) OR "influenza vaccines"[All Fields] OR ("trivalent"[All Fields] AND "influenza"[All Fields] AND "vaccine"[All Fields]) OR "trivalent influenza vaccine"[All Fields]) OR ("influenza vaccines"[MeSH Terms] OR ("influenza"[All Fields] AND "vaccines"[All Fields]) OR "influenza vaccines"[All Fields] OR ("quadrivalent"[All Fields] AND "influenza"[All Fields] AND "vaccine"[All Fields]) OR "quadrivalent influenza vaccine"[All Fields]) OR "Pneumococcal Vaccines"[MeSH Terms] OR ("Pneumococcal Vaccines"[MeSH Terms] OR ("pneumococcal"[All Fields] AND "vaccines"[All Fields]) OR "Pneumococcal Vaccines"[All Fields]) OR ("Pneumococcal Vaccines"[MeSH Terms] OR ("pneumococcal"[All Fields] AND "vaccines"[All Fields]) OR "Pneumococcal Vaccines"[All Fields] OR ("pneumococcal"[All Fields] AND "vaccine"[All Fields]) OR "pneumococcal vaccine"[All Fields]) OR ("Pneumococcal Vaccines"[MeSH Terms] OR ("pneumococcal"[All Fields] AND "vaccines"[All Fields]) OR "Pneumococcal Vaccines"[All Fields] OR "pneumovax"[All Fields]) OR ("Pneumococcal Vaccines"[MeSH Terms] OR ("pneumococcal"[All Fields] AND "vaccines"[All Fields]) OR "Pneumococcal Vaccines"[All Fields] OR ("pneumococcal"[All Fields] AND "polysaccharide"[All Fields] AND "vaccine"[All Fields]) OR "pneumococcal polysaccharide vaccine"[All Fields]) OR ("Pneumococcal Vaccines"[MeSH Terms] OR ("pneumococcal"[All Fields] AND "vaccines"[All Fields]) OR "Pneumococcal Vaccines"[All Fields] OR ("pnu"[All Fields] AND "imune"[All Fields] AND "vaccine"[All Fields]) OR "pnu imune vaccine"[All Fields]) OR ("Pneumococcal Vaccines"[MeSH Terms] OR ("pneumococcal"[All Fields] AND "vaccines"[All Fields]) OR "Pneumococcal Vaccines"[All Fields] OR ("pnuimune"[All Fields] AND "vaccine"[All Fields])) OR (("conjugable"[All Fields] OR "conjugal"[All Fields] OR "conjugally"[All Fields] OR "conjugant"[All Fields] OR "conjugants"[All Fields] OR "conjugate"[All Fields] OR "conjugate s"[All Fields] OR "conjugated"[All Fields] OR "conjugates"[All Fields] OR "conjugating"[All Fields] OR "conjugation"[All Fields] OR "conjugational"[All Fields] OR "conjugations"[All Fields] OR "conjugative"[All Fields] OR "conjugatively"[All Fields]) AND "penumococcal"[All Fields] AND ("vaccin"[Supplementary Concept] OR "vaccin"[All Fields] OR "vaccination"[MeSH Terms] OR "vaccination"[All Fields] OR "vaccinable"[All Fields] OR "vaccinal"[All Fields] OR "vaccinate"[All Fields] OR "vaccinated"[All Fields] OR "vaccinates"[All Fields] OR "vaccinating"[All Fields] OR "vaccinations"[All Fields] OR "vaccination s"[All Fields] OR "vaccinator"[All Fields] OR "vaccinators"[All Fields] OR "vaccine s"[All Fields] OR "vaccined"[All Fields] OR "vaccines"[MeSH Terms] OR "vaccines"[All Fields] OR "vaccine"[All Fields] OR "vaccins"[All Fields])) OR ("herpes zoster vaccine"[MeSH Terms] OR ("herpes"[All Fields] AND "zoster"[All Fields] AND "vaccine"[All Fields]) OR "herpes zoster vaccine"[All Fields]) OR ("herpes zoster vaccine"[MeSH Terms] OR ("herpes"[All Fields] AND "zoster"[All Fields] AND "vaccine"[All Fields]) OR "herpes zoster vaccine"[All Fields] OR ("zoster"[All Fields] AND "vaccine"[All Fields]) OR "zoster vaccine"[All Fields]) OR ("herpes zoster vaccine"[MeSH Terms] OR ("herpes"[All Fields] AND "zoster"[All Fields] AND "vaccine"[All Fields]) OR "herpes zoster vaccine"[All Fields] OR ("shingles"[All Fields] AND "vaccine"[All Fields]) OR "shingles vaccine"[All Fields]) OR "Hepatitis B Vaccines"[MeSH Terms] OR ("Hepatitis B Vaccines"[MeSH Terms] OR "Hepatitis B Vaccines"[All Fields]) OR ("Hepatitis B Vaccines"[MeSH Terms] OR "Hepatitis B Vaccines"[All Fields] OR "hepatitis b vaccine"[All Fields]) OR "Papillomavirus Vaccines"[MeSH Terms] OR ("Papillomavirus Vaccines"[MeSH Terms] OR ("papillomavirus"[All Fields] AND "vaccines"[All Fields]) OR "Papillomavirus Vaccines"[All Fields]) OR ("Papillomavirus Vaccines"[MeSH Terms] OR ("papillomavirus"[All Fields] AND "vaccines"[All Fields]) OR "Papillomavirus Vaccines"[All Fields] OR ("papillomavirus"[All Fields] AND "vaccine"[All Fields]) OR "papillomavirus vaccine"[All Fields]) OR ("Papillomavirus Vaccines"[MeSH Terms] OR ("papillomavirus"[All Fields] AND "vaccines"[All Fields]) OR "Papillomavirus Vaccines"[All Fields] OR ("human"[All Fields] AND "papillomavirus"[All Fields] AND "vaccines"[All Fields]) OR "human papillomavirus vaccines"[All Fields]) OR ("Papillomavirus Vaccines"[MeSH Terms] OR ("papillomavirus"[All Fields] AND "vaccines"[All Fields]) OR "Papillomavirus Vaccines"[All Fields] OR ("hpv"[All Fields] AND "vaccine"[All Fields]) OR "hpv vaccine"[All Fields]) OR ("Papillomavirus Vaccines"[MeSH Terms] OR ("papillomavirus"[All Fields] AND "vaccines"[All Fields]) OR "Papillomavirus Vaccines"[All Fields] OR ("human"[All Fields] AND "papilloma"[All Fields] AND "virus"[All Fields] AND "vaccine"[All Fields]) OR "human papilloma virus vaccine"[All Fields]) OR ("Papillomavirus Vaccines"[MeSH Terms] OR ("papillomavirus"[All Fields] AND "vaccines"[All Fields]) OR "Papillomavirus Vaccines"[All Fields] OR ("hpv"[All Fields] AND "vaccines"[All Fields]) OR "hpv vaccines"[All Fields]) OR ("Papillomavirus Vaccines"[MeSH Terms] OR ("papillomavirus"[All Fields] AND "vaccines"[All Fields]) OR "Papillomavirus Vaccines"[All Fields] OR ("human"[All Fields] AND "papilloma"[All Fields] AND "virus"[All Fields] AND "vaccines"[All Fields]) OR "human papilloma virus vaccines"[All Fields]) OR ("Papillomavirus Vaccines"[MeSH Terms] OR ("papillomavirus"[All Fields] AND "vaccines"[All Fields]) OR "Papillomavirus Vaccines"[All Fields] OR ("human"[All Fields] AND "papillomavirus"[All Fields] AND "vaccine"[All Fields]) OR "human papillomavirus vaccine"[All Fields]) OR "Poliovirus Vaccines"[MeSH Terms] OR ("Poliovirus Vaccines"[MeSH Terms] OR ("poliovirus"[All Fields] AND "vaccines"[All Fields]) OR "Poliovirus Vaccines"[All Fields]) OR ("Poliovirus Vaccines"[MeSH Terms] OR ("poliovirus"[All Fields] AND "vaccines"[All Fields]) OR "Poliovirus Vaccines"[All Fields] OR ("poliovirus"[All Fields] AND "vaccine"[All Fields]) OR "poliovirus vaccine"[All Fields]) OR "salk"[All Fields] OR "Hepatitis A Vaccines"[MeSH Terms] OR ("Hepatitis A Vaccines"[MeSH Terms] OR "Hepatitis A Vaccines"[All Fields]) OR ("Hepatitis A Vaccines"[MeSH Terms] OR "Hepatitis A Vaccines"[All Fields] OR ("hepatovirus"[All Fields] AND "vaccines"[All Fields])) OR ("Hepatitis A Vaccines"[MeSH Terms] OR "Hepatitis A Vaccines"[All Fields] OR "hepatitis a vaccine"[All Fields]) OR ("hepatitis a vaccine inactivated"[All Fields] OR "Hepatitis A Vaccines"[MeSH Terms] OR "Hepatitis A Vaccines"[All Fields] OR "vaqta"[All Fields]) OR ("Hepatitis A Vaccines"[MeSH Terms] OR "Hepatitis A Vaccines"[All Fields] OR "havrix"[All Fields]) OR "Meningococcal Vaccines"[MeSH Terms] OR ("Meningococcal Vaccines"[MeSH Terms] OR ("meningococcal"[All Fields] AND "vaccines"[All Fields]) OR "Meningococcal Vaccines"[All Fields] OR ("meningococcal"[All Fields] AND "vaccine"[All Fields]) OR "meningococcal vaccine"[All Fields]) OR ("Meningococcal Vaccines"[MeSH Terms] OR ("meningococcal"[All Fields] AND "vaccines"[All Fields]) OR "Meningococcal Vaccines"[All Fields] OR "menactra"[All Fields]) OR ("Meningococcal Vaccines"[MeSH Terms] OR ("meningococcal"[All Fields] AND "vaccines"[All Fields]) OR "Meningococcal Vaccines"[All Fields] OR ("tetravalent"[All Fields] AND "meningococcal"[All Fields] AND "vaccine"[All Fields]) OR "tetravalent meningococcal vaccine"[All Fields]) OR ("Meningococcal Vaccines"[MeSH Terms] OR ("meningococcal"[All Fields] AND "vaccines"[All Fields]) OR "Meningococcal Vaccines"[All Fields] OR "menomune"[All Fields]) OR ("Meningococcal Vaccines"[MeSH Terms] OR ("meningococcal"[All Fields] AND "vaccines"[All Fields]) OR "Meningococcal Vaccines"[All Fields] OR "menveo"[All Fields]) OR ("Meningococcal Vaccines"[MeSH Terms] OR ("meningococcal"[All Fields] AND "vaccines"[All Fields]) OR "Meningococcal Vaccines"[All Fields] OR ("bivalent"[All Fields] AND "meningococcal"[All Fields] AND "vaccine"[All Fields]) OR "bivalent meningococcal vaccine"[All Fields]) OR ("Meningococcal Vaccines"[MeSH Terms] OR ("meningococcal"[All Fields] AND "vaccines"[All Fields]) OR "Meningococcal Vaccines"[All Fields] OR ("meningococcal"[All Fields] AND "polysaccharide"[All Fields] AND "vaccine"[All Fields]) OR "meningococcal polysaccharide vaccine"[All Fields]) OR "Diphtheria-Tetanus-Pertussis Vaccine"[MeSH Terms] OR ("Diphtheria-Tetanus-Pertussis Vaccine"[MeSH Terms] OR ("diphtheria tetanus pertussis"[All Fields] AND "vaccine"[All Fields]) OR "Diphtheria-Tetanus-Pertussis Vaccine"[All Fields] OR ("diphtheria"[All Fields] AND "tetanus"[All Fields] AND "pertussis"[All Fields] AND "vaccine"[All Fields]) OR "Diphtheria-Tetanus-Pertussis Vaccine"[All Fields]) OR ("Diphtheria-Tetanus-Pertussis Vaccine"[MeSH Terms] OR ("diphtheria tetanus pertussis"[All Fields] AND "vaccine"[All Fields]) OR "Diphtheria-Tetanus-Pertussis Vaccine"[All Fields] OR ("diphtheria"[All Fields] AND "tetanus"[All Fields] AND "pertussis"[All Fields] AND "vaccine"[All Fields]) OR "Diphtheria-Tetanus-Pertussis Vaccine"[All Fields]) OR ("Diphtheria-Tetanus-Pertussis Vaccine"[MeSH Terms] OR ("diphtheria tetanus pertussis"[All Fields] AND "vaccine"[All Fields]) OR "Diphtheria-Tetanus-Pertussis Vaccine"[All Fields] OR ("dtwp"[All Fields] AND "vaccine"[All Fields]) OR "dtwp vaccine"[All Fields]) OR ("Diphtheria-Tetanus-Pertussis Vaccine"[MeSH Terms] OR ("diphtheria tetanus pertussis"[All Fields] AND "vaccine"[All Fields]) OR "Diphtheria-Tetanus-Pertussis Vaccine"[All Fields] OR ("dpt"[All Fields] AND "vaccine"[All Fields]) OR "dpt vaccine"[All Fields]) OR ("Diphtheria-Tetanus-Pertussis Vaccine"[MeSH Terms] OR ("diphtheria tetanus pertussis"[All Fields] AND "vaccine"[All Fields]) OR "Diphtheria-Tetanus-Pertussis Vaccine"[All Fields] OR ("dtp"[All Fields] AND "vaccine"[All Fields]) OR "dtp vaccine"[All Fields]) OR ("Diphtheria-Tetanus-Pertussis Vaccine"[MeSH Terms] OR ("diphtheria tetanus pertussis"[All Fields] AND "vaccine"[All Fields]) OR "Diphtheria-Tetanus-Pertussis Vaccine"[All Fields] OR ("di"[All Fields] AND "te"[All Fields] AND "per"[All Fields] AND "vaccine"[All Fields]) OR "di te per vaccine"[All Fields]) OR ("Diphtheria-Tetanus-Pertussis Vaccine"[MeSH Terms] OR ("diphtheria tetanus pertussis"[All Fields] AND "vaccine"[All Fields]) OR "Diphtheria-Tetanus-Pertussis Vaccine"[All Fields] OR ("diphtheria"[All Fields] AND "pertussis"[All Fields] AND "tetanus"[All Fields] AND "vaccine"[All Fields]) OR "diphtheria pertussis tetanus vaccine"[All Fields]) OR ("haemophilus influenza type b polysaccharide vaccine tetanus toxin conjugate"[Supplementary Concept] OR "haemophilus influenza type b polysaccharide vaccine tetanus toxin conjugate"[All Fields] OR "haemophilus influenza type b polysaccharide vaccine tetanus toxin conjugate"[All Fields]) OR ("haemophilus influenza type b polysaccharide vaccine tetanus toxin conjugate"[Supplementary Concept] OR "haemophilus influenza type b polysaccharide vaccine tetanus toxin conjugate"[All Fields] OR "prp t"[All Fields]) OR ("haemophilus influenza type b polysaccharide vaccine tetanus toxin conjugate"[Supplementary Concept] OR "haemophilus influenza type b polysaccharide vaccine tetanus toxin conjugate"[All Fields] OR "hib tt"[All Fields]) OR ("haemophilus influenza type b polysaccharide vaccine tetanus toxin conjugate"[Supplementary Concept] OR "haemophilus influenza type b polysaccharide vaccine tetanus toxin conjugate"[All Fields] OR "omnihib"[All Fields]) OR ("haemophilus influenza type b polysaccharide vaccine tetanus toxin conjugate"[Supplementary Concept] OR "haemophilus influenza type b polysaccharide vaccine tetanus toxin conjugate"[All Fields] OR "acthib"[All Fields]))

24 results

**1.2 Following there are search strategy in Embase**

Results

450

#32 #25 AND #31

715,285

#31 #26 OR #27 OR #28 OR #29 OR #30

4,696

#30 'baricitinib'/syn

9,684

#29 'tofacitinib'/syn

714,689

#28 'protein kinase inhibitor'/syn

2,099

#27 'upadacitinib'/syn

30,220

#26 'janus kinase inhibitor'/syn

3,124

#25 #12 AND #24

149,994

#24 #13 OR #14 OR #15 OR #16 OR #17 OR #18 OR #19 OR #20 OR #21 OR #22 OR #23

36

#23 'haemophilus influenza type b polysaccharide vaccine tetanus toxin conjugate'/syn

22,584

#22 'diphtheria pertussis tetanus vaccine'/syn

8,774

#21 'meningococcal vaccines'/syn

5,982

#20 'hepatitis a vaccines'/syn

15,713

#19 'poliovirus vaccines'/syn

18,091

#18 'human papilloma virus vaccine'/syn

25,125

#17 'hepatitis b vaccine'/syn

4,441

#16 'herpes zoster vaccine'/syn

25,896

#15 'pneumococcus vaccine'/syn

48,734

#14 'influenza vaccines'/syn

10,379

#13 'inactivated vaccine'/syn

582,415

#12 #1 OR #2 OR #3 OR #4 OR #5 OR #6 OR #7 OR #8 OR #9 OR #10 OR #11

5,506

#11 'mixed connective tissue disease'/syn

43,158

#10 'systemic sclerosis'/syn

35,919

#9 'sjoegren syndrome'/syn

9,231

#8 'systemic vasculitis'/syn

52,684

#7 'myositis'/syn

141,094

#6 'systemic lupus erythematosus'/syn

40,970

#5 'ankylosing spondylitis'/syn

33,640

#4'psoriatic arthritis'/syn                                                                                                                                      73,128

#3'spondylarthritis'/syn

282,694

#2'rheumatoid arthritis'/syn

56,077

#1 'rheumatic disease'/de

**1.3 Following there are search strategy in Cochrane Library**

ID	Search	Hits

#1	MeSH descriptor: [Rheumatic Diseases] explode all trees	20731

#2	MeSH descriptor: [Arthritis, Rheumatoid] explode all trees	7302

#3	MeSH descriptor: [Spondylarthritis] explode all trees	1823

#4	MeSH descriptor: [Arthritis, Psoriatic] explode all trees	634

#5	MeSH descriptor: [Spondylitis, Ankylosing] explode all trees	826

#6	MeSH descriptor: [Lupus Erythematosus, Systemic] explode all trees	1425

#7	MeSH descriptor: [Myositis] explode all trees	318

#8	MeSH descriptor: [Systemic Vasculitis] explode all trees	266

#9	MeSH descriptor: [Sjogren's Syndrome] explode all trees	365

#10	MeSH descriptor: [Scleroderma, Systemic] explode all trees	717

#11	MeSH descriptor: [Mixed Connective Tissue Disease] explode all trees	13

#12	#1 OR #2 OR #3 OR #4 OR #5 OR #6 OR #7 OR #8 OR #9 OR #10 OR #11	24888

#13	MeSH descriptor: [Vaccines, Inactivated] explode all trees	1148

#14	MeSH descriptor: [Influenza Vaccines] explode all trees	1853

#15	MeSH descriptor: [Pneumococcal Vaccines] explode all trees	1178

#16	MeSH descriptor: [Herpes Zoster Vaccine] explode all trees	110

#17	MeSH descriptor: [Hepatitis B Vaccines] explode all trees	1003

#18	MeSH descriptor: [Papillomavirus Vaccines] explode all trees	549

#19	MeSH descriptor: [Poliovirus Vaccines] explode all trees	544

#20	MeSH descriptor: [Hepatitis A Vaccines] explode all trees	258

#21	MeSH descriptor: [Meningococcal Vaccines] explode all trees	438

#22	MeSH descriptor: [Diphtheria-Tetanus-Pertussis Vaccine] explode all trees	585

#23	#13 OR #14 OR #15 OR #16 OR #17 OR #18 OR #19 OR #20 OR #21 OR #22	5937

#24	MeSH descriptor: [Janus Kinase Inhibitors] explode all trees	145

#25	MeSH descriptor: [Protein Kinase Inhibitors] explode all trees	1498

#26	#24 OR #25	1498

#27	#12 AND #23 AND #26 **3**

**QUESTION 09. Is there a benefit to vaccine response by temporarily stopping methotrexate, mycophenolate, iJAKs before or after vaccination?**

**1.1 Following there are search strategy in PubMed/MEDLINE**

**P = Patients with immune-mediated rheumatic diseases**

(Rheumatic Diseases) OR (Rheumatic Disease) OR (Rheumatism) OR (Immune-mediated rheumatic diseases) OR (Arthritis, Rheumatoid) OR (Rheumatoid Arthritis) OR (Rheumatoid Nodule) OR (Rheumatoid Vasculitis) OR (Sjogren's Syndrome) OR "Spondylarthritis" [Mesh] OR (Spondylarthritis) OR (Spondylarthritides) OR (Spinal Arthritis) OR "Arthritis, Psoriatic" [Mesh] OR (Arthritis, Psoriatic) OR (Arthritic Psoriasis) OR (Psoriatic Arthritis) OR (Psoriasis Arthropathica) OR (Psoriatic Arthropathy) OR (Psoriatic Arthropathies) OR "Spondylitis, Ankylosing" [Mesh] OR (Spondylitis, Ankylosing) OR (Bechterew's Disease) OR (Bechterews Disease) OR (Marie Struempell Disease) OR (Spondylarthritis Ankylopoietica) OR (Spondyloarthritis Ankylopoietica) OR (Ankylosing Spondylitis) OR (Ankylosing Spondylarthritis) OR (Ankylosing Spondylarthritides) OR (Ankylosing Spondyloarthritis) OR (Spondylitis Ankylopoietica) OR (Bechterew Disease) OR (Rheumatoid Spondylitis) OR "Lupus Erythematosus, Systemic" [Mesh] OR (Lupus Erythematosus, Systemic) OR (Systemic Lupus Erythematosus) OR (Lupus Erythematosus Disseminatus) OR (Libman Sacks Disease) OR "Myositis" [Mesh] OR (Myositis) OR (Myositides) OR (Inflammatory Muscle Diseases) OR (Inflammatory Muscle Disease) OR (Inflammatory Myopathy) OR (Inflammatory Myopathies) OR (Proliferative Myositis) OR (Infectious Myositides) OR (Infectious Myositis) OR (Idiopathic Inflammatory Myopathies) OR (Idiopathic Inflammatory Myopathy) OR (Idiopathic Inflammatory Myositis) OR (Focal Myositis) OR "Systemic Vasculitis" [Mesh] OR (Systemic Vasculitis) OR (Systemic Vasculitides) OR "Sjogren's Syndrome" [Mesh] OR (Sjogren's Syndrome) OR (Sjogrens Syndrome) OR (Sjogren Syndrome) OR (Sicca Syndrome) OR "Scleroderma, Systemic" [Mesh] OR (Scleroderma, Systemic) OR (Systemic Sclerosis) OR (Systemic Scleroderma) OR "Mixed Connective Tissue Disease" [Mesh] OR (Mixed Connective Tissue Disease) OR (Sharp Syndrome) OR (MCTD)

**I =** stopping methotrexate, mycophenolate, iJAKs

"Methotrexate" [Mesh] OR (Methotrexate) OR (Amethopterin) OR (Mexate) OR (Methotrexate Sodium) OR (Methotrexate Hydrate) OR (Dicesium Salt Methotrexate) OR "Mycophenolic Acid" [Mesh] OR (Mycophenolic Acid) OR (Mycophenolate Mofetil) OR (Mycophenolic Acid) OR (Morpholinoethyl Ester) OR (Cellcept) OR (Mycophenolate Sodium) OR (Sodium Mycophenolat) OR (Myfortic) OR (Mycophenolate Mofetil Hydrochloride) OR (RS 61443) OR "Abatacept" [Mesh] OR (Abatacept) OR (CTLA-4-Ig) OR (Cytotoxic T Lymphocyte-Associated Antigen 4-Immunoglobulin) OR (Cytotoxic T Lymphocyte Associated Antigen 4 Immunoglobulin) OR (CTLA4-Ig) OR (CTLA4-Ig Immunoconjugate) OR (CTLA4 Ig Immunoconjugate) OR (BMS224818) OR (BMS 224818) OR (Belatacept) OR (Orencia) OR (BMS 188667) OR (LEA29Y) OR (Nulojix) OR "Janus Kinase Inhibitors" [Mesh] OR (Janus Kinase Inhibitors) OR (JAK Inhibitors) OR (Janus Kinase Inhibitor) OR (JAK Inhibitor) OR "upadacitinib" [Mesh] OR (upadacitinib) OR (ABT-494) OR (Rinvoq) OR "Protein Kinase Inhibitors" [Mesh] OR (Protein Kinase Inhibitors) OR (Protein Kinase Inhibitor) OR "tofacitinib" [Mesh] OR (tofacitinib) OR (tasocitinib) OR (tofacitinib citrate) OR (Xeljanz) OR (CP 690550) OR "baricitinib" [Mesh] OR (baricitinib) OR (INCB-28050) OR (Olumiant) OR (baricitinib phosphate) OR (baricitinib phosphate salt) OR (INCB028050) OR (LY3009104)

O - vaccines

"Vaccines, Inactivated" [Mesh] OR (Vaccines, Inactivated) OR (Inactivated Vaccines) OR (Killed Vaccine) OR (Killed Vaccines) OR (Inactivated Vaccine) OR (Influenza Vaccines) OR (Influenza Vaccine) OR (Influenzavirus Vaccines) OR (Influenza Virus Vaccines) OR (Influenzavirus Vaccine) OR (Influenza Virus Vaccine) OR (High-Dose Trivalent Influenza Vaccine) OR (High Dose Trivalent Influenza Vaccine) OR (Trivalent Influenza Vaccine) OR (Quadrivalent Influenza Vaccine) OR "Pneumococcal Vaccines" [Mesh] OR (Pneumococcal Vaccines) OR (Pneumococcal Vaccine) OR (Pneumovax) OR (Pneumococcal Polysaccharide Vaccine) OR (Pnu Imune Vaccine) OR (PnuImune Vaccine) OR (Conjugated penumococcal vaccine) OR (Herpes Zoster Vaccine) OR (Zoster Vaccine) OR (Shingles Vaccine) OR "Hepatitis B Vaccines" [Mesh] OR (Hepatitis B Vaccines) OR (Hepatitis B Vaccine) OR "Papillomavirus Vaccines" [Mesh] OR (Papillomavirus Vaccines) OR (Papillomavirus Vaccine) OR (Human Papillomavirus Vaccines) OR (HPV Vaccine) OR (Human Papilloma Virus Vaccine) OR (HPV Vaccines) OR (Human Papilloma Virus Vaccines) OR (Human Papillomavirus Vaccine) OR "Poliovirus Vaccines" [Mesh] OR (Poliovirus Vaccines) OR (Poliovirus Vaccine) OR (salk) OR "Hepatitis A Vaccines" [Mesh] OR (Hepatitis A Vaccines) OR (Hepatovirus Vaccines) OR (Hepatitis A Vaccine) OR (Vaqta) OR (Havrix) OR "Meningococcal Vaccines" [Mesh] OR (Meningococcal Vaccine) OR (Menactra) OR (Tetravalent Meningococcal Vaccine) OR (Menomune) OR (Menveo) OR (Bivalent Meningococcal Vaccine) OR (Meningococcal Polysaccharide Vaccine) OR "Diphtheria-Tetanus-Pertussis Vaccine" [Mesh] OR (Diphtheria-Tetanus-Pertussis Vaccine) OR (Diphtheria Tetanus Pertussis Vaccine) OR (DTwP Vaccine) OR (DPT Vaccine) OR (DTP Vaccine) OR (Di Te Per Vaccine) OR (Diphtheria Pertussis Tetanus Vaccine) OR (Haemophilus influenza type b polysaccharide vaccine-tetanus toxin conjugate) OR (PRP-T) OR (Hib-TT) OR (OmniHIB) OR (ActHIB)

#1 AND #2 AND #3

("rheumatic diseases"[MeSH Terms] OR ("rheumatic"[All Fields] AND "diseases"[All Fields]) OR "rheumatic diseases"[All Fields] OR ("rheumatic diseases"[MeSH Terms] OR ("rheumatic"[All Fields] AND "diseases"[All Fields]) OR "rheumatic diseases"[All Fields] OR ("rheumatic"[All Fields] AND "disease"[All Fields]) OR "rheumatic disease"[All Fields]) OR ("rheumatic diseases"[MeSH Terms] OR ("rheumatic"[All Fields] AND "diseases"[All Fields]) OR "rheumatic diseases"[All Fields] OR "rheumatics"[All Fields] OR "rheumatism"[All Fields] OR "rheumatic"[All Fields] OR "rheumatisms"[All Fields]) OR ("Immune-mediated"[All Fields] AND ("rheumatic diseases"[MeSH Terms] OR ("rheumatic"[All Fields] AND "diseases"[All Fields]) OR "rheumatic diseases"[All Fields])) OR ("arthritis, rheumatoid"[MeSH Terms] OR ("arthritis"[All Fields] AND "rheumatoid"[All Fields]) OR "rheumatoid arthritis"[All Fields] OR ("arthritis"[All Fields] AND "rheumatoid"[All Fields]) OR "arthritis rheumatoid"[All Fields]) OR ("arthritis, rheumatoid"[MeSH Terms] OR ("arthritis"[All Fields] AND "rheumatoid"[All Fields]) OR "rheumatoid arthritis"[All Fields] OR ("rheumatoid"[All Fields] AND "arthritis"[All Fields])) OR ("rheumatoid nodule"[MeSH Terms] OR ("rheumatoid"[All Fields] AND "nodule"[All Fields]) OR "rheumatoid nodule"[All Fields]) OR ("rheumatoid vasculitis"[MeSH Terms] OR ("rheumatoid"[All Fields] AND "vasculitis"[All Fields]) OR "rheumatoid vasculitis"[All Fields]) OR ("Sjogren's Syndrome"[MeSH Terms] OR ("sjogren s"[All Fields] AND "syndrome"[All Fields]) OR "Sjogren's Syndrome"[All Fields]) OR "Spondylarthritis"[MeSH Terms] OR ("Spondylarthritis"[MeSH Terms] OR "Spondylarthritis"[All Fields] OR "spondylarthritides"[All Fields]) OR ("Spondylarthritis"[MeSH Terms] OR "Spondylarthritis"[All Fields] OR "spondylarthritides"[All Fields]) OR ("Spondylarthritis"[MeSH Terms] OR "Spondylarthritis"[All Fields] OR ("spinal"[All Fields] AND "arthritis"[All Fields]) OR "spinal arthritis"[All Fields]) OR "arthritis, psoriatic"[MeSH Terms] OR ("arthritis, psoriatic"[MeSH Terms] OR ("arthritis"[All Fields] AND "psoriatic"[All Fields]) OR "psoriatic arthritis"[All Fields] OR ("arthritis"[All Fields] AND "psoriatic"[All Fields]) OR "arthritis psoriatic"[All Fields]) OR ("arthritis, psoriatic"[MeSH Terms] OR ("arthritis"[All Fields] AND "psoriatic"[All Fields]) OR "psoriatic arthritis"[All Fields] OR ("arthritic"[All Fields] AND "psoriasis"[All Fields]) OR "arthritic psoriasis"[All Fields]) OR ("arthritis, psoriatic"[MeSH Terms] OR ("arthritis"[All Fields] AND "psoriatic"[All Fields]) OR "psoriatic arthritis"[All Fields] OR ("psoriatic"[All Fields] AND "arthritis"[All Fields])) OR ("arthritis, psoriatic"[MeSH Terms] OR ("arthritis"[All Fields] AND "psoriatic"[All Fields]) OR "psoriatic arthritis"[All Fields] OR ("psoriasis"[All Fields] AND "arthropathica"[All Fields]) OR "psoriasis arthropathica"[All Fields]) OR ("arthritis, psoriatic"[MeSH Terms] OR ("arthritis"[All Fields] AND "psoriatic"[All Fields]) OR "psoriatic arthritis"[All Fields] OR ("psoriatic"[All Fields] AND "arthropathy"[All Fields]) OR "psoriatic arthropathy"[All Fields]) OR ("arthritis, psoriatic"[MeSH Terms] OR ("arthritis"[All Fields] AND "psoriatic"[All Fields]) OR "psoriatic arthritis"[All Fields] OR ("psoriatic"[All Fields] AND "arthropathies"[All Fields]) OR "psoriatic arthropathies"[All Fields]) OR "spondylitis, ankylosing"[MeSH Terms] OR ("spondylitis, ankylosing"[MeSH Terms] OR ("spondylitis"[All Fields] AND "ankylosing"[All Fields]) OR "ankylosing spondylitis"[All Fields] OR ("spondylitis"[All Fields] AND "ankylosing"[All Fields]) OR "spondylitis ankylosing"[All Fields]) OR ("spondylitis, ankylosing"[MeSH Terms] OR ("spondylitis"[All Fields] AND "ankylosing"[All Fields]) OR "ankylosing spondylitis"[All Fields] OR ("bechterew s"[All Fields] AND "disease"[All Fields]) OR "bechterew s disease"[All Fields]) OR ("spondylitis, ankylosing"[MeSH Terms] OR ("spondylitis"[All Fields] AND "ankylosing"[All Fields]) OR "ankylosing spondylitis"[All Fields] OR ("bechterews"[All Fields] AND "disease"[All Fields]) OR "bechterews disease"[All Fields]) OR ("spondylitis, ankylosing"[MeSH Terms] OR ("spondylitis"[All Fields] AND "ankylosing"[All Fields]) OR "ankylosing spondylitis"[All Fields] OR ("marie"[All Fields] AND "struempell"[All Fields] AND "disease"[All Fields]) OR "marie struempell disease"[All Fields]) OR ("spondylitis, ankylosing"[MeSH Terms] OR ("spondylitis"[All Fields] AND "ankylosing"[All Fields]) OR "ankylosing spondylitis"[All Fields] OR ("Spondylarthritis"[All Fields] AND "ankylopoietica"[All Fields]) OR "spondylarthritis ankylopoietica"[All Fields]) OR ("spondylitis, ankylosing"[MeSH Terms] OR ("spondylitis"[All Fields] AND "ankylosing"[All Fields]) OR "ankylosing spondylitis"[All Fields] OR ("spondyloarthritis"[All Fields] AND "ankylopoietica"[All Fields]) OR "spondyloarthritis ankylopoietica"[All Fields]) OR ("spondylitis, ankylosing"[MeSH Terms] OR ("spondylitis"[All Fields] AND "ankylosing"[All Fields]) OR "ankylosing spondylitis"[All Fields] OR ("ankylosing"[All Fields] AND "spondylitis"[All Fields])) OR ("spondylitis, ankylosing"[MeSH Terms] OR ("spondylitis"[All Fields] AND "ankylosing"[All Fields]) OR "ankylosing spondylitis"[All Fields] OR ("ankylosing"[All Fields] AND "Spondylarthritis"[All Fields]) OR "ankylosing spondylarthritis"[All Fields]) OR ("spondylitis, ankylosing"[MeSH Terms] OR ("spondylitis"[All Fields] AND "ankylosing"[All Fields]) OR "ankylosing spondylitis"[All Fields] OR ("ankylosing"[All Fields] AND "spondylarthritides"[All Fields]) OR "ankylosing spondylarthritides"[All Fields]) OR ("spondylitis, ankylosing"[MeSH Terms] OR ("spondylitis"[All Fields] AND "ankylosing"[All Fields]) OR "ankylosing spondylitis"[All Fields] OR ("ankylosing"[All Fields] AND "spondyloarthritis"[All Fields]) OR "ankylosing spondyloarthritis"[All Fields]) OR ("spondylitis, ankylosing"[MeSH Terms] OR ("spondylitis"[All Fields] AND "ankylosing"[All Fields]) OR "ankylosing spondylitis"[All Fields] OR ("spondylitis"[All Fields] AND "ankylopoietica"[All Fields]) OR "spondylitis ankylopoietica"[All Fields]) OR ("spondylitis, ankylosing"[MeSH Terms] OR ("spondylitis"[All Fields] AND "ankylosing"[All Fields]) OR "ankylosing spondylitis"[All Fields] OR ("bechterew"[All Fields] AND "disease"[All Fields]) OR "bechterew disease"[All Fields]) OR ("spondylitis, ankylosing"[MeSH Terms] OR ("spondylitis"[All Fields] AND "ankylosing"[All Fields]) OR "ankylosing spondylitis"[All Fields] OR ("rheumatoid"[All Fields] AND "spondylitis"[All Fields]) OR "rheumatoid spondylitis"[All Fields]) OR "lupus erythematosus, systemic"[MeSH Terms] OR ("lupus erythematosus, systemic"[MeSH Terms] OR ("lupus"[All Fields] AND "erythematosus"[All Fields] AND "systemic"[All Fields]) OR "systemic lupus erythematosus"[All Fields] OR ("lupus"[All Fields] AND "erythematosus"[All Fields] AND "systemic"[All Fields]) OR "lupus erythematosus systemic"[All Fields]) OR ("lupus erythematosus, systemic"[MeSH Terms] OR ("lupus"[All Fields] AND "erythematosus"[All Fields] AND "systemic"[All Fields]) OR "systemic lupus erythematosus"[All Fields] OR ("systemic"[All Fields] AND "lupus"[All Fields] AND "erythematosus"[All Fields])) OR ("lupus erythematosus, systemic"[MeSH Terms] OR ("lupus"[All Fields] AND "erythematosus"[All Fields] AND "systemic"[All Fields]) OR "systemic lupus erythematosus"[All Fields] OR ("lupus"[All Fields] AND "erythematosus"[All Fields] AND "disseminatus"[All Fields]) OR "lupus erythematosus disseminatus"[All Fields]) OR ("lupus erythematosus, systemic"[MeSH Terms] OR ("lupus"[All Fields] AND "erythematosus"[All Fields] AND "systemic"[All Fields]) OR "systemic lupus erythematosus"[All Fields] OR ("libman"[All Fields] AND "sacks"[All Fields] AND "disease"[All Fields]) OR "libman sacks disease"[All Fields]) OR "Myositis"[MeSH Terms] OR ("Myositis"[MeSH Terms] OR "Myositis"[All Fields] OR "myositides"[All Fields]) OR ("Myositis"[MeSH Terms] OR "Myositis"[All Fields] OR "myositides"[All Fields]) OR ("Myositis"[MeSH Terms] OR "Myositis"[All Fields] OR ("inflammatory"[All Fields] AND "muscle"[All Fields] AND "diseases"[All Fields]) OR "inflammatory muscle diseases"[All Fields]) OR ("Myositis"[MeSH Terms] OR "Myositis"[All Fields] OR ("inflammatory"[All Fields] AND "muscle"[All Fields] AND "disease"[All Fields]) OR "inflammatory muscle disease"[All Fields]) OR ("Myositis"[MeSH Terms] OR "Myositis"[All Fields] OR ("inflammatory"[All Fields] AND "myopathy"[All Fields]) OR "inflammatory myopathy"[All Fields]) OR ("Myositis"[MeSH Terms] OR "Myositis"[All Fields] OR ("inflammatory"[All Fields] AND "myopathies"[All Fields]) OR "inflammatory myopathies"[All Fields]) OR ("Myositis"[MeSH Terms] OR "Myositis"[All Fields] OR ("proliferative"[All Fields] AND "Myositis"[All Fields]) OR "proliferative myositis"[All Fields]) OR ("Myositis"[MeSH Terms] OR "Myositis"[All Fields] OR ("infectious"[All Fields] AND "myositides"[All Fields]) OR "infectious myositides"[All Fields]) OR ("Myositis"[MeSH Terms] OR "Myositis"[All Fields] OR ("infectious"[All Fields] AND "Myositis"[All Fields]) OR "infectious myositis"[All Fields]) OR ("Myositis"[MeSH Terms] OR "Myositis"[All Fields] OR ("idiopathic"[All Fields] AND "inflammatory"[All Fields] AND "myopathies"[All Fields]) OR "idiopathic inflammatory myopathies"[All Fields]) OR ("Myositis"[MeSH Terms] OR "Myositis"[All Fields] OR ("idiopathic"[All Fields] AND "inflammatory"[All Fields] AND "myopathy"[All Fields]) OR "idiopathic inflammatory myopathy"[All Fields]) OR ("Myositis"[MeSH Terms] OR "Myositis"[All Fields] OR ("idiopathic"[All Fields] AND "inflammatory"[All Fields] AND "Myositis"[All Fields]) OR "idiopathic inflammatory myositis"[All Fields]) OR ("Myositis"[MeSH Terms] OR "Myositis"[All Fields] OR ("focal"[All Fields] AND "Myositis"[All Fields]) OR "focal myositis"[All Fields]) OR "Systemic Vasculitis"[MeSH Terms] OR ("Systemic Vasculitis"[MeSH Terms] OR ("systemic"[All Fields] AND "vasculitis"[All Fields]) OR "Systemic Vasculitis"[All Fields]) OR ("Systemic Vasculitis"[MeSH Terms] OR ("systemic"[All Fields] AND "vasculitis"[All Fields]) OR "Systemic Vasculitis"[All Fields] OR ("systemic"[All Fields] AND "vasculitides"[All Fields]) OR "systemic vasculitides"[All Fields]) OR "Sjogren's Syndrome"[MeSH Terms] OR ("Sjogren's Syndrome"[MeSH Terms] OR ("sjogren s"[All Fields] AND "syndrome"[All Fields]) OR "Sjogren's Syndrome"[All Fields]) OR ("Sjogren's Syndrome"[MeSH Terms] OR ("sjogren s"[All Fields] AND "syndrome"[All Fields]) OR "Sjogren's Syndrome"[All Fields] OR ("sjogrens"[All Fields] AND "syndrome"[All Fields]) OR "sjogrens syndrome"[All Fields]) OR ("Sjogren's Syndrome"[MeSH Terms] OR ("sjogren s"[All Fields] AND "syndrome"[All Fields]) OR "Sjogren's Syndrome"[All Fields] OR ("sjogren"[All Fields] AND "syndrome"[All Fields]) OR "sjogren syndrome"[All Fields]) OR ("Sjogren's Syndrome"[MeSH Terms] OR ("sjogren s"[All Fields] AND "syndrome"[All Fields]) OR "Sjogren's Syndrome"[All Fields] OR ("sicca"[All Fields] AND "syndrome"[All Fields]) OR "sicca syndrome"[All Fields]) OR "scleroderma, systemic"[MeSH Terms] OR ("scleroderma, systemic"[MeSH Terms] OR ("scleroderma"[All Fields] AND "systemic"[All Fields]) OR "systemic scleroderma"[All Fields] OR ("scleroderma"[All Fields] AND "systemic"[All Fields]) OR "scleroderma systemic"[All Fields]) OR ("scleroderma, systemic"[MeSH Terms] OR ("scleroderma"[All Fields] AND "systemic"[All Fields]) OR "systemic scleroderma"[All Fields] OR ("systemic"[All Fields] AND "sclerosis"[All Fields]) OR "systemic sclerosis"[All Fields]) OR ("scleroderma, systemic"[MeSH Terms] OR ("scleroderma"[All Fields] AND "systemic"[All Fields]) OR "systemic scleroderma"[All Fields] OR ("systemic"[All Fields] AND "scleroderma"[All Fields])) OR "Mixed Connective Tissue Disease"[MeSH Terms] OR ("Mixed Connective Tissue Disease"[MeSH Terms] OR ("mixed"[All Fields] AND "connective"[All Fields] AND "tissue"[All Fields] AND "disease"[All Fields]) OR "Mixed Connective Tissue Disease"[All Fields]) OR ("Mixed Connective Tissue Disease"[MeSH Terms] OR ("mixed"[All Fields] AND "connective"[All Fields] AND "tissue"[All Fields] AND "disease"[All Fields]) OR "Mixed Connective Tissue Disease"[All Fields] OR ("sharp"[All Fields] AND "syndrome"[All Fields]) OR "sharp syndrome"[All Fields]) OR ("Mixed Connective Tissue Disease"[MeSH Terms] OR ("mixed"[All Fields] AND "connective"[All Fields] AND "tissue"[All Fields] AND "disease"[All Fields]) OR "Mixed Connective Tissue Disease"[All Fields] OR "mctd"[All Fields])) AND ("Methotrexate"[MeSH Terms] OR ("Methotrexate"[MeSH Terms] OR "Methotrexate"[All Fields] OR "methotrexate s"[All Fields] OR "methotrexates"[All Fields]) OR ("Methotrexate"[MeSH Terms] OR "Methotrexate"[All Fields] OR "amethopterin"[All Fields]) OR ("Methotrexate"[MeSH Terms] OR "Methotrexate"[All Fields] OR "mexate"[All Fields]) OR ("Methotrexate"[MeSH Terms] OR "Methotrexate"[All Fields] OR ("Methotrexate"[All Fields] AND "sodium"[All Fields]) OR "methotrexate sodium"[All Fields]) OR ("Methotrexate"[MeSH Terms] OR "Methotrexate"[All Fields] OR ("Methotrexate"[All Fields] AND "hydrate"[All Fields]) OR "methotrexate hydrate"[All Fields]) OR ("Methotrexate"[MeSH Terms] OR "Methotrexate"[All Fields] OR ("dicesium"[All Fields] AND "salt"[All Fields] AND "Methotrexate"[All Fields])) OR "Mycophenolic Acid"[MeSH Terms] OR ("Mycophenolic Acid"[MeSH Terms] OR ("mycophenolic"[All Fields] AND "acid"[All Fields]) OR "Mycophenolic Acid"[All Fields]) OR ("Mycophenolic Acid"[MeSH Terms] OR ("mycophenolic"[All Fields] AND "acid"[All Fields]) OR "Mycophenolic Acid"[All Fields] OR ("mycophenolate"[All Fields] AND "mofetil"[All Fields]) OR "mycophenolate mofetil"[All Fields]) OR ("Mycophenolic Acid"[MeSH Terms] OR ("mycophenolic"[All Fields] AND "acid"[All Fields]) OR "Mycophenolic Acid"[All Fields]) OR ("Morpholinoethyl"[All Fields] AND ("esteric"[All Fields] OR "esterized"[All Fields] OR "esters"[MeSH Terms] OR "esters"[All Fields] OR "ester"[All Fields])) OR ("Mycophenolic Acid"[MeSH Terms] OR ("mycophenolic"[All Fields] AND "acid"[All Fields]) OR "Mycophenolic Acid"[All Fields] OR "cellcept"[All Fields] OR ("mycophenolate"[All Fields] AND "mofetil"[All Fields]) OR "mycophenolate mofetil"[All Fields]) OR ("Mycophenolic Acid"[MeSH Terms] OR ("mycophenolic"[All Fields] AND "acid"[All Fields]) OR "Mycophenolic Acid"[All Fields] OR ("mycophenolate"[All Fields] AND "sodium"[All Fields]) OR "mycophenolate sodium"[All Fields]) OR (("sodium"[MeSH Terms] OR "sodium"[All Fields] OR "sodiums"[All Fields]) AND "Mycophenolat"[All Fields]) OR ("Mycophenolic Acid"[MeSH Terms] OR ("mycophenolic"[All Fields] AND "acid"[All Fields]) OR "Mycophenolic Acid"[All Fields] OR "myfortic"[All Fields]) OR ("Mycophenolic Acid"[MeSH Terms] OR ("mycophenolic"[All Fields] AND "acid"[All Fields]) OR "Mycophenolic Acid"[All Fields] OR ("mycophenolate"[All Fields] AND "mofetil"[All Fields] AND "hydrochloride"[All Fields]) OR "mycophenolate mofetil hydrochloride"[All Fields]) OR ("Mycophenolic Acid"[MeSH Terms] OR ("mycophenolic"[All Fields] AND "acid"[All Fields]) OR "Mycophenolic Acid"[All Fields] OR "rs 61443"[All Fields]) OR "Abatacept"[MeSH Terms] OR ("Abatacept"[MeSH Terms] OR "Abatacept"[All Fields]) OR ("Abatacept"[MeSH Terms] OR "Abatacept"[All Fields] OR "ctla 4 ig"[All Fields]) OR ("Abatacept"[MeSH Terms] OR "Abatacept"[All Fields] OR "cytotoxic t lymphocyte associated antigen 4 immunoglobulin"[All Fields]) OR ("Abatacept"[MeSH Terms] OR "Abatacept"[All Fields] OR "cytotoxic t lymphocyte associated antigen 4 immunoglobulin"[All Fields]) OR ("Abatacept"[MeSH Terms] OR "Abatacept"[All Fields] OR ("ctla4"[All Fields] AND "ig"[All Fields]) OR "ctla4 ig"[All Fields]) OR ("Abatacept"[MeSH Terms] OR "Abatacept"[All Fields] OR ("ctla4"[All Fields] AND "ig"[All Fields] AND "immunoconjugate"[All Fields]) OR "ctla4 ig immunoconjugate"[All Fields]) OR ("Abatacept"[MeSH Terms] OR "Abatacept"[All Fields] OR ("ctla4"[All Fields] AND "ig"[All Fields] AND "immunoconjugate"[All Fields]) OR "ctla4 ig immunoconjugate"[All Fields]) OR ("Abatacept"[MeSH Terms] OR "Abatacept"[All Fields] OR "bms224818"[All Fields]) OR ("Abatacept"[MeSH Terms] OR "Abatacept"[All Fields] OR "bms 224818"[All Fields]) OR ("Abatacept"[MeSH Terms] OR "Abatacept"[All Fields] OR "belatacept"[All Fields]) OR ("Abatacept"[MeSH Terms] OR "Abatacept"[All Fields] OR "orencia"[All Fields]) OR ("Abatacept"[MeSH Terms] OR "Abatacept"[All Fields] OR "bms 188667"[All Fields]) OR ("Abatacept"[MeSH Terms] OR "Abatacept"[All Fields] OR "lea29y"[All Fields]) OR ("Abatacept"[MeSH Terms] OR "Abatacept"[All Fields] OR "belatacept"[All Fields] OR "nulojix"[All Fields]) OR "Janus Kinase Inhibitors"[MeSH Terms] OR ("Janus Kinase Inhibitors"[Pharmacological Action] OR "Janus Kinase Inhibitors"[MeSH Terms] OR ("janus"[All Fields] AND "kinase"[All Fields] AND "inhibitors"[All Fields]) OR "Janus Kinase Inhibitors"[All Fields]) OR ("Janus Kinase Inhibitors"[Pharmacological Action] OR "Janus Kinase Inhibitors"[MeSH Terms] OR ("janus"[All Fields] AND "kinase"[All Fields] AND "inhibitors"[All Fields]) OR "Janus Kinase Inhibitors"[All Fields] OR ("jak"[All Fields] AND "inhibitors"[All Fields]) OR "jak inhibitors"[All Fields]) OR ("Janus Kinase Inhibitors"[Pharmacological Action] OR "Janus Kinase Inhibitors"[MeSH Terms] OR ("janus"[All Fields] AND "kinase"[All Fields] AND "inhibitors"[All Fields]) OR "Janus Kinase Inhibitors"[All Fields] OR ("janus"[All Fields] AND "kinase"[All Fields] AND "inhibitor"[All Fields]) OR "janus kinase inhibitor"[All Fields]) OR ("Janus Kinase Inhibitors"[Pharmacological Action] OR "Janus Kinase Inhibitors"[MeSH Terms] OR ("janus"[All Fields] AND "kinase"[All Fields] AND "inhibitors"[All Fields]) OR "Janus Kinase Inhibitors"[All Fields] OR ("jak"[All Fields] AND "inhibitor"[All Fields]) OR "jak inhibitor"[All Fields]) OR ("upadacitinib"[Supplementary Concept] OR "upadacitinib"[All Fields]) OR ("upadacitinib"[Supplementary Concept] OR "upadacitinib"[All Fields] OR "abt 494"[All Fields]) OR ("upadacitinib"[Supplementary Concept] OR "upadacitinib"[All Fields] OR "rinvoq"[All Fields]) OR "Protein Kinase Inhibitors"[MeSH Terms] OR ("Protein Kinase Inhibitors"[Pharmacological Action] OR "Protein Kinase Inhibitors"[MeSH Terms] OR ("protein"[All Fields] AND "kinase"[All Fields] AND "inhibitors"[All Fields]) OR "Protein Kinase Inhibitors"[All Fields]) OR ("Protein Kinase Inhibitors"[Pharmacological Action] OR "Protein Kinase Inhibitors"[MeSH Terms] OR ("protein"[All Fields] AND "kinase"[All Fields] AND "inhibitors"[All Fields]) OR "Protein Kinase Inhibitors"[All Fields] OR ("protein"[All Fields] AND "kinase"[All Fields] AND "inhibitor"[All Fields]) OR "protein kinase inhibitor"[All Fields]) OR ("tofacitinib"[Supplementary Concept] OR "tofacitinib"[All Fields] OR "tofacitinib s"[All Fields]) OR ("tofacitinib"[Supplementary Concept] OR "tofacitinib"[All Fields] OR "tasocitinib"[All Fields]) OR ("tofacitinib"[Supplementary Concept] OR "tofacitinib"[All Fields] OR "tofacitinib citrate"[All Fields]) OR ("tofacitinib"[Supplementary Concept] OR "tofacitinib"[All Fields] OR "xeljanz"[All Fields] OR "tofacitinib s"[All Fields]) OR ("tofacitinib"[Supplementary Concept] OR "tofacitinib"[All Fields] OR "cp 690550"[All Fields]) OR ("baricitinib"[Supplementary Concept] OR "baricitinib"[All Fields]) OR ("baricitinib"[Supplementary Concept] OR "baricitinib"[All Fields]) OR ("baricitinib"[Supplementary Concept] OR "baricitinib"[All Fields] OR "olumiant"[All Fields]) OR ("baricitinib"[Supplementary Concept] OR "baricitinib"[All Fields]) OR ("baricitinib"[Supplementary Concept] OR "baricitinib"[All Fields]) OR ("baricitinib"[Supplementary Concept] OR "baricitinib"[All Fields] OR "incb028050"[All Fields]) OR ("baricitinib"[Supplementary Concept] OR "baricitinib"[All Fields] OR "ly3009104"[All Fields])) AND ("vaccines, inactivated"[MeSH Terms] OR ("vaccines, inactivated"[MeSH Terms] OR ("vaccines"[All Fields] AND "inactivated"[All Fields]) OR "inactivated vaccines"[All Fields] OR ("vaccines"[All Fields] AND "inactivated"[All Fields]) OR "vaccines inactivated"[All Fields]) OR ("vaccines, inactivated"[MeSH Terms] OR ("vaccines"[All Fields] AND "inactivated"[All Fields]) OR "inactivated vaccines"[All Fields] OR ("inactivated"[All Fields] AND "vaccines"[All Fields])) OR ("vaccines, inactivated"[MeSH Terms] OR ("vaccines"[All Fields] AND "inactivated"[All Fields]) OR "inactivated vaccines"[All Fields] OR ("killed"[All Fields] AND "vaccine"[All Fields]) OR "killed vaccine"[All Fields]) OR ("vaccines, inactivated"[MeSH Terms] OR ("vaccines"[All Fields] AND "inactivated"[All Fields]) OR "inactivated vaccines"[All Fields] OR ("killed"[All Fields] AND "vaccines"[All Fields]) OR "killed vaccines"[All Fields]) OR ("vaccines, inactivated"[MeSH Terms] OR ("vaccines"[All Fields] AND "inactivated"[All Fields]) OR "inactivated vaccines"[All Fields] OR ("inactivated"[All Fields] AND "vaccine"[All Fields]) OR "inactivated vaccine"[All Fields]) OR ("influenza vaccines"[MeSH Terms] OR ("influenza"[All Fields] AND "vaccines"[All Fields]) OR "influenza vaccines"[All Fields]) OR ("influenza vaccines"[MeSH Terms] OR ("influenza"[All Fields] AND "vaccines"[All Fields]) OR "influenza vaccines"[All Fields] OR ("influenza"[All Fields] AND "vaccine"[All Fields]) OR "influenza vaccine"[All Fields]) OR ("influenza vaccines"[MeSH Terms] OR ("influenza"[All Fields] AND "vaccines"[All Fields]) OR "influenza vaccines"[All Fields] OR ("influenzavirus"[All Fields] AND "vaccines"[All Fields])) OR ("influenza vaccines"[MeSH Terms] OR ("influenza"[All Fields] AND "vaccines"[All Fields]) OR "influenza vaccines"[All Fields] OR ("influenza"[All Fields] AND "virus"[All Fields] AND "vaccines"[All Fields]) OR "influenza virus vaccines"[All Fields]) OR ("influenza vaccines"[MeSH Terms] OR ("influenza"[All Fields] AND "vaccines"[All Fields]) OR "influenza vaccines"[All Fields] OR ("influenzavirus"[All Fields] AND "vaccine"[All Fields]) OR "influenzavirus vaccine"[All Fields]) OR ("influenza vaccines"[MeSH Terms] OR ("influenza"[All Fields] AND "vaccines"[All Fields]) OR "influenza vaccines"[All Fields] OR ("influenza"[All Fields] AND "virus"[All Fields] AND "vaccine"[All Fields]) OR "influenza virus vaccine"[All Fields]) OR ("influenza vaccines"[MeSH Terms] OR ("influenza"[All Fields] AND "vaccines"[All Fields]) OR "influenza vaccines"[All Fields] OR ("high"[All Fields] AND "dose"[All Fields] AND "trivalent"[All Fields] AND "influenza"[All Fields] AND "vaccine"[All Fields]) OR "high dose trivalent influenza vaccine"[All Fields]) OR ("influenza vaccines"[MeSH Terms] OR ("influenza"[All Fields] AND "vaccines"[All Fields]) OR "influenza vaccines"[All Fields] OR ("high"[All Fields] AND "dose"[All Fields] AND "trivalent"[All Fields] AND "influenza"[All Fields] AND "vaccine"[All Fields]) OR "high dose trivalent influenza vaccine"[All Fields]) OR ("influenza vaccines"[MeSH Terms] OR ("influenza"[All Fields] AND "vaccines"[All Fields]) OR "influenza vaccines"[All Fields] OR ("trivalent"[All Fields] AND "influenza"[All Fields] AND "vaccine"[All Fields]) OR "trivalent influenza vaccine"[All Fields]) OR ("influenza vaccines"[MeSH Terms] OR ("influenza"[All Fields] AND "vaccines"[All Fields]) OR "influenza vaccines"[All Fields] OR ("quadrivalent"[All Fields] AND "influenza"[All Fields] AND "vaccine"[All Fields]) OR "quadrivalent influenza vaccine"[All Fields]) OR "Pneumococcal Vaccines"[MeSH Terms] OR ("Pneumococcal Vaccines"[MeSH Terms] OR ("pneumococcal"[All Fields] AND "vaccines"[All Fields]) OR "Pneumococcal Vaccines"[All Fields]) OR ("Pneumococcal Vaccines"[MeSH Terms] OR ("pneumococcal"[All Fields] AND "vaccines"[All Fields]) OR "Pneumococcal Vaccines"[All Fields] OR ("pneumococcal"[All Fields] AND "vaccine"[All Fields]) OR "pneumococcal vaccine"[All Fields]) OR ("Pneumococcal Vaccines"[MeSH Terms] OR ("pneumococcal"[All Fields] AND "vaccines"[All Fields]) OR "Pneumococcal Vaccines"[All Fields] OR "pneumovax"[All Fields]) OR ("Pneumococcal Vaccines"[MeSH Terms] OR ("pneumococcal"[All Fields] AND "vaccines"[All Fields]) OR "Pneumococcal Vaccines"[All Fields] OR ("pneumococcal"[All Fields] AND "polysaccharide"[All Fields] AND "vaccine"[All Fields]) OR "pneumococcal polysaccharide vaccine"[All Fields]) OR ("Pneumococcal Vaccines"[MeSH Terms] OR ("pneumococcal"[All Fields] AND "vaccines"[All Fields]) OR "Pneumococcal Vaccines"[All Fields] OR ("pnu"[All Fields] AND "imune"[All Fields] AND "vaccine"[All Fields]) OR "pnu imune vaccine"[All Fields]) OR ("Pneumococcal Vaccines"[MeSH Terms] OR ("pneumococcal"[All Fields] AND "vaccines"[All Fields]) OR "Pneumococcal Vaccines"[All Fields] OR ("pnuimune"[All Fields] AND "vaccine"[All Fields])) OR (("conjugable"[All Fields] OR "conjugal"[All Fields] OR "conjugally"[All Fields] OR "conjugant"[All Fields] OR "conjugants"[All Fields] OR "conjugate"[All Fields] OR "conjugate s"[All Fields] OR "conjugated"[All Fields] OR "conjugates"[All Fields] OR "conjugating"[All Fields] OR "conjugation"[All Fields] OR "conjugational"[All Fields] OR "conjugations"[All Fields] OR "conjugative"[All Fields] OR "conjugatively"[All Fields]) AND "penumococcal"[All Fields] AND ("vaccin"[Supplementary Concept] OR "vaccin"[All Fields] OR "vaccination"[MeSH Terms] OR "vaccination"[All Fields] OR "vaccinable"[All Fields] OR "vaccinal"[All Fields] OR "vaccinate"[All Fields] OR "vaccinated"[All Fields] OR "vaccinates"[All Fields] OR "vaccinating"[All Fields] OR "vaccinations"[All Fields] OR "vaccination s"[All Fields] OR "vaccinator"[All Fields] OR "vaccinators"[All Fields] OR "vaccine s"[All Fields] OR "vaccined"[All Fields] OR "vaccines"[MeSH Terms] OR "vaccines"[All Fields] OR "vaccine"[All Fields] OR "vaccins"[All Fields])) OR ("herpes zoster vaccine"[MeSH Terms] OR ("herpes"[All Fields] AND "zoster"[All Fields] AND "vaccine"[All Fields]) OR "herpes zoster vaccine"[All Fields]) OR ("herpes zoster vaccine"[MeSH Terms] OR ("herpes"[All Fields] AND "zoster"[All Fields] AND "vaccine"[All Fields]) OR "herpes zoster vaccine"[All Fields] OR ("zoster"[All Fields] AND "vaccine"[All Fields]) OR "zoster vaccine"[All Fields]) OR ("herpes zoster vaccine"[MeSH Terms] OR ("herpes"[All Fields] AND "zoster"[All Fields] AND "vaccine"[All Fields]) OR "herpes zoster vaccine"[All Fields] OR ("shingles"[All Fields] AND "vaccine"[All Fields]) OR "shingles vaccine"[All Fields]) OR "Hepatitis B Vaccines"[MeSH Terms] OR ("Hepatitis B Vaccines"[MeSH Terms] OR "Hepatitis B Vaccines"[All Fields]) OR ("Hepatitis B Vaccines"[MeSH Terms] OR "Hepatitis B Vaccines"[All Fields] OR "hepatitis b vaccine"[All Fields]) OR "Papillomavirus Vaccines"[MeSH Terms] OR ("Papillomavirus Vaccines"[MeSH Terms] OR ("papillomavirus"[All Fields] AND "vaccines"[All Fields]) OR "Papillomavirus Vaccines"[All Fields]) OR ("Papillomavirus Vaccines"[MeSH Terms] OR ("papillomavirus"[All Fields] AND "vaccines"[All Fields]) OR "Papillomavirus Vaccines"[All Fields] OR ("papillomavirus"[All Fields] AND "vaccine"[All Fields]) OR "papillomavirus vaccine"[All Fields]) OR ("Papillomavirus Vaccines"[MeSH Terms] OR ("papillomavirus"[All Fields] AND "vaccines"[All Fields]) OR "Papillomavirus Vaccines"[All Fields] OR ("human"[All Fields] AND "papillomavirus"[All Fields] AND "vaccines"[All Fields]) OR "human papillomavirus vaccines"[All Fields]) OR ("Papillomavirus Vaccines"[MeSH Terms] OR ("papillomavirus"[All Fields] AND "vaccines"[All Fields]) OR "Papillomavirus Vaccines"[All Fields] OR ("hpv"[All Fields] AND "vaccine"[All Fields]) OR "hpv vaccine"[All Fields]) OR ("Papillomavirus Vaccines"[MeSH Terms] OR ("papillomavirus"[All Fields] AND "vaccines"[All Fields]) OR "Papillomavirus Vaccines"[All Fields] OR ("human"[All Fields] AND "papilloma"[All Fields] AND "virus"[All Fields] AND "vaccine"[All Fields]) OR "human papilloma virus vaccine"[All Fields]) OR ("Papillomavirus Vaccines"[MeSH Terms] OR ("papillomavirus"[All Fields] AND "vaccines"[All Fields]) OR "Papillomavirus Vaccines"[All Fields] OR ("hpv"[All Fields] AND "vaccines"[All Fields]) OR "hpv vaccines"[All Fields]) OR ("Papillomavirus Vaccines"[MeSH Terms] OR ("papillomavirus"[All Fields] AND "vaccines"[All Fields]) OR "Papillomavirus Vaccines"[All Fields] OR ("human"[All Fields] AND "papilloma"[All Fields] AND "virus"[All Fields] AND "vaccines"[All Fields]) OR "human papilloma virus vaccines"[All Fields]) OR ("Papillomavirus Vaccines"[MeSH Terms] OR ("papillomavirus"[All Fields] AND "vaccines"[All Fields]) OR "Papillomavirus Vaccines"[All Fields] OR ("human"[All Fields] AND "papillomavirus"[All Fields] AND "vaccine"[All Fields]) OR "human papillomavirus vaccine"[All Fields]) OR "Poliovirus Vaccines"[MeSH Terms] OR ("Poliovirus Vaccines"[MeSH Terms] OR ("poliovirus"[All Fields] AND "vaccines"[All Fields]) OR "Poliovirus Vaccines"[All Fields]) OR ("Poliovirus Vaccines"[MeSH Terms] OR ("poliovirus"[All Fields] AND "vaccines"[All Fields]) OR "Poliovirus Vaccines"[All Fields] OR ("poliovirus"[All Fields] AND "vaccine"[All Fields]) OR "poliovirus vaccine"[All Fields]) OR "salk"[All Fields] OR "Hepatitis A Vaccines"[MeSH Terms] OR ("Hepatitis A Vaccines"[MeSH Terms] OR "Hepatitis A Vaccines"[All Fields]) OR ("Hepatitis A Vaccines"[MeSH Terms] OR "Hepatitis A Vaccines"[All Fields] OR ("hepatovirus"[All Fields] AND "vaccines"[All Fields])) OR ("Hepatitis A Vaccines"[MeSH Terms] OR "Hepatitis A Vaccines"[All Fields] OR "hepatitis a vaccine"[All Fields]) OR ("hepatitis a vaccine inactivated"[All Fields] OR "Hepatitis A Vaccines"[MeSH Terms] OR "Hepatitis A Vaccines"[All Fields] OR "vaqta"[All Fields]) OR ("Hepatitis A Vaccines"[MeSH Terms] OR "Hepatitis A Vaccines"[All Fields] OR "havrix"[All Fields]) OR "Meningococcal Vaccines"[MeSH Terms] OR ("Meningococcal Vaccines"[MeSH Terms] OR ("meningococcal"[All Fields] AND "vaccines"[All Fields]) OR "Meningococcal Vaccines"[All Fields] OR ("meningococcal"[All Fields] AND "vaccine"[All Fields]) OR "meningococcal vaccine"[All Fields]) OR ("Meningococcal Vaccines"[MeSH Terms] OR ("meningococcal"[All Fields] AND "vaccines"[All Fields]) OR "Meningococcal Vaccines"[All Fields] OR "menactra"[All Fields]) OR ("Meningococcal Vaccines"[MeSH Terms] OR ("meningococcal"[All Fields] AND "vaccines"[All Fields]) OR "Meningococcal Vaccines"[All Fields] OR ("tetravalent"[All Fields] AND "meningococcal"[All Fields] AND "vaccine"[All Fields]) OR "tetravalent meningococcal vaccine"[All Fields]) OR ("Meningococcal Vaccines"[MeSH Terms] OR ("meningococcal"[All Fields] AND "vaccines"[All Fields]) OR "Meningococcal Vaccines"[All Fields] OR "menomune"[All Fields]) OR ("Meningococcal Vaccines"[MeSH Terms] OR ("meningococcal"[All Fields] AND "vaccines"[All Fields]) OR "Meningococcal Vaccines"[All Fields] OR "menveo"[All Fields]) OR ("Meningococcal Vaccines"[MeSH Terms] OR ("meningococcal"[All Fields] AND "vaccines"[All Fields]) OR "Meningococcal Vaccines"[All Fields] OR ("bivalent"[All Fields] AND "meningococcal"[All Fields] AND "vaccine"[All Fields]) OR "bivalent meningococcal vaccine"[All Fields]) OR ("Meningococcal Vaccines"[MeSH Terms] OR ("meningococcal"[All Fields] AND "vaccines"[All Fields]) OR "Meningococcal Vaccines"[All Fields] OR ("meningococcal"[All Fields] AND "polysaccharide"[All Fields] AND "vaccine"[All Fields]) OR "meningococcal polysaccharide vaccine"[All Fields]) OR "Diphtheria-Tetanus-Pertussis Vaccine"[MeSH Terms] OR ("Diphtheria-Tetanus-Pertussis Vaccine"[MeSH Terms] OR ("diphtheria tetanus pertussis"[All Fields] AND "vaccine"[All Fields]) OR "Diphtheria-Tetanus-Pertussis Vaccine"[All Fields] OR ("diphtheria"[All Fields] AND "tetanus"[All Fields] AND "pertussis"[All Fields] AND "vaccine"[All Fields]) OR "Diphtheria-Tetanus-Pertussis Vaccine"[All Fields]) OR ("Diphtheria-Tetanus-Pertussis Vaccine"[MeSH Terms] OR ("diphtheria tetanus pertussis"[All Fields] AND "vaccine"[All Fields]) OR "Diphtheria-Tetanus-Pertussis Vaccine"[All Fields] OR ("diphtheria"[All Fields] AND "tetanus"[All Fields] AND "pertussis"[All Fields] AND "vaccine"[All Fields]) OR "Diphtheria-Tetanus-Pertussis Vaccine"[All Fields]) OR ("Diphtheria-Tetanus-Pertussis Vaccine"[MeSH Terms] OR ("diphtheria tetanus pertussis"[All Fields] AND "vaccine"[All Fields]) OR "Diphtheria-Tetanus-Pertussis Vaccine"[All Fields] OR ("dtwp"[All Fields] AND "vaccine"[All Fields]) OR "dtwp vaccine"[All Fields]) OR ("Diphtheria-Tetanus-Pertussis Vaccine"[MeSH Terms] OR ("diphtheria tetanus pertussis"[All Fields] AND "vaccine"[All Fields]) OR "Diphtheria-Tetanus-Pertussis Vaccine"[All Fields] OR ("dpt"[All Fields] AND "vaccine"[All Fields]) OR "dpt vaccine"[All Fields]) OR ("Diphtheria-Tetanus-Pertussis Vaccine"[MeSH Terms] OR ("diphtheria tetanus pertussis"[All Fields] AND "vaccine"[All Fields]) OR "Diphtheria-Tetanus-Pertussis Vaccine"[All Fields] OR ("dtp"[All Fields] AND "vaccine"[All Fields]) OR "dtp vaccine"[All Fields]) OR ("Diphtheria-Tetanus-Pertussis Vaccine"[MeSH Terms] OR ("diphtheria tetanus pertussis"[All Fields] AND "vaccine"[All Fields]) OR "Diphtheria-Tetanus-Pertussis Vaccine"[All Fields] OR ("di"[All Fields] AND "te"[All Fields] AND "per"[All Fields] AND "vaccine"[All Fields]) OR "di te per vaccine"[All Fields]) OR ("Diphtheria-Tetanus-Pertussis Vaccine"[MeSH Terms] OR ("diphtheria tetanus pertussis"[All Fields] AND "vaccine"[All Fields]) OR "Diphtheria-Tetanus-Pertussis Vaccine"[All Fields] OR ("diphtheria"[All Fields] AND "pertussis"[All Fields] AND "tetanus"[All Fields] AND "vaccine"[All Fields]) OR "diphtheria pertussis tetanus vaccine"[All Fields]) OR ("haemophilus influenza type b polysaccharide vaccine tetanus toxin conjugate"[Supplementary Concept] OR "haemophilus influenza type b polysaccharide vaccine tetanus toxin conjugate"[All Fields] OR "haemophilus influenza type b polysaccharide vaccine tetanus toxin conjugate"[All Fields]) OR ("haemophilus influenza type b polysaccharide vaccine tetanus toxin conjugate"[Supplementary Concept] OR "haemophilus influenza type b polysaccharide vaccine tetanus toxin conjugate"[All Fields] OR "prp t"[All Fields]) OR ("haemophilus influenza type b polysaccharide vaccine tetanus toxin conjugate"[Supplementary Concept] OR "haemophilus influenza type b polysaccharide vaccine tetanus toxin conjugate"[All Fields] OR "hib tt"[All Fields]) OR ("haemophilus influenza type b polysaccharide vaccine tetanus toxin conjugate"[Supplementary Concept] OR "haemophilus influenza type b polysaccharide vaccine tetanus toxin conjugate"[All Fields] OR "omnihib"[All Fields]) OR ("haemophilus influenza type b polysaccharide vaccine tetanus toxin conjugate"[Supplementary Concept] OR "haemophilus influenza type b polysaccharide vaccine tetanus toxin conjugate"[All Fields] OR "acthib"[All Fields]))

145 results

**1.2 Following there are search strategy in Embase**

**No.**

**Query**

**Results**

**1,160**

**#35**

**#25 AND #34**

**967,981**

**#34**

**#26 OR #27 OR #28 OR #29 OR #30 OR #31 OR #32 OR #33**

**4,696**

**#33**

**'baricitinib'/syn**

**9,684**

**#32**

**'tofacitinib'/syn**

**714,689**

**#31**

**'protein kinase inhibitor'/syn**

**2,099**

**#30**

**'upadacitinib'/syn**

**30,220**

**#29**

**'janus kinase inhibitor'/syn**

**13,795**

**#28**

**'abatacept'/syn**

**90,399**

**#27**

**'mycophenolic acid'/syn**

**220,948**

**#26**

**'methotrexate'/syn**

**3,124**

**#25**

**#12 AND #24**

**149,994**

**#24**

**#13 OR #14 OR #15 OR #16 OR #17 OR #18 OR #19 OR #20 OR #21 OR #22 OR #23**

**36**

**#23**

**'haemophilus influenza type b polysaccharide vaccine tetanus toxin conjugate'/syn**

**22,584**

**#22**

**'diphtheria pertussis tetanus vaccine'/syn**

**8,774**

**#21**

**'meningococcal vaccines'/syn**

**5,982**

**#20**

**'hepatitis a vaccines'/syn**

**15,713**

**#19**

**'poliovirus vaccines'/syn**

**18,091**

**#18**

**'human papilloma virus vaccine'/syn**

**25,125**

**#17**

**'hepatitis b vaccine'/syn**

**4,441**

**#16**

**'herpes zoster vaccine'/syn**

**25,896**

**#15**

**'pneumococcus vaccine'/syn**

**48,734**

**#14**

**'influenza vaccines'/syn**

**10,379**

**#13**

**'inactivated vaccine'/syn**

**582,415**

**#12**

**#1 OR #2 OR #3 OR #4 OR #5 OR #6 OR #7 OR #8 OR #9 OR #10 OR #11**

**5,506**

**#11**

**'mixed connective tissue disease'/syn**

**43,158**

**#10**

**'systemic sclerosis'/syn**

**35,919**

**#9**

**'sjoegren syndrome'/syn**

**9,231**

**#8**

**'systemic vasculitis'/syn**

**52,684**

**#7**

**'myositis'/syn**

**141,094**

**#6**

**'systemic lupus erythematosus'/syn**

**40,970**

**#5**

**'ankylosing spondylitis'/syn**

**33,640**

**#4**

**'psoriatic arthritis'/syn**

**73,128**

**#3**

**'spondylarthritis'/syn**

**282,694**

**#2**

**'rheumatoid arthritis'/syn**

**56,077**

**#1**

**'rheumatic disease'/de**

**1.3 Following there are search strategy in Cochrane Library**

ID	Search	Hits

#1	MeSH descriptor: [Rheumatic Diseases] explode all trees	20731

#2	MeSH descriptor: [Arthritis, Rheumatoid] explode all trees	7302

#3	MeSH descriptor: [Spondylarthritis] explode all trees	1823

#4	MeSH descriptor: [Arthritis, Psoriatic] explode all trees	634

#5	MeSH descriptor: [Spondylitis, Ankylosing] explode all trees	826

#6	MeSH descriptor: [Lupus Erythematosus, Systemic] explode all trees	1425

#7	MeSH descriptor: [Myositis] explode all trees	318

#8	MeSH descriptor: [Systemic Vasculitis] explode all trees	266

#9	MeSH descriptor: [Sjogren's Syndrome] explode all trees	365

#10	MeSH descriptor: [Scleroderma, Systemic] explode all trees	717

#11	MeSH descriptor: [Mixed Connective Tissue Disease] explode all trees	13

#12	#1 OR #2 OR #3 OR #4 OR #5 OR #6 OR #7 OR #8 OR #9 OR #10 OR #11	24888

#13	MeSH descriptor: [Vaccines, Inactivated] explode all trees	1148

#14	MeSH descriptor: [Influenza Vaccines] explode all trees	1853

#15	MeSH descriptor: [Pneumococcal Vaccines] explode all trees	1178

#16	MeSH descriptor: [Herpes Zoster Vaccine] explode all trees	110

#17	MeSH descriptor: [Hepatitis B Vaccines] explode all trees	1003

#18	MeSH descriptor: [Papillomavirus Vaccines] explode all trees	549

#19	MeSH descriptor: [Poliovirus Vaccines] explode all trees	544

#20	MeSH descriptor: [Hepatitis A Vaccines] explode all trees	258

#21	MeSH descriptor: [Meningococcal Vaccines] explode all trees	438

#22	MeSH descriptor: [Diphtheria-Tetanus-Pertussis Vaccine] explode all trees	585

#23	#13 OR #14 OR #15 OR #16 OR #17 OR #18 OR #19 OR #20 OR #21 OR #22	5937

#24	MeSH descriptor: [Janus Kinase Inhibitors] explode all trees	145

#25	MeSH descriptor: [Protein Kinase Inhibitors] explode all trees	1498

#26	MeSH descriptor: [Methotrexate] explode all trees	4768

#27	MeSH descriptor: [Mycophenolic Acid] explode all trees	1572

#28	MeSH descriptor: [Abatacept] explode all trees	355

#29	#24 OR #25 OR #26 OR #27 OR #28	7998

#30	#12 AND #23 AND #29	**25**

**QUESTION 10 - Is there a difference in the immune response between conventional “tri- and tetravalent’’ and high-dose influenza vaccines in patients with immune-mediated rheumatic diseases (IMRD)?**

**1.1 Following there are search strategy in PubMed/MEDLINE**

**P = Patients with immune-mediated rheumatic diseases**

(Rheumatic Diseases) OR (Rheumatic Disease) OR (Rheumatism) OR (Immune-mediated rheumatic diseases) OR (Arthritis, Rheumatoid) OR (Rheumatoid Arthritis) OR (Rheumatoid Nodule) OR (Rheumatoid Vasculitis) OR (Sjogren's Syndrome) OR "Spondylarthritis" [Mesh] OR (Spondylarthritis) OR (Spondylarthritides) OR (Spinal Arthritis) OR "Arthritis, Psoriatic" [Mesh] OR (Arthritis, Psoriatic) OR (Arthritic Psoriasis) OR (Psoriatic Arthritis) OR (Psoriasis Arthropathica) OR (Psoriatic Arthropathy) OR (Psoriatic Arthropathies) OR "Spondylitis, Ankylosing" [Mesh] OR (Spondylitis, Ankylosing) OR (Bechterew's Disease) OR (Bechterews Disease) OR (Marie Struempell Disease) OR (Spondylarthritis Ankylopoietica) OR (Spondyloarthritis Ankylopoietica) OR (Ankylosing Spondylitis) OR (Ankylosing Spondylarthritis) OR (Ankylosing Spondylarthritides) OR (Ankylosing Spondyloarthritis) OR (Spondylitis Ankylopoietica) OR (Bechterew Disease) OR (Rheumatoid Spondylitis) OR "Lupus Erythematosus, Systemic" [Mesh] OR (Lupus Erythematosus, Systemic) OR (Systemic Lupus Erythematosus) OR (Lupus Erythematosus Disseminatus) OR (Libman Sacks Disease) OR "Myositis" [Mesh] OR (Myositis) OR (Myositides) OR (Inflammatory Muscle Diseases) OR (Inflammatory Muscle Disease) OR (Inflammatory Myopathy) OR (Inflammatory Myopathies) OR (Proliferative Myositis) OR (Infectious Myositides) OR (Infectious Myositis) OR (Idiopathic Inflammatory Myopathies) OR (Idiopathic Inflammatory Myopathy) OR (Idiopathic Inflammatory Myositis) OR (Focal Myositis) OR "Systemic Vasculitis" [Mesh] OR (Systemic Vasculitis) OR (Systemic Vasculitides) OR "Sjogren's Syndrome" [Mesh] OR (Sjogren's Syndrome) OR (Sjogrens Syndrome) OR (Sjogren Syndrome) OR (Sicca Syndrome) OR "Scleroderma, Systemic" [Mesh] OR (Scleroderma, Systemic) OR (Systemic Sclerosis) OR (Systemic Scleroderma) OR "Mixed Connective Tissue Disease" [Mesh] OR (Mixed Connective Tissue Disease) OR (Sharp Syndrome) OR (MCTD)

**I = Vaccination with inactivated vaccine**

"Vaccines, Inactivated" [Mesh] OR (Vaccines, Inactivated) OR (Inactivated Vaccines) OR (Killed Vaccine) OR (Killed Vaccines) OR (Inactivated Vaccine) OR (Influenza Vaccines) OR (Influenza Vaccine) OR (Influenzavirus Vaccines) OR (Influenza Virus Vaccines) OR (Influenzavirus Vaccine) OR (Influenza Virus Vaccine) OR (High-Dose Trivalent Influenza Vaccine) OR (High Dose Trivalent Influenza Vaccine) OR (Trivalent Influenza Vaccine) OR (Quadrivalent Influenza Vaccine) OR "Pneumococcal Vaccines" [Mesh] OR (Pneumococcal Vaccines) OR (Pneumococcal Vaccine) OR (Pneumovax) OR (Pneumococcal Polysaccharide Vaccine) OR (Pnu Imune Vaccine) OR (PnuImune Vaccine) OR (Conjugated penumococcal vaccine) OR (Herpes Zoster Vaccine) OR (Zoster Vaccine) OR (Shingles Vaccine) OR "Hepatitis B Vaccines" [Mesh] OR (Hepatitis B Vaccines) OR (Hepatitis B Vaccine) OR "Papillomavirus Vaccines" [Mesh] OR (Papillomavirus Vaccines) OR (Papillomavirus Vaccine) OR (Human Papillomavirus Vaccines) OR (HPV Vaccine) OR (Human Papilloma Virus Vaccine) OR (HPV Vaccines) OR (Human Papilloma Virus Vaccines) OR (Human Papillomavirus Vaccine) OR "Poliovirus Vaccines" [Mesh] OR (Poliovirus Vaccines) OR (Poliovirus Vaccine) OR (salk) OR "Hepatitis A Vaccines" [Mesh] OR (Hepatitis A Vaccines) OR (Hepatovirus Vaccines) OR (Hepatitis A Vaccine) OR (Vaqta) OR (Havrix) OR "Meningococcal Vaccines" [Mesh] OR (Meningococcal Vaccine) OR (Menactra) OR (Tetravalent Meningococcal Vaccine) OR (Menomune) OR (Menveo) OR (Bivalent Meningococcal Vaccine) OR (Meningococcal Polysaccharide Vaccine) OR "Diphtheria-Tetanus-Pertussis Vaccine" [Mesh] OR (Diphtheria-Tetanus-Pertussis Vaccine) OR (Diphtheria Tetanus Pertussis Vaccine) OR (DTwP Vaccine) OR (DPT Vaccine) OR (DTP Vaccine) OR (Di Te Per Vaccine) OR (Diphtheria Pertussis Tetanus Vaccine) OR (Haemophilus influenza type b polysaccharide vaccine-tetanus toxin conjugate) OR (PRP-T) OR (Hib-TT) OR (OmniHIB) OR (ActHIB)

#1 AND #2

("rheumatic diseases"[MeSH Terms] OR ("rheumatic"[All Fields] AND "diseases"[All Fields]) OR "rheumatic diseases"[All Fields] OR ("rheumatic diseases"[MeSH Terms] OR ("rheumatic"[All Fields] AND "diseases"[All Fields]) OR "rheumatic diseases"[All Fields] OR ("rheumatic"[All Fields] AND "disease"[All Fields]) OR "rheumatic disease"[All Fields]) OR ("rheumatic diseases"[MeSH Terms] OR ("rheumatic"[All Fields] AND "diseases"[All Fields]) OR "rheumatic diseases"[All Fields] OR "rheumatics"[All Fields] OR "rheumatism"[All Fields] OR "rheumatic"[All Fields] OR "rheumatisms"[All Fields]) OR ("Immune-mediated"[All Fields] AND ("rheumatic diseases"[MeSH Terms] OR ("rheumatic"[All Fields] AND "diseases"[All Fields]) OR "rheumatic diseases"[All Fields])) OR ("arthritis, rheumatoid"[MeSH Terms] OR ("arthritis"[All Fields] AND "rheumatoid"[All Fields]) OR "rheumatoid arthritis"[All Fields] OR ("arthritis"[All Fields] AND "rheumatoid"[All Fields]) OR "arthritis rheumatoid"[All Fields]) OR ("arthritis, rheumatoid"[MeSH Terms] OR ("arthritis"[All Fields] AND "rheumatoid"[All Fields]) OR "rheumatoid arthritis"[All Fields] OR ("rheumatoid"[All Fields] AND "arthritis"[All Fields])) OR ("rheumatoid nodule"[MeSH Terms] OR ("rheumatoid"[All Fields] AND "nodule"[All Fields]) OR "rheumatoid nodule"[All Fields]) OR ("rheumatoid vasculitis"[MeSH Terms] OR ("rheumatoid"[All Fields] AND "vasculitis"[All Fields]) OR "rheumatoid vasculitis"[All Fields]) OR ("Sjogren's Syndrome"[MeSH Terms] OR ("sjogren s"[All Fields] AND "syndrome"[All Fields]) OR "Sjogren's Syndrome"[All Fields]) OR "Spondylarthritis"[MeSH Terms] OR ("Spondylarthritis"[MeSH Terms] OR "Spondylarthritis"[All Fields] OR "spondylarthritides"[All Fields]) OR ("Spondylarthritis"[MeSH Terms] OR "Spondylarthritis"[All Fields] OR "spondylarthritides"[All Fields]) OR ("Spondylarthritis"[MeSH Terms] OR "Spondylarthritis"[All Fields] OR ("spinal"[All Fields] AND "arthritis"[All Fields]) OR "spinal arthritis"[All Fields]) OR "arthritis, psoriatic"[MeSH Terms] OR ("arthritis, psoriatic"[MeSH Terms] OR ("arthritis"[All Fields] AND "psoriatic"[All Fields]) OR "psoriatic arthritis"[All Fields] OR ("arthritis"[All Fields] AND "psoriatic"[All Fields]) OR "arthritis psoriatic"[All Fields]) OR ("arthritis, psoriatic"[MeSH Terms] OR ("arthritis"[All Fields] AND "psoriatic"[All Fields]) OR "psoriatic arthritis"[All Fields] OR ("arthritic"[All Fields] AND "psoriasis"[All Fields]) OR "arthritic psoriasis"[All Fields]) OR ("arthritis, psoriatic"[MeSH Terms] OR ("arthritis"[All Fields] AND "psoriatic"[All Fields]) OR "psoriatic arthritis"[All Fields] OR ("psoriatic"[All Fields] AND "arthritis"[All Fields])) OR ("arthritis, psoriatic"[MeSH Terms] OR ("arthritis"[All Fields] AND "psoriatic"[All Fields]) OR "psoriatic arthritis"[All Fields] OR ("psoriasis"[All Fields] AND "arthropathica"[All Fields]) OR "psoriasis arthropathica"[All Fields]) OR ("arthritis, psoriatic"[MeSH Terms] OR ("arthritis"[All Fields] AND "psoriatic"[All Fields]) OR "psoriatic arthritis"[All Fields] OR ("psoriatic"[All Fields] AND "arthropathy"[All Fields]) OR "psoriatic arthropathy"[All Fields]) OR ("arthritis, psoriatic"[MeSH Terms] OR ("arthritis"[All Fields] AND "psoriatic"[All Fields]) OR "psoriatic arthritis"[All Fields] OR ("psoriatic"[All Fields] AND "arthropathies"[All Fields]) OR "psoriatic arthropathies"[All Fields]) OR "spondylitis, ankylosing"[MeSH Terms] OR ("spondylitis, ankylosing"[MeSH Terms] OR ("spondylitis"[All Fields] AND "ankylosing"[All Fields]) OR "ankylosing spondylitis"[All Fields] OR ("spondylitis"[All Fields] AND "ankylosing"[All Fields]) OR "spondylitis ankylosing"[All Fields]) OR ("spondylitis, ankylosing"[MeSH Terms] OR ("spondylitis"[All Fields] AND "ankylosing"[All Fields]) OR "ankylosing spondylitis"[All Fields] OR ("bechterew s"[All Fields] AND "disease"[All Fields]) OR "bechterew s disease"[All Fields]) OR ("spondylitis, ankylosing"[MeSH Terms] OR ("spondylitis"[All Fields] AND "ankylosing"[All Fields]) OR "ankylosing spondylitis"[All Fields] OR ("bechterews"[All Fields] AND "disease"[All Fields]) OR "bechterews disease"[All Fields]) OR ("spondylitis, ankylosing"[MeSH Terms] OR ("spondylitis"[All Fields] AND "ankylosing"[All Fields]) OR "ankylosing spondylitis"[All Fields] OR ("marie"[All Fields] AND "struempell"[All Fields] AND "disease"[All Fields]) OR "marie struempell disease"[All Fields]) OR ("spondylitis, ankylosing"[MeSH Terms] OR ("spondylitis"[All Fields] AND "ankylosing"[All Fields]) OR "ankylosing spondylitis"[All Fields] OR ("Spondylarthritis"[All Fields] AND "ankylopoietica"[All Fields]) OR "spondylarthritis ankylopoietica"[All Fields]) OR ("spondylitis, ankylosing"[MeSH Terms] OR ("spondylitis"[All Fields] AND "ankylosing"[All Fields]) OR "ankylosing spondylitis"[All Fields] OR ("spondyloarthritis"[All Fields] AND "ankylopoietica"[All Fields]) OR "spondyloarthritis ankylopoietica"[All Fields]) OR ("spondylitis, ankylosing"[MeSH Terms] OR ("spondylitis"[All Fields] AND "ankylosing"[All Fields]) OR "ankylosing spondylitis"[All Fields] OR ("ankylosing"[All Fields] AND "spondylitis"[All Fields])) OR ("spondylitis, ankylosing"[MeSH Terms] OR ("spondylitis"[All Fields] AND "ankylosing"[All Fields]) OR "ankylosing spondylitis"[All Fields] OR ("ankylosing"[All Fields] AND "Spondylarthritis"[All Fields]) OR "ankylosing spondylarthritis"[All Fields]) OR ("spondylitis, ankylosing"[MeSH Terms] OR ("spondylitis"[All Fields] AND "ankylosing"[All Fields]) OR "ankylosing spondylitis"[All Fields] OR ("ankylosing"[All Fields] AND "spondylarthritides"[All Fields]) OR "ankylosing spondylarthritides"[All Fields]) OR ("spondylitis, ankylosing"[MeSH Terms] OR ("spondylitis"[All Fields] AND "ankylosing"[All Fields]) OR "ankylosing spondylitis"[All Fields] OR ("ankylosing"[All Fields] AND "spondyloarthritis"[All Fields]) OR "ankylosing spondyloarthritis"[All Fields]) OR ("spondylitis, ankylosing"[MeSH Terms] OR ("spondylitis"[All Fields] AND "ankylosing"[All Fields]) OR "ankylosing spondylitis"[All Fields] OR ("spondylitis"[All Fields] AND "ankylopoietica"[All Fields]) OR "spondylitis ankylopoietica"[All Fields]) OR ("spondylitis, ankylosing"[MeSH Terms] OR ("spondylitis"[All Fields] AND "ankylosing"[All Fields]) OR "ankylosing spondylitis"[All Fields] OR ("bechterew"[All Fields] AND "disease"[All Fields]) OR "bechterew disease"[All Fields]) OR ("spondylitis, ankylosing"[MeSH Terms] OR ("spondylitis"[All Fields] AND "ankylosing"[All Fields]) OR "ankylosing spondylitis"[All Fields] OR ("rheumatoid"[All Fields] AND "spondylitis"[All Fields]) OR "rheumatoid spondylitis"[All Fields]) OR "lupus erythematosus, systemic"[MeSH Terms] OR ("lupus erythematosus, systemic"[MeSH Terms] OR ("lupus"[All Fields] AND "erythematosus"[All Fields] AND "systemic"[All Fields]) OR "systemic lupus erythematosus"[All Fields] OR ("lupus"[All Fields] AND "erythematosus"[All Fields] AND "systemic"[All Fields]) OR "lupus erythematosus systemic"[All Fields]) OR ("lupus erythematosus, systemic"[MeSH Terms] OR ("lupus"[All Fields] AND "erythematosus"[All Fields] AND "systemic"[All Fields]) OR "systemic lupus erythematosus"[All Fields] OR ("systemic"[All Fields] AND "lupus"[All Fields] AND "erythematosus"[All Fields])) OR ("lupus erythematosus, systemic"[MeSH Terms] OR ("lupus"[All Fields] AND "erythematosus"[All Fields] AND "systemic"[All Fields]) OR "systemic lupus erythematosus"[All Fields] OR ("lupus"[All Fields] AND "erythematosus"[All Fields] AND "disseminatus"[All Fields]) OR "lupus erythematosus disseminatus"[All Fields]) OR ("lupus erythematosus, systemic"[MeSH Terms] OR ("lupus"[All Fields] AND "erythematosus"[All Fields] AND "systemic"[All Fields]) OR "systemic lupus erythematosus"[All Fields] OR ("libman"[All Fields] AND "sacks"[All Fields] AND "disease"[All Fields]) OR "libman sacks disease"[All Fields]) OR "Myositis"[MeSH Terms] OR ("Myositis"[MeSH Terms] OR "Myositis"[All Fields] OR "myositides"[All Fields]) OR ("Myositis"[MeSH Terms] OR "Myositis"[All Fields] OR "myositides"[All Fields]) OR ("Myositis"[MeSH Terms] OR "Myositis"[All Fields] OR ("inflammatory"[All Fields] AND "muscle"[All Fields] AND "diseases"[All Fields]) OR "inflammatory muscle diseases"[All Fields]) OR ("Myositis"[MeSH Terms] OR "Myositis"[All Fields] OR ("inflammatory"[All Fields] AND "muscle"[All Fields] AND "disease"[All Fields]) OR "inflammatory muscle disease"[All Fields]) OR ("Myositis"[MeSH Terms] OR "Myositis"[All Fields] OR ("inflammatory"[All Fields] AND "myopathy"[All Fields]) OR "inflammatory myopathy"[All Fields]) OR ("Myositis"[MeSH Terms] OR "Myositis"[All Fields] OR ("inflammatory"[All Fields] AND "myopathies"[All Fields]) OR "inflammatory myopathies"[All Fields]) OR ("Myositis"[MeSH Terms] OR "Myositis"[All Fields] OR ("proliferative"[All Fields] AND "Myositis"[All Fields]) OR "proliferative myositis"[All Fields]) OR ("Myositis"[MeSH Terms] OR "Myositis"[All Fields] OR ("infectious"[All Fields] AND "myositides"[All Fields]) OR "infectious myositides"[All Fields]) OR ("Myositis"[MeSH Terms] OR "Myositis"[All Fields] OR ("infectious"[All Fields] AND "Myositis"[All Fields]) OR "infectious myositis"[All Fields]) OR ("Myositis"[MeSH Terms] OR "Myositis"[All Fields] OR ("idiopathic"[All Fields] AND "inflammatory"[All Fields] AND "myopathies"[All Fields]) OR "idiopathic inflammatory myopathies"[All Fields]) OR ("Myositis"[MeSH Terms] OR "Myositis"[All Fields] OR ("idiopathic"[All Fields] AND "inflammatory"[All Fields] AND "myopathy"[All Fields]) OR "idiopathic inflammatory myopathy"[All Fields]) OR ("Myositis"[MeSH Terms] OR "Myositis"[All Fields] OR ("idiopathic"[All Fields] AND "inflammatory"[All Fields] AND "Myositis"[All Fields]) OR "idiopathic inflammatory myositis"[All Fields]) OR ("Myositis"[MeSH Terms] OR "Myositis"[All Fields] OR ("focal"[All Fields] AND "Myositis"[All Fields]) OR "focal myositis"[All Fields]) OR "Systemic Vasculitis"[MeSH Terms] OR ("Systemic Vasculitis"[MeSH Terms] OR ("systemic"[All Fields] AND "vasculitis"[All Fields]) OR "Systemic Vasculitis"[All Fields]) OR ("Systemic Vasculitis"[MeSH Terms] OR ("systemic"[All Fields] AND "vasculitis"[All Fields]) OR "Systemic Vasculitis"[All Fields] OR ("systemic"[All Fields] AND "vasculitides"[All Fields]) OR "systemic vasculitides"[All Fields]) OR "Sjogren's Syndrome"[MeSH Terms] OR ("Sjogren's Syndrome"[MeSH Terms] OR ("sjogren s"[All Fields] AND "syndrome"[All Fields]) OR "Sjogren's Syndrome"[All Fields]) OR ("Sjogren's Syndrome"[MeSH Terms] OR ("sjogren s"[All Fields] AND "syndrome"[All Fields]) OR "Sjogren's Syndrome"[All Fields] OR ("sjogrens"[All Fields] AND "syndrome"[All Fields]) OR "sjogrens syndrome"[All Fields]) OR ("Sjogren's Syndrome"[MeSH Terms] OR ("sjogren s"[All Fields] AND "syndrome"[All Fields]) OR "Sjogren's Syndrome"[All Fields] OR ("sjogren"[All Fields] AND "syndrome"[All Fields]) OR "sjogren syndrome"[All Fields]) OR ("Sjogren's Syndrome"[MeSH Terms] OR ("sjogren s"[All Fields] AND "syndrome"[All Fields]) OR "Sjogren's Syndrome"[All Fields] OR ("sicca"[All Fields] AND "syndrome"[All Fields]) OR "sicca syndrome"[All Fields]) OR "scleroderma, systemic"[MeSH Terms] OR ("scleroderma, systemic"[MeSH Terms] OR ("scleroderma"[All Fields] AND "systemic"[All Fields]) OR "systemic scleroderma"[All Fields] OR ("scleroderma"[All Fields] AND "systemic"[All Fields]) OR "scleroderma systemic"[All Fields]) OR ("scleroderma, systemic"[MeSH Terms] OR ("scleroderma"[All Fields] AND "systemic"[All Fields]) OR "systemic scleroderma"[All Fields] OR ("systemic"[All Fields] AND "sclerosis"[All Fields]) OR "systemic sclerosis"[All Fields]) OR ("scleroderma, systemic"[MeSH Terms] OR ("scleroderma"[All Fields] AND "systemic"[All Fields]) OR "systemic scleroderma"[All Fields] OR ("systemic"[All Fields] AND "scleroderma"[All Fields])) OR "Mixed Connective Tissue Disease"[MeSH Terms] OR ("Mixed Connective Tissue Disease"[MeSH Terms] OR ("mixed"[All Fields] AND "connective"[All Fields] AND "tissue"[All Fields] AND "disease"[All Fields]) OR "Mixed Connective Tissue Disease"[All Fields]) OR ("Mixed Connective Tissue Disease"[MeSH Terms] OR ("mixed"[All Fields] AND "connective"[All Fields] AND "tissue"[All Fields] AND "disease"[All Fields]) OR "Mixed Connective Tissue Disease"[All Fields] OR ("sharp"[All Fields] AND "syndrome"[All Fields]) OR "sharp syndrome"[All Fields]) OR ("Mixed Connective Tissue Disease"[MeSH Terms] OR ("mixed"[All Fields] AND "connective"[All Fields] AND "tissue"[All Fields] AND "disease"[All Fields]) OR "Mixed Connective Tissue Disease"[All Fields] OR "mctd"[All Fields])) AND ("vaccines, inactivated"[MeSH Terms] OR ("vaccines, inactivated"[MeSH Terms] OR ("vaccines"[All Fields] AND "inactivated"[All Fields]) OR "inactivated vaccines"[All Fields] OR ("vaccines"[All Fields] AND "inactivated"[All Fields]) OR "vaccines inactivated"[All Fields]) OR ("vaccines, inactivated"[MeSH Terms] OR ("vaccines"[All Fields] AND "inactivated"[All Fields]) OR "inactivated vaccines"[All Fields] OR ("inactivated"[All Fields] AND "vaccines"[All Fields])) OR ("vaccines, inactivated"[MeSH Terms] OR ("vaccines"[All Fields] AND "inactivated"[All Fields]) OR "inactivated vaccines"[All Fields] OR ("killed"[All Fields] AND "vaccine"[All Fields]) OR "killed vaccine"[All Fields]) OR ("vaccines, inactivated"[MeSH Terms] OR ("vaccines"[All Fields] AND "inactivated"[All Fields]) OR "inactivated vaccines"[All Fields] OR ("killed"[All Fields] AND "vaccines"[All Fields]) OR "killed vaccines"[All Fields]) OR ("vaccines, inactivated"[MeSH Terms] OR ("vaccines"[All Fields] AND "inactivated"[All Fields]) OR "inactivated vaccines"[All Fields] OR ("inactivated"[All Fields] AND "vaccine"[All Fields]) OR "inactivated vaccine"[All Fields]) OR ("influenza vaccines"[MeSH Terms] OR ("influenza"[All Fields] AND "vaccines"[All Fields]) OR "influenza vaccines"[All Fields]) OR ("influenza vaccines"[MeSH Terms] OR ("influenza"[All Fields] AND "vaccines"[All Fields]) OR "influenza vaccines"[All Fields] OR ("influenza"[All Fields] AND "vaccine"[All Fields]) OR "influenza vaccine"[All Fields]) OR ("influenza vaccines"[MeSH Terms] OR ("influenza"[All Fields] AND "vaccines"[All Fields]) OR "influenza vaccines"[All Fields] OR ("influenzavirus"[All Fields] AND "vaccines"[All Fields])) OR ("influenza vaccines"[MeSH Terms] OR ("influenza"[All Fields] AND "vaccines"[All Fields]) OR "influenza vaccines"[All Fields] OR ("influenza"[All Fields] AND "virus"[All Fields] AND "vaccines"[All Fields]) OR "influenza virus vaccines"[All Fields]) OR ("influenza vaccines"[MeSH Terms] OR ("influenza"[All Fields] AND "vaccines"[All Fields]) OR "influenza vaccines"[All Fields] OR ("influenzavirus"[All Fields] AND "vaccine"[All Fields]) OR "influenzavirus vaccine"[All Fields]) OR ("influenza vaccines"[MeSH Terms] OR ("influenza"[All Fields] AND "vaccines"[All Fields]) OR "influenza vaccines"[All Fields] OR ("influenza"[All Fields] AND "virus"[All Fields] AND "vaccine"[All Fields]) OR "influenza virus vaccine"[All Fields]) OR ("influenza vaccines"[MeSH Terms] OR ("influenza"[All Fields] AND "vaccines"[All Fields]) OR "influenza vaccines"[All Fields] OR ("high"[All Fields] AND "dose"[All Fields] AND "trivalent"[All Fields] AND "influenza"[All Fields] AND "vaccine"[All Fields]) OR "high dose trivalent influenza vaccine"[All Fields]) OR ("influenza vaccines"[MeSH Terms] OR ("influenza"[All Fields] AND "vaccines"[All Fields]) OR "influenza vaccines"[All Fields] OR ("high"[All Fields] AND "dose"[All Fields] AND "trivalent"[All Fields] AND "influenza"[All Fields] AND "vaccine"[All Fields]) OR "high dose trivalent influenza vaccine"[All Fields]) OR ("influenza vaccines"[MeSH Terms] OR ("influenza"[All Fields] AND "vaccines"[All Fields]) OR "influenza vaccines"[All Fields] OR ("trivalent"[All Fields] AND "influenza"[All Fields] AND "vaccine"[All Fields]) OR "trivalent influenza vaccine"[All Fields]) OR ("influenza vaccines"[MeSH Terms] OR ("influenza"[All Fields] AND "vaccines"[All Fields]) OR "influenza vaccines"[All Fields] OR ("quadrivalent"[All Fields] AND "influenza"[All Fields] AND "vaccine"[All Fields]) OR "quadrivalent influenza vaccine"[All Fields]) OR "Pneumococcal Vaccines"[MeSH Terms] OR ("Pneumococcal Vaccines"[MeSH Terms] OR ("pneumococcal"[All Fields] AND "vaccines"[All Fields]) OR "Pneumococcal Vaccines"[All Fields]) OR ("Pneumococcal Vaccines"[MeSH Terms] OR ("pneumococcal"[All Fields] AND "vaccines"[All Fields]) OR "Pneumococcal Vaccines"[All Fields] OR ("pneumococcal"[All Fields] AND "vaccine"[All Fields]) OR "pneumococcal vaccine"[All Fields]) OR ("Pneumococcal Vaccines"[MeSH Terms] OR ("pneumococcal"[All Fields] AND "vaccines"[All Fields]) OR "Pneumococcal Vaccines"[All Fields] OR "pneumovax"[All Fields]) OR ("Pneumococcal Vaccines"[MeSH Terms] OR ("pneumococcal"[All Fields] AND "vaccines"[All Fields]) OR "Pneumococcal Vaccines"[All Fields] OR ("pneumococcal"[All Fields] AND "polysaccharide"[All Fields] AND "vaccine"[All Fields]) OR "pneumococcal polysaccharide vaccine"[All Fields]) OR ("Pneumococcal Vaccines"[MeSH Terms] OR ("pneumococcal"[All Fields] AND "vaccines"[All Fields]) OR "Pneumococcal Vaccines"[All Fields] OR ("pnu"[All Fields] AND "imune"[All Fields] AND "vaccine"[All Fields]) OR "pnu imune vaccine"[All Fields]) OR ("Pneumococcal Vaccines"[MeSH Terms] OR ("pneumococcal"[All Fields] AND "vaccines"[All Fields]) OR "Pneumococcal Vaccines"[All Fields] OR ("pnuimune"[All Fields] AND "vaccine"[All Fields])) OR (("conjugable"[All Fields] OR "conjugal"[All Fields] OR "conjugally"[All Fields] OR "conjugant"[All Fields] OR "conjugants"[All Fields] OR "conjugate"[All Fields] OR "conjugate s"[All Fields] OR "conjugated"[All Fields] OR "conjugates"[All Fields] OR "conjugating"[All Fields] OR "conjugation"[All Fields] OR "conjugational"[All Fields] OR "conjugations"[All Fields] OR "conjugative"[All Fields] OR "conjugatively"[All Fields]) AND "penumococcal"[All Fields] AND ("vaccin"[Supplementary Concept] OR "vaccin"[All Fields] OR "vaccination"[MeSH Terms] OR "vaccination"[All Fields] OR "vaccinable"[All Fields] OR "vaccinal"[All Fields] OR "vaccinate"[All Fields] OR "vaccinated"[All Fields] OR "vaccinates"[All Fields] OR "vaccinating"[All Fields] OR "vaccinations"[All Fields] OR "vaccination s"[All Fields] OR "vaccinator"[All Fields] OR "vaccinators"[All Fields] OR "vaccine s"[All Fields] OR "vaccined"[All Fields] OR "vaccines"[MeSH Terms] OR "vaccines"[All Fields] OR "vaccine"[All Fields] OR "vaccins"[All Fields])) OR ("herpes zoster vaccine"[MeSH Terms] OR ("herpes"[All Fields] AND "zoster"[All Fields] AND "vaccine"[All Fields]) OR "herpes zoster vaccine"[All Fields]) OR ("herpes zoster vaccine"[MeSH Terms] OR ("herpes"[All Fields] AND "zoster"[All Fields] AND "vaccine"[All Fields]) OR "herpes zoster vaccine"[All Fields] OR ("zoster"[All Fields] AND "vaccine"[All Fields]) OR "zoster vaccine"[All Fields]) OR ("herpes zoster vaccine"[MeSH Terms] OR ("herpes"[All Fields] AND "zoster"[All Fields] AND "vaccine"[All Fields]) OR "herpes zoster vaccine"[All Fields] OR ("shingles"[All Fields] AND "vaccine"[All Fields]) OR "shingles vaccine"[All Fields]) OR "Hepatitis B Vaccines"[MeSH Terms] OR ("Hepatitis B Vaccines"[MeSH Terms] OR "Hepatitis B Vaccines"[All Fields]) OR ("Hepatitis B Vaccines"[MeSH Terms] OR "Hepatitis B Vaccines"[All Fields] OR "hepatitis b vaccine"[All Fields]) OR "Papillomavirus Vaccines"[MeSH Terms] OR ("Papillomavirus Vaccines"[MeSH Terms] OR ("papillomavirus"[All Fields] AND "vaccines"[All Fields]) OR "Papillomavirus Vaccines"[All Fields]) OR ("Papillomavirus Vaccines"[MeSH Terms] OR ("papillomavirus"[All Fields] AND "vaccines"[All Fields]) OR "Papillomavirus Vaccines"[All Fields] OR ("papillomavirus"[All Fields] AND "vaccine"[All Fields]) OR "papillomavirus vaccine"[All Fields]) OR ("Papillomavirus Vaccines"[MeSH Terms] OR ("papillomavirus"[All Fields] AND "vaccines"[All Fields]) OR "Papillomavirus Vaccines"[All Fields] OR ("human"[All Fields] AND "papillomavirus"[All Fields] AND "vaccines"[All Fields]) OR "human papillomavirus vaccines"[All Fields]) OR ("Papillomavirus Vaccines"[MeSH Terms] OR ("papillomavirus"[All Fields] AND "vaccines"[All Fields]) OR "Papillomavirus Vaccines"[All Fields] OR ("hpv"[All Fields] AND "vaccine"[All Fields]) OR "hpv vaccine"[All Fields]) OR ("Papillomavirus Vaccines"[MeSH Terms] OR ("papillomavirus"[All Fields] AND "vaccines"[All Fields]) OR "Papillomavirus Vaccines"[All Fields] OR ("human"[All Fields] AND "papilloma"[All Fields] AND "virus"[All Fields] AND "vaccine"[All Fields]) OR "human papilloma virus vaccine"[All Fields]) OR ("Papillomavirus Vaccines"[MeSH Terms] OR ("papillomavirus"[All Fields] AND "vaccines"[All Fields]) OR "Papillomavirus Vaccines"[All Fields] OR ("hpv"[All Fields] AND "vaccines"[All Fields]) OR "hpv vaccines"[All Fields]) OR ("Papillomavirus Vaccines"[MeSH Terms] OR ("papillomavirus"[All Fields] AND "vaccines"[All Fields]) OR "Papillomavirus Vaccines"[All Fields] OR ("human"[All Fields] AND "papilloma"[All Fields] AND "virus"[All Fields] AND "vaccines"[All Fields]) OR "human papilloma virus vaccines"[All Fields]) OR ("Papillomavirus Vaccines"[MeSH Terms] OR ("papillomavirus"[All Fields] AND "vaccines"[All Fields]) OR "Papillomavirus Vaccines"[All Fields] OR ("human"[All Fields] AND "papillomavirus"[All Fields] AND "vaccine"[All Fields]) OR "human papillomavirus vaccine"[All Fields]) OR "Poliovirus Vaccines"[MeSH Terms] OR ("Poliovirus Vaccines"[MeSH Terms] OR ("poliovirus"[All Fields] AND "vaccines"[All Fields]) OR "Poliovirus Vaccines"[All Fields]) OR ("Poliovirus Vaccines"[MeSH Terms] OR ("poliovirus"[All Fields] AND "vaccines"[All Fields]) OR "Poliovirus Vaccines"[All Fields] OR ("poliovirus"[All Fields] AND "vaccine"[All Fields]) OR "poliovirus vaccine"[All Fields]) OR "salk"[All Fields] OR "Hepatitis A Vaccines"[MeSH Terms] OR ("Hepatitis A Vaccines"[MeSH Terms] OR "Hepatitis A Vaccines"[All Fields]) OR ("Hepatitis A Vaccines"[MeSH Terms] OR "Hepatitis A Vaccines"[All Fields] OR ("hepatovirus"[All Fields] AND "vaccines"[All Fields])) OR ("Hepatitis A Vaccines"[MeSH Terms] OR "Hepatitis A Vaccines"[All Fields] OR "hepatitis a vaccine"[All Fields]) OR ("hepatitis a vaccine inactivated"[All Fields] OR "Hepatitis A Vaccines"[MeSH Terms] OR "Hepatitis A Vaccines"[All Fields] OR "vaqta"[All Fields]) OR ("Hepatitis A Vaccines"[MeSH Terms] OR "Hepatitis A Vaccines"[All Fields] OR "havrix"[All Fields]) OR "Meningococcal Vaccines"[MeSH Terms] OR ("Meningococcal Vaccines"[MeSH Terms] OR ("meningococcal"[All Fields] AND "vaccines"[All Fields]) OR "Meningococcal Vaccines"[All Fields] OR ("meningococcal"[All Fields] AND "vaccine"[All Fields]) OR "meningococcal vaccine"[All Fields]) OR ("Meningococcal Vaccines"[MeSH Terms] OR ("meningococcal"[All Fields] AND "vaccines"[All Fields]) OR "Meningococcal Vaccines"[All Fields] OR "menactra"[All Fields]) OR ("Meningococcal Vaccines"[MeSH Terms] OR ("meningococcal"[All Fields] AND "vaccines"[All Fields]) OR "Meningococcal Vaccines"[All Fields] OR ("tetravalent"[All Fields] AND "meningococcal"[All Fields] AND "vaccine"[All Fields]) OR "tetravalent meningococcal vaccine"[All Fields]) OR ("Meningococcal Vaccines"[MeSH Terms] OR ("meningococcal"[All Fields] AND "vaccines"[All Fields]) OR "Meningococcal Vaccines"[All Fields] OR "menomune"[All Fields]) OR ("Meningococcal Vaccines"[MeSH Terms] OR ("meningococcal"[All Fields] AND "vaccines"[All Fields]) OR "Meningococcal Vaccines"[All Fields] OR "menveo"[All Fields]) OR ("Meningococcal Vaccines"[MeSH Terms] OR ("meningococcal"[All Fields] AND "vaccines"[All Fields]) OR "Meningococcal Vaccines"[All Fields] OR ("bivalent"[All Fields] AND "meningococcal"[All Fields] AND "vaccine"[All Fields]) OR "bivalent meningococcal vaccine"[All Fields]) OR ("Meningococcal Vaccines"[MeSH Terms] OR ("meningococcal"[All Fields] AND "vaccines"[All Fields]) OR "Meningococcal Vaccines"[All Fields] OR ("meningococcal"[All Fields] AND "polysaccharide"[All Fields] AND "vaccine"[All Fields]) OR "meningococcal polysaccharide vaccine"[All Fields]) OR "Diphtheria-Tetanus-Pertussis Vaccine"[MeSH Terms] OR ("Diphtheria-Tetanus-Pertussis Vaccine"[MeSH Terms] OR ("diphtheria tetanus pertussis"[All Fields] AND "vaccine"[All Fields]) OR "Diphtheria-Tetanus-Pertussis Vaccine"[All Fields] OR ("diphtheria"[All Fields] AND "tetanus"[All Fields] AND "pertussis"[All Fields] AND "vaccine"[All Fields]) OR "Diphtheria-Tetanus-Pertussis Vaccine"[All Fields]) OR ("Diphtheria-Tetanus-Pertussis Vaccine"[MeSH Terms] OR ("diphtheria tetanus pertussis"[All Fields] AND "vaccine"[All Fields]) OR "Diphtheria-Tetanus-Pertussis Vaccine"[All Fields] OR ("diphtheria"[All Fields] AND "tetanus"[All Fields] AND "pertussis"[All Fields] AND "vaccine"[All Fields]) OR "Diphtheria-Tetanus-Pertussis Vaccine"[All Fields]) OR ("Diphtheria-Tetanus-Pertussis Vaccine"[MeSH Terms] OR ("diphtheria tetanus pertussis"[All Fields] AND "vaccine"[All Fields]) OR "Diphtheria-Tetanus-Pertussis Vaccine"[All Fields] OR ("dtwp"[All Fields] AND "vaccine"[All Fields]) OR "dtwp vaccine"[All Fields]) OR ("Diphtheria-Tetanus-Pertussis Vaccine"[MeSH Terms] OR ("diphtheria tetanus pertussis"[All Fields] AND "vaccine"[All Fields]) OR "Diphtheria-Tetanus-Pertussis Vaccine"[All Fields] OR ("dpt"[All Fields] AND "vaccine"[All Fields]) OR "dpt vaccine"[All Fields]) OR ("Diphtheria-Tetanus-Pertussis Vaccine"[MeSH Terms] OR ("diphtheria tetanus pertussis"[All Fields] AND "vaccine"[All Fields]) OR "Diphtheria-Tetanus-Pertussis Vaccine"[All Fields] OR ("dtp"[All Fields] AND "vaccine"[All Fields]) OR "dtp vaccine"[All Fields]) OR ("Diphtheria-Tetanus-Pertussis Vaccine"[MeSH Terms] OR ("diphtheria tetanus pertussis"[All Fields] AND "vaccine"[All Fields]) OR "Diphtheria-Tetanus-Pertussis Vaccine"[All Fields] OR ("di"[All Fields] AND "te"[All Fields] AND "per"[All Fields] AND "vaccine"[All Fields]) OR "di te per vaccine"[All Fields]) OR ("Diphtheria-Tetanus-Pertussis Vaccine"[MeSH Terms] OR ("diphtheria tetanus pertussis"[All Fields] AND "vaccine"[All Fields]) OR "Diphtheria-Tetanus-Pertussis Vaccine"[All Fields] OR ("diphtheria"[All Fields] AND "pertussis"[All Fields] AND "tetanus"[All Fields] AND "vaccine"[All Fields]) OR "diphtheria pertussis tetanus vaccine"[All Fields]) OR ("haemophilus influenza type b polysaccharide vaccine tetanus toxin conjugate"[Supplementary Concept] OR "haemophilus influenza type b polysaccharide vaccine tetanus toxin conjugate"[All Fields] OR "haemophilus influenza type b polysaccharide vaccine tetanus toxin conjugate"[All Fields]) OR ("haemophilus influenza type b polysaccharide vaccine tetanus toxin conjugate"[Supplementary Concept] OR "haemophilus influenza type b polysaccharide vaccine tetanus toxin conjugate"[All Fields] OR "prp t"[All Fields]) OR ("haemophilus influenza type b polysaccharide vaccine tetanus toxin conjugate"[Supplementary Concept] OR "haemophilus influenza type b polysaccharide vaccine tetanus toxin conjugate"[All Fields] OR "hib tt"[All Fields]) OR ("haemophilus influenza type b polysaccharide vaccine tetanus toxin conjugate"[Supplementary Concept] OR "haemophilus influenza type b polysaccharide vaccine tetanus toxin conjugate"[All Fields] OR "omnihib"[All Fields]) OR ("haemophilus influenza type b polysaccharide vaccine tetanus toxin conjugate"[Supplementary Concept] OR "haemophilus influenza type b polysaccharide vaccine tetanus toxin conjugate"[All Fields] OR "acthib"[All Fields]))

1,128 results

**1.2 Following there are search strategy in Embase**

**No.**

**Query**

**Results**

**#25	3,124**

**#12 AND #24**

**149,994**

**#24**

**#13 OR #14 OR #15 OR #16 OR #17 OR #18 OR #19 OR #20 OR #21 OR #22 OR #23**

**36**

**#23**

**'haemophilus influenza type b polysaccharide vaccine tetanus toxin conjugate'/syn**

**22,584**

**#22**

**'diphtheria pertussis tetanus vaccine'/syn**

**8,774**

**#21**

**'meningococcal vaccines'/syn**

**5,982**

**#20**

**'hepatitis a vaccines'/syn**

**15,713**

**#19**

**'poliovirus vaccines'/syn**

**18,091**

**#18**

**'human papilloma virus vaccine'/syn**

**25,125**

**#17**

**'hepatitis b vaccine'/syn**

**4,441**

**#16**

**'herpes zoster vaccine'/syn**

**25,896**

**#15**

**'pneumococcus vaccine'/syn**

**48,734**

**#14**

**'influenza vaccines'/syn**

**10,379**

**#13**

**'inactivated vaccine'/syn**

**582,415**

**#12**

**#1 OR #2 OR #3 OR #4 OR #5 OR #6 OR #7 OR #8 OR #9 OR #10 OR #11**

**5,506**

**#11**

**'mixed connective tissue disease'/syn**

**43,158**

**#10**

**'systemic sclerosis'/syn**

**35,919**

**#9**

**'sjoegren syndrome'/syn**

**9,231**

**#8**

**'systemic vasculitis'/syn**

**52,684**

**#7**

**'myositis'/syn**

**141,094**

**#6**

**'systemic lupus erythematosus'/syn**

**40,970**

**#5**

**'ankylosing spondylitis'/syn**

**33,640**

**#4**

**'psoriatic arthritis'/syn**

**73,128**

**#3**

**'spondylarthritis'/syn**

**282,694**

**#2**

**'rheumatoid arthritis'/syn**

**56,077**

**#1**

**'rheumatic disease'/de**

**1.3 Following there are search strategy in Cochrane Library**

ID	Search	Hits

#1	MeSH descriptor: [Rheumatic Diseases] explode all trees	20731

#2	MeSH descriptor: [Arthritis, Rheumatoid] explode all trees	7302

#3	MeSH descriptor: [Spondylarthritis] explode all trees	1823

#4	MeSH descriptor: [Arthritis, Psoriatic] explode all trees	634

#5	MeSH descriptor: [Spondylitis, Ankylosing] explode all trees	826

#6	MeSH descriptor: [Lupus Erythematosus, Systemic] explode all trees	1425

#7	MeSH descriptor: [Myositis] explode all trees	318

#8	MeSH descriptor: [Systemic Vasculitis] explode all trees	266

#9	MeSH descriptor: [Sjogren's Syndrome] explode all trees	365

#10	MeSH descriptor: [Scleroderma, Systemic] explode all trees	717

#11	MeSH descriptor: [Mixed Connective Tissue Disease] explode all trees	13

#12	#1 OR #2 OR #3 OR #4 OR #5 OR #6 OR #7 OR #8 OR #9 OR #10 OR #11	24888

#13	MeSH descriptor: [Vaccines, Inactivated] explode all trees	1148

#14	MeSH descriptor: [Influenza Vaccines] explode all trees	1853

#15	MeSH descriptor: [Pneumococcal Vaccines] explode all trees	1178

#16	MeSH descriptor: [Herpes Zoster Vaccine] explode all trees	110

#17	MeSH descriptor: [Hepatitis B Vaccines] explode all trees	1003

#18	MeSH descriptor: [Papillomavirus Vaccines] explode all trees	549

#19	MeSH descriptor: [Poliovirus Vaccines] explode all trees	544

#20	MeSH descriptor: [Hepatitis A Vaccines] explode all trees	258

#21	MeSH descriptor: [Meningococcal Vaccines] explode all trees	438

#22	MeSH descriptor: [Diphtheria-Tetanus-Pertussis Vaccine] explode all trees	585

#23	#13 OR #14 OR #15 OR #16 OR #17 OR #18 OR #19 OR #20 OR #21 OR #22	5937

#24	#12 AND #23	62

**QUESTION 11. Is there a difference in the immune response between conventional “tri- and tetravalent) and high-dose Hepatitis B Vaccines in patients with immune-mediated rheumatic diseases (IMRD)?**

**1.1 Following there are search strategy in PubMed/MEDLINE**

**P = Patients with immune-mediated rheumatic diseases**

("rheumatic diseases"[MeSH Terms] OR ("rheumatic"[All Fields] AND "diseases"[All Fields]) OR "rheumatic diseases"[All Fields] OR ("rheumatic diseases"[MeSH Terms] OR ("rheumatic"[All Fields] AND "diseases"[All Fields]) OR "rheumatic diseases"[All Fields] OR ("rheumatic"[All Fields] AND "disease"[All Fields]) OR "rheumatic disease"[All Fields]) OR ("rheumatic diseases"[MeSH Terms] OR ("rheumatic"[All Fields] AND "diseases"[All Fields]) OR "rheumatic diseases"[All Fields] OR "rheumatics"[All Fields] OR "rheumatism"[All Fields] OR "rheumatic"[All Fields] OR "rheumatisms"[All Fields]) OR ("Immune-mediated"[All Fields] AND ("rheumatic diseases"[MeSH Terms] OR ("rheumatic"[All Fields] AND "diseases"[All Fields]) OR "rheumatic diseases"[All Fields])) OR ("arthritis, rheumatoid"[MeSH Terms] OR ("arthritis"[All Fields] AND "rheumatoid"[All Fields]) OR "rheumatoid arthritis"[All Fields] OR "arthritis rheumatoid"[All Fields]) OR ("arthritis, rheumatoid"[MeSH Terms] OR ("arthritis"[All Fields] AND "rheumatoid"[All Fields]) OR "rheumatoid arthritis"[All Fields] OR ("rheumatoid"[All Fields] AND "arthritis"[All Fields])) OR ("rheumatoid nodule"[MeSH Terms] OR ("rheumatoid"[All Fields] AND "nodule"[All Fields]) OR "rheumatoid nodule"[All Fields]) OR ("rheumatoid vasculitis"[MeSH Terms] OR ("rheumatoid"[All Fields] AND "vasculitis"[All Fields]) OR "rheumatoid vasculitis"[All Fields]) OR ("Sjogren's Syndrome"[MeSH Terms] OR ("sjogren s"[All Fields] AND "syndrome"[All Fields]) OR "Sjogren's Syndrome"[All Fields]) OR "Spondylarthritis"[MeSH Terms] OR ("Spondylarthritis"[MeSH Terms] OR "Spondylarthritis"[All Fields] OR "spondylarthritides"[All Fields]) OR ("Spondylarthritis"[MeSH Terms] OR "Spondylarthritis"[All Fields] OR "spondylarthritides"[All Fields]) OR ("Spondylarthritis"[MeSH Terms] OR "Spondylarthritis"[All Fields] OR ("spinal"[All Fields] AND "arthritis"[All Fields]) OR "spinal arthritis"[All Fields]) OR "arthritis, psoriatic"[MeSH Terms] OR ("arthritis, psoriatic"[MeSH Terms] OR ("arthritis"[All Fields] AND "psoriatic"[All Fields]) OR "psoriatic arthritis"[All Fields] OR "arthritis psoriatic"[All Fields]) OR ("arthritis, psoriatic"[MeSH Terms] OR ("arthritis"[All Fields] AND "psoriatic"[All Fields]) OR "psoriatic arthritis"[All Fields] OR ("arthritic"[All Fields] AND "psoriasis"[All Fields]) OR "arthritic psoriasis"[All Fields]) OR ("arthritis, psoriatic"[MeSH Terms] OR ("arthritis"[All Fields] AND "psoriatic"[All Fields]) OR "psoriatic arthritis"[All Fields] OR ("psoriatic"[All Fields] AND "arthritis"[All Fields])) OR ("arthritis, psoriatic"[MeSH Terms] OR ("arthritis"[All Fields] AND "psoriatic"[All Fields]) OR "psoriatic arthritis"[All Fields] OR ("psoriasis"[All Fields] AND "arthropathica"[All Fields]) OR "psoriasis arthropathica"[All Fields]) OR ("arthritis, psoriatic"[MeSH Terms] OR ("arthritis"[All Fields] AND "psoriatic"[All Fields]) OR "psoriatic arthritis"[All Fields] OR ("psoriatic"[All Fields] AND "arthropathy"[All Fields]) OR "psoriatic arthropathy"[All Fields]) OR ("arthritis, psoriatic"[MeSH Terms] OR ("arthritis"[All Fields] AND "psoriatic"[All Fields]) OR "psoriatic arthritis"[All Fields] OR ("psoriatic"[All Fields] AND "arthropathies"[All Fields]) OR "psoriatic arthropathies"[All Fields]) OR "spondylitis, ankylosing"[MeSH Terms] OR ("spondylitis, ankylosing"[MeSH Terms] OR ("spondylitis"[All Fields] AND "ankylosing"[All Fields]) OR "ankylosing spondylitis"[All Fields] OR "spondylitis ankylosing"[All Fields]) OR ("spondylitis, ankylosing"[MeSH Terms] OR ("spondylitis"[All Fields] AND "ankylosing"[All Fields]) OR "ankylosing spondylitis"[All Fields] OR ("bechterew s"[All Fields] AND "disease"[All Fields]) OR "bechterew s disease"[All Fields]) OR ("spondylitis, ankylosing"[MeSH Terms] OR ("spondylitis"[All Fields] AND "ankylosing"[All Fields]) OR "ankylosing spondylitis"[All Fields] OR ("bechterews"[All Fields] AND "disease"[All Fields]) OR "bechterews disease"[All Fields]) OR ("spondylitis, ankylosing"[MeSH Terms] OR ("spondylitis"[All Fields] AND "ankylosing"[All Fields]) OR "ankylosing spondylitis"[All Fields] OR ("marie"[All Fields] AND "struempell"[All Fields] AND "disease"[All Fields]) OR "marie struempell disease"[All Fields]) OR ("spondylitis, ankylosing"[MeSH Terms] OR ("spondylitis"[All Fields] AND "ankylosing"[All Fields]) OR "ankylosing spondylitis"[All Fields] OR ("Spondylarthritis"[All Fields] AND "ankylopoietica"[All Fields]) OR "spondylarthritis ankylopoietica"[All Fields]) OR ("spondylitis, ankylosing"[MeSH Terms] OR ("spondylitis"[All Fields] AND "ankylosing"[All Fields]) OR "ankylosing spondylitis"[All Fields] OR ("spondyloarthritis"[All Fields] AND "ankylopoietica"[All Fields]) OR "spondyloarthritis ankylopoietica"[All Fields]) OR ("spondylitis, ankylosing"[MeSH Terms] OR ("spondylitis"[All Fields] AND "ankylosing"[All Fields]) OR "ankylosing spondylitis"[All Fields] OR ("ankylosing"[All Fields] AND "spondylitis"[All Fields])) OR ("spondylitis, ankylosing"[MeSH Terms] OR ("spondylitis"[All Fields] AND "ankylosing"[All Fields]) OR "ankylosing spondylitis"[All Fields] OR ("ankylosing"[All Fields] AND "Spondylarthritis"[All Fields]) OR "ankylosing spondylarthritis"[All Fields]) OR ("spondylitis, ankylosing"[MeSH Terms] OR ("spondylitis"[All Fields] AND "ankylosing"[All Fields]) OR "ankylosing spondylitis"[All Fields] OR ("ankylosing"[All Fields] AND "spondylarthritides"[All Fields]) OR "ankylosing spondylarthritides"[All Fields]) OR ("spondylitis, ankylosing"[MeSH Terms] OR ("spondylitis"[All Fields] AND "ankylosing"[All Fields]) OR "ankylosing spondylitis"[All Fields] OR ("ankylosing"[All Fields] AND "spondyloarthritis"[All Fields]) OR "ankylosing spondyloarthritis"[All Fields]) OR ("spondylitis, ankylosing"[MeSH Terms] OR ("spondylitis"[All Fields] AND "ankylosing"[All Fields]) OR "ankylosing spondylitis"[All Fields] OR ("spondylitis"[All Fields] AND "ankylopoietica"[All Fields]) OR "spondylitis ankylopoietica"[All Fields]) OR ("spondylitis, ankylosing"[MeSH Terms] OR ("spondylitis"[All Fields] AND "ankylosing"[All Fields]) OR "ankylosing spondylitis"[All Fields] OR ("bechterew"[All Fields] AND "disease"[All Fields]) OR "bechterew disease"[All Fields]) OR ("spondylitis, ankylosing"[MeSH Terms] OR ("spondylitis"[All Fields] AND "ankylosing"[All Fields]) OR "ankylosing spondylitis"[All Fields] OR ("rheumatoid"[All Fields] AND "spondylitis"[All Fields]) OR "rheumatoid spondylitis"[All Fields]) OR "lupus erythematosus, systemic"[MeSH Terms] OR ("lupus erythematosus, systemic"[MeSH Terms] OR ("lupus"[All Fields] AND "erythematosus"[All Fields] AND "systemic"[All Fields]) OR "systemic lupus erythematosus"[All Fields] OR "lupus erythematosus systemic"[All Fields]) OR ("lupus erythematosus, systemic"[MeSH Terms] OR ("lupus"[All Fields] AND "erythematosus"[All Fields] AND "systemic"[All Fields]) OR "systemic lupus erythematosus"[All Fields] OR ("systemic"[All Fields] AND "lupus"[All Fields] AND "erythematosus"[All Fields])) OR ("lupus erythematosus, systemic"[MeSH Terms] OR ("lupus"[All Fields] AND "erythematosus"[All Fields] AND "systemic"[All Fields]) OR "systemic lupus erythematosus"[All Fields] OR ("lupus"[All Fields] AND "erythematosus"[All Fields] AND "disseminatus"[All Fields]) OR "lupus erythematosus disseminatus"[All Fields]) OR ("lupus erythematosus, systemic"[MeSH Terms] OR ("lupus"[All Fields] AND "erythematosus"[All Fields] AND "systemic"[All Fields]) OR "systemic lupus erythematosus"[All Fields] OR ("libman"[All Fields] AND "sacks"[All Fields] AND "disease"[All Fields]) OR "libman sacks disease"[All Fields]) OR "Myositis"[MeSH Terms] OR ("Myositis"[MeSH Terms] OR "Myositis"[All Fields] OR "myositides"[All Fields]) OR ("Myositis"[MeSH Terms] OR "Myositis"[All Fields] OR "myositides"[All Fields]) OR ("Myositis"[MeSH Terms] OR "Myositis"[All Fields] OR ("inflammatory"[All Fields] AND "muscle"[All Fields] AND "diseases"[All Fields]) OR "inflammatory muscle diseases"[All Fields]) OR ("Myositis"[MeSH Terms] OR "Myositis"[All Fields] OR ("inflammatory"[All Fields] AND "muscle"[All Fields] AND "disease"[All Fields]) OR "inflammatory muscle disease"[All Fields]) OR ("Myositis"[MeSH Terms] OR "Myositis"[All Fields] OR ("inflammatory"[All Fields] AND "myopathy"[All Fields]) OR "inflammatory myopathy"[All Fields]) OR ("Myositis"[MeSH Terms] OR "Myositis"[All Fields] OR ("inflammatory"[All Fields] AND "myopathies"[All Fields]) OR "inflammatory myopathies"[All Fields]) OR ("Myositis"[MeSH Terms] OR "Myositis"[All Fields] OR ("proliferative"[All Fields] AND "Myositis"[All Fields]) OR "proliferative myositis"[All Fields]) OR ("Myositis"[MeSH Terms] OR "Myositis"[All Fields] OR ("infectious"[All Fields] AND "myositides"[All Fields]) OR "infectious myositides"[All Fields]) OR ("Myositis"[MeSH Terms] OR "Myositis"[All Fields] OR ("infectious"[All Fields] AND "Myositis"[All Fields]) OR "infectious myositis"[All Fields]) OR ("Myositis"[MeSH Terms] OR "Myositis"[All Fields] OR ("idiopathic"[All Fields] AND "inflammatory"[All Fields] AND "myopathies"[All Fields]) OR "idiopathic inflammatory myopathies"[All Fields]) OR ("Myositis"[MeSH Terms] OR "Myositis"[All Fields] OR ("idiopathic"[All Fields] AND "inflammatory"[All Fields] AND "myopathy"[All Fields]) OR "idiopathic inflammatory myopathy"[All Fields]) OR ("Myositis"[MeSH Terms] OR "Myositis"[All Fields] OR ("idiopathic"[All Fields] AND "inflammatory"[All Fields] AND "Myositis"[All Fields]) OR "idiopathic inflammatory myositis"[All Fields]) OR ("Myositis"[MeSH Terms] OR "Myositis"[All Fields] OR ("focal"[All Fields] AND "Myositis"[All Fields]) OR "focal myositis"[All Fields]) OR "Systemic Vasculitis"[MeSH Terms] OR ("Systemic Vasculitis"[MeSH Terms] OR ("systemic"[All Fields] AND "vasculitis"[All Fields]) OR "Systemic Vasculitis"[All Fields]) OR ("Systemic Vasculitis"[MeSH Terms] OR ("systemic"[All Fields] AND "vasculitis"[All Fields]) OR "Systemic Vasculitis"[All Fields] OR ("systemic"[All Fields] AND "vasculitides"[All Fields]) OR "systemic vasculitides"[All Fields]) OR "Sjogren's Syndrome"[MeSH Terms] OR ("Sjogren's Syndrome"[MeSH Terms] OR ("sjogren s"[All Fields] AND "syndrome"[All Fields]) OR "Sjogren's Syndrome"[All Fields]) OR ("Sjogren's Syndrome"[MeSH Terms] OR ("sjogren s"[All Fields] AND "syndrome"[All Fields]) OR "Sjogren's Syndrome"[All Fields] OR ("sjogrens"[All Fields] AND "syndrome"[All Fields]) OR "sjogrens syndrome"[All Fields]) OR ("Sjogren's Syndrome"[MeSH Terms] OR ("sjogren s"[All Fields] AND "syndrome"[All Fields]) OR "Sjogren's Syndrome"[All Fields] OR ("sjogren"[All Fields] AND "syndrome"[All Fields]) OR "sjogren syndrome"[All Fields]) OR ("Sjogren's Syndrome"[MeSH Terms] OR ("sjogren s"[All Fields] AND "syndrome"[All Fields]) OR "Sjogren's Syndrome"[All Fields] OR ("sicca"[All Fields] AND "syndrome"[All Fields]) OR "sicca syndrome"[All Fields]) OR "scleroderma, systemic"[MeSH Terms] OR ("scleroderma, systemic"[MeSH Terms] OR ("scleroderma"[All Fields] AND "systemic"[All Fields]) OR "systemic scleroderma"[All Fields] OR "scleroderma systemic"[All Fields]) OR ("scleroderma, systemic"[MeSH Terms] OR ("scleroderma"[All Fields] AND "systemic"[All Fields]) OR "systemic scleroderma"[All Fields] OR ("systemic"[All Fields] AND "sclerosis"[All Fields]) OR "systemic sclerosis"[All Fields]) OR ("scleroderma, systemic"[MeSH Terms] OR ("scleroderma"[All Fields] AND "systemic"[All Fields]) OR "systemic scleroderma"[All Fields] OR ("systemic"[All Fields] AND "scleroderma"[All Fields])) OR "Mixed Connective Tissue Disease"[MeSH Terms] OR ("Mixed Connective Tissue Disease"[MeSH Terms] OR ("mixed"[All Fields] AND "connective"[All Fields] AND "tissue"[All Fields] AND "disease"[All Fields]) OR "Mixed Connective Tissue Disease"[All Fields]) OR ("Mixed Connective Tissue Disease"[MeSH Terms] OR ("mixed"[All Fields] AND "connective"[All Fields] AND "tissue"[All Fields] AND "disease"[All Fields]) OR "Mixed Connective Tissue Disease"[All Fields] OR ("sharp"[All Fields] AND "syndrome"[All Fields]) OR "sharp syndrome"[All Fields]) OR ("Mixed Connective Tissue Disease"[MeSH Terms] OR ("mixed"[All Fields] AND "connective"[All Fields] AND "tissue"[All Fields] AND "disease"[All Fields]) OR "Mixed Connective Tissue Disease"[All Fields] OR "mctd"[All Fields])) AND ("Hepatitis B Vaccines"[MeSH Terms] OR ("Hepatitis B Vaccines"[MeSH Terms] OR "Hepatitis B Vaccines"[All Fields] OR "vaccines hepatitis b"[All Fields]) OR ("Hepatitis B Vaccines"[MeSH Terms] OR "Hepatitis B Vaccines"[All Fields] OR "hepatitis b vaccine"[All Fields]) OR ("Hepatitis B Vaccines"[MeSH Terms] OR "Hepatitis B Vaccines"[All Fields] OR "vaccine hepatitis b"[All Fields]))

118 results

**1.2 Following there are search strategy in Embase**

**#14**

#12 AND #13

565

**#13**

**'hepatitis b vaccines'**/syn

[25,457](https://www-embase.ez27.periodicos.capes.gov.br/)

**#12**

#1 OR #2 OR #3 OR #4 OR #5 OR #6 OR #7 OR #8 OR #9 OR #10 OR #11

[601,200](https://www-embase.ez27.periodicos.capes.gov.br/)

**#11**

**'mixed connective tissue disease'**/syn

[5,648](https://www-embase.ez27.periodicos.capes.gov.br/)

- [Edit](https://www-embase.ez27.periodicos.capes.gov.br/)
- [Email alert](https://www-embase.ez27.periodicos.capes.gov.br/)
- [RSS feed](https://www-embase.ez27.periodicos.capes.gov.br/)

**#10**

**'systemic sclerosis'**/syn

[44,328](https://www-embase.ez27.periodicos.capes.gov.br/)

**#9**

**'sjoegren syndrome'**/syn

[36,942](https://www-embase.ez27.periodicos.capes.gov.br/)

**#8**

**'systemic vasculitis'**/syn

[9,461](https://www-embase.ez27.periodicos.capes.gov.br/)

**#7**

**'myositis'**/syn

[59,317](https://www-embase.ez27.periodicos.capes.gov.br/)

**#6**

**'systemic lupus erythematosus'**/syn

[144,607](https://www-embase.ez27.periodicos.capes.gov.br/)

**#5**

**'ankylosing spondylitis'**/syn

[42,035](https://www-embase.ez27.periodicos.capes.gov.br/)

**#4**

**'psoriatic arthritis'**/syn

[35,052](https://www-embase.ez27.periodicos.capes.gov.br/)

**#3**

**'spondylarthritis'**/syn

[75,587](https://www-embase.ez27.periodicos.capes.gov.br/)

**#2**

**'rheumatoid arthritis'**/syn

[289,084](https://www-embase.ez27.periodicos.capes.gov.br/)

**#1**

**'rheumatic disease'**/de

[57,132](https://www-embase.ez27.periodicos.capes.gov.br/)

**1.3 Following there are search strategy in Cochrane Library**

 Search Name:

Date Run:  	08/11/2023 12:29:58

Comment:

ID  	Search Hits

#1   	MeSH descriptor: [Rheumatic Diseases] explode all trees   21099

#2   	MeSH descriptor: [Arthritis, Rheumatoid] explode all trees       	7384

#3   	MeSH descriptor: [Spondylarthritis] explode all trees    	1857

#4   	MeSH descriptor: [Arthritis, Psoriatic] explode all trees 	646

#5   	MeSH descriptor: [Spondylitis, Ankylosing] explode all trees   	842

#6   	MeSH descriptor: [Lupus Erythematosus, Systemic] explode all trees  	1449

#7   	MeSH descriptor: [Myositis] explode all trees    	325

#8   	MeSH descriptor: [Systemic Vasculitis] explode all trees   275

#9   	MeSH descriptor: [Sjogren's Syndrome] explode all trees   374

#10 	MeSH descriptor: [Scleroderma, Systemic] explode all trees     	733

#11 	MeSH descriptor: [Mixed Connective Tissue Disease] explode all trees	13

#12 	#1 OR #2 OR #3 OR #4 OR #5 OR #6 OR #7 OR #8 OR #9 OR #10 OR #11 	25338

#13 	MeSH descriptor: [Hepatitis B Vaccines] explode all trees 1006

#14 	#12 AND #13 2
