# Supplementary material for: Brazilian Society of Rheumatology – 2025 recommendations on vaccination in immune-mediated rheumatic diseases
Source: Adv Rheumatol. 2026 Jan 23;66:14. doi: 10.1186/s42358-026-00520-8 (PMC13488773; doi:10.1186/s42358-026-00520-8)
Supplement: Supplementary file 2 [file 42358_2026_520_MOESM2_ESM.docx]

**RECOMMENDATIONS OF THE BRAZILIAN SOCIETY OF RHEUMATOLOGY FOR VACCINATION IN PATIENTS WITH IMMUNE MEDIATED RHEUMATIC DISEASES - *Risk of bias***

**QUESTION 1. What is the risk of symptom flare up or worsening of IMRD disease activity after vaccination with the inactivated vaccine?**

| **QUESTION 1** | **Y (%)** | **N (%)** | **U (%)** |
| --- | --- | --- | --- |
| RANDOMIZED CLINICAL TRIAL | 57,68 | 15,50 | 26,83 |
| COHORT | 80,56 | 19,44 | 0 |
| CASE CONTROL | 87,5 | 12,5 | 0 |
| CASE SERIES | 87,5 | 12,5 | 0 |

**QUESTION 2. What is the risk of infection and  symptom flare up or worsening of IMRD disease activity after vaccination with live attenuated vaccine (LAV)?**

| **JBI tools** | **Y (%)** | **N (%)** | **U (%)** |
| --- | --- | --- | --- |
| RANDOMIZED CLINICAL TRIAL | 54,55 | 3,03 | 42,42 |
| COHORT | 90,0 | 6,67 | 3,33 |
| CROSS SECTIONAL | 96,43 | 7,14 | 3,57 |
| CASE SERIES | 87,50 | 0 | 12,5 |

*Note: Y - yes, N - no; U - unclear*

**QUESTION 03. What is the impact of the cs-DMARDs (MTX, LEF, SZL, HXQ) use on the response to inactivated vaccines in patients with immune-mediated rheumatic diseases?**

| **QUESTION 3** | **Y (%)** | **N (%)** | **U (%)** |
| --- | --- | --- | --- |
| RANDOMIZED CLINICAL TRIAL | 28,01 | 47,18 | 1,72 |
| COHORT | 52.15 | 21.53 | 2.87 |
| CROSS SECTIONAL | 37.5 | 43.75 | 6.25 |
| CASE-CONTROL | 18.75 | 25 | 6.25 |

**QUESTION 4. What is the impact of the immunosuppressants “azathioprine, mycophenolate mofetil, cyclosporine, cyclophosphamide’’ use on the response to inactivated vaccine in patients with immune-mediated rheumatic diseases?**

| **JBI tools** | **Y (%)** | **N (%)** | **U (%)** |
| --- | --- | --- | --- |
| **RANDOMIZED CLINICAL TRIAL** | **56,16** | **16,57** | **27,27** |
| **COHORT** | **84,0** | **16,0** | **0** |
| **CASE SERIES** | **87,50** | **12,50** | **0** |
| **CASE CONTROL** | **75,0** | **25,0** | **0** |
| **CROSS SECTIONAL** | **62,50** | **25,0** | **0** |

***Note: Y - yes, N - no; U – unclear***

**QUESTION 5. What is the effect of bDMARDs use (except RTX) on the response to inactivated vaccines in patients with immune-mediated rheumatic diseases (IMRD)?**

| **QUESTION 5** | **Y (%)** | **N (%)** | **U (%)** |
| --- | --- | --- | --- |
| RANDOMIZED CLINICAL TRIAL | 56.16 | 16.57 | 27.27 |
| COHORT | 84.00 | 16.00 | 0.00 |
| CASE CONTROL | 75.00 | 25.00 | 0.00 |
| CROSS-SECTIONAL | 62.50 | 25.00 | 0.00 |

**QUESTION 6 - What is the impact of the RTX use on the response to inactivated vaccine in patients with immune-mediated rheumatic diseases?**

| **QUESTION 6** | **Y (%)** | **N (%)** | **U (%)** |
| --- | --- | --- | --- |
| RANDOMIZED CLINICAL TRIAL | 24.14 | 49.40 | 2.36 |
| COHORT | 43.03 | 36.06 | 3.63 |

**QUESTION 7. What is the effect of corticosteroid use on the response to inactivated vaccines in patients with immune-mediated rheumatic diseases?**

| **QUESTION 7** | **Y (%)** | **N (%)** | **U (%)** |
| --- | --- | --- | --- |
| RANDOMIZED CLINICAL TRIAL | 56.16 | 16.57 | 27.27 |
| COHORT | 84.00 | 16.00 | 0.00 |
| CASE CONTROL | 75.00 | 25.00 | 0.00 |
| CROSS-SECTIONAL | 62.50 | 25.00 | 0.00 |

**QUESTION 8. What is the impact of JAKis use on the response to inactivated vaccines in patients with immune-mediated rheumatic diseases?**

| **JBI tools** | **Y (%)** | **N (%)** | **U (%)** |
| --- | --- | --- | --- |
| RANDOMIZED CLINICAL TRIAL | 56.92 | 1.81 | 30.90 |
| COHORT | 51.94 | 1.78 | 26.78 |
| CASE CONTROL | 70.0 | 12.5 | 0.0 |

**QUESTION 09. Is there a benefit to vaccine response by temporarily stopping methotrexate, mycophenolate, iJAKs before or after vaccination?**

|  | **Y (%)** | **N (%)** | **U (%)** |
| --- | --- | --- | --- |
| RANDOMIZED CLINICAL TRIAL | 72,72 | 0 | 0 |
| COHORT | 62,5 | 37,5 | 0 |

**QUESTION 10 - Is there a difference in the immune response between conventional “tri- and tetravalent’’ and high-dose influenza vaccines in patients with immune-mediated rheumatic diseases (IMRD)?**

| **QUESTION 10** | **Y (%)** | **N (%)** | **U (%)** |
| --- | --- | --- | --- |
| RANDOMIZED CLINICAL TRIAL | 60.86 | 12.64 | 26.48 |
| COHORT | 87.5 | 12.5 | 0 |

**QUESTION 11. Is there a difference in the immune response between conventional “tri- and tetravalent) and high-dose Hepatitis B Vaccines in patients with immune-mediated rheumatic diseases (IMRD)?**

| **QUESTION 11** | **Y (%)** | **N (%)** | **U (%)** |
| --- | --- | --- | --- |
| RANDOMIZED CLINICAL TRIAL | 54.54 | 20.20 | 27.27 |
