# Supplementary material for: Brazilian Society of Rheumatology – 2025 recommendations on vaccination in immune-mediated rheumatic diseases
Source: Adv Rheumatol. 2026 Jan 23;66:14. doi: 10.1186/s42358-026-00520-8 (PMC13488773; doi:10.1186/s42358-026-00520-8)
Supplement: Supplementary file 3 [file 42358_2026_520_MOESM3_ESM.docx]

**RECOMMENDATIONS OF THE BRAZILIAN SOCIETY OF RHEUMATOLOGY FOR VACCINATION IN PATIENTS WITH IMMUNE MEDIATED RHEUMATIC DISEASES - *Narrative tables***

**QUESTION 1. What is the risk of symptom flare up or worsening of IMRD disease activity after vaccination with the inactivated vaccine?**

| **Table 1. Main characteristics of the included studies** | | | | | | | |
| --- | --- | --- | --- | --- | --- | --- | --- |
| **Reference** | **Location** | **Journal** | **Study Design** | **IMRD** | **Drugs use** | **Inactivated vaccine** | **Follow-up** |
| Abu-Shakra et al., 2000 | Israel, Asia | The Journal of Rheumatology | Randomized clinical trial | SLE | Immunosuppressant without specification | Influenza Virus Vaccine Monovalent (H1N1) | 12 weeks |
| Acharya et al., 2019 | United States, North America | Arthritis and Rheumatology | Randomized clinical trial | RA, SLE, VAS and Pss | csDMARDs, bDMARDs and corticosteroids | Adjuvanted Herpes Zoster Subunit Vaccine (HZ/su) | NI |
| Adler et al., 2012 | Switzerland, Europe | Rheumatology | Cohort | MCTD, RA, SpA and VAS | csDMARDs, bDMARDs and corticosteroids | Adjuvanted split influenza A/H1N1 vaccine | 26 weeks |
| Baranova et al., 2022 | Russian, Asia | Antibiotiki i Khimioterapiya | Randomized clinical trial | AS and PsA | Immunosuppressant without specification | 23-valent pneumococcal polysaccharide vaccine (PPV-23) | 52 weeks |
| Borba et al., 2011 | Brazil, South America | Lupus | Randomized clinical trial | SLE | Immunomodulators and corticosteroids | Novel split virion, inactivated, non-adjuvanted pandemic H1N1 influenza vaccine | 52 weeks |
| Brodman et al., 1978 | United States, North America | Annals of Internal Medicine | Cohort | SLE | csDMARDs and corticosteroids | Influenza Virus Vaccine Monovalent (H1N1) | 4 weeks |
| Buhler et al., 2019 | Switzerland, Europe | Rheumatology | Cohort | RA, PsA, VAS | csDMARDs, bDMARDs and corticosteroids | dT (diphtheria and tetanus) vaccines | 52 weeks |
| Bukhanova et al., 2018 (1) | Russian, Asia | Clinical and Experimental Rheumatology | Randomized clinical trial | AIRD | csDMARDs, bDMARDs and corticosteroids | Inactivated split-vaccine against influenza | 26 weeks |
| Bukhanova et al., 2018 (2) | Russian, Asia | Annals of the Rheumatic Diseases | Randomized clinical trial | RA | csDMARDs, bDMARDs and corticosteroids | 23-valent pneumococcal polysaccharide vaccine (PPV-23) | 209 weeks |
| Bukhanova et al., 2019 (1) | Russian, Asia | Annals of the Rheumatic Diseases | Randomized clinical trial | RA and APS | csDMARDs, bDMARDs and corticosteroids | Trivalent inactivated influenza vaccine | 26 weeks |
| Bukhanova et al., 2019 (2) | Russian, Asia | Annals of the Rheumatic Diseases | Randomized clinical trial | RA | csDMARDs, bDMARDs and corticosteroids | 23-valent pneumococcal polysaccharide vaccine (PPV-23) | 261 weeks |
| Caporuscio et al., 2015 | Italy, Europe | Annals of the Rheumatic Diseases | Randomized clinical trial | RA | csDMARDs, bDMARDs and corticosteroids | 13-valent pneumococcal vaccine (PCV-13) | 26 weeks |
| Caso et al., 2016 | United States, North America | Clinical and Experimental Rheumatology | Cohort | PsA | bDMARDs | Adjuvanted vaccine for seasonal influenza | 13 weeks |
| Claudino Formiga et al., 2021 | Brazil, South America | Lupus | Randomized clinical trial | SLE | Immunosuppressant without specification | Influenza Virus Vaccine (H3N2) | 4 weeks |
| Colmegna et al., 2019 | Canada, North America | Lancet Rheumatol | Randomized clinical trial | RA | csDMARDs, bDMARDs, tsDMARDs and corticosteroids | High-dose trivalent inactivated influenza vaccine (HD-TIV) and standard-dose quadrivalent influenza vaccine (SD-QIV) | 73 weeks |
| Crowe et al., 2011 | United States, North America | Arthritis Rheumatism | Cohort | SLE | Steroids and Antimalarials | Trivalent inactivated influenza vaccine | 12 weeks |
| Del Porto et al., 2006 | Italy, Europe | Vaccine | Randomized clinical trial | SLE and RA | csDMARDs and corticosteroids | Trivalent inactivated influenza vaccine | 26 weeks |
| Dhar et al., 2014 | United States, North America | Arthritis and Rheumatology | Randomized clinical trial | SLE | Immunosuppressant without specification | Quadrivalent human papillomavirus (HPV) vaccine | 26 weeks |
| Dhar et al., 2017 | United States, North America | Vaccine | Cohort | SLE | csDMARDs and corticosteroids | Quadrivalent human papillomavirus (HPV) vaccine | 26 weeks |
| Eberhardson et al., 2017 | United States, North America | Clinical Infectious Diseases | Randomized clinical trial | MCTD, SSc, PsA, RA and SLE | bDMARDs | Inactivated Varicella Zoster Virus Vaccine | 4 weeks |
| Elkayam et al., 2002 (1) | Israel, Asia | Arthritis Care & Research | Randomized clinical trial | RA, SLE, PsA and AS | csDMARDs, bDMARDs and corticosteroids | Influenza Virus Vaccine Monovalent (H1N1) | 6 weeks |
| Elkayam et al., 2002 (2) | Israel, Asia | Annals of the Rheumatic Diseases | Randomized clinical trial | RA | csDMARDs and corticosteroids | Hepatitis B vaccine (HBV) | 30 weeks |
| Elkayam et al., 2005 | Israel, Asia | Autoimmunity | Randomized clinical trial | SLE | csDMARDs and corticosteroids | 23-valent pneumococcal polysaccharide vaccine (PPV-23) | 9 weeks |
| Fomin et al., 2005 | Israel, Asia | Annals of the Rheumatic Diseases | Randomized clinical trial | RA | csDMARDs, bDMARDs and corticosteroids | Trivalent inactivated influenza vaccine | 13 weeks |
| Furer et al., 2020 | Israel, Asia | Vaccine | Randomized clinical trial | PsA | csDMARDs and bDMARDs | Seasonal influenza vaccine(H1N1, H3N2, B) | 6 weeks |
| Gabay et al., 2011 | Italy, Europe | Arthritis and Rheumatism | Cohort | RA, SpA, PsA, SLE, ANCA-AAV or SRDs or AIRD | csDMARDs, bDMARDs and corticosteroids | Adjuvanted split influenza A/H1N1 vaccine | 24 weeks |
| Grabar et al., 2017 | France, Europe | Vaccine | Randomized clinical trial | SLE | csDMARDs, bDMARDs, corticosteroids and antimalarials | 23-valent PPSV vaccines targeting various serotypes with adjuvant | 52 weeks |
| Hernandez et al., 2015 | Philippines, Asia | Annals of the Rheumatic Diseases | Randomized clinical trial | SLE | Immunomodulators and corticosteroids | Influenza and/ or pneumococcal vaccinations | 8 weeks |
| Herron et al., 1979 | United States, North America | JAMA | Randomized clinical trial | SLE, RA and SRDs | Corticosteroids | Bivalent influenza virus vaccine | 17 weeks |
| Holvast et., 2009 | Poland, Europe | Rheumatology (Oxford) | Randomized clinical trial | SLE | csDMARDs and corticosteroids | Trivalent influenza subunit vaccine | 4 weeks |
| Intongkam et al., 2018 | Thailand, Asia | Journal of Clinical Rheumatology | Randomized clinical trial | RA | csDMARDs, bDMARDs and corticosteroids | Hepatitis B vaccine (HBV) | 24 weeks |
| Jarrett et al., 1980 | United States, North America | Arthritis and Rheumatism | Randomized clinical trial | SLE | csDMARDs and corticosteroids | 14-valent pneumococcal polysaccharide (PPV-14) | 52 weeks |
| Jeffs et al., 2015 | Australia, Oceania | Nephrology (Carlton) | Randomized clinical trial | ANCA- AAV | csDMARDs and corticosteroids | Trivalent inactivated influenza vaccine | 26 weeks |
| Källmark et al., 2020 | Sweden, Europe | Arthritis and Rheumatology | Randomized clinical trial | RA | tsDMARDs | Adjuvanted Herpes Zoster Subunit Vaccine (HZ/su) | 6 weeks |
| Karsh et al., 1980 | United States, North America | Arthritis Rheumatism | Randomized clinical trial | pSS | csDMARDs and corticosteroids | Polyvalent pneumococcal polysaccharide vaccine | 26 weeks |
| Klippel et al., 1979 | United States, North America | Arthritis & Rheumatism | Randomized clinical trial | SLE | csDMARDs, corticosteroids and antimalarials | Polyvalent pneumococcal polysaccharide vaccine | 4 weeks |
| Kostianovsky et al., 2010 | France, Europe | Arthritis and Rheumatism | Randomized clinical trial | SV, SSc, SLE and pSS | Immunosuppressant without specification | Seasonal influenza vaccine (H1N1) | 3 weeks |
| Kostianovsky et al., 2012 | France, Europe | Clinical and Experimental Rheumatology | Randomized clinical trial | SNV,SSc and pSS | bDMARDs and corticosteroids | Trivalent inactivated influenza vaccine | 26 weeks |
| Kuruma et al., 2007 | Brazil, South America | Lupus | Randomized clinical trial | SLE | csDMARDs, corticosteroids and other no especificated | Hepatitis B vaccine (HBV) | 4 weeks |
| Leaung et al., 2022 | United States, North America | Arthritis & Rheumatology | Case series | AIRD | Corticosteroids | Herpes zoster vaccine (HZ) | 52 weeks |
| Lenfant et al., 2021 | United States, North America | Rheumatology (Oxford) | Cohort | RA, VAS, PMR, SLE, PsA, pSS, SpA, Myositis, SSc | csDMARDs, bDMARDs, tsDMARDs and corticosteroids | Recombinant zoster vaccine (RZV) | 52 weeks |
| Litinsky et al., 2012 | Israel, Asia | Clinical and Experimental Rheumatology | Cohort | SSc | csDMARDs and corticosteroids | Trivalent influenza subunit vaccine | 6 weeks |
| Louie et al., 1978 | United States, North America | Annals of Internal Medicine | Case series | SLE | csDMARDs and corticosteroids | Bivalent influenza virus vaccine | 13 weeks |
| Lu et al., 2011 | China, Asia | Vaccine | Cohort | SLE | csDMARDs and corticosteroids | Influenza Virus Vaccine Monovalent (H1N1) | 26 weeks |
| Lukina et al., 2022 | Russian, Asia | Annals of the Rheumatic Diseases | Randomized clinical trial | RA | bDMARDs | 13-valent pneumococcal vaccine (PCV-13) | 52 weeks |
| Mathian et al., 2010 | France, Europe | Arthritis and Rheumatism | Randomized clinical trial | SLE | Immunosuppressant without specification | Influenza Virus Vaccine Monovalent (H1N1) | 6 weeks |
| Miossi et al., 2011 | Brazil, South America |  | Randomized clinical trial | MCTD | csDMARDs and corticosteroids | Trivalent inactivated influenza vaccine | 3 weeks |
| Mok et al., 2011 | China, Asia | Arthritis and Rheumatism | Randomized clinical trial | SLE | Immunosuppressant without specification | Quadrivalent human papillomavirus (HPV) vaccine | 26 weeks |
| Mok et al., 2013 | China, Asia | Annals of the Rheumatic Diseases | Case control | SLE | bDMARDs | Quadrivalent human papillomavirus (HPV) vaccine | 52 weeks |
| Morgan et al., 2016 | United Kingdom, Europe | Arthritis Care and Research | Cohort | SV | csDMARDs, bDMARDs and corticosteroids | Pn 7-valent conjugate vaccine; Haemophilus influenza type b (Hib) and meningococcal (Men) | 26 weeks |
| Muravyeva et al., 2020 | Russian, Asia | Annals of the Rheumatic Diseases | Randomized clinical trial | AS | csDMARDs, bDMARDs and corticosteroids | 23-valent pneumococcal vaccine (PPV-23) | 13 weeks |
| Muravyeva et al., 2021 | Russian, Asia | Annals of the Rheumatic Diseases | Randomized clinical trial | SpA | csDMARDs and bDMARDs | 23-valent pneumococcal polysaccharide vaccine (PPV-23) | 52 weeks |
| Naumtseva et al., 2015 | Russian, Asia | Annals of the Rheumatic Diseases | Randomized clinical trial | RA | csDMARDs | 23-valent pneumococcal polysaccharide vaccine (PPV-23) | 52 weeks |
| Naumtseva et al., 2016 | Russian, Asia | International Journal of Rheumatic Diseases | Randomized clinical trial | RA | csDMARDs | 23-valent pneumococcal polysaccharide vaccine (PPV-23) | 104 weeks |
| Pasoto et al., 2012 | Brazil, South America | Arthritis and Rheumatism | Randomized clinical trial | pSS | csDMARDs and corticosteroids | Influenza Virus Vaccine Monovalent (H1N1) | 52 weeks |
| Pasoto et al., 2013 | Brazil, South America | Pediatric Rheumatology | Cohort | pSS | csDMARDs, corticosteroids and antimalarials | Influenza Virus Vaccine Monovalent (H1N1) | 52 weeks |
| Pasoto et al., 2023 | Brazil, South America | Clinical Rheumatology | Randomized Clinical Trial | pSS | csDMARDs and corticosteroids | Bivalent influenza virus vaccine | 4 weeks |
| Pons et al., 2022 | Spain, Europe | Annals of the Rheumatic Diseases | Randomized clinical trial | RA | csDMARDs and tsDMARDs | New recombinant vaccine effective against HZ | 9 weeks |
| Rezende et al., 2016 | Brazil, South America | Lupus | Cohort | SLE | csDMARDs and corticosteroids | 23-valent pneumococcal polysaccharide vaccine (PPV-23) | 52 weeks |
| Ristow et al., 1978 | United States, North America | Annals of Internal Medicine | Randomized clinical trial | SLE | csDMARDs and corticosteroids | Influenza Virus Vaccine Monovalent (H1N1) | 8 week |
| Romão et al., 2020 | Portugal, Europe | Annals of the Rheumatic Diseases | Randomized clinical trial | AIRD | csDMARDs, bDMARDs and corticosteroids | Hepatitis B vaccine (HBV) | 26 weeks |
| Saad et al., 2011 | Brazil, South America | Annals of the Rheumatic Diseases | Cohort | SLE, RA, AS, SSc, PsA, MCTD and pSS | Immunomodulators and corticosteroids | Influenza Virus Vaccine Monovalent (H1N1) | 3 weeks |
| Sergeeva et al., 2017 | Russian, Asia | Annals of the Rheumatic Diseases | Randomized clinical trial | RA | csDMARDs | 23-valent pneumococcal polysaccharide vaccine (PPV-23) | 104 weeks |
| Setti et al., 2009 | Italy, Europe |  | Cohort | SSc | Immunosuppressant without specification | Trivalent inactivated influenza vaccine | 52 weeks |
| Stevens et al., 2019 | United States, North America | Annals of the Rheumatic Diseases | Randomized clinical trial | RA | csDMARDs, bDMARDs and corticosteroids | Zoster Recombinant Adjuvanted (ZRA) vaccine | 40 weeks |
| Stevens et al., 2020 | United States, North America | ACR Open Rheumatology | Randomized clinical trial | RA and SRDs | csDMARDs, bDMARDs and corticosteroids | Zoster Recombinant Adjuvanted (ZRA) vaccine | 12 weeks |
| Stojanovich L. (2006) | Serbia, Europe | Clinical & Developmental Immunology | Randomized clinical trial | SLE and RA | Immunosuppressant without specification | Trivalent inactivated influenza vaccine | 52 weeks |
| Tarasova et al., 2016 | Russian, Asia | Annals of the Rheumatic Diseases | Randomized clinical trial | SLE | csDMARDs, bDMARDs and corticosteroids | 23-valent pneumococcal polysaccharide vaccine (PPV-23) | 43 weeks |
| Tarasova et al., 2017 | Russian, Asia | Annals of the Rheumatic Diseases | Randomized clinical trial | SLE | csDMARDs, bDMARDs and corticosteroids | 23-valent pneumococcal polysaccharide vaccine (PPV-23) | 52 weeks |
| Tarasova et al., 2018 | Russian, Asia | Annals of the Rheumatic Diseases | Randomized clinical trial | SLE | csDMARDs, bDMARDs and corticosteroids | 23-valent pneumococcal polysaccharide vaccine (PPV-23) | 52 weeks |
| Tarasova et al., 2020 | Russian, Asia | Annals of the Rheumatic Diseases | Randomized clinical trial | SLE | csDMARDs, bDMARDs and corticosteroids | 23-valent pneumococcal polysaccharide vaccine (PPV-23) | 52 weeks |
| Tarasova et al., 2021 (1) | Russian, Asia | Annals of the Rheumatic Diseases | Randomized clinical trial | SLE | csDMARDs, bDMARDs and corticosteroids | 23-valent pneumococcal polysaccharide vaccine (PPV-23) | 13 weeks |
| Tarasova et al., 2021 (2) | Russian, Asia | Annals of the Rheumatic Diseases | Randomized clinical trial | APS | csDMARDs, bDMARDs and corticosteroids | 23-valent pneumococcal polysaccharide vaccine (PPV-23) | 52 weeks |
| Tarasova et al., 2022 | Russian, Asia | Annals Rheumatic Diseases | Randomized clinical trial | SV | csDMARDs, bDMARDs and corticosteroids | 23-valent pneumococcal polysaccharide vaccine (PPV-23) | 26 weeks |
| Tarján et al., 2002 | Hungary, Europe | Scandinavian Journal of Rheumatology | Cohort | SLE | csDMARDs and corticosteroids | 23-valent pneumococcal polysaccharide vaccine (PPV-23) | 4 weeks |
| Vagelli et al., 2017 | Italy, Europe | Annals of the Rheumatic Diseases | Randomized clinical trial | SLE, RA, SSc, pSS, BD and Dermatomyositis | bDMARDs and corticosteroids | Influenza and/or meningococcal C vaccinations | 52 weeks |
| Venerito et al., 2023 | Italy, Europe | International Journal of Molecular Sciences | Cohort | RA | bDMARDs and tsDMARDs | Adjuvanted Recombinant Zoster Vaccine | 4 weeks |
| Wallin et al., 2009 | Brazil, South America | Acta Reumatologica Portuguesa | Randomized clinical trial | SLE | csDMARDs and corticosteroids | Trivalent inactivated influenza vaccine | 6 weeks |
| Wiesik-Szewczyk et al., 2010 | Poland, Europe | Clinical Rheumatology | Randomized clinical trial | SLE | csDMARDs and corticosteroids | Trivalent inactivated influenza vaccine | 12 weeks |
| Williams et al., 1978 | United States, North America | Annals of Internal Medicine | Randomized clinical trial | SLE | Immunosuppressant without specification | Bivalent influenza virus vaccine | 20 weeks |
| Winthrop et al., 2019 | United States and Puerto Rico, North America | Arthritis Research & Therapy | Randomized clinical trial | RA | tsDMARDs | 13-valent pneumococcal vaccine (PCV-13) | 52 weeks |
| Zhou et al., 2021 | China, Asia | Rheumatology (Oxford) | Cohort | pSS | Immunosuppressant without specification | Quadrivalent influenza vaccine (live attenuated) | 13 weeks |
| ***Note****: Diseases: Immune-mediated rheumatic disease (IMRD); Ankylosing spondylitis (AS), Antineutrophil cytoplasmic antibody associated vasculitis (ANCA- AAV), Antiphospholipid syndrome (APS), Autoimmune inflammatory rheumatic diseases (AIRD), Behçet disease (BD), Immune-mediated inflammatory diseases (IMIDs), Mixed connective tissue disease (MCTD), Other systemic rheumatic diseases (SRDs), Polimialgia reumática (PMR), Primary Sjögren’s Syndrome (pSS) , Psoriatic arthritis (PsA), Rheumatoid arthritis (RA), Spondyloarthritis (SpA), Systemic lupus erythematosus (SLE) , Systemic necrotising vasculitis (SNV), Systemic sclerosis (SSc), Systemic vasculitis (SV), Vasculitis (VAS); Medications: Biologic Disease-Modifying Antirheumatic Drugs(bDMARDs), Conventional synthetic DMARDs (csDMARDs) and Targeted synthetic DMARDs (tsDMARDs).* | | | | | | | |

| **Table 2 - Description of flare** | | | | | |
| --- | --- | --- | --- | --- | --- |
| **Reference** | **IMRD** | **Inactivated vaccine** | **Evaluation type of flare up** | **Patients with inactivated vaccine** | **Control group** |
|  |  |  |  | **n symptom flare up** | **n symptom flare up** |
| Abu-Shakra et al., 2000 | SLE | Influenza Virus Vaccine Monovalent (H1N1) | NI | NI | NI |
| Acharya et al., 2019 | RA, SLE, VAS and Pss | Adjuvanted Herpes Zoster Subunit Vaccine (HZ/su) | Events of underlying rheumatic disease flare ups | 4 | NI |
| Acharya et al., 2019 | RA, SLE, VAS and Pss | Adjuvanted Herpes Zoster Subunit Vaccine (HZ/su) | Events of underlying rheumatic disease flare ups | 4 | NI |
| Adler et al., 2012 | MCTD, RA, SpA and VAS | Adjuvanted split influenza A/H1N1 vaccine | NI | NI | NI |
| Baranova et al., 2022 | AS and PsA | 23-valent pneumococcal polysaccharide vaccine (PPV-23) | NI | NI | NI |
| Borba et al., 2011 | SLE | Novel split virion, inactivated, non-adjuvanted pandemic H1N1 influenza vaccine | NI | NI | NI |
| Brodman et al., 1978 | SLE | Influenza Virus Vaccine Monovalent (H1N1) | NI | NI | NI |
| Buhler et al., 2019 | RA, PsA, VAS | dT (diphtheria and tetanus) vaccines | NI | NI | NI |
| Bukhanova et al., 2018 (1) | AIRD | Inactivated split-vaccine against influenza | NI | NI | NI |
| Bukhanova et al., 2018 (2) | RA | 23-valent pneumococcal polysaccharide vaccine (PPV-23) | NI | NI | NI |
| Bukhanova et al., 2019 (1) | RA and APS | Trivalent inactivated influenza vaccine | NI | NI | NI |
| Bukhanova et al., 2019 (2) | RA | 23-valent pneumococcal polysaccharide vaccine (PPV-23) | NI | NI | NI |
| Bukhanova et al., 2019 (2) | RA | 23-valent pneumococcal polysaccharide vaccine (PPV-23) | NI | NI | NI |
| Caporuscio et al., 2015 | RA | 13-valent pneumococcal vaccine (PCV-13) | NI | NI | NI |
| Caso et al., 2016 | PsA | Adjuvanted vaccine for seasonal influenza | NI | NI | NI |
| Claudino Formiga et al., 2021 | SLE | Influenza Virus Vaccine (H3N2) | NI | NI | NI |
| Colmegna et al., 2019 | RA | High-dose trivalent inactivated influenza vaccine (HD-TIV) and standard-dose quadrivalent influenza vaccine (SD-QIV) | NI | NI | NI |
| Crowe et al., 2011 | SLE | Trivalent inactivated influenza vaccine | SELENA-SLEDAI flare index - after 6 weeks | 14 | NI |
| Crowe et al., 2011 | SLE | Trivalent inactivated influenza vaccine | Mild/moderate | 10 | NI |
| Crowe et al., 2011 | SLE | Trivalent inactivated influenza vaccine | Severe flares | 4 | NI |
| Crowe et al., 2011 | SLE | Trivalent inactivated influenza vaccine | SELENA-SLEDAI flare index - after 12 weeks | 19 | NI |
| Crowe et al., 2011 | SLE | Trivalent inactivated influenza vaccine | Mild/moderate | 16 | NI |
| Crowe et al., 2011 | SLE | Trivalent inactivated influenza vaccine | Severe flares | 3 | NI |
| Crowe et al., 2011 | SLE | Trivalent inactivated influenza vaccine | Flaring at 6 weeks | 10 | NI |
| Del Porto et al., 2006 | SLE and RA | Trivalent inactivated influenza vaccine | NI | NI | NI |
| Dhar et al., 2014 | SLE | Quadrivalent human papillomavirus (HPV) vaccine | NI | NI | NI |
| Dhar et al., 2017 | SLE | Quadrivalent human papillomavirus (HPV) vaccine | NI | NI | NI |
| Eberhardson et al., 2017 | MCTD, SSc, PsA, RA and SLE | Inactivated Varicella Zoster Virus Vaccine | NI | NI | NI |
| Elkayam et al., 2002 (1) | RA, SLE, PsA and AS | Influenza Virus Vaccine Monovalent (H1N1) | NI | NI | NI |
| Elkayam et al., 2002 (2) | RA | Hepatitis B vaccine (HBV) | NI | NI | NI |
| Elkayam et al., 2005 | SLE | 23-valent pneumococcal polysaccharide vaccine (PPV-23) | NI | NI | NI |
| Fomin et al., 2005 | RA | Trivalent inactivated influenza vaccine | NI | NI | NI |
| Furer et al., 2020 | PsA | Seasonal influenza vaccine(H1N1, H3N2, B) | NI | NI | NI |
| Gabay et al., 2011 | RA, SpA, PsA, SLE, ANCA-AAV or SRDs or AIRD | Adjuvanted split influenza A/H1N1 vaccine | NI | NI | NI |
| Grabar et al., 2017 | SLE | 23-valent PPSV vaccines targeting various serotypes with adjuvant | NI | NI | NI |
| Hernandez et al., 2015 | SLE | Influenza and/ or pneumococcal vaccinations | NI | NI | NI |
| Herron et al., 1979 | SLE, RA and SRDs | Bivalent influenza virus vaccine | Systemic lupus erythematosus - Increased joint symptoms | 1 | 1 |
| Herron et al., 1979 | SLE, RA and SRDs | Bivalent influenza virus vaccine | Increased joint symptoms and altered affect | 1 | NI |
| Herron et al., 1979 | SLE, RA and SRDs | Bivalent influenza virus vaccine | Vasculitis (skin) | 1 | NI |
| Herron et al., 1979 | SLE, RA and SRDs | Bivalent influenza virus vaccine | Increased nephritis (4 mo) | 1 | NI |
| Herron et al., 1979 | SLE, RA and SRDs | Bivalent influenza virus vaccine | Other rheumatic diseases | 3 | NI |
| Herron et al., 1979 | SLE, RA and SRDs | Bivalent influenza virus vaccine | Rheumatoid arthritis | 6 | NI |
| Herron et al., 1979 | SLE, RA and SRDs | Bivalent influenza virus vaccine | Rheumatoid arthritis (4 mo) | 7 | NI |
| Herron et al., 1979 | SLE, RA and SRDs | Bivalent influenza virus vaccine | Degenerative joint disease | 0 | NI |
| Holvast et., 2009 | SLE | Trivalent influenza subunit vaccine | NI | NI | NI |
| Intongkam et al., 2018 | RA | Hepatitis B vaccine (HBV) | NI | NI | NI |
| Jarrett et al., 1980 | SLE | 14-valent pneumococcal polysaccharide (PPV-14) | NI | NI | NI |
| Jeffs et al., 2015 | ANCA- AAV | Trivalent inactivated influenza vaccine | NI | NI | NI |
| Källmark et al., 2020 | RA | Adjuvanted Herpes Zoster Subunit Vaccine (HZ/su) | NI | NI | NI |
| Karsh et al., 1980 | pSS | Polyvalent pneumococcal polysaccharide vaccine | NI | NI | NI |
| Klippel et al., 1979 | SLE | Polyvalent pneumococcal polysaccharide vaccine | NI | NI | NI |
| Kostianovsky et al., 2010 | SV, SSc, SLE and pSS | Seasonal influenza vaccine (H1N1) | Polyneuritis in a CSS patient | 2 | NI |
| Kostianovsky et al., 2010 | SV, SSc, SLE and pSS | Seasonal influenza vaccine (H1N1) | Temporally unrelated AID flares | 4 | NI |
| Kostianovsky et al., 2012 | SNV,SSc and pSS | Trivalent inactivated influenza vaccine | AID flares - related | 6 | NI |
| Kostianovsky et al., 2012 | SNV,SSc and pSS | Trivalent inactivated influenza vaccine | AID flares - unrelated | 13 | NI |
| Kuruma et al., 2007 | SLE | Hepatitis B vaccine (HBV) | Flared two months after the second dose with cutaneous manifestation (rash and alopecia) associated with a decrease in complement levels and anti-dsDNA antibodies | 1 | NI |
| Kuruma et al., 2007 | SLE | Hepatitis B vaccine (HBV) | Flared five days and two weeks after the third dose. The former had a mild photosensitivity and discoid rash associated with a decrease incomplement levels with a good response to low-dose prednisone (10 mg/day). The latter had polyarthritis associated with a decrease in complement levels and required the introduction of prednisone (20 mg/day) and methotrexate (10 mg/week). | 2 | NI |
| Leaung et al., 2022 | AIRD | Herpes zoster vaccine (HZ) | NI | NI | NI |
| Lenfant et al., 2021 | RA, VAS, PMR, SLE, PsA, pSS, SpA, Myositis, SSc | Recombinant zoster vaccine (RZV) | Outbreaks after the second RZV dose, then 2,4, 10 and 10 months respectively. | 4 | NI |
| Lenfant et al., 2021 | RA, VAS, PMR, SLE, PsA, pSS, SpA, Myositis, SSc | Recombinant zoster vaccine (RZV) | Flare in subgroups | 59 | NI |
| Lenfant et al., 2021 | RA, VAS, PMR, SLE, PsA, pSS, SpA, Myositis, SSc | Recombinant zoster vaccine (RZV) | RA | 21 | NI |
| Lenfant et al., 2021 | RA, VAS, PMR, SLE, PsA, pSS, SpA, Myositis, SSc | Recombinant zoster vaccine (RZV) | Vasculitis | 5 | NI |
| Lenfant et al., 2021 | RA, VAS, PMR, SLE, PsA, pSS, SpA, Myositis, SSc | Recombinant zoster vaccine (RZV) | PMR | 5 | NI |
| Lenfant et al., 2021 | RA, VAS, PMR, SLE, PsA, pSS, SpA, Myositis, SSc | Recombinant zoster vaccine (RZV) | SLE | 4 | NI |
| Lenfant et al., 2021 | RA, VAS, PMR, SLE, PsA, pSS, SpA, Myositis, SSc | Recombinant zoster vaccine (RZV) | PsA | 4 | NI |
| Lenfant et al., 2021 | RA, VAS, PMR, SLE, PsA, pSS, SpA, Myositis, SSc | Recombinant zoster vaccine (RZV) | Inflammatory arthritis | 3 | NI |
| Lenfant et al., 2021 | RA, VAS, PMR, SLE, PsA, pSS, SpA, Myositis, SSc | Recombinant zoster vaccine (RZV) | Sjögren | 2 | NI |
| Lenfant et al., 2021 | RA, VAS, PMR, SLE, PsA, pSS, SpA, Myositis, SSc | Recombinant zoster vaccine (RZV) | SpA | 2 | NI |
| Lenfant et al., 2021 | RA, VAS, PMR, SLE, PsA, pSS, SpA, Myositis, SSc | Recombinant zoster vaccine (RZV) | Myositis | 2 | NI |
| Lenfant et al., 2021 | RA, VAS, PMR, SLE, PsA, pSS, SpA, Myositis, SSc | Recombinant zoster vaccine (RZV) | Scleroderma | 0 | NI |
| Litinsky et al., 2012 | SSc | Trivalent influenza subunit vaccine | NI | NI | NI |
| Louie et al., 1978 | SLE | Bivalent influenza virus vaccine | NI | NI | NI |
| Lu et al., 2011 | SLE | Influenza Virus Vaccine Monovalent (H1N1) | NI | NI | NI |
| Lukina et al., 2022 | RA | 13-valent pneumococcal vaccine (PCV-13) | NI | NI | NI |
| Mathian et al., 2010 | SLE | Influenza Virus Vaccine Monovalent (H1N1) | NI | NI | NI |
| Miossi et al., 2011 | MCTD | Trivalent inactivated influenza vaccine | NI | NI | NI |
| Mok et al., 2011 | SLE | Quadrivalent human papillomavirus (HPV) vaccine | Mild/moderate lupus flare at month 0-2 | 1 | NI |
| Mok et al., 2011 | SLE | Quadrivalent human papillomavirus (HPV) vaccine | Mild/moderate lupus flares at month 3-6 | 2 | NI |
| Mok et al., 2011 | SLE | Quadrivalent human papillomavirus (HPV) vaccine | Mild/moderate lupus flares at month 7-12 | 7 | NI |
| Mok et al., 2013 | SLE | Quadrivalent human papillomavirus (HPV) vaccine | NI | NI | NI |
| Morgan et al., 2016 | SV | Pn 7-valent conjugate vaccine; Haemophilus influenza type b (Hib) and meningococcal (Men) | NI | NI | NI |
| Muravyeva et al., 2020 | AS | 23-valent pneumococcal vaccine (PPV-23) | NI | NI | NI |
| Muravyeva et al., 2021 | SpA | 23-valent pneumococcal polysaccharide vaccine (PPV-23) | NI | NI | NI |
| Naumtseva et al., 2015 | RA | 23-valent pneumococcal polysaccharide vaccine (PPV-23) | NI | NI | NI |
| Naumtseva et al., 2016 | RA | 23-valent pneumococcal polysaccharide vaccine (PPV-23) | NI | NI | NI |
| Pasoto et al., 2012 | pSS | Influenza Virus Vaccine Monovalent (H1N1) | NI | NI | NI |
| Pasoto et al., 2013 | pSS | Influenza Virus Vaccine Monovalent (H1N1) | Parotiditis - 1-Year prior vaccination | 3 | 0 |
| Pasoto et al., 2013 | pSS | Influenza Virus Vaccine Monovalent (H1N1) | Parotiditis - 1-Year after vaccination | 2 | NI |
| Pasoto et al., 2013 | pSS | Influenza Virus Vaccine Monovalent (H1N1) | Arthritis - 1-Year prior vaccination | 6 | NI |
| Pasoto et al., 2013 | pSS | Influenza Virus Vaccine Monovalent (H1N1) | Arthritis - 1-Year after vaccination | 2 | NI |
| Pasoto et al., 2013 | pSS | Influenza Virus Vaccine Monovalent (H1N1) | Cutaneous vasculitis - 1-Year prior vaccination | 1 | NI |
| Pasoto et al., 2013 | pSS | Influenza Virus Vaccine Monovalent (H1N1) | Cutaneous vasculitis - 1-Year after vaccination | 0 | NI |
| Pasoto et al., 2013 | pSS | Influenza Virus Vaccine Monovalent (H1N1) | Pneumonitis- 1-Year prior vaccination | 1 | NI |
| Pasoto et al., 2013 | pSS | Influenza Virus Vaccine Monovalent (H1N1) | Pneumonitis - 1-Year after vaccination | 2 | NI |
| Pasoto et al., 2023 | pSS | Bivalent influenza virus vaccine | NI | NI | NI |
| Pons et al., 2022 | RA | New recombinant vaccine effective against HZ | NI | NI | NI |
| Rezende et al., 2016 | SLE | 23-valent pneumococcal polysaccharide vaccine (PPV-23) | NI | NI | NI |
| Ristow et al., 1978 | SLE | Influenza Virus Vaccine Monovalent (H1N1) | NI | NI | NI |
| Ristow et al., 1978 | SLE | Influenza Virus Vaccine Monovalent (H1N1) | NI | NI | NI |
| Romão et al., 2020 | AIRD | Hepatitis B vaccine (HBV) | Disease flares, of which 7 were mild and did not require therapy adjustment | 14 | NI |
| Saad et al., 2011 | SLE, RA, AS, SSc, PsA, MCTD and pSS | Influenza Virus Vaccine Monovalent (H1N1) | NI | NI | NI |
| Sergeeva et al., 2017 | RA | 23-valent pneumococcal polysaccharide vaccine (PPV-23) | NI | NI | NI |
| Setti et al., 2009 | SSc | Trivalent inactivated influenza vaccine | NI | NI | NI |
| Stevens et al., 2019 | RA | Zoster Recombinant Adjuvanted (ZRA) vaccine | Incidence of flare following the first dose | 9 | NI |
| Stevens et al., 2019 | RA | Zoster Recombinant Adjuvanted (ZRA) vaccine | Incidence of flare following the second dose | 4 | NI |
| Stevens et al., 2019 | RA | Zoster Recombinant Adjuvanted (ZRA) vaccine | Flared after both the first and second dose | 1 | NI |
| Stevens et al., 2020 | RA and SRDs | Zoster Recombinant Adjuvanted (ZRA) vaccine | Flares total | 27 | NI |
| Stevens et al., 2020 | RA and SRDs | Zoster Recombinant Adjuvanted (ZRA) vaccine | After the first dose | 23 | NI |
| Stevens et al., 2020 | RA and SRDs | Zoster Recombinant Adjuvanted (ZRA) vaccine | After the second dose | 5 | NI |
| Stevens et al., 2020 | RA and SRDs | Zoster Recombinant Adjuvanted (ZRA) vaccine | Both doses | 1 | NI |
| Stevens et al., 2020 | RA and SRDs | Zoster Recombinant Adjuvanted (ZRA) vaccine | Patients with RA | 19 | NI |
| Stevens et al., 2020 | RA and SRDs | Zoster Recombinant Adjuvanted (ZRA) vaccine | Patients with an SRD | 9 | NI |
| Stojanovich L. (2006) | SLE and RA | Trivalent inactivated influenza vaccine | NI | NI | NI |
| Tarasova et al., 2016 | SLE | 23-valent pneumococcal polysaccharide vaccine (PPV-23) | NI | NI | NI |
| Tarasova et al., 2017 | SLE | 23-valent pneumococcal polysaccharide vaccine (PPV-23) | NI | NI | NI |
| Tarasova et al., 2018 | SLE | 23-valent pneumococcal polysaccharide vaccine (PPV-23) | NI | NI | NI |
| Tarasova et al., 2020 | SLE | 23-valent pneumococcal polysaccharide vaccine (PPV-23) | NI | NI | NI |
| Tarasova et al., 2021 (1) | SLE | 23-valent pneumococcal polysaccharide vaccine (PPV-23) | NI | NI | NI |
| Tarasova et al., 2021 (2) | APS | 23-valent pneumococcal polysaccharide vaccine (PPV-23) | NI | NI | NI |
| Tarasova et al., 2022 | VS | 23-valent pneumococcal polysaccharide vaccine (PPV-23) | NI | NI | NI |
| Tarján et al., 2002 | SLE | 23-valent pneumococcal polysaccharide vaccine (PPV-23) | NI | NI | NI |
| Vagelli et al., 2017 | SLE, RA, SSc, pSS, BD and Dermatomyositis | Influenza and/or meningococcal C vaccinations | NI | NI | NI |
| Venerito et al., 2023 | RA | Adjuvanted Recombinant Zoster Vaccine | NI | NI | NI |
| Wallin et al., 2009 | SLE | Trivalent inactivated influenza vaccine | NI | NI | NI |
| Wiesik-Szewczyk et al., 2010 | SLE | Trivalent inactivated influenza vaccine | NI | NI | NI |
| Williams et al., 1978 | SLE | Bivalent influenza virus vaccine | NI | NI | NI |
| Winthrop et al., 2019 | RA | 13-valent pneumococcal vaccine (PCV-13) | NI | NI | NI |
| Zhou et al., 2021 | pSS | Quadrivalent influenza vaccine (live attenuated) | NI | NI | NI |
| *Note : Immune-mediated rheumatic disease (IMRD); Ankylosing spondylitis (AS), Antineutrophil cytoplasmic antibody associated vasculitis (ANCA- AAV), Antiphospholipid syndrome (APS), Autoimmune inflammatory rheumatic diseases (AIRD), Behçet disease (BD), Immune-mediated inflammatory diseases (IMIDs), Mixed connective tissue disease (MCTD), Other systemic rheumatic diseases (SRDs), Polimialgia reumática (PMR), Primary Sjögren’s Syndrome (pSS) , Psoriatic arthritis (PsA), Rheumatoid arthritis (RA), Spondyloarthritis (SpA), Systemic lupus erythematosus (SLE) , Systemic necrotising vasculitis (SNV), Systemic sclerosis (SSc), Systemic vasculitis (SV) and Vasculitis (VAS); Medications: Biologic Disease-Modifying Antirheumatic Drugs(bDMARDs), Conventional synthetic DMARDs (csDMARDs) and Targeted synthetic DMARDs (tsDMARDs). *Items in gray were used in the meta-analysis.* | | | | | |

| **Table 3: Description of worsening disease activity** | | | | | | |
| --- | --- | --- | --- | --- | --- | --- |
| **Reference** | **IMRD** | **Inactivated vaccine** | **Patients with inactivated vaccine - Worsening of IMRD disease activity** | | **Control group - Worsening of IMRD disease activity** | |
|  |  |  | **Evaluation** | **n or mean (sd)** | **Evaluation** | **n or mean (sd)** |
| Abu-Shakra et al., 2000 | SLE | Influenza Virus Vaccine Monovalent (H1N1) | NI | NI | NI | NI |
| Acharya et al., 2019 | RA, SLE, VAS and Pss | Adjuvanted Herpes Zoster Subunit Vaccine (HZ/su) | NI | NI | NI | NI |
| Adler et al., 2012 | MCTD, RA, SpA and VAS | Adjuvanted split influenza A/H1N1 vaccine | NI | NI | NI | NI |
| Baranova et al., 2022 | AS and PsA | 23-valent pneumococcal polysaccharide vaccine (PPV-23) | NI | NI | NI | NI |
| Borba et al., 2011 | SLE | Novel split virion, inactivated, non-adjuvanted pandemic H1N1 influenza vaccine | Increase in SLEDAI | 12 | NI | NI |
| Borba et al., 2011 | SLE | Novel split virion, inactivated, non-adjuvanted pandemic H1N1 influenza vaccine | SLEDAI stable | 133 | NI | NI |
| Borba et al., 2011 | SLE | Novel split virion, inactivated, non-adjuvanted pandemic H1N1 influenza vaccine | Decrease in SLEDAI | 28 | NI | NI |
| Borba et al., 2011 | SLE | Novel split virion, inactivated, non-adjuvanted pandemic H1N1 influenza vaccine | High | 40 | NI | NI |
| Brodman et al., 1978 | SLE | Influenza Virus Vaccine Monovalent (H1N1) | NI | NI | NI | NI |
| Buhler et al., 2019 | RA, PsA, VAS | dT (diphtheria and tetanus) vaccines | Remission - RA | 74 | NI | NI |
| Buhler et al., 2019 | RA, PsA, VAS | dT (diphtheria and tetanus) vaccines | Low- RA | 17 | NI | NI |
| Buhler et al., 2019 | RA, PsA, VAS | dT (diphtheria and tetanus) vaccines | Moderate- RA | 30 | NI | NI |
| Buhler et al., 2019 | RA, PsA, VAS | dT (diphtheria and tetanus) vaccines | High- RA | 7 | NI | NI |
| Buhler et al., 2019 | RA, PsA, VAS | dT (diphtheria and tetanus) vaccines | Remission- RA | 39 | NI | NI |
| Buhler et al., 2019 | RA, PsA, VAS | dT (diphtheria and tetanus) vaccines | Remission - SpA/PsA | 11 | NI | NI |
| Buhler et al., 2019 | RA, PsA, VAS | dT (diphtheria and tetanus) vaccines | Low - SpA/PsA | 4 | NI | NI |
| Buhler et al., 2019 | RA, PsA, VAS | dT (diphtheria and tetanus) vaccines | Moderate - SpA/PsA | 7 | NI | NI |
| Buhler et al., 2019 | RA, PsA, VAS | dT (diphtheria and tetanus) vaccines | High -SpA/PsA | 1 | NI | NI |
| Buhler et al., 2019 | RA, PsA, VAS | dT (diphtheria and tetanus) vaccines | Remission - SpA/PsA | 42 | NI | NI |
| Bukhanova et al., 2018 (1) | AIRD | Inactivated split-vaccine against influenza | NI | NI | NI | NI |
| Bukhanova et al., 2018 (2) | RA | 23-valent pneumococcal polysaccharide vaccine (PPV-23) | NI | NI | NI | NI |
| Bukhanova et al., 2019 (1) | RA and APS | Trivalent inactivated influenza vaccine | NI | NI | NI | NI |
| Bukhanova et al., 2019 (2) | RA | 23-valent pneumococcal polysaccharide vaccine (PPV-23) | NI | NI | NI | NI |
| Caporuscio et al., 2015 | RA | 13-valent pneumococcal vaccine (PCV-13) | NI | NI | NI | NI |
| Caso et al., 2016 | PsA | Adjuvanted vaccine for seasonal influenza | Tender joints- BASELINE | 1.64±1.93 | tender joint count- T0 | 1.48±1.44 |
| Caso et al., 2016 | PsA | Adjuvanted vaccine for seasonal influenza | Tender joints - FOLLOW UP | 2.36± 2.34 | tender joint count- T3 | 2.04±2.61 |
| Caso et al., 2016 | PsA | Adjuvanted vaccine for seasonal influenza | Swollen joints- BASELINE | 0.12±0.33 | swollen joint count- T0 | 0.2±0.58 |
| Caso et al., 2016 | PsA | Adjuvanted vaccine for seasonal influenza | Swollen joints - FOLLOW UP | 0.12± 0.33 | swollen joint count- T3 | 0.12±0.44 |
| Caso et al., 2016 | PsA | Adjuvanted vaccine for seasonal influenza | BASDAI- BASELINE | 3.18±1.08 | BASDAI- T0 | 3.3±1.37 |
| Caso et al., 2016 | PsA | Adjuvanted vaccine for seasonal influenza | BASDAI - FOLLOW UP | 3.43± 1.6 | BASDAI- T3 | 2.9±1.21 |
| Caso et al., 2016 | PsA | Adjuvanted vaccine for seasonal influenza | BASFI- BASELINE | 2.99±1.67 | BASFI - T0 | 2.74±1.55 |
| Caso et al., 2016 | PsA | Adjuvanted vaccine for seasonal influenza | BASFI - FOLLOW UP | 3.35± 2.19 | BASFI - T3 | 2.47±1.66 |
| Caso et al., 2016 | PsA | Adjuvanted vaccine for seasonal influenza | MASES- BASELINE | 1.12±1.79 | MASES - T0 | 1.2±1.66 |
| Caso et al., 2016 | PsA | Adjuvanted vaccine for seasonal influenza | MASES - FOLLOW UP | 0.8± 1.04 | MASES - T3 | 0.96±1.4 |
| Caso et al., 2016 | PsA | Adjuvanted vaccine for seasonal influenza | PASI- BASELINE | 1.67±2.2 | PASI - T0 | 1.96±2.85 |
| Caso et al., 2016 | PsA | Adjuvanted vaccine for seasonal influenza | PASI - FOLLOW UP | 1.9± 2.99 | PASI - T3 | 1.98±2.78 |
| Claudino Formiga et al., 2021 | SLE | Influenza Virus Vaccine (H3N2) | NI | NI | NI | NI |
| Colmegna et al., 2019 | RA | High-dose trivalent inactivated influenza vaccine (HD-TIV) and standard-dose quadrivalent influenza vaccine (SD-QIV) | NI | NI | NI | NI |
| Crowe et al., 2011 | SLE | Trivalent inactivated influenza vaccine | Low | 8 | NI | NI |
| Del Porto et al., 2006 | SLE and RA | Trivalent inactivated influenza vaccine | NI | NI | NI | NI |
| Dhar et al., 2014 | SLE | Quadrivalent human papillomavirus (HPV) vaccine | NI | NI | NI | NI |
| Dhar et al., 2017 | SLE | Quadrivalent human papillomavirus (HPV) vaccine | NI | NI | NI | NI |
| Eberhardson et al., 2017 | MCTD, SSc, PsA, RA and SLE | Inactivated Varicella Zoster Virus Vaccine | NI | NI | NI | NI |
| Elkayam et al., 2002 (1) | RA, SLE, PsA and AS | Influenza Virus Vaccine Monovalent (H1N1) | DAS28 - RA - BASELINE | 4.4 ± 1.3 | NI | NI |
| Elkayam et al., 2002 (1) | RA, SLE, PsA and AS | Influenza Virus Vaccine Monovalent (H1N1) | DAS28 - RA - FOLLOW UP | 4.4 ± 1.3 | NI | NI |
| Elkayam et al., 2002 (1) | RA, SLE, PsA and AS | Influenza Virus Vaccine Monovalent (H1N1) | DAS28 - PsA- BASELINE | 3.2 ± 1.2 | NI | NI |
| Elkayam et al., 2002 (1) | RA, SLE, PsA and AS | Influenza Virus Vaccine Monovalent (H1N1) | DAS28 - PsA - FOLLOW UP | 3.1 ± 1.3 | NI | NI |
| Elkayam et al., 2002 (1) | RA, SLE, PsA and AS | Influenza Virus Vaccine Monovalent (H1N1) | BASDAI - AS- BASELINE | 2.2±1.7 | NI | NI |
| Elkayam et al., 2002 (1) | RA, SLE, PsA and AS | Influenza Virus Vaccine Monovalent (H1N1) | BASDAI - AS - FOLLOW UP | 3.4± 2.4 | NI | NI |
| Elkayam et al., 2002 (1) | RA, SLE, PsA and AS | Influenza Virus Vaccine Monovalent (H1N1) | SLEDAI - SLE- BASELINE | 0.9±1 | NI | NI |
| Elkayam et al., 2002 (1) | RA, SLE, PsA and AS | Influenza Virus Vaccine Monovalent (H1N1) | SLEDAI - SLE - FOLLOW UP | 0.3± 0.7 | NI | NI |
| Elkayam et al., 2002 (2) | RA | Hepatitis B vaccine (HBV) | Daytime pain- BASELINE | 3.25±2.4 | Daytime pain - mean (SD) - week 0 | 3.8±2.1 |
| Elkayam et al., 2002 (2) | RA | Hepatitis B vaccine (HBV) | Daytime pain - FOLLOW UP | 2.6± 2.0 | Daytime pain - mean (SD) week 30 | 3.7±2.2 |
| Elkayam et al., 2002 (2) | RA | Hepatitis B vaccine (HBV) | Morning stiffness- BASELINE | 38.6±39.2 | Morning stiffness (min) - mean (SD) - week 0 | 57.7±59.1 |
| Elkayam et al., 2002 (2) | RA | Hepatitis B vaccine (HBV) | Morning stiffness - FOLLOW UP | 25.8± 26.1 | Morning stiffness (min) - mean (SD) - week 30 | 65.2±68 |
| Elkayam et al., 2002 (2) | RA | Hepatitis B vaccine (HBV) | Tender joints- BASELINE | 4.5±3.9 | No of tender joints - mean (SD) - week 0 | 5.9±6.1 |
| Elkayam et al., 2002 (2) | RA | Hepatitis B vaccine (HBV) | Tender joints - FOLLOW UP | 3.8± 4.0 | No of tender joints - mean (SD) - week 30 | 5.0±4.6 |
| Elkayam et al., 2002 (2) | RA | Hepatitis B vaccine (HBV) | Swollen joints- BASELINE | 2.6±2.1 | No of swollen joints - mean (SD) - week 0 | 3.36±4.0 |
| Elkayam et al., 2002 (2) | RA | Hepatitis B vaccine (HBV) | Swollen joints - FOLLOW UP | 1.7± 1.7 | No of swollen joints - mean (SD) - week 30 | 2.9±3.1 |
| Elkayam et al., 2002 (2) | RA | Hepatitis B vaccine (HBV) | CRP- BASELINE | 1.71±0.9 | CRP (mg/l) - mean (SD) - week 0 | 2.5±2.8 |
| Elkayam et al., 2002 (2) | RA | Hepatitis B vaccine (HBV) | CRP - FOLLOW UP | 1.0± 1.1 | CRP (mg/l) - mean (SD) - week 30 | 2.1±2.3 |
| Elkayam et al., 2002 (2) | RA | Hepatitis B vaccine (HBV) | ESR- BASELINE | 27.2±17.5 | ESR (mm/1st h) - mean (SD) - week 0 | 38±18.4 |
| Elkayam et al., 2002 (2) | RA | Hepatitis B vaccine (HBV) | ESR - FOLLOW UP | 28± 19.7 | ESR (mm/1st h) - mean (SD) - week 30 | 32.7±16.3 |
| Elkayam et al., 2005 | SLE | 23-valent pneumococcal polysaccharide vaccine (PPV-23) | Fatigue score- BASELINE | 1.5±0.5 | NI | NI |
| Elkayam et al., 2005 | SLE | 23-valent pneumococcal polysaccharide vaccine (PPV-23) | Fatigue score - FOLLOW UP | 1.3± 0.5 | NI | NI |
| Elkayam et al., 2005 | SLE | 23-valent pneumococcal polysaccharide vaccine (PPV-23) | ESR- BASELINE | 23±13.9 | NI | NI |
| Elkayam et al., 2005 | SLE | 23-valent pneumococcal polysaccharide vaccine (PPV-23) | ESR - FOLLOW UP | 22.2± 16.2 | NI | NI |
| Elkayam et al., 2005 | SLE | 23-valent pneumococcal polysaccharide vaccine (PPV-23) | CRP- BASELINE | 0.99±0.93 | NI | NI |
| Elkayam et al., 2005 | SLE | 23-valent pneumococcal polysaccharide vaccine (PPV-23) | CRP - FOLLOW UP | 0.94± 0.83 | NI | NI |
| Elkayam et al., 2005 | SLE | 23-valent pneumococcal polysaccharide vaccine (PPV-23) | SLEDAI- BASELINE | 4.41±2.92 | NI | NI |
| Elkayam et al., 2005 | SLE | 23-valent pneumococcal polysaccharide vaccine (PPV-23) | SLEDAI - FOLLOW UP | 4.47± 3.22 | NI | NI |
| Fomin et al., 2005 | RA | Trivalent inactivated influenza vaccine | Tender joints- BASELINE | 10.3±12.3 | NI | NI |
| Fomin et al., 2005 | RA | Trivalent inactivated influenza vaccine | Tender joints - FOLLOW UP | 9.1± 12.1 | NI | NI |
| Fomin et al., 2005 | RA | Trivalent inactivated influenza vaccine | Swollen joints- BASELINE | 2.2±3.7 | NI | NI |
| Fomin et al., 2005 | RA | Trivalent inactivated influenza vaccine | Swollen joints - FOLLOW UP | 1.5±5.1 | NI | NI |
| Furer et al., 2020 | PsA | Seasonal influenza vaccine(H1N1, H3N2, B) | Remission | 5 | NI | NI |
| Furer et al., 2020 | PsA | Seasonal influenza vaccine(H1N1, H3N2, B) | Low | 17 | NI | NI |
| Furer et al., 2020 | PsA | Seasonal influenza vaccine(H1N1, H3N2, B) | Moderate | 7 | NI | NI |
| Furer et al., 2020 | PsA | Seasonal influenza vaccine(H1N1, H3N2, B) | High | 2 | NI | NI |
| Gabay et al., 2011 | RA, SpA, PsA, SLE, ANCA-AAV or SRDs or AIRD | Adjuvanted split influenza A/H1N1 vaccine | NI | NI | NI | NI |
| Grabar et al., 2017 | SLE | 23-valent PPSV vaccines targeting various serotypes with adjuvant | NI | NI | NI | NI |
| Hernandez et al., 2015 | SLE | Influenza and/ or pneumococcal vaccinations | Increase in SLEDAI | 10 | NI | NI |
| Herron et al., 1979 | SLE, RA and SRDs | Bivalent influenza virus vaccine | NI | NI | NI | NI |
| Holvast et., 2009 | SLE | Trivalent influenza subunit vaccine | NI | NI | NI | NI |
| Intongkam et al., 2018 | RA | Hepatitis B vaccine (HBV) | NI | NI | NI | NI |
| Jarrett et al., 1980 | SLE | 14-valent pneumococcal polysaccharide (PPV-14) | Mild activation | 10 | NI | NI |
| Jeffs et al., 2015 | ANCA- AAV | Trivalent inactivated influenza vaccine | NI | NI | NI | NI |
| Källmark et al., 2020 | RA | Adjuvanted Herpes Zoster Subunit Vaccine (HZ/su) | NI | NI | NI | NI |
| Karsh et al., 1980 | pSS | Polyvalent pneumococcal polysaccharide vaccine | NI | NI | NI | NI |
| Klippel et al., 1979 | SLE | Polyvalent pneumococcal polysaccharide vaccine | NI | NI | NI | NI |
| Kostianovsky et al., 2010 | SV, SSc, SLE and pSS | Seasonal influenza vaccine (H1N1) | NI | NI | NI | NI |
| Kostianovsky et al., 2012 | SNV,SSc and pSS | Trivalent inactivated influenza vaccine | NI | NI | NI | NI |
| Kuruma et al., 2007 | SLE | Hepatitis B vaccine (HBV) | SLEDAI- BASELINE | 0.14±0.52 | NI | NI |
| Kuruma et al., 2007 | SLE | Hepatitis B vaccine (HBV) | Remission | 26 | NI | NI |
| Leaung et al., 2022 | AIRD | Herpes zoster vaccine (HZ) | NI | NI | NI | NI |
| Lenfant et al., 2021 | RA, VAS, PMR, SLE, PsA, pSS, SpA, Myositis, SSc | Recombinant zoster vaccine (RZV) | NI | NI | NI | NI |
| Litinsky et al., 2012 | SSc | Trivalent influenza subunit vaccine | NI | NI | NI | NI |
| Louie et al., 1978 | SLE | Bivalent influenza virus vaccine | NI | NI | NI | NI |
| Lu et al., 2011 | SLE | Influenza Virus Vaccine Monovalent (H1N1) | NI | NI | NI | NI |
| Lukina et al., 2022 | RA | 13-valent pneumococcal vaccine (PCV-13) | NI | NI | NI | NI |
| Mathian et al., 2010 | SLE | Influenza Virus Vaccine Monovalent (H1N1) | NI | NI | NI | NI |
| Miossi et al., 2011 | MCTD | Trivalent inactivated influenza vaccine | NI | NI | NI | NI |
| Mok et al., 2011 | SLE | Quadrivalent human papillomavirus (HPV) vaccine | NI | NI | NI | NI |
| Mok et al., 2013 | SLE | Quadrivalent human papillomavirus (HPV) vaccine | NI | NI | NI | NI |
| Morgan et al., 2016 | SV | Pn 7-valent conjugate vaccine; Haemophilus influenza type b (Hib) and meningococcal (Men) | NI | NI | NI | NI |
| Muravyeva et al., 2020 | AS | 23-valent pneumococcal vaccine (PPV-23) | NI | NI | NI | NI |
| Muravyeva et al., 2021 | SpA | 23-valent pneumococcal polysaccharide vaccine (PPV-23) | NI | NI | NI | NI |
| Naumtseva et al., 2015 | RA | 23-valent pneumococcal polysaccharide vaccine (PPV-23) | NI | NI | NI | NI |
| Naumtseva et al., 2016 | RA | 23-valent pneumococcal polysaccharide vaccine (PPV-23) | NI | NI | NI | NI |
| Pasoto et al., 2012 | pSS | Influenza Virus Vaccine Monovalent (H1N1) | NI | NI | NI | NI |
| Pasoto et al., 2013 | pSS | Influenza Virus Vaccine Monovalent (H1N1) | NI | NI | NI | NI |
| Pasoto et al., 2023 | pSS | Bivalent influenza virus vaccine | NI | NI | NI | NI |
| Pons et al., 2022 | RA | New recombinant vaccine effective against HZ | NI | NI | NI | NI |
| Rezende et al., 2016 | SLE | 23-valent pneumococcal polysaccharide vaccine (PPV-23) | NI | NI | NI | NI |
| Ristow et al., 1978 | SLE | Influenza Virus Vaccine Monovalent (H1N1) | NI | NI | NI | NI |
| Romão et al., 2020 | AIRD | Hepatitis B vaccine (HBV) | NI | NI | NI | NI |
| Saad et al., 2011 | SLE, RA, AS, SSc, PsA, MCTD and pSS | Influenza Virus Vaccine Monovalent (H1N1) | NI | NI | NI | NI |
| Sergeeva et al., 2017 | RA | 23-valent pneumococcal polysaccharide vaccine (PPV-23) | NI | NI | NI | NI |
| Setti et al., 2009 | SSc | Trivalent inactivated influenza vaccine | NI | NI | NI | NI |
| Stevens et al., 2019 | RA | Zoster Recombinant Adjuvanted (ZRA) vaccine | NI | NI | NI | NI |
| Stevens et al., 2020 | RA and SRDs | Zoster Recombinant Adjuvanted (ZRA) vaccine | NI | NI | NI | NI |
| Stojanovich L. (2006) | SLE and RA | Trivalent inactivated influenza vaccine | NI | NI | NI | NI |
| Tarasova et al., 2016 | SLE | 23-valent pneumococcal polysaccharide vaccine (PPV-23) | NI | NI | NI | NI |
| Tarasova et al., 2017 | SLE | 23-valent pneumococcal polysaccharide vaccine (PPV-23) | High | 1 | NI | NI |
| Tarasova et al., 2017 | SLE | 23-valent pneumococcal polysaccharide vaccine (PPV-23) | Low | 20 | NI | NI |
| Tarasova et al., 2017 | SLE | 23-valent pneumococcal polysaccharide vaccine (PPV-23) | Moderate | 4 | NI | NI |
| Tarasova et al., 2017 | SLE | 23-valent pneumococcal polysaccharide vaccine (PPV-23) | Remission | 5 | NI | NI |
| Tarasova et al., 2018 | SLE | 23-valent pneumococcal polysaccharide vaccine (PPV-23) | NI | NI | NI | NI |
| Tarasova et al., 2020 | SLE | 23-valent pneumococcal polysaccharide vaccine (PPV-23) | NI | NI | NI | NI |
| Tarasova et al., 2021 (1) | SLE | 23-valent pneumococcal polysaccharide vaccine (PPV-23) | Low | 33 | NI | NI |
| Tarasova et al., 2021 (1) | SLE | 23-valent pneumococcal polysaccharide vaccine (PPV-23) | Remission | 6 | NI | NI |
| Tarasova et al., 2021 (1) | SLE | 23-valent pneumococcal polysaccharide vaccine (PPV-23) | Moderate | 12 | NI | NI |
| Tarasova et al., 2021 (1) | SLE | 23-valent pneumococcal polysaccharide vaccine (PPV-23) | High | 9 | NI | NI |
| Tarasova et al., 2021 (1) | SLE | 23-valent pneumococcal polysaccharide vaccine (PPV-23) | Moderate exacerbation | 7 | NI | NI |
| Tarasova et al., 2021 (2) | APS | 23-valent pneumococcal polysaccharide vaccine (PPV-23) | NI | NI | NI | NI |
| Tarasova et al., 2022 | VS | 23-valent pneumococcal polysaccharide vaccine (PPV-23) | NI | NI | NI | NI |
| Tarján et al., 2002 | SLE | 23-valent pneumococcal polysaccharide vaccine (PPV-23) | NI | NI | NI | NI |
| Vagelli et al., 2017 | SLE, RA, SSc, pSS, BD and Dermatomyositis | Influenza and/or meningococcal C vaccinations | NI | NI | NI | NI |
| Venerito et al., 2023 | RA | Adjuvanted Recombinant Zoster Vaccine | Remission - JAK | 14 | NI | NI |
| Venerito et al., 2023 | RA | Adjuvanted Recombinant Zoster Vaccine | Remission -bDMARDs | 9 | NI | NI |
| Venerito et al., 2023 | RA | Adjuvanted Recombinant Zoster Vaccine | DAS28 remission, n - baseline- JAKi | 10 | NI | NI |
| Venerito et al., 2023 | RA | Adjuvanted Recombinant Zoster Vaccine | DAS28,remission, n - At 2nd Shot- JAKi | 11 | NI | NI |
| Venerito et al., 2023 | RA | Adjuvanted Recombinant Zoster Vaccine | DAS28, remission, n -3-Month Follow-Up- JAKi | 14 | NI | NI |
| Venerito et al., 2023 | RA | Adjuvanted Recombinant Zoster Vaccine | DAS28 remission, n - baseline- bDMARDs | 9 | NI | NI |
| Venerito et al., 2023 | RA | Adjuvanted Recombinant Zoster Vaccine | DAS28,remission, n - At 2nd Shot- bDMARDs | 9 | NI | NI |
| Venerito et al., 2023 | RA | Adjuvanted Recombinant Zoster Vaccine | DAS28, remission, n -3-Month Follow-Up- bDMARDs | 9 | NI | NI |
| Wallin et al., 2009 | SLE | Trivalent inactivated influenza vaccine | SLEDAI- BASELINE | 1.19±2 | A/Wiscosin/67/2005 - Antibody level - before | 1059±1114 |
| Wallin et al., 2009 | SLE | Trivalent inactivated influenza vaccine | SLEDAI - FOLLOW UP | 1.6± 2.6 | A/Wiscosin/67/2005 - Antibody level - after | 1888±108.3 |
| Wiesik-Szewczyk et al., 2010 | SLE | Trivalent inactivated influenza vaccine | NI | NI | NI | NI |
| Williams et al., 1978 | SLE | Bivalent influenza virus vaccine | NI | NI | NI | NI |
| Winthrop et al., 2019 | RA | 13-valent pneumococcal vaccine (PCV-13) | NI | NI | NI | NI |
| Zhou et al., 2021 | pSS | Quadrivalent influenza vaccine (live attenuated) | ESSDAI- BASELINE | 1.18±0.33 | NI | NI |
| Zhou et al., 2021 | pSS | Quadrivalent influenza vaccine (live attenuated) | ESSDAI - FOLLOW UP | 0.75± 0.21 | NI | NI |
| Zhou et al., 2021 | pSS | Quadrivalent influenza vaccine (live attenuated) | ESSPRI - FOLLOW UP | 1.86± 0.52 | NI | NI |
| *Note: Immune-mediated rheumatic disease(IMRD); Ankylosing spondylitis (AS), Antineutrophil cytoplasmic antibody associated vasculitis (ANCA- AAV), Antiphospholipid syndrome (APS), Autoimmune inflammatory rheumatic diseases (AIRD), Behçet disease (BD), Immune-mediated inflammatory diseases (IMIDs), Mixed connective tissue disease (MCTD), Other systemic rheumatic diseases (SRDs), Polimialgia reumática (PMR), Primary Sjögren’s Syndrome (pSS) , Psoriatic arthritis (PsA), Rheumatoid arthritis (RA), Spondyloarthritis (SpA), Systemic lupus erythematosus (SLE) , Systemic necrotising vasculitis (SNV), Systemic sclerosis (SSc), Systemic vasculitis (SV) and Vasculitis (VAS); Medications: Biologic Disease-Modifying Antirheumatic Drugs(bDMARDs), Conventional synthetic DMARDs (csDMARDs) and Targeted synthetic DMARDs (tsDMARDs). *Items in gray were used in the meta-analysis.* | | | | | | |

**QUESTION 2. What is the risk of infection and  symptom flare up or worsening of IMRD disease activity after vaccination with live attenuated vaccine (LAV)?**

**Table 1** - Characteristics of included studies.

| **Reference** | **Location** | **Journal** | **Design** | **Acronym** | **Drugs use** | **Live attenuated vaccine** | **Patients number** | **Follow-up** |
| --- | --- | --- | --- | --- | --- | --- | --- | --- |
| Casagrande *et al.,* 2022 | Espírito Santo, Brazil | Vaccines | Cohort prospective | SS | Synthetic DMARDs (non-steroidal anti-inflammatory drugs [17.6%, n = 9], prednisone < 20 mg/day [2.0%, n = 1], methotrexate < 20 mg/week [33.3%, n = 17], lefluno mide [7.8%, n = 4] or sulfasalazine [19.6%, n = 10]), and 49% (n = 25) with a history of prior biological immunotherapy (BIO-IT) (adali mumab [8.0%, n = 4], etanercept [10.0%, n = 5] or infliximab [31.0%, n = 16]) | 17DD-YF vaccine [yellow fever vaccine ] | 74  43♀️  31♂️  51 with IMRD disease  23 healthy control | 4 weeks |
| dos Reis *et al.,* 2022 | São Paulo, Brazil | Clinical Rheumatology | Case series | RA | 10 patients received  lefunomide, 7 received methotrexate, 6 received biologic  DMARDs (2 infiximab, 2 tocilizumab, 1 abatacept, 1 rituxi mab), 6 received prednisone (doses from 2.5 to 10 mg/day;  median 5.0 mg/day), and 1 received tofacitinib | 17DD-YF vaccine [yellow fever vaccine ] - a single  dose of the YF 17DD vaccine | 12  9 ♀️  3 ♂️ | 89 weeks |
| Tonacio *et al.,* 2021 | São Paulo, Brazil | PLOS NEGLECTED TROPICAL DISEASES | Cohort prospective | SLE, SLC and Chronic Inflammatory Diseases | Hydroxychloroquine 66 (41.5); Prednisone 25 (15.7); Methotrexate 24 (15.1); Leflunomide 14 (8.8); Sulfasalazine 6 (3.8) | 17DD-YF vaccine [yellow fever vaccine ] - a single  dose of the YF 17DD vaccine | 318  272 ♀️  46 ♂️ | 4 weeks |
| Costa-Rocha *et al.,* 2021 | Espírito Santo, Brazil | Scientific Reports | Cross-sectional | RA, SS, AS, SSc, SLE | NI | 17DD-YF vaccine [yellow fever vaccine ] - a single  dose of the YF 17DD vaccine | 161 | NA |
| Elkayam *et al.,* 2019 | Israel | Arthritis Rheumatol (abstract) | Multicenter | RA | Conventional disease-modifying anti rheumatic drugs (cDMARD) and/or low-dose glucocorticoids (GC), candidates to receive biologics or tofacitinib. | Live attenuated HZ vaccine | 167 | 6 weeks |
| Valim *et al.,* 2019 | Brazil | Ann. Rheum. Dis (abstract) | Multicenter | RA, SS, AS, SSc, SLE | NI | 17DD (Biomanguinhos-FIOCRUZ) YF vaccine | 190 | 28 days /  4 weeks |
| Koh *et al.,* 2018 | Seoul, South Korea | The Journal of Rheumatology | Cohort prospective | RA and AO | All patients with RA received DMARD, and 44% received combination DMARD therapy: MTX and leflunomide (n = 8; 19.5%), MTX and hydroxychloroquine (n = 8;19.5%), or MTX and sulfasalazine (n = 2; 4.9%). Low-dose GC (prednisolone or equivalent dose ≤ 7.5 g/day) were prescribed for 61% of patients with RA. | Live attenuated HZ vaccine | 69  66 ♀️  3 ♂️ | 12 weeks |
| Lessardi *et al.,* 2013 | Oklahoma City, USA | Ann. Rheum. Dis (abstract) | Pilot open-label | SLE | NI | Live attenuated HZ vaccine | 20 | 14 days |
| Chakarvarty *et al.,* 2013 | Oklahoma City, USA | Ann. Rheum. Dis (abstract) | Pilot open-label | SLE | Azathioprine, methotrexate, anti-malarials, and ≤ 10 mg prednisone daily. | Zostavax vaccine | 20  20♀️  0 ♂️ | 12 weeks |
| Zhang *et al.,* 2011 | United States | Arthritis Research & Therapy | Cohort retrospective | RA, PsA, PsO, inflammatory bowel diseases and AS | TNF antagonists (etanercept; adalimumab; infliximab), biologiscs and conventional DMARDS, oral glucocorticoids | Live attenuated HZ vaccine | 44115  27443 ♀️  16672 ♂️ | 4 weeks |
| Mok *et al.,* 2018 | Hong Kong, China | Annals of the rheumatic diseases (abstract) | RCT | SLE | NI | HZ vaccine (Zostavax) | 90  81♀️  9 ♂️ | 6 weeks |
| Calabrese *et al.,* 2020 | Ohio, China | Arthritis Care & Research | RCT | RA | Tofacitinib 5 mg twice daily (BID), tofacitinib 5 mg BID plus MTX, or ADA 40 mg  every other week plus MTX (1:1:1 randomization). | Live Zoster Vaccine | 1146  950 ♀️  196 ♂️ | 42 days |
| Muniz *et al.,* 2014 | Brasilia, Brazil | Revista Brasileira Reumatologia | Cohort prospective | RA | 55 (80%) MTX, 18 (26%) anti-malarials, 17 (25%) leflunomide, 8 (11%) sulfasalazine, 13 (19%) prednisone, 6 (8.8%) infliximabe, 1 (1.4%) etanercepte, 2 (2.9%) adalimumabe, 4 (5.8%) rituximabe, 2 (2.9%) abatacepte | 6 (8,8%) contra tríplice viral (SCR – sarampo, catapora e rubéola), 30 (44%) com a dT, 30 (44%) contra febre amarela, 15 (22%) contra hepatite B, 29 (42%) contra gripe, 42 (61,7%) contra influenza H1N1, quatro (5,8%) contra pneumonia, um (1,4%) contra meningite, um (1,4%) contra va | 68 |  |
| Guthridge *et al.,* 2013 | Oklahoma City, USA | J Rheumatol. | pilot open-label | SLE | Prednisone ≤ 10 mg daily; hydroxychloroquine ≤ 6.5 mg/kg daily; methotrexate ≤ 20 mg weekly; or azathioprine ≤ 150 mg daily. | Varicella zoster virus (VZV) | 20  20 ♀️  0 ♂️ | 12 weeks |
| Zhang et al., 2012 | Birmingham | JAMA | Cohort retrospective | RA, PsO,PsA, inflamaroty bowel diseases and AS | Anti-TNF biologics (adalimumab, etanercept, infliximab, certolizumab, and golimumab), non-TNF biologics (abatacept and rituximab), non-biologic immunosuppressive medications (or disease modifying antirheumatic drugs [DMARDs]) (methotrexate, hydroxycholoroquine, sulfasalazine, azathioprine, leflunomide, cyclosporine, and 6-mercaptopurine), and oral glucocorticoids. Exposure to biologics and non-biologic DMARDs was determined based on prescription date and days of supply and for infusions, the recommended dosing intervals. | Vaccination for Herpes Zoster | 663 | 42 days |
| Grabar *et al.,* 2017 | French | Vaccines | RCT | SLE | Mycophenolate mofetil, azathioprine | 23-valent PPS vaccines targeting serotypes 1, 2, 3, 4, 5, 6B, 7F, 8, 9N, 9V, 10A, 11A, 12F, 14, 15B, 17F, 18C, 19F, 19A, 20, 22F, 23F and 33F of Streptococcus pneumoniae | 42  37 ♀️  5 ♂️ | 52 weeks |

*Note: SS – Spondyloarthritis; RA - Rheumatoid Arthritis; SLE - Systemic Lupus Erythematosous ; SLC - Lupus Cutaneous; SS - Sjögren’s Syndrome; AO – Osteoarthrithis; AS - Ankylosing Spondylitis; PsA - Psoriatic arthitis, PsO – Psoriasis ; NI – not informated ; NA – Not applicable ; RCT – randomised clinical trial.*

**Table 2 -** Characteristics about the risk of infection and  symptom flare up of IMRD disease activity after vaccination with live attenuated vaccine (LAV)

| **Reference** | **Immune-mediated**  **rheumatic disease** | **LAV** | **N**  **intervention group** | **Flare up**  **(patients with inactivated vaccine)** | **N**  **control group** | **Flare**  **up (control group)** |
| --- | --- | --- | --- | --- | --- | --- |
| Elkayam et al., 2019 | RA | HZ vaccine | 132 | 67 | 35 | 12 |
| Koh et al., 2018 | RA and AO | HZ vaccine | 69 | 39 | NI | NI |
| Lessardi et al., 2013 | SLE | HZ vaccine | 10 | 0 | 10 | 0 |
| Chakarvarty et al., 2013 | SLE | HZ vaccine | 10 | 0 | 10 | 0 |
| Zhang et al., 2011 | RA, PsO,PsA, inflammatory bowel diseases and AS | HZ vaccine | 551 | 5 | 43564 | NI |
| Mok et al., 2018 | SLE | HZ vaccine | 45 | 0 | 45 | 0 |
| Calabrese et al., 2020 | RA | HZ vaccine | 216 | 3 | 930 | 15 |
| Grabar et al., 2017 | SLE | 23-valent PPS vaccines | 17 | 2 | 25 | 4 |

*Note: LAV - live attenuated vaccine; SS – Spondyloarthritis; RA - Rheumatoid Arthritis; SLE - Systemic Lupus Erythematosous ; SLC - Lupus Cutaneous; SS - Sjögren’s Syndrome; AO – Osteoarthrithis; AS - Ankylosing Spondylitis; PsA - Psoriatic arthitis, PsO – Psoriasis ; NI – not informated ; NA – Not applicable ; RCT – randomised clinical trial.*

**Table 3** - Characteristics about worsing of IMRD disease activity after vaccination with live attenuated vaccine (LAV)

| **Reference** | **Immune-mediated rheumatic disease** | **LAV** | **N intervention**  **group** | **Evaluation worsing** | **worsening (intervention group)** | **N control group** | **worsening**  **(control group)** |
| --- | --- | --- | --- | --- | --- | --- | --- |
| Casagrande et  al., 2022 | SS | 17DD-YF vaccine [yellow fever vaccine] | 51 | Geometric means (95%CI) of YF-specific  neutralizing antibodies test (PRNT) *baseline* | 5.5 (5.1-6.0) | 23 | 6.9 (5.4-8.7) |
| Casagrande et  al., 2022 | SS | 17DD-YF vaccine [yellow fever vaccine] | 51 | Geometric means  (95%CI) of YF-specific neutralizing antibodies test (PRNT) *follow-up*  *(day 28)* | 112 (73-170) | 23 | 440 (291-667) |
| Casagrande et  al., 2022 | SS | 17DD-YF vaccine [yellow fever vaccine] | 51 | Geometric means (95%CI) of BASDAI < 4 *baseline* | 5.7 (5.0-6.4) | 23 | - |
| Casagrande et  al., 2022 | SS | 17DD-YF vaccine [yellow fever vaccine] | 51 | Geometric means (95%CI) of BASDAI < 4 - *follow-up (day 28)* | 118 (72-193) | 23 | - |
| Casagrande et  al., 2022 | SS | 17DD-YF vaccine [yellow fever vaccine] | 51 | Geometric means (95%CI) of BASDAI > 4 - *baseline* | 5.4 (4.7-8.2) | 23 | - |
| Casagrande et  al., 2022 | SS | 17DD-YF vaccine [yellow fever vaccine] | 51 | Geometric means (95%CI) of BASDAI > 4 - *follow-up (day 28)* | 92 (35-239) | 23 | - |
| Casagrande et  al., 2022 | SS | 17DD-YF vaccine [yellow fever vaccine] | 51 | BASDAI Scores - *baseline* | 2.7 (0.0 - 8.8) | 23 | - |
| Casagrande et  al., 2022 | SS | 17DD-YF vaccine [yellow fever vaccine] | 51 | BASDAI Scores - *follow-up* | 2.8 (0.0 - 7.6) | 23 | - |
| dos Reis et al., 2022 | RA | 17DD-YF vaccine [yellow fever vaccine] | 12 | DAS-28 | 7 | - | - |
| Tonacio et al., 2021 | SLE (n=65), SLC (n=2) and Chronic Inflammatory Diseases | 17DD-YF vaccine [yellow fever vaccine ] | 159 | DAS-28 | 118 | 120 | 116 |
| Elkayam et al., 2019 | RA | HZ vaccine | 132 | DAS-28 | 7 | 35 | 0 |
| Valim et al., 2019 | RA (n=47), SS (n=36), AS (n=48), Systemic Sclerosis (n=8), SLE (n=24) | 17DD-YF vaccine [yellow fever vaccine ] | 161 | DAS-28 | 33 | 29 | 21 |
| Koh et al., 2018 | RA and AO | HZ vaccine | 69 | Low activity | 2 | - | - |
| Chakarvarty et al., 2013 | SLE | HZ vaccine | 10 | SLEDAI score (MEAN) | 1.1 | 10 |  |
| Muniz et al., 2014 | RA | Mixed | 29 | DAS28 - baseline | 3,65+-1,64 | 39 | DAS28 - baseline  3,65+-1,64 follow-up: 3,6 ± 1,6 |
|  |  |  |  | DAS28 - follow-up | 3,6 ± 1,7 |  |  |
| Guthridge et al., 2013 | SLE | varicella zoster  virus (VZV) | 10 | SLEDAI score  < 4 (mean) | 0 | 10 |  |
| Grabar et al., 2017 | SLE | 23-valent PPS vaccine | 17 | SLEDAI score > 4 (MEAN) | 3 | 25 | 7 |

*Note: LAV - live attenuated vaccine; SS – Spondyloarthritis; RA - Rheumatoid Arthritis; SLE - Systemic Lupus Erythematosous ; SLC - Lupus Cutaneous; SS - Sjögren’s Syndrome; AO – Osteoarthrithis; AS - Ankylosing Spondylitis; PsA - Psoriatic arthitis, PsO – Psoriasis ; NI – not informated ; NA – Not applicable ; RCT – randomised clinical trial.*

**QUESTION 03. What is the impact of the cs-DMARDs (MTX, LEF, SZL, HXQ) use on the response to inactivated vaccines in patients with immune-mediated rheumatic diseases?**

Table 1. Main characteristics of the included studies.

| **Reference** | **Local** | **Journal** | **Study Design** | **Immune-mediated rheumatic disease** | **cs-DMARDs (MTX, LEF, SZL, HXQ)** | **Inactivated vaccine** | **Follow-up**  **(weeks)** |
| --- | --- | --- | --- | --- | --- | --- | --- |
| O'Dell et al., 1996 | USA (United States), North America | Clinical rheumatology | Clinical trial | RA | MTX | Pneumococcal vaccine | 6 |
| Abu-Shakara et al., 2002 | Israel, Asia (also considered part of the Middle East) | Journal of Rheumatology | Clinical trial | SLE | HXQ, MTX | Inactivated split-vaccine against influenza | 12 |
| Adler et al., 2012 | France, Europe | Rheumatology | Cohort | AR, EpA, SV or CTD | MTX | Influenza vaccine A/H1N1 | 26 |
| Andrade et al., 2012 | Brazil, South America | Rheumatology | Clinical trial | SSc | MTX | Trivalent subunit influenza vaccine | 6 |
| Askling et al., 2014 | Sweden, Europe | Travel medicine and infectious disease | Cohort | RA | MTX | Hepatitis A vaccine | 104 |
| Barros et al., 2017 | Brazil, South America | Journal of Rheumatology | Cohort | SSc | MTX | H1N1 vaccine, monovalent, non-adjuvanted, inactivated, split-virus vaccine | 3 |
| Bingham et al., 2016 | USA (United States), North America | Annals of the Rheumatic Diseases | Clinical trial | RA | MTX | 23-valent pneumococcal polysaccharide vaccine | 8 |
| Borba et al., 2012 | Brazil, South America | Rheumatology | Cohort | SLE | MTX, HXQ | Influenza vaccine A/H1N1 | 4 |
| Broyde et al., 2016 | Israel, Asia (also considered part of the Middle East) | Journal of Rheumatology | Cohort | RA, PsA, AS or IBD associated SpA, BD, TA and JRA | MTX | 23-valent pneumococcal polysaccharide vaccine |  |
| Bühler et al., 2019 | Switzerland, Europe | Rheumatology | Cohort | RA, axial SpA/PsA, VS (BD, ANCA-AAV) | MTX | Diphtheria/tetanus vaccine | 13 |
| Bukhanova et al., 2018 | Russia, Asia (partly in Europe and partly in Asia) | Annals of the Rheumatic Diseases | Clinical trial | RA | MTX, LEF | 23-valent pneumococcal polysaccharide vaccine | 209 |
| Bukhanova et al., 2019 | Russia, Asia (partly in Europe and partly in Asia) | Annals of the Rheumatic Diseases | Clinical trial | RA and AS | MTX, SLZ | Trivalent subunit influenza vaccine | 26 |
| Bukhanova et al., 2019 | Russia, Asia (partly in Europe and partly in Asia) | Annals of the Rheumatic Diseases | Clinical trial | RA | MTX | 23-valent pneumococcal polysaccharide vaccine | 260 |
| Bukhanova et al., 2012 | Russia, Asia (partly in Europe and partly in Asia) | Clinical and Experimental Rheumatology | Clinical trial | RA, AS and SSD | MTX | Inactivated split-vaccine against influenza | 26 |
| Bukhanova et al., 2018 | Russia, Asia (partly in Europe and partly in Asia) | Annals of the Rheumatic Diseases | Clinical trial | RA, AS and SSD | MTX, SZL, LEF | Inactivated split-vaccine against influenza | 26 |
| Caporuscio et al., 2018 | Italy, Europe | Clinical Immunology | Clinical trial | RA | MTX | 13-valent pneumococcal conjugate vaccine | 26 |
| Caporuscio et al., 2015 | Italy, Europe | Annals of the Rheumatic Diseases | Clinical trial | RA | MTX | 13-valent pneumococcal conjugate vaccine | 26 |
| Colmegna et al., 2019 | Canada, North America | Lancet Rheumatology | Clinical trial | RA | MTX | Influenza (trivalent x quadrivalent) | 52 |
| De Juanes et al., 2012 | Spain, Europe | Annals of the Rheumatic Diseases | Clinical trial | PsA | MTX | Hepatitis B virus vaccine |  |
| Dhar et al., 2017 | USA (United States), North America | Vaccine | Cohort | SLE | HXQ | Quadrivalent HPV vaccine, Gardasil is a virus like particle vaccine | 26 |
| Elmér et al., 2022 | Sweden, Europe | Journal of Immunology Research | Cohort | RA | MTX | 13-valent pneumococcal conjugate vaccine | 12 |
| Fomin et al., 2006 | Israel, Asia (also considered part of the Middle East) | Annals of the Rheumatic Diseases | Clinical trial | RA | MTX, LEF, HXQ, SZL | Influenza vaccine | 6 |
| França et al., 2012 | Brazil, South America | Rheumatology | Cohort | RA | MTX, LEF, HXQ, SZL | H1N1 vaccine, monovalent, non-adjuvanted, inactivated, split-virus vaccine | 3 |
| Furer et al., 2020 | Israel, Asia (also considered part of the Middle East) | Vaccine | Clinical trial | PsA | MTX | Sanofi Pasteur vaccine | 4 |
| Gabay et al., 2011 | Switzerland, Europe | Arthritis & Rheumatology | Cohort | RA, SpA and SRDs | MTX, LEF, HXQ, SZL | Adjuvanted split influenza A/09/H1N1 vaccine | 24 |
| Garcia et al., 2022 | Netherlands, Europe | Vaccines | Cohort | RA, Pso, PsA, SA, NAD, SRDs, Crohn’s, and UC | MTX | Combination 13-valent pneumococcal conjugate vaccine and 23-valent pneumococcal polysaccharide vaccine | 173 |
| Gelinck et al., 2008 | Netherlands, Europe | Vaccine | Clinical trial | RA and Crohn's | MTX | 23-valent pneumococcal polysaccharide vaccine | 4 |
| Hesselstrand et al., 2018 | Sweden, Europe | Rheumatology | Clinical trial | SSc | HXQ | Combination 13-valent pneumococcal conjugate vaccine and 23-valent pneumococcal polysaccharide vaccine | 6 |
| Intongkam et al., 2019 | Thailand, Asia | Clinical rheumatology | Clinical trial | RA | MTX, LEF, SZL | Hepatitis B virus vaccine | 32 |
| Jain et al., 2015 | India, Asia | Journal of Rheumatic Diseases | Clinical trial | RA | MTX | Trivalent seasonal influenza vaccine | 4 |
| Kapetanovic et al., 2006 | Sweden, Europe | Rheumatology | Clinical trial | RA | MTX | Pneumococcal vaccine | 6 |
| Kapetanovic et al., 2007 | Sweden, Europe | Rheumatology | Clinical trial | RA | MTX | Influenza vaccine | 6 |
| Kapetanovic et al., 2011 | Sweden, Europe | Arthritis and rheumatism | Cohort | RA or SpA | MTX | 7-valent pneumococcal conjugate vaccine | 6 |
| Kapetanovic et al., 2011 | Sweden, Europe | Clinical rheumatology | Clinical trial | RA | MTX | Combination 23-valent pneumococcal polysaccharide vaccine and 7-valent pneumococcal conjugate vaccine | 6 |
| Kapetanovic et al., 2014 | Sweden, Europe | Arthritis Research & Therapy | Cohort | RA | MTX | Influenza vaccine A/H1N1 | 22 |
| Kapetanovic et al., 2017 | Sweden, Europe | Vaccine | Clinical trial | RA | MTX | 13-valent pneumococcal conjugate vaccine | 6 |
| Kapetanovic et al., 2012 | Sweden, Europe | Arthritis Research & Therapy | Cohort | RA or SpA | MTX | 7-valent pneumococcal conjugate vaccine | 6 |
| Kifayat et al., 2022 | USA (United States), North America | American College of Rheumatology | Clinical trial | RA, PsA, and SLE | MTX | Herpes zoster |  |
| Kim et al., 2018 | South Korea, Asia | Arthritis & Rheumatology | Clinical trial | RA | MTX | Seasonal influenza vaccination | 2 |
| Kivitz et al., 2014 | USA (United States), North America | The Journal of rheumatology | Clinical trial | RA | MTX | 23-valent pneumococcal polysaccharide vaccine and trivalent influenza virus vaccine | 6 |
| Kogure et al., 2014 | Japan, Asia | Clinical rheumatology | Cohort | RA | MTX | Influenza vaccine | 4 |
| Kumar et al., 2020 | Denmark, Europe | Annals of the Rheumatic Diseases | Cohort | AIIRD | MTX | Influenza vaccine | 60 |
| Lakota et al., 2015 | Slovenia, Europe | Annals of the Rheumatic Diseases | Clinical trial | RA | MTX | Trivalent seasonal influenza vaccine | 25 |
| Litinsky et al., 2012 | Israel, Asia (also considered part of the Middle East) | Arthritis & Rheumatology | Cohort | SSc | MTX | Trivalent subunit influenza vaccine | 6 |
| Measeet et al., 2004 | USA (United States), North America | Journal of Rheumatology | Clinical trial | PsA | MTX | 23-valent pneumococcal polysaccharide vaccine | 4 |
| Migita et al., 2015 | Japan, Asia | Arthritis Research & Therapy | Clinical trial | RA | MTX | 23-valent pneumococcal polysaccharide vaccine | 6 |
| Migita et al., 2015 | Japan, Asia | Medicine | Clinical trial | RA | MTX | 23-valent pneumococcal polysaccharide vaccine | 6 |
| Mori et al., 2012 | Japan, Asia | Annals of the Rheumatic Diseases | Clinical trial | RA | MTX | Influenza vaccine | 6 |
| Mori et al., 2013 | Japan, Asia | Annals of the Rheumatic Diseases | Clinical trial | RA | MTX | Pneumococcal vaccine | 6 |
| Mori et al., 2022 | Japan, Asia | Modern rheumatology | Clinical trial | RA | MTX | 13-valent pneumococcal conjugate vaccine | 26 |
| Muravyeva et al., 2020 | Russia, Asia (partly in Europe and partly in Asia) | Annals of the Rheumatic Diseases | Clinical trial | AS | MTX, SLZ | 23-valent pneumococcal polysaccharide vaccine | 60 |
| Muravyeva et al., 2021 | Russia, Asia (partly in Europe and partly in Asia) | Annals of the Rheumatic Diseases | Clinical trial | SpA | MTX, SLZ | 23-valent pneumococcal polysaccharide vaccine | 52 |
| Nagel et al., 2017 | Denmark, Europe | Lupus | Clinical trial | SLE | HXQ | 13-valent pneumococcal conjugate vaccine | 6 |
| Nagel et al., 2023 | Sweden, Europe | Vaccine | Clinical trial | [RA, SpA and PsA](https://www.sciencedirect.com/topics/medicine-and-dentistry/spondyloarthropathy) | MTX | 7-valent pneumococcal conjugate vaccine | 521 |
| Naumtseva et al., 2016 | Russia, Asia (partly in Europe and partly in Asia) | International Journal of Rheumatic Diseases | Clinical trial | RA | MTX,LEF | 23-valent pneumococcal polysaccharide vaccine | 104 |
| Naumtseva et al., 2015 | Russia, Asia (partly in Europe and partly in Asia) | Annals of the Rheumatic Diseases | Clinical trial | RA | MTX | 23-valent pneumococcal polysaccharide vaccine | 56 |
| Nguyen et al., 2017 | Denmark, Europe | The Journal of rheumatology | Clinical trial | RA | MTX, SZL, HXQ | Combination 13-valent pneumococcal conjugate vaccine and 23-valent pneumococcal polysaccharide vaccine | 24 |
| Nived et al., 2016 | Sweden, Europe | Annals of the Rheumatic Diseases | Clinical trial | GPA, CSV, GCV, TA and SRDs | MTX | 13-valent pneumococcal conjugate vaccine | 6 |
| Nived et al., 2017 | Sweden, Europe | Vaccine | Clinical trial | SV | MTX | 13-valent pneumococcal conjugate vaccine | 6 |
| Nived et al., 2018 | Sweden, Europe | BMC Rheumatology | Clinical trial | RA and pSS | MTX | 13-valent pneumococcal conjugate vaccine | 8 |
| Nived et al., 2020 | Sweden, Europe | Arthritis Research & Therapy | Clinical trial | AIIRD | MTX | Combination 13-valent pneumococcal conjugate vaccine and 23-valent pneumococcal polysaccharide vaccine | 8 |
| Nived et al., 2021 | Sweden, Europe | Scientific Reports | Clinical trial | RA | MTX | 13-valent pneumococcal conjugate vaccine | 12 |
| Park et al., 2017 | South Korea, Asia | Annals of the Rheumatic Diseases | Clinical trial | RA | MTX, LEF, HXQ, SZL | Trivalent influenza vaccine (H1N1, H3N2 and B-Yamagata) | 4 |
| Park et al., 2018 | South Korea, Asia | Annals of the Rheumatic Diseases | Clinical trial | RA | MTX, LEF, HXQ, SZL | Quadrivalent seasonal influenza vaccine containing H1N1, H3N2, B-Yamagata and B-Victoria. | 4 |
| Park et al., 2018 | South Korea, Asia | Annals of the Rheumatic Diseases | Clinical trial | RA | MTX, LEF, HXQ, SZL | Quadrivalent seasonal influenza vaccine containing H1N1, H3N2, B-Yamagata and B-Victoria. | 6 |
| Park et al., 2019 | South Korea, Asia | Annals of the Rheumatic Diseases | Clinical trial | RA | HXQ, LEF, MTX | Quadrivalent seasonal influenza vaccine containing H1N1, H3N2, B-Yamagata and B-Victoria. | 6 |
| Park et al., 2022 | South Korea, Asia | [Arthritis & Rheumatology](https://acrjournals.onlinelibrary.wiley.com/journal/23265205) | Clinical trial | RA | MTX | Quadrivalent seasonal influenza vaccine containing H1N1, H3N2, B-Yamagata and B-Victoria. | 16 |
| Pelka et al., 2021 | Poland, Europe | Dermatologic Therapy | Cohort | SLE | HXQ | Pneumococcal vaccine | 6 |
| Rákóczi et al., 2014 | Hungary, Europe | Annals of the Rheumatic Diseases | Cohort | RA | MTX | 13-valent pneumococcal conjugate vaccine | 8 |
| Rákóczi et al., 2016 | Hungary, Europe | Joint Bone Spine | Clinical trial | RA and OA | MTX | 23-valent pneumococcal polysaccharide vaccine | 8 |
| Rasmussen et al., 2020 | Denmark, Europe | Scandinavian Journal of Rheumatology | Clinical trial | RA, SpA or PsA | MTX | 23-valent pneumococcal polysaccharide vaccine | 521 |
| Rasmussen et al., 2018 | Denmark, Europe | Annals of the Rheumatic Diseases | Coss-sectional | RA, Spa, PsA or JRA | MTX | 23-valent pneumococcal polysaccharide vaccine | 26 |
| Rasmussen et al., 2023 | Denmark, Europe | Scandinavian Journal of Rheumatology | Clinical trial | AIIRD | MTX | 23-valent pneumococcal polysaccharide vaccine | 13 |
| Ribeiro et al., 2011 | Brazil, South America | Annals of the Rheumatic Diseases | Clinical trial | RA | MTX, HXQ, LEF | Influenza vaccine A/H1N1 | 23 |
| Ribeiro et al., 2013 | Brazil, South America | Arthritis Care & Research | Clinical trial | RA | MTX | Influenza vaccine A/H1N1 | 24 |
| Romão et al., 2020 | Portugal, Europe | Annals of the Rheumatic Diseases | Clinical trial | AR, PsA/AS, and SRDs | MTX, SZL, LEF | Hepatitis B virus vaccine | 26 |
| Rosdahl et al., 2018 | Sweden, Europe | Travel Medicine and Infectious Disease | Clinical trial | RA | MTX | Hepatitis A vaccine | 4 |
| Szabó et al., 2016 | Hungary, Europe | Clinical and Experimental Rheumatology | Clinical trial | SLE and pSS | MTX | Pneumococcal vaccine | 4 |
| Tarasova et al., 2017 | Russia, Asia (partly in Europe and partly in Asia) | Annals of the Rheumatic Diseases | Clinical trial | SLE | MTX | 23-valent pneumococcal polysaccharide vaccine | 43 |
| Tarasova et al., 2017 | Russia, Asia (partly in Europe and partly in Asia) | Annals of the Rheumatic Diseases | Clinical trial | SLE | cs-DMARD | 23-valent pneumococcal polysaccharide vaccine | 56 |
| Tarasova et al., 2018 | Russia, Asia (partly in Europe and partly in Asia) | Annals of the Rheumatic Diseases | Clinical trial | SLE | HXQ | 23-valent pneumococcal polysaccharide vaccine | 56 |
| Tarasova et al., 2019 | Russia, Asia (partly in Europe and partly in Asia) | Annals of the Rheumatic Diseases | Clinical trial | SLE | HXQ, MTX | 23-valent pneumococcal polysaccharide vaccine | 52 |
| Tarasova et al., 2020 | Russia, Asia (partly in Europe and partly in Asia) | Antibiotiki i Khimioterapiya | Clinical trial | SLE | HXQ | 23-valent pneumococcal polysaccharide vaccine | 56 |
| Tarasova et al., 2020 | Russia, Asia (partly in Europe and partly in Asia) | Annals of the Rheumatic Diseases | Clinical trial | SLE | HXQ | 23-valent pneumococcal polysaccharide vaccine | 52 |
| Tsuru et al., 2014 | Japan, Asia | Modern Rheumatology | Clinical trial | RA or CD | MTX | Inactivated split-vaccine against influenza | 12 |
| Van Assen et al., 2010 | Netherlands, Europe | Arthritis Research & Therapy | Clinical trial | RA | MTX | Trivalent subunit influenza vaccine | 43 |
| Visvanathan et al., 2007 | USA (United States), North America | The Journal of rheumatology | Clinical trial | RA | MTX | Pneumococcal vaccine | 34 |
| Wallin et al., 2009 | Brazil, South America | Acta Reumatológica Portuguesa | Case-control | SLE | MTX | Influenza vaccine | 6 |
| Westra et al., 2014 | Netherlands, Europe | Clinical and experimental immunology | Clinical trial | RA | MTX | Trivalent subunit influenza vaccine | 43 |
| Winthrop et al., 2019 | USA (United States), North America | Arthritis Research & Therapy | Clinical trial | RA | MTX | 13-valent pneumococcal conjugate vaccine | 12 |
| Zhilyaev et al., 2022 | Russia, Asia (partly in Europe and partly in Asia) | Annals of the Rheumatic Diseases | Cohort | RA | MTX | Combination 13-valent pneumococcal conjugate vaccine and 23-valent pneumococcal polysaccharide vaccine | 45 |

*Note:Ankylosing spondylitis (AS), Antineutrophil cytoplasmic antibody associated vasculitis (ANCA- AAV), Autoimmune inflammatory rheumatic diseases (AIIRD), Behçet disease (BD), Castleman’s disease (CD), Churg-Strauss vasculitis (CSV), Connective tissue diseases (CTD), Crohn’s disease (Crohn’s), Giant Cell Casculitis (GCV), granulomatosis with polyangiitis (GPA), Juvenile rheumatoid arthritis (JRA), Neurological autoimmune disease (NAD), Osteoarthritis (OA), Other systemic rheumatic diseases (SRDs), Primary Sjögren’s Syndrome (pSS), Psoriasis (Pso), Psoriatic arthritis (PsA), Rheumatoid arthritis (RA), Spondyloarthritis (SpA), Systemic lupus erythematosus (SLE), Systemic Scleroderma (SSD), Systemic sclerosis (SSc), Systemic vasculitis (SV), Takayasu arteritis (TA), Ulcerative colitis (UC); Medications: Methotrexate (MTX), Hydroxychloroquine (HQ), Sulfasalazine (SLZ), Leflunomide (LEF).*

**QUESTION 4. What is the impact of the immunosuppressants “azathioprine, mycophenolate mofetil, cyclosporine, cyclophosphamide’’ use on the response to inactivated vaccine in patients with immune-mediated rheumatic diseases?**

| **Table 1. Main characteristics of the included studies** | | | | | | | | |
| --- | --- | --- | --- | --- | --- | --- | --- | --- |
| **Reference** | **Local** | **Title** | **Journal** | **Study design** | **IMRD** | **Drugs AZA, MMF, CYC, CP** | **Inactivated vaccine** | **Follow-up (weeks)** |
| Abu-Shakra et al., 2002 | Israel, Asia | Influenza Virus Vaccination of Patients with SLE: Effects on Generation of Autoantibodies | Clinical Rheumatology | Case control | SLE | Immunosuppressant without specification | Inactivated influenza vírus | 12 |
| Acharya et al., 2019 | USA, North America | Safety of adjuvanted herpes zoster subunit vaccine (HZ/SU, Shingrix) among patients with autoimmune inflammatory diseases | Arthritis and Rheumatology | Randomized clinical trial | RA, SLE, VAS and pSS | AZA | Zoster recombinant adjuvanted (ZRA) | NI |
| Adler et al., 2012 | Switzerland, Europe | Protective effect of A/H1N1 vaccination in immune-mediated disease—a prospectively controlled vaccination study | Rheumatology | Cohort | MCTD, RA, SpA and VAS | AZA,MMF, CYC and CP | Adjuvanted split influenza A/H1N1 vaccine | 26 |
| Andrade et al., 2012 | Brazil, South America | Is the H1N1 influenza vaccine safe and effective in patients with SSc? | Rheumatology | Randomized clinical trial | SSc | Immunosuppressant without specification | Trivalent seasonal flu vaccine with A/California/7/2009/H1N1-like virus | 3 |
| Assen et al., 2010 | Netherlands, Europe | Humoral Responses After Influenza Vaccination Are Severely Reduced in Patients With Rheumatoid Arthritis Treated With Rituximab | Arthritis & Rheumatism | Randomized clinical trial | RA | AZA | Trivalent influenza subunit vaccine | 52 |
| Battafarano et al., 1998 | USA, North America | Antigen-specific antibody responses in lupus patients following immunization | Arthritis and Rheumatism | Randomized clinical trial | SLE | AZA, CP | Tetanus toxoid | 12 |
| Borba et al., 2011 | Brazil, South America | The novel pandemic influenza a (H1N1) 2009 vaccine: low rate of seroconversion after vaccination in SLE | Lupus | Randomized clinical trial | SLE | Immunosuppressant without specification | Novel split virion, inactivated, non-adjuvanted pandemic H1N1 influenza vaccine | 52 |
| Borba et al., 2012 | Brazil, South America | Influenza A/H1N1 vaccination of patients with SLE: Can antimalarial drugs restore diminished response under immunosuppressive therapy? | Rheumatology (United Kingdom) | Cohort | SLE | AZA,MMF and CYC | Monovalent, unadjuvanted, inactivated split-virus pandemic H1N1 influenza vaccine | 3 |
| Brodman et al., 1978 | USA, North America | Influenza vaccine response in systemic lupus erythematosus | Annals of Internal Medicine | Cohort | SLE | AZA | Influenza Virus Vaccine Monovalent, Type A | 4 |
| Buhler et al., 2019 | Switzerland, Europe | Safety and immunogenicity of tetanus/diphtheria vaccination in patients with rheumatic diseases—a prospective multi-centre cohort study | Rheumatology | Cohort | RA, SpA, PsA, VAS, BD and ANCA-AAV | AZA | dT (diphtheria and tetanus) vaccine | 52 |
| Eberhardson et al., 2017 | USA, North America | Safety and Immunogenicity of Inactivated Varicella-Zoster Virus Vaccine in Adults with Autoimmune Disease: A Phase 2, Randomized, Double-Blind, Placebo-Controlled Clinical Trial |  | Randomized clinical trial | MCTD, SSc, PsA, RA and SLE | Immunosuppressant without specification | Inactivated Varicella Zoster Virus Vaccine | 4 |
| Elkayam et al., 2002 | Israel, Asia | Immunogenicity and safety of pneumococcal vaccination in patients with rheumatoid arthritis or systemic lupus erythematosus | Clinical Infectious Diseases | Randomized clinical trial | RA and SLE | AZA, CYC | 23-valent pneumococcal polysaccharide vaccine (PPV23) | 9 |
| Elkayam et al., 2002 (2) | Israel, Asia | Safety and efficacy of vaccination against hepatitis B in patients with rheumatoid arthritis | Annals of the Rheumatic Diseases | Randomized clinical trial | RA | AZA | Hepatitis B vaccine | 30 |
| Elkayam et al., 2005 | Israel, Asia | Pneumococcal vaccination of patients with systemic lupus erythematosus: Effects on generation of autoantibodies | Autoimmunity | Randomized clinical trial | SLE | CP | 23-valent pneumococcal polysaccharide vaccine (PPV23) | 9 |
| Fischer et al., 2015 | Italy, Europe | Pneumococcal polysaccharide vaccination in adults undergoing immunosuppressive treatment for inflammatory diseases - A longitudinal study | Arthritis Research and Therapy | Randomized clinical trial | SLE, dermatomyositis, MCTD, PMR and SSc | AZA,MMF, CYC and CP | 23-valent pneumococcal polysaccharide vaccine (PPV23) | 52 |
| França et al., 2012 | Brazil, South America | TNF blockers show distinct patterns of immune response to the pandemic influenza A H1N1 vaccine in inflammatory arthritis patients | Rheumatology (Oxford) | Cohort | RA, AS and PsA | AZA, CYC and MMF | Inactivated influenza vírus | NI |
| Gabay et al., 2011 | Italy, Europe | Impact of synthetic and biologic disease-modifying antirheumatic drugs on antibody responses to the AS03-adjuvanted pandemic influenza vaccine: A prospective, open-label, parallel-cohort, single-center study | Arthritis and Rheumatism | Cohort | RA, SpA, PsA, SLE, ANCA–AAV, MCTD and SRDs | AZA, CYC and MMF | Adjuvanted split influenza A/H1N1 vaccine | 24 |
| Garrido et al.,2022 | Amsterdam, Europe | Immunogenicity of the 13-Valent Pneumococcal Conjugate Vaccine (PCV13) Followed by the 23-Valent Pneumococcal Polysaccharide Vaccine (PPSV23) in Adults with and without Immunosuppressive Therapy | Vaccines | Cohort | RA, PsA and SpA | MMF | Combination of PCV13 and PPV23 | 52 |
| Gelinck et al., 2007 | Netherlands, Europe | The effect of anti-tumour necrosis factor a treatment on the antibody response to influenza vaccination | Annals of the Rheumatic Diseases | Cohort | RA, PsA and AS | AZA | Trivalent influenza subunit vaccine | 4 |
| Gelinck et al., 2008 | Netherlands, Europe | Synergistic immunosuppressive effect of anti-TNF combined with methotrexate on antibody responses to the 23 valent pneumococcal polysaccharide vaccine | Vaccine | Randomized clinical trial | RA | AZA | 23-valent pneumococcal polysaccharide vaccine (PPV23) | 4 |
| Grabar et al., 2017 | France, Europe | Pneumococcal vaccination in patients with systemic lupus erythematosus: A multicenter placebo-controlled randomized double-blind study | Vaccine | Randomized clinical trial | SLE | AZA and MMF | 23-valent pneumococcal polysaccharide vaccine (PPV23) | 52 |
| Hesselstrand et al., 2018 | Sweden, Europe | Immunogenicity and safety of pneumococcal vaccination in patients with systemic sclerosis | Rheumatology (United Kingdom) | Randomized clinical trial | SSc | AZA and MMF | Combination of PCV13 and PPV23 | 6 |
| Holvast et a., 2006 | Netherlands, Europe | Safety and efficacy of influenza vaccination in systemic lupus erythematosus patients with quiescent disease | Annals of the Rheumatic Diseases | Cohort | SLE | AZA | Trivalent influenza subunit vaccine | 9 |
| Holvast et., 2009 | Poland, Europe | Effect of a second, booster, influenza vaccination on antibody responses in quiescent systemic lupus erythematosus: an open, prospective, controlled study | Rheumatology (Oxford) | Cohort | SLE | AZA, CYC and MMF | Trivalent influenza subunit vaccine | 4 |
| Intongkam et al., 2018 | Thailand, Asia | Efficacy and Safety of Hepatitis B Vaccination in Rheumatoid Arthritis Patients Receiving Disease-Modifying Antirheumatic Drugs and/or Biologics Therapy | Journal of Clinical Rheumatology | Randomized clinical trial | RA | AZA | Hepatitis B vaccine | 24 |
| Jain et al., 2017 | India, Asia | Effect of disease-modifying antirheumatic drug therapy on immune response to trivalent influenza vaccine in rheumatoid arthritis | Indian Journal of Medical Research | Randomized clinical trial | RA | Immunosuppressant without specification | Trivalent seasonal flu vaccine with A/California/7/2009/H1N1-like virus | 17 |
| Jarrett et al., 1980 | USA, North America | Impaired response to pneumococcal vaccine in systemic lupus erythematosus | Arthritis and Rheumatism | Randomized clinical trial | SLE | AZA | 14 valent pneumococcal vaccine | 52 |
| Jeffs et al., 2015 | Australia, Oceania | Randomized trial investigating the safety and efficacy of influenza vaccination in patients with antineutrophil cytoplasmic antibody-associated vasculitis. | Nephrology (Carlton) | Randomized clinical trial | ANCA-AAV | AZA, CYC and MMF | Trivalent split influenza vaccine | 26 |
| Kapetanovic et al., 2006 | Sweden, Europe | Influence of methotrexate, TNF blockers and prednisolone on antibody responses to pneumococcal polysaccharide vaccine in patients with rheumatoid arthritis | Rheumatology | Randomized clinical trial | RA | AZA and CYC | 23-valent pneumococcal polysaccharide vaccine (PPV23) | 6 |
| Kapetanovic et al., 2011 | Sweden, Europe | Antibody Response Is Reduced Following Vaccination With 7-Valent Conjugate Pneumococcal Vaccine in Adult Methotrexate-Treated Patients With Established Arthritis, but Not Those Treated With Tumor Necrosis Factor Inhibitors | Arthritis & Rheumatism | Cohort | RA and SpA | Immunosuppressant without specification | 7-valent conjugate pneumococcal vaccine | 6 |
| Kuruma et al., 2007 | Brazil, South America | Safety and efficacy of hepatitis B vaccine in systemic lupus erythematosus | Lupus | Randomized clinical trial | SLE | Immunosuppressant without specification | Hepatitis B vaccine | 4 |
| Lenfant et al., 2021 | USA, North America | Safety of recombinant zoster vaccine: a retrospective study of 622 rheumatology patients | Rheumatology (Oxford) | Cohort | RA, VAS, PMR, SLE, PsA, Pss, SpA, Myositis and SSc | AZA, CYC and MMF | Zoster recombinant vaccine (ZRV) | 52 |
| Lipnick et al.,1985 | USA, North America | Pneumococcal immunization in patients with systemic lupus erythematosus treated with immunosuppressives | Rheumatology | Randomized clinical trial | SLE | Immunosuppressant without specification | 14 valent pneumococcal vaccine | 26 |
| Louie et al., 1978 | USA, North America | Clinical and antibody responses after influenza immunization in systemic lupus erythematosus. | Annals of Internal Medicine | Case series | SLE | AZA | Bivalent (non disrupted) vaccine prepared from influenza | 13 |
| Lu et al., 2011 | Taiwan, Asia | A/H1N1 influenza vaccination in patients with systemic lupus erythematosus: Safety and immunity | Vaccine | Cohort | SLE | AZA | Monovalent, unadjuvanted, inactivated split-virus pandemic H1N1 influenza vaccine | 26 |
| Mathian et al., 2011 | France, Europe | Factors influencing the efficacy of two injections of a pandemic 2009 influenza A (H1N1) non adjuvanted vaccine in systemic lupus erythematosus | Arthritis and Rheumatism | Cohort | SLE | AZA, MMF and CP | Monovalent, unadjuvanted, inactivated split-virus pandemic H1N1 influenza vaccine | 52 |
| Mercado et al., 2009 | Mexico, North America | Antibody Response to Pneumococcal Polysaccharide Vaccine in Systemic Sclerosis | The Journal of Rheumatology | Randomized clinical trial | SSc | CP | 23-valent pneumococcal polysaccharide vaccine (PPV23) | 52 |
| Miossi et al., 2011 | Brazil, South America | Influenza H1N1 vaccination in mixed connective tissue disease: Effectiveness and safety independent of disease or therapy |  | Randomized clinical trial | MCTD | Immunosuppressant without specification | Trivalent seasonal flu vaccine with A/California/7/2009/H1N1-like virus | 3 |
| Miossi et al., 2013 | Brazil, South America | Immunogenicity of influenza H1N1 vaccination in mixed connective tissue disease: effect of disease and therapy | Clinics (Sao Paulo) | Cohort | MCTD | AZA | Influenza A/California/7/2009/H1N1-like virus vaccine | 3 |
| Mischlinger et al., 2022 | Switzerland, Europe | Long-term persistence of antibodies after diphtheria/tetanus vaccination in immunosuppressed patients with inflammatory rheumatic diseases and healthy controls | Vaccine | Cohort | RA, SpA, VAS and PsA | Immunosuppressant without specification | dT (diphtheria and tetanus) vaccine | 834 |
| Mok et al., 2011 | China, Asia | Safety of a quadrivalent human papillomavirus (HPV) vaccine in patients with systemic lupus erythematosus | Arthritis and Rheumatism | Randomized clinical trial | SLE | Immunosuppressant without specification | Quadrivalent human papillomavirus (HPV) vaccine | 26 |
| Mok et al., 2013 | China, Asia | Immunogenicity and safety of a quadrivalent human papillomavirus vaccine in patients with systemic lupus erythematosus: A case-control study | Annals of the Rheumatic Diseases | Case control | SLE | AZA, CYC and MMF | Quadrivalent human papillomavirus (HPV) vaccine | 52 |
| Mok et al., 2018 | China, Asia | Long-term immunogenicity of a quadrivalent human papillomavirus vaccine in systemic lupus erythematosus | Vaccine | Cohort | SLE | AZA,MMF, CYC and CP | Quadrivalent human papillomavirus (HPV) vaccine | 261 |
| Morgan et al., 2016 | UK, Europe | Association of Low B Cell Count and IgG Levels with Infection, and Poor Vaccine Response with All-Cause Mortality in an Immunosuppressed Vasculitis Population | Arthritis Care and Research | Cohort | SV | AZA and MMF | 7-valent conjugate pneumococcal vaccine | 26 |
| Müller et al., 2013 | Switzerland, Europe | Efficient boosting of the antiviral T cell response in B cell-depleted patients with autoimmune rheumatic diseases following influenza vaccination | Clinical and Experimental Rheumatology | Cohort | AIRD | AZA, CYC and MMF | Influenza A/California/7/2009/H1N1-like virus vaccine | 26 |
| Nagel et al., 2017 | Switzerland, Europe | Treatment with belimumab in systemic lupus erythematosus does not impair antibody response to 13-valent pneumococcal conjugate vaccine | Lupus | Randomized clinical trial | SLE | AZA | 13-valent conjugated pneumococcal vaccine (PCV13) | 39 |
| Nguyen et al., 2017 | Southern Denmark, Europe | Initial serological response after prime-boost pneumococcal vaccination in rheumatoid arthritis patients: Results of a randomized controlled trial | Journal of Rheumatology | Randomized clinical trial | RA | Immunosuppressant without specification | Combination of PCV13 and PPV23 | 16 |
| Nived et al., 2016 | Switzerland, Europe | Immunogenicity of 13-valent pneumococcal conjugate vaccine in patients with vasculitis receiving standard of care therapy and controls | Annals of the Rheumatic Diseases | Randomized clinical trial | SV | AZA, MMF and CP | 13-valent conjugated pneumococcal vaccine (PCV13) | 6 |
| Nived et al., 2017 | Switzerland, Europe | Immune response to pneumococcal conjugate vaccine in patients with systemic vasculitis receiving standard of care therapy | Vaccine | Randomized clinical trial | SV | AZA, MMF and CP | 13-valent conjugated pneumococcal vaccine (PCV13) | 8 |
| Nived et al., 2020 | Sweden, Europe | Prime-boost vaccination strategy enhances immunogenicity compared to single pneumococcal conjugate vaccination in patients receiving conventional DMARDs, to some extent in abatacept but not in rituximab-treated patients | Arthritis Research & Therapy | Cohort | AIRD | AZA and MMF | Combination of PCV13 and PPV23 | 8 |
| Oren et al., 2008 | Israel, Asia | Vaccination against influenza in patients with rheumatoid arthritis: the effect of rituximab on the humoral response. | Annals of the Rheumatic Diseases | Randomized clinical trial | RA | AZA | Adjuvanted split influenza A/H1N1 vaccine | 13 |
| Pasoto et al., 2012 | Brazil, South America | Long-Term Changes in Autoantibody Profile After Pandemic Unadjuvanted Influenza A/H1N1 Vaccine in Sjogren’s Syndrome. | Arthritis and Rheumatism | Randomized clinical trial | pSS | Immunosuppressant without specification | Influenza A/California/7/2009/H1N1-like virus vaccine | 52 |
| Pasoto et al., 2013 | Brazil, South America | Short and long-term effects of pandemic unadjuvanted influenza A(H1N1)pdm09 vaccine on clinical manifestations and autoantibody profile in primary Sjögren's syndrome | Pediatric Rheumatology | Cohort | pSS | AZA and MMF | Monovalent, unadjuvanted, inactivated split-virus pandemic H1N1 influenza vaccine | 52 |
| Pasoto et al., 2023 | Brazil, South America | Robust immunogenicity to the H3N2 component of influenza A vaccine in primary Sjögren syndrome | Clinical Rheumatology | Randomized Clinical Trial | pSS | AZA and MMF | Non-adjuvanted inactivated influenza vaccine | 4 |
| Rasmussen et al., 2020 | North Denmark, Europe | Pneumococcal antibody protection in patients with autoimmune inflammatory rheumatic diseases with varying vaccination status | Scandinavian Journal of Rheumatology | Cross sectional | RA, SpA, PsA and VAS | Immunosuppressant without specification | 23-valent pneumococcal polysaccharide vaccine (PPV23) | 17 |
| Rezende et al., 2016 | Brazil, South America | Immunogenicity of pneumococcal polysaccharide vaccine in adult systemic lupus erythematosus patients undergoing immunosuppressive treatment | Lupus | Cohort | SLE | AZA, MMF and CP | 23-valent pneumococcal polysaccharide vaccine (PPV23) | 52 |
| Richi et al., 2021 | Spain, Europe | Impact of biological therapies on the immune response after pneumococcal vaccination in patients with autoimmune inflammatory diseases | Vaccines | Cohort | RA, SpA, PsA and MCTD | AZA and CYC | Combination of PCV13 and PPV23 | 4 |
| Ristow et al., 1978 | USA, North America | Influenza vaccination of patients with systemic lupus erythematosus | Annals of Internal Medicine | Randomized clinical trial | SLE | AZA and CP | Influenza Virus Vaccine Monovalent, Type A | 8 |
| Romão et al., 2020 | Portugal, Europe | Poor response to hepatitis b vaccination in rheumatic patients treated with biologic therapy-implications for clinical practice | Annals of the Rheumatic Diseases | Randomized clinical trial | RA | Immunosuppressant without specification | Hepatitis B vaccine | 26 |
| Saad et al., 2011 | Brazil, South America | Immunogenicity and safety of the 2009 non-adjuvanted influenza A/H1N1 vaccine in a large cohort of autoimmune rheumatic diseases | Annals of the Rheumatic Diseases | Cohort | RA, SLE, AS, SSc, pSS, PsA and MCTD | Immunosuppressant without specification | Monovalent, unadjuvanted, inactivated split-virus pandemic H1N1 influenza vaccine | 3 |
| Sampaio Barros et al., 2018 | Brazil, South America | Pandemic non-adjuvanted influenza A H1N1 vaccine in a cohort of patients with systemic sclerosis | Rheumatology (United Kingdom) | Cohort | SSc | AZA, CYC and MMF | Influenza A/California/7/2009/H1N1-like virus vaccine | 3 |
| Stevens et al., 2020 | USA, North America | Safety of the Zoster Vaccine Recombinant Adjuvanted in Rheumatoid Arthritis and Other Systemic Rheumatic Disease Patients: A Single Center's Experience With 400 Patients. | ACR Open Rheumatology | Randomized clinical trial | RA and SRDs | AZA, MMF and CP | Zoster recombinant adjuvanted (ZRA) | 12 |
| Szabó et al., 2016 | Hungary, Europe | Immune response (immunogenicity and safety) in patients with systemic autoimmune diseases after pneumococcal conjugate vaccine | Clinical and Experimental Rheumatology | Randomized clinical trial | SLE and Pss | AZA and CP | Pneumococcal conjugate vaccine | 4 |
| Tarasova et al., 2016 | Russian, Asia | Efficacy and safety of 23-valent pneumococcal vaccine in SLE patients (preliminary results) | Annals of the Rheumatic Diseases | Randomized clinical trial | SLE | AZA and MMF | 23-valent pneumococcal polysaccharide vaccine (PPV23) | 43 |
| Tarasova et al., 2017 | Russian, Asia | Tolerability, efficacy and immunogenicity of 23-valent pneumococcal vaccine in SLE patients | Annals of the Rheumatic Diseases | Randomized clinical trial | SLE | Immunosuppressant without specification | 23-valent pneumococcal polysaccharide vaccine (PPV23) | 52 |
| Tarasova et al., 2018 | Russian, Asia | Safety and immunogenicity of 23-valent pneumococcal vaccine in SLE patients | Annals of the Rheumatic Diseases | Randomized clinical trial | SLE | Immunosuppressant without specification | 23-valent pneumococcal polysaccharide vaccine (PPV23) | 52 |
| Tarasova et al., 2019 | Russian, Asia | Use of 23-valent polysaccharide pneumococcal vaccine in patients with systemic lupus erythematosus: The relationship of immunogenicity with therapy | Annals of the Rheumatic Diseases | Randomized clinical trial | SLE | AZA, MMF and CP | 23-valent pneumococcal polysaccharide vaccine (PPV23) | 13 |
| Tarasova et al., 2020 | Russian, Asia | Immunogenicity of 23-valent polysaccharide pneumococcal vaccine in patients with systemic lupus erythematosus: Effect of biologic therapy on the vaccinal response | Annals of the Rheumatic Diseases | Randomized clinical trial | SLE | Immunosuppressant without specification | 23-valent pneumococcal polysaccharide vaccine (PPV23) | 52 |
| Tarasova et al., 2021 | Russian, Asia | Safety of 23-valent polysaccharide pneumococcal vaccine in patients with systemic lupus erythematosus | Annals of the Rheumatic Diseases | Randomized clinical trial | SLE | Immunosuppressant without specification | 23-valent pneumococcal polysaccharide vaccine (PPV23) | 13 |
| Tarasova et al., 2022 | Russian, Asia | Tolerability and safety of 23-valent polysaccharide pneumococcal vaccine in patients with systemic vasculitis, preliminary results | Annals Rheumatic Diseases | Randomized clinical trial | SV | AZA, MMF and CP | 23-valent pneumococcal polysaccharide vaccine (PPV23) | 26 |
| Tsuru et al., 2013 | Japan, Asia | Immune response to influenza vaccine and pneumococcal polysaccharide vaccine under IL-6 signal inhibition therapy with tocilizumab | Modern Rheumatology | Randomized clinical trial | RA | CYC | 23-valent pneumococcal polysaccharide vaccine (PPV23) | 12 |
| Wallin et al., 2009 | Brazil, South America | Safety and efficiency of influenza vaccination in systemic lupus erythematosus patients. | Acta Reumatologica Portuguesa | Randomized clinical trial | SLE | AZA and MMF | Influenza A/California/7/2009/H1N1-like virus vaccine | 6 |
| Wiesik-Szewczyk et al., 2010 | Poland, Europe | Anti-influenza vaccination in systemic lupus erythematosus patients: An analysis of specific humoral response and vaccination safety | Clinical Rheumatology | Randomized clinical trial | SLE | AZA and CP | Inactivated trivalent anti-influenza vaccine containing split virions | 12 |
| Williams et al., 1978 | USA, North America | Influenza immunization in systemic lupus erythematosus. A double-blind trial. | Annals of Internal Medicine | Randomized clinical trial | SLE | Immunosuppressant without specification | Bivalent (non disrupted) vaccine prepared from influenza | 20 |
| **Legend:** Diseases: Immune-mediated rheumatic disease (IMRD), Ankylosing spondylitis (AS), Antineutrophil cytoplasmic antibody associated vasculitis (ANCA- AAV), Antiphospholipid syndrome (APS), Autoimmune inflammatory rheumatic diseases (AIRD), Behçet disease (BD), Immune-mediated inflammatory diseases (IMIDs), Mixed connective tissue disease (MCTD), Other systemic rheumatic diseases (SRDs), Polimialgia reumática (PMR), Primary Sjögren’s Syndrome (pSS), Psoriatic arthritis (PsA), Rheumatoid arthritis (RA), Spondyloarthritis (SpA), Systemic lupus erythematosus (SLE), Systemic necrotising vasculitis (SNV), Systemic sclerosis (SSc), Systemic vasculitis (SV), Vasculitis (VAS). Medications: Azathioprine (AZA), mycophenolate mofetil (MMF), cyclosporine (CYC), cyclophosphamide (CP) | | | | | | | | |

**QUESTION 5. What is the effect of bDMARDs use (except RTX) on the response to inactivated vaccines in patients with immune-mediated rheumatic diseases (IMRD)?**

| **First author and year** | **Local** | **Study design** | **Immune-mediated rheumatic disease** | **bDMARDs name** | **Dosage bDMARDs use** | **Use time bDMARDs** | **Inactivated vaccine** | **Follow-up** |
| --- | --- | --- | --- | --- | --- | --- | --- | --- |
| Adler 2012 | Switzerland | Clinical trial | RA, SpA, Vasculitis, and CTD | TNFi, non-TNFi (Abatacept) | NI | NI | Influenza (H1N1) | 26 weeks |
| Alcocer 2015 | Spain | Cohort | RA, SpA, PsA, Pso, IBD, and Connectives | Infliximab, Adalimumab, Etanercept, Certolizumab, Golimumab, Tocilizumab, Abatacept, Rituximab, Ustekinumab or other biological therapy | NI | NI | Influenza | 6 weeks |
| Alten 2016 | Germany | Clinical trial | RA | Abatacept | 125mg | NI | Polyvalent pneumococcal polysaccharide (PPV23) and Influenza (Trivalent) | 4 weeks |
| Askling 2014 | Sweden | Clinical trial | RA | TNFi (Etanercept, Infliximab, and Adalimumab) | NI | NI | Hepatitis A | 52 weeks |
| Aydin 2020 | Turkey | Cohort | RA, AS, Pso, and Reactive arthritis | TNFi (Etanercept, Infliximab, Adalimumab, Certolizumab, and Golimimab), non-TNFi (Abatacept, Tocilizumab, and Ustekinumab) | NI | NI | Hepatitis B | 26 weeks |
| Azoicai 2018 | Romania | Cohort | SLE | Belimumab | 10mg/kg | NI | Polyvalent pneumococcal polysaccharide (PPV23) | 4 weeks |
| Bingham 2014 | USA | Clinical trial | RA | Tocilizumab | 8mg/kg | NI | Polyvalent pneumococcal polysaccharide (PPV23) and Tetanus | 8 weeks |
| Broyde 2016 | Israel | Cohort | RA, AS, PsA, IBD-associated SpA, and others | TNFi (Etanercept, Infliximab, and Adalimumab), non-TNFi (Tocilizumab) | NI | 43,4 mos (1-192) | Polyvalent pneumococcal polysaccharide (PPV23) | 520 weeks |
| Bühler 2019 | Switzerland | Cohort | RA, SpA, and Vasculitis | TNFi (Infliximab, Adalimumab, Golimumab, and Certolizumab) | NI | NI | Tetanus and Diphtheria | 12 weeks |
| Burmester 2016 | NI | Clinical trial | RA | Adalimumab | NI | NI | Influenza | 11 weeks |
| Calabrese 2020 | NI | Clinical trial | RA | Adalimumab | 40mg | NI | Live Herpes Zoster | 52 weeks |
| Caporuscio 2018 | Italy | Clinical trial | RA | TNFi | NI | NI | 13-valent pneumococcal conjugate (PCV13) | 52 weeks |
| Çaşkurlu, 2020 | Turkey | Cohort | RA, PsA, and AS | Adalimumab | 40mg | NI | 13-valent pneumococcal conjugate (PCV13) | 4 weeks |
| Caso 2016 | Italy | Case-control | PsA | TNFi | NI | NI | Influenza | 12 weeks |
| Chatham 2012 | USA | Clinical trial | SLE | Belimumab | 1 and 10mg/kg | NI | Influenza, Polyvalent pneumococcal polysaccharide (PPV12), and Tetanus | 52 weeks |
| Chatham 2017 | USA | Clinical trial | SLE | Belimumab | 10mg/kg | NI | Polyvalent pneumococcal polysaccharide (PPV23) | 4 weeks |
| Chen 2018 | Taiwan | Case-control | RA | TNFi (Etanercept and Adalimumab) | NI | NI | Influenza | NI |
| Colmegna 2020 | Canada | Clinical trial | RA | TNFi (Etanercept, Infliximab, Adalimumab, Certolizumab, and Golimumab), non-TNFi (Tocilizumab, Sarilumab, Abatacept), Rituximab | NI | NI | Influenza (Trivalent) | 104 weeks |
| Elkayam 2011 | Israel | Clinical trial | RA, SLE, PsA, and AS | TNFi (Etanercet, Infliximab, and Adalimumab) | NI | NI | Influenza (H1N1) | 4 weeks |
| Elkayam 2004 | Israel | Case-control | RA and AS | TNFi (Etanercet and Infliximab) | ETN: 25mg IFX: 3mg/kg | NI | Polyvalent pneumococcal polysaccharide (PPV23) | 4 weeks |
| Elkayam 2010 | Israel | Case-control | RA and AS | Infliximab | NI | NI | Influenza (Trivalent) | 6 weeks |
| Fernández-Prada 2023 | Spain | Cohort | RA, PsA, AS, IBD, SLE, and others | TNFi, non-TNFi | NI | NI | Hepatitis B | 4 weeks |
| Fomin 2006 | Israel | Case-control | RA | TNFi (Etanercept and Infliximab) | NI | NI | Influenza (Trivalent) | 6 weeks |
| França 2012 | São Paulo, Brazil | Case-control | RA and SpA | TNFi (Etanercept, Infliximab, and Adalimumab) | ETN: 50mg IFX: 3-5mg/kg ADA: 40mg | NI | Influenza (H1N1) | 3 weeks |
| Furer 2020 | Israel | Case-control | PsA | Secukinumab | 300mg (150-300) | 13 mos (13-70) | Influenza (Trivalent) | 6 weeks |
| Garrido 2022 | Netherlands | Cohort | IBD, RA, Pso, Psa, SpA, Neurological autoimmune disease, other autoinflammatory disease, and solid organ transplant recipient | NI | NI | NI | Pneumococcal (PCV13 and PPV23) | 52 weeks |
| Gabay 2011 | Switzerland | Cohort | RA, SpA, SLE, and ANCA associated necrotizing vasculitis | TNFi | NI | NI | Influenza (H1N1) | 8 weeks |
| Gelinck 2007 | Netherlands | Case-control | RA, Juvenile chronic arthritis6, PsA, SpA, IBD, and Still's disease | TNFi (Etanercept, Infliximab, and Adalimumab) | NI | NI | Influenza (Trivalent) | 4 weeks |
| Gelinck 2008 | Netherlands | Case-control | RA, AS, Pso, and CD | TNFi | NI | NI | Polyvalent pneumococcal polysaccharide (PPV23) | 4 weeks |
| Genovese 2013 | NI | Clinical trial | RA | Abatacept | 125mg | NI | Polyvalent pneumococcal polysaccharide (PPV23) and Influenza | 4 weeks |
| Hayashi 2012 | Japan | Cohort | RA | TNFi (Etanercept, Infliximab, and Adalimumab), non-TNFi (Tocilizumab and Abatacept) | NI | NI | Influenza (Trivalent) | 8 weeks |
| Intongkam 2018 | Thailand | Clinical trial | RA | TNFi (Etanercept and Infliximab) and Rituximab | NI | NI | Hepatitis B | 32 weeks |
| Ishikawa 2014 | Japan | Cohort | RA | TNFi (Golimumab), non-TNFi (Tocilizumab and Abatacept) | NI | NI | Influenza (Trivalent) | 8 weeks |
| Izumi 2017 | Japan | Clinical trial | RA | TNFi, non-TNFi (Toclizumab and Abatacept) | NI | NI | Polyvalent pneumococcal polysaccharide (PPV23) | 52 weeks |
| Kaine 2007 | USA | Clinical trial | RA | Adalimumab | 40mg | NI | Polyvalent pneumococcal polysaccharide (PPV23) and Influenza (Trivalent) | 4 weeks |
| Kapetanovic 2007 | Sweden | Case-control | RA | TNFi (Etanercept and Infliximab) | ETN: 25mg IFX: 3mg/kg | NI | Influenza (Trivalent) | 6 weeks |
| Kapetanovic 2006 | Sweden | Case-control | RA | TNFi (Etanercept and Infliximab) | ETN: 25mg IFX: 3mg/kg | NI | Polyvalent pneumococcal polysaccharide (PPV23) | 6 weeks |
| Kapetanovic 2011a | Sweden | Case-control | RA and SpA | TNFi | NI | NI | 7-valent pneumococcal conjugate | 6 weeks |
| Kapetanovic 2011b | Sweden | Case-control | RA | NI | NI | NI | Polyvalent pneumococcal polysaccharide (PPV23) | 6 weeks |
| Kapetanovic 2013 | Sweden | Cohort | RA and SpA | TNFi | NI | NI | 7-valent pneumococcal conjugate | 78 weeks |
| Kapetanovic 2014 | Sweden | Cohort | RA and SpA | TNFi, non-TNFi (Tocilizumab and Abatacept) | NI | NI | Influenza (H1N1) | 32 weeks |
| Kivitz 2014 | USA | Clinical trial | RA | Certolizumab | 400mg | NI | Polyvalent pneumococcal polysaccharide (PPV23) and Influenza (Trivalent) | 4 weeks |
| Kogure 2014 | Japan | Cohort | RA | NI | NI | NI | Influenza (Trivalent) | 4 weeks |
| Kostianovsky 2012 | France | Clinical trial | SNV, SSc, and SLE | NI | NI | NI | Influenza (Trivalent) | 3 weeks |
| Kubota 2007 | Japan | Case-control | RA | TNFi (Etanercept and Infliximab) | NI | NI | Influenza (Trivalent) | 6 weeks |
| Lakota 2019 | Slovenia | Case-control | RA, PsA, AS, Mixed CTD, Juvenile RA, and Still disease | TNFi (Etanercept, Infliximab, and Adalimumab), non-TNFi (Tocilizumab), Rituximab | NI | NI | Influenza (Trivalent) | 26 weeks |
| Migita 2015a | Japan | Clinical trial | RA | Abatacept | 547,6 ± 127,9 | NI | Polyvalent pneumococcal polysaccharide (PPV23) | 6 weeks |
| Migita 2015b | Japan | Clinical trial | RA | Golimumab | 52,08 ± 10,21 | NI | Polyvalent pneumococcal polysaccharide (PPV23) | 6 weeks |
| Mischlinger 2022 | Switzerland | Case-control | RA, SpA, PsA, Vasculitis | NI | NI | NI | Tetanus and Diphtheria | NI |
| Mori 2012 | Japan | Case-control | RA | Tocilizumab | 8mg/kg | NI | Influenza (Trivalent) | 6 weeks |
| Mori 2013 | Japan | Case-control | RA | Tocilizumab | 8mg/kg | NI | Polyvalent pneumococcal polysaccharide (PPV23) | 6 weeks |
| Mozaffarian 2013 | USA | Cohort | RA | Adalimumab | 40mg | NI | Influenza | 38 weeks |
| Muravyeva 2021 | NI | Cohort | SpA | Etanercept and Secukinumab | NI | NI | Polyvalent pneumococcal polysaccharide (PPV23) | 52 weeks |
| Nagel 2017 | Sweden | Case-control | SLE | Belimumab | NI | NI | 12-valent pneumococcal conjugate (PCV12) | 6 weeks |
| Nguyen 2017 | Denmark | Clinical trial | RA | TNFi, non-TNFi, RTX | NI | NI | Pneumococcal (PCV13 and PPV23) | 4 weeks |
| Nived 2017 | Sweden | Case-control | Systemic vasculitis | TNFi | NI | NI | 13-valent pneumococcal conjugate (PCV13) | 6 weeks |
| Nived 2020 | Sweden | Case-control | NI | Abatacept | NI | median: 3,7 (IQR: 0,7-10,2) | Pneumococcal (PCV13 and PPV23) | 6 weeks |
| Polachek 2015 | Israel | Case-control | PsA and Pso | TNFi (Etanercept, Infliximab, Adalimumab, and Golimumab) | NI | NI | Influenza (Trivalent) | 6 weeks |
| Rákóczi 2016 | Hungary | Case-control | RA | Etanercept | 50mg | NI | 13-valent pneumococcal conjugate (PCV13) | 8 weeks |
| Rasmussen 2023 | Denmark | Cohort | RA, SpA, and PsA | TNFi, non-TNFi | NI | NI | Polyvalent pneumococcal polysaccharide (PPV23) | 12 weeks |
| Ribeiro 2013 | São Paulo, Brazil | Case-control | RA | Abatacept | NI | NI | Influenza (H1N1) | 3 weeks |
| Richi 2019 | Spain | Case-control | PsA and AS | Secukinumab | NI | NI | Influenza (Trivalent) | 4 weeks |
| Richi 2021a | Spain | Cohort | RA, SpA, PsA, Undifferentiated arthritis, CTD, Pso, and IBD | TNFi (Etanercept, Infliximab, Adalimumab, Golimumab, and Certolizumab), non-TNFi (Tocilizumab and Abatacept), Rituximab, Anakinra | NI | NI | Pneumococcal (PCV13 and PPV23) | 4 weeks |
| Richi 2021b | Spain | Cohort | NI | TNFi (Etanercept, Infliximab, Adalimumab, Golimumab, and Certolizumab), Tocilizumab, Rituximab, Anakinra | NI | NI | Influenza (Trivalent) | 4 weeks |
| Romão 2020 | Portugal | Case-control | RA, PsA, AS, IBD-related SpA | TNFi, non-TNFi (Tocilizumab and Abatacept), Rituximab, Belimumab, Anakinra | NI | NI | Hepatitis B | 4 weeks |
| Rosdahl 2018 | Sweden | Clinical trial | RA | TNFi | NI | NI | Hepatitis A | 8 weeks |
| Sergeeva 2017 | Russia | Case-control | RA | TNFi | NI | NI | Polyvalent pneumococcal polysaccharide (PPV23) | 104 weeks |
| Solay 2019 | Turkey | Clinical trial | Pso, CD, UC, Hidradenitis supurativa, Behcet’s disease, and AS | TNFi (Etanercept, Infliximab, Adalimumab, and Golimumab), non-TNFi (Ustekinumab) | NI | NI | Hepatitis B | NI |
| Stapleton 2020 | USA | Clinical trial | RA | TNFi (Etanercept, Infliximab, and Adalimumab) | NI | NI | Influenza (Trivalent) | 26 weeks |
| Tarasova 2018 | Russia | Clinical trial | SLE | NI | NI | NI | Polyvalent pneumococcal polysaccharide (PPV23) | NI |
| Tarasova 2019 | Russia | Clinical trial | SLE | NI | NI | NI | Polyvalent pneumococcal polysaccharide (PPV23) | 52 weeks |
| Tarasova 2020 | Russia | Clinical trial | SLE | NI | NI | NI | Polyvalent pneumococcal polysaccharide (PPV23) | 52 weeks |
| Tarasova 2021 | Russia | Clinical trial | SLE | Belimumab and Rituximab | NI | NI | Polyvalent pneumococcal polysaccharide (PPV23) | 4 weeks |
| Tsuru 2013a | Japan | Clinical trial | RA | Tocilizumab | 8mg/kg | NI | Polyvalent pneumococcal polysaccharide (PPV23) and Influenza (Trivalent) | 12 weeks |
| Tsuru 2013b | Japan | Clinical trial | RA | TNFi (Infliximab and Etanercept), non-TNFi (Tocilizumab) | NI | NI | Influenza (H1N1) | 8 weeks |
| Venerito 2023 | Italy | Cohort | RA | Abatacept | NI | NI | Herpes Zoster | 12 weeks |
| Visvanathan 2007 | NI | Clinical trial | RA | Infliximab | 3 and 6mg/kg | NI | Polyvalent pneumococcal polysaccharide (PPV23) | 4 weeks |

**Table 2.** Studies presenting the mean antibody levels at any time during the follow-up period.

| **First author and year** | **Immune-mediated rheumatic disease** | **Vaccine** | **Follow-up (weeks)** | **Subunit** | **bDMARDs** | | | | **No bDMARDs** | | | | **Control** | | | |
| --- | --- | --- | --- | --- | --- | --- | --- | --- | --- | --- | --- | --- | --- | --- | --- | --- |
|  |  |  |  |  | **Mean baseline** | **SD baseline** | **Mean follow-up** | **SD follow-up** | **Mean baseline** | **SD baseline** | **Mean follow-up** | **SD follow-up** | **Mean baseline** | **SD baseline** | **Mean follow-up** | **SD follow-up** |
| Adler 2012 | Rheumatoid arthritis, Spondyloarthritis, Vasculitis, and Connective tissue disease | Influenza (H1N1) | 3 | H1N1 | 8,5 | NI | 47,7 | NI | NI | NI | NI | NI | 8,7 | NI | 116 | NI |
|  |  |  | 6 |  |  |  | 36,2 | NI | NI | NI | NI | NI | 8,7 | NI | 93 | NI |
|  |  |  | 26 |  |  |  | 19,6 | NI | NI | NI | NI | NI | 8,7 | NI | 51 | NI |
| Bühler 2019 | Rheumatic diseases (Rheumatoid arthritis, Spondyloarthritis, and Vasculitis) | Tetanus | 4 | Total | 1,37 | NI | 7,58 | NI | NI | NI | NI | NI | 1,16 | NI | 6,32 | NI |
|  |  | Diphtheria | NI | Total | 0,17 | NI | 0,65 | NI | NI | NI | NI | NI | 0,08 | NI | 0,3 | NI |
|  |  | Tetanus | 12 | Total | NI | NI | 4,59 | NI | NI | NI | NI | NI | NI | NI | 4,2 | NI |
|  |  | Diphtheria | NI | Total | NI | NI | 0,43 | NI | NI | NI | NI | NI | NI | NI | 0,23 | NI |
| Elkayam 2011 | Rheumatoid arthritis | Influenza (H1N1) | 4 | H1N1 | 5,72 | NI | 64,29 | NI | NI | NI | NI | NI | 4,3 | NI | 127 | NI |
|  | Psoriatic arthritis |  |  |  | 5,6 | NI | 55,5 | NI | NI | NI | NI | NI | NI | NI | NI | NI |
|  | Ankylosing spondylitis |  |  |  | 2,33 | NI | 57,04 | NI | NI | NI | NI | NI | NI | NI | NI | NI |
| Elkayam 2004 | Rheumatoid arthritis and Ankylosing spondylitis | Polyvalent pneumococcal polysaccharide (PPV23) | 4 | 14 | 5,18 | NI | 14,39 | NI | 1,89 | NI | 5,22 | NI | NI | NI | NI | NI |
|  |  |  |  | 23F | 2,45 | NI | 3,51 | NI | 1,98 | NI | 5,8 | NI | NI | NI | NI | NI |
|  |  |  |  | 4 | 2 | NI | 2,87 | NI | 2,02 | NI | 3,91 | NI | NI | NI | NI | NI |
|  |  |  |  | 8 | 3,66 | NI | 7,82 | NI | 2,2 | NI | 7,83 | NI | NI | NI | NI | NI |
|  |  |  |  | 9N | 2,76 | NI | 4,67 | NI | 2,24 | NI | 7,91 | NI | NI | NI | NI | NI |
|  |  |  |  | 7F | 2,56 | NI | 5 | NI | 1,81 | NI | 10,01 | NI | NI | NI | NI | NI |
|  |  |  |  | 2 | 2,87 | NI | 5,23 | NI | 3,47 | NI | 8,4 | NI | NI | NI | NI | NI |
| Elkayam 2010 | Rheumatoid arthritis | Influenza (Trivalent) | 6 | H1N1 | 3,1 | 3,6 | 4,7 | 1,3 | NI | NI | NI | NI | 2,72 | 2,1 | 4,1 | 1,8 |
|  |  |  |  | H3N2 | 2,8 | 2,2 | 5,3 | 1,7 | NI | NI | NI | NI | 2,5 | 2,3 | 4,3 | 2 |
|  |  |  |  | B/Malaysia | 2,5 | 2,5 | 4,2 | 2,3 | NI | NI | NI | NI | 2 | 2,2 | 3,7 | 1 |
|  | Ankylosing spondylitis |  |  | H1N1 | 1,75 | 2,1 | 4,3 | 2 | NI | NI | NI | NI | 3,4 | 1,8 | 4,8 | 1,3 |
|  |  |  |  | H3N2 | 1,87 | 2,2 | 4,4 | 1,7 | NI | NI | NI | NI | 2,8 | 1,8 | 5,2 | 1,2 |
|  |  |  |  | B/Malaysia | 0,53 | 1,24 | 2,6 | 2,2 | NI | NI | NI | NI | 0,7 | 1,5 | 2,7 | 2 |
| Fomin 2006 | Rheumatoid arthritis | Influenza (Trivalent) | 6 | B/Hong Kong | 4 | 1,4 | 5,3 | 1,2 | NI | NI | NI | NI | 3,83 | 1,5 | 6,1 | 0,9 |
|  |  |  |  | A/Panama | 4,03 | 1,4 | 5,33 | 1,5 | NI | NI | NI | NI | 4,4 | 1,4 | 5,8 | 1,2 |
|  |  |  |  | A/New Caledonia | 3,4 | 1,4 | 4,7 | 1,4 | NI | NI | NI | NI | 2,9 | 1 | 5,1 | 1,3 |
| Gabay 2011 | Rheumatoid arthritis, Spondyloarthritis, Systemic lupus erythematosus, and ANCA associated necrotizing vasculitis | Influenza (H1N1) | 4 | H1N1 | 13 | NI | 146 | NI | NI | NI | NI | NI | 9 | NI | 340 | NI |
|  | Rheumatoid arthritis, Spondyloarthritis, Systemic lupus erythematosus, and ANCA associated necrotizing vasculitis |  | 8 |  | 13 | NI | 287 | NI | NI | NI | NI | NI | NI | NI | NI | NI |
| Ishikawa 2014 | Rheumatoid arthritis | Polyvalent pneumococcal polysaccharide (PPV23) | NI | 9V | 2,6 | NI | 6,1 | NI | 2,65 | NI | 6,23 | NI | NI | NI | NI | NI |
|  |  |  |  | 14 | 2,22 | NI | 5,09 | NI | 1,45 | NI | 4,4 | NI | NI | NI | NI | NI |
|  |  |  |  | 18C | 2,9 | NI | 7,05 | NI | 2,84 | NI | 7,49 | NI | NI | NI | NI | NI |
|  |  |  |  | 19F | 1,96 | NI | 3,96 | NI | 2,16 | NI | 4,16 | NI | NI | NI | NI | NI |
|  |  |  |  | 23F | 1,84 | NI | 4,01 | NI | 2,26 | NI | 4,22 | NI | NI | NI | NI | NI |
|  |  | Influenza (Trivalent) |  | H1N1 | 47,16 | NI | 162,64 | NI | 51,89 | NI | 188,68 | NI | NI | NI | NI | NI |
|  |  |  |  | H3N2 | 105,86 | NI | 472,25 | NI | 89,13 | NI | 602,81 | NI | NI | NI | NI | NI |
|  |  |  |  | B (Hong Kong) | 22,62 | NI | 73,86 | NI | 22,71 | NI | 92,94 | NI | NI | NI | NI | NI |
| Kapetanovic 2007 | Rheumatoid arthritis | Influenza (Trivalent) | 6 | H1N1 | 7,59 | NI | 49,45 | NI | 9,22 | NI | 123,3 | NI | 12,28 | NI | 84,66 | NI |
|  |  |  |  | H3N2 | 14,14 | NI | 91,06 | NI | 10,01 | NI | 80,96 | NI | 22,5 | NI | 68,99 | NI |
|  |  |  |  | B | 18,91 | NI | 170,65 | NI | 22,77 | NI | 279,15 | NI | 39 | NI | 109,9 | NI |
| Kapetanovic 2006 | Rheumatoid arthritis | Polyvalent pneumococcal polysaccharide (PPV23) | 6 | 6B | 0,9 | NI | 3,8 | NI | 1,1 | NI | 2,1 | NI | 2,1 | NI | 4,6 | NI |
|  |  |  |  | 23F | 0,7 | NI | 2,4 | NI | 0,8 | NI | 1,6 | NI | 1,2 | NI | 2,8 | NI |
| Kapetanovic 2011b | Rheumatoid arthritis | 7-valent pneumococcal conjugate | 6 | 6B | 1,4 | NI | 3,6 | NI | 2 | NI | 3,5 | NI | NI | NI | NI | NI |
|  |  |  |  | 23F | 0,6 | NI | 1,9 | NI | 0,7 | NI | 1,9 | NI | NI | NI | NI | NI |
|  | Spondylarthropathy (Psoriatic arthritis and Ankylosing spondylitis) |  |  | 6B | 1,5 | NI | 4,8 | NI | 2,9 | NI | 9,5 | NI | NI | NI | NI | NI |
|  |  |  |  | 23F | 0,7 | NI | 3,1 | NI | 0,97 | NI | 6,4 | NI | NI | NI | NI | NI |
| Kapetanovic 2014 | Rheumatoid arthritis | Influenza (H1N1) | 32 | H1N1 | 9,6 | NI | 48 | NI | 8,6 | NI | 37,8 | NI | NI | NI | NI | NI |
|  |  |  |  | Total | 17,4 | NI | 40 | NI | NI | NI | NI | NI | NI | NI | NI | NI |
|  |  |  |  | Total | 28,3 | NI | 113,1 | NI | NI | NI | NI | NI | NI | NI | NI | NI |
|  | Spondyloarthropathy |  |  | Total | 9,2 | NI | 76 | NI | 9,3 | NI | 52,4 | NI | NI | NI | NI | NI |
| Kivitz 2014 | Rheumatoid arthritis | Polyvalent pneumococcal polysaccharide (PPV23) | 4 | 6B | 0,5 | NI | 1,2 | NI | NI | NI | NI | NI | 0,5 | NI | 1,5 | NI |
|  |  |  |  | 9V | 0,3 | NI | 1,2 | NI | NI | NI | NI | NI | 0,5 | NI | 1,5 | NI |
|  |  |  |  | 14 | 1,4 | NI | 4,6 | NI | NI | NI | NI | NI | 1,4 | NI | 6,1 | NI |
|  |  |  |  | 18C | 0,8 | NI | 3,3 | NI | NI | NI | NI | NI | 0,8 | NI | 2,9 | NI |
|  |  |  |  | 19F | 0,7 | NI | 1,9 | NI | NI | NI | NI | NI | 0,8 | NI | 2,1 | NI |
|  |  |  |  | 23F | 0,5 | NI | 1,3 | NI | NI | NI | NI | NI | 0,5 | NI | 1,5 | NI |
|  |  | Influenza (Trivalent) |  | H1N1 | 16,6 | NI | 65,7 | NI | NI | NI | NI | NI | 16,7 | NI | 89,7 | NI |
|  |  |  |  | H3N2 | 16,4 | NI | 105 | NI | NI | NI | NI | NI | 19,4 | NI | 193,2 | NI |
|  |  |  |  | B | 11 | NI | 43,1 | NI | NI | NI | NI | NI | 11 | NI | 46,1 | NI |
| Kogure 2014 | Rheumatoid arthritis | Influenza (Trivalent) | 4 | H1N1 | 33,5 | 64,1 | 99,7 | 236,3 | NI | NI | NI | NI | NI | NI | NI | NI |
|  |  |  |  | H3N2 | 62,7 | 176,4 | 220,4 | 315,9 | NI | NI | NI | NI | NI | NI | NI | NI |
|  |  |  |  | B | 14,2 | 10,5 | 36,4 | 87,3 | NI | NI | NI | NI | NI | NI | NI | NI |
| Kostianovsky 2012 | Autoimmune diseases (Systemic necrotising vasculitides, Systemic sclerosis, and Systemic lupus erythematosus) | Influenza (Trivalent) | 3 | Total | 22,1 | NI | 88,7 | NI | NI | NI | NI | NI | 13,4 | 40,7 | NI | NI |
| Lakota 2019 | Autoimmune inflammatory diseases (Rheumatoid arthritis, Psoriatic arthritis, Ankylosing spondylitis, Mixed connective tissue disease, Juvenile rheumatoid arthritis, and Still disease) | Influenza (Trivalent) | 26 | H1N1 | 45,8 | NI | 182,6 | NI | NI | NI | NI | NI | 64,2 | NI | 133,9 | NI |
|  |  |  |  | H3N2 | 131,3 | NI | 312 | NI | NI | NI | NI | NI | 709,6 | NI | 1065 | NI |
|  |  |  |  | B | 116,1 | NI | 262,6 | NI | NI | NI | NI | NI | 131,2 | NI | 253,8 | NI |
| Migita 2015a | Rheumatoid arthritis | Polyvalent pneumococcal polysaccharide (PPV23) | 6 | 6B | 1,12 | NI | 2,29 | NI | 1,42 | NI | 4,36 | NI | 0,84 | NI | 4,05 | NI |
|  |  |  |  | 23F | 1,22 | NI | 4,61 | NI | 1,79 | NI | 7,41 | NI | 1,17 | NI | 11,61 | NI |
| Migita 2015b | Rheumatoid arthritis | Polyvalent pneumococcal polysaccharide (PPV23) | 6 | 6B | 1,35 | NI | 1,28 | NI | 1,42 | NI | 4,36 | NI | 0,84 | NI | 4,05 | NI |
|  |  |  |  | 23F | 2,13 | NI | 3,04 | NI | 1,79 | NI | 7,41 | NI | 1,17 | NI | 11,61 | NI |
|  |  |  |  | Total | NI | NI | NI | NI | NI | NI | NI | NI | NI | NI | NI | NI |
| Mischlinger 2022 | Rheumatoid arthritis | Tetanus | Short-term | Total | NI | NI | 0,82 | NI | NI | NI | NI | NI | NI | NI | 1,66 | NI |
|  | Spondyloarthritis |  |  |  | NI | NI | 1,76 | NI | NI | NI | NI | NI | NI | NI | NI | NI |
|  | Vasculitis |  |  |  | NI | NI | 1,24 | NI | NI | NI | NI | NI | NI | NI | NI | NI |
|  | Psoriatic arthritis |  |  |  | NI | NI | 2,06 | NI | NI | NI | NI | NI | NI | NI | NI | NI |
| Mori 2012 | Rheumatoid arthritis | Influenza (Trivalent) | 6 | H1N1 | 62 | NI | 211,7 | NI | 31,7 | NI | 120,5 | NI | 15,3 | NI | 169,4 | NI |
|  |  |  |  | H3N2 | 55,2 | NI | 237,8 | NI | 37,9 | NI | 120,2 | NI | 36,9 | NI | 93,9 | NI |
|  |  |  |  | B | 72,1 | NI | 161,8 | NI | 45,5 | NI | 103,1 | NI | 23,9 | NI | 68,9 | NI |
|  |  |  |  | 6B | 1,3 | NI | 6,1 | NI | 1,2 | NI | 2,2 | NI | 1,1 | NI | 2,5 | NI |
|  |  |  |  | 23F | 1,3 | NI | 4,6 | NI | 1 | NI | 2,4 | NI | 1 | NI | 3,6 | NI |
| Nagel 2017 | Systemic lupus erythematosus | 12-valent pneumococcal conjugate (PCV12) | 6 | 1 | 1,5 | NI | 13,6 | NI | 2,3 | NI | 7,1 | NI | 1,9 | NI | 32,3 | NI |
|  |  |  |  | 3 | 0,1 | NI | 0,5 | NI | 0,2 | NI | 0,9 | NI | 0,2 | NI | 0,6 | NI |
|  |  |  |  | 4 | 0,1 | NI | 0,8 | NI | 0,2 | NI | 0,6 | NI | 0,2 | NI | 1,4 | NI |
|  |  |  |  | 5 | 0,2 | NI | 0,5 | NI | 0,3 | NI | 0,3 | NI | 0,2 | NI | 0,6 | NI |
|  |  |  |  | 6B | 0,1 | NI | 1,3 | NI | 0,4 | NI | 2 | NI | 0,3 | NI | 2,2 | NI |
|  |  |  |  | 7F | 0,3 | NI | 2,4 | NI | 0,4 | NI | 2,7 | NI | 0,4 | NI | 2,6 | NI |
|  |  |  |  | 9V | 0,5 | NI | 1 | NI | 1,4 | NI | 5,1 | NI | 0,7 | NI | 4,8 | NI |
|  |  |  |  | 14A | 1,8 | NI | 8,6 | NI | 2,5 | NI | 11 | NI | 1,3 | NI | 9,2 | NI |
|  |  |  |  | 18C | 0,4 | NI | 1,9 | NI | 0,9 | NI | 4,8 | NI | 0,8 | NI | 5,1 | NI |
|  |  |  |  | 19A | 2,6 | NI | 7,4 | NI | 8,8 | NI | 27 | NI | 3,4 | NI | 18,6 | NI |
|  |  |  |  | 19F | 2,7 | NI | 8,3 | NI | 6,3 | NI | 17,5 | NI | 2 | NI | 11,5 | NI |
|  |  |  |  | 23F | 0,5 | NI | 3,6 | NI | 1,4 | NI | 8,8 | NI | 1,1 | NI | 10,5 | NI |
|  |  |  |  | 6B | 0,5 | NI | 1,7 | NI | NI | NI | NI | NI | 0,8 | NI | 3,1 | NI |
|  |  |  |  | 23F | 0,3 | NI | 1,8 | NI | NI | NI | NI | NI | 0,6 | NI | 3,3 | NI |
| Nived 2020 | Inflammatory rheumatic disease | 13-valent pneumococcal conjugate (PCV13) | 6 | 1 | 0,11 | NI | 0,37 | NI | 0,1 | NI | 0,27 | NI | 0,12 | NI | 1,09 | NI |
|  |  |  |  | 3 | 0,1 | NI | 0,35 | NI | 0,06 | NI | 0,14 | NI | 0,08 | NI | 0,68 | NI |
|  |  |  |  | 4 | 0,13 | NI | 0,28 | NI | 0,11 | NI | 0,36 | NI | 0,18 | NI | 2,15 | NI |
|  |  |  |  | 5 | 0,17 | NI | 0,48 | NI | 0,11 | NI | 0,54 | NI | 0,09 | NI | 0,61 | NI |
|  |  |  |  | 6B | 0,2 | NI | 0,77 | NI | 0,17 | NI | 0,62 | NI | 0,16 | NI | 1,32 | NI |
|  |  |  |  | 7F | 0,31 | NI | 1,8 | NI | 0,51 | NI | 2,57 | NI | 0,23 | NI | 3,48 | NI |
|  |  |  |  | 9V | 0,22 | NI | 0,93 | NI | 0,12 | NI | 0,58 | NI | 0,15 | NI | 1,6 | NI |
|  |  |  |  | 14 | 1,6 | NI | 5,89 | NI | 0,54 | NI | 2,89 | NI | 1,1 | NI | 6,77 | NI |
|  |  |  |  | 18C | 0,76 | NI | 3,9 | NI | 0,41 | NI | 2,83 | NI | 1,03 | NI | 5,74 | NI |
|  |  |  |  | 19A | 0,31 | NI | 1,01 | NI | 0,2 | NI | 0,86 | NI | 0,2 | NI | 1,4 | NI |
|  |  |  |  | 19F | 0,76 | NI | 1,25 | NI | 0,85 | NI | 1,89 | NI | 0,78 | NI | 3,09 | NI |
|  |  |  |  | 23F | 0,7 | NI | 3,1 | NI | 0,34 | NI | 1,17 | NI | 0,41 | NI | 6,12 | NI |
|  |  | Polyvalent pneumococcal polysaccharide (PPV23) |  | 1 | NI | NI | 0,4 | NI | NI | NI | 0,84 | NI | NI | NI | 3,21 | NI |
|  |  |  |  | 3 | NI | NI | 0,38 | NI | NI | NI | 0,21 | NI | NI | NI | 1,9 | NI |
|  |  |  |  | 4 | NI | NI | 0,29 | NI | NI | NI | 0,77 | NI | NI | NI | 4,07 | NI |
|  |  |  |  | 5 | NI | NI | 0,53 | NI | NI | NI | 0,89 | NI | NI | NI | 1,52 | NI |
|  |  |  |  | 6B | NI | NI | 0,76 | NI | NI | NI | 0,67 | NI | NI | NI | 2,41 | NI |
|  |  |  |  | 7F | NI | NI | 1,89 | NI | NI | NI | 2,42 | NI | NI | NI | 4,04 | NI |
|  |  |  |  | 9V | NI | NI | 0,92 | NI | NI | NI | 0,58 | NI | NI | NI | 1,94 | NI |
|  |  |  |  | 14 | NI | NI | 6,44 | NI | NI | NI | 3,28 | NI | NI | NI | 13,07 | NI |
|  |  |  |  | 18C | NI | NI | 4,21 | NI | NI | NI | 2,37 | NI | NI | NI | 6,33 | NI |
|  |  |  |  | 19A | NI | NI | 1,05 | NI | NI | NI | 0,71 | NI | NI | NI | 2,19 | NI |
|  |  |  |  | 19F | NI | NI | 1,53 | NI | NI | NI | 4,38 | NI | NI | NI | 12,52 | NI |
|  |  |  |  | 23F | NI | NI | 2,83 | NI | NI | NI | 1,46 | NI | NI | NI | 6,3 | NI |
| Rákóczi 2016 | Rheumatoid arthritis | 13-valent pneumococcal conjugate (PCV13) | 4 | Total | 68,2 | 247,7 | 155,2 | NI | NI | NI | NI | NI | 124 | 99 | 417,7 | 198,3 |
|  |  |  | 8 |  |  | 207,6 | 127,6 | NI | NI | NI | NI | NI | NI | NI | 356,4 | 171,7 |
| Rasmussen 2023 | Rheumatoid arthritis, Spondyloarthritis, and Psoriatic arthritis | Polyvalent pneumococcal polysaccharide (PPV23) | 12 | Total | NI | NI | NI | NI | NI | NI | NI | NI | NI | NI | NI | NI |
| Ribeiro 2013 | Rheumatoid arthritis | Influenza (H1N1) | 3 | Total | 6 | NI | 10,7 | NI | 6 | NI | 52,6 | NI | 6,6 | NI | 76,1 | NI |
| Richi 2019 | Psoriatic arthritis and Ankylosing spondylitis | Influenza (Trivalent) | 4 | H1N1 | 60 | NI | 276 | NI | NI | NI | NI | NI | 107 | NI | 428 | NI |
|  |  |  |  | H3N2 | 65 | NI | 91 | NI | NI | NI | NI | NI | 85 | NI | 86 | NI |
|  |  |  |  | B | 20 | NI | 74 | NI | NI | NI | NI | NI | 32 | NI | 171 | NI |
| Solay 2019 | Autoimmune diseases (Psoriasis, Crohn’s disease, Rheumatoid arthritis, Ulcerative colitis, Hidradenitis supurativa, Behcet’s disease, Ankylosing spondylitis) | Hepatitis B | NI | Total | NI | NI | 245 | NI | NI | NI | NI | NI | NI | NI | NI | NI |

**Table 3.** Studies presenting serological responses at any time during follow-up period.

| **First author and year** | **Immune-mediated rheumatic disease** | **bDMARDs name** | **Vaccine** | **Follow-up (weeks)** | **Subunit** | **Type of serological response** | **bDMARDs serological response** | **No bDMARDs serological response** | **Control serological response** |
| --- | --- | --- | --- | --- | --- | --- | --- | --- | --- |
| Adler 2012 | Autoimmune diseases | TNFi, non-TNFi (Abatacept) | Influenza (H1N1) | 3 | H1N1 | Seroconversion | 95 (64,0%) | NI | 34 (85,0%) |
|  |  |  |  | 6 |  |  | 80 (54,0%) | NI | 32 (80,0%) |
|  |  |  |  | 26 |  |  | 49 (33,0%) | NI | 26 (65,0%) |
| Alcocer 2015 | RA, SpA, PsA, Pso, IBD, and Connectives | Infliximab, Adalimumab, Etanercept, Certolizumab, Golimumab, Tocilizumab, Abatacept, Rituximab, Ustekinumab or other biological therapy | Influenza | 6 | A | Seroconversion | 6 (6,2%) | NI | NI |
|  |  |  |  |  | B |  | 4 (4,2%) | NI | NI |
| Alten 2016 | RA | Abatacept | Polyvalent pneumococcal polysaccharide (PPV23) | 4 | Total | Seroprotection | 62 (55,4%) | NI | NI |
|  |  |  | Influenza (Trivalent) |  |  |  | 91 (49,5%) | NI | NI |
| Askling 2014 | RA | TNFi (Etanercept, Infliximab, and Adalimumab) | Hepatitis A | 4 | Total | Seroprotection | 11 (72,0%) | 1 (6,0%) | NI |
|  |  |  |  | 26 |  |  | 9 (60,0%) | 7 (41,0%) | NI |
|  |  |  |  | 30 |  |  | 15 (100,0%) | 14 (82,0%) | NI |
|  |  |  |  | 52 |  |  | 14 (93,0%) | 14 (82,0%) | NI |
| Aydin 2020 | RA, AS, Pso, and Reactive arthritis | TNFi (Etanercept, Infliximab, Adalimumab, Certolizumab, and Golimimab), non-TNFi (Abatacept, Tocilizumab, and Ustekinumab) | Hepatitis B | 26 | Total | Immunogenicity | 72 (85,7%) | NI | NI |
| Azoicai 2018 | SLE | Belimumab | Polyvalent pneumococcal polysaccharide (PPV23) | 4 | Total | Seroprotection | 44 (97,0%) | NI | 33 (97,0%) |
| Bingham 2014 | RA | Tocilizumab | Polyvalent pneumococcal polysaccharide (PPV23) | NI | Total | Immunogenicity | 30 (60,0%) | 17 (70,8%) | NI |
|  |  |  | Tetanus |  |  |  | 21 (42,0%) | 9 (39,1%) | NI |
| Broyde 2016 | Autoimmune diseases | TNFi (Etanercept, Infliximab, and Adalimumab), non-TNFi (Tocilizumab) | Polyvalent pneumococcal polysaccharide (PPV23) | 520 | Total | Seroprotection | 79 (89,8%) | NI | 29 (69,0%) |
| Bühler 2019 | Autoimmune diseases | TNFi (Infliximab, Adalimumab, Golimumab, and Certolizumab) | Tetanus | 4 | Total | Seroprotection | 77 (100,0%) | NI | 27 (96,0%) |
|  |  |  | Diphtheria |  |  |  | 70 (91,0%) | NI | 21 (75,0%) |
|  |  |  | Tetanus | 12 |  |  | 75 (100,0%) | NI | 27 (96,0%) |
|  |  |  | Diphtheria |  |  |  | 64 (85,0%) | NI | 19 (68,0%) |
| Caporuscio 2018 | RA | TNFi | 13-valent pneumococcal conjugate (PCV13) | 4 | Total | Seroconversion | 13 (92,9%) | NI | NI |
|  |  |  |  |  | 1 |  | 10 (71,4%) | NI | NI |
|  |  |  |  |  | 3 |  | 8 (57,1%) | NI | NI |
|  |  |  |  |  | 4 |  | 10 (71,4%) | NI | NI |
|  |  |  |  |  | 5 |  | 10 (71,4%) | NI | NI |
|  |  |  |  |  | 6A |  | 6 (42,9%) | NI | NI |
|  |  |  |  |  | 6B |  | 8 (57,1%) | NI | NI |
|  |  |  |  |  | 7F |  | 6 (42,9%) | NI | NI |
|  |  |  |  |  | 9V |  | 12 (85,7%) | NI | NI |
|  |  |  |  |  | 14 |  | 8 (57,1%) | NI | NI |
|  |  |  |  |  | 18C |  | 8 (57,1%) | NI | NI |
|  |  |  |  |  | 19A |  | 9 (64,3%) | NI | NI |
|  |  |  |  |  | 19F |  | 11 (78,6%) | NI | NI |
|  |  |  |  |  | 23F |  | 8 (57,1%) | NI | NI |
| Çaşkurlu 2020 | RA, PsA, and AS | Adalimumab | 13-valent pneumococcal conjugate (PCV13) | 4 | Total | Seroprotection | 36 (100,0%) | NI | NI |
| Chatham 2017 | SLE | Belimumab | Polyvalent pneumococcal polysaccharide (PPV23) | 4 | Total | Immunogenicity | 40 (97,6%) | NI | 32 (97,0%) |
| Colmegna 2019 | RA | TNFi (Etanercept, Infliximab, Adalimumab, Certolizumab, and Golimumab), non-TNFi (Tocilizumab, Sarilumab, Abatacept), Rituximab | Influenza (Trivalent) | 52 | H3N2 | Seroconversion | 31 (22,0%) | NI | 12 (9,0%) |
|  |  |  |  |  | B |  | 62 (45,0%) | NI | 40 (29,0%) |
|  |  |  |  |  | H1N1 |  | 36 (52,0%) | NI | 18 (25,0%) |
|  |  |  |  | 104 | H3N2 |  | 31 (22,0%) | NI | 12 (9,0%) |
|  |  |  |  |  | B |  | 62 (45,0%) | NI | 40 (29,0%) |
|  |  |  |  |  | H1N1 |  | 32 (46,0%) | NI | 17 (26,0%) |
| Elkayam 2011 | RA | TNFi (Etanercet, Infliximab, and Adalimumab) | Influenza (H1N1) | 4 | H1N1 | Immunogenicity | 23 (56,0%) | NI | 21 (84,0%) |
|  | PsA |  |  |  |  |  | 10 (59,0%) | NI | NI |
|  | AS |  |  |  |  |  | 8 (53,0%) | NI | NI |
|  | RA |  |  |  |  | Seroprotection | 29 (71,0%) | NI | 23 (92,0%) |
|  | PsA |  |  |  |  |  | 13 (76,0%) | NI | NI |
|  | AS |  |  |  |  |  | 9 (60,0%) | NI | NI |
| Elkayam 2004 | RA and AS | TNFi (Etanercet and Infliximab) | Polyvalent pneumococcal polysaccharide (PPV23) | 4 | 14 | Immunogenicity | 7 (44,0%) | 9 (53,0%) | NI |
|  |  |  |  |  | 23F |  | 2 (13,0%) | 9 (53,0%) | NI |
|  |  |  |  |  | 4 |  | 3 (19,0%) | 6 (35,0%) | NI |
|  |  |  |  |  | 8 |  | 8 (50,0%) | 12 (71,0%) | NI |
|  |  |  |  |  | 9N |  | 4 (25,0%) | 10 (59,0%) | NI |
|  |  |  |  |  | 7F |  | 7 (44,0%) | 12 (71,0%) | NI |
|  |  |  |  |  | 2 |  | 6 (38,0%) | 10 (59,0%) | NI |
| Fernández-Prada 2022 | Autoimmune diseases | TNFi, non-TNFi | Hepatitis B | 4 | Total | Seroconversion | 247 (82,1%) | NI | NI |
|  |  |  |  | 26 |  |  | 268 (89,0%) | NI | NI |
| Fomin 2006 | RA | TNFi (Etanercept and Infliximab) | Influenza (Trivalent) | 6 | B/Hong Kong | Immunogenicity | 51 (67,0%) | NI | 26 (87,0%) |
|  |  |  |  |  | A/Panama |  | 43 (53,0%) | NI | 16 (54,0%) |
|  |  |  |  |  | A/New Caledonia |  | 43 (53,0%) | NI | 20 (68,0%,) |
|  |  | Infliximab |  |  | B/Hong Kong |  | 17 (77,0%) | 39 (65,0%) | NI |
|  |  |  |  |  | A/Panama |  | 12 (55,0%) | 25 (42,0%) | NI |
|  |  |  |  |  | A/New Caledonia |  | 12 (56,0%) | 26 (44,0%) | NI |
| Furer 2019 | PsA | Secukinumab | Influenza (Trivalent) | 6 | H1N1 | Immunogenicity | 15 (51,0%) | NI | 4 (26,7%) |
|  |  |  |  |  | H3N2 |  | 23 (79,0%) | NI | 9 (60,0%) |
|  |  |  |  |  | B |  | 9 (30,0%) | NI | 0 (0%) |
|  |  |  |  |  | H1N1 | Seroprotection | 25 (81,0%) | NI | 14 (93,3%) |
|  |  |  |  |  | H3N2 |  | 28 (90,0%) | NI | 15 (100,0%) |
|  |  |  |  |  | B |  | 31 (100,0%) | NI | 15 (100,0%) |
| Garrido 2022 | Autoimmune diseases | NI | Pneumococcal (PCV13 and PPV23) | 8 | PPV13 | Seroprotection | 26 (53,0%) | 25 (57,0%) | 24 (75,0%) |
|  |  |  |  |  | PPV23 |  | 1 (2,0%) | 0 (0%) | 3 (9,4%) |
|  |  |  |  |  | Total |  | 6 (12,0%) | 5 (11,0%) | 10 (31,0%) |
|  |  |  |  | 16 | PPV13 |  | 24 (52,0%) | 24 (53,0%) | 29 (85,0%) |
|  |  |  |  |  | PPV23 |  | 31 (67,0%) | 29 (64,0%) | 28 (82,0%) |
|  |  |  |  |  | Total |  | 26 (57,0%) | 26 (58,0%) | 28 (82,0%) |
|  |  |  |  | 26 | PPV13 |  | 19 (49,0%) | 21 (54,0%) | 24 (75,0%) |
|  |  |  |  |  | PPV23 |  | 24 (62,0%) | 21 (54,0%) | 27 (84,0%) |
|  |  |  |  |  | Total |  | 19 (49,0%) | 21 (54,0%) | 24 (75,0%) |
|  |  |  |  | 52 | PPV13 |  | 16 (35,0%) | 18 (41,0%) | 20 (57,0%) |
|  |  |  |  |  | PPV23 |  | 22 (48,0%) | 23 (52,0%) | 26 (74,0%) |
|  |  |  |  |  | Total |  | 18 (39,0%) | 21 (48,0%) | 22 (63,0%) |
| Gelinck 2007 | Autoimmune diseases | TNFi (Etanercept, Infliximab, and Adalimumab) | Influenza (Trivalent) | 4 | H1N1 | Seroprotection | 52 (81,0%) | 40 (85,0%) | 16 (89,0%) |
|  |  |  |  |  | H3N2 |  | 52 (81,0%) | 41 (86,0%) | 17 (94,0%) |
|  |  |  |  |  | B |  | 51 (80,0%) | 42 (88,0%) | 16 (89,0%) |
| Gelinck 2008 | RA, AS, Pso, and CD | TNFi | Polyvalent pneumococcal polysaccharide (PPV23) | 4 | 6B | Immunogenicity | 3 (6,0%) | 4 (10,0%) | 1 (6,0%) |
|  |  |  |  |  |  | Seroprotection | 8 (15,0%) | 10 (24,0%) | 3 (17,0%) |
|  |  |  |  |  | 9V | Immunogenicity | 23 (44,0%) | 24 (59,0%) | 12 (67,0%) |
|  |  |  |  |  |  | Seroprotection | 52 (100,0%) | 41 (100,0%) | 17 (94,0%) |
|  |  |  |  |  | 19F | Immunogenicity | 7 (14,0%) | 14 (34,0%) | 8 (44,0%) |
|  |  |  |  |  |  | Seroprotection | 49 (94,0%) | 38 (93,0%) | 15 (83,0%) |
|  |  |  |  |  | 23F | Immunogenicity | 18 (35,0%) | 18 (44,0%) | 7 (39,0%) |
|  |  |  |  |  |  | Seroprotection | 43 (83,0%) | 34 (83,0%) | 15 (83,0%) |
| Genovese 2013 | RA | Abatacept | Polyvalent pneumococcal polysaccharide (PPV23) | 4 | Total | Seroprotection | 94 (83,9%) | NI | NI |
|  |  |  | Influenza |  |  |  | 151 (82,1%) | NI | NI |
| Hayashi 2012 | RA | Infliximab | Influenza (Trivalent) | 8 | Total | Seroconversion | 6 (46,2%) | 8 (54,3%) | NI |
|  |  |  |  |  |  | Seroprotection | 7 (61,1%) | 10 (64,4%) | NI |
|  |  | Etanercept |  |  |  | Seroconversion | 8 (73,3%) | NI | NI |
|  |  |  |  |  |  | Seroprotection | 10 (87,9%) | NI | NI |
|  |  | Adalimumab |  |  |  | Seroconversion | 2 (62,5%) | NI | NI |
|  |  |  |  |  |  | Seroprotection | 2 (66,7%) | NI | NI |
|  |  | Tocilizumab |  |  |  | Seroconversion | 6 (48,3%) | NI | NI |
|  |  |  |  |  |  | Seroprotection | 7 (58,3%) | NI | NI |
|  |  | Abatacept |  |  |  | Seroconversion | 2 (21,4%) | NI | NI |
|  |  |  |  |  |  | Seroprotection | 4 (33,3%) | NI | NI |
| Intongkam 2018 | RA | TNFi (Etanercept and Infliximab) and Rituximab | Hepatitis B | 32 | Total | Immunogenicity | 6 (50,0%) | 23 (69,7%) | 9 (100,0%) |
|  |  | Etanercept or Infliximab |  |  |  |  | 4 (80,0%) | NI | NI |
| Ishikawa 2014 | RA | Abatacept | Influenza (Trivalent) | 4 | H1N1 | Seroprotection | 11 (29,7%) | NI | NI |
|  |  |  |  |  | H3N2 |  | 22 (59,5%) | NI | NI |
|  |  |  |  |  | B |  | 10 (27,0%) | NI | NI |
|  |  | Tocilizumab |  |  | H1N1 |  | 42 (52,5%) | NI | NI |
|  |  |  |  |  | H3N2 |  | 69 (86,3%) | NI | NI |
|  |  |  |  |  | B |  | 40 (50,0%) | NI | NI |
|  |  | Golimumab |  |  | H1N1 |  | 7 (41,2%) | NI | NI |
|  |  |  |  |  | H3N2 |  | 10 (58,8%) | NI | NI |
|  |  |  |  |  | B |  | 5 (29,4%) | NI | NI |
|  |  | Abatacept |  | 8 | H1N1 |  | 12 (32,4%) | NI | NI |
|  |  |  |  |  | H3N2 |  | 26 (70,3%) | NI | NI |
|  |  |  |  |  | B |  | 10 (27,0%) | NI | NI |
|  |  | Tocilizumab |  |  | H1N1 |  | 49 (61,3%) | NI | NI |
|  |  |  |  |  | H3N2 |  | 72 (90,0%) | NI | NI |
|  |  |  |  |  | B |  | 44 (55,0%) | NI | NI |
|  |  | Golimumab |  |  | H1N1 |  | 8 (47,1%) | NI | NI |
|  |  |  |  |  | H3N2 |  | 13 (76,4%) | NI | NI |
|  |  |  |  |  | B |  | 5 (29,4%) | NI | NI |
|  |  | Abatacept |  | 4 | H1N1 | Seroconversion | 30 (81,0%) | NI | NI |
|  |  |  |  |  | H3N2 |  | 6 (16,2%) | NI | NI |
|  |  |  |  |  | B |  | 1 (2,7%) | NI | NI |
|  |  | Tocilizumab |  |  | H1N1 |  | 23 (28,8%) | NI | NI |
|  |  |  |  |  | H3N2 |  | 36 (45,0%) | NI | NI |
|  |  |  |  |  | B |  | 26 (32,5%) | NI | NI |
|  |  | Golimumab |  |  | H1N1 |  | 5 (29,4%) | NI | NI |
|  |  |  |  |  | H3N2 |  | 4 (23,5%) | NI | NI |
|  |  |  |  |  | B |  | 3 (17,6%) | NI | NI |
|  |  | Abatacept |  | 8 | H1N1 |  | 4 (10,8%) | NI | NI |
|  |  |  |  |  | H3N2 |  | 10 (27,0%) | NI | NI |
|  |  |  |  |  | B |  | 1 (2,7%) | NI | NI |
|  |  | Tocilizumab |  |  | H1N1 |  | 27 (33,8%) | NI | NI |
|  |  |  |  |  | H3N2 |  | 43 (53,8%) | NI | NI |
|  |  |  |  |  | B |  | 32 (40,0%) | NI | NI |
|  |  | Golimumab |  |  | H1N1 |  | 6 (35,3%) | NI | NI |
|  |  |  |  |  | H3N2 |  | 8 (47,1%) | NI | NI |
|  |  |  |  |  | B |  | 3 (17,6%) | NI | NI |
| Kaine 2007 | RA | Adalimumab | Polyvalent pneumococcal polysaccharide (PPV23) | 4 | Total | Seroprotection | 37 (37,4%) | 44 (40,4%) | NI |
|  |  |  |  |  | 9V |  | 45 (45,5%) | 46 (42,2%) | NI |
|  |  |  |  |  | 14 |  | 41 (41,4%) | 54 (49,5%) | NI |
|  |  |  |  |  | 18C |  | 46 (46,5%) | 53 (48,6%) | NI |
|  |  |  |  |  | 19F |  | 36 (36,4%) | 39 (35,8%) | NI |
|  |  |  |  |  | 23F |  | 44 (44,4%) | 41 (37,6%) | NI |
|  |  |  | Influenza (Trivalent) | 4 | Total |  | 51 (51,5%) | 69 (63,3%) | NI |
|  |  |  |  |  | H1N1 |  | 50 (50,5%) | 61 (56,0%) | NI |
|  |  |  |  |  | H3N2 |  | 58 (58,6%) | 74 (67,9%) | NI |
|  |  |  |  |  | B (Hong Kong) |  | 48 (48,5%) | 66 (60,6%) | NI |
| Kapetanovic 2007 | RA | TNFi (Etanercept and Infliximab) | Influenza (Trivalent) | 6 | H1N1 | Seroprotection | 36 (58,1%) | 33 (89,2%) | 14 (77,8%) |
|  |  |  |  |  | H3N2 |  | 46 (74,2%) | 28 (75,7%) | 13 (72,2) |
|  |  |  |  |  | B |  | 54 (87,1%) | 35 (94,6%) | 12 (66,7%) |
| Kapetanovic 2006 | RA | TNFi (Etanercept and Infliximab) | Polyvalent pneumococcal polysaccharide (PPV23) | 6 | 6B | Seroconversion | 42 (67,6%) | 9 (23,6%) | 26 (54,7%) |
|  |  |  |  |  | 23F |  | 42 (68,0%) | 13 (35,0%) | 24 (51,0%) |
|  |  |  |  |  | Total |  | 31 (50,0%) | 5 (14,0%) | 18 (37,8%) |
| Kapetanovic 2011b | RA | TNFi | 7-valent pneumococcal conjugate | 6 | 6B | Seroconversion | 38 (48,0%) | 23 (27,0%) | NI |
|  |  |  |  |  | 23F |  | 44 (56,0%) | 41 (48,5%) | NI |
|  |  |  |  |  | Total |  | 29 (36,6%) | 18 (21,4%) | NI |
|  | SpA |  |  |  | 6B |  | 46 (55,0%) | 44 (52,0%) | NI |
|  |  |  |  |  | 23F |  | 61 (74,0%) | 68 (80,0%) | NI |
|  |  |  |  |  | Total |  | 41 (49,8%) | 40 (47,2%) | NI |
| Kapetanovic 2011a | RA | NI | Polyvalent pneumococcal polysaccharide (PPV23) | 6 | 6B | Seroconversion | 42 (68,0%) | 13 (35,2%) | 24 (50,8%) |
|  |  |  |  |  | 23F |  | 42 (68,0%) | 9 (24,4%) | 26 (55,4%) |
|  |  |  |  |  | Total |  | 31 (50,0%) | 5 (14,0%) | 19 (40,0%) |
| Kapetanovic 2013 | RA | TNFi | 7-valent pneumococcal conjugate | 78 | Total | Seroprotection | 16 (32,0%) | 23 (40,0%) | NI |
|  | SpA |  |  |  |  |  | 28 (60,0%) | 30 (70,0%) | NI |
| Kapetanovic 2014 | RA | TNFi | Influenza (H1N1) | 32 | H1N1 | Seroconversion | 20 (53,0%) | 21 (42,0%) | NI |
|  |  | Abatacept |  |  |  |  | 1 (20,0%) | NI | NI |
|  |  | Tocilizumab |  |  |  |  | 1 (50,0%) | NI | NI |
|  | SpA |  |  |  |  |  | 31 (76,0%) | 24 (59,0%) | NI |
|  | RA | TNFi |  |  |  | Seroprotection | 22 (58,0%) | 25 (50,0%) | NI |
|  |  | Abatacept |  |  |  |  | 2 (40,0%) | NI | NI |
|  |  | Tocilizumab |  |  |  |  | 2 (100,0%) | NI | NI |
|  | SpA |  |  |  |  |  | 31 (76,0%) | 27 (66,0%) | NI |
| Kivitz 2014 | RA | Certolizumab | Polyvalent pneumococcal polysaccharide (PPV23) | 4 | Total | Seroconversion | 57 (53,3%) | NI | 64 (58,2%) |
|  |  |  | Influenza (Trivalent) |  |  |  | 54 (50,5%) | NI | 59 (54,1%) |
|  |  |  | Polyvalent pneumococcal polysaccharide (PPV23) |  | 6B |  | 41 (38,3%) | NI | 50 (45,5%) |
|  |  |  |  |  | 9V |  | 61 (57,0%) | NI | 54 (49,1%) |
|  |  |  |  |  | 14 |  | 50 (46,7%) | NI | 68 (61,8%) |
|  |  |  |  |  | 18C |  | 69 (64,5%) | NI | 59 (53,6%) |
|  |  |  |  |  | 19F |  | 47 (43,9%) | NI | 57 (51,8%) |
|  |  |  |  |  | 23F |  | 42 (39,3%) | NI | 53 (48,2%) |
|  |  |  | Influenza (Trivalent) |  | H1N1 |  | 49 (45,8%) | NI | 55 (50,5%) |
|  |  |  |  |  | H3N2 |  | 61 (57,0%) | NI | 77 (70,6%) |
|  |  |  |  |  | B |  | 48 (44,9%) | NI | 48 (44,0%) |
| Kogure 2014 | RA | NI | Influenza (Trivalent) | 4 | H1N1 | Seroprotection | 36 (63,2%) | NI | NI |
|  |  |  |  |  | H3N2 |  | 46 (80,7%) | NI | NI |
|  |  |  |  |  | B |  | 15 (26,3%) | NI | NI |
| Kostianovsky 2012 | Autoimmune diseases | NI | Influenza (Trivalent) | 3 | Total | Seroprotection | 139 (80,3%) | NI | 127 (64,5%) |
|  |  |  |  |  |  | Seroconversion | 55 (39,6%) | NI | 91 (71,7%) |
| Kubota 2007 | RA | TNFi (Etanercept and Infliximab) | Influenza (Trivalent) | 6 | H1N1 | Seroconversion | 12 (44,4%) | 8 (22,2%) | 9 (17,3%) |
|  |  |  |  |  | H3N2 |  | 12 (44,4%) | 12 (33,3%) | 13 (25,0%) |
|  |  |  |  |  | B |  | 8 (29,6%) | 8 (22,2%) | 5 (9,6%) |
| Lakota 2019 | Autoimmune diseases | TNFi (Etanercept, Infliximab, and Adalimumab), non-TNFi (Tocilizumab), Rituximab | Influenza (Trivalent) | 26 | H1N1 | Seroprotection | 122 (89,0%) | NI | 50 (92,0%) |
|  |  |  |  |  | H3N2 |  | 137 (100,0%) | NI | 54 (100,0%) |
|  |  |  |  |  | B |  | 134 (98,0%) | NI | 54 (100,0%) |
|  |  |  |  |  | Total | Seroconversion | 15 (12,0%) | NI | 1 (6,0%) |
|  |  |  |  |  | H1N1 |  | 62 (50,4%) | NI | 5 (29,4%) |
|  |  |  |  |  | H3N2 |  | 35 (28,5%) | NI | 8 (47,1%) |
|  |  |  |  |  | B |  | 41 (33,3%) | NI | 10 (58,8%) |
| Migita 2015a | RA | Abatacept | Polyvalent pneumococcal polysaccharide (PPV23) | 6 | 6B | Seroconversion | 9 (43,0%) | 31 (56,0%) | 25 (72,0%) |
|  |  |  |  |  | 23F |  | 14 (67,0%) | 36 (65,0%) | 29 (83,0%) |
|  |  |  |  |  | Total |  | 9 (43,0%) | 28 (51,0%) | 24 (68,0%) |
| Migita 2015b | RA | Golimumab | Polyvalent pneumococcal polysaccharide (PPV23) | 6 | 6B | Seroconversion | 6 (25,0%) | 31 (56,0%) | 25 (72,0%) |
|  |  |  |  |  | 23F |  | 11 (46,0%) | 36 (65,0%) | 29 (83,0%) |
|  |  |  |  |  | Total |  | 5 (21,0%) | 28 (51,0%) | 24 (68,0%) |
| Mischlinger 2022 | RA, SpA, PsA, Vasculitis | TNFi | Tetanus | Short-term | Total | Seroprotection | 42 (100,0%) | NI | 164 (97,0%) |
|  |  |  |  | Long-term |  |  | 32 (76,2%) | NI | 122 (72,2%) |
|  |  |  | Diphtheria | Short-term |  |  | 31 (73,8%) | NI | 110 (65,5%) |
|  |  |  |  | Long-term |  |  | 4 (9,5%) | NI | 8 (4,8%) |
|  |  | Other bDMARDs | Tetanus | Short-term |  |  | 22 (84,6%) | NI | 164 (97,0%) |
|  |  |  |  | Long-term |  |  | 12 (46,2%) | NI | 122 (72,2%) |
|  |  |  | Diphtheria | Short-term |  |  | 10 (38,5%) | NI | 110 (65,5%) |
|  |  |  |  | Long-term |  |  | 0 (0,0%) | NI | 8 (4,8%) |
|  |  | TNFi and MTX | Tetanus | Short-term |  |  | 19 (86,4%) | NI | 164 (97,0%) |
|  |  |  |  | Long-term |  |  | 15 (68,2%) | NI | 122 (72,2%) |
|  |  |  | Diphtheria | Short-term |  |  | 10 (45,5%) | NI | 110 (65,5%) |
|  |  |  |  | Long-term |  |  | 1 (4,6%) | NI | 8 (4,8%) |
| Mori 2012 | RA | Tocilizumab | Influenza (Trivalent) | 6 | H1N1 | Seroprotection | 50 (81,0%) | 45 (69,0%) | 13 (72,0%) |
|  |  |  |  |  | H3N2 |  | 62 (100,0%) | 45 (69,0%) | 13 (72,0%) |
|  |  |  |  |  | B |  | 60 (96,0%) | 50 (77,0%) | 12 (67,0%) |
|  |  |  |  |  | H1N1 | Seroconversion | 40 (64,0%) | 33 (50,0%) | 8 (45,0%) |
|  |  |  |  |  | H3N2 |  | 46 (74,0%) | 32 (49,0%) | 9 (52,0%) |
|  |  |  |  |  | B |  | 34 (55,0%) | 27 (42,0%) | 16 (91,0%) |
| Mori 2013 | RA | Tocilizumab | Polyvalent pneumococcal polysaccharide (PPV23) | 6 | Total | Seroconversion | 23 (46,0%) | 20 (33,0%) | 5 (21,0%) |
|  |  |  |  |  | 6B |  | 28 (56,0%) | 23 (37,0%) | 9 (37,0%) |
|  |  |  |  |  | 23F |  | 36 (72,0%) | 35 (57,0%) | 16 (67,0%) |
| Nguyen 2017 | RA | TNFi, non-TNFi, RTX | Pneumococcal (PCV13 and PPV23) | 4 | 1 | Seroconversion | 51 (81,0%) | 24 (73,0%) | NI |
|  |  |  |  |  | 3 |  | 34 (54,0%) | 15 (45,0%) | NI |
|  |  |  |  |  | 4 |  | 41 (65,0%) | 19 (58,0%) | NI |
|  |  |  |  |  | 5 |  | 39 (62,0%) | 16 (48,0%) | NI |
|  |  |  |  |  | 6B |  | 49 (78,0%) | 20 (61,0%) | NI |
|  |  |  |  |  | 7F |  | 59 (94,0%) | 31 (94,0%) | NI |
|  |  |  |  |  | 9V |  | 50 (70,0%) | 21 (64,0%) | NI |
|  |  |  |  |  | 14 |  | 55 (87,0%) | 29 (88,0%) | NI |
|  |  |  |  |  | 18C |  | 57 (90,0%) | 29 (88,0%) | NI |
|  |  |  |  |  | 19A |  | 56 (89,0%) | 32 (92,0%) | NI |
|  |  |  |  |  | 19F |  | 60 (95,0%) | 31 (94,0%) | NI |
|  |  |  |  |  | 23F |  | 57 (90,0%) | 30 (91,0%) | NI |
| Nived 2017 | Systemic vasculitis | TNFi | 13-valent pneumococcal conjugate (PCV13) | 6 | Total | Seroprotection | 24 (49,0%) | NI | 30 (61,0%) |
|  |  |  |  |  | 6B |  | 32 (65,0%) | NI | 35 (71,0%) |
|  |  |  |  |  | 23F |  | 32 (65,0%) | NI | 37 (76,0%) |
| Polachek 2015 | PsA and Pso | TNFi (Etanercept, Infliximab, Adalimumab, and Golimumab) | Influenza (Trivalent) | 6 | H1N1 | Seroprotection | 66 (98,5%) | NI | 28 (93,3%) |
|  |  |  |  |  | H3N2 |  | 63 (94,0%) | NI | NI |
|  |  |  |  |  | B |  | 67 (100,0%) | NI | NI |
|  |  |  |  |  | H1N1 | Seroconversion | 30 (44,8%) | NI | 6 (20,0%) |
|  |  |  |  |  | H3N2 |  | 28 (41,8%) | NI | 8 (26,7%) |
|  |  |  |  |  | B |  | 12 (17,9%) | NI | 3 (10,0%) |
| Rasmussen 2023 | RA, SpA, and PsA | TNFi, non-TNFi | Polyvalent pneumococcal polysaccharide (PPV23) | 12 | Total | Seroprotection | 80 (36,0%) | 3 (25,0%) | NI |
| Ribeiro 2013 | RA | Abatacept | Influenza (H1N1) | 3 | Total | Seroconversion | 0 (0%) | 19 (58,0%) | 36 (66,0%) |
|  |  |  |  |  |  | Seroprotection | 1 (9,0%) | 19 (58,0%) | 36 (66,0%) |
| Richi 2019 | PsA and AS | Secukinumab | Influenza (Trivalent) | 4 | H1N1 | Seroconversion | 10 (58,82%) | NI | 7 (53,85%) |
|  |  |  |  |  | H3N2 |  | 2 (11,69%) | NI | 1 (7,69%) |
|  |  |  |  |  | B |  | 6 (35,29%) | NI | 6 (46,15%) |
| Richi 2021b | Autoimmune diseases | TNFi (Etanercept, Infliximab, Adalimumab, Golimumab, and Certolizumab), Tocilizumab, Rituximab, Anakinra | Influenza (Trivalent) | 4 | A | Soropositividade | 60 (88,2%) | 33 (71,7%) | 43 (89,6%) |
|  |  |  |  |  | B |  | 29 (42,7%) | 19 (41,3%) | 26 (54,2%) |
| Richi 2021a | Autoimmune diseases | TNFi (Etanercept, Infliximab, Adalimumab, Golimumab, and Certolizumab), non-TNFi (Tocilizumab and Abatacept), Rituximab, Anakinra | Pneumococcal (PCV13 and PPV23) | 4 | 1 | Seroconversion | 80 (44,0%) | NI | NI |
|  |  |  |  |  | 3 |  | 86 (47,3%) | NI | NI |
|  |  |  |  |  | 7F |  | 65 (35,7%) | NI | NI |
|  |  |  |  |  | 14 |  | 81 (44,5%) | NI | NI |
|  |  |  |  |  | 19A |  | 65 (35,7%) | NI | NI |
|  |  |  |  |  | 19F |  | 60 (33,0%) | NI | NI |
| Romão 2020 | Autoimmune diseases | TNFi, non-TNFi (Tocilizumab and Abatacept), Rituximab, Belimumab, Anakinra | Hepatitis B | 4 | Total | Seroconversion | 20 (30,0%) | NI | 68 (97,0%) |
| Rosdahl 2020 | RA | TNFi | Hepatitis A | 4 | Total | Seroprotection | 44 (64,0%) | NI | NI |
|  |  |  |  | 8 |  |  | 61 (84,0%) | NI | NI |
|  |  |  | Hepatitis A (Normal doses) | 4 |  | Seroconversion | 19 (60,0%) | NI | 44 (94,0%) |
|  |  |  | Hepatitis A (Booster dose at 1st month) |  |  |  | 25 (68,0%) | NI | NI |
|  |  |  | Hepatitis A (Normal doses) | 8 |  |  | 29 (90,6%) | NI | 40 (85,0%) |
|  |  |  | Hepatitis A (Booster dose at 1st month) |  |  |  | 32 (86,5%) | NI | NI |
| Sergeeva 2017 | RA | TNFi | Polyvalent pneumococcal polysaccharide (PPV23) | 52 | Total | Immunogenicity | 48 (61,0%) | NI | NI |
|  |  |  |  |  |  |  |  | NI | 22 (70,0%) |
| Solay 2019 | Autoimmune diseases | TNFi (Etanercept, Infliximab, Adalimumab, and Golimumab), non-TNFi (Ustekinumab) | Hepatitis B | NI | Total | Seroconversion | 58 (53,2%) | NI | NI |
| Stapleton | RA | TNFi (Etanercept, Infliximab, and Adalimumab) | Influenza (Trivalent) | 1 | H1N1 | Seroconversion | 9 (36,0%) | NI | 5 (20,0%) |
|  |  |  |  | 3 |  |  | 17 (48,0%) | NI | 7 (28,0%) |
|  |  |  |  | 26 |  |  | 1 (4,0%) | NI | 5 (20,0%) |
|  |  |  |  | 1 | H3N2 |  | 7 (28,0%) | NI | 8 (32,0%) |
|  |  |  |  | 3 |  |  | 10 (40,0%) | NI | 10 (40,0%) |
|  |  |  |  | 26 |  |  | 5 (21,0%) | NI | 3 (12,0%) |
|  |  |  |  | 1 | B |  | 5 (20,0%) | NI | 2 (8,0%) |
|  |  |  |  | 3 |  |  | 7 (28,0%) | NI | 6 (24,0%) |
|  |  |  |  | 26 |  |  | 2 (8,0%) | NI | 2 (8,0%) |
| Tarasova 2018 | SLE | NI | Polyvalent pneumococcal polysaccharide (PPV23) | NI | Total | Immunogenicity | 25 (83,0%) | NI | NI |
|  |  |  |  |  |  |  | 19 (63,0%) | NI | NI |
| Tarasova 2019 | SLE | NI | Polyvalent pneumococcal polysaccharide (PPV23) | 4 | Total | Immunogenicity | 28 (82,0%) | NI | NI |
|  |  |  |  | 12 |  |  | 33 (97,0%) | NI | NI |
|  |  |  |  | 52 |  |  | 22 (65,0%) | NI | NI |
| Tarasova 2020 | SLE | NI | Polyvalent pneumococcal polysaccharide (PPV23) | 8 | Total | Immunogenicity | 41 (78,7%) | NI | NI |
|  |  |  |  | 52 |  |  | 32 (61,5%) | NI | NI |
| Tarasova 2021 | SLE | Belimumab and Rituximab | Polyvalent pneumococcal polysaccharide (PPV23) | 4 | Total | Immunogenicity | 47 (78,7%) | NI | NI |
|  |  |  |  |  |  |  | 9 (23,0%) | 25 (68,0%0 | NI |
| Tsuru 2013a | RA | Tocilizumab | Influenza (Trivalent) | 12 | Total | Immunogenicity | 21 (100,0%,) | NI | NI |
|  |  |  |  |  | H1N1 |  | 10 (35,7%) | 18 (75,0%) | NI |
|  |  |  |  |  | H3N2 |  | 13 (46,4%) | 13 (54,2%) | NI |
|  |  |  |  |  | B |  | 18 (64,3%) | 19 (79,2%) | NI |
|  |  |  |  |  | H1N1 | Seroprotection | 26 (92,9%) | 22 (91,7%) | NI |
|  |  |  |  |  | H3N2 |  | 25 (89,3%) | 23 (95,8%) | NI |
|  |  |  |  |  | B |  | 22 (78,6%) | 21 (87,5%) | NI |
|  |  |  |  |  | H1N1 | Seroconversion | 5 (71,4%) | 10 (83,3%) | NI |
|  |  |  |  |  | H3N2 |  | 3 (50,0%) | 10 (90,9%) | NI |
|  |  |  |  |  | B |  | 10 (62,5%) | 13 (81,3%) | NI |
|  |  | TNFi (Etanercept and Infliximab) |  |  | H1N1 | Immunogenicity | 6 (40,0%) | 18 (75,0%) | NI |
|  |  |  |  |  | H3N2 |  | 8 (53,3%) | 13 (54,2%) | NI |
|  |  |  |  |  | B |  | 4 (26,7%) | 19 (79,2%) | NI |
|  |  |  |  |  | H1N1 | Seroprotection | 11 (73,3%) | 22 (91,7%) | NI |
|  |  |  |  |  | H3N2 |  | 12 (80,0%) | 23 (95,8%) | NI |
|  |  |  |  |  | B |  | 8 (53,3%) | 21 (87,5%) | NI |
|  |  |  |  |  | H1N1 | Seroconversion | 3 (42,9%) | 10 (83,3%) | NI |
|  |  |  |  |  | H3N2 |  | 6 (66,7%) | 10 (90,9%) | NI |
|  |  |  |  |  | B |  | 3 (30,0%) | 13 (81,3%) | NI |
| Tsuru 2013 | RA | Infliximab | Influenza (H1N1) | 4 | H1N1 | Immunogenicity | 8 (30,0%) | 35 (60,0%) | 18 (59,0%) |
|  |  | Etanercept |  |  |  |  | 10 (53,0%) | NI | NI |
|  |  | Tocilizumab |  |  |  |  | 17 (77,0%) | NI | NI |
|  |  | Infliximab |  |  |  | Seroprotection | 6 (22,0%) | 24 (40,0%) | 10 (34,0%) |
|  |  | Etanercept |  |  |  |  | 6 (35,0%) | NI | NI |
|  |  | Tocilizumab |  |  |  |  | 11 (50,0%) | NI | NI |
|  |  | Infliximab |  |  |  | Seroconversion | 10 (37,0%) | 35 (59,0%) | 17 (55,0%) |
|  |  | Etanercept |  |  |  |  | 10 (53,0%) | NI | NI |
|  |  | Tocilizumab |  |  |  |  | 14 (64,0%) | NI | NI |
|  |  | Infliximab |  | 8 |  | Immunogenicity | 15 (56,0%) | 30 (74,0%) | 21 (69,0%) |
|  |  | Etanercept |  |  |  |  | 12 (65,0%) | NI | NI |
|  |  | Tocilizumab |  |  |  |  | 17 (76,0%) | NI | NI |
|  |  | Infliximab |  |  |  | Seroprotection | 10 (37,0%) | 28 (47,0%) | 14 (45,0%) |
|  |  | Etanercept |  |  |  |  | 8 (47,0%) | NI | NI |
|  |  | Tocilizumab |  |  |  |  | 10 (48,0%) | NI | NI |
|  |  | Infliximab |  |  |  | Seroconversion | 13 (48,0%) | 40 (68,0%) | 20 (66,0%) |
|  |  | Etanercept |  |  |  |  | 12 (65,0%) | NI | NI |
|  |  | Tocilizumab |  |  |  |  | 16 (71,0%) | NI | NI |
| Venerito 2023 | RA | Abatacept and Rituximab | Herpes Zoster | 12 | Total | Seroconversion | 24 (92,3%) | 26 (100,0%) | NI |
| Visvanathan 2007 | RA | Infliximab | Polyvalent pneumococcal polysaccharide (PPV23) | 4 | 1 | Immunogenicity | 4 (20,0%) | NI | 3 (21,0%) |
|  |  |  |  |  | 3 | NI | 5 (25,0%) | NI | 1 (7,0%) |
|  |  |  |  |  | 4 | NI | 4 (20,0%) | NI | 4 (28,0%) |
|  |  |  |  |  | 6B | NI | 6 (30,0%) | NI | 2 (14,0%) |
|  |  |  |  |  | 7F | NI | 5 (25,0%) | NI | 2 (14,0%) |
|  |  |  |  |  | 8 | NI | 7 (35,0%) | NI | 4 (28,0%) |
|  |  |  |  |  | 9N | NI | 9 (45,0%) | NI | 7 (50,0%) |
|  |  |  |  |  | 12F | NI | 2 (10,0%) | NI | 6 (43,0%) |
|  |  |  |  |  | 14 | NI | 7 (35,0%) | NI | 2 (14,0%) |
|  |  |  |  |  | 18C | NI | 8 (40,0%) | NI | 5 (35,0%) |
|  |  |  |  |  | 19F | NI | 5 (25,0%) | NI | 2 (14,0%) |
|  |  |  |  |  | 23F | NI | 4 (20,0%) | NI | 6 (43,0%) |
|  |  |  |  |  | 1 | NI | 9 (25,0%) | NI | 3 (21,0%) |
|  |  |  |  |  | 3 | NI | 11 (31,0%) | NI | 1 (7,0%) |
|  |  |  |  |  | 4 | NI | 12 (33,0%) | NI | 4 (28,0%) |
|  |  |  |  |  | 6B | NI | 4 (11,0%) | NI | 2 (14,0%) |
|  |  |  |  |  | 7F | NI | 17 (47,0%) | NI | 2 (14,0%) |
|  |  |  |  |  | 8 | NI | 10 (28,0%) | NI | 4 (28,0%) |
|  |  |  |  |  | 9N | NI | 7 (20,0%) | NI | 7 (50,0%) |
|  |  |  |  |  | 12F | NI | 8 (22,0%) | NI | 6 (43,0%) |
|  |  |  |  |  | 14 | NI | 11 (31,0%) | NI | 2 (14,0%) |
|  |  |  |  |  | 18C | NI | 17 (47,0%) | NI | 5 (35,0%) |
|  |  |  |  |  | 19F | NI | 12 (33,0%) | NI | 2 (14,0%) |
|  |  |  |  |  | 23F | NI | 10 (28,0%) | NI | 6 (43,0%) |

**QUESTION 6 - What is the impact of the RTX use on the response to inactivated vaccine in patients with immune-mediated rheumatic diseases?**

Table 1. Main characteristics of the included studies.

| **Reference** | **Local** | **Journal** | **Study design** | **Immune-mediated rheumatic disease** | **Dosage RTX** | **Inactivated vaccine** | **Follow-up**  **(weeks)** |
| --- | --- | --- | --- | --- | --- | --- | --- |
| Adler et al., 2012 | France, Europe | Rheumatology | Clinical trial | RA, SpA, VAS or CTD |  | Adjuvanted A/H1N1 Influenza Vaccine | 26 |
| Alcocer et al, 2015 | Spain, Europe | Annals of the Rheumatic Diseases | Cohort | RA, SpA, Pso, IID or MCTD |  | Influenza virus A and B, and hepatitis B virus vaccine (HBV) | 6 |
| Arad et al., 2011 | Israel, Asia (also considered part of the Middle East) | Vaccine | Clinical trial | RA | 2 × 1000 mg | Trivalent Influenza Subunit Vaccine | 6 |
| Bingham et al., 2010 | USA (United States), North America | Arthritis & Rheumatism | Clinical trial | RA |  | Tetanus toxoid adsorbed vaccine and 23-Valent Polysaccharide Pneumococcal Vaccine (PPV23) | 36 |
| Bühler et al., 2019 | Germany, Europe | Rheumatology | Cohort | RA, SpA/PsA, BD and ANCA-AAV |  | Diphtheria/Tetanus Vaccine | 13 |
| Chen et al., 2018 | People's Republic of China, Asia | International Journal of Rheumatic Diseases | Cohort | RA |  | Influenza vaccine | 104 |
| Colmegna et al., 2020 | Canada, North America | Lancet Rheumatology | Clinical trial | RA |  | Trivalent inactivated influenza vaccine (HD-TIV) and quadrivalent influenza vaccine (SD-QIV) | 57 |
| Gabay et al., 2011 | Switzerland, Europe | Arthritis & Rheumatism | Cohort | RA, SpA or AIRD |  | Adjuvanted A/H1N1 Influenza Vaccine | 43 |
| Garrido et al., 2022 | Netherlands, Europe | Vaccines | Cohort | UC, RA, PsA and AS |  | 13-Valent Protein-Conjugated Pneumococcal Vaccine (PCV13) and 23-Valent Polysaccharide Pneumococcal Vaccine (PPV23) | 52 |
| Intongkam et al., 2019 | Thailand, Asia | Journal of Clinical Rheumatology | Clinical trial | RA |  | Hepatitis B virus vaccine (HBV) | 32 |
| Kapetanovic et al., 2014 | Sweden, Europe | Arthritis Research & Therapy | Cohort | RA |  | Adjuvanted pH1N1 Vaccine | 22 |
| Kapetanovicet al., 2013 | Sweden, Europe | Arthritis Research & Therapy | Cohort | RA |  | Heptavalent Pneumococcal Conjugate Vaccine | 6 |
| Kostianovsky et al., 2012 | France, Europe | Clinical and Experimental Rheumatology | Clinical trial | SNV, SSc and pSS |  | Seasonal (SFV) and A/H1N1 influenza vaccines (HFV) | 26 |
| Lakota et al., 2015 | Switzerland, Europe | Scientific Abstracts | Clinical trial | RA |  | Trivalent seasonal influenza vaccine | 26 |
| Lakota et al., 2019 | Slovenia, Europe | Clinical Rheumatology | Clinical trial | RA,PsA, AS, MCTD, RA and AOSD |  | Trivalent seasonal influenza vaccine | 26 |
| Mischlinger et al., 2022 | Switzerland, Europe | Vaccine | Cohort | RA, SpA, PsA or VAS (BD or ANCA-AAV) |  | Diphtheria/tetanus (dT)-vaccine |  |
| Morgan et al., 2016 | UK (United Kingdom), Europe | Arthritis care & research | Cohort | SV |  | Pneumococcal 7-valent conjugate, Haemophilus influenzae type b, and meningococcal group C conjugate vaccine and meningococcal polysaccharide groups A, C, Y, and W135 vaccines | 104 |
| Müller et al., 2013 | Switzerland, Europe | Clinical and Experimental Immunology | Cohort | ASS, ITP, RA and pSS | 1000mg | Split virion inactivated vaccine (Pandemrx) | 8 |
| Nagel et al., 2023 | Sweden, Europe | Vaccine | Clinical trial | RA and SpA |  | Hepavalent Conjugated Pneumococcal Vaccine (PCV7) | 521 |
| Nguyen et al., 2017 | Denmark, Europe | The Journal of rheumatology | Clinical trial | RA |  | 23-Valent Polysaccharide Pneumococcal Vaccine (PPV23) and 13-Valent Protein-Conjugated Pneumococcal Vaccine (PCV13) | 24 |
| Nived et al., 2016 | Sweden, Europe | Annals of the Rheumatic Diseases | Clinical trial | SV |  | 13-Valent Protein-Conjugated Pneumococcal Vaccine (PCV13) | 6 |
| Nived et al., 2017 | Sweden, Europe | Vaccine | Clinical trial | SV |  | 13-Valent Protein-Conjugated Pneumococcal Vaccine (PCV13) | 8 |
| Nived et al., 2020 | Sweden, Europe | Arthritis Research & Therapy | Clinical trial | AIRD |  | 23-Valent Polysaccharide Pneumococcal Vaccine (PPV23) and 13-Valent Protein-Conjugated Pneumococcal Vaccine (PCV13) | 8 |
| Oren et al., 2008 | Israel, Asia (also considered part of the Middle East) | Annals of the rheumatic | Clinical trial | RA | 1000mg | Adjuvanted A/H1N1 Influenza Vaccine | 26 |
| Park et al., 2018 | South Korea, Asia | Annals of the Rheumatic Diseases | Clinical trial | RA |  | Seasonal Quadrivalent Influenza Vaccine (GC Flu) | 4 |
| Rehnberg et al., 2010 | Sweden, Europe | Arthritis Research & Therapy | Cohort | RA | 1000mg | 23-Valent Polysaccharide Pneumococcal Vaccine (PPV23) | 26 |
| Rehnberg et al., 2009 | Sweden, Europe | Arthritis Research & Therapy | Clinical trial | RA |  | 23-Valent Polysaccharide Pneumococcal Vaccine (PPV23) | 26 |
| Richi et al., 2019 | Spain, Europe | Medicina Clinica | Cohort | AIIRD, RA, PsA,SpA, UA or CTD, Pso or IBD |  | Seasonal Inactivated Trivalent Influenza Vaccine (TIV) | 16 |
| Richi et al., 2020 | Spain, Europe | Clinical Rheumatology | Cohort | Pso or IBD and AIRDs |  | Hepatitis B vaccine | 4 |
| Richi et al., 2021 | Spain, Europe | Vaccines | Cohort | RA, SpA, PsA, UA or CTD, Pso or IBD |  | 23-Valent Polysaccharide Pneumococcal Vaccine (PPV23) and 13-Valent Protein-Conjugated Pneumococcal Vaccine (PCV13) | 4 |
| Tarasova et al., 2021 | Russia, Asia (partly in Europe and partly in Asia) | Annals of the Rheumatic Diseases | Clinical trial | SLE |  | 23-Valent Polysaccharide Pneumococcal Vaccine (PPV23) | 46 |
| Tarasova et al., 2016 | Russia, Asia (partly in Europe and partly in Asia) | Annals of the Rheumatic Diseases | Clinical trial | SLE | 500-1000mg | 23-Valent Polysaccharide Pneumococcal Vaccine (PPV23) | 43 |
| Tarasova et al., 2017 | Russia, Asia (partly in Europe and partly in Asia) | Annals of the rheumatic | Clinical trial | SLE |  | 23-Valent Polysaccharide Pneumococcal Vaccine (PPV23) | 56 |
| Tarasova et al., 2018 | Russia, Asia (partly in Europe and partly in Asia) | Annals of the Rheumatic Diseases | Clinical trial | SLE |  | 23-Valent Polysaccharide Pneumococcal Vaccine (PPV23) | 52 |
| Tarasova et al., 2019 | Russia, Asia (partly in Europe and partly in Asia) | Annals of the Rheumatic Diseases | Clinical trial | SLE | 500-1000mg | 23-Valent Polysaccharide Pneumococcal Vaccine (PPV23) | 52 |
| Tarasova et al., 2020 | Russia, Asia (partly in Europe and partly in Asia) | Annals of the Rheumatic Diseases | Clinical trial | SLE |  | 23-Valent Polysaccharide Pneumococcal Vaccine (PPV23) | 52 |
| Tarasova et al., 2020 | Russia, Asia (partly in Europe and partly in Asia) | Antibiotiki i Khimioterapiya | Clinical trial | SLE |  | 23-Valent Polysaccharide Pneumococcal Vaccine (PPV23) | 52 |
| Tarasova et al., 2022 | Russia, Asia (partly in Europe and partly in Asia) | Annals Rheumatic Diseases | Clinical trial | SV |  | 23-Valent Polysaccharide Pneumococcal Vaccine (PPV23) | 26 |
| Van Assen et al., 2010 | Netherlands, Europe | Arthritis Research & Therapy | Clinical trial | RA | 1000mg | Trivalent subunit influenza vaccine | 43 |
| Venerito et al., 2023 | Italy, Europe | International journal of molecular sciences | Cohort | RA |  | Adjuvanted Recombinant Zoster Vaccine | 4 |
| Westra et al.,2014 | Netherlands, Europe | Clinical and Experimental Immunology | Clinical trial | RA |  | Trivalent subunit influenza vaccine | 43 |

**Legend:** Diseases: Adult-Onset Still's Disease (AOSD), Ankylosing spondylitis (AS), Antineutrophil cytoplasmic antibody associated vasculitis (ANCA- AAV), Antisynthetase syndrome (ASS), Autoimmune inflammatory rheumatic diseases (AIRD), Behçet disease (BD), Connective tissue diseases (CTD), Inflammatory bowel disease (IBD), Inflammatory intestinal disease (IID), Immunothrombopenia (ITP), Mixed connective tissue disease (MCTD), Primary Sjögren’s Syndrome (pSS), Psoriasis (Pso), Psoriatic arthritis (PsA), Rheumatoid arthritis (RA), Spondyloarthritis (SpA), Systemic lupus erythematosus (SLE), Systemic necrotising vasculitides (SNV), Systemic sclerosis (SSc), Systemic vasculitis (SV), Vasculitis (VAS), Ulcerative colitis (UC), Undifferentiated arthritis (UA)

**QUESTION 7. What is the effect of corticosteroid use on the response to inactivated vaccines in patients with immune-mediated rheumatic diseases?**

**Table 1.** Characteristics of the included studies.

| **First author and Year** | **Local** | **Study design** | **Immune-mediated rheumatic disease** | **Dostoge corticosteroids** | **Vaccine** | **Follow-up** |
| --- | --- | --- | --- | --- | --- | --- |
| Abu-Shakra 2002 | Israel | Cohort | SLE | 12 (2.5-40) | Influenza (Trivalent) | 12 weeks |
| Acharya 2019 | USA | Cohort | Autoimmune diseases | 1.63-1.79 | Herpes Zoster | 26 weeks |
| Adler 2012 | Switzerland | Case-control | RA, SpA, Vasculitis, and CTD | NI | Influenza (H1N1) | 26 weeks |
| Andrade 2012 | São Paulo, Brazil | Case-control | Systemic sclerosis | NI | Influenza (H1N1) | 3 weeks |
| Aydin 2020 | Turkey | Cohort | RA, AS, Pso, Reactive arthritis | NI | Hepatitis B | 26 weeks |
| Battafarano 1998 | USA | Cohort | SLE | NI | Polyvalent pneumococcal polysaccharide (PPV23), Influenza (B), and Tetanus | 12 weeks |
| Borba 2012 | São Paulo, Brazil | Case-control | SLE | 7.7 ± 11.3 | Influenza (H1N1) | 3 weeks |
| Brodman 1978 | USA | Clinical trial | SLE | 20 | Influenza (Bivalent) | 4 weeks |
| Broyde 2016 | Israel | Cohort | RA, AS, PsA, IBD-associated SpA, and others | NI | Polyvalent pneumococcal polysaccharide (PPV23) | 520 weeks |
| Caporuscio 2018 | Italy | Clinical trial | RA | NI | 13-valent pneumococcal conjugate (PCV13) | 4 weeks |
| Chen 2018 | Taiwan | Case-control | RA | NI | Influenza | 4 weeks |
| Coulson 2011 | UK | Cohort | RA | NI | Polyvalent pneumococcal polysaccharide (PPV23) | 520 weeks |
| Dagnew 2021 |  | Clinical trial | Autoimmune diseases | NI | Herpes Zoster | 4 weeks |
| Del Porto 2006 | Italy | Clinical trial | SLE and RA | NI | Influenza (Trivalent) | 4 weeks |
| Dhar 2017 | USA | Clinical trial | SLE | NI | Quadrivalent human papillomavirus (HPV) | 4 weeks |
| Elkayam 2002 | Israel | Case-control | RA and SLE | NI | Polyvalent pneumococcal polysaccharide (PPV23) | 9 weeks |
| Elkayam 2005 | Israel | Clinical trial | SLE | NI | Polyvalent pneumococcal polysaccharide (PPV23) | 8 weeks |
| Elkayam 2011 | Israel | Clinical trial | RA, SLE, PsA, and AS | NI | Influenza (H1N1) | 4 weeks |
| Evison 2009 | Switzerland | Clinical trial | Chronic idiopathic arthritis, SpA, Autoimmune connectivitis, Sarcoidosis, Vasculitis | NI | Influenza (Trivalent) | 6 weeks |
| Fernández-Prada 2022 | Spain | Cohort | RA, PsA, AS, Pso, IBD, SLE, and others | NI | Hepatitis B | 26 weeks |
| Fischer 2016 | Switzerland | Cohort | RA, SpA, Pso, PsA, CTD, Systemic vasculitis, and others | median: 10; IQR: 5-30 | Polyvalent pneumococcal polysaccharide (PPV23) | 52 weeks |
| Fomin 2006 | Israel | Case-control | RA | 8 ± 3.7 | Influenza (Trivalent) | 6 weeks |
| Gabay 2011 | Switzerland | Cohort | RA, SpA, SLE, and ANCA associated necrotizing vasculitis | NI | Influenza (H1N1) | 4 weeks |
| Garrido 2022 | Netherlands | Cohort | CD, UC, RA, Pso/PsA, Spondylitis ankylopoietica, Neurological autoimmune disease, other autoinflammatory disease, Solid organ transplant recipient | NI | Pneumococcal (PCV13 and PPV23) | 52 weeks |
| Gerard 2020 | France | Cohort | SLE | NI | Pneumococcal (PCV13 and PPV23) | 144 weeks |
| Grabar 2017 | France | Clinical trial | SLE | 7 ± 2 | Pneumococcal (PCV7 and PPV23) | 52 weeks |
| Hernandez 2015 | Philippines | Cohort | SLE | NI | Influenza and Pneumococcal | 8 weeks |
| Herron 1979 | USA | Clinical trial | RA, SLE, and other | NI | Influenza (H3N2) | 16 weeks |
| Holvast 2005 | Netherlands | Case-control | SLE | NI | Influenza (Trivalent) | 3 weeks |
| Holvast 2009a | Netherlands | Case-control | SLE | median: 5; IQR: 1.25-25 | Influenza (Trivalent) | 8 weeks |
| Holvast 2009b | Netherlands | Case-control | SLE | median: 5; IQR: 1.25-15 | Influenza (Trivalent) | 16 weeks |
| Intongkam 2018 | Thailand | Clinical trial | RA | 10 | Hepatitis B | 32 weeks |
| Izumi 2017 | Japan | Clinical trial | RA | 4.54 ± 2.79 | Polyvalent pneumococcal polysaccharide (PPV23) | 52 weeks |
| Jain 2017 | India | Case-control | RA | NI | Influenza (Trivalent) | 4 weeks |
| Jarrett 1980 | USA | Case-control | SLE | 15 | 14-valent pneumococcal conjugate (PCV14) | 52 weeks |
| Jeffs 2015 | Australia | Clinical trial | Vasculitis | NI | Influenza (Trivalent) | 4 weeks |
| Kivitz 2014 | USA | Clinical trial | RA | NI | Polyvalent pneumococcal polysaccharide (PPV23) and Influenza (Trivalent) | 4 weeks |
| Klippel 1980 | USA | Clinical trial | SLE | NI | Polyvalent pneumococcal polysaccharide (PPV23) | 4 weeks |
| Kogure 2014 | Japan | Cohort | RA | NI | Influenza (Trivalent) | 4 weeks |
| Kuruma 2007 | São Paulo, Brazil | Clinical trial | SLE | 2.86 ± 3.06 | Hepatitis B | 12 weeks |
| Lakota 2019 | Slovenia | Case-control | RA, PsA, AS, MCTD, Juvenile RA, and SD | NI | Influenza | 26 weeks |
| Lenfant 2021 | USA | Cohort | Autoimmune diseases | NI | Herpes Zoster | 12 weeks |
| Louie 1978 | USA | Case-control | SLE | NI | Influenza (Bivalent) | 4 weeks |
| Lu 2011 | Taiwan | Clinical trial | SLE | NI | Influenza (H1N1) | 26 weeks |
| Mathian 2011 | France | Cohort | SLE | NI | Influenza (H1N1) | 6 weeks |
| Mercado 2003 | Mexico | Cohort | SLE | 5-50mg/day (mean: 18.1mg/day) | Polyvalent pneumococcal polysaccharide (PPV23), Influenza (B), and Tetanus | 4 weeks |
| Mercado 2004 | Mexico | Clinical trial | SLE | median: 14.02 (IQR: 2.5-50.0) | Influenza (Trivalent) | 4 weeks |
| Mercado 2009 | Mexico | Clinical trial | Systemic sclerosis | 2.0-5.0 | Polyvalent pneumococcal polysaccharide (PPV23) | 4 weeks |
| Miossi 2013 | São Paulo, Brazil | Case-control | MCTD | 10.5 ± 7.2 | Influenza (H1N1) | 3 weeks |
| Mischlinger 2022 | Switzerland | Case-control | RA, SpA, PsA, Vasculitis | NI | Tetanus | NI |
| Mok 2012 | Hong-Kong | Case-control | SLE | 4.8 ± 2.0 | Quadrivalent human papillomavirus (HPV) | 52 weeks |
| Morgan 2016 | UK | Cohort | Vasculitis | median: 5; IQR: 5-10 | 7-valent pneumococcal conjugate (PCV7), Influenza (B), and Meningococcal group C | 104 weeks |
| Muravyeva 2021 | Russia | Cohort | SpA | NI | Polyvalent pneumococcal polysaccharide (PPV23) | 52 weeks |
| Nagel 2023 | Sweden | Case-control | RA | 41.9 ± 22.5 | 7-valent pneumococcal conjugate (PCV7) | 4 weeks |
| Nived 2017 | Sweden | Case-control | Vasculitis | median: 7.5; IQR: 0-65 | 13-valent pneumococcal conjugate (PCV13) | 6 weeks |
| Oren 2008 | Israel | Clinical trial | RA | 6.3 ± 2.2 | Influenza (Trivalent) | 4 weeks |
| Pasoto 2013 | São Paulo, Brazil | Case-control | SjS | 2.1 ± 4.9 | Influenza (H1N1) | 3 weeks |
| Pons 2022 | Spain | Cross-sectional | RA | 4.5 ± 3.2 | Herpes Zoster | 2 weeks |
| Rasmussen 2018 | Denmark | Cross-sectional | RA, SpA, PsA, and Juvenile idiopathic arthritis | NI | Polyvalent pneumococcal polysaccharide (PPV23) | 26 weeks |
| Rezende 2016 | Rio de Janeiro, Brazil | Cohort | SLE | median: 5; IQR: 2.1-20 | Polyvalent pneumococcal polysaccharide (PPV23) | 4 weeks |
| Ribeiro 2013 | São Paulo, Brazil | Case-control | RA | 7.5 (5-10) | Influenza (H1N1) | 3 weeks |
| Richi 2021b | Spain | Cohort | Autoimmune diseases | NI | Influenza (Trivalent) | 4 weeks |
| Richi 2021a | Spain | Cohort | RA, SpA, PsA, Undifferentiated arthritis, CTD, Pso, and IBD | NI | Pneumococcal (PCV13 and PPV23) | 4 weeks |
| Saad 2011 | São Paulo, Brazil | Cohort | SLE, RA, AS, Systemic sclerosis, PsA, MCTD, and SjS | 5.4 ± 9.3 | Influenza (H1N1) | 3 weeks |
| Shinjo 2012 | Brazil | Case-control | Dermatomyositis and Polymyositis | median: 9.7; IQR: 2.5-60 | Influenza (H1N1) | 3 weeks |
| Soybilgic 2013 | USA | Cohort | SLE | 12.6 (0-36) | Quadrivalent human papillomavirus (HPV) | 36 weeks |
| Stevens 2020 | USA | Cohort | RA, Giant-cell arteritis, SLE, PsA, SpA, and SjS | median: 5; IQR: 4-8 | Herpes Zoster | 12 weeks |
| Tarasova 2019 | Russia | Clinical trial | SLE | 5 to 30 | Polyvalent pneumococcal polysaccharide (PPV23) | 52 weeks |
| Tarasova 2021a | Russia | Clinical trial | SLE | 5 to 40 | Polyvalent pneumococcal polysaccharide (PPV23) | 8 weeks |
| Tarasova 2021b | Russia | Clinical trial | SLE | 5 to 30 | Polyvalent pneumococcal polysaccharide (PPV23) | 52 weeks |
| Tarasova 2022 | Russia | Clinical trial | Vasculitis | 2.5 to 60 | Polyvalent pneumococcal polysaccharide (PPV23) | 26 weeks |
| Tarján 2002 | Hungary | Case-control | SLE | 5.9 (0-20) | Polyvalent pneumococcal polysaccharide (PPV23) | 4 weeks |
| Wallin 2009 | Brazil | Case-control | SLE | 10.8 ±5.9 | Influenza (Trivalent) | 6 weeks |
| Więsik-Szewczyk 2010 | Poland | Clinical Trial | SLE | NI | Influenza (Trivalent) | 12 weeks |
| Williams 1978 | USA | Clinical Trial | SLE | NI | Influenza (Bivalent) | 20 weeks |
| Winthrop 2017 | USA and Puerto Rico | Clinical trial | RA | 6.2 ± 2.7 | Polyvalent pneumococcal polysaccharide (PPV23) and Tetanus | 5 weeks |

**Table 2.** Studies presenting the mean antibody levels at any time during the follow-up period.

| **First author and year** | **Study design** | **Immune-mediated rheumatic disease** | **Vaccine** | **Follow-up (weeks)** | **Subunit** | **Corticosteroids** | | | | **No corticosteroids** | | | | **Control** | | | |
| --- | --- | --- | --- | --- | --- | --- | --- | --- | --- | --- | --- | --- | --- | --- | --- | --- | --- |
|  |  |  |  |  |  | **Mean baseline** | **SD baseline** | **Mean follow-up** | **SD follow-up** | **Mean baseline** | **SD baseline** | **Mean follow-up** | **SD follow-up** | **Mean baseline** | **SD baseline** | **detecção de anticorpos de modo quantitativo (média do follow-up)** | **detecção de anticorpos de modo quantitativo (desvio-padrão do follow-up)** |
| Andrade 2012 | Case-control | Systemic sclerosis | Influenza (H1N1) | 3 | Total | 11.2 | NI | 134.4 | NI | NI | NI | NI | NI | 9.3 | NI | 122.9 | NI |
| Brodman 1978 | Clinical trial | SLE | Influenza (H1N1) | 4 | Total | 7.5 | 5 | 51 | 6 | NI | NI | NI | NI | 9.6 | 5 | 77 | 7 |
|  |  |  |  |  |  | 6.9 | 4 | 7.4 | 4 | NI | NI | NI | NI | 7.6 | 4 | 9.1 | 5 |
|  |  |  | Influenza (H1N1 and H3N2) |  |  | 7.5 | 5 | 80 | 5 | NI | NI | NI | NI | 9.6 | 5 | 102 | 5 |
|  |  |  |  |  |  | 6.9 | 4 | 18 | 6 | NI | NI | NI | NI | 7.6 | 4 | 17 | 6 |
| Caporuscio 2018 | Clinical trial | RA | Polyvalent pneumococcal polysaccharide (PPV13) | 4 | 1 | 1.7 | 3.1 | 30.3 | 51.2 | NI | NI | NI | NI | 6 | 2 | 28 | 19.6 |
|  |  |  |  |  | 3 | 58.7 | 56.2 | 82.9 | 46.9 | NI | NI | NI | NI | 24 | 18.5 | 75 | 45.9 |
|  |  |  |  |  | 4 | 16.7 | 12.68 | 36.4 | 31.5 | NI | NI | NI | NI | 13 | 10 | 53 | 35.7 |
|  |  |  |  |  | 5 | 10.6 | 7.4 | 110.7 | 83 | NI | NI | NI | NI | 18 | 16.8 | 74.2 | 53 |
|  |  |  |  |  | 6A | 24.4 | 21.9 | 56.6 | 52.3 | NI | NI | NI | NI | 24 | 18.8 | 50 | 27.4 |
|  |  |  |  |  | 6B | 20.1 | 19.31 | 59.7 | 78.6 | NI | NI | NI | NI | 38 | 19.8 | 249 | 77 |
|  |  |  |  |  | 7F | 5.6 | 4.8 | 58 | 48.3 | NI | NI | NI | NI | 27.6 | 27 | 49 | 49 |
|  |  |  |  |  | 9V | 15.2 | 13.6 | 45.2 | 42.5 | NI | NI | NI | NI | 16.4 | 16.6 | 75 | 56.5 |
|  |  |  |  |  | 14 | 21.6 | 2 | 73.2 | 49 | NI | NI | NI | NI | 11.54 | 11.4 | 60 | 37.7 |
|  |  |  |  |  | 18C | 13.3 | 7.25 | 45.3 | 38 | NI | NI | NI | NI | 17 | 11.6 | 54 | 34.2 |
|  |  |  |  |  | 19A | 12.05 | 15.2 | 32 | 27 | NI | NI | NI | NI | 17 | 13.4 | 93 | 72.7 |
|  |  |  |  |  | 19F | 6.9 | 9.2 | 22.8 | 20 | NI | NI | NI | NI | 13 | 15.1 | 32 | 30.3 |
|  |  |  |  |  | 23F | 6.9 | 6 | 32.3 | 36 | NI | NI | NI | NI | 6.59 | 4.6 | 44 | 29.4 |
| Del Porto 2006 | Clinical trial | SLE | Influenza (Trivalent) | 4 | H1N1 | 5 | NI | 67.5 | NI | NI | NI | NI | NI | 5.2 | NI | 145.8 | NI |
|  |  |  |  |  | H3N2 | 11.6 | NI | 230.7 | NI | NI | NI | NI | NI | 17.6 | NI | 152.7 | NI |
|  |  |  |  |  | B | 6.5 | NI | 26 | NI | NI | NI | NI | NI | 8.9 | NI | 54 | NI |
|  |  | RA |  |  | H1N1 | 7.5 | NI | 57.5 | NI | NI | NI | NI | NI | 5.2 | NI | 145.8 | NI |
|  |  |  |  |  | H3N2 | 21.8 | NI | 84.7 | NI | NI | NI | NI | NI | 17.6 | NI | 152.7 | NI |
|  |  |  |  |  | B | 5.6 | NI | 18.3 | NI | NI | NI | NI | NI | 8.9 | NI | 54 | NI |
| Elkayam 2011 | Clinical trial | RA | Influenza (H1N1) | 4 | H1N1 | 5.72 | NI | 64.29 | NI | NI | NI | NI | NI | 4.3 | NI | 127 | NI |
| Fernández-Prada 2022 | Cohort | RA, PsA, AS, Pso, IBD, SLE, Others | Hepatitis B | 26 | Total | NI | NI | 202.8 | NI | NI | NI | 295.2 | NI | NI | NI | NI | NI |
| Gabay 2011 | Cohort | RA, SpA, SLE, and ANCA associated necrotizing vasculitis | Influenza (H1N1) | 4 | Total | 13 | NI | 146 | NI | NI | NI | NI | NI | 9 | NI | 340 | NI |
| Holvast 2009a | Case-control | SLE | Influenza (Trivalent) | 4 | H1N1 | 39.5 | NI | 72.5 | NI | 32 | NI | 130.5 | NI | NI | NI | NI | NI |
|  |  |  |  |  | H3N2 | 20 | NI | 39 | NI | 39.2 | NI | 78.4 | NI | NI | NI | NI | NI |
|  |  |  |  |  | B | 22.9 | NI | 36.7 | NI | 18.4 | NI | 40.8 | NI | NI | NI | NI | NI |
|  |  |  |  | 8 | H1N1 | 39.5 | NI | 92.8 | NI | 32 | NI | 130.5 | NI | NI | NI | NI | NI |
|  |  |  |  |  | H3N2 | 20 | NI | 41 | NI | 39.2 | NI | 83.3 | NI | NI | NI | NI | NI |
|  |  |  |  |  | B | 22.9 | NI | 40 | NI | 18.4 | NI | 43.4 | NI | NI | NI | NI | NI |
| Jain 2017 | Case-control | RA | Influenza (Trivalent) | 4 | H1N1 | 53.94 | 78.3 | 212.85 | 254.65 | NI | NI | NI | NI | 80 | 136.75 | 367.58 | 226.53 |
|  |  |  |  |  | H3N2 | 94.17 | 120.97 | 212.85 | 201.24 | NI | NI | NI | NI | 91.89 | 81.01 | 296.28 | 246.88 |
|  |  |  |  |  | B | 67.69 | 141.95 | 287.03 | 234.1 | NI | NI | NI | NI | 16.62 | 29.65 | 94.77 | 195.91 |
| Jarrett 1980 | Case-control | SLE | Polyvalent pneumococcal polysaccharide (PPV14) | 4 | Total | 264.6 | NI | 900.8 | NI | NI | NI | NI | NI | 427.5 | NI | 1750.6 | NI |
|  |  |  |  | 52 |  | 264.6 | NI | 676.8 | NI | NI | NI | NI | NI | 427.5 | NI | 1328.2 | NI |
| Jeffs 2015 | Clinical trial | Vasculitis | Influenza (Trivalent) | 4 | Total | 10.4 | NI | 9.7 | NI | NI | NI | NI | NI | 12.4 | NI | 12.6 | NI |
| Klippel 1979 | Clinical trial | SLE | Polyvalent pneumococcal polysaccharide | 4 | Total | 177 | NI | 1045 | NI | NI | NI | NI | NI | 164 | NI | 153 | NI |
| Kogure 2014 | Cohort | RA | Influenza (Trivalent) | 4 | H1N1 | 33.5 | 64.1 | 99.7 | 236.3 | NI | NI | NI | NI | NI | NI | NI | NI |
|  |  |  |  |  | H3N2 | 62.7 | 176.4 | 220.4 | 315.9 | NI | NI | NI | NI | NI | NI | NI | NI |
|  |  |  |  |  | B | 14.2 | 10.5 | 36.4 | 87.3 | NI | NI | NI | NI | NI | NI | NI | NI |
| Lakota 2019 | Case-control | RA, PsA, AS, MCTD, Juvenile rheumatoid arthritis, and Still disease | Influenza | NI | Total | 45.8 | NI | 182.6 | NI | NI | NI | NI | NI | 64.2 | NI | 133.9 | NI |
| Mischlinger 2022 | Case-control | RA | Tetanus | NI | Total | NI | NI | 0.82 | NI | NI | NI | NI | NI | NI | NI | 1.66 | NI |
| Pasoto 2013 | Case-control | SjS | Influenza (H1N1) | 3 | Total | 8.9 | NI | 95.1 | NI | NI | NI | NI | NI | 8.1 | NI | 89.8 | NI |
| Ribeiro 2013 | Case-control | RA | Influenza (H1N1) | 3 | 7,5mg | 6 | NI | 10.7 | NI | NI | NI | NI | NI | 6.6 | NI | 76.1 | NI |
|  |  |  |  |  |  |  | NI | NI | NI | NI | NI | NI | NI | NI | NI | NI | NI |
|  |  |  |  |  | 10mg |  | NI | 52.6 | NI | NI | NI | NI | NI | 6.6 | NI | 76.1 | NI |
|  |  |  |  |  |  |  | NI | NI | NI | NI | NI | NI | NI | NI | NI | NI | NI |
| Tarján 2002 | Case-control | SLE | Polyvalent pneumococcal polysaccharide (PPV23) | 4 | Total | 12.9 | 2.88 | 12.22 | 2.85 | NI | NI | NI | NI | 12.36 | 3.4 | 11.63 | 2.63 |
| Wallin 2009 | Case-control | SLE | Influenza (Trivalent) | 6 | H1N1 | 159 | 239 | 900.2 | 1012 | NI | NI | NI | NI | 1059 | 1114 | 1888 | 1083 |
|  |  |  |  |  | H3N2 | 138.8 | 220 | 856 | 970 | NI | NI | NI | NI | 1305 | 1120 | 1994 | 1019 |
|  |  |  |  |  | B | 139 | 174.6 | 508.9 | 682.1 | NI | NI | NI | NI | 980 | 1409 | 1383 | 1059 |
| Więsik-Szewczyk 2010 | Clinical Trial | SLE | Influenza (Trivalent) | 4 | H1N1 | 6.27 | NI | 39.06 | NI | NI | NI | NI | NI | 6.33 | NI | 104.32 | NI |
|  |  |  |  |  | H3N2 | 6.5 | NI | 42.97 | NI | NI | NI | NI | NI | 6.42 | NI | 91.36 | NI |
|  |  |  |  |  | B | 7.24 | NI | 50.8 | NI | NI | NI | NI | NI | 6.82 | NI | 81.19 | NI |
|  |  |  |  | 12 | H1N1 | 6.27 | NI | 24.21 | NI | NI | NI | NI | NI | 6.33 | NI | 69.03 | NI |
|  |  |  |  |  | H3N2 | 6.5 | NI | 25.71 | NI | NI | NI | NI | NI | 6.42 | NI | 60.45 | NI |
|  |  |  |  |  | B | 7.24 | NI | 28.28 | NI | NI | NI | NI | NI | 6.82 | NI | 52.16 | NI |

**Table 3.** Studies presenting serological responses at any time during follow-up period.

| **First author and year** | **Immune-mediated rheumatic disease** | **Vaccine** | **Follow-up (weeks)** | **Subunit** | **Type of serological response** | **Corticosteroids serological response** | **No corticosteroids serological response** | **Control serological response** |
| --- | --- | --- | --- | --- | --- | --- | --- | --- |
| Abu-Shakra 2002 | SLE | Influenza (Trivalent) | 12 | H1N1 | Seroconversion | 9 (37.5%) | NI | NI |
|  |  |  |  | H3N2 |  | 14 (58.3%) | NI | NI |
|  |  |  |  | B |  | 15 (62.5%) | NI | NI |
|  |  |  |  | Total (at least 1) | Seroprotection | 18 (75.0%) | NI | NI |
|  |  |  |  | H1N1 | Immunogenicity | 9 (37.5%) | NI | NI |
|  |  |  |  | H3N2 |  | 16 (66.6%) | NI | NI |
|  |  |  |  | B |  | 22 (91.7%) | NI | NI |
|  |  |  | 6 | H1N1 | Seroprotection | 8 (33.3%) | NI | NI |
|  |  |  |  | H3N2 |  | 16 (66.7%) | NI | NI |
|  |  |  |  | B |  | 22 (91.6%) | NI | NI |
|  |  |  | 12 | H1N1 |  | 6 (25.0%) | NI | NI |
|  |  |  |  | H3N2 |  | 14 (58.3%) | NI | NI |
|  |  |  |  | B |  | 18 (75.0%) | NI | NI |
| Adler 2012 | RA | Influenza (H1N1) | 3 | Total | Seroconversion | 25 (53.0%) | NI | 34 (85.0%) |
|  |  |  | 6 |  |  | 20 (43.0%) | NI | 32 (80.0%) |
|  |  |  | 26 |  |  | 13 (28.0%) | NI | 26 (65.0%) |
|  | SpA |  | 3 |  |  | 44 (75.0%) | NI | 34 (85.0%) |
|  |  |  | 6 |  |  | 37 (63.0%) | NI | 32 (80.0%) |
|  |  |  | 26 |  |  | 25 (42.0%) | NI | 26 (65.0%) |
|  | Vasculitis |  | 3 |  |  | 7 (47.0%) | NI | 34 (85.0%) |
|  |  |  | 6 |  |  | 6 (40.0%) | NI | 32 (80.0%) |
|  |  |  | 26 |  |  | 3 (20.0%) | NI | 26 (65.0%) |
|  | CTD |  | 3 |  |  | 19 (68.0%) | NI | 34 (85.0%) |
|  |  |  | 6 |  |  | 17 (61.0%) | NI | 32 (80.0%) |
|  |  |  | 26 |  |  | 8 (29.0%) | NI | 26 (65.0%) |
|  | RA, SpA, Vasculitis, and CTD |  | 3 |  |  | 95 (64.0%) | NI | 34 (85.0%) |
|  |  |  | 6 |  |  | 80 (54.0%) | NI | 32 (80.0%) |
|  |  |  | 26 |  |  | 49 (33.0%) | NI | 26 (65.0%) |
| Andrade 2012 | Systemic sclerosis | Influenza (H1N1) | 3 | Total | Seroprotection | 103 (81.1%) | NI | 194 (82.9%) |
|  |  |  |  |  | Seroconversion | 92 (72.4%) | NI | 180 (76.9%) |
| Aydin 2020 | RA, AS, Pso, Reactive arthritis | Hepatitis B | 26 | Total | Immunogenicity | 72 (85.7%) | NI | NI |
|  | RA |  |  |  |  | 13 (68.4%) | NI | NI |
|  | AS |  |  |  |  | 39 (90.7%) | NI | NI |
|  | Pso |  |  |  |  | 19 (90.5%) | NI | NI |
|  | Reactive arthritis |  |  |  |  | 1 (100.0%) | NI | NI |
| Battafarano 1998 | SLE | Polyvalent pneumococcal polysaccharide (PPV23) | 12 | Total | Immunogenicity | NI | NI | NI |
|  |  | Influenza (B) |  |  |  | 64 (88.0%) | NI | NI |
|  |  | Tetanus |  |  |  | 65 (90.0%) | NI | NI |
| Borba 2012 | SLE | Influenza (H1N1) | 3 | Total | Seroprotection | 359 (64.7%) | NI | 143 (84.1%) |
|  |  |  |  |  | Seroconversion | 337 (60.7%) | NI | 136 (80.0%) |
|  |  |  |  |  | Seroprotection | 59 (59.6%) | 56 (74.7%) | 143 (84.1%) |
|  |  |  |  |  | Seroconversion | 56 (56.6%) | 54 (72.0%) | 136 (80.0%) |
| Broyde 2016 | RA, AS, PsA, IBD-associated SpA, and others | Polyvalent pneumococcal polysaccharide (PPV23) | 520 | Total | Seroprotection | 79 (89.8%) | NI | 29 (69.0%) |
| Caporuscio 2018 | RA | 13-valent pneumococcal conjugate (PCV13) | 4 | 1 | Immunogenicity | 19 (67.8%) | NI | 13 (65.0%) |
|  |  |  |  | 3 |  | 8 (34.8%) | NI | 7 (35.0%) |
|  |  |  |  | 4 |  | 21 (70.0%) | NI | 16 (80.0%) |
|  |  |  |  | 5 |  | 20 (87.0%) | NI | 8 (40.0%) |
|  |  |  |  | 6A |  | 10 (62.5%) | NI | 12 (60.0%) |
|  |  |  |  | 6B |  | 11 (68.8%) | NI | 12 (60.0%) |
|  |  |  |  | 7F |  | 13 (59.1%) | NI | 19 (95.0%) |
|  |  |  |  | 9V |  | 16 (69.6%) | NI | 15 (75.0%) |
|  |  |  |  | 14 |  | 11 (45.8%) | NI | 13 (65.0%) |
|  |  |  |  | 18C |  | 19 (82.6%) | NI | 18 (90.0%) |
|  |  |  |  | 19A |  | 4 (19.0%) | NI | 13 (65.0%) |
|  |  |  |  | 19F |  | 3 (13.0%) | NI | 13 (65.0%) |
|  |  |  |  | 23F |  | 14 (73.7%) | NI | 13 (65.0%) |
|  |  |  |  | 1 | Seroconversion | 26 (68.4%) | NI | 13 (65.0%) |
|  |  |  |  | 3 |  | 23 (60.5%) | NI | 7 (35.0%) |
|  |  |  |  | 4 |  | 32 (84.0%) | NI | 18 (90.0%) |
|  |  |  |  | 5 |  | 23 (60.5%) | NI | 8 (40.0%) |
|  |  |  |  | 6A |  | 16 (42.0%) | NI | 12 (60.0%) |
|  |  |  |  | 6B |  | 16 (42.0%) | NI | 12 (60.0%) |
|  |  |  |  | 7F |  | 22 (57.9%) | NI | 19 (95.0%) |
|  |  |  |  | 9V |  | 26 (68.4%) | NI | 15 (75.0%) |
|  |  |  |  | 14 |  | 24 (63.1%) | NI | 13 (65.0%) |
|  |  |  |  | 18C |  | 23 (60.5%) | NI | 18 (90.0%) |
|  |  |  |  | 19A |  | 21 (55.3%) | NI | 13 (65.0%) |
|  |  |  |  | 19F |  | 23 (60.5%) | NI | 13 (65.0%) |
|  |  |  |  | 23F |  | 19 (50.0%) | NI | 13 (65.0%) |
| Del Porto 2006 | SLE | Influenza (Trivalent) | 4 | H1N1 | Seroconversion | 8 (57.0%) | NI | 7 (70.0%) |
|  |  |  |  | H3N2 |  | 13 (93.0%) | NI | 5 (50.0%) |
|  |  |  |  | B |  | 5 (36.0%) | NI | 5 (50.0%) |
|  |  |  |  | H1N1 | Seroprotection | 8 (57.0%) | NI | 7 (70.0%) |
|  |  |  |  | H3N2 |  | 14 (100.0%) | NI | 8 (80.0%) |
|  |  |  |  | B |  | 5 (36.0%) | NI | 6 (60.0%) |
|  | RA |  |  | H1N1 | Seroconversion | 4 (40.0%) | NI | 7 (70.0%) |
|  |  |  |  | H3N2 |  | 5 (50.0%) | NI | 5 (50.0%) |
|  |  |  |  | B |  | 2 (20.0%) | NI | 5 (50.0%) |
|  |  |  |  | H1N1 | Seroprotection | 6 (60.0%) | NI | 7 (70.0%) |
|  |  |  |  | H3N2 |  | 7 (70.0%) | NI | 8 (80.0%) |
|  |  |  |  | B |  | 2 (20.0%) | NI | 6 (60.0%) |
| Dhar 2017 | SLE | Quadrivalent human papillomavirus (HPV) | 4 | 6 | Seroconversion | 16 (100.0%) | NI | NI |
|  |  |  |  | 11 |  | 27 (100.0%) | NI | NI |
|  |  |  |  | 16 |  | 19 (100.0%) | NI | NI |
|  |  |  |  | 18 |  | 27 (100.0%) | NI | NI |
| Elkayam 2002 | RA | Polyvalent pneumococcal polysaccharide (PPV23) | 9 | 14 | Seroconversion | 19 (45.9%) | NI | 13 (64.7%) |
|  |  |  |  | 23F |  | 25 (60.5%) | NI | 11 (55.0%) |
|  |  |  |  | 4 |  | 14 (34.2%) | NI | 11 (55.0%) |
|  |  |  |  | 8 |  | 28 (65.8%) | NI | 15 (75.0%) |
|  |  |  |  | 9N |  | 23 (55.3%) | NI | 17 (85.0%) |
|  |  |  |  | 7F |  | 28 (65.8%) | NI | 19 (95.0%) |
|  |  |  |  | 2 |  | 23 (55.3%) | NI | 17 (85.0%) |
|  | SLE |  |  | 14 |  | 15 (61.9%) | NI | 13 (64.7%) |
|  |  |  |  | 23F |  | 15 (63.6%) | NI | 11 (55.0%) |
|  |  |  |  | 4 |  | 9 (36.4%) | NI | 11 (55.0%) |
|  |  |  |  | 8 |  | 11 (45.5%) | NI | 15 (75.0%) |
|  |  |  |  | 9N |  | 17 (71.4%) | NI | 17 (85.0%) |
|  |  |  |  | 7F |  | 19 (81.0%) | NI | 19 (95.0%) |
|  |  |  |  | 2 |  | 13 (54.5%) | NI | 17 (85.0%) |
| Elkayam 2011 | RA | Influenza (H1N1) | 4 | H1N1 | Immunogenicity | 23 (56.0%) | NI | 21 (84.0%) |
|  | SLE |  |  |  |  | 10 (59.0%) | NI | NI |
|  | PsA |  |  |  |  | 8 (53.0%) | NI | NI |
|  | RA |  |  |  | Seroprotection | 29 (71.0%) | NI | 23 (92.0%) |
|  | SLE |  |  |  |  | 13 (76.0%) | NI | NI |
|  | PsA |  |  |  |  | 9 (60.0%) | NI | NI |
| Evison 2009 | Chronic idiopathic arthritis, SpA, Autoimmune connectivitis, Sarcoidosis, Vasculitis | Influenza (Trivalent) | 6 | Total | Seroprotection | 10 (35.7%) | NI | 3 (15.8%) |
|  |  |  |  |  | Seroconversion | 8 (28.6%) | NI | 1 (5.3%) |
| Fernández-Prada 2022 | RA, PsA, AS, Pso, IBD, SLE, and others | Hepatitis B | 26 | Total | Seroconversion | 247 (82.1%) | 163 (87.6%) | NI |
| Fischer 2016 | RA, SpA, Pso, PsA, CTD, Systemic vasculitis, and others | Polyvalent pneumococcal polysaccharide (PPV23) | 52 | Total | Soropositividade | 33 (67.3%) | NI | 122 (90.4%) |
|  |  |  | 8 |  |  | 46 (87.0%) | NI | NI |
| Fomin 2006 | RA | Influenza (Trivalent) | 6 | B | Immunogenicity | 51 (67.0%) | NI | 26 (87.0%) |
|  |  |  |  | H3N2 |  | 43 (53.0%) | NI | 16 (54.0%) |
|  |  |  |  | H1N1 |  | 43 (53.0%) | NI | 20 (68.0%) |
| Garrido 2022 | CD, UC, RA, Pso/PsA, Spondylitis ankylopoietica, Neurological autoimmune disease, other autoinflammatory disease, Solid organ transplant recipient | Pneumococcal (PCV13 and PPV23) | 8 | PPV13 | Seroprotection | 26 (53.0%) | 25 (57.0%) | 24 (75.0%) |
|  |  |  |  | PPV23 |  | 1 (2.0%) | 0 (0%) | 3 (9.4%) |
|  |  |  |  | Total |  | 6 (12.0%) | 5 (11.0%) | 10 (31.0%) |
|  |  |  | 16 | PPV13 |  | 24 (52.0%) | 24 (53.0%) | 29 (85.0%) |
|  |  |  |  | PPV23 |  | 31 (67.0%) | 29 (64.0%) | 28 (82.0%) |
|  |  |  |  | Total |  | 26 (57.0%) | 26 (58.0%) | 28 (82.0%) |
|  |  |  | 26 | PPV13 |  | 19 (49.0%) | 21 (54.0%) | 24 (75.0%) |
|  |  |  |  | PPV23 |  | 24 (62.0%) | 21 (54.0%) | 27 (84.0%) |
|  |  |  |  | Total |  | 19 (49.0%) | 21 (54.0%) | 24 (75.0%) |
|  |  |  | 52 | PPV13 |  | 16 (35.0%) | 18 (41.0%) | 20 (57.0%) |
|  |  |  |  | PPV23 |  | 22 (48.0%) | 23 (52.0%) | 26 (74.0%) |
|  |  |  |  | Total |  | 18 (39.0%) | 21 (48.0%) | 22 (63.0%) |
| Gerard 2020 | SLE | Pneumococcal (PCV13 and PPV23) | 144 | Total | Seroprotection | 10 (47.6%) | NI | NI |
| Grabar 2017 | SLE | Pneumococcal (PCV7 and PPV23) | 4 | Total | Immunogenicity | 2 (11.8%) | NI | 0 (0%) |
|  |  |  | 24 |  |  | 2 (11.8%) | NI | 0 (0%) |
|  |  |  | 28 |  |  | 7 (41.2%) | NI | 6 (24.0%) |
|  |  |  | 52 |  |  | 4 (23.5%) | NI | 4 (16.0%) |
|  |  |  | 4 | 4 | Seroprotection | 12 (70.6%) | NI | 7 (28.0%) |
|  |  |  | 24 | 6B |  | 16 (94.1%) | NI | 17 (68.0%) |
|  |  |  | 28 | 9V |  | 16 (94.1%) | NI | 12 (48.0%) |
|  |  |  | 52 | 14 |  | 16 (94.1%) | NI | 19 (76.0%) |
|  |  |  | 4 | 18C |  | 13 (76.5%) | NI | 11 (44.0%) |
|  |  |  | 24 | 19F |  | 16 (94.1%) | NI | 20 (80.0%) |
|  |  |  | 28 | 23F |  | 13 (76.5%) | NI | 13 (52.0%) |
|  |  |  | 52 | 4 |  | 12 (70.6%) | NI | 7 (28.0%) |
|  |  |  | 4 | 6B |  | 15 (88.2%) | NI | 15 (60.0%) |
|  |  |  | 24 | 9V |  | 16 (94.1%) | NI | 12 (48.0%) |
|  |  |  | 28 | 14 |  | 15 (88.2%) | NI | 19 (76.0%) |
|  |  |  | 52 | 18C |  | 13 (76.5%) | NI | 12 (48.0%) |
|  |  |  | 4 | 19F |  | 15 (88.2%) | NI | 18 (72.0%) |
|  |  |  | 24 | 23F |  | 13 (76.5%) | NI | 12 (48.0%) |
|  |  |  | 28 | 4 |  | 14 (82.4%) | NI | 19 (76.0%) |
|  |  |  | 52 | 6B |  | 15 (88.2%) | NI | 22 (88.0%) |
|  |  |  | 4 | 9V |  | 16 (94.1%) | NI | 22 (88.0%) |
|  |  |  | 24 | 14 |  | 16 (94.1%) | NI | 23 (92.0%) |
|  |  |  | 28 | 18C |  | 15 (88.2%) | NI | 21 (84.0%) |
|  |  |  | 52 | 19F |  | 16 (94.1%) | NI | 23 (92.0%) |
|  |  |  | 4 | 23F |  | 14 (82.4%) | NI | 19 (76.0%) |
|  |  |  | 24 | 4 |  | 11 (64.7%) | NI | 16 (64.0%) |
|  |  |  | 28 | 6B |  | 13 (76.5%) | NI | 21 (84.0%) |
|  |  |  | 52 | 9V |  | 13 (76.5%) | NI | 20 (80.0%) |
|  |  |  | 4 | 14 |  | 13 (76.5%) | NI | 21 (84.0%) |
|  |  |  | 24 | 18C |  | 12 (70.6%) | NI | 18 (72.0%) |
|  |  |  | 28 | 19F |  | 13 (76.5%) | NI | 22 (88.0%) |
|  |  |  | 52 | 23F |  | 11 (64.7%) | NI | 17 (68.0%) |
| Herron 1979 | RA | Influenza (H3N2) | 16 | H3N2 | Seroconversion | 10 (65.0%) | NI | 21 (69.0%) |
|  | SLE |  |  |  |  | 12 (67.0%) | NI | NI |
|  | Other |  |  |  |  | 8 (62.0%) | NI | NI |
| Holvast 2005 | SLE | Influenza (Trivalent) | 3 | H1N1 | Seroconversion | 24 (42.9%) | 18 (42.9%) | 16 (88.9%) |
|  |  |  |  | H3N2 |  | 22 (39.3%) | 16 (38.1%) | 15 (83.3%) |
|  |  |  |  | B |  | 23 (41.1%) | 18 (42.9%) | 12 (66.7%) |
| Holvast 2009b | SLE | Influenza (Trivalent) | 4 | H1N1 | Seroconversion | 24 (42.1%) | NI | 42 (77.8%) |
|  |  |  |  | H3N2 |  | 37 (68.5%) | NI | 41 (75.9%) |
|  |  |  |  | H1N1 | Seroprotection | 44 (81.5%) | NI | 48 (88.9%) |
|  |  |  |  | H3N2 |  | 41 (75.9%) | NI | 50 (92.6%) |
|  |  |  | 16 | H1N1 |  | 36 (66.7%) | NI | 39 (72.2%) |
|  |  |  |  | H3N2 |  | 37 (68.5%) | NI | 45 (83.3%) |
| Holvast 2009a | SLE | Influenza (Trivalent) | 4 | Total | Seroprotection | 27 (51.9%) | NI | 8 (28.6%) |
|  |  |  |  | H1N1 |  | 45 (86.5%) | NI | 24 (85.7%) |
|  |  |  |  | H3N2 |  | 42 (80.8%) | NI | 23 (82.1%) |
|  |  |  |  | B |  | 32 (61.5%) | NI | 12 (42.9%) |
|  |  |  |  | H1N1 | Seroconversion | 18 (34.6%) | NI | 8 (28.6%) |
|  |  |  |  | H3N2 |  | 13 (25.0%) | NI | 3 (10.7%) |
|  |  |  |  | B |  | 10 (19.2%) | NI | 3 (10.7%) |
|  |  |  | 8 | Total | Seroprotection | 29 (55.8%) | NI | NI |
|  |  |  |  | H1N1 |  | 48 (92.3%) | NI | NI |
|  |  |  |  | H3N2 |  | 42 (80.8%) | NI | NI |
|  |  |  |  | B |  | 33 (63.5%) | NI | NI |
|  |  |  |  | H1N1 | Seroconversion | 23 (44.2%) | NI | NI |
|  |  |  |  | H3N2 |  | 13 (25.0%) | NI | NI |
|  |  |  |  | B |  | 10 (19.2%) | NI | NI |
|  |  |  | 4 | H1N1 | Seroprotection | 23 (82.1%) | 16 (94.1%) | NI |
|  |  |  |  | H3N2 |  | 19 (67.9%) | 16 (94.1%) | NI |
|  |  |  |  | B |  | 17 (60.7%) | 11 (64.7%) | NI |
|  |  |  |  | H1N1 | Seroconversion | 4 (14.3%) | 11 (64.7%) | NI |
|  |  |  |  | H3N2 |  | 5 (17.9%) | 5 (29.4%) | NI |
|  |  |  |  | B |  | 3 (10.7%) | 5 (29.4%) | NI |
|  |  |  | 8 | H1N1 | Seroprotection | 25 (89.3%) | 16 (94.1%) | NI |
|  |  |  |  | H3N2 |  | 19 (67.9%) | 16 (94.1%) | NI |
|  |  |  |  | B |  | 17 (60.7%) | 10 (58.8%) | NI |
|  |  |  |  | H1N1 | Seroconversion | 3 (10.7%) | 1 (5.9%) | NI |
|  |  |  |  | H3N2 |  | 0 (0%) | 0 (0%) | NI |
|  |  |  |  | B |  | 0 (0%) | 0 (0%) | NI |
| Intongkam 2018 | RA | Hepatitis B | 32 | Total | Immunogenicity | 14 (63.6%) | 15 (65.2%) | NI |
| Jain 2017 | RA | Influenza (Trivalent) | 4 | H1N1 | Seroprotection | 50 (98.0%) | NI | 44 (99.0%) |
|  |  |  |  | H3N2 |  | 50 (98.0%) | NI | 43 (96.0%) |
|  |  |  |  | B |  | 50 (98.0%) | NI | 38 (85.0%) |
|  |  |  |  | H1N1 | Immunogenicity | 29 (57.0%) | NI | 29 (64.0%) |
|  |  |  |  | H3N2 |  | 19 (38.0%) | NI | 26 (58.0%) |
|  |  |  |  | B |  | 29 (57.0%) | NI | 26 (58.0%) |
|  |  |  |  | H1N1 | Seroprotection | 50 (98.0%) | NI | 44 (99.0%) |
|  |  |  |  | H3N2 |  | 48 (94.0%) | NI | 43 (96.0%) |
|  |  |  |  | B |  | 48 (94.0%) | NI | 38 (85.0%) |
|  |  |  |  | H1N1 | Immunogenicity | 36 (70.0%) | NI | 29 (64.0%) |
|  |  |  |  | H3N2 |  | 23 (45.0%) | NI | 26 (58.0%) |
|  |  |  |  | B |  | 18 (36.0%) | NI | 26 (58.0%) |
| Jarrett 1980 | SLE | 14-valent pneumococcal conjugate (PCV14) | 4 | Total | Immunogenicity | 28 (73.7%) | NI | 14 (82.4%) |
| Jeffs 2015 | Vasculitis | Influenza (Trivalent) | 4 | H1N1 | Seroconversion | 13 (54.2%) | NI | 34 (64.2%) |
|  |  |  |  | H3N2 |  | 12 (50.0%) | NI | 43 (81.1%) |
|  |  |  |  | B |  | 8 (33.3%) | NI | 24 (45.3%) |
|  |  |  |  | H1N1 | Seroprotection | 17 (70.8%) | NI | 48 (90.6%) |
|  |  |  |  | H3N2 |  | 17 (70.8%) | NI | 51 (96.2%) |
|  |  |  |  | B |  | 10 (41.7%) | NI | 30 (56.6%) |
| Kivitz 2014 | RA | Polyvalent pneumococcal polysaccharide (PPV23) | 4 | Total | Immunogenicity | 23 (60.5%) | 25 (50.0%) | 22 (66.7%) |
|  |  | Influenza (Trivalent) |  |  |  | 18 (47.4%) | 28 (58.3%) | 17 (58.6%) |
|  |  | Polyvalent pneumococcal polysaccharide (PPV23) |  |  |  | 23 (60.5%) | 25 (50.0%) | 33 (60.0%) |
|  |  | Influenza (Trivalent) |  |  |  | 18 (47.4%) | 28 (58.3%) | 34 (63.0%) |
| Kogure 2014 | RA | Influenza (Trivalent) | 4 | H1N1 | Seroprotection | 36 (63.2%) | NI | NI |
|  |  |  |  | H3N2 |  | 46 (80.7%) | NI | NI |
|  |  |  |  | B |  | 15 (26.3%) | NI | NI |
| Kuruma 2007 | SLE | Hepatitis B | 4 | Total | Seroconversion | 1 (4.0%) | NI | NI |
|  |  |  | 9 |  |  | 15 (54.0%) | NI | NI |
|  |  |  | 12 |  |  | 26 (93%) | NI | NI |
| Lakota 2019 | RA, PsA, AS, MCTD, Juvenile RA, and SD | Influenza | 26 | H1N1 | Seroprotection | 122 (89.0%) | NI | NI |
| Louie 1978 | SLE | Influenza (H1N1 and H3N2) | 4 | H1N1 | Seroconversion | 8 (80.0%) | NI | 7 (100.0%) |
|  |  |  |  | H3N2 |  | 7 (63.6%) | NI | 5 (62.5%) |
| Lu 2011 | SLE | Influenza (H1N1) | 26 | Total | Seroconversion | 11 (52.4%) | NI | 8 (53.3%) |
|  |  |  | 3 |  |  | 16 (76.2%) | NI | 12 (80.0%) |
|  |  |  | 26 |  | Seroprotection | 14 (66.7%) | NI | 9 (60.0%) |
|  |  |  | 3 |  |  | 16 (76.2%) | NI | 12 (80.0%) |
| Mathian 2011 | SLE | Influenza (H1N1) | 3 | Total | Seroprotection | 74 (66.7%) | NI | NI |
|  |  |  | 6 |  |  | 88 (80.0%) | NI | NI |
|  |  |  | 3 |  | Seroconversion | 67 (60.4%) | NI | NI |
|  |  |  | 6 |  |  | 79 (71.8%) | NI | NI |
|  |  |  | 3 |  | Seroprotection | 18 (47.4%) | NI | NI |
|  |  |  | 6 |  |  | 27 (73.0%) | NI | NI |
|  |  |  | 3 |  | Seroconversion | 16 (42.1%) | NI | NI |
|  |  |  | 6 |  |  | 25 (67.6%) | NI | NI |
| Mercado 2003 | SLE | Polyvalent pneumococcal polysaccharide (PPV23) | 4 | Total | Immunogenicity | 6 (54.5%) | NI | NI |
|  |  | Influenza (B) |  |  |  | 5 (41.7%) | NI | NI |
|  |  | Tetanus |  |  |  | 3 (25.0%) | NI | NI |
| Mercado 2004 | SLE | Influenza (Trivalent) | 4 | H1N1 | Seroprotection | 13 (72.0%) | NI | 14 (77.0%) |
|  |  |  |  | H3N2 |  | 12 (67.0%) | NI | 17 (94.0%) |
|  |  |  |  | B |  | 11 (61.0%) | NI | 17 (94.0%) |
| Mercado 2009 | Systemic sclerosis | Polyvalent pneumococcal polysaccharide (PPV23) | 4 | 4 | Seroprotection | 14 (77.8%) | NI | NI |
|  |  |  |  | 6B |  | 15 (83.3%) | NI | NI |
|  |  |  |  | 12F |  | 13 (72.2%) | NI | NI |
|  |  |  |  | 14 |  | 16 (88.9%) | NI | NI |
|  |  |  |  | At least one |  | 18 (100.0%) | NI | NI |
|  |  |  |  | At least three |  | 15 (83.3%) | NI | NI |
| Miossi 2013 | MCTD | Influenza (H1N1) | 3 | Total | Seroprotection | 52 (75.4%) | NI | 49 (71.0%) |
|  |  |  |  |  | Seroconversion | 47 (68.1%) | NI | 45 (65.2%) |
| Mischlinger 2022 | RA | Tetanus | Short-term | Total | Seroprotection | 72 (84.7%) | NI | 164 (97.0%) |
|  | SpA |  |  |  |  | 43 (97.7%) | NI | NI |
|  | Vasculitis |  |  |  |  | 14 (93.3%) | NI | NI |
|  | PsA |  |  |  |  | 19 (100.0%) | NI | NI |
|  | RA |  | Long-term |  |  | 47 (55.3%) | NI | 122 (72.2%) |
|  | SpA |  |  |  |  | 30 (68.2%) | NI | NI |
|  | Vasculitis |  |  |  |  | 11 (73.3%) | NI | NI |
|  | PsA |  |  |  |  | 15 (79.0%) | NI | NI |
|  | RA | Diphtheria | Short-term |  |  | 32 (37.7%) | NI | 110 (65.5%) |
|  | SpA |  |  |  |  | 30 (68.2%) | NI | NI |
|  | Vasculitis |  |  |  |  | 6 (40.0%) | NI | NI |
|  | PsA |  |  |  |  | 13 (68.4%) | NI | NI |
|  | RA |  | Long-term |  |  | 3 (3.5%) | NI | 8 (4.8%) |
|  | SpA |  |  |  |  | 4 (9.1%) | NI | NI |
|  | Vasculitis |  |  |  |  | 0 (0%) | NI | NI |
|  | PsA |  |  |  |  | 1 (5.3%) | NI | NI |
|  | RA, SpA, PsA, Vasculitis | Tetanus | Short-term |  |  | 6 (75.0%) | NI | 164 (97.0%) |
|  |  |  | Long-term |  |  | 4 (50.0%) | NI | 122 (72.2%) |
|  |  | Diphtheria | Short-term |  |  | 3 (37.5%) | NI | 110 (65.5%) |
|  |  |  | Long-term |  |  | 0 (0.0%) | NI | 8 (4.8%) |
| Mok 2012 | SLE | Quadrivalent human papillomavirus (HPV) | 30 | 6 | Seroconversion | 29 (74.0%) | NI | 43 (96.0%) |
|  |  |  |  | 11 |  | 29 (76.0%) | NI | 42 (95.0%) |
|  |  |  |  | 16 |  | 36 (92.0%) | NI | 43 (98.0%) |
|  |  |  |  | 18 |  | 29 (76.0%) | NI | 37 (93.0%) |
|  |  |  | 52 | 6 |  | 32 (82.0%) | NI | 44 (98.0%) |
|  |  |  |  | 11 |  | 34 (89.0%) | NI | 43 (98.0%) |
|  |  |  |  | 16 |  | 37 (95.0%) | NI | 43 (98.0%) |
|  |  |  |  | 18 |  | 29 (76.0%) | NI | 32 (80.0%) |
| Morgan 2016 | Systemic Vasculitis | 7-valent pneumococcal conjugate (PCV7) | 4 | 4 | Immunogenicity | 42 (45.7%) | NI | NI |
|  |  |  |  | 6B |  | 67 (72.8%) | NI | NI |
|  |  |  |  | 9V |  | 82 (89.1%) | NI | NI |
|  |  |  |  | 14 |  | 74 (80.4%) | NI | NI |
|  |  |  |  | 18C |  | 88 (95.7%) | NI | NI |
|  |  |  |  | 19F |  | 77 (83.7%) | NI | NI |
|  |  |  |  | 23F |  | 74 (80.4%) | NI | NI |
|  |  | Influenza (B) |  | B |  | 68 (73.9%) | NI | NI |
|  |  | Meningococcal group C |  | MenA |  | 79 (85.9%) | NI | NI |
|  |  |  |  | MenC |  | 54 (58.7%) | NI | NI |
|  |  |  |  | MenW135 |  | 23 (25.0%) | NI | NI |
|  |  |  |  | MenY |  | 49 (53.3%) | NI | NI |
| Nived 2017 | Systemic Vasculitis | 13-valent pneumococcal conjugate (PCV13) | 6 | Total | Seroprotection | 24 (49.0%) | NI | NI |
|  |  |  |  | 6B |  | 32 (65.0%) | NI | NI |
|  |  |  |  | 23F |  | 32 (65.0%) | NI | NI |
| Oren 2008 | RA | Influenza (Trivalent) | 4 | H1N1 | Immunogenicity | 11 (37.0%) | NI | 9 (45.0%) |
|  |  |  |  | H3N2 |  | 19 (67.0%) | NI | 8 (40.0%) |
|  |  |  |  | B |  | 9 (30.0%) | NI | 8 (40.0%) |
|  |  |  |  | H1N1 |  | 5 (36.0%) | NI | NI |
|  |  |  |  | H3N2 |  | 4 (26.0%) | NI | NI |
|  |  |  |  | B |  | 3 (21.0%) | NI | NI |
| Pasoto 2013 | SjS | Influenza (H1N1) | 3 | Total | Seroprotection | 30 (83.3%) | NI | 26 (72.2%) |
|  |  |  |  |  | Seroconversion | 28 (77.8%) | NI | 25 (69.4%) |
| Rasmussen 2018 | RA, SpA, PsA, and Juvenile idiopathic arthritis | Polyvalent pneumococcal polysaccharide (PPV23) | 26 | Total | Seroprotection | 56 (30.0%) | NI | 0 (0%) |
| Rezende 2016 | SLE | Polyvalent pneumococcal polysaccharide (PPV23) | 4 | 4 | Seroconversion | 26 (54.2%) | NI | NI |
|  |  |  |  | 6B |  | 21 (48.8%) | NI | NI |
|  |  |  |  | 9V |  | 22 (50.0%) | NI | NI |
|  |  |  |  | 14 |  | 18 (78.3%) | NI | NI |
|  |  |  |  | 18C |  | 24 (72.7%) | NI | NI |
|  |  |  |  | 19F |  | 13 (52.0%) | NI | NI |
|  |  |  |  | 23F |  | 22 (51.2%) | NI | NI |
|  |  |  |  | 4 | Immunogenicity | 24 (44.4%) | NI | NI |
|  |  |  |  | 6B |  | 21 (38.9%) | NI | NI |
|  |  |  |  | 9V |  | 26 (48.1%) | NI | NI |
|  |  |  |  | 14 |  | 31 (57.4%) | NI | NI |
|  |  |  |  | 18C |  | 35 (64.8%) | NI | NI |
|  |  |  |  | 19F |  | 17 (31.5%) | NI | NI |
|  |  |  |  | 23F |  | 27 (50.0%) | NI | NI |
|  |  |  |  | Total | Seroconversion | 21 (38.9%) | NI | NI |
|  |  |  |  | Total | Immunogenicity | 16 (29.6%) | NI | NI |
| Ribeiro 2013 | RA | Influenza (H1N1) | 3 | Total | Seroconversion | 0 (0%) | NI | 36 (66.0%) |
|  |  |  |  |  | Seroprotection | 1 (9.0%) | NI | 36 (66.0%) |
|  |  |  |  |  | Seroconversion | 19 (58.0%) | NI | 36 (66.0%) |
|  |  |  |  |  | Seroprotection | 19 (58.0%) | NI | 36 (66.0%) |
| Richi 2021a | Autoimmune diseases | Influenza (Trivalent) | 4 | A | Soropositividade | 93 (81.6%) | NI | 43 (89.6%) |
|  |  |  |  | B |  | 48 (42.1%) | NI | 26 (54.2%) |
| Richi 2021b | RA, SpA, PsA, Undifferentiated arthritis, CTD, Pso, and IBD | Pneumococcal (PCV13 and PPV23) | 4 | 1 | Seroconversion | 80 (44.0%) | NI | NI |
|  |  |  |  | 3 |  | 86 (47.3%) | NI | NI |
|  |  |  |  | 7F |  | 65 (35.7%) | NI | NI |
|  |  |  |  | 14 |  | 81 (44.5%) | NI | NI |
|  |  |  |  | 19A |  | 65 (35.7%) | NI | NI |
|  |  |  |  | 19F |  | 60 (33.0%) | NI | NI |
| Saad 2011 | SLE | Influenza (H1N1) | 3 | Total | Seroprotection | 392 (64.9%) | NI | 194 (82.9%) |
|  | RA |  |  |  |  | 223 (60.1%) | NI | NI |
|  | AS |  |  |  |  | 112 (73.7%) | NI | NI |
|  | Systemic sclerosis |  |  |  |  | 103 (81.1%) | NI | NI |
|  | PsA |  |  |  |  | 66 (65.3%) | NI | NI |
|  | MCTD |  |  |  |  | 52 (75.4%) | NI | NI |
|  | SjS |  |  |  |  | 30 (83.3%) | NI | NI |
|  | SLE |  |  |  | Seroconversion | 346 (60.5%) | NI | 180 (76.9%) |
|  | RA |  |  |  |  | 183 (53.4%) | NI | NI |
|  | AS |  |  |  |  | 106 (69.8%) | NI | NI |
|  | Systemic sclerosis |  |  |  |  | 93 (73.2%) | NI | NI |
|  | PsA |  |  |  |  | 58 (57.4%) | NI | NI |
|  | MCTD |  |  |  |  | 47 (68.1%) | NI | NI |
|  | SjS |  |  |  |  | 28 (77.8%) | NI | NI |
| Shinjo 2012 | Dermatomyositis and Polymyositis | Influenza (H1N1) | 3 | Total | Seroconversion | 42 (72.4%) | NI | 91 (78.4%) |
|  |  |  |  |  | Seroprotection | 42 (72.4%) | NI | 98 (84.5%) |
| Soybilgic 2013 | SLE | Quadrivalent human papillomavirus (HPV) | 36 | 6 | Seroprotection | 15 (93.8%) | NI | NI |
|  |  |  |  | 11 |  | 16 (100.0%) | NI | NI |
|  |  |  |  | 16 |  | 16 (100.0%) | NI | NI |
|  |  |  |  | 18 |  | 15 (93.8%) | NI | NI |
| Tarasova 2019 | SLE | Polyvalent pneumococcal polysaccharide (PPV23) | 4 | Total | Immunogenicity | 28 (82.4%) | NI | NI |
|  |  |  | 12 |  |  | 33 (97.0%) | NI | NI |
|  |  |  | 52 |  |  | 22 (64.7%) | NI | NI |
| Tarasova 2021 | SLE | Polyvalent pneumococcal polysaccharide (PPV23) | 8 | Total | Seroprotection | 47 (78.7%) | NI | NI |
| Wallin 2009 | SLE | Influenza (Trivalent) | 6 | H3N2 | Seroconversion | 29 (61.7%) | NI | 22 (81.4%) |
|  |  |  |  | H1N1 |  | 27 (57.4%) | NI | 22 (81.4%) |
|  |  |  |  | B |  | 24 (51.1%) | NI | 17 (62.9%) |
|  |  |  |  | H3N2 | Seroprotection | 32 (68.0%) | NI | 23 (85.2%) |
|  |  |  |  | H1N1 |  | 36 (76.6%) | NI | 23 (85.2%) |
|  |  |  |  | B |  | 31 (65.9%) | NI | 23 (85.2%) |
| Więsik-Szewczyk 2010 | SLE | Influenza (Trivalent) | 4 | H1N1 | Seroconversion | 29 (53.0%) | NI | 39 (83.0%) |
|  |  |  |  | H3N2 |  | 30 (55.0%) | NI | 40 (85.0%) |
|  |  |  |  | B |  | 30 (56.0%) | NI | 34 (72.0%) |
|  |  |  | 12 | H1N1 |  | 17 (32.0%) | NI | 37 (79.0%) |
|  |  |  |  | H3N2 |  | 22 (40.0%) | NI | 33 (70.0%) |
|  |  |  |  | B |  | 20 (37.0%) | NI | 31 (64.0%) |
|  |  |  | 4 | H1N1 | Seroprotection | 33 (62.0%) | NI | 46 (98.0%) |
|  |  |  |  | H3N2 |  | 36 (67.0%) | NI | 44 (94.0%) |
|  |  |  |  | B |  | 39 (73.0%) | NI | 42 (90.0%) |
|  |  |  | 12 | H1N1 |  | 23 (43.0%) | NI | 44 (94.0%) |
|  |  |  |  | H3N2 |  | 28 (52.0%) | NI | 43 (91.0%) |
|  |  |  |  | B |  | 29 (53.0%) | NI | 37 (79.0%) |
| Williams 1978 | SLE | Influenza (Bivalent) | 20 | H1N1 | Seroconversion | 9 (47.0%) | NI | 0 (0%) |
|  |  |  |  | H3N2 |  | 6 (32.0%) | NI | 0 (0%) |
| Winthrop 2019 | RA | Polyvalent pneumococcal polysaccharide (PPV23) | 5 | Total | Immunogenicity | 22 (71.0%) | 48 (67.0%) | NI |
|  |  | Tetanus |  |  |  | 16 (5.2%) | 28 (39.0%) | NI |

**QUESTION 8. What is the impact of JAKis use on the response to inactivated vaccines in patients with immune-mediated rheumatic diseases?**

| **Reference** | **Location** | **Journal** | **Design** | **Acronym** | **JAKIs use** | **Dosage JAKis** | **Inactivated vaccine** | **Follow-up** |
| --- | --- | --- | --- | --- | --- | --- | --- | --- |
| Mori et al., 2023 | Kumamoto, Japan | Mod Rheumatol | Cohort prospective | RA | tofacitinib, peficitinib or baricitinib | tofacitinib 5 mg twice daily, peficitinib 150 mg daily, or baricitinib 4 mg daily | 13-valent pneumococcal conjugate vaccine: single dose of vaccine (0.5 ml suspension) intramus cularly in the upper arm | 4 and 6 weeks |
| Zhilyaev et al., 2022 | Moscow, Russian | Ann. Rheum. Dis. (abstract) | Cohort retrospective - Moscow Unified Arthritis Registry (MUAR) | RA | targeted anti-inflammatory drugs (tDMARDs), biological or tofacitinib | NI | 23-valent pneumococcal polysaccharide vaccine (PPV23) and 13-valent pneumococcal conjugate vaccine (PCV13) | The duration of observation was 319 ± 198 days |
| Winthrop et al., 2021 | Arizona, USA | Ann. Rheum. Dis. (abstract) | FROM A PHASE 2 OPEN-LABEL EXTENSION STUDY | RA | Upadacitinib (UPA) | UPA 15mg once daily (QD) or 30mg QD and background MTX for ≥4 weeks | Prevnar 13® (Pneumococcal 13-valent Conjugate Vaccine [Diphtheria CRM197 Protein]; PCV13) | 4 and 12 weeks |
| Källmark et al., 2020 | Gothenburg, Sweden | Arthritis Rheum (abstract) | Cohort prospective | RA | JAKis | NI | novel subunit vaccine against HZ (HZ/su; Shingrix) composed of VZV glycoprotein E (gE) and adjuvant system AS01B -  All participants were immunized with two intramuscular doses of  50 mg HZ/su vaccine administered at least two months apart | 4 and 6 weeks |
| Winthrop et al., 2019 | Portland, USA | Arthritis Research & Therapy | long-term extension trial substudy | RA | baricitinib | 2-mg or 4-mg | pneumococcal and tetanus vaccine: single-dose 0.5-mL intramuscular injec tions of PCV-13 (Prevnar 13®, Wyeth Pharmaceuticals, a subsidiary of Pfizer, Inc.) and TTV (Boostrix®, GlaxoS mithKline Biologicals) | 5 and 12 weeks |
| Kaine et al., 2020 | China and Japan | Clinical Rheumatology | randomized, parallel-group, open-label study | RA | Tofacitinib | ORAL Sequel with tofacitinib 5 mg | Pneumococcal and influenza vaccines were adminis tered to all patients on day 8 | 43 days |
| Winthrop et al., 2016 | Portland, USA | Ann Rheum Dis | RCT (2 independent studies evalluating vaccine responses in relation to tofacitinib use) | RA | Tofacitinib | Tofacitinib 10 mg twice daily | Influenza and pneumococcal | 35 days |
| Venerito et al., 2023 | Bari, Italy | International Journal of Molecular Sciences | Cohort prospective | RA | 26 patients on JAKi at RZV baseline; in particular, 8 out of 52 (15.38%) were on Filgotinib (FIL), 7/52 (13.46%) on Baricitinib (BAR), 6/52 (11.54%) on Upadacitinib (UPA), and 5/52 (9.62%) on Tofacitinib (TOF). | NI | Adjuvanted Recombinant Zoster Vaccine | 3 months |
| Oren et al., 2008 | Israel | Annals of the Rheumatic Diseases | Case-control | RA |  |  | Influenza | 4 |
| Pons et al., 2022 | Spain | Arthritis & Rheumatology | Cohort | RA |  | 4,5 ± 3,2 | Herpes Zoster | 2 |
| Lukina et al., 2022 | Moscow, Russian | Annals of the Rheumatic Diseases | Abstract | RA | tofacitinib (TOFA) |  | 13-valent pneumococcal vaccine | 52 weeks |
| Stevens et al., 2020 | Boston | ACR Open Rheumatology | Cohort | RA and not specific | MTX, Prednisone, Tofacitinib, TNF inhibitors (adalimumab, certolizumab pegol, etanercept, golimumab, infliximab), other biologic therapy (abatacept, belimumab, tocilizumab, rituximab, sarilumab), other immunosuppressants (azathioprine, cyclophosphamide, mycophenolic acid, leflunomide) |  | Zoster recombinant adjuvanted (ZRA) | 12 weeks |

*Note: SS – Spondyloarthritis; RA - Rheumatoid Arthritis; SLE - Systemic Lupus Erythematosous ; SLC - Lupus Cutaneous; SS - Sjögren’s Syndrome; AO – Osteoarthrithis; AS - Ankylosing Spondylitis; PsA - Psoriatic arthitis, PsO – Psoriasis ; NI – not informated ; NA – Not applicable ; RCT – randomised clinical trial.*

*Vaccine response*

|  | **JAKi use group** |  |  | **Control group** |  |  |
| --- | --- | --- | --- | --- | --- | --- |
| **Reference** | **N follow-up** | **Type of serological response** | **Serological response (yes)** | **N follow-up** | **Type of serological response** | **Serological response (yes)** |
| Mori et al., 2023 | 43 | Positive antibody response JAKI group | 19 | 10 | Positive antibody response | 9 |
| Mori et al., 2023 | 43 | Positive antibody response MTX + JAKI | 12 | 10 | Positive antibody response | 9 |
| Winthrop et al., 2019 | 106 | achieved humoral response | 70 | NI | NI | NI |
| Winthrop et al., 2019 | 106 | achieved humoral response | 44 | NI | NI | NI |
| Winthrop et al., 2019 | 106 | achieved humoral response | 11 | NI | NI | NI |
| Winthrop et al., 2019 | 106 | achieved humoral response | 59 | NI | NI | NI |
| Winthrop et al., 2019 | 106 | achieved humoral response | 5 | NI | NI | NI |
| Winthrop et al., 2019 | 106 | achieved humoral response | 39 | NI | NI | NI |
| Winthrop et al., 2016 | 102 | achieving satisfactory humoral response | 46 | NI | NI | NI |
| Winthrop et al., 2016 | 102 | achieving satisfactory humoral response | 58 | NI | NI | NI |
| Oren et al., 2008 | 29 | Immunogenicity | 11 | NI | NI | NI |
| Oren et al., 2008 | 29 | Immunogenicity | 19 | NI | NI | NI |
| Oren et al., 2008 | 29 | Immunogenicity | 9 | NI | NI | NI |
| Oren et al., 2008 | 29 | Immunogenicity | 5 | NI | NI | NI |
| Oren et al., 2008 | 29 | Immunogenicity | 4 | NI | NI | NI |
| Oren et al., 2008 | 29 | Immunogenicity | 3 | NI | NI | NI |
| Stevens et al., 2020 | NI | NI | NI | 27 (6,7%) | NI | NI |
| Stevens et al., 2020 | NI | NI | NI | 23 (5,7%) | NI | NI |
| Stevens et al., 2020 | NI | NI | NI | 5 (2,3%) | NI | NI |
| Stevens et al., 2020 | NI | NI | NI | 1 (0,3%) | NI | NI |
| Stevens et al., 2020 | NI | NI | NI | 19 (5,0%) | NI | NI |
| Stevens et al., 2020 | NI | NI | NI | 9 (3,6%) | NI | NI |

**QUESTION 09. Is there a benefit to vaccine response by temporarily stopping methotrexate, mycophenolate, iJAKs before or after vaccination?**

| **Reference** | **Location** | **Journal** | **Design** | **Acronym** | **Drug evaluated** | **temporarily stopping methotrexate, mycophenolate, Ijak** | **Inactivated vaccine** | **Follow-up** |
| --- | --- | --- | --- | --- | --- | --- | --- | --- |
| Park et al., 2023 | Seoul, South Korea | Arthritis & Rheumatology | RCT | RA | MTX | discontinue MTX for 1 week or for 2 weeks after they received the quadrivalent 2021–2022 seasonal influenza vaccine containing H1N1, H3N2, B/Yamagata, and B/Victoria strains | Influenza vaccine | 1-2 weeks |
| Winthrop et al., 2016 | Portland, USA | Ann Rheum Dis | RCT | RA | Tofacitinib | tofacitinib was withdrawn at the time of randomisation for 1 week prior to receiving the influenza and pneumococcal vaccinations, and then resumed 1 week after vaccination | Influenza vaccine | 1-2 weeks |
| Lenfant et al., 2021 | Ohio | Rheumatology (Oxford) | Cohort | RA, Vasculitis, PMR, SLE, PsA, Inflammatory arthritis, Sjögren, SpA, Myositis, Scleroderma | GC: glucocorticoids; Jak-i: Jakinhibitors; Other biologic: abatacept, apremilast, belimumab, mepolizumab, rituximab, secukinumab, tocilizumab, ustekinumab; Other IS: other immunosuppressant, AZA: azathioprine, LEF: leflunomide, cyclosporine, MMF: mycophenolate mofetil, tacrolimus; RZV: recombinant zoster vaccine; TNF-i: TNF inhibitors. | NI | recombinant zoster vaccine (RZV) | 52 weeks |

*Note: SS – Spondyloarthritis; RA - Rheumatoid Arthritis; SLE - Systemic Lupus Erythematosous ; SLC - Lupus Cutaneous; SS - Sjögren’s Syndrome; AO – Osteoarthrithis; AS - Ankylosing Spondylitis; PsA - Psoriatic arthitis, PsO – Psoriasis ; NI – not informated ; NA – Not applicable ; RCT – randomised clinical trial.*

*Vaccine response*

|  | **Vaccine use with  temporarily stopping methotrexate, mycophenolate, iJAK** |  |  | **Vaccine use without  temporarily stopping methotrexate, mycophenolate, iJAK** |  |  |
| --- | --- | --- | --- | --- | --- | --- |
| **Reference** | **N follow-up** | **Stopping_response** | **Serological response (yes)** | **N follow-up** | **Response** | **Serological response (yes)** |
| Park et al., 2023 | 90 | Satisfactory vaccine response | 78 | 62 | Satisfactory vaccine response | NI |
| Winthrop et al., 2016 | 88 | Patients achieving satisfactory* humoral responses at 35 days after vaccination | 73 | 51 | Patients achieving satisfactory* humoral responses at 35 days after vaccination | NI |
| Lenfant et al., 2021 | 473 | Positive ANA - before vaccination | 96 | NI | positive ANA - before vaccination | NI |

**QUESTION 10 - Is there a difference in the immune response between conventional “tri- and tetravalent’’ and high-dose influenza vaccines in patients with immune-mediated rheumatic diseases (IMRD)?**

| **Table 1. Main characteristics of the included studies** | | | | | | | | | |
| --- | --- | --- | --- | --- | --- | --- | --- | --- | --- |
| **Reference** | **Local** | **Title** | **Journal** | **Study design** | **IMRD** | **conventional “tri- and tetravalent" use time** | **high-dose influenza vaccine use time** | **inactivated vaccine** | **Follow-up (weeks)** |
| Adler et al., 2012 | Switzerland, Europe | Protective effect of A/H1N1 vaccination in immune-mediated disease—a prospectively controlled vaccination study | Rheumatology | Cohort | MCTD, RA, SpA and VAS | Adjuvanted split influenza A/H1N1 vaccine and seasonal influenza vaccination with split inactivated influenza virus |  |  | 26 |
| Andrade et al., 2012 | Brazil, South America | Is the H1N1 influenza vaccine safe and effective in patients with SSc? | Rheumatology | Randomized clinical trial | SSc | Trivalent seasonal influenza vaccine |  |  | 3 |
| Assen et al., 2010 | Netherlands, Europe | Humoral Responses After Influenza Vaccination Are Severely Reduced in Patients With Rheumatoid Arthritis Treated With Rituximab | Arthritis & Rheumatism | Randomized clinical trial | RA | Trivalent influenza subunit vaccine |  |  | 52 |
| Borba et al., 2012 | Brazil, South America | Influenza A/H1N1 vaccination of patients with SLE: Can antimalarial drugs restore diminished response under immunosuppressive therapy? | Rheumatology (United Kingdom) | Cohort | SLE | Novel monovalent adjuvant-free vaccine |  |  | 3 |
| Bukhanova et al., 2018 | Russian, Asia | Safety and efficacy of a trivalent, inactivated influenza vaccine in patients with rheumatic diseases (preliminary results) | Clinical and Experimental Rheumatology | Randomized clinical trial | AIRD |  |  | Inactivated split-vaccine against influenza | 26 |
| Bukhanova et al., 2019 (1) | Russian, Asia | Assessment of efficacy, safety and immunogenicity of a trivalent split-virus influenza vaccine in patients with rheumatic diseases | Annals of the Rheumatic Diseases | Randomized clinical trial | RA and SpA |  |  | Inactivated trivalent influenza vaccine | 26 |
| Colmegna et al., 2018 | Canada, North America | Efficacy of High-Dose Versus Standard-Dose Influenza Vaccine in Seropositive Rheumatoid Arthritis Patients | American College of Rheumatology | Randomized clinical trial | RA |  | High-dose trivalent inactivated influenza vaccine (HD-TIV) |  | 73 |
| Colmegna et al., 2019 | Canada, North America | Immunogenicity and safety of high-dose versus standard-dose inactivated influenza vaccine in rheumatoid arthritis patients: a randomised, double-blind, active-comparator trial | Lancet Rheumatol | Randomized clinical trial | RA |  | High-dose trivalent inactivated influenza vaccine (HD-TIV) |  | 73 |
| Del Porto et al., 2006 | Italy, Europe | Influenza vaccine administration in patients with systemic lupus erythematosus and rheumatoid arthritis. Safety and immunogenicity | Vaccine | Randomized clinical trial | SLE and RA | Adjuvanted split influenza A/H1N1 vaccine |  |  | 26 |
| Fomin et al., 2005 | Israel, Asia | Vaccination against influenza in rheumatoid arthritis: the effect of disease modifying drugs, including TNF alfa blockers | Annals of the Rheumatic Diseases | Randomized clinical trial | RA |  |  | Inactivated split-vaccine against influenza | 13 |
| França et al., 2012 | Brazil, South America | TNF blockers show distinct patterns of immune response to the pandemic influenza A H1N1 vaccine in inflammatory arthritis patients | Rheumatology (Oxford) | Cohort | RA, AS and PsA |  |  | Inactivated split-vaccine against influenza | NI |
| Holvast et al., 2009 | Poland, Europe | Studies of Cell-Mediated Immune Responses to Influenza Vaccination in Systemic Lupus Erythematosus | Arthritis & Rheumatism | Cohort | SLE | Trivalent influenza subunit vaccine |  |  | 8 |
| Kaine et al., 2007 | USA, North America | Immune responses following administration of influenza and pneumococcal vaccines to patients with rheumatoid arthritis receiving adalimumab | The Journal of Rheumatology | Randomized clinical trial | RA | Trivalent influenza subunit vaccine |  |  | 60 |
| Kapetanovic et al., 2007 | Sweden, Europe | Influenza vaccination as model for testing immune modulation induced by anti-TNF and methotrexate therapy in rheumatoid arthritis patients | Rheumatology (Oxford) | Randomized clinical trial | RA |  |  | Inactivated trivalent influenza vaccine | 6 |
| Kapetanovic et al., 2013 | Sweden, Europe | Rituximab and abatacept but not tocilizumab impair antibody response to pneumococcal conjugate vaccine in patients with rheumatoid arthritis | Arthritis Research & Therapy | Cohort | RA and SpA | Novel monovalent adjuvant-free vaccine |  |  | 17 |
| Kivitz et al., 2014 | USA, North America | Vaccine responses in patients with rheumatoid arthritis treated with certolizumab pegol: results from a single-blind randomized phase IV trial. | The Journal of Rheumatology | Randomized clinical trial | RA | Trivalent influenza virus and the 23-valent pneumococcal vaccines |  |  | 6 |
| Kogure et al., 2014 | Japan, Asia | Investigation of clinical characteristics as predictive factors for the humoral immune response to the influenza vaccine in patients with rheumatoid arthritis. | Clinical Rheumatology | Randomized clinical trial | RA | Trivalent influenza subunit vaccine |  |  | 4 |
| Kumar et al., 2020 | Denmark, Europe | Influenza vaccination compliance and response in autoimmune inflammatory rheumatic disease patients: A cohort study | Annals of the Rheumatic Diseases | Randomized clinical trial | AIRD | Trivalent seasonal influenza vaccine |  |  | 17 |
| Lakota et al., 2015 | Slovenia, Europe | The influence of seasonal influenza vaccination on immunogenicity in patients with rheumatoid arthritis | Scientific Abstracts | Randomized clinical trial | RA | Trivalent seasonal influenza vaccine |  |  | 26 |
| Litinsky et al., 2012 | Israel, Asia | Vaccination against influenza in patients with systemic sclerosis | Clinical and Experimental Rheumatology | Cohort | SSc | Trivalent influenza subunit vaccine |  |  | 6 |
| Miossi et al., 2013 | Brazil, South America | Immunogenicity of influenza H1N1 vaccination in mixed connective tissue disease: effect of disease and therapy | Clinical Sciences | Cohort | MCTD | Trivalent seasonal influenza vaccine |  |  | 3 |
| Mori et al., 2012 | Japan, Asia | Impact of tocilizumab therapy on antibody response to influenza vaccine in patients with rheumatoid arthritis | Clinical and epidemiological research | Randomized clinical trial | RA |  |  | Inactivated trivalent influenza vaccine | 6 |
| Oren et al., 2008 | Israel, Asia | Vaccination against influenza in patients with rheumatoid arthritis: the effect of rituximab on the humoral response. | Annals of the Rheumatic Diseases | Randomized clinical trial | RA |  |  | Inactivated split-vaccine against influenza | 13 |
| Park et al, 2018 | South Korea, Asia | Impact of temporary methotrexate discontinuation for 2 weeks on immunogenicity of seasonal influenza vaccination in patients with rheumatoid arthritis: A randomized clinical trial | Annals of the Rheumatic Diseases | Randomized clinical trial | RA | Quadrivalent seasonal influenza vaccine |  |  | 4 |
| Park et al, 2022 | South Korea, Asia | A Multicenter, Prospective, Randomized, Parallel-Group Trial on the Effects of Temporary Methotrexate Discontinuation for One Week Versus Two Weeks on Seasonal Influenza Vaccination in Patients With Rheumatoid Arthritis | Arthritis & Rheumatology | Randomized clinical trial | RA | Quadrivalent seasonal influenza vaccine |  |  | 16 |
| Park et al., 2017 | South Korea, Asia | Effect of methotrexate discontinuation on efficacy of seasonal influenza vaccination in patients with rheumatoid arthritis: a randomized clinical trial | Clinical and Epidemiology Research | Randomized clinical trial | RA | Trivalent seasonal influenza vaccine |  |  | 20 |
| Ribeiro et al., 2011 | Brazil, South America | Reduced seroprotection after pandemic H1N1 influenza adjuvant-free vaccination in patients with rheumatoid arthritis: Implications for clinical practice | Annals of the Rheumatic Diseases | Randomized clinical trial | RA | Novel monovalent adjuvant-free vaccine |  |  | 3 |
| Ribeiro et al., 2013 | Brazil, South America | Abatacept and reduced immune response to pandemic 2009 influenza A/H1N1 vaccination in patients with rheumatoid arthritis. | Arthritis Care & Research | Randomized clinical trial | RA | Novel monovalent adjuvant-free vaccine |  |  | 3 |
| Richi et al., 2021 | Spain, Europe | Serological response to influenza vaccine in patients with autoimmune inflammatory diseases: Results of RIER study. | Medicina Clínica | Cohort | MCTD, RA, SpA and PsA |  |  | Inactivated trivalent influenza vaccine | 4 |
| Vagelli et al., 2017 | Italy, Europe | Influenza and meningococcal c vaccinations in a cohort of patients with autoimmune rheumatic diseases: Adherence, safety and immunogenicity | Annals of the Rheumatic Diseases | Randomized clinical trial | SLE, RA, SSc, pSS, BD and Dermatomyositis | Influenza and meningococcal C vaccine |  |  | 52 |
| Westra et al., 2014 | Netherlands, Europe | Rituximab impairs immunoglobulin (Ig)M and IgG (subclass) responses after influenza vaccination in rheumatoid arthritis patients | Clinical & Experimental Immunology | Cohort | RA | Trivalent influenza subunit vaccine |  |  | 4 |
| Winthrop et al., 2016 | USA, North America | The effect of tofacitinib on pneumococcal and influenza vaccine responses in rheumatoid arthritis | Annals of the Rheumatic Diseases | Randomized clinical trial | RA | Trivalent influenza virus and the 23-valent pneumococcal vaccines |  |  | 9 |
| **Legend**: Diseases: Immune-mediated rheumatic disease (IMRD), Rheumatoid arthritis (RA), Systemic lupus erythematosus (SLE), Vasculitis (VAS), Mixed connective tissue disease (MCTD), Spondyloarthritis (SpA), Autoimmune inflammatory rheumatic diseases (AIRD), Systemic sclerosis (SSc), Ankylosing spondylitis (AS), Primary Sjögren’s Syndrome (pSS), Behçet disease (BD) and Psoriatic arthritis (PsA). | | | | | | | | | |

**QUESTION 11. Is there a difference in the immune response between conventional “tri- and tetravalent) and high-dose Hepatitis B Vaccines in patients with immune-mediated rheumatic diseases (IMRD)?**

| **Table 1. Main characteristics of the included studies** | | | | | | | | |
| --- | --- | --- | --- | --- | --- | --- | --- | --- |
| **Reference** | **Local** | **Title** | **Journal** | **Study design** | **IMRD** | **Drugs use** | **High-dose Hepatitis B Vaccines** | **Follow-up (weeks)** |
| Intongkam et al., 2018 | Thailand, Asia | Efficacy and Safety of Hepatitis B Vaccination in Rheumatoid Arthritis Patients Receiving Disease-Modifying Antirheumatic Drugs and/or Biologics Therapy | Journal of Clinical Rheumatology | Randomized clinical trial | RA | csDMARDs, bDMARDs and corticosteroids | Hepatitis B virus (HBV) vaccine | 24 |
| Romão et al., 2020 | Portugal, Europe | Poor response to hepatitis b vaccination in rheumatic patients treated with biologic therapy-implications for clinical practice | Annals of the Rheumatic Diseases | Randomized clinical trial | RA | csDMARDs, bDMARDs and corticosteroids | Hepatitis B virus (HBV) vaccine | 26 |
| De Juanes et al., 2012 | Spain, Europe | Vaccination against hepatitis B in patients with psoriatic arthritis (PA). Preliminary results with double dose schedule | Annals of the Rheumatic Diseases | Randomized clinical trial | PsA | csDMARDs and corticosteroids | Recombinant hepatitis B vaccine | NI |
| Intongkam et al., 2016 | Thailand, Asia | The short-term and long-term of humeral immune responses to hepatitis b vaccination are impaired in rheumatoid arthritis patients receiving conventional and biological disease modifying anti-rheumatic drugs | Annals of the Rheumatic Diseases | Randomized clinical trial | RA | csDMARDs and bDMARDs | Recombinant hepatitis B vaccine | 52 |
| Richi et al., 2020 | Spain, Europe | Evaluation of the immune response to hepatitis B vaccine in patients on biological therapy: results of the RIER cohort study | Clinical Rheumatology | Randomized clinical trial | AIRDs and PsA | csDMARDs, bDMARDs and corticosteroids | Hepatitis B virus (HBV) vaccine | 4 |
| Aydin et al., 2020 | Turkey, Asia | An evaluation of the efficacy of high-dose hepatitis b vaccine in patients using biological agents | Viral Hepatitis Journal | Randomized clinical trial | AS, PsA and RA | csDMARDs, bDMARDs and corticosteroids | Hepatitis B virus (HBV) vaccine | 26 |
| Solay and Eser (2019) | Turkey, Asia | High Dose Hepatitis B Vaccine Is Not Effective In Patients Using Immunomodulatory Drugs: A Pilot Study | Human Vaccines & Immunotherapeutics | Randomized clinical trial | PsA, RA, BD and AS | bDMARDs | Hepatitis B virus (HBV) vaccine | 24 |
| Kuruma et al., 2007 | Brazil, South America | Safety and efficacy of hepatitis B vaccine in systemic lupus erythematosus | Lupus | Randomized clinical trial | SLE | csDMARDs and corticosteroids | Recombinant hepatitis B vaccine | 4 |
| Elkayam et al., 2002 | Israel, Asia | Safety and efficacy of vaccination against hepatitis B in patients with rheumatoid arthritis | Annals of the Rheumatic Diseases | Randomized clinical trial | RA | csDMARDs and corticosteroids | Hepatitis B virus (HBV) vaccine | 30 |
| **Legend**: Diseases: Immune-mediated rheumatic disease (IMRD), Autoimmune inflammatory rheumatic diseases (AIRD), Ankylosing spondylitis (AS), Psoriatic arthritis (PsA), Rheumatoid arthritis (RA) and Systemic lupus erythematosus (SLE); Medications: Biologic Disease-Modifying Antirheumatic Drugs(bDMARDs), Conventional synthetic DMARDs (csDMARDs) and Targeted synthetic DMARDs (tsDMARDs). | | | | | | | | |
